# The ceRNA Crosstalk between mRNAs and lncRNAs in Diabetes Myocardial Infarction

**DOI:** 10.1155/2022/4283534

**Published:** 2022-05-09

**Authors:** Yun Zhou, Chengjun Zhou, Lilong Wei, Chengwu Han, Yongtong Cao

**Affiliations:** Department of Clinical Laboratory, China-Japan Friendship Hospital, Beijing 100029, China

## Abstract

Competitive endogenous RNA regulation suggests an intricate network of all transcriptional RNAs that have the function of repressing miRNA function and regulating mRNA expression. Today, the specific ceRNA regulatory mechanisms of lncRNA–miRNA–mRNA in patients who have diabetes mellitus (DM) and myocardial infarction (MI) are still unknown. Two data sets, GSE34198 and GSE112690, were rooted in the Gene Expression Omnibus database to search for changes of lncRNA, miRNA, and mRNA in MI patients with diabetes. Weighted gene correlation network analysis (WGCNA) was used to identify the modules related to the development of diabetes in patients with MI. Target genes of miRNAs were predicted using miRWalk, TargetScan, mirDB, RNA22, and miRanda. Then, functional and enrichment analyses were performed to build the lncRNA–miRNA–mRNA interaction network. We built ceRNA regulatory networks with three lncRNAs, two miRNAs, and nine mRNAs. Differentially expressed genes enriched in biological process, including neutrophil activation, refer to immune response and positive system of defense feedback. Besides, there is significant enrichment in molecular function of calcium toll−like receptor binding, icosanoid binding, RAGE receptor binding, and arachidonic acid binding. Kyoto Encyclopedia of Genes and Genomes (KEGG) analysis enriched differentially expressed genes (DEGs) in pathways that were well known in MI, indicating inflammation and immune response. Pathways associated with diabetes were also significantly enriched. We confirmed significantly altered lncRNA, miRNA, and mRNA in MI patients with diabetes, which might serve as biomarkers for the progress and development of diabetic cardiovascular diseases. We constructed a ceRNA regulatory network of lncRNA–miRNA–mRNA, which will enable us to understand the novel molecular mechanisms included in the initiation, progression, and interaction between DM and MI, laying the foundation for clinical diagnosis and treatment.

## 1. Introduction

Morbidity and mortality from cardiovascular disease (CVD) are extremely high [1, 2]. Moreover, CVD is also the leading cause of death in urban and rural areas, overshadowing cancer and other diseases in China. The incidence of CVD and mortality among Chinese patients also remains high, and more importantly, the upward trend is projected to continue into the next decade [3]. The increasing burden of CVD has become a significant public health problem. Myocardial infarction (MI) is particularly severe, resulting in progressive heart failure and cardiovascular mortality, and diabetes mellitus (DM) is a major risk factor for CVD [4, 5]. Patients with diabetes are twice as likely to have an MI as healthy people. Diabetes and impaired glucose tolerance are quite common among people with MI (seen in almost two-thirds of patients) and are associated with a twofold increase in mortality rate compared to those with normoglycemia [6]. In addition, there have been numerous studies proving that DM is a strong prognostic biomarker in patients with established coronary artery disease [7–9]. Follow-up studies have shown that 39.5% of patients with type 2 DM (T2DM) died within 2 years of their first MI, compared with 28.5% of nondiabetic patients with MI only [10]. However, mechanisms that can explain the complex association between diabetes and MI remain mysterious. Therefore, to improve the poor outcome of CVDs, effort should be made toward discovering a potential pathogenesis and exploiting novel medication targets and therapeutic strategies.

Noncoding RNAs, including miRNA and lncRNA, are essential for the regulation of RNA expression [11]. miRNAs have the ability to negatively regulate the expression of target genes [12], and lncRNAs have no or little function in encoding proteins that affect many biological processes [13]. lncRNAs are important in regulating basic biological processes and may cause diseases if aberrant expression occurs [14, 15]. However, little is known about whether or how lncRNA is related to DM and MI.

lncRNAs can compete with target genes for miRNA response elements and weaken the repressive effect of miRNAs on target genes [16, 17]. Thus, they can indirectly regulate the expression of target genes and influence the onset and progression of disease [18]. Nevertheless, the association between ceRNAs and diabetic MI remains unclear. In this manuscript, we summarize the regulatory roles of lncRNAs, miRNAs, and mRNAs and discuss a regulatory network built of ceRNA.

## 2. Materials and Methods

### 2.1. Data Acquisition

miRNAs and lncRNAs in MI patients with or without DM were collected from two microarray data sets: GSE34198 (Czech Republic: 15 patients with DM and 34 controls) and GSE112690 (USA: 85 patients with DM and 244 controls), which are based on the platform of GPL6102 Illumina human-6 v2.0 expression beadchip and GPL24804 Biomark high-throughput human miRNA RT-qPCR assay (MGH, Boston, MA, USA). The GEO database is an international public repository.

### 2.2. Data Processing and Differential Expression Analysis

After quantile normalization to ensure differentially expressed genes (DEGs), probe identifiers of GSE34198 were transformed into gene symbols, and the single expression value of the gene was calculated. To screen related differentially expressed lncRNAs (DELncs), the probe identifiers of GSE34198 were blasted against the GENCODE (https://www.gencodegenes.org/human/) long noncoding RNA database to be reannotated [19]. GEO series matrix files of GSE112690 were downloaded and the quantile was normalized to obtain the differentially expressed miRNAs (DEMics). Subsequently, the differential expression analysis was executed with the threshold of *P* value < 0.05 using Student's *t*-test in R software.

### 2.3. Gene Ontology

Gene ontology is a channel for performing gene annotation, which classifies genes into three categories [20, 21]. A gene database was used to assign genomes to specific pathway maps of molecular interactions, reactions, and relationship networks [22]. The gene ontology (GO) function annotation and enrichment analysis of DEGs were performed using the R package clusterProfiler [23], and a *P* value < 0.05 was considered significant.

### 2.4. Weighted Gene Correlation Network Analysis

We proceeded to conduct gene correlation analysis of DEGs and DELncs to verify the key genes and lncRNAs associated with diabetes in MI patients. The purpose of the WGCNA is to find coexpressed gene modules and explore any relevance between gene networks and related phenotypes. First, hierarchical cluster analysis is performed with the hclust function. The modules are constructed with soft thresholds filtered by the pickSoftThreshold function. Candidate powers (1–30) are used to test the average degree of connectivity of different patterns and their independence. If the degree of independence is >0.8, a suitable power value is selected. For the present study, coexpression networks were constructed [24] with the smallest module size set at 30, and each pattern was labeled with a different color. The relationship of functional modules and concerned phenotype (in our case, diabetes) was also evaluated.

The hub genes were also described in this study as genes that were most strongly related to disease. A module that was highly related to diabetes was explored, and a regulatory network of DEGs and DELncs in the module was constructed and visualized by Cytoscape software [25], which was used because it can visualize complex networks.

### 2.5. miRNA Target Prediction

The construction of a ceRNA background network requires a large number of lncRNAs, miRNAs, and mRNAs, along with their interactions. Target genes of differentially expressed miRNAs found in GSE112690 were predicted with the help of the miRWalk [26], TargetScan [27], miRDB [28], RNA22 [29], and miRanda databases [30]. The predicted target genes were intersected with DEGs found in GSE34198 to be further explored.

### 2.6. lncRNA–miRNA–mRNA ceRNA Network

The relationship between DELncs and DEMics was calculated with the TargetScan 6.1 algorithm [31]. Then, according to ceRNA theory, gene pairs with opposite expressions were screened out based on the expression of differentially expressed miRNAs and differentially expressed lncRNAs for construction of the background network.

## 3. Results

### 3.1. Identification of DEGs, DELncs, and DEMics

**Figure 1:**
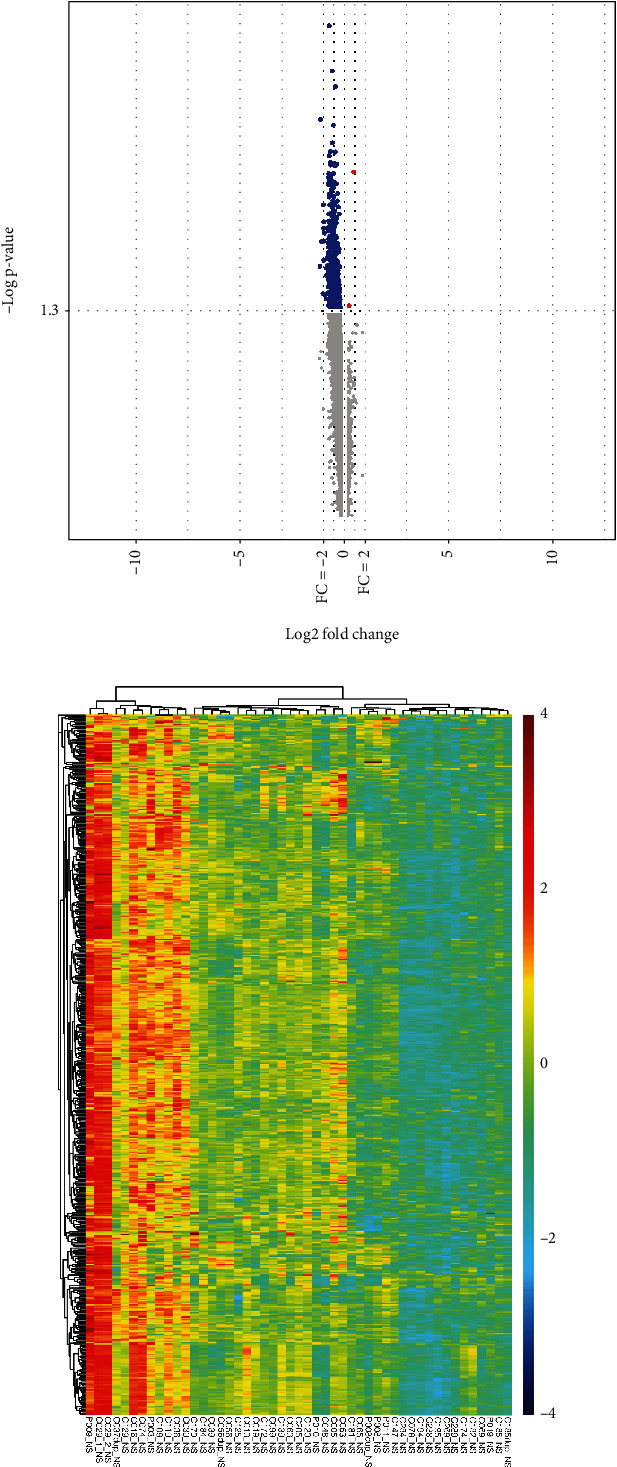
Overview of differentially expressed genes and lncRNAs. (a) Volcano plot of differentially expressed genes and lncRNAs between patients with DM and controls. Red dots represent significantly upregulated genes and lncRNAs, while blue dots represent those that are significantly downregulated. (b) Heat map of differentially expressed genes and lncRNAs between patients with DM and controls. Red blocks represent significantly upregulated genes and lncRNAs, while blue blocks represent those that are significantly downregulated.

To screen DEGs, lncRNAs, and miRNAs, the series matrix files of GSE34198 and GSE112690 were downloaded from the GEO database. Subsequently, Student's *t*-test was executed to analyze the DEGs and DELncs between controls and patients with DM in the GSE34198 data set. Meanwhile, the differential expression analysis of DEMics between controls and patients with DM was implemented with the threshold of *P* value < 0.05 by Student's *t*-test in R software. As shown in Figures 1, 499 DEGs and 23 DELncs were identified from GSE34198 microarray data using cutoff criteria of *P* < 0.05 (detailed information about the DEGs and DELncs can be found in Table S1), while four DEMics were found in GSE112690, using the same threshold (Table S2).

### 3.2. KEGG Pathway Enrichment Analysis

**Figure 2:**
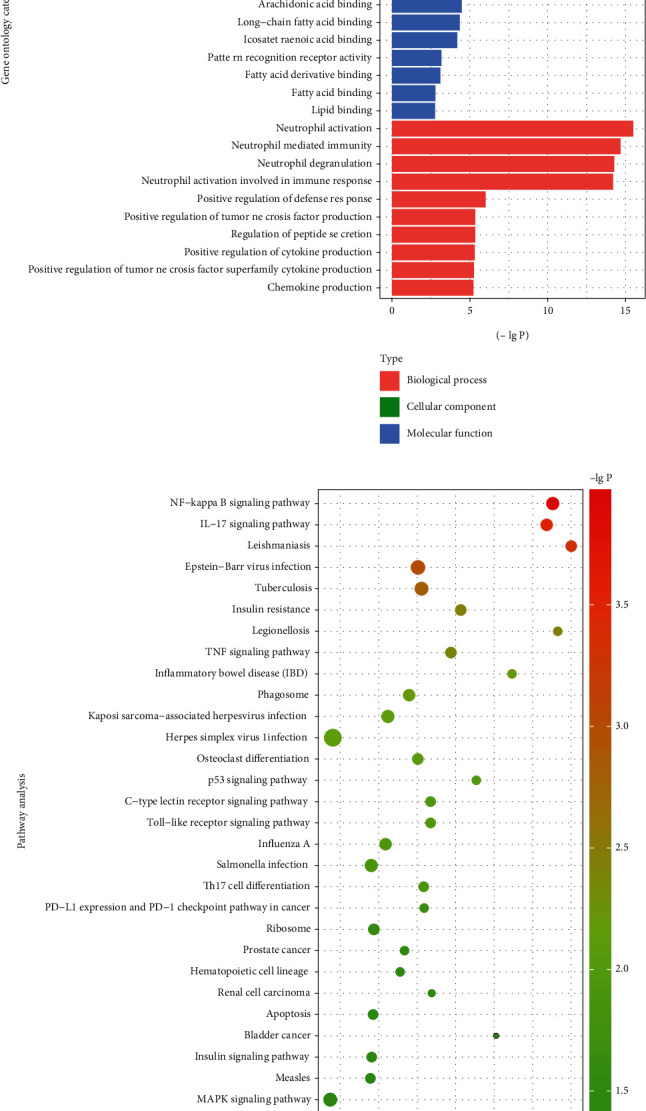
Gene ontology enrichment and KEGG pathway annotation of DEGs. (a) Bar plot showing the top 10 enriched gene ontology terms in each category. The *x*-axis represents the negative logarithmic of different *P* values, and *y*-axis represents GO terms. (b) *x*-axis representing an enriched factor, and the *y*-axis represents KEGG pathway terms. Sizes of the circles indicate gene counts, and the color of the circles represents different adjusted *P* values.

DEGs were subjected to GO functional annotation and Kyoto Encyclopedia of Genes and Genomes (KEGG) pathway enrichment analysis, with a *P* value < 0.05 deemed significant. The results showed that DEGs mainly enriched biological processes (Figure 2(a)) in specific granules, secretory granule lumen, cytoplasmic vesicle lumen, vesicle lumen, and secretory granule membrane (Figure 2(a)). In addition, these DEGs showed enrichment in the molecular functions of calcium toll−like receptor binding, icosanoid binding, RAGE receptor binding, and arachidonic acid binding (Figure 2(a), Table S3).

KEGG analysis enriched DEGs in pathways of leishmaniasis, Epstein–Barr virus infection, and tuberculosis, among others (Figure 2(b)). Several pathways are well known in MI that indicate inflammation and immune response. Additionally, pathways related to diabetes were also enriched, including the insulin resistance pathway and insulin signaling pathway (Table S4).

### 3.3. Weighted Gene Correlation Network Analysis (WGCNA)

**Figure 3:**
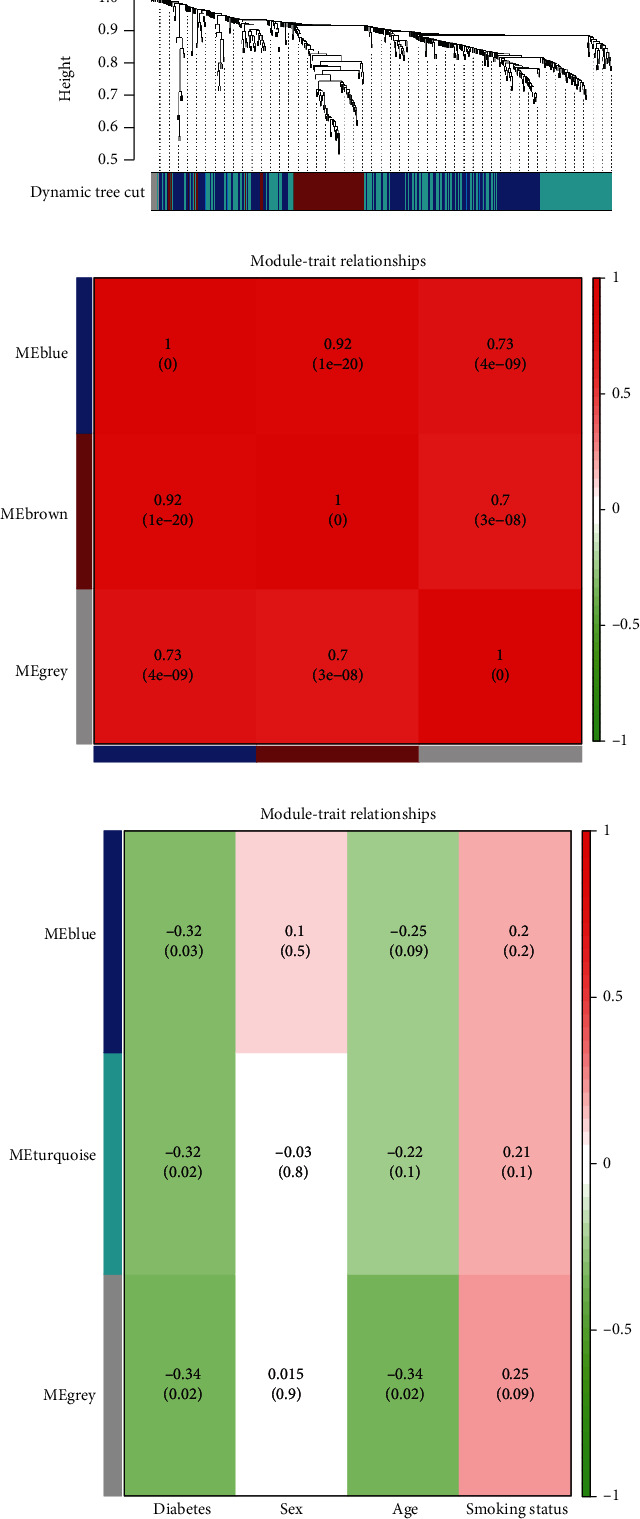
WGCNA used for the identification of key modules associated with MI patients with diabetes. (a) Dendrogram obtained by clustering the dissimilarities based on consensus topological overlap with the corresponding module colors indicated by the color row. (b) The relationships between different modules, where red indicates a positive correlation. (c) Heat map of the correlation between module eigengenes and clinical traits (diabetes, gender, age, and smoking status). Each cell contains the correlation coefficient and *P* value.

**Figure 4:**
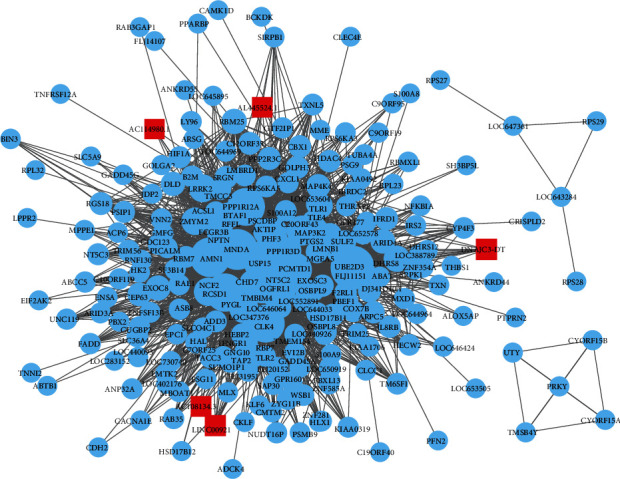
DM-related lncRNA–mRNA regulatory network. For the DM-related lncRNA–mRNA regulatory network in module MEturquoise, where the red squares stand in for hub lncRNAs, while blue circles indicate DEGs in the module.

To determine the key elements most closely related to MI patients who also have diabetes, WGCNA was performed using the expression profile of DEGs and DELncs that were identified earlier. All three modules were determined (Figure 3(a); detailed mRNAs and lncRNAs in each module can be found in Table S5). The blue module was negatively correlated with DM (correlation coefficient = −0.32, *P* = 0.03; Figure 3(b)), and the turquoise module was also negatively correlated with DM (correlation coefficient = −0.32, *P* = 0.02; Figure 3(b)). At the same time, the gray module was negatively correlated with DM (correlation coefficient = −0.34, *P* = 0.02; Figure 3(b)). All three modules were positively correlated, and each of them was positively correlated with each other (Figure 3(c)). Hub nodes have a key role in maintaining the overall connectivity of the network. Therefore, the top 5% were selected as hubs. We chose differentially expressed genes and lncRNAs in MEturquoise to construct the correlation network (Figure 4), with 10 DEGs and 5 DELncs identified as hub genes and lncRNAs (UBE2D3, MNDA, AMN1, TMBIM4, RCSD1, CLK4, PPP1R12A, USP15, NT5C2, PCMTD1, DNAJC3-DT, LINC00921, AC108134.3, AL445524.1, and AC114980.1, Table S6).

### 3.4. lncRNA–miRNA–mRNA ceRNA Network

**Figure 5:**
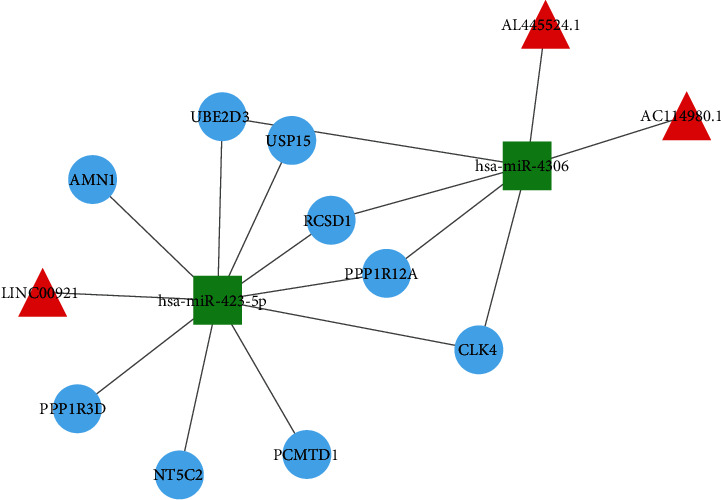
The lncRNA–miRNA–mRNA ceRNA regulatory network. Three lncRNAs, two miRNAs, and nine mRNAs were included in the network, with red triangles representing lncRNAs, green squares indicating miRNAs, and the blue nodes representing mRNAs, respectively. In addition, the edges represent the interactions between lncRNAs, miRNAs, and mRNAs.

To survey the regulation of ceRNAs and identify DM-related lncRNAs in MI patients, we built a network. As shown in Figure 5, three lncRNAs, two miRNAs, and nine mRNAs were included in the network, and the red, green, and blue nodes represent lncRNAs, miRNAs, and mRNAs, respectively. The edges represent the interactions between lncRNAs, miRNAs, and mRNAs. The lncRNA acts as a natural miRNA sponge to suppress the function of miRNAs, and the expression of lncRNA–miRNA and miRNA–mRNA were negatively correlated. Notably, the key DEGs and DELncs related to MI patients with DM that we had identified in WGCNA were also found in the ceRNA regulatory network, suggesting they also correlate with the DEMics we screened from GSE112690. In addition, based on ceRNA mechanisms, we identified LINC00921, AL445524.1, and AC114980.1 as candidate lncRNA biomarkers for diabetic MI.

## 4. Discussion

Salmena et al. proposed how mRNAs and lncRNAs communicate with each other [16]. lncRNAs regulate mRNA expression posttranscriptionally by competing for miRNAs [32]. Dysregulated lncRNAs and mRNAs (sequences complementary to lncRNAs) affect the expression levels of target mRNAs through interference of miRNAs, together with target mRNAs harbored by lncRNAs called miRNA response elements (MREs) [33, 34]. This endogenous RNA exchange forms a large-scale regulatory network across the transcriptome that plays a crucial role in the physiological and pathological processes of disease [35]. Next, we integrated genome-wide lncRNA, mRNA, and miRNA expression profiling data and experimentally validated miRNA–target interactions to construct a regulatory network in diabetic MI patients. Three candidate lncRNA biomarkers were identified for the comorbidity of MI and DM.

Diabetes affects the heart, including metabolic disturbances, abnormalities in subcellular components, microvascular damage, and autonomic dysfunction [36, 37]. The myocardium shows local inflammation, coronary endothelial dysfunction, necrosis, apoptosis, and autophagy [38, 39]. The hub miRNA (miR-4306) is downregulated in coronary artery disease (CAD), and miR-4306 is an independent poor prognostic factor in CAD [40].

miR-4306 noticeably inhibited the human monocyte-derived macrophages (HMDMs) in vitro and reduced the number of macrophage cells in cardiac tissue in MI mice. Another hub miRNA (miR-423-5p) was found to have significantly higher expression in the vitreous of the eyes with proliferative diabetic retinopathy (PDR) and was proved to be related to angiogenesis and fibrosis [41].

Regarding the hub genes we found in the ceRNA network, studies had proved that the ubiquitin-conjugating enzyme UBE2D3 was involved in RNA processing and splicing and was associated with increased fasting plasma glucose [42]. PPP1R3G is downregulated in the liver by fasting and increased by feeding, and it plays an important role in the control of postprandial glucose homeostasis through its regulation of hepatic gluconeogenesis during the fasting–feeding transition [43]. The 5′-nucleotidase cytosolic II (NT5C2) variants were reported to be nominally associated with coronary heart disease (CHD) susceptibility in the subgroups of males and with hypertension and diabetes [44]. Ubiquitin-specific protease 15 (USP15) was found to be a potential target of miR-26a and mediated the proautophagic and cardioprotective effects of miR-26a against ischemic injury [45]. Protein phosphatase 1 regulatory subunit 12A (PPP1R12A) was reported to be a member of the insulin-stimulated IRS1 signaling complex, and the interaction of PPP1R12A with IRS1 was dependent on Akt and mTOR/raptor activation [46]. To summarize, all the genes and miRNAs we identified in the ceRNA network were associated with either DM complication or MI side effects, indicating the analysis we conducted was solid and effective. However, no research has been found to elucidate that the lncRNA biomarkers we found are strongly related to the comorbidity of DM and MI. In the days to come, molecular biology methods, including qPCR, luciferase reporter systems, and co-immunoprecipitation assays could be helpful to substantiate our findings, thus unraveling the molecular mechanisms of the mutual effects that DM and MI bring to bear during the complex and development of certain diseases.

## 5. Conclusion

In conclusion, our study provides more comprehensive material for the ceRNA link between mRNAs and lncRNAs in diabetic MI by constructing competing endogenous RNA networks, and three candidate lncRNA biomarkers for the comorbidity of MI and DM were identified. The findings will improve comprehension of the molecular mechanisms underlying the pathology of MI and DM from a ceRNA perspective and provide potential therapeutic targets for the treatment of diabetic MI in clinical practice.

## Data Availability

The data sets used and analyzed during the current study are available from the corresponding author on reasonable request.

## Supplementary Materials

Supplementary 1 Table S1: the differentially expressed mRNAs and lncRNAs identified from the GEO data set.

Supplementary 2 Table S2: the differentially expressed miRNAs found in GSE112690.

Supplementary 3 Table S3: the gene ontology (GO) term enrichment for differentially expressed genes.

Supplementary 4 Table S4: the Kyoto Encyclopedia of Genes and Genomes (KEGG) pathway enrichment for differentially expressed genes.

Supplementary 5 Table S5: detailed mRNAs and lncRNAs in each WGCNA module.

Supplementary 6 Table S6: hub genes and lncRNAs in module MEturquoise in the lncRNA–mRNA regulatory network.
