# Supplementary material for: The ceRNA Crosstalk between mRNAs and lncRNAs in Diabetes Myocardial Infarction
Source: Dis Markers. 2022 May 9;2022:4283534. doi: 10.1155/2022/4283534 (PMC9112177; doi:10.1155/2022/4283534)
Supplement: Supplementary 1 — Table S1: the differentially expressed mRNAs and lncRNAs identified from the GEO data set. [file 4283534.f1.pdf]

Probe_Set_ID	pvalues	foldchange	C037dup_NS	C065_NS	C039_NS	C172_NS
ILMN_1656233	0.02067925	0.76199224	13.7036841	13.0411745	13.0510934	13.5524037
ILMN_1657996	0.01228782	0.67051593	7.43785692	7.34683814	7.47090611	8.30702271
ILMN_1660357	0.00584948	0.77720835	7.26775395	7.09902899	7.31435484	7.22266258
ILMN_1661461	0.03976483	0.68392708	7.44159054	7.10474446	7.43366639	7.28064644
ILMN_1676480	0.02162666	0.61039278	7.23192387	6.45502385	6.97131247	6.91709515
ILMN_1683250	0.04801097	0.68730819	8.62550977	7.49188195	7.26785318	7.97275353
ILMN_1693740	0.03252467	0.71305284	7.61514553	6.63606817	6.60157886	7.44597825
ILMN_1723320	0.0428202	0.85177271	6.51793562	6.25826432	6.40240533	6.03845396
ILMN_1729421	0.01202769	0.63930722	7.68633874	6.64663359	6.93139822	7.44336961
ILMN_1745931	0.01211735	0.84047449	6.5670921	6.50545018	6.58701538	6.45780636
ILMN_1775444	0.0106002	0.68187302	7.64737568	7.27598292	7.24616293	7.51524962
ILMN_1790412	0.02851932	0.71899209	8.96304171	7.61597312	8.27021122	8.04716809
ILMN_1805064	0.04246256	0.74537749	7.18331679	7.83866945	7.68779627	7.30602066
ILMN_1824139	0.00428839	0.67076339	7.71141713	7.38400058	7.38833594	7.46585124
ILMN_1859584	0.02593932	0.69738358	7.18638368	7.49227302	6.72933116	7.47230174
ILMN_1871457	0.02965886	0.79713812	7.8366898	7.11647289	7.28801243	7.13889432
ILMN_1872261	0.00999095	0.6856316	7.31914913	6.84113281	7.70162599	7.58450332
ILMN_1880280	0.03697789	0.72634022	7.05665397	7.74490042	8.05174187	7.90382884
ILMN_1882711	0.04294172	0.84968705	6.32027472	5.91176348	5.92304694	5.58340896
ILMN_1883279	0.01724662	0.75453837	7.4066251	6.44986936	6.49701192	6.47549237
ILMN_1893633	0.02324721	0.68083968	10.3882778	7.56405801	7.54147612	7.36834494
ILMN_1906626	0.02112377	0.74175133	6.14869601	5.88647113	6.20757742	6.08286201
ILMN_1907042	0.03927828	0.68218712	8.89345899	7.58878358	7.9369759	8.08806419
ILMN_1343293	0.00750878	0.66242	12.3508113	11.5458622	12.0386094	12.7061349
ILMN_1651228	0.03776029	0.66225561	14.1056467	11.2697964	11.75231	10.7298213
ILMN_1651498	0.02549122	0.78438022	6.59430714	6.18458836	6.04371546	6.15202153
ILMN_1651850	0.03282375	0.7767501	14.7832254	13.3997719	13.8235971	13.556233
ILMN_1651964	0.04852667	0.71739939	9.0101826	7.70146103	8.06361134	8.18855473
ILMN_1652003	0.04791611	0.62799015	8.40382712	6.79568904	7.39130976	7.56933387
ILMN_1652128	0.02817528	0.61236662	10.837944	9.04808848	9.91694189	9.29708019
ILMN_1652230	0.04509359	0.76359332	15.0209387	13.5785142	14.2323025	13.4932225
ILMN_1652631	0.02670336	0.77529925	13.1149118	12.331675	12.5771855	12.9421941
ILMN_1652736	0.02924844	0.78563991	6.67141086	6.1041173	6.24870864	6.19892509
ILMN_1653412	0.01434316	0.68862792	8.36079881	8.36456193	7.96140107	8.68841958
ILMN_1653567	0.02585267	0.82025215	5.91685709	5.79004451	5.64227991	5.38906736
ILMN_1653871	0.03086367	0.63611829	12.104775	11.0471907	12.4754039	13.1240622
ILMN_1654320	0.02303977	0.64999991	9.99473669	9.22478361	8.96419269	9.46728356
ILMN_1654516	0.03216181	0.70498793	9.86409528	8.44437263	9.1849388	9.21667069
ILMN_1656057	0.0347521	0.79740085	6.72905783	6.08320379	6.4875579	6.08351592
ILMN_1656393	0.04451557	0.77687322	7.29273943	7.25941562	6.88684397	7.15061959
ILMN_1656676	0.02964941	0.62291349	10.210664	8.61307411	9.4863765	9.51077801
ILMN_1656961	0.00776051	0.69043306	8.85311084	8.21329916	8.40966333	8.24648742
ILMN_1657317	0.00602072	0.73106502	7.37486995	6.7106136	6.9742597	6.71784775
ILMN_1657398	0.00077359	0.60388716	7.24446945	7.61500939	7.6547291	7.74330895
ILMN_1657497	0.01952165	0.78023424	6.6960286	6.32854497	6.58704194	6.38050782
ILMN_1657515	0.01974223	0.59706498	9.53176723	8.90733686	9.4100557	8.81252454
ILMN_1658798	0.00670908	0.69646692	7.00671206	7.02054332	7.67780631	7.38454461

ILMN_1658921	0.03618521	0.7269563	7.5559327	7.27876073	7.63971549	7.48024008
ILMN_1658957	0.04264963	0.72975832	7.48149512	6.83563864	7.66009076	6.17522358
ILMN_1659047	0.02466382	0.73791088	9.61691912	9.86937237	10.0319507	10.6300423
ILMN_1659437	0.04117212	0.71987015	11.6992289	10.6251755	10.634527	10.9777464
ILMN_1659490	0.04721526	0.72227408	7.47577558	7.54706032	7.49987139	7.39536704
ILMN_1659878	0.03389557	0.79246669	6.76722574	6.38453684	6.92886772	6.7015948
ILMN_1660498	0.03931401	0.76945244	15.1541995	14.0454235	14.5304789	14.108629
ILMN_1660549	0.04279546	0.63991796	8.02541478	6.78804878	7.22575018	7.19876031
ILMN_1660942	0.0465267	0.82710653	6.44044913	6.48011273	6.54106919	5.98766535
ILMN_1661196	0.02226929	0.68657612	9.06924594	7.90449253	9.15974043	9.21391443
ILMN_1661466	0.03229566	0.80559606	6.47850221	6.03020805	6.25508769	6.02769859
ILMN_1661636	0.04686079	0.69838424	8.83556064	7.48790112	7.91218578	7.89300538
ILMN_1662617	0.02640941	0.65874607	10.3694872	9.08038639	9.56154629	10.0893181
ILMN_1662846	0.02745936	0.64437652	7.9109215	7.08725491	7.83651245	8.52299378
ILMN_1662865	0.03529648	0.6034407	8.1428295	6.87772585	7.59898148	7.82817648
ILMN_1663422	0.0180202	0.70012101	9.55626348	8.9931572	9.48736113	10.1940957
ILMN_1663437	0.02170087	0.71354447	7.41761373	6.60580039	7.02776227	7.19153304
ILMN_1664047	0.02361668	0.74655969	6.60804379	6.67496056	6.36725468	6.38335835
ILMN_1664577	0.037362	0.67480187	8.4658847	7.13993704	7.57521021	7.31875519
ILMN_1664698	0.04028014	0.76298462	9.53053938	8.78447225	8.96663782	9.66757693
ILMN_1664706	0.01919351	0.7133548	7.60836646	6.66423251	6.9766088	7.00836923
ILMN_1664750	0.02655861	0.74802412	12.4272311	11.533793	11.80144	11.868103
ILMN_1664802	0.04079438	0.67119674	9.82810442	7.80492025	8.21374436	8.60514242
ILMN_1665384	0.04194668	0.75782178	8.79350772	8.13139245	8.21582661	8.42048872
ILMN_1665557	0.03538694	0.63500425	9.52823823	8.23921949	8.7851778	9.45492354
ILMN_1665781	0.03568531	0.80682594	8.98231056	8.54793637	9.09906082	8.67311894
ILMN_1665982	0.04896871	0.68812735	8.96534766	6.85300133	7.53244243	7.32287095
ILMN_1666376	0.03540638	0.68503491	9.77168638	8.25459404	8.14652896	8.28733515
ILMN_1666480	0.027722	0.70586064	10.2218638	8.65748603	9.2187739	9.11513705
ILMN_1667162	0.04594816	0.67131193	7.03456459	6.46622789	6.68274042	6.69403327
ILMN_1667319	0.02547702	0.6475955	8.8749454	7.62472309	8.21737469	8.35337887
ILMN_1667460	0.02678717	0.61610888	9.4654607	7.35594039	8.30781738	8.31908499
ILMN_1667561	0.0219473	0.60215467	9.8788559	8.63012367	9.45172441	9.79712925
ILMN_1667970	0.02028456	0.83352828	7.18997969	6.60507158	6.96744992	6.56620934
ILMN_1667985	0.00745955	0.61890288	9.07832723	8.44782133	8.83600269	8.73765702
ILMN_1668379	0.03742933	0.83385299	6.51412037	6.16203859	6.16410402	5.78168442
ILMN_1669177	0.00761851	0.7061977	7.74948131	7.15156763	7.32843169	6.95784613
ILMN_1669608	0.02841213	0.7278004	8.16330148	7.51359733	8.03551779	7.59103233
ILMN_1669790	0.03302285	0.65122668	14.1203238	12.6130108	13.2279161	12.9432307
ILMN_1670130	0.04589559	0.69867587	10.7084632	9.17976307	9.91975213	10.3619953
ILMN_1670821	0.03840955	0.59309786	6.21393474	5.98161546	6.1392243	5.61110833
ILMN_1671516	0.02129075	0.61296651	9.80903172	8.39377269	8.09806525	8.73488707
ILMN_1671933	0.04752139	0.77511537	7.35592013	6.63650152	7.18428426	7.16527183
ILMN_1672004	0.03164279	0.70753517	9.18318253	8.55053594	9.71048756	9.34397404
ILMN_1672661	0.02848531	0.65065818	8.99319882	8.40974987	8.01056279	8.50864098
ILMN_1672988	0.02024643	0.64795249	7.2746344	6.36948585	6.83161507	7.18308251
ILMN_1673113	0.04356854	0.61302364	9.3328292	7.5454997	8.10165795	8.85850668
ILMN_1674590	0.04267189	0.72809209	6.67035033	6.16165574	6.42486953	6.81141708

ILMN_1674650	0.04467613	0.76477245	9.42299507	7.56194622	7.44027536	7.34041717
ILMN_1675117	0.03899492	0.62746484	9.49966275	7.90644089	8.82165984	9.22093402
ILMN_1675258	0.0356111	0.66140331	8.00817648	7.18796762	8.41678022	7.24395273
ILMN_1675424	0.03630798	0.66895473	9.14615776	8.50191439	9.14611499	9.06661396
ILMN_1675939	0.03846476	0.70361537	12.5907412	11.3882687	12.2310567	12.767015
ILMN_1676016	0.0291055	0.80943544	6.53161555	6.89967715	6.66906646	5.85337069
ILMN_1676665	0.03123601	0.7577298	7.79741187	8.58538135	9.00285889	8.57076119
ILMN_1676737	0.04696222	0.7752066	6.54357244	6.46922128	6.584805	6.40941521
ILMN_1676767	0.03943497	0.5385914	8.81501593	7.96751967	8.64946888	9.85626833
ILMN_1677262	0.04607949	0.59397151	13.0813721	11.143461	10.5218105	11.3686729
ILMN_1677511	0.04606087	0.6409039	8.75300342	7.18606272	7.91506132	8.58777602
ILMN_1677843	0.03395397	0.71547996	11.5580299	10.7458163	10.5124778	10.7892687
ILMN_1678170	0.00792294	0.59601979	11.107374	10.1451692	11.3156908	11.6339143
ILMN_1678690	0.00149587	0.66288364	6.81206433	6.6216963	6.69241743	6.4700305
ILMN_1678707	0.03991451	0.69297866	7.94437825	7.00703288	7.74309685	6.69047297
ILMN_1678757	0.0270338	0.65826664	9.53813887	9.67539272	9.25927696	9.70147351
ILMN_1678799	0.03407905	0.75742717	6.24211238	6.00109085	6.62423486	6.14296925
ILMN_1678939	0.04313604	0.70311962	13.6567891	12.8136283	13.1236217	13.6593471
ILMN_1679666	0.01862454	0.79574732	6.26521277	6.14769724	6.15639844	5.71676131
ILMN_1679920	0.02931304	0.64358563	12.5785319	12.1815912	10.1079794	9.67077679
ILMN_1680279	0.03350039	0.62837153	11.1475745	10.3148664	10.7502799	10.8158964
ILMN_1680314	0.00864226	0.64756847	11.5294159	10.8388636	11.4845823	12.1286562
ILMN_1681130	0.03091266	0.76590747	8.06707129	6.84268473	7.22700842	6.97950522
ILMN_1681683	0.01489961	0.57023733	8.86477858	6.93739501	7.15100943	7.92991347
ILMN_1681888	0.03851427	0.76608665	6.8810442	6.84150019	7.26723333	6.59561465
ILMN_1682165	0.03172151	0.65292393	11.5317952	10.0068212	10.2825545	10.9064585
ILMN_1682443	0.03615179	0.6495768	12.0345985	10.6096986	10.6898207	11.2135107
ILMN_1682699	0.01361885	0.64028818	8.96425797	7.10367807	7.54076272	8.11589731
ILMN_1682757	0.02993952	0.55798965	7.72561575	7.17229949	7.40774872	8.8658916
ILMN_1683494	0.02904661	0.66051778	10.8387013	10.6752849	11.2707719	11.3963827
ILMN_1683959	0.01705165	0.76547399	7.0288277	6.44984279	6.76743896	6.48298087
ILMN_1684054	0.04932318	0.80571873	7.48399308	7.1718213	8.09495966	7.92856812
ILMN_1684585	0.02691987	0.56307844	11.6869141	10.665645	10.6923897	12.2690942
ILMN_1685312	0.02211059	0.64373813	7.99478109	7.02790379	7.54299933	7.3956181
ILMN_1685493	0.04494352	0.65174709	7.61396328	6.95989416	7.87800676	7.78979314
ILMN_1685628	0.01240387	0.65756989	12.7642351	11.972711	11.8486926	12.3651282
ILMN_1686116	0.03519944	0.58272003	8.38021688	7.77662319	9.02748833	8.53320045
ILMN_1686464	0.04760029	0.69823557	8.02752012	7.23859868	7.76616115	8.34873966
ILMN_1686750	0.0433905	0.72327785	12.4417122	11.2331241	11.7441863	11.9574496
ILMN_1686862	0.02000637	0.73257137	7.64631605	7.26415826	7.04105348	7.26510023
ILMN_1686981	0.02359098	0.59853707	8.05828619	6.24122851	7.04651071	7.01958962
ILMN_1687057	0.049469	0.70480187	8.28477136	6.76967522	7.00687696	7.04246815
ILMN_1687384	0.02690716	0.68006639	11.2582492	8.90552811	10.1533443	10.2830004
ILMN_1687805	0.02092321	0.73151486	14.7458376	13.8233016	13.6738313	13.6149468
ILMN_1688490	0.01551608	0.67404311	8.33775346	8.40535338	8.18426591	8.45136156
ILMN_1688580	0.03985474	0.68048617	11.0949499	9.20124863	11.1220605	10.6720867
ILMN_1688666	0.03725174	0.74410908	7.25755568	6.94669811	6.57239555	6.85940806
ILMN_1689004	0.00188777	0.74093787	6.9097803	6.17444677	6.49805137	6.74734309

ILMN_1689955	0.04109953	0.82527325	7.02382309	6.59511259	6.59133788	6.35107085
ILMN_1690114	0.02584932	0.66870912	9.77636032	9.03347201	8.86500695	9.11015023
ILMN_1691364	0.04228554	0.70764278	12.4410254	12.5133881	10.8801521	11.0762872
ILMN_1691428	0.01810185	0.72831086	13.9460811	13.5855518	13.5894077	13.8858422
ILMN_1691508	0.03608453	0.73901795	8.55917095	8.92282342	9.1850952	9.12166923
ILMN_1691574	0.03275834	0.60818227	8.67455916	6.6846238	7.0105986	7.82965615
ILMN_1691881	0.02573877	0.8133981	6.15282429	6.75532959	6.80457248	6.52060438
ILMN_1692145	0.04103045	0.62621689	12.1852781	11.4104487	11.8847524	11.840444
ILMN_1693394	0.03766476	0.76046797	10.4487905	9.70959685	10.0051122	10.0533585
ILMN_1693494	0.04680144	0.79860694	7.26344856	6.75371952	6.63814018	6.46762609
ILMN_1693789	0.02384987	0.61698714	11.7712732	10.8879098	11.0538261	11.0916939
ILMN_1694075	0.01160109	0.72305822	8.34758328	7.75085904	7.82534871	8.92650979
ILMN_1694399	0.0230504	0.70607854	9.44927041	8.85414748	8.82315799	9.0826052
ILMN_1694584	0.03041394	0.78982682	6.4479309	5.88388836	6.28900538	6.08748427
ILMN_1694742	0.04359298	0.79662118	15.0813332	14.0430247	14.5658036	13.9275835
ILMN_1695435	0.01054341	0.69671593	7.08459795	6.69807537	6.9881325	7.72902315
ILMN_1695468	0.04652008	0.77244158	6.6781029	6.6877312	7.30754237	7.40977193
ILMN_1695853	0.03060682	0.65524188	8.06743544	6.83843499	7.07149454	7.12160708
ILMN_1696027	0.04350034	0.67986662	9.66067713	9.13720302	9.30851242	9.47469764
ILMN_1696162	0.04043779	0.84911952	6.14416305	6.04886239	6.44034979	6.16887666
ILMN_1696187	0.03766499	0.62357425	11.9791647	10.0492646	11.196816	12.2423039
ILMN_1696466	0.02183896	0.72959243	8.77798971	8.13963832	8.74248898	9.47740514
ILMN_1696704	0.02722924	0.72265839	7.94818674	7.38870789	7.84383251	7.78866679
ILMN_1697304	0.04993868	0.76825905	7.12891921	6.75346682	6.92669094	6.71483622
ILMN_1697377	0.04463471	0.69850338	6.68453544	6.14996656	6.27927256	6.31012196
ILMN_1698213	0.0193828	0.80446305	7.81481828	6.95995094	7.70470877	6.83527987
ILMN_1699015	0.0240332	0.76984958	14.7089109	14.0060543	14.5236624	14.3798136
ILMN_1699331	0.02644333	0.60266054	6.83494881	6.57904812	6.24671799	5.79081686
ILMN_1699383	0.03358542	0.81936599	7.19239047	6.67053304	6.77972855	7.03828253
ILMN_1699687	0.02189761	0.75701865	6.45707515	6.13667148	6.1831931	5.9308868
ILMN_1700182	0.03012897	0.71409936	13.1976412	11.9265755	12.1409675	12.7420622
ILMN_1700610	0.02743074	0.69400317	8.39584256	7.86277372	7.92270332	8.71672448
ILMN_1700896	0.01363125	0.50642127	8.42860011	7.17834217	8.17501354	8.00350442
ILMN_1701242	0.0253789	0.74418201	6.4530001	6.15891481	6.18696499	5.81842624
ILMN_1701747	0.04934732	0.83021473	6.69189424	6.20652504	6.26426543	6.07102337
ILMN_1701957	0.02126631	0.58892087	11.675393	10.9970778	11.4726015	11.6803037
ILMN_1702168	0.03741472	0.73249678	9.17733532	7.98855745	8.07178787	8.3837436
ILMN_1703720	0.00762214	0.68139965	10.674157	9.15202924	9.70243695	9.80523392
ILMN_1704385	0.01810021	0.51213732	11.2140027	8.97206478	9.77500641	10.396258
ILMN_1704476	0.04609397	0.81323076	6.64697337	6.27236515	6.34911754	5.92940116
ILMN_1705477	0.02914213	0.77925703	6.51225565	6.4156642	6.70260214	6.41179603
ILMN_1705605	0.02532555	0.62850884	8.39276562	7.3199656	8.35252903	6.89336828
ILMN_1706502	0.01556267	0.58390627	9.9963379	10.8837583	9.55903344	10.035272
ILMN_1708485	0.01864396	0.70205561	7.72367187	7.07135617	7.33528762	6.65473053
ILMN_1708841	0.04742674	0.67021081	10.1869388	8.47797597	9.24214492	9.33322514
ILMN_1708936	0.03337196	0.70874144	9.77830124	8.09165541	8.41219552	8.44806756
ILMN_1709164	0.02941149	0.75703754	7.68162636	7.94043106	7.71373293	7.40607657
ILMN_1709707	0.04238071	0.85383488	6.23196994	5.95561364	6.17467866	5.79357106

ILMN_1710362	0.04157112	0.76920064	7.79037326	6.98816431	7.17351525	6.85153293
ILMN_1710974	0.03533472	0.63323745	12.943121	10.8873318	11.9649004	12.8217624
ILMN_1711073	0.01405347	0.6411207	10.9501265	10.1850414	9.93271806	10.448742
ILMN_1711156	0.03433604	0.76010835	14.5006907	13.921099	13.9466596	13.9328339
ILMN_1711617	0.01600477	0.66135292	13.008043	11.4844045	11.8463919	12.668585
ILMN_1711729	0.03453758	0.60814527	12.2679457	10.4152749	10.4558686	10.2735963
ILMN_1712389	0.01597405	0.69113487	12.9883481	11.8465439	12.3997706	12.4690539
ILMN_1712596	0.03288592	0.72797317	7.31390881	7.06657252	7.11042484	6.97708297
ILMN_1712688	0.02426725	0.79722532	7.56396805	6.7717628	6.91859256	7.17882218
ILMN_1712918	0.03314652	0.62320921	8.23012374	7.97656011	8.91504488	9.65829201
ILMN_1713319	0.0194869	0.65622699	9.4046624	8.72892149	8.69518096	8.98705221
ILMN_1713636	0.01729628	0.6907968	12.8429727	12.9072978	12.840846	13.1198204
ILMN_1713706	0.01842635	0.67755647	9.40308789	9.33017339	9.3746033	9.91578051
ILMN_1714065	0.01035268	0.73820948	6.76346071	5.94913545	6.4788746	6.00271895
ILMN_1714418	0.00910934	0.60836911	7.3269166	7.74961136	8.93145939	7.89667389
ILMN_1714848	0.00569427	0.62554357	7.37641021	6.57746931	6.712908	6.95465697
ILMN_1714991	0.0422315	0.71365708	13.33423	12.367012	12.9937824	13.7108968
ILMN_1715693	0.03416076	0.83468095	6.23954545	6.29292141	6.03669893	5.62217748
ILMN_1715809	0.03716245	0.75966037	8.22476287	7.54548811	7.97384486	8.00551676
ILMN_1716465	0.01729694	0.64472115	9.6502991	8.23297823	9.04851875	8.61037747
ILMN_1717371	0.04602444	0.82876408	6.69975705	6.40713824	7.10840632	6.31085304
ILMN_1718303	0.03887466	0.49428875	6.6763591	6.34442428	6.32386686	6.50420246
ILMN_1719951	0.04653004	0.60741284	9.04208779	7.40041411	7.93357087	7.96686244
ILMN_1719988	0.03731506	0.72172595	7.26644925	6.73449151	6.94726288	7.66706114
ILMN_1720604	0.03548573	0.75934736	7.7214212	6.89778617	7.32006853	6.92360084
ILMN_1721116	0.03411519	0.68364283	10.0016404	9.77312157	10.4331285	11.5624175
ILMN_1721204	0.00791158	0.64737784	10.1758938	9.65516271	10.3062736	10.1711956
ILMN_1721349	0.03914856	0.5993326	10.9021769	10.048367	10.2729073	10.318745
ILMN_1721648	0.04615408	0.70733795	9.34233629	7.71428262	8.45275397	8.63114103
ILMN_1721729	0.0320432	0.62937111	8.94360579	8.02801268	8.13329328	8.22948218
ILMN_1722076	0.04703175	0.67202513	12.0845759	11.1363619	11.3079248	11.9621922
ILMN_1722223	0.04278418	0.78316468	6.79129706	6.22355094	6.23781856	6.01812605
ILMN_1723079	0.02970603	0.72043097	9.42156058	8.60740689	9.20456136	9.96539615
ILMN_1723486	0.00912968	0.64900163	8.63381116	7.86020675	8.58298044	8.92498615
ILMN_1723909	0.02017026	0.67179385	7.31473277	6.86509729	7.05863146	7.30631602
ILMN_1724315	0.02762385	0.80674242	6.97842218	6.42657306	6.66762961	6.53338191
ILMN_1724407	0.03429979	0.70724607	10.2648308	8.56658458	8.99849679	8.9574811
ILMN_1724533	0.01351725	0.59918915	11.5842297	10.7403281	10.4145732	11.1693463
ILMN_1725427	0.03136878	0.77197894	14.9948409	14.138759	14.1347817	14.6419095
ILMN_1726289	0.01747537	0.59825303	10.777626	9.96441842	10.6607046	11.2136644
ILMN_1726636	0.01297901	0.76907172	6.81861669	6.52236088	6.66187926	6.56383065
ILMN_1726786	0.01713798	0.70672295	9.57843949	9.34323747	9.44228418	9.84673556
ILMN_1727603	0.04412871	0.81207953	7.46742867	7.20633651	7.56505637	7.01267146
ILMN_1728639	0.03972765	0.73634149	13.9531825	13.1609396	13.4442009	14.2925661
ILMN_1728677	0.01055091	0.6364999	8.18943369	8.67686575	9.50302607	9.73853801
ILMN_1728724	0.01527998	0.6232623	9.58361592	9.64983907	9.91338806	9.68721487
ILMN_1728844	0.04727955	0.77639162	7.0063272	6.46018507	6.48630731	6.78151971
ILMN_1729294	0.04638838	0.70887944	10.9886705	9.49222254	10.2418962	10.235252

ILMN_1729659	0.01151918	0.70426537	6.95742364	6.24194522	7.02798338	7.02049887
ILMN_1729801	0.02480952	0.80433254	15.0076686	14.63653	14.6822611	14.4996871
ILMN_1730454	0.02587184	0.57417077	10.6717224	9.67630585	9.50798012	11.5757841
ILMN_1730678	0.02061662	0.7051953	11.9694675	11.1794364	11.1308324	11.3685834
ILMN_1730928	0.0048553	0.63050483	8.64566204	8.17274002	9.2939183	9.56826549
ILMN_1731048	0.02388277	0.50621945	10.0515543	7.73021673	8.27441869	8.97588665
ILMN_1731203	0.03803663	0.75827104	7.60273523	7.45376942	7.1340864	7.43155026
ILMN_1731546	0.04360587	0.69795296	12.5705687	11.9784774	12.2777494	10.9767381
ILMN_1731785	0.0458795	0.67462806	6.49153856	5.98718555	6.4046002	6.46669367
ILMN_1732311	0.03794346	0.77945233	6.72030036	7.15487379	6.81274849	6.72603828
ILMN_1733983	0.03516943	0.77609426	6.5135307	6.32947974	6.67017751	5.84673425
ILMN_1734991	0.02888959	0.74080227	6.81523726	7.35116275	7.88445567	6.88736198
ILMN_1735014	0.04129974	0.72928513	12.9149378	12.5280712	12.4517131	12.6030122
ILMN_1735506	0.02313855	0.66811802	9.02996714	9.28016875	8.86833304	8.15707454
ILMN_1735955	0.02614024	0.67721495	9.30644476	7.76441052	7.9938668	7.97083813
ILMN_1736008	0.04987902	0.63545161	10.7293735	9.51853868	9.7997066	10.1669652
ILMN_1736190	0.04827008	0.62863899	11.4841749	9.77053357	10.8636001	11.1764312
ILMN_1736982	0.01215593	0.69087927	6.42304093	7.07989114	7.24433624	6.65986464
ILMN_1737426	0.04791158	0.64576947	10.5681652	8.82056225	9.41667658	9.91593957
ILMN_1737878	0.04495929	0.81087537	6.74534491	6.58660098	6.58718209	6.49259413
ILMN_1738243	0.03038466	0.7416694	14.8957499	13.8117399	14.4019301	13.8207185
ILMN_1738482	0.03217564	0.71381727	8.91391203	8.34023779	8.11964875	8.61209332
ILMN_1738643	0.04149091	0.75593989	6.80506411	6.07832504	6.29717564	5.94869452
ILMN_1738821	0.04391898	0.8091662	7.77800812	6.95790648	7.3819478	6.98258409
ILMN_1739263	0.03678171	0.68662298	14.5238553	11.9281242	12.8762403	12.2481325
ILMN_1739283	0.04562368	0.73384391	9.02205066	9.26282611	9.33432782	9.00768663
ILMN_1739423	0.01894898	0.75456789	6.83405256	6.33794082	6.32998985	6.1475375
ILMN_1739587	0.008502	0.64481903	6.31246896	6.09842482	6.10652923	5.77442153
ILMN_1739622	0.03797193	0.61741953	9.24863445	7.40932389	8.22104764	8.46171517
ILMN_1739876	0.04014082	0.72778106	9.75641838	9.07589706	8.54862928	8.54217214
ILMN_1740165	0.02940505	0.71813807	8.84310701	9.15167848	8.9225737	8.90827102
ILMN_1741073	0.0350871	0.80687698	6.40386469	6.17685431	6.68323747	6.59278635
ILMN_1741917	0.0169043	0.684229	9.06561324	8.44459212	9.0453941	9.25286787
ILMN_1743034	0.01881818	0.69390438	7.27053081	6.96984005	7.05088692	7.71537244
ILMN_1743281	0.04631418	0.67846131	8.95011055	7.42542657	7.82634319	8.26883718
ILMN_1743476	0.01661299	0.73435808	7.30736021	6.8927128	6.93506955	7.13057674
ILMN_1746020	0.04993035	0.70883883	6.55361639	6.22185273	6.50586077	6.69179236
ILMN_1746276	0.01501639	0.65630005	6.86862627	6.60747147	7.03580643	6.60559804
ILMN_1746577	0.01566441	0.68593279	10.4187961	9.41689103	9.40040387	10.1043118
ILMN_1746864	0.04432744	0.6138112	11.4387295	9.77456685	10.3434145	10.7783794
ILMN_1746919	0.04003091	0.74508699	6.9707812	6.33112296	6.35555178	6.58105442
ILMN_1747047	0.04730674	0.80481102	6.74913063	6.44879542	6.65745994	6.07110341
ILMN_1747205	0.04360287	0.76509833	7.57503166	6.78453408	7.22149819	7.62429248
ILMN_1748090	0.03450901	0.6557916	6.86961852	6.32885459	6.61155927	6.79204322
ILMN_1748366	0.04668742	0.81750259	6.21450156	5.98624322	5.960527	6.10563639
ILMN_1748767	0.02574605	0.64144703	13.006595	11.2294059	11.6937413	12.7783731
ILMN_1748915	0.02617672	0.44861085	10.229636	8.17540983	9.48881784	10.3402351
ILMN_1749006	0.04308302	0.70484262	9.94282969	8.55647578	9.32009268	9.1181427

ILMN_1749673	0.03098645	0.74057841	8.80394317	8.01529908	9.19996527	8.83149854
ILMN_1750093	0.0168406	0.73019352	11.6221754	11.3162549	11.0506126	11.2037383
ILMN_1750880	0.02748177	0.72560601	7.15769557	6.30049168	6.5476565	6.81551907
ILMN_1750961	0.04949418	0.72044766	8.91982422	7.94429332	9.08190706	9.50828325
ILMN_1751051	0.03975061	0.70579569	8.2967061	7.39952831	7.50965705	7.84244086
ILMN_1751351	0.0484634	0.69265059	6.63487943	6.20903395	6.16232808	5.93183337
ILMN_1751749	0.01836375	0.72815944	7.49206307	6.83477188	7.55572108	6.84845769
ILMN_1753712	0.02948522	0.74102814	10.6286943	9.41774621	10.0152018	9.76632715
ILMN_1753819	0.03810533	0.66379162	9.49695302	8.09531209	9.20304401	9.31801105
ILMN_1754395	0.04776421	0.7677589	7.63522692	6.64545381	7.1334505	6.49317112
ILMN_1754859	0.02630922	0.76672095	7.48236429	6.9799533	6.8394797	7.33306051
ILMN_1755049	0.02884803	0.76637773	6.58276222	6.06265861	6.34809783	5.83403865
ILMN_1755077	0.03531818	0.64369729	11.2539332	9.68949434	10.0911802	10.5068758
ILMN_1755114	0.01355935	0.68312006	8.60732218	7.9335809	7.82491802	8.15421914
ILMN_1755115	0.03674114	0.64243278	12.1857817	7.91256005	9.73690434	9.02826248
ILMN_1756506	0.04857105	0.7225517	6.24985166	6.014089	6.02256667	5.56074919
ILMN_1756998	0.04439977	0.69757131	6.31149676	6.29930255	6.70550107	6.44828516
ILMN_1758418	0.02016829	0.63914595	11.6766192	11.3393573	10.45165	11.2998233
ILMN_1758658	0.01579283	0.69136037	9.66447253	8.20156868	8.62305879	9.09252539
ILMN_1759208	0.02449679	0.66571621	12.4151206	11.5770573	11.450586	12.5226132
ILMN_1759250	0.04647064	1.15609374	6.80355641	6.72584066	7.02740622	6.0807943
ILMN_1759613	0.03611932	0.81729371	6.37595794	6.05350911	6.0540338	5.72891448
ILMN_1759872	0.04013308	0.75798727	14.753037	13.8070147	14.0099231	14.2815613
ILMN_1760347	0.01976178	0.75067419	14.282615	13.9134635	14.4107442	14.4307067
ILMN_1760509	0.00654443	1.34180301	11.8703767	10.2775338	10.3447153	10.1866962
ILMN_1760542	0.00489019	0.73703635	6.50661416	6.39981845	6.7099316	6.38301941
ILMN_1760779	0.04561215	0.7412942	7.80284526	7.07855433	7.44762961	7.57773622
ILMN_1761411	0.04186005	0.78271634	7.61079011	6.82749768	7.04842584	7.14557862
ILMN_1763260	0.04728466	0.67986491	7.66554413	7.33723297	8.30787975	8.2825005
ILMN_1763408	0.024028	0.74325947	6.64057408	6.12464969	6.51443239	6.39697495
ILMN_1763452	0.04738204	0.72732674	12.5519687	11.4474382	12.26113	12.8108287
ILMN_1763809	0.02689206	0.55733105	9.44020454	8.31856179	10.6778403	10.9230667
ILMN_1763837	0.03015834	0.6262042	10.0331105	9.18101979	9.63646766	9.64657401
ILMN_1764082	0.03456126	0.75384766	8.61045868	7.91116306	8.25892166	8.36132754
ILMN_1764396	0.01846108	0.58395347	9.51877549	7.82007299	7.63533504	7.96641605
ILMN_1764415	0.0185249	0.73911887	8.13214084	7.05868665	7.2207256	7.28851822
ILMN_1765061	0.03218534	0.70733821	8.08643933	7.41101857	7.08047859	7.28220718
ILMN_1765122	0.04646311	0.65451904	9.08672566	8.46016344	8.68726124	9.09746174
ILMN_1766176	0.04185669	0.75392774	6.5435064	6.86885959	7.42948792	6.78799003
ILMN_1767809	0.03398026	0.68931984	7.93066875	6.96557306	7.6838417	8.16027231
ILMN_1768117	0.04019261	0.68370785	10.4770894	9.11796693	9.50521391	9.64032267
ILMN_1768394	0.03999826	0.62248965	12.7110915	10.9597339	11.1697885	12.3137934
ILMN_1769705	0.03683845	0.76314354	11.5833066	10.8959547	11.6125055	12.1217899
ILMN_1769734	0.03159924	0.58294769	9.69140016	9.20710976	8.70641728	9.36048434
ILMN_1769741	0.04036404	0.82345668	6.58839458	6.34595658	6.32908179	6.07437549
ILMN_1769764	0.03556279	0.71514382	7.82089056	7.08236303	7.20716273	7.20127804
ILMN_1769937	0.02965659	0.61492565	11.6576583	10.2297694	9.97065669	8.83850621
ILMN_1770161	0.04573148	0.75238697	8.1653452	8.03478906	8.20992411	8.12913926

ILMN_1770244	0.02481804	0.65812975	8.48891061	7.41310745	7.78106328	7.92829748
ILMN_1770831	0.03185773	0.81723647	6.28009166	6.61162861	6.73671175	6.12682752
ILMN_1771048	0.03484549	0.71765577	7.22183456	8.33553149	8.63677939	7.90553021
ILMN_1771320	0.02180083	0.72534908	8.19475242	8.63994624	8.47394192	9.01656123
ILMN_1771664	0.02349416	0.61499023	8.11061955	7.08854234	8.17376707	8.69763067
ILMN_1771815	0.04473703	0.7453723	12.3852835	11.4797484	11.6326627	11.908667
ILMN_1772163	0.03896007	0.61677744	6.38228371	6.07101629	5.97672129	5.74012803
ILMN_1772387	0.04699854	0.70632147	7.74357152	7.54856455	7.97965669	8.47256738
ILMN_1772492	0.02685648	0.64919763	12.4282207	11.5074215	11.5494073	12.0162686
ILMN_1773154	0.0359757	0.69737534	12.8150124	11.8658678	12.5007899	13.1157806
ILMN_1773650	0.02168685	0.60217216	7.28423279	6.85930245	8.43835149	5.46324284
ILMN_1773757	0.0364226	0.77438003	7.03090876	6.82659933	6.75541906	6.94034649
ILMN_1775268	0.03883714	0.65027683	7.19165246	6.72496535	7.21724817	7.63641509
ILMN_1776195	0.03293121	0.70180933	6.07968573	5.81563178	6.01267906	5.46605149
ILMN_1776290	0.0224419	0.69775818	10.808883	9.9900841	9.49340756	10.2958022
ILMN_1776515	0.04724214	0.74797572	11.1039264	9.78430652	9.47572674	10.1424185
ILMN_1776649	0.01605471	0.52227826	10.0642739	8.64870199	9.36396432	9.91495178
ILMN_1777565	0.0390811	0.69544625	10.5873866	9.70087021	8.89706431	8.8275739
ILMN_1778202	0.01340747	0.61458112	9.33968814	8.67161855	8.33088955	8.58085399
ILMN_1778457	0.01335202	0.68407871	13.1638857	12.36177	12.2177663	12.6993085
ILMN_1778709	0.0379524	0.73942115	6.96280233	6.1358209	6.80580448	6.84120453
ILMN_1778930	0.01901809	0.65601231	7.50931739	6.23768589	6.55595416	6.66560737
ILMN_1779228	0.00826694	0.69482096	7.10452223	6.17609215	6.58685345	6.08893746
ILMN_1779572	0.04673331	0.79404381	6.48773088	6.34498504	6.79113293	6.59399788
ILMN_1780496	0.03800162	0.82404777	6.80129707	6.49553576	6.59193174	6.38134845
ILMN_1781198	0.03791742	0.7246858	8.7404979	7.66256157	8.0129245	8.52832908
ILMN_1782459	0.04190805	0.54682459	10.6120539	8.0372647	8.83668443	9.07209133
ILMN_1783060	0.01680589	0.62135054	7.52463339	6.87549135	7.2769693	7.6061209
ILMN_1783085	0.04838214	0.67817979	13.1058473	11.7557985	12.3105972	12.9253276
ILMN_1783675	0.04322112	0.73523955	9.46638669	7.80446277	8.52333691	8.43362091
ILMN_1783795	0.00304682	0.45763251	7.97448113	5.75005279	6.97940919	5.50409184
ILMN_1783805	0.04729595	0.69426592	7.90750181	7.28602171	7.09514169	6.60562545
ILMN_1784300	0.02213331	0.61468043	10.4989758	8.2203822	8.47255926	9.2596816
ILMN_1784380	0.0304786	0.78341161	7.51467855	7.96905434	7.34826784	7.86767597
ILMN_1784554	0.04595285	0.65621355	9.00299443	8.3614802	8.46976634	8.32159083
ILMN_1785167	0.03527524	0.69430791	9.69643398	8.49505873	8.97683502	9.03225929
ILMN_1785345	0.04711228	0.80414041	6.60259762	6.44660743	6.32831966	6.77765773
ILMN_1785615	0.03294807	0.68078784	7.5982781	6.81359138	7.0720672	7.37416138
ILMN_1785703	0.03204618	0.59856199	10.4080898	9.93522208	9.9257102	10.0150261
ILMN_1786847	0.00946975	0.60642473	8.12042971	7.09074548	8.25753871	7.19666409
ILMN_1787186	0.02839866	0.64341348	8.33599366	7.51482686	7.82764185	8.1990765
ILMN_1787314	0.04706392	0.78177086	7.28966942	6.85258943	6.64169572	6.72909319
ILMN_1787378	0.02743585	0.639343	9.98051235	8.92851423	9.20121064	9.55019307
ILMN_1787808	0.03068515	0.67208946	10.347556	8.9766257	9.41396043	9.59312646
ILMN_1787897	0.01317226	0.69570607	8.6052769	7.83879297	7.95739462	8.60244352
ILMN_1788143	0.03425984	0.74749932	7.35704609	6.81147617	7.16091414	6.62367185
ILMN_1788439	0.02716675	0.77971949	6.26979433	6.58296449	6.71625016	6.2800653
ILMN_1788701	0.04740497	0.71525557	9.55544328	7.56950932	7.9156882	7.62307014

ILMN_1790625	0.03910606	0.76299063	8.13315968	8.63534774	8.76957407	8.45689689
ILMN_1790689	0.00517829	0.60528173	10.8749944	10.2657264	10.8474215	11.6453234
ILMN_1790715	0.03834913	0.83643415	6.18703489	6.33286598	6.64582557	5.83193195
ILMN_1790891	0.00703242	0.63119232	9.9306958	9.58562865	10.2352604	10.5538433
ILMN_1790985	0.04901393	0.65119385	8.87508121	7.36046436	8.16545223	8.95772166
ILMN_1791253	0.0078129	0.58920987	7.48090408	6.52625211	7.20893858	7.52332672
ILMN_1791536	0.04423675	0.66030353	9.67023896	7.64657023	7.94199774	7.78235942
ILMN_1792207	0.04825472	0.79038023	6.91256005	6.2867117	6.31577832	6.24607246
ILMN_1792997	0.04642067	0.63940014	10.4700243	8.64886733	8.78465971	9.22820133
ILMN_1793461	0.02940973	0.6210913	6.67039566	6.32057024	6.49576391	6.21460239
ILMN_1794223	0.04412885	0.68083928	10.3500474	8.25953755	8.38936203	8.5540462
ILMN_1794333	0.04462818	0.77314884	7.5614155	7.25167839	7.04294304	6.57236705
ILMN_1795461	0.03893925	0.71402967	7.36692278	6.77181825	6.62844102	6.84006411
ILMN_1796138	0.01968872	0.69396535	6.95375064	6.69619278	7.35704961	8.24944155
ILMN_1796316	0.01063792	0.49972424	10.6776104	10.1267561	11.6561249	12.2401754
ILMN_1796335	0.04311004	0.65858764	6.64412739	8.41862596	6.65146937	7.01050125
ILMN_1796642	0.02288272	0.57283501	11.4640214	10.2998662	10.8969991	11.7085834
ILMN_1797207	0.01881661	0.7407949	6.38462957	6.05179732	6.05261087	5.75152076
ILMN_1797745	0.02634497	0.67312823	8.73046334	7.78079695	8.33997646	8.74720913
ILMN_1797875	0.03585933	0.7155672	13.8352787	13.1469368	13.6912899	13.9161118
ILMN_1798233	0.04845199	0.73773195	8.77269001	7.78192226	7.72217295	7.89829483
ILMN_1798581	0.00593864	0.63486918	12.7663272	11.8254804	12.0279972	12.1063395
ILMN_1798804	0.03901965	0.68114526	9.56862196	8.69552968	9.29140188	10.3701043
ILMN_1799030	0.00715106	0.59068826	12.1977426	10.713536	11.3284034	12.0248336
ILMN_1799106	0.04179542	0.64639203	9.01627038	8.7090128	9.27756429	10.2182823
ILMN_1799644	0.01217579	0.60176589	9.33309742	8.40908369	9.51735405	8.92338394
ILMN_1799848	0.01828948	0.62787298	7.69065874	7.91091611	7.34907241	7.91615893
ILMN_1800638	0.02961955	0.74641136	10.0552296	9.35606676	9.61154256	10.2848831
ILMN_1800695	0.03206478	0.82522497	5.94515094	6.17921962	6.15968206	5.73028834
ILMN_1801216	0.01496856	0.5070089	11.7177201	10.8492338	12.3321854	10.9552845
ILMN_1801776	0.01689787	0.67818632	7.42235328	6.71603335	6.85313486	6.6681475
ILMN_1801866	0.04988812	0.80500975	6.60089993	6.9914901	7.23367815	6.4737848
ILMN_1802096	0.03680355	0.72635799	11.6427065	10.8168677	10.7470533	11.4716646
ILMN_1802348	0.04791188	0.73140149	7.8321267	6.73322146	7.5246326	7.57854931
ILMN_1802758	0.02878836	0.64726546	9.30792459	9.40009236	9.72545846	10.0196435
ILMN_1802799	0.02635078	0.77635704	9.02611534	9.58778558	9.51277009	9.51929284
ILMN_1803500	0.02925383	0.7716853	7.46071062	6.49352069	6.75547248	6.96833733
ILMN_1803953	0.03227583	0.71365198	10.9212035	8.51220674	9.04982378	9.27507365
ILMN_1805104	0.01883709	0.7458432	7.36910262	6.88397998	6.87886142	6.98463492
ILMN_1805668	0.02913686	0.72239003	13.1620668	12.526698	12.6391614	12.711573
ILMN_1806122	0.03078035	0.6451768	9.79233497	8.92043802	9.55358226	8.70282963
ILMN_1806224	0.03287129	0.7346874	6.79191433	6.25607317	6.48020549	6.23935871
ILMN_1806999	0.03654815	0.69589108	7.81718578	7.28126705	7.36936877	7.91262412
ILMN_1807003	0.02689459	0.73765361	6.65544343	6.99920292	7.61088901	6.97707266
ILMN_1807529	0.01062232	0.6211844	11.6769197	10.9159951	12.1675119	12.780803
ILMN_1809585	0.0071602	0.63282531	7.14420344	6.6858841	7.26468467	7.28090065
ILMN_1809750	0.04402628	0.65824237	8.8906224	8.04855063	8.26648204	8.33374126
ILMN_1810229	0.03304353	0.71740175	8.7465684	7.62647637	8.25024109	8.07100052

ILMN_1811221	0.0408638	0.71370982	7.74780898	7.03672091	7.1630448	7.48400678
ILMN_1811330	0.04464779	0.779333	7.36878581	6.36054618	6.7914781	5.99331432
ILMN_1811608	0.0138339	0.75880595	6.78173345	6.40714622	6.56525017	6.2203404
ILMN_1811616	0.03020277	0.80320578	7.56013256	6.80913787	7.33757195	7.0259944
ILMN_1812105	0.01850934	0.67697696	10.724883	10.1537332	10.0793541	10.2817238
ILMN_1812571	0.02109215	0.74257288	9.32507335	8.15668785	8.90908603	8.87022476
ILMN_1812640	0.02405475	0.72366837	12.013381	11.5347503	11.2585802	11.5038392
ILMN_1812856	0.04442435	0.8091471	8.95048795	8.15831986	8.08821529	8.21827891
ILMN_1813139	0.00943211	0.70710146	8.33039795	6.71299877	7.46023635	6.53805232
ILMN_1813455	0.01062909	0.70669942	8.55923826	8.39547844	8.54712512	8.24024229
ILMN_1813746	0.04019327	0.73940812	8.52828883	7.62712455	7.86875281	7.18475834
ILMN_1814039	0.01652675	0.66746337	11.9413389	10.6425779	10.538351	11.3015271
ILMN_1814153	0.04993095	0.81693982	6.66296311	5.91334039	6.41589097	6.04869764
ILMN_1814204	0.01593455	0.7064202	13.7470464	12.8582635	12.9858026	13.3815565
ILMN_1815121	0.02723987	0.77230106	6.84662369	7.14425954	7.25573204	7.16193201
ILMN_1815578	0.03012795	0.70750039	9.69315221	9.35486248	8.612612	9.4601067
ILMN_1815668	0.01820735	0.65800904	8.79033742	8.18578727	9.24392656	9.11104175
ILMN_1816732	0.01095392	0.816986	6.36119952	6.36799198	6.48422532	6.22183185
ILMN_1818346	0.02814533	0.60333514	8.40880568	8.9697897	8.43970873	8.39771099
ILMN_1818621	0.03569277	0.83624447	6.29919043	6.32753378	6.19685251	6.20697819
ILMN_1819313	0.04180983	0.7372267	6.9818595	7.47674458	7.73628083	7.40711988
ILMN_1820160	0.04551196	0.8513076	6.22536807	5.99316349	6.17882875	5.52245724
ILMN_1820302	0.04813147	0.82337135	6.656299	6.14223718	6.347121	6.17211983
ILMN_1821042	0.03250955	0.79028422	6.46118621	6.52846396	7.03047403	6.65558081
ILMN_1821887	0.02615189	0.76249494	6.51823644	6.4441517	6.80531826	6.32357886
ILMN_1822005	0.04771273	0.8630624	6.07049044	5.89729295	5.89173948	5.60598672
ILMN_1823533	0.0126724	0.76283529	6.82660823	6.54705353	6.47643305	6.50417449
ILMN_1823543	0.01780721	0.71262235	7.44600225	7.12884087	7.66601517	7.9714901
ILMN_1824666	0.04795528	0.80947393	6.44676489	6.51550321	6.48872371	6.41671664
ILMN_1824898	0.04413627	0.70001396	7.81576241	7.31734297	7.37200223	7.15960194
ILMN_1825186	0.04559145	0.83672596	6.4377326	5.98381858	6.12422411	5.51881931
ILMN_1825233	0.02115388	0.82931764	6.57258579	6.11042589	6.2952655	5.83515352
ILMN_1831158	0.005815	0.72307832	7.33018997	6.65833289	6.77522046	6.35011955
ILMN_1833928	0.04290278	0.83854386	6.2182792	6.18904925	6.10042939	5.89854141
ILMN_1835631	0.04362519	0.71555091	7.33160491	6.81762837	7.67996068	7.71077958
ILMN_1835722	0.00671501	0.59894885	6.5679392	6.53444063	6.85368009	6.57961629
ILMN_1837686	0.01556524	0.70561596	7.32829435	7.24857848	7.37837723	7.42743018
ILMN_1839609	0.04115986	0.75393819	6.62059565	6.44130287	6.7970039	6.93273562
ILMN_1846462	0.04895442	0.86430369	6.35049283	6.02134225	6.07627796	5.92439685
ILMN_1846910	0.01435395	0.63193734	7.71249261	7.22764193	7.6101796	8.4517102
ILMN_1847530	0.02734796	0.69806709	7.19186167	6.81236858	6.91842321	7.0559147
ILMN_1850677	0.02950122	0.76378325	7.0017302	6.82609718	7.07275645	7.07303506
ILMN_1853167	0.0077749	0.75888274	7.55112838	6.94425173	7.13140119	6.63814394
ILMN_1853438	0.04262768	0.77016442	6.77951451	6.55893861	6.44542396	6.46604595
ILMN_1854183	0.04459308	0.84611768	5.9351061	5.87423187	5.96652922	5.49525289
ILMN_1855286	0.03291709	0.75364754	7.27299176	6.805157	6.88538645	6.44652506
ILMN_1855791	0.04529562	0.84468658	6.14206498	6.0938707	6.00735407	5.45231472
ILMN_1856070	0.04556333	0.67996538	6.67769991	6.93589461	7.62967063	7.96929672

ILMN_1856459	0.04162967	0.75957552	6.40789145	6.33575549	6.48221777	6.21604155
ILMN_1857413	0.023415	0.69525159	6.70763798	6.77187238	6.99831061	6.64656015
ILMN_1860735	0.03043853	0.79453188	7.0835857	6.40110718	6.63930742	6.47794668
ILMN_1863656	0.02918945	0.76871731	6.83615137	6.2847586	6.42585437	6.01333786
ILMN_1864422	0.04664826	0.76974065	7.32047836	6.67007126	6.92235962	6.73687813
ILMN_1864712	0.04759332	0.7325765	7.52018571	6.52684615	7.13442119	7.40807834
ILMN_1866464	0.02648992	0.64713909	9.07474786	8.08855029	8.27621562	8.29565048
ILMN_1867588	0.0123663	0.69085049	7.91181919	7.33864726	7.65180514	7.81850293
ILMN_1869106	0.02632155	0.85046536	6.21607785	5.96237596	5.88248019	5.77246661
ILMN_1870041	0.0280778	0.59274667	7.49593495	7.08599394	7.11387133	7.17317642
ILMN_1870457	0.04074444	0.74951537	6.80860128	6.29659121	6.35293522	6.30054274
ILMN_1870646	0.02856793	0.72529649	7.05890523	6.35790577	7.1728575	7.03210195
ILMN_1872036	0.01080359	0.69131032	8.27402865	8.27232559	8.19019852	8.27492927
ILMN_1873788	0.04724456	0.78441698	6.35499124	6.44087315	6.65413476	6.28735926
ILMN_1879066	0.02559121	0.72575709	7.35564533	6.44604709	7.15346489	6.97378804
ILMN_1880134	0.03567275	0.66995096	7.30157393	6.52205277	7.15983603	7.12951898
ILMN_1880611	0.03174699	0.7577662	6.62260331	6.11093154	6.39903451	6.25139038
ILMN_1883154	0.03642713	0.74258468	6.63108478	6.74012614	7.13697984	7.08269514
ILMN_1883525	0.00901723	0.80190506	6.18836077	6.23282171	6.18663862	5.95910591
ILMN_1883732	0.04630286	0.85137522	5.94782954	5.87401935	5.90342228	5.44523606
ILMN_1884015	0.04195862	0.84550564	6.4163439	5.74202885	5.83448698	5.57000017
ILMN_1884193	0.01252282	0.72370806	7.03356616	6.5912321	7.01093086	6.96997355
ILMN_1887093	0.04826435	0.83489617	6.20326753	5.90748751	5.83800327	5.51963279
ILMN_1888392	0.04402327	0.80450584	6.52814886	6.35007658	6.6910665	6.38202097
ILMN_1888504	0.03605075	0.84509748	6.10288233	6.03310117	6.07654969	5.69023773
ILMN_1890989	0.03843611	0.76837348	6.18715172	5.76669825	5.91602012	5.70380476
ILMN_1896967	0.04161309	0.68343908	7.48232315	6.97316697	6.74518085	7.50718493
ILMN_1898453	0.01101381	0.66172582	7.08590261	6.79381397	6.95813391	6.17963465
ILMN_1899629	0.04126294	0.83874599	6.45057569	6.19542457	6.22708199	5.92762415
ILMN_1900263	0.0238475	0.70147798	7.87877867	7.2895643	7.17871165	6.52575538
ILMN_1902251	0.02132747	0.71450017	7.22980708	6.68973945	7.25381913	7.11069314
ILMN_1902594	0.03996881	0.82689736	6.55692428	6.17292229	6.61308354	6.57282087
ILMN_1903159	0.02990212	0.64268008	9.35815952	7.72009784	8.42641447	8.72684177
ILMN_1907834	0.01470141	0.72355167	6.51152528	6.24727664	6.55107191	6.38009668
ILMN_1910550	0.01003398	0.79205029	6.91368769	6.65906828	6.91895623	6.42634159
ILMN_1911719	0.02403035	0.74499229	6.75927969	6.468669	6.77012867	6.65301414
ILMN_1912083	0.03703453	0.74140649	6.97179873	6.30810873	6.59015781	6.64035413
ILMN_1912662	0.02780781	0.60628383	9.31284167	8.74599291	8.5383461	8.40448636
ILMN_1914473	0.01568292	0.67435412	7.76571905	6.48019871	6.95230095	6.72152168
ILMN_1914800	0.0350841	0.63456296	7.23172712	6.57244239	6.76920973	7.00921839
ILMN_1915031	0.0460836	0.83435836	6.30106922	5.97454124	6.07647936	6.00432363
ILMN_1916292	0.01816967	0.47380096	7.02358139	6.41284773	6.81598395	6.69638477
ILMN_2038776	0.00331489	0.69052448	12.2133597	11.7375068	12.332438	12.741946

C181_NS	C125_NS	C205_NS	C013_NS	P019_NS	C185_NS	C195_NS
13.4585092	13.3893934	13.6358043	14.1197858	13.2577877	13.3431288	12.8532698
7.32976585	6.827175	7.76274811	7.40573629	7.1054057	6.486507	6.91856155
7.59149018	7.53268631	7.16738503	7.20255779	6.64681069	7.11199842	6.63501878
6.92865339	6.12740382	6.10128995	6.76280191	6.51838013	6.84701349	6.25420229
7.25222546	6.8333683	7.15737018	7.32331422	7.1113068	6.2093241	6.05735994
7.50199	6.72804294	8.13002684	7.70986821	7.28354865	6.97145159	6.79127491
6.71307165	7.08999806	7.0250011	7.48991779	6.50522769	6.6537723	6.02043797
5.76570394	5.88514213	6.22308578	5.92362532	5.72270372	6.25511943	6.11082424
6.58765692	7.64270663	8.14783632	8.02772247	7.12595273	7.56175076	6.71731725
6.48637811	7.05096743	6.54648103	6.55791381	6.26504433	6.30956203	6.19393611
7.27060743	7.10391406	6.87295185	6.96559153	6.94484674	6.80234187	6.53158296
8.03825564	8.19837972	8.38461411	8.42699892	7.61594738	7.81340476	7.72750324
6.58098209	5.95512957	7.10799851	7.08615321	6.19645499	6.3688806	6.20222317
7.00672552	7.75136597	7.41351539	7.45537243	6.90992188	6.9251212	6.71871917
7.29910909	6.88333611	6.78515949	6.63902113	6.20236502	6.96961331	6.67539396
6.89765429	6.72156269	7.54998253	7.18895868	6.77645409	7.24193645	6.86774947
7.57510278	7.92411058	7.417369	7.5934225	7.1691313	7.22108825	6.77426127
8.09659605	7.92368997	7.75788266	7.69119634	7.33197118	7.4654856	6.88488482
5.30099091	5.56271683	5.67416288	5.40225557	5.5535694	5.78590752	5.51194584
5.98829136	6.17700725	6.4576176	6.12929023	6.09708992	6.34878445	6.0901012
7.71323115	7.7004529	8.43047346	7.28437717	8.00275371	6.80385859	7.15019002
5.50238566	5.80216012	5.82039548	5.45429238	5.39000967	5.44539631	5.43822265
7.65235025	8.188931	8.66673955	8.15800876	7.87406289	7.67689783	7.16100285
11.7938821	11.1543561	12.1200593	12.0057358	11.5795799	11.1335507	11.3465259
11.1055615	10.7873451	12.7055203	13.0177422	12.1269429	11.0074023	11.4899317
5.78888404	5.7615023	6.02033284	6.48715252	5.7178335	5.70382801	5.64274056
13.7183531	13.5399028	14.2680902	14.5248159	13.8254796	13.8654394	14.0460966
7.71236398	7.86823919	8.1191201	8.70633231	8.29147003	7.93701593	7.30836725
7.22269445	7.87642486	8.03481217	8.20941389	6.96999197	6.3149796	5.99627665
9.09940699	10.4234868	9.98265282	10.1347164	9.16339659	9.04379861	8.76779448
13.4243264	12.5379393	14.2085377	14.2784771	13.7027993	13.647681	13.8450264
12.4142529	12.4973579	12.7798878	13.03613	12.7049394	12.8275124	12.5771954
5.67245391	6.12110557	6.05603201	5.81757082	5.91281103	5.91759406	5.87900189
8.14467201	8.31131873	8.63973936	8.64222033	7.56703048	7.65340044	7.08403449
5.03814554	5.46400623	5.37082618	5.24710494	5.20341535	5.37514337	5.27929745
12.4033679	12.7099251	12.1033586	12.5283872	11.7442393	11.4159715	11.0397717
9.36599232	9.60453488	10.0802899	10.8087503	9.33756995	8.93949547	8.27000886
8.57925747	9.14991551	9.57580261	8.96764647	9.19039782	9.39515558	8.90997047
5.74131974	6.44466011	6.51413078	6.17362577	5.86928044	6.28265682	6.01658947
6.72779523	6.97180908	7.4129373	7.88592145	6.75467242	6.74792844	6.41572521
9.26021883	9.56210387	9.57166904	9.92242385	8.63710556	9.16923956	8.52851858
7.89794889	7.82942085	8.10171047	8.17521869	7.89426847	8.03193659	7.6112137
6.26491113	6.23005926	6.48654979	6.61100325	6.4849888	6.73463782	6.76840402
7.27344502	7.19930623	7.40070186	7.53894317	6.63389339	6.89112751	6.50238503
6.08472783	6.36689918	6.05068344	6.47095702	6.20532663	6.0566971	5.78854478
8.96412914	9.56946546	9.03110512	8.47811556	8.25409276	8.3302088	7.63439143
7.25254244	7.28070026	7.01490312	6.87914088	6.47379422	7.04244298	6.42970483

7.3451586	7.68921388	7.53737917	8.0687455	6.82681794	6.92597094	6.31157069
5.93795473	6.44987778	7.42546014	7.28982064	6.89256589	7.02481388	7.04894563
9.78228143	9.08358729	10.0194003	10.12818	9.36735578	9.91216571	10.0097685
10.7068301	9.73318247	11.0602582	11.0172942	10.4589705	9.94722065	9.8939904
7.74934856	7.68939142	7.42016993	7.10179083	6.72980426	6.70720174	6.37308996
6.79481613	7.21732379	6.61773368	6.89602476	6.34761535	6.48015459	6.04456155
14.1517063	13.0902403	14.5143362	14.6126491	14.0624653	14.2092508	14.0936222
7.39122382	8.09055496	7.96471861	7.66179876	6.61888761	6.91593434	6.70546375
5.99848618	6.23120236	6.25375787	6.15231933	5.98393097	6.04677675	5.80794193
8.9406874	9.48392297	9.66410928	10.086476	8.77043826	9.43879996	8.78251243
5.45532788	5.87463174	5.91734343	5.67738646	5.61736167	5.78669019	5.7351406
7.53760436	8.10527998	7.90973531	8.22241046	7.19461504	7.30708967	6.8592428
9.22337018	9.8087681	9.56833542	9.92107373	8.97684848	8.77405235	8.92686495
6.93186386	8.02124318	7.76008785	7.94287748	7.12971988	7.05844963	6.89879122
7.3848226	7.94481687	7.78158594	8.352371	7.0459016	7.13331075	6.7269824
9.25245847	8.85202288	9.60781649	9.41733082	9.29738472	9.28665224	8.69999574
6.71368757	7.43588608	7.631666	7.02593241	7.05808591	6.94845251	6.56929725
6.46012067	6.05164769	6.26954769	6.1600275	5.81377529	5.81931206	5.60662581
7.36112177	7.77175223	7.68136352	7.60627861	6.85319351	6.49905195	6.49817841
9.66574006	9.84107886	9.87773551	10.1116182	9.35257802	9.43684269	8.8647235
6.51546568	6.45030366	6.91488498	6.77702644	6.46513994	6.18442234	6.06303434
11.5312084	11.7923568	11.9734527	11.9756427	11.37047	11.4651547	11.3576603
8.22259931	8.60429413	9.17862029	9.50151438	8.00298037	7.44538655	7.17458419
7.81811367	7.90718151	8.0201399	7.67003797	8.36039062	8.09243739	8.09207262
8.5580621	9.23775108	9.14972451	8.98436348	8.31112479	8.45201385	8.09942514
8.64968228	9.17734703	9.24782738	8.71083639	8.26837778	8.62074218	8.64947966
7.29663727	7.90726324	7.58792825	7.98166489	6.96950855	6.5146066	6.34199126
8.62526026	8.28037131	8.52788395	8.27528926	8.14655748	7.34732283	7.12303397
8.91600244	9.62051915	9.39488558	9.66969136	9.3217721	9.23021647	8.87328876
6.44960528	6.42222577	6.12208397	6.29709143	5.86362828	7.12964777	6.11541764
8.01419291	8.21575333	8.44284234	8.0429611	7.83001695	7.78278138	7.6859997
7.75389798	9.48960294	9.08429897	9.22567717	8.51867518	7.80185189	7.28024091
9.1960451	9.75387743	9.2554691	9.30701588	8.41563657	8.29426509	7.88053616
6.39051637	6.70304808	6.8195775	6.62410514	6.37505021	6.52185841	6.5812372
8.31525193	7.62461856	8.74140429	8.52550867	8.26280991	8.35160227	7.89534262
5.38805622	5.6087983	6.07153101	5.99244692	5.55610105	5.74275998	5.93784647
6.83377308	7.16175671	7.80813209	6.97429297	6.68332451	6.89476563	6.86884971
7.43896563	7.98760908	7.61937559	7.89060538	7.56375989	7.1226282	6.95345849
13.2432289	13.6212071	13.4338736	13.5736945	12.750001	12.5560553	12.0312415
10.2053436	10.0851989	10.3780726	9.97537654	9.54210634	9.96095892	9.13680968
5.32616519	7.99247331	8.02306125	7.9484636	5.37662518	7.21991872	7.1692987
8.82995196	8.97323758	8.93560619	9.17496412	8.42619136	7.68616573	7.28118172
6.50821772	6.85504661	6.80682283	6.90679682	6.52468604	6.63945388	6.50789277
8.71995312	8.89473017	8.44218837	8.84242201	9.11700901	8.72120072	8.56541858
8.0979768	8.59462468	8.6932674	9.08111243	7.87249882	7.40785917	6.90832416
6.27851618	6.41537537	6.51564733	6.81442609	6.17960142	6.08560672	5.77881308
9.17954763	8.75947179	8.42070429	8.71358688	8.34072916	8.07060132	7.41497269
6.55085604	6.11901082	5.87900311	5.99225316	5.75097597	5.90369028	5.48120561

8.25698091	8.80997001	7.78684594	8.68552743	7.49692566	7.40078126	7.05214306
9.04022012	9.52648692	9.06684479	9.76898817	8.73856013	7.84606012	7.35892455
7.74266042	7.879416	8.19923751	7.74822237	7.3431093	6.75765802	6.6054967
9.21645123	8.79708433	8.69618722	9.04729735	8.46937116	8.8372971	8.1063656
11.9451508	12.482244	11.9093351	11.9840243	11.3366421	11.5563432	11.3966604
5.63079889	6.0832721	6.06665647	6.35052306	6.03908091	6.11849387	5.94330859
8.34103573	8.73375733	9.13160929	9.23346342	8.35165704	8.99706146	7.90466593
6.21425228	6.4741688	6.31494463	6.39513908	5.98546263	6.29283033	6.00431441
8.53828944	8.63492791	8.47029648	7.98842736	7.24540344	7.23733809	6.69481613
11.1623989	10.1009133	11.6950099	12.9042878	10.9254832	10.1554899	9.93609948
8.28210801	8.47684785	7.8566546	8.19456186	7.76093214	7.2301492	6.86373035
10.5158368	11.4787797	11.6919114	11.1115772	10.6739583	10.581805	10.125274
10.8858557	10.976514	11.0779212	11.0664418	10.250469	10.8128159	9.98200388
6.01788317	6.54246732	6.72070256	6.57789057	6.10515403	6.18572326	6.17745761
7.69733111	6.36441818	6.9012036	6.20909382	6.42998964	6.20963311	6.0795106
9.38093763	9.34212713	9.56647213	10.2347983	8.66838758	8.74117404	8.08915383
5.8536267	6.16237159	5.85351392	5.82989217	5.70770423	5.74853879	5.56143612
12.2206214	13.3432996	12.8641171	13.3175836	12.4570579	12.7059721	12.2684224
5.61088222	6.35724514	6.08699538	5.88737076	5.468827	5.67710283	5.57482964
9.67061376	9.89957384	10.1609278	11.5902188	9.89922851	10.0982795	10.7227264
10.6511028	11.5178854	11.5261192	11.7458226	10.163388	9.65964044	8.75908791
11.2262857	10.4946886	11.5362953	11.3296456	10.8321962	10.4489691	10.4973469
6.29178992	6.85339315	7.23355641	7.5401761	6.69458355	6.52504826	6.70367245
7.60127978	7.88525404	8.52178023	8.49374059	7.43705542	7.81749407	6.60293502
6.46478114	6.77956966	6.6712962	6.75804328	6.21463483	6.41480597	6.17490771
10.8132377	10.8372006	10.7122983	11.3575289	10.2262689	10.1905778	9.42397497
10.6895892	11.284613	11.0775228	11.2351173	10.2606232	10.0698149	9.94920428
7.75091329	7.41873389	8.71525231	8.7238186	8.23535705	7.73670769	7.51511634
7.1347446	7.70487542	7.64279542	7.29981062	7.00154669	6.91526752	6.44842609
11.0236088	11.5480474	11.4003645	11.2033364	10.4736368	10.2823413	9.56788137
5.90677542	6.57586322	6.65889981	6.52566816	6.29500462	6.35602211	6.16830908
7.7072977	8.06820646	7.90337094	7.77532122	7.35537841	7.75309304	7.1325532
11.1504867	11.3561086	10.9996499	11.8440537	10.4800752	10.0439842	8.95042555
7.3170951	7.57264566	7.55754258	7.67629209	7.46768054	6.99263589	6.3921649
7.32417605	8.25685593	7.73563455	7.09456018	7.32409952	6.63878313	6.20015375
12.1943059	12.7295041	12.8373256	13.2352176	12.0922986	12.0185	11.4110613
6.53851209	7.74349612	6.75588502	7.69966406	7.22474937	6.57701983	6.82811871
7.98532908	8.09934676	7.88243763	7.49074821	7.4034927	7.27196905	6.76603722
12.0042854	12.1524525	11.8128291	11.9114242	11.7284027	11.5054682	10.8238138
7.05009896	7.46973623	7.3389565	7.32828537	7.20707291	7.15406164	7.06829354
6.38361946	7.91666891	7.19131984	7.91827769	6.9954065	6.25816707	6.1319016
6.71299189	7.39571492	7.27864924	7.50943062	6.31583012	6.44687487	6.19305515
10.5961273	9.99166072	10.540193	10.7992209	10.2816646	10.9709155	10.6863972
13.3862896	13.3353736	13.924729	14.2819974	13.2758534	13.9012176	13.6675174
8.17799451	8.21487957	8.79731881	8.9774986	7.88521377	7.73970299	7.20796604
9.8507324	9.23595246	10.3103742	11.6038836	10.8048595	9.78715687	10.1301085
6.64959678	6.22567809	7.2850415	7.52836346	6.26090166	6.50639058	6.5682252
6.25258975	6.37714267	7.03480446	6.59742912	6.14633223	6.58700307	6.35776294

6.51245731	6.52784964	6.48460783	6.67233765	6.51227746	6.44097545	6.37428434
9.07854659	9.17550938	9.64762301	9.98366915	8.82183201	8.44014619	7.65609302
11.9198585	11.3374764	12.0413334	11.5885207	11.2566699	10.2883055	10.6790279
13.6314602	13.8983898	13.8183565	14.4402456	13.3318827	13.4253562	12.516894
9.10814884	8.95058039	9.16496222	9.04992885	9.00386895	8.65934948	8.48767486
7.2105116	7.75371216	8.38874921	8.05272454	7.39006317	7.45331097	6.6139428
5.77123617	6.15182858	6.28258714	6.47146924	5.69823626	6.25055481	5.88146714
11.8790839	12.6890316	12.5445446	12.9786963	11.0189853	10.7923267	9.72059792
9.77957081	10.5401406	10.6158742	10.6363962	10.0439008	10.4568462	10.1844018
5.9546586	6.09155746	6.61108811	6.74778078	6.63003467	6.50113115	6.48457287
11.1649352	11.7007639	11.8658392	11.7296883	10.3498496	10.0792154	9.19046647
7.06618286	7.70812653	8.00813613	8.17077734	7.28835118	7.71502024	7.39326517
8.83307192	8.98098609	9.4271649	9.93255995	8.94089731	8.74791267	8.01571555
5.48196536	6.26397767	6.2740445	6.05591838	5.72286244	5.98461578	5.75799716
14.0396139	13.1895712	14.6029383	14.6222349	14.1514027	14.4285152	14.4444869
6.63003278	6.23799461	6.99094125	7.64289648	6.98495955	7.2028281	7.01900575
7.05062467	7.86785323	7.73376919	7.71412185	6.75907326	7.40411291	6.56148088
6.79154842	7.36751697	7.32385191	7.33299967	6.52136723	6.48987653	6.28619974
9.70324556	9.48173204	10.1532683	10.0674008	9.12286448	8.41238143	7.75338926
5.65758256	5.79326333	6.04444505	5.81041508	5.44789653	5.95943077	5.74434815
11.1897498	11.4616186	11.4981187	11.5402375	10.5710558	10.3838717	9.76467375
8.38117217	8.51941138	8.98379795	8.5517544	8.52563669	9.0099302	8.57504793
7.39362556	7.85385721	7.83731634	7.793747	7.18881729	7.28260475	6.79236604
6.72389299	7.1196861	7.08459688	7.2119895	6.16255548	6.38854517	6.1292049
6.63706838	7.11047705	7.24441426	7.04945761	6.65735418	6.72993746	6.20813894
7.05363704	7.29921809	7.51273256	7.73669416	7.45829669	7.23814866	6.97647591
13.973013	14.0942988	14.2558171	14.3688488	13.969338	14.0532522	13.9639834
6.42266275	6.0201817	6.26031364	6.2840273	5.59314966	5.88626431	5.64971518
6.73978736	7.0199665	7.31237021	7.31033558	6.5499696	6.94119789	6.64553883
5.44168764	6.37548347	6.38082588	6.4184089	5.6115513	5.91123546	5.77361214
12.8429839	13.2450762	13.4074537	13.7599745	12.6315975	12.7869163	11.9035268
8.84944889	8.13170368	9.20062524	8.88256266	7.2543172	7.84658358	7.73813618
6.97194125	7.28347271	7.64285028	8.01299711	7.05575964	6.47422024	6.4656859
5.32790038	6.17496462	6.71734741	6.38708367	5.80449736	6.52880071	6.34456276
5.67283405	6.16524429	6.07136904	6.00977645	5.85114511	5.98803271	6.0363875
11.075538	11.4377843	11.9235611	11.5004376	10.9532405	10.814359	10.151283
7.51669283	9.04467443	8.34553964	8.60250736	8.06054787	8.02637847	8.10393452
9.42521632	9.17600056	9.68203159	9.78460262	9.46513435	9.19254755	9.33379022
9.72359286	9.54125734	10.3305158	10.753363	9.64469614	8.53847453	8.144155
5.78352868	5.93525205	6.11546237	5.96500941	5.90221712	5.95980122	5.76480234
6.14934433	6.73347637	6.41192293	6.3768198	6.23531387	6.42766986	6.15086467
6.04529618	6.93781635	8.12150347	7.94519976	7.66030544	7.11762129	7.5375438
9.66827694	9.1582739	9.68409216	9.39893732	8.83137819	8.96178855	8.27799358
6.44087266	7.52425805	7.46829413	6.913921	6.69379777	6.47188246	6.3124784
9.11554233	9.46784211	8.59277245	8.72152476	8.72774657	8.40518445	8.02006321
8.7811551	8.62056222	8.91829141	9.01830535	8.05816819	8.02475128	7.86785076
7.29033962	7.74154047	7.72912379	8.14298364	7.28342641	7.4665064	7.29443971
5.47545848	5.7516019	5.9340883	5.81348339	5.71688831	5.85325765	5.77702591

6.97556828	7.2936289	7.17994491	7.50853403	6.6275638	6.78561332	6.45378275
12.3366976	12.6804039	12.492745	12.8291178	11.9110391	11.3908649	10.8366256
10.5830715	10.7826715	10.9746447	11.6181441	10.3739766	9.94725529	9.31303947
13.822768	13.8872444	13.8230418	14.3866661	13.5703408	13.4089215	12.6689964
11.9243694	11.7690994	12.9033099	12.822138	12.1017778	11.7256683	11.1913609
10.1921806	8.53665014	10.0271499	10.6844436	9.27588611	8.82267662	8.78115149
11.9200108	11.1180696	12.5140707	12.2668507	12.150757	11.7361393	11.8819716
6.5485345	7.433628	7.50680582	7.04692099	6.78367142	7.02616155	6.8017006
6.78623564	7.05608925	6.94608632	7.16096959	6.65274174	6.88588181	6.66443591
7.58006406	9.51860084	8.39842178	9.42115743	7.51532374	7.41288143	8.47311962
8.77060278	9.13066734	9.43410422	9.64875088	8.3365378	8.18617913	7.39932322
12.8914807	11.329728	13.1059766	12.4562417	12.2699705	12.0873352	12.0043156
9.65101939	10.0014349	10.0673281	10.4732907	9.22874054	9.19924315	8.41064935
5.7265378	6.30703519	6.53040571	6.43391521	6.05875352	6.38243451	5.8484324
7.16310218	8.09705135	8.16890774	7.3386321	7.11876462	7.86618286	7.34981287
6.45162012	6.84438565	7.31405569	6.86745914	6.26780675	6.16619362	5.96138371
13.1051271	12.575811	13.4533504	13.4435963	13.033957	12.6694908	12.4522788
5.3875297	5.76290474	5.9707713	5.82699584	5.56245462	6.10230119	5.83909845
7.78159184	8.6954834	8.15828956	8.22170553	7.88282338	8.06440111	7.51035355
8.3013837	8.46977082	9.40207642	8.69196889	8.30655937	8.34579509	8.16115255
5.98685619	6.04931318	6.46607842	6.25301466	6.13822408	6.30186599	6.04246684
5.88596061	6.00576803	6.47667217	6.48001673	6.16231821	6.15072538	6.27365482
7.75112224	7.14257273	7.40998741	7.5103488	7.38992039	7.18493087	6.8455026
6.76680185	7.4342454	7.03640772	7.4129136	6.87413915	6.75156147	6.45741257
6.67893811	7.3790664	7.30655755	7.21371957	7.0228294	7.14620207	6.5390662
10.4507834	10.990811	10.8737009	10.8188905	10.7865678	10.6333576	10.0204522
10.1044245	10.1897097	10.7489132	10.8041165	9.45067021	10.0826853	9.18276876
10.1213418	11.3661441	11.0963785	11.3522018	9.46242191	9.42128117	8.52997356
8.55829175	8.96563481	8.68883776	8.51744775	7.98045836	8.01671081	7.61785972
7.97036708	7.71975775	8.33395677	8.35411403	7.49895449	7.49599889	7.23207166
11.9261955	11.8328497	11.5505814	11.4891992	11.0104987	10.9537716	10.2256003
5.85339265	6.1972161	6.47014246	6.29529967	5.72037944	5.94575493	5.68695203
8.96722165	9.33536624	9.99122988	9.8531528	9.79092937	9.59042874	8.80002043
8.49147514	8.47832026	8.61517717	8.3685523	8.24609973	8.39198076	7.74816198
7.18623807	7.25556682	7.07703573	7.13411927	6.62714381	6.34002899	5.96835991
6.33212215	6.27796709	6.47680257	6.7043837	6.20942997	6.38438609	6.10540109
8.7805811	9.68916006	9.4741677	9.31117794	8.6549614	8.85930307	8.78070445
10.8291178	10.4136248	11.3218221	11.1054319	10.0462987	10.1598335	9.93833549
14.3666405	14.0584214	14.0568104	14.6031378	14.1484798	13.8849155	13.7926642
11.112158	11.1710842	10.8801337	10.7752934	10.1886123	10.0221625	9.33649094
6.18776181	6.83867323	6.71699912	6.59516885	6.20530727	6.29660057	5.96628187
9.57983276	9.01597697	9.4913168	9.50875764	9.17830905	9.26776378	8.65699289
7.13735597	6.86172093	7.04897832	6.97178839	6.75843644	6.98481262	6.6227137
13.5682495	13.6428775	13.1709771	13.6072321	13.2662271	13.2694172	12.6943222
9.22509462	8.99425669	8.84121051	8.6440596	8.34296046	8.40412484	7.48674574
9.31713513	9.56950742	9.54732084	9.69370317	9.27014766	9.25630848	8.74292344
5.61055627	6.8312553	6.44515509	6.56520538	6.34625097	6.20716547	6.2342961
9.84342238	10.9700707	10.7009607	10.7825929	9.89203016	10.2261665	9.85070099

6.13925521	6.67047497	6.99176672	7.09227405	6.48809994	6.51071449	6.26842728
14.4204741	13.914672	14.362839	14.7824193	14.4263119	14.4255497	14.210927
10.2239956	10.4364763	10.1632773	12.6706554	9.25837358	9.98808533	10.1388836
11.4189605	11.5980716	11.8673008	12.2787956	11.2669212	11.1047566	10.4869205
7.99202231	8.24378093	8.62067804	8.12550749	8.09211069	8.79069842	7.64828783
8.41120123	8.54961219	8.72858517	9.49044177	7.93036479	7.45317843	6.75819789
7.14830269	7.56177061	7.92236676	8.25309912	7.16910123	7.35282649	6.74135103
11.4108054	10.4276397	11.4223329	11.5618698	10.7752391	10.9674194	11.3346755
5.93479435	5.72862475	6.11679682	6.34008598	5.70771058	6.68475704	5.95500167
6.72054799	6.83542895	6.99925254	6.85113061	6.18831665	6.25076399	6.34567722
5.9145894	6.95183371	6.33949659	5.95614589	6.07774525	5.94305043	5.68313836
6.66513833	7.04585793	6.79033546	6.58282784	6.35632431	6.77681726	6.30605604
12.4457138	12.8402665	12.5860049	12.477479	11.8229926	12.380083	11.8985288
8.78238301	8.75563971	8.88635901	8.96186349	8.03693076	8.32058937	7.92476441
8.01195066	8.2143881	8.80071227	9.02161908	7.7211115	7.64894641	7.40745983
9.84848039	10.8267161	10.572614	10.8850542	9.4765431	8.91171165	8.29237041
10.9586288	11.3350771	11.322287	10.9341368	10.3374736	10.5367222	9.60611572
5.72702979	5.83599353	6.54839686	6.23276896	6.24510524	6.13973777	6.37871744
9.65634137	9.94717915	9.7294039	9.6877088	8.722562	8.8538148	8.45073577
6.18507741	6.62584777	6.6762265	6.83172653	6.08423928	6.28415727	5.96090574
13.8478981	12.8375869	14.3003969	14.586373	13.8401731	13.6631945	13.8403
8.56483237	8.741528	9.23113548	9.83250335	8.65645812	8.43419827	7.91305934
5.82422712	6.14615135	6.85385471	6.15590544	5.94073338	5.86952475	5.63696503
6.97198951	6.81823448	7.32333584	7.25866496	7.30837089	7.22305951	6.93880683
12.4222498	11.2504281	13.3509185	13.5819843	12.7435552	12.3816595	12.3115684
8.98229173	7.44031357	9.33883085	8.65890731	8.39200481	8.63123891	8.18844296
5.57330878	5.65429289	6.08557146	6.06688999	5.85705563	5.99162229	5.86469849
5.53701427	6.49918369	6.98491854	6.89278322	5.4564221	6.30599787	6.26508897
7.97441574	8.33464023	8.02513347	8.51890057	7.5594421	7.15331186	6.81980484
8.6542583	8.97128977	8.98249796	9.07926103	8.50390562	8.45406482	8.24682989
8.65911074	7.33748543	9.57856384	8.70844937	8.00650001	8.30339446	7.95345674
5.6250612	5.94182771	6.29829045	6.01207674	5.74112016	5.70812763	5.76106476
8.38247835	8.72406153	8.85723209	8.55319767	8.99837803	8.32823599	8.95518056
6.96966626	7.46281879	7.71080643	7.0726107	6.75750602	6.90468279	6.39577729
8.12961016	8.15099928	8.13463937	8.41954437	7.64270157	6.86633435	6.68370066
6.89382572	7.17333934	7.40919998	7.55262914	6.71195325	6.58700712	6.48030342
6.97221474	6.56108383	6.57461426	6.34756999	5.91327097	6.06775293	5.92746036
6.4707363	6.47560229	6.43745181	6.34774236	6.16200293	6.03363752	5.72061228
9.57856177	9.43124414	9.88011964	9.75213139	9.1823432	9.14078045	9.10561104
10.2820749	10.7146036	10.2616636	10.8601846	9.96192511	9.30161028	8.76262953
6.3003176	6.65103159	6.96300008	7.06962914	5.88366871	5.95082275	5.65432354
5.75698083	6.55427695	6.33473666	6.25491655	5.94572544	5.93081633	6.03231005
7.07251638	6.86266587	7.1866709	7.17339631	7.14647601	7.41418121	7.18967831
5.94897852	7.05996089	6.9468174	6.87491374	6.32382561	6.57499701	6.25748044
5.56607931	6.04038793	6.01154797	5.68068808	5.53551988	5.85004128	5.57400914
12.5091022	12.1420078	12.8858879	12.8015633	11.7427153	11.5881913	11.1852199
9.01856269	8.19221883	9.65509454	9.88044769	7.94273441	7.13824108	6.92778389
8.81146718	9.34421623	9.27693465	9.32944655	8.79150935	8.39574019	8.20008252

8.73699986	9.0705667	9.13406535	9.55068735	8.85910704	9.53007269	8.58565588
11.1286962	11.4009605	11.8170272	12.1488356	11.2401438	11.2065657	10.7277342
6.38339931	6.78994308	6.59679936	6.66329623	6.15181377	6.05121112	5.75732147
9.02616902	9.23726781	9.22180677	9.23026475	8.38682474	8.35804191	8.16819975
7.57016689	8.24650262	7.96100625	7.81959027	7.06378658	7.83763613	7.32104147
5.63668958	6.00982683	6.33017222	6.0914064	5.77278593	6.40937702	6.09413809
6.31932927	7.14079501	7.06725795	6.75683116	6.29265753	6.71286674	6.61761968
9.50478582	10.221511	10.1834431	9.82044258	9.66855901	9.93797064	9.61936294
8.80949484	9.11532696	8.93588636	8.78252619	8.60416698	8.00787273	7.67820433
6.17893011	6.393868	6.60443373	6.2538178	6.45025895	6.62147726	6.52974194
6.65361612	6.77676858	6.95394847	7.08981379	6.54480374	6.79852235	6.51725459
6.02540426	6.26118345	6.21712658	6.41659134	5.81462092	5.84338871	5.79919664
10.4381951	10.1495706	10.8316398	11.1644928	10.0904142	9.71854932	8.55598789
8.1124442	8.66567061	8.73424389	9.01377209	8.04627932	8.03064948	7.46116591
9.48118086	8.85094566	10.4226777	10.8718452	9.71426407	8.1368476	8.06294953
5.28391252	6.74923524	7.18776577	7.12940873	5.39397728	6.28825833	6.25937946
5.68854071	6.31717652	6.50311145	6.48886057	5.72171962	5.87317536	5.72562284
11.0338002	10.589506	11.1016448	11.2036973	11.0824917	10.2389075	9.94357737
8.74170056	8.62952602	9.20134225	9.23352909	8.37495904	8.85253856	8.00604944
11.6140569	12.0550851	11.968427	12.0066872	11.0967527	11.0772143	11.149803
6.69098831	6.16237481	6.74710001	6.07948777	6.55282492	6.95040776	6.01130151
5.50289639	5.66426323	6.03477828	6.05620828	5.73214626	6.03974487	5.89455587
13.9897608	14.2135113	14.0212689	14.5688557	13.5521179	13.58444	12.7940864
14.0822239	14.0886458	14.0371789	14.0639015	14.1028295	13.6242929	13.3029002
10.1538091	10.1088368	10.5132075	10.8359501	10.6647886	10.5030853	10.268956
5.91913673	6.18226423	6.3312423	6.32040814	5.76831456	6.18318238	6.0432255
7.41135167	7.88240523	8.08695463	8.32412518	7.36689743	7.00212753	6.97266049
6.62808386	7.12772716	6.8349387	7.15842186	6.60168582	6.70542366	6.68512301
7.68059301	8.17112625	8.03861286	8.09396153	7.36928587	7.04605991	6.64124471
6.15393299	6.34428472	6.19762049	6.38629915	5.96846313	5.92378741	5.92425289
12.2547355	12.9249696	12.7502148	12.3786563	11.881824	12.2673596	11.4554274
9.52334181	10.2180364	9.36356206	9.42293139	9.6834454	8.89121137	8.23573328
8.7811774	9.28765652	9.75599598	10.0254275	9.32828021	9.37928587	8.87234337
8.16230925	8.48428921	8.42741594	8.46994782	8.1720164	7.83410693	7.46264378
7.85060942	8.45942793	8.3784531	7.94097481	7.42688699	7.11645833	6.7635749
6.84697714	6.63557869	7.53826034	7.1696695	6.66202458	6.79597729	7.15752783
6.37313434	7.19242105	7.51087808	7.1797867	6.72918974	7.40008189	7.14913639
9.16591897	9.3499862	8.78645097	8.96611895	8.55246469	7.44007682	7.29375671
6.60659324	7.64459106	7.31614936	7.0701188	6.47427233	6.14461522	5.90124657
7.24977715	8.01330704	7.70817689	8.16490291	7.22282581	7.09063434	6.83735797
9.42537622	9.72571321	9.64470515	9.89367164	9.2977724	8.80557042	8.54051671
11.8419476	12.1215549	12.0443593	12.4345824	11.3807132	10.9148851	10.5205607
11.5819698	12.3099518	11.9284854	12.1772766	11.85267	11.4079629	11.1744495
9.19770275	9.48414317	9.67434977	9.46045672	8.03089332	7.696793	7.17376707
5.81842266	6.31518435	6.41758521	5.96510202	6.02007522	5.77904141	5.72923761
7.18279458	7.59073	7.45260944	7.4679674	6.25733127	6.59088042	6.42512525
9.21153865	10.014273	10.5114947	10.624726	9.38112522	8.92420413	10.1434228
7.54940349	8.41708118	8.06541183	7.80275157	7.39658595	7.5438678	7.76296531

7.71919919	8.4055674	8.49274248	8.05073785	7.56563297	7.25393067	6.86510472
5.85366038	5.69471057	6.10072267	5.93274531	5.71690477	6.17411295	5.99774516
7.7442775	7.29363074	7.54323989	7.37695451	7.05118936	7.22645073	7.2310589
8.98590312	9.55837498	9.02424376	9.24514147	8.74042133	8.89692964	8.15958428
7.91585429	8.18831784	7.455148	7.42458035	7.59688074	7.7223253	7.09855641
11.6360844	11.7593431	11.8873981	11.3691556	11.4540273	11.3425731	11.1948449
5.38538099	7.38437365	7.48228523	7.74968309	5.65792263	7.5335361	7.16491698
7.67745468	8.28734439	7.71535116	7.46858413	6.94556674	7.28801612	6.94969484
11.8318503	12.5003104	12.5324468	12.8564497	11.0745155	10.9104134	10.1244975
11.7948861	12.1725541	12.1579131	12.8075246	12.5015036	12.1309936	11.5802397
5.46160615	7.0721444	8.00319519	6.62311565	5.975253	6.95728317	7.46247528
6.33203046	6.26946634	6.79527863	6.38674664	6.04557315	6.18854236	6.20768225
7.1545822	7.79362737	7.40649075	6.91422605	6.35504552	6.71772714	6.19841813
5.17153671	5.82646027	6.76835638	7.21434438	5.34398656	6.1399708	6.07564729
10.2230508	10.4028501	10.7879173	11.506799	10.3928047	9.97558863	9.36150717
9.34392431	10.9921602	10.6663714	10.3775218	10.3950167	10.6493119	9.68060338
9.99936868	9.79947818	9.36630604	9.85927169	8.73928917	7.98200045	7.72936991
9.18154786	9.41078946	9.29347142	9.26694134	8.62902433	8.21960998	8.40612548
8.41738884	8.74157721	9.00377621	9.55760538	8.06976982	7.85944099	7.19740052
12.5013272	12.966082	13.0769669	13.4853803	12.5719936	12.4416023	11.9293727
6.73528117	6.90483329	7.15850972	6.95489306	6.24407798	6.53956612	6.21640772
5.98910698	6.74415168	6.69299064	6.8421454	6.17863239	6.05384076	5.80950345
5.92313373	6.67396057	6.06569164	6.79501492	6.21923533	6.48820938	6.02316374
6.2602743	6.45231308	6.24293056	6.18188791	6.20512914	6.34577549	5.97376624
6.10794852	6.30465779	6.55851164	6.78697006	6.15662334	6.28865442	6.08792231
7.61004668	8.06456494	8.0438705	7.8593099	7.68622247	7.72363228	7.24165812
8.73625647	9.83431492	9.07275404	9.38944248	7.63525377	7.40471157	6.97719176
7.00331664	7.43999789	7.59736655	7.60635931	6.7702886	6.51882623	6.39014677
12.2344091	12.6320373	12.2666255	12.5705025	12.4340689	12.0262042	11.5554078
8.05754667	8.4651268	8.1443554	8.19082669	8.38258858	8.16099933	8.06263767
6.91031773	5.52215433	5.61943003	7.00544153	5.25769596	5.52200206	6.82586829
6.69121589	7.03796419	6.81461835	8.45565148	7.76305232	6.23079998	7.50737208
8.3342259	9.00606403	9.07331388	9.60178787	8.73190142	8.31222452	8.00890925
7.6245089	7.17549167	7.46660755	7.08272494	7.23238159	7.39680683	7.34508318
8.23092013	8.96095154	8.93797608	9.27859256	7.62729457	7.55930672	6.7977121
8.57647792	9.18488377	9.27333895	8.83800188	8.33289408	8.31596829	8.00839949
5.53016597	6.00067656	6.58959063	6.40664211	6.03404356	5.8101189	5.98410716
6.86522847	7.51593002	7.32342231	6.80691432	6.39190757	6.48071444	6.14646481
9.86696375	10.7594422	10.5726851	10.874392	9.20351955	8.84763494	7.91712619
8.35012503	7.97994073	9.05043204	8.52358587	7.46104555	8.08215011	7.32577855
8.25958745	8.99576065	7.96565501	7.58333103	7.76460094	7.41309645	7.41799762
6.47688615	6.53811318	7.1390192	7.36826898	6.55834509	6.21788017	6.10850919
9.20977184	9.63670545	9.43321883	9.74619417	8.58516537	8.32071572	7.81348365
10.0678215	10.037341	9.83116867	9.77217154	8.99602091	8.77333276	8.51272782
8.02980126	8.74035892	8.51460676	8.41098127	7.71477853	8.05512894	7.90671386
6.34272771	6.77486347	6.66673529	6.941543	6.66952147	6.49565775	6.29280291
5.82028365	5.84954847	6.20354515	5.76501194	5.68392772	5.96092334	5.77257534
6.96172055	8.27278419	7.84508102	8.14142666	7.14104852	7.05783256	6.86780382

7.81105931	8.09018495	8.35851539	8.02803313	7.26747587	7.56675428	7.85406112
10.7070718	10.1798021	10.7528949	10.187362	10.2956943	10.0871459	10.0548064
5.79499621	6.14725988	5.90658784	5.92478686	5.5406293	5.93725705	5.69469358
9.67460948	9.77270567	9.71578031	10.0361874	9.70244942	9.10892062	9.08402306
8.04032918	8.27879	8.05589084	8.42434562	7.69530208	6.99084484	6.86046378
6.90538941	7.51622794	7.72029078	6.86492278	6.40583073	6.42195153	6.1245341
7.66357945	7.92175527	8.14063297	8.49335712	7.40865667	7.28898047	7.00768888
5.9621883	6.40235245	6.50332122	6.61407315	6.0278076	6.00263659	5.84835403
8.89404408	9.42443191	9.29962039	9.56789278	8.23802825	8.03425974	7.46238612
5.85799866	6.31342346	6.08030334	5.99799687	5.83176959	6.34702176	6.22006891
8.48874154	7.69098761	9.14361255	9.07209052	7.90614982	7.47258687	7.52926343
6.24707588	7.48656395	6.99269253	6.68486922	6.60415393	6.70657752	6.23618147
6.07864933	7.28188182	6.22138856	7.47719646	6.62772706	6.93275688	6.9424887
6.82891079	6.99328262	7.24945008	6.972317	6.84994298	7.18018168	6.52771579
10.7993213	11.2963203	11.081281	11.0418036	10.8111611	10.5531646	9.62026089
7.87320723	8.20039449	7.10150406	8.08399728	7.64931582	7.00834234	6.57741085
11.3665353	11.7412605	11.6016691	11.694673	11.0932254	10.1976037	9.83778702
5.4164341	6.25632193	6.41710263	6.28792067	5.52469089	6.15305401	5.98600629
8.64331796	8.78955712	8.5303244	8.59396921	7.91005444	7.91399757	7.2276275
13.2114252	13.1404885	13.62546	13.3435785	13.1963499	13.2585727	12.6418057
7.8524184	8.24079038	8.49450482	8.16677519	7.61878925	7.46384966	7.34606157
12.0041594	12.4211278	12.6607311	12.9118685	11.6861595	11.936961	11.1958631
9.08978228	9.32323179	9.40149864	9.76559176	8.87550978	8.83327215	8.00154556
11.4175915	10.7716991	12.138747	11.5622586	11.4679667	11.3122	11.0946682
9.09096587	9.61715226	9.1107195	8.40103778	8.56920017	8.90386558	8.41727574
8.8358644	8.89778708	8.55400437	8.7479714	8.43371145	8.15402517	7.70969179
7.4176728	6.82159798	7.32941874	7.04163944	6.65798435	6.40477696	6.52061334
9.33074527	10.1121827	9.86242876	9.69068719	9.12236885	9.90698737	9.07535025
5.4550352	5.6709992	5.58260357	5.64890543	5.55072986	5.5789689	5.60761853
12.0407414	12.1833547	12.7026959	12.615754	8.40968157	11.6481836	11.932775
6.21990072	6.64950481	6.89273344	6.71724047	6.67347728	6.23106063	6.05106991
5.96574574	6.3507877	6.49520187	6.53188437	6.12159091	6.3471366	5.97308911
11.1246655	11.7982936	12.0498982	12.2124271	11.6388029	11.4114575	10.6076382
6.63979145	7.49066238	7.62188923	7.6516839	6.78107902	6.50790989	6.37344637
9.28169895	9.94065466	9.84061524	9.44382232	8.59465081	9.06891636	8.51430101
8.7490961	9.09463539	8.67345254	9.24556625	8.91126644	9.32737951	9.14261127
6.63419261	6.68307742	6.90202412	6.91990732	6.76594177	6.91916248	6.60733623
9.27652807	8.78803801	10.2219133	9.97642665	9.28337779	8.87551254	8.96582872
6.21964618	7.37533576	7.24413451	7.12451839	6.64300374	7.01728614	6.56990086
12.6386165	13.0228772	13.1565943	13.6527643	12.5576221	12.2526323	11.6581718
8.60352954	8.42829065	8.64162261	8.18815066	8.47183573	8.41914361	8.03379354
6.00427014	6.39592446	6.09037076	6.33937437	5.54814991	5.96127002	5.67456488
7.46510842	7.9881359	8.63265174	8.53959194	7.92048089	7.83956221	7.47921358
6.76559707	6.97222278	6.58723371	6.6904576	6.51082951	6.42097447	5.87283221
12.0197335	12.085668	12.4991261	12.563972	11.6774796	12.0131	11.2420225
6.77383549	6.94083508	7.07791377	6.89584062	6.41008547	6.46986303	6.07714321
8.14182513	8.71028022	8.71350578	9.07759471	7.73351704	7.40316119	6.75542441
7.65698825	8.57656635	8.29889792	8.29231841	7.6289427	7.2074683	6.79798699

7.36070753	7.7272568	7.80719068	7.89782489	7.0011582	6.55236816	6.21362374
6.02534778	6.78262873	6.64587311	6.64510214	6.14328178	6.24682172	6.16800976
5.8477875	6.45390586	6.51396204	6.19223836	5.99752117	6.03744987	5.71092405
6.89411686	7.22219983	7.36699532	6.98524653	6.82934727	7.3405685	6.65856997
10.2756962	10.6492005	10.8333179	11.3178371	10.0515679	9.70509875	9.23067016
8.72304005	8.22464182	8.62241794	8.64781924	8.60759626	9.15666459	8.75124645
11.5293105	11.7164144	12.102833	12.4698893	11.3799745	11.478436	10.9391906
8.11538497	7.86860652	8.55369303	8.47048481	8.00832665	8.26513821	8.28896018
6.3374312	6.39410303	7.14828235	7.36492518	6.84295747	6.70071297	6.44130303
8.78241446	8.64002472	8.03845374	8.23869182	7.86914346	7.70383853	7.43369726
7.52047018	7.84714193	8.17679299	7.9899831	7.46205968	7.62917226	7.32640252
11.2494773	11.190284	11.6185768	12.6087801	11.3579313	10.7724152	10.329793
5.29980036	6.38018692	6.02520048	6.37002614	6.1003642	6.56190438	6.30261334
13.1941452	13.61374	13.6133452	14.0875698	13.0999055	13.0552711	12.4340199
7.11451279	6.93401751	7.07359961	6.85768496	6.64117172	6.68345084	6.40519355
9.38119251	9.54608358	9.86029786	10.5048081	9.27266151	9.00394258	8.30542658
8.35378156	8.84161781	8.51623819	9.22247929	8.44462152	8.45724543	7.89486504
5.94188287	6.20939936	6.10571935	6.08525	5.97965224	6.35724144	6.16357521
9.06189498	8.63631285	8.64668902	8.29435102	7.51045167	7.42363783	6.85336196
5.81778056	5.89946472	5.91078542	6.12870912	5.96096572	6.26447976	5.88447387
7.01107179	7.51765207	7.26343541	7.11791733	6.62354457	7.16757269	6.19376406
5.59805429	6.10765753	5.96203719	5.63015498	5.55592625	5.77293976	5.53553885
5.58025887	5.81777519	5.95781526	6.16272022	5.86697695	6.18590103	5.94468255
6.37719231	6.17923176	6.31007359	6.30028666	6.11537623	6.36141554	6.02599573
6.27796244	6.35049336	6.47257696	6.28753084	5.88515726	6.03342322	5.7924811
5.34296891	5.73568247	5.60524689	5.6648702	5.42908465	5.52407214	5.62166598
6.43290798	6.60810857	6.6921733	6.31075782	6.01614193	5.98853655	6.0459955
7.84397694	7.85608372	7.75209559	7.82620779	7.33813913	7.33688141	6.62957782
6.2237245	6.50221761	6.34296446	6.21376292	6.12455871	6.0265573	5.9191446
6.97073749	7.44706586	6.88808445	6.96024526	6.40867875	6.25666198	5.97120463
5.46625775	5.70710205	5.7745317	5.71926039	5.6001389	5.71974803	5.49783939
5.57063506	5.95097061	6.02298449	5.88852652	5.67596804	6.16011183	5.92715051
6.11737796	6.44911909	6.98090957	6.86435455	6.30164855	6.44105382	6.01174966
5.49431128	5.7441506	5.81038012	5.49519307	5.60540455	5.64366632	5.68626772
7.83952946	8.28526164	7.41443071	7.71580073	7.02635745	6.93074562	6.42746957
6.18776438	6.52230391	6.56552102	6.58145505	5.91473959	5.95300234	5.76696586
7.42281074	7.69502223	7.24330634	7.20371016	6.62012817	6.74167192	6.28881828
6.6387924	6.48607123	6.18357703	6.29541723	6.00893435	6.26049073	5.92753147
5.64669349	5.87341138	5.8756324	5.79953782	5.79409662	6.00604326	5.83832329
7.71989051	8.18180126	7.88609039	8.00761488	7.27344129	7.35509557	6.71794917
6.23378052	7.40087773	7.03315977	6.48608314	6.67731514	6.6196975	6.21010867
7.50433198	7.2694222	7.06017604	7.28486573	6.57162433	6.82409318	6.33324893
6.5914845	6.27215946	6.97620208	6.83406647	6.99962688	7.22565476	6.90722238
6.46647916	6.59862425	6.18087647	6.14988265	6.09632069	6.03761548	5.75556888
5.19846665	5.50293521	5.40198536	5.21490502	5.45982765	5.51881553	5.40276958
6.15079341	6.64487005	6.48915803	6.35421517	6.01759571	6.18935946	6.22476692
5.49138527	5.69590726	5.56222105	5.35836321	5.48490053	5.57420326	5.63302427
6.7700639	6.38881361	6.87061748	6.80086238	6.4002399	6.09228187	6.04753185

5.47454442	5.95672045	6.07625166	5.81380222	5.50880481	5.60331626	5.50804162
6.41600232	6.55804258	6.28742132	6.47124226	5.87675169	6.20917728	6.12767372
5.89576599	6.36874932	6.32389675	6.81968223	6.13344578	6.34507395	6.26526454
5.78215953	6.11899255	6.17391754	6.19999814	5.67473407	5.8062364	5.59740871
6.89445523	7.02092217	6.72365139	7.11020766	6.46508834	6.39578859	5.99577416
7.18268336	7.72223307	7.85478861	6.83089417	6.55924002	7.28043209	6.4880694
8.23433462	8.55445801	8.81329797	8.73767088	7.64362245	7.57575857	7.00957224
7.26136438	7.54000401	7.49174008	7.07874751	6.85491824	6.90323234	6.71634757
5.49242621	5.86753031	5.61727056	5.84699569	5.69151434	5.79289022	5.63884539
7.12545893	7.30445993	6.52827641	6.90494766	6.64253956	6.48876869	6.04672896
6.27289399	6.5655032	6.73807739	6.63977582	5.91254401	5.93225114	5.61158199
7.08961997	7.53234657	7.09809316	6.65141053	6.23719619	6.27914922	5.99608476
8.61341157	8.73136418	8.44103107	8.27149291	7.29658864	7.7843646	7.38889556
6.13510797	6.13369668	6.00100886	5.92007293	5.62302725	6.06179412	5.95950658
7.05431453	7.23669638	6.86260884	6.92146609	6.26285368	6.36702136	6.26219992
6.78367797	7.02968146	6.96530754	6.58868211	6.4867866	6.74479483	6.30558609
6.10442033	5.98429951	6.17617255	5.89369493	5.56608388	5.86433945	5.64894252
6.84930251	6.94123898	6.51613164	6.63010619	6.18578965	6.50334858	5.96415416
5.83821107	6.1983199	5.95617517	6.17391734	5.91733985	5.90810195	5.71168267
5.22376079	5.51963971	5.62094206	5.38055146	5.35409913	5.36923125	5.34647118
5.27331596	5.82148624	5.73224695	5.37981154	5.18610631	5.49458394	5.56931093
6.55525479	6.56259336	6.90269226	6.5441481	6.24363443	6.51215181	6.22888121
5.22159988	5.89581906	5.77835184	5.48986177	5.32182782	5.41365501	5.37768982
6.34718958	6.58126055	6.40131559	6.16426979	5.77061606	6.09682161	5.95549648
5.49761588	5.79136791	5.891478	5.64475384	5.49862618	5.5691824	5.61151648
5.36357095	5.4826831	5.83057326	5.5771599	5.64894482	5.61550311	5.65181145
7.67455669	6.88435249	7.51631226	7.29901015	6.70178884	6.48615492	5.95865061
7.01016777	6.74563265	6.49578996	6.39526147	6.12710538	6.11094114	5.94823919
5.56719647	6.1150449	6.1438548	5.7322977	5.83531676	6.00202962	5.79677632
7.18956268	7.12280522	7.36377032	6.99996731	6.33638016	6.29937765	6.05429564
7.14740338	7.20764399	7.19321486	6.67208019	6.23536558	6.47324921	6.22621854
6.12093021	6.38087747	6.32615387	6.07382623	6.12079649	6.40684122	6.10875151
8.10913277	8.57877421	8.67027738	8.71434169	7.51703155	7.98920885	7.42225992
6.13449614	6.72903471	6.58891922	6.27546546	6.3100145	6.06319875	5.66559885
6.62289595	6.40923019	6.95574056	7.02542863	6.49660344	6.83679393	6.59330895
6.18399597	6.56212838	6.21460394	6.21803076	5.78593131	6.04694238	5.82079702
6.08703443	6.58120375	6.7480828	5.95948085	5.84493871	6.21556329	5.61650474
8.4296122	9.11244967	9.13236349	9.50493357	7.95134184	7.57486369	6.95867219
6.21782086	7.21508059	6.85954224	6.94514766	6.64579675	6.82650527	6.09995601
7.43446394	6.87229812	7.00644278	6.83366937	6.11149565	6.50806033	6.3955816
5.81093095	6.0837714	6.17762245	5.63224873	5.53293811	5.67131376	5.46975348
7.13344331	7.38180335	6.61003989	6.20386968	5.88949618	5.94030819	5.69205917
11.979303	11.3127321	12.2150406	11.9152568	11.7353682	11.6704195	11.7510369

C185dup_NS	C238_NS	P010_NS	C078_NS	C016_NS	C074_NS	P003_NS
13.1992291	12.9973796	12.9743375	13.1036073	14.4249319	14.4124607	13.8196412
6.32061663	6.10646474	6.85616156	6.83332276	8.69045237	8.01216917	7.31250454
6.86697225	6.57902639	6.85465773	6.85944658	7.85763147	7.8006301	7.78604218
6.67602476	5.86096056	8.07055516	5.98051663	7.93372547	8.37230267	8.68416792
6.06976854	6.3741499	7.88080489	6.66123797	9.45385973	8.39334498	9.16515071
6.7151301	6.60187922	7.61086318	6.81915595	8.8163069	8.48948473	8.86308265
6.33959404	5.95632411	6.92653677	6.32314071	8.01700992	7.91897829	8.22189883
5.85000401	5.96592462	5.7966193	5.99027214	6.44605867	6.39368596	6.51342932
7.31510245	6.55044821	7.16377444	6.43519419	8.70260387	8.46872545	8.54842892
6.17660682	6.09893659	6.28507425	6.23114071	6.92471632	6.9677842	6.90492599
6.4265318	6.4426876	7.0507607	6.75819523	8.0084275	7.77071873	8.52316947
7.73053535	7.57404775	8.48366223	7.35535903	9.17994267	9.02710289	9.29694717
6.02048331	5.756908	7.10396545	6.03351551	8.21614344	7.67300796	6.92069704
6.65942369	6.6312443	7.52646951	6.77337661	8.19837334	7.85330956	9.37703978
6.73528117	5.96610187	7.33211086	5.94242137	7.81755355	7.98953926	8.53945779
7.12222025	6.50191996	7.95065446	6.6677176	7.85569263	7.72670045	8.0303874
7.08863456	7.24571052	7.28929594	7.56556291	8.79710023	8.1201881	8.15473155
7.25933087	7.26224522	7.64822598	7.65970483	8.46254154	8.33919574	7.90479657
5.3547152	5.24155307	5.55299272	5.33050498	6.31087267	6.37972821	6.13675124
5.85175704	5.92616622	6.39370964	5.86592797	6.99149577	7.11631485	7.36782883
6.59066579	5.48181557	7.89656673	5.92988471	9.25368749	8.04792276	8.38584687
5.27668537	5.09887976	6.05141735	5.2771784	6.84310827	6.59076129	6.50298181
7.32507964	7.00990138	8.83205136	7.16674858	9.22570802	8.67386732	9.46168018
11.0186224	11.2298252	12.4308521	11.6295288	13.053839	12.9038702	13.2372153
11.1933714	10.8922377	11.2678641	10.4414181	13.5494779	13.6934314	13.0975321
5.29315011	5.61986224	5.90333044	5.40533075	7.05674825	7.00909855	6.56423365
13.8462416	13.4683568	13.6458408	13.1256363	14.7609122	14.8094953	13.9379531
7.65912823	7.02768819	8.03919818	7.49873115	9.21409833	8.18753172	9.50912931
5.9820695	5.71413944	7.16127199	6.05157287	9.35958179	9.26974618	9.17377881
9.38748834	8.23996411	9.30756878	8.61158774	11.2379497	10.9801389	10.9282711
13.8166317	13.4302801	13.7048871	13.3462201	14.7745549	14.8083961	14.2626043
12.6702544	12.6068984	12.8165721	12.4380974	13.4110157	13.7897394	13.5325739
5.52438312	5.52878602	5.98233857	5.57772489	6.52812322	6.52068122	6.72443336
7.33471932	6.99272992	7.63754359	7.21559221	9.36215512	9.30430819	8.91147049
5.1209358	4.96026936	5.08481519	5.03038376	6.17106188	6.26069678	5.91236149
11.4774466	10.9651566	12.7773441	11.4895595	13.4985949	13.1890491	13.1062992
8.95042788	8.61579183	8.73044738	8.82567625	11.3402446	11.4049712	10.3502143
9.15315197	8.83291944	9.5631587	8.56824991	10.0316847	9.84432366	11.071693
5.97059831	5.8592428	6.14988082	6.16002488	7.0967394	6.79382177	6.81381197
6.59862634	6.47852	6.60270495	6.41502121	8.22633608	8.14378165	7.77490432
9.11508164	8.53862119	9.32158744	8.43922501	11.1501249	10.2598868	10.6088591
7.4783506	7.70075874	8.38076494	7.57970739	9.10701694	9.19758973	9.10168211
6.40084239	6.09882693	6.68996583	6.07719044	8.03736355	7.63759213	7.78646176
6.68289349	6.46928788	7.79170339	6.4249733	8.42461184	7.86586439	8.16743772
5.82640866	5.77354251	5.80712691	5.89270576	7.30834449	6.84949643	7.03250092
8.0899234	7.48127795	9.21360221	7.46611155	10.2101782	9.68046854	10.41299
6.68503047	6.40470476	7.30444351	6.70124407	8.13494221	7.96958683	8.06213197

6.54454736	6.38054851	7.13587855	6.38238384	8.55138797	8.82145576	8.17073829
6.80152601	6.67327939	6.68118077	6.65404165	8.80137048	8.23624577	7.65769931
9.66513673	9.16190078	10.0755112	9.83405699	11.1212764	10.9527857	10.8205452
9.70271104	9.4016727	10.9431741	9.49851018	11.6220349	11.4766434	11.2249001
6.37004289	6.35843776	7.26835392	6.7167934	8.17975486	7.77866066	8.25501629
6.21987827	6.03821755	6.49869494	6.20089233	7.5675348	7.25420286	7.12510553
14.0705579	14.0540285	13.880654	13.9019209	15.1898562	14.9851984	14.6736514
6.79361761	6.46549164	7.57603985	6.59041384	9.34637943	8.68718985	9.12895631
5.70216964	5.59483754	5.76214441	5.5643989	6.77700144	6.81630146	5.96369144
9.30902062	8.71301939	9.51538012	8.51453812	10.2331379	10.7876635	9.98142029
5.52998675	5.51569574	6.0812951	5.36576811	6.60596169	6.38933621	6.62545651
7.01379498	6.98584308	7.60781705	6.88100625	9.0154368	8.28781717	8.5666329
8.62069197	8.80292278	10.0532784	8.80094454	10.9258882	10.275858	10.1898814
6.81706551	6.30698435	8.0727586	6.58831197	8.58054802	8.57596878	9.26496962
6.57441173	6.37831289	7.47324466	6.26625471	9.52320575	9.54911408	8.31506712
9.01560879	8.81474203	9.69521785	8.94083361	9.72322338	10.461108	11.1628495
6.51122101	6.2045778	7.35669669	6.59627132	7.9893752	7.55904052	8.2097024
5.50548257	5.73651504	6.70942161	5.94121056	7.38380796	7.15702754	6.74598753
6.15978134	6.35956387	7.32505625	6.18136259	8.44948131	8.67815749	8.45517143
9.39737537	8.82550263	9.97487343	8.61158221	10.3206866	10.4660132	9.96927427
6.0506151	5.74363318	6.45428103	5.93557101	7.8360472	7.83629089	7.68085739
11.4839478	11.1309926	11.8639152	11.146267	12.7774201	12.5816255	12.2956619
7.32650769	7.05776759	8.26293296	7.12536491	10.0496811	10.085013	9.87778136
7.8027477	7.9971456	8.892071	8.06873261	9.18412678	9.04092092	9.01090345
8.18803194	7.85449878	8.85383538	7.7996242	10.2451236	10.0605838	9.91685399
8.51371299	8.48496424	8.85692813	8.44105183	9.33743615	9.6621227	8.79975467
6.37533333	6.02205483	7.20361327	6.25654725	8.91576527	8.23770759	8.41946895
7.17483283	7.20503371	8.56620439	7.33933339	9.33890794	9.72245168	9.86264604
9.2597395	8.86974898	9.29116471	8.75240072	10.2562582	10.5651249	10.379726
7.08452141	5.54214319	6.47719711	5.87380902	7.84383314	7.14954899	7.27100914
7.61037599	7.41598086	8.36636681	7.71480943	9.64783758	9.38911031	10.1630987
7.57540159	7.2076518	8.79770432	7.41160166	9.88496478	9.94320417	10.0521836
8.45106148	7.67133725	9.32601954	7.63691241	10.4019493	9.63699522	10.4554526
6.28461907	6.29129523	6.81276518	6.23903098	7.1006697	7.18562971	6.78957407
8.20252547	8.05386996	8.76366055	7.99209031	10.045271	10.4201866	10.6580776
5.45224717	5.51358991	5.76336164	5.57581967	6.50912796	6.48251273	6.31040684
6.6171117	6.55186542	7.28133475	6.609626	7.61507195	7.5391177	8.44732462
6.904077	6.79669379	7.2763608	7.18120196	8.57659016	8.04644741	8.43371354
12.5523771	11.8933205	13.4507331	12.1345858	14.4382506	14.6302432	14.1795515
9.68669836	9.29616829	10.1190903	9.65128315	11.0552309	10.994004	11.6559175
6.95383908	6.8181859	5.31262851	6.72688434	8.26264174	8.14970089	6.0348784
7.53300105	7.20885764	8.79946976	7.34095298	10.1233092	10.179526	10.1394759
6.50639137	6.32069533	6.94068476	6.33785753	7.94831992	7.5342702	7.32568862
8.58194472	8.27948198	9.55448237	8.26083864	9.72784185	9.49114119	10.8283166
7.12039549	6.6671229	7.76886647	6.51437353	9.89473835	10.2270202	8.99501692
5.68774995	5.62669173	6.63006745	5.89050652	7.76855223	7.98551238	8.04760382
7.95661661	6.90154997	9.09387414	7.06015009	9.48425013	8.45380769	8.55537061
5.57915812	5.2115095	6.23901398	5.23648497	7.26588036	6.83243309	6.59811654

7.17849356	6.43004252	7.38879656	6.98217448	8.59787676	8.64283621	7.92722566
7.71596336	7.17859116	8.76369473	7.18223145	10.1867989	10.1795919	10.2358383
6.55043744	6.63685819	8.6254042	7.38964252	9.9537083	9.14066722	9.74783767
8.68957838	8.77841197	9.11789266	8.38064591	10.4343624	10.1430439	10.4964134
11.6423613	11.4864192	12.7284046	11.5779589	13.3739554	13.1027214	13.0217762
5.65997421	5.69457686	5.86690551	5.55466927	6.84894086	7.08869815	6.2851673
8.64011295	8.28798197	8.6685794	7.38498315	9.54470697	9.57875327	8.87929039
5.75170178	5.92827769	5.91792745	5.88063974	7.17142002	6.88094138	6.69895881
7.10250487	7.31775177	9.26978171	7.55034047	9.18753667	9.44608333	11.087901
10.2301273	9.43904337	10.8377068	8.47204377	13.5686972	13.0873301	12.2328679
6.79260551	6.45269163	7.69330992	6.58700217	9.00224007	8.95931895	8.56650771
10.485458	10.246085	11.0567908	10.6473955	12.0816369	12.3225206	11.4627139
10.7448725	10.3552322	11.4135703	10.9990825	12.3363757	11.9190689	12.8533563
6.03270809	6.26236907	6.51122829	5.80904523	7.31074273	7.62310599	7.14834133
5.82511856	5.98454834	7.57204457	6.44443202	7.18432295	7.55192431	7.38800781
8.3914219	7.88868907	9.03621786	8.14024746	11.1321212	10.4256251	9.79148152
5.43773709	5.55572844	5.57322242	5.44617946	6.45747265	6.620023	6.14247634
12.5620301	12.0024016	13.4305636	12.4199962	14.2032004	13.891741	13.9292085
5.38758484	5.42554759	5.5787718	5.6944033	6.12161536	6.72665686	6.17991625
9.95523593	10.9636815	10.0565388	9.39226246	11.9804845	12.3732964	12.1004028
9.48708413	9.03897275	10.2658928	9.35207301	12.5788335	12.5133104	11.9574235
10.2073841	10.414348	11.9943064	10.9088054	12.4096879	12.2546416	12.707894
6.28018095	6.23714628	6.75285829	6.36894431	7.56024875	7.48061735	8.47539744
7.60415808	6.96636125	7.88027533	6.29414467	9.26825004	9.17229799	9.55639359
6.06648749	6.02899474	6.61439153	5.9975914	7.55531385	7.77329287	6.87249267
10.1094272	9.70650154	11.1344661	9.65218602	12.0832117	11.827145	11.8430055
10.1416614	9.7794472	10.7750785	9.75538234	12.3369865	12.3606307	11.894684
7.28501837	7.22132901	7.91376486	7.39355263	9.30626315	9.04397819	9.00441684
6.62849847	7.20613525	9.23636681	6.87586114	8.26075727	8.48110919	10.6604818
10.3209709	9.70922963	10.9124193	9.45177427	12.4076491	12.2272886	11.2250851
6.10026663	5.81233136	6.39917691	5.88821136	7.37610368	7.39957188	7.19164358
7.46382596	7.16713811	7.97269323	7.25688894	7.70328623	8.08555478	8.43145373
9.90777668	9.26806028	11.7215935	9.73315484	12.88273	13.0275579	13.0347177
6.82634637	6.25041667	7.57803826	6.25773273	8.80410101	9.19220872	8.77357541
6.57152559	6.24192272	7.92654685	6.70194544	8.54836457	8.18507116	8.76418192
12.0082045	11.5305327	11.9221726	11.755206	13.8409284	13.9423789	13.2912067
6.30828114	6.35128715	7.2340874	6.63587669	9.03188009	8.80692978	10.3426636
6.97376509	6.68747087	8.00893782	6.99104673	8.56557281	8.20426008	8.10489381
11.2921122	11.0722298	11.7698205	11.1941948	12.7506053	12.5300249	12.824427
6.93301547	6.74544711	7.44398866	6.65107035	8.34758815	8.44125107	8.83593449
6.1439003	5.81338616	6.8546864	5.77911812	8.6747304	8.9306859	9.0611916
6.09714935	5.95795591	6.57107531	5.76035722	8.21487569	8.54068176	8.2112079
10.8172467	10.6226238	10.644572	10.0230405	11.4639749	13.1324719	10.8160549
14.0218068	13.5806249	13.8803498	12.9814861	15.0631869	14.9372809	14.2745987
7.56214013	7.10797446	7.70771942	7.55262146	9.65227908	9.81409358	9.0906616
9.54091438	9.48151995	10.1327799	9.11281818	11.2308371	12.2949268	11.1369578
6.17276692	6.05910323	6.69214819	5.95119868	8.06112814	8.63665507	7.06604505
6.39715983	6.22525661	6.65696966	6.07169125	7.55993685	7.70759933	7.29282039

6.01908072	6.24762338	6.25987054	5.98259458	7.10813237	7.17672468	7.37497686
8.5199452	8.11200937	8.55471048	8.35433623	10.5652811	10.5802708	10.2245938
10.3991946	10.78005	11.1644978	10.1390294	12.2581871	13.0172601	11.9697304
13.2853503	13.0591496	13.5984666	13.1196454	14.9269459	14.9415681	14.1435148
8.41057346	8.63305988	9.26252994	8.44223607	9.37912988	10.0356363	9.78392297
7.27853867	6.73473807	7.65064682	6.45349338	8.96878687	8.74980776	9.20650062
5.86164526	5.78699017	6.15629326	5.53045784	7.39648748	6.77806136	6.56838954
10.6574775	10.2125146	11.2639053	10.5179134	13.6820202	13.7782414	12.6836561
10.182521	10.0197811	10.3265227	9.63391213	11.0572682	11.2503778	11.4079969
6.30005928	6.32596487	6.39905485	6.48460219	7.40194698	7.28040054	7.00005861
9.90657617	9.76334219	11.003995	10.0835046	12.8324359	12.779357	12.2136167
7.52254858	7.78053189	8.31638834	7.65574179	9.08705623	8.91144952	8.98525962
8.51991769	8.19791551	8.46424554	8.54278968	10.2852901	10.2998456	9.99861158
5.830144	5.69573108	5.96048854	5.53642161	7.13389843	6.85231102	6.64748721
14.3666419	14.171011	14.1802697	13.9800717	15.1523974	14.9591675	14.6466189
6.95219143	6.27564982	6.80590506	6.30383919	8.00288195	8.24325254	7.86862874
7.21455906	6.41150794	7.15745305	6.40632305	8.14722325	7.97900097	7.09080581
6.22304136	6.02138445	6.80069286	5.93946003	8.339022	8.02570158	7.92437429
8.15354089	7.76529671	9.34602721	7.91036451	10.6862528	10.3409038	9.01357057
5.7436566	5.51303336	5.85073518	5.613257	6.51396299	6.32297908	6.22100451
10.3976672	10.2623004	11.6715818	10.3164795	13.0089384	12.4062893	12.9766241
8.72963808	8.48850135	9.35385961	8.65628971	9.68919781	10.1630169	10.0388299
6.86191816	6.72889463	7.51127906	6.90568665	8.52747935	8.41939942	8.25357097
6.02588879	5.98751211	6.22132417	6.12291743	7.80081968	7.71366489	7.46770988
6.58850792	6.01210916	6.05583012	5.99783546	7.48157827	7.26109354	6.59161794
6.70924927	7.17461814	7.01039831	6.84717514	7.44143451	7.70158579	7.6664328
14.1606577	13.8644696	14.3348431	14.0025909	15.1585209	15.1707627	14.7642138
5.73602465	5.40280948	5.84541453	5.23824231	6.19887167	8.54301364	6.44849514
6.35526422	6.347121	6.43047296	6.369832	7.65142488	7.91385041	7.21162747
5.52631425	5.45453027	5.58547156	5.45794127	7.08967293	6.91714768	6.25371685
12.5858045	12.2304315	12.3698634	12.4284939	14.0375084	14.2411989	13.444998
7.62752513	7.84938633	8.34345224	8.12816553	9.34838957	9.06553435	8.99151079
6.22112423	6.33081902	8.39866461	6.08203724	9.70739537	9.56164939	10.1575882
6.12799989	5.9473024	5.54252551	5.76350001	7.19307092	7.14108327	6.1609593
5.82786093	5.6972762	5.79985431	5.77115164	6.86901387	6.91501888	6.53960954
10.6665667	10.1247495	11.5946176	10.7438053	12.6300894	12.7807022	13.0107604
7.84016422	7.22766407	8.23883939	7.54879494	9.00584627	9.04159263	8.44038127
9.11578133	8.99104956	9.46778771	8.87983134	10.4430544	10.4354348	10.2709961
8.47309608	8.38901778	9.87057803	8.57728871	12.4696439	12.452903	11.9225052
5.60308774	5.60710568	5.81190485	5.51438584	6.64238562	6.69785442	7.2006093
6.00338883	6.44471427	6.40710679	5.85488409	7.38908319	7.59969791	7.23632327
6.98192684	6.81654467	7.53832941	6.41797265	9.73139303	8.88797148	8.24103407
8.6675285	8.1635249	9.68557894	8.31837334	10.4943316	12.1087032	10.4996018
6.28059894	5.70538771	6.95365869	6.10576879	7.92052019	7.78893949	8.00044796
8.06607143	8.06811561	9.15998307	8.06722729	10.2856877	10.1818691	10.1766631
7.86666199	7.73861856	8.57546966	7.71626113	9.52938643	9.8536988	9.14008076
7.14285757	6.81449275	6.88164871	6.78957668	8.9650183	8.79661657	8.17074831
5.54062899	5.39533294	5.70915965	5.47340898	6.47945888	6.4398937	6.34285836

6.54058281	6.30975991	6.48365893	6.23136655	8.11101827	7.73996283	7.36099892
11.4893046	10.8892127	12.633671	11.1799551	13.6419129	13.1490744	13.6092353
9.8682486	9.50219692	10.1417189	9.83098193	12.180045	12.1654629	11.7148624
13.4209405	12.9423437	14.0187766	13.1818536	14.7593629	14.6428217	14.3800836
11.6874168	11.088127	12.4290348	11.1719893	13.2063336	13.2636533	13.3750096
8.96304518	8.96689704	10.3465835	8.37386524	12.3559575	11.6436984	11.3537842
11.5660331	11.6845865	12.4105059	11.9391751	13.2662119	13.2451071	13.1516182
6.81887867	6.29054531	7.64205964	6.30974209	7.81855534	8.18996635	8.38063898
6.67445218	6.36328359	6.74416783	6.5390558	7.65589274	7.58735599	7.47176624
7.25704363	8.71233165	8.32931379	7.13138885	9.48492679	8.68852553	9.68569789
8.08128689	7.56865919	8.53442857	7.99074502	10.3827904	10.444605	9.77802356
11.9175977	12.1064301	13.3792294	12.2069481	13.5582042	13.9750096	13.8657014
8.90675534	8.43998086	9.15768369	8.73488605	10.9717591	10.7691023	10.5049163
6.12532688	5.76153687	5.96555067	5.9562005	6.85065068	6.86553781	7.06167253
7.77377814	7.66383061	7.80129449	7.1476191	8.94179356	8.58042893	8.21483102
5.7739726	5.80467366	5.93331462	5.59259577	8.06885028	7.16595213	7.78607747
12.4678814	12.1471477	13.6434865	12.6325266	14.1637802	14.2673507	14.0755368
5.79553813	5.73065789	5.73584653	5.69698449	6.38970963	6.61185697	6.15227835
7.78796262	7.25348163	8.12823565	7.16507984	8.93107994	8.83035421	8.93578149
8.04607246	7.97445647	9.25599256	8.04535232	9.87172924	9.69500691	10.1625562
5.95009906	5.70220483	5.77969388	6.02590695	7.10236629	6.89023144	6.51727223
5.6594648	5.92104471	5.89930689	6.12134329	6.54971091	6.72012248	7.48550089
6.99192994	6.88187993	8.64495763	6.93166651	8.48451761	8.14505174	8.75355373
6.4117313	6.18626244	7.00827734	6.4936515	8.34476815	7.90812177	8.54365556
6.47436709	6.53872625	7.09069044	6.13971669	7.88463089	7.86407345	7.81727789
10.6449243	9.85279196	11.0366778	9.94800155	11.647699	11.1491704	11.677314
10.0635286	9.57837949	10.0636971	9.80808188	11.6677334	11.6786864	10.5432668
9.18773138	8.88243611	9.95526717	9.06056462	12.3249189	12.5905709	11.3929479
7.83626501	7.68323045	8.34813724	7.56409613	9.09621704	9.06958591	9.441051
7.15924267	6.96385324	7.54624626	7.05215284	9.36044812	9.1118863	7.64868689
10.8087881	10.2017221	11.6696287	10.2846587	12.647359	12.4824046	12.4586758
5.54895319	5.58469498	5.69179795	5.31997336	6.93468044	6.85692844	6.34400734
9.39261366	8.93642064	9.90120405	8.88436226	10.21463	9.88647501	10.3976832
8.04429958	7.85044191	8.04051714	7.86577595	9.5954328	9.48103632	10.1970225
6.01488839	6.14840743	7.20842557	6.50135507	8.32163682	7.77864687	7.97136306
6.16482066	5.98893117	6.47209081	6.05174078	7.51561548	7.12360102	7.35396104
8.70136434	8.18030901	8.94975202	8.6779687	10.650191	10.0595878	10.484687
9.95294417	9.6088619	11.0656989	9.81431473	12.2554529	12.2450803	11.9490687
13.9330645	13.5033438	14.3047021	13.5020809	15.2362448	15.1710564	14.7915994
9.95531295	9.59438227	11.1755758	10.0096803	11.6494439	11.8215635	10.9883112
5.92645185	5.8456599	6.20913789	5.85146822	7.35419648	7.34847677	6.86786188
9.04165176	9.05398098	9.2778388	9.39588732	10.1813473	9.74879966	9.64156391
6.71158438	6.5258678	6.92964189	6.50058108	7.73813145	7.62448989	7.29622612
13.3072179	13.295789	13.7580597	12.9858636	14.4285741	14.4414671	14.0911643
8.10526426	7.83391978	8.82815839	8.75769635	9.64408069	9.32671957	9.02674557
9.22378574	9.32286825	10.00532	9.69746647	11.1220625	11.644758	10.4904227
5.9073493	5.96214804	6.12401789	5.76860278	7.66471251	7.37710032	7.17064317
10.1796578	9.72731824	10.3468328	9.70092915	11.412926	11.6266144	11.7025407

6.28199529	6.33544592	6.35733365	5.95586096	7.66393874	7.2851266	7.82027727
14.3942798	13.8780797	14.4724967	14.0260064	15.3166994	15.3947347	14.81053
9.95089732	10.3298782	11.4200218	10.4448637	11.9297045	11.9242001	11.8425951
11.0035074	10.6218797	10.9879333	10.843037	12.7437706	12.8630778	12.2124602
8.56063472	7.94211918	8.5799585	7.53235047	9.7672423	9.3136277	10.333616
7.33492576	6.64590336	8.14123965	6.59271416	10.8260948	11.1807521	10.751185
6.91292286	6.50426444	6.63239692	6.71873835	8.53366301	8.54917435	8.27365948
10.9009611	10.7506501	12.1166585	10.4633854	12.4314605	12.0509295	11.4924706
6.28342826	5.27204522	6.06954064	5.44530526	7.07675682	6.78104491	8.50204532
6.11707537	5.93521881	6.32331512	6.13001943	7.59229033	7.38471383	7.30612003
5.85877327	5.23548697	5.57090885	5.70000351	6.40553039	6.85407172	6.2073738
6.25068119	6.10592673	6.70242206	6.29986867	7.69293556	7.84840386	7.0501904
12.1837427	11.9709475	12.7800156	12.1560568	13.4852696	13.6873134	12.600532
8.24384708	7.68248692	8.45039473	7.12965807	10.1062672	10.5763313	8.71850822
7.42470883	7.61306011	8.42923892	6.91686832	9.26924593	9.60412823	9.41339014
8.71036839	8.16785766	9.87773076	8.45508429	11.6415384	11.6041357	11.509199
10.2170465	9.75941536	11.9452251	9.99716113	12.1930426	11.3680863	12.101328
5.98382519	5.97077476	6.9412155	6.12785714	7.7989111	7.06163149	7.32607978
8.76502812	8.41612151	9.8350687	8.40951823	11.0204105	10.606878	10.3031688
6.08128785	5.87262391	5.96877069	5.76555252	7.31898109	7.48363603	7.06156669
13.7481425	13.3728188	13.9316507	13.2513525	15.0531041	14.8468023	14.5140581
8.15377899	7.87893374	7.91071707	7.98828477	9.99476073	10.1084356	9.45815209
5.46493091	5.31452035	5.88396337	5.53589992	6.75718197	7.54655959	6.65047737
6.72946032	6.91246425	7.2421747	6.9308816	7.67324122	7.71222364	7.9875295
12.3042061	11.9032323	12.3038144	11.6907942	14.1552752	14.1163123	13.7293457
8.42477096	8.13910313	8.59530411	8.66918023	9.85840846	9.52945066	8.5843714
5.66491086	5.58921129	5.95915994	5.58544272	6.8904698	7.0937746	6.71077062
6.07782428	5.87100799	5.43037059	5.97875804	7.72235536	7.46706831	6.21639724
6.94467341	6.79184793	8.12724222	6.82000404	9.81441536	9.73134738	9.61587604
8.1737296	8.08739707	8.84415463	7.91524182	9.67061695	9.94391737	9.0589477
8.15293074	7.74485402	8.72659522	7.92664173	9.85009118	9.46558865	8.31941923
5.59238976	5.50390268	5.90504469	5.93697637	6.96711557	7.02620471	6.71288462
8.22117093	8.99541442	9.13800218	8.79769686	9.69126737	10.3326196	10.1586879
6.79968183	6.64694746	6.9559207	7.12824184	7.95434406	7.86022413	9.01611407
6.75725399	6.44880698	7.96086034	6.65410324	9.27229409	8.98272868	8.78011387
6.36381569	6.07475802	6.61891476	6.32266242	8.28677998	8.24377998	7.55643018
5.7868752	5.44522678	5.99229282	5.83476986	6.631368	7.05754991	6.56265228
5.96088072	5.51695278	6.6119319	5.53524209	7.44632323	7.76210361	6.98754485
9.29293678	8.98283647	9.79785847	8.85458948	10.7218673	10.8809839	10.2381802
9.01289434	8.69064093	10.8255003	8.47663726	11.7885899	11.8517366	11.4295574
5.69372139	5.58853656	5.80265077	5.5620357	7.62119181	7.77527644	7.01797645
5.70838094	5.61448518	5.82014883	5.6691983	7.03810361	7.00565935	6.37819479
6.84305675	6.79918576	7.69602303	6.86946923	7.81038398	8.02899783	8.36225727
6.15413173	5.83579425	6.14365912	5.95956431	7.88557681	7.3774639	9.13182944
5.53978136	5.39433064	5.80962718	5.43839865	6.51510403	6.5248247	6.49055746
11.3448995	10.9910236	12.53547	11.3463819	13.9851597	13.8708251	13.3150703
7.02475405	6.89597267	9.74738774	7.26276012	11.575032	11.6172392	12.0770484
8.30950364	8.35365058	9.11383617	8.41094609	10.23786	9.92103756	10.1131001

9.22328787	8.60225532	9.2173696	8.73080398	9.93781958	10.2649527	10.0215963
11.220408	10.8783606	10.8878816	10.9703928	12.6077194	12.6855202	12.0932565
5.80962538	5.62659537	6.25243326	5.74595044	7.81927093	7.89159295	7.41558799
8.18522432	7.9292442	9.24947307	8.06358329	9.81553476	9.83277611	9.78815011
7.54664293	6.90349507	8.11439097	7.0032188	8.38669931	8.29654643	8.88084437
5.78577442	5.7843061	5.92860412	5.90585822	6.58219513	6.64349879	6.53916052
6.27843979	6.71973243	6.48732781	6.71474419	7.89427514	7.88166829	6.92132921
9.85145032	9.70538322	9.90151588	10.075546	11.1937021	11.1984217	11.2304186
7.71611294	7.60047717	8.7347252	7.88168175	10.1845791	9.87798499	9.81659587
6.27537718	6.29037058	6.5486151	6.06590723	7.21283097	7.62554302	7.14121103
6.38703833	6.37058158	6.87399771	6.70711061	7.81268625	8.04875441	7.8186512
5.45401924	5.56304034	5.83574752	5.46540478	7.23551167	7.30890053	6.37666216
9.56858244	8.79586855	10.6507242	8.58934142	11.7924857	11.8895032	11.5960933
7.65841073	7.53896877	7.81345479	7.5081101	9.70843782	9.75505798	9.11971411
8.09947669	7.84116616	8.98174079	7.71724664	11.4721247	11.5720654	11.0608168
6.04650961	5.94257034	5.35748409	5.94559881	7.12923974	7.05699205	6.01828209
5.59552723	5.16710418	5.60001427	5.21083107	8.18355748	8.0587321	7.34103395
10.1490358	10.3419157	11.1542359	10.2049183	11.5687276	13.0865683	12.5877864
8.56394022	8.07737226	9.38587275	8.15279236	10.151647	9.65813149	10.0890545
11.0067111	11.0483309	12.0419096	10.8679891	12.9053103	12.7338848	12.3920558
6.58628482	6.44473216	6.5409389	6.11940826	7.03383263	6.93672155	6.73766378
5.646014	5.68491129	5.5037755	5.51827767	6.29492782	6.48351496	7.64341249
13.6747583	13.1653593	13.9511964	13.3346043	15.3461619	15.1588625	14.3997532
13.4397387	13.1894325	14.3417634	13.5831568	14.9358115	14.7978363	14.6925627
10.3389893	9.50482852	10.2050149	9.89753072	10.3832226	9.64019016	8.8975941
5.70581981	5.60890828	6.25255739	5.86590051	7.44598156	6.94679986	6.53592526
6.80840307	6.54050237	7.41720611	6.42052165	8.30069437	7.93863278	7.76263451
6.51334451	6.07098217	6.64833889	6.52413484	7.92339464	7.67548499	7.38393666
6.77614479	6.59303789	7.83682358	6.70216576	8.78439307	8.50751678	8.84660144
5.69215684	5.54226579	5.92607653	5.55967413	7.57456098	7.33213951	6.66100846
12.0325803	11.6566389	12.0995346	11.8926878	13.2266566	13.3056656	13.0791291
8.83094803	8.55997623	11.1163466	8.80713367	11.2581897	10.5154673	11.7145881
9.22277655	9.2322535	10.256572	9.22465918	11.4171806	10.5936563	11.7120304
7.66141898	7.75111621	8.60639781	7.64821879	8.90728097	9.39773538	9.16242658
7.05474756	6.64601429	8.00401536	6.48859727	9.99772145	9.90698136	9.69035371
6.6217593	6.47798746	6.89653222	6.58944756	7.85791694	7.79211547	7.99647914
7.19847254	7.37218599	7.37864254	7.00322442	8.89105032	8.58822666	8.9243434
7.28410043	7.30000289	8.92653974	7.48694521	10.3614734	9.84846129	9.45176624
5.94792466	6.04114177	6.2658423	5.88828151	7.67281708	7.30770993	7.00890701
6.92810853	6.48126342	7.25704174	6.74418667	8.60226274	8.87803068	9.04672323
8.74099264	8.42712508	9.39181003	8.29421132	10.7052082	10.4408079	10.4821656
11.0438879	10.1187387	12.0472218	10.0475643	13.4179211	13.3163607	12.7395522
11.2151174	10.7582582	11.6497443	11.1577941	12.2016112	12.7033203	12.2647241
7.45756557	6.97391085	9.02012073	7.4187027	10.4952305	10.6777681	9.22651816
5.5566115	5.6069266	6.04558036	5.73763323	6.62435697	6.53493936	6.79786122
6.33350388	6.28603982	7.27453005	6.11702299	8.80353478	8.12109779	7.73010462
8.87352132	8.39324371	10.8616546	9.33859445	11.0931594	11.4833699	10.6283216
7.25312797	7.3549678	8.24115137	7.61358322	8.65598251	8.84571365	9.53447856

6.81113447	6.69626791	7.64682509	7.02507532	8.73142765	8.44944665	8.36861428
5.69185209	5.62934176	5.98857608	5.75875781	7.17532998	6.89448312	6.63844953
7.13883187	7.14673973	7.22794896	7.25162916	8.66562834	8.56311465	7.39371135
8.56123982	8.46915297	8.27505316	8.60659953	9.5916596	9.48915072	8.99975963
7.20706509	6.71487879	9.00748454	7.56996267	8.72292055	8.34515461	10.1249239
11.2353753	11.064864	12.074379	11.0648802	12.6665203	12.6208636	12.6592179
7.29365924	6.81368244	5.56790711	6.93020273	8.41403868	8.22573524	6.15394312
7.06639708	6.62130054	7.86670278	6.58484317	8.71233715	8.86898363	8.96688003
10.8284888	10.5882818	11.5139743	10.8269377	13.3472449	13.4517649	12.6618549
12.0281685	11.7992618	12.955446	11.7064516	13.6563388	13.2031144	12.7029252
6.65549065	5.32466482	5.72085439	5.62128032	7.33580568	7.0826366	7.31200709
6.00664564	6.35953944	7.09467312	6.91400236	7.9050867	8.16348364	7.58770627
6.56036738	6.20948709	7.97259617	6.08234536	8.34327812	8.22115449	8.03666487
5.95946068	5.68301789	5.3897905	5.79017748	7.38501077	7.08510593	6.08070435
9.83693638	9.44617226	9.91069594	9.66963751	11.6333249	11.7916086	11.3242076
10.4436605	9.24356198	10.3380678	9.27646061	11.3571616	11.2514049	10.5005173
8.16518689	7.67696128	9.93511303	7.67329706	11.3179807	11.2644919	11.0435755
7.93609727	7.93296411	8.69289791	7.87958693	9.77688896	10.3615535	9.61628528
7.72532604	7.47278587	8.096255	7.71119684	10.69863	10.6986439	9.90130332
12.1782329	11.9600106	12.3498955	12.0461653	14.0476105	14.0459925	13.3496987
6.34681474	6.08973203	6.75615331	6.11513044	7.29271366	7.43425458	7.0976761
5.82535138	5.65261728	6.42550613	5.52420193	8.21714976	7.83952442	8.25265834
6.18753795	6.35654088	7.00793102	6.00832149	7.43112064	6.41761745	8.25334216
6.01013957	5.75144765	6.11865396	5.80138037	6.5633772	7.11386299	6.30125419
6.04556944	5.95776327	6.30969007	5.9391765	7.23880405	7.55556388	6.95391007
7.44189841	7.19361505	8.23656724	7.24714236	8.74412947	8.89768273	9.10743941
7.39161136	7.1049939	9.06324244	6.97298897	11.3531667	11.2308503	10.4080569
6.16526359	6.15631591	7.0967531	6.32130201	9.05831839	8.45054443	8.65298726
12.041526	11.5998273	12.8446764	11.9573785	13.7878954	13.3533123	13.5207709
7.89995865	8.10876384	8.77014486	8.03941205	8.89354545	9.17658159	9.4252052
5.11924079	6.72592925	5.20079937	4.98961952	5.98220233	8.11077352	8.34182701
6.01383539	7.04698318	6.65936946	6.66340582	7.63347117	7.86100562	9.14683873
8.21305463	7.72081541	8.88951978	7.7101073	10.9385497	10.5639189	11.1111533
7.05469439	6.88311857	7.09453906	7.27902735	7.8146709	8.63036069	7.98372158
7.02163629	6.8673121	8.3910876	6.77669095	9.98691858	10.1417151	9.43623994
8.12982187	8.10074801	9.08194407	8.14787497	9.68477877	9.68979221	10.6238614
5.71563863	6.00893972	5.80635139	5.93037709	6.82700728	6.88276286	7.09771192
6.32170879	6.18946107	6.89235095	6.64633261	8.32753199	7.75806661	7.88495257
8.29212834	8.23604577	9.85568267	8.54109468	11.7519574	11.8197289	11.0413238
7.92322883	7.48228281	7.31632317	7.52074753	9.39676103	9.18850255	9.02383224
7.17896954	7.09352743	8.60664136	6.63734086	8.8173377	9.23933837	9.2324689
6.00409931	6.03865807	6.03637036	6.14179367	7.68832795	7.56295138	7.34748318
8.24191024	7.7659769	9.3164112	7.42460134	10.8493824	10.4759198	9.64070859
8.67435013	8.40540274	9.8340935	8.54070771	10.7112153	10.2406053	10.3599834
7.63794999	7.43785692	8.40972357	7.72572616	9.57709833	9.38145232	8.49639204
6.35062754	5.86660858	6.44683551	5.75361176	7.38646178	7.20912287	6.81889784
5.68301171	5.57176023	5.88237845	5.74448755	7.28649215	7.29784529	6.64782535
6.86835094	6.8156177	7.70094319	6.52360414	9.10238834	8.84742291	9.04821548

7.35060598	7.47313018	8.00487775	7.40324812	9.01576767	8.97622987	7.95799757
9.73929406	10.0509207	11.3474082	10.2659807	12.056935	11.5862013	12.4406317
5.69061404	5.41622633	5.89463663	5.26474622	6.3111138	6.43025466	6.22196599
9.13464836	9.62113043	10.0217823	9.51475508	10.7021891	11.1740462	11.872586
6.95538256	6.9841769	8.24686313	6.95799583	9.18539184	9.25074371	10.5386371
6.2739703	6.25454038	7.35557662	7.02821109	9.27130451	8.01276252	7.54048998
7.1472548	6.82987619	7.34974476	6.62474063	9.42143897	9.52664458	9.02213114
5.6536144	5.53632835	6.08757719	5.5806454	7.3244371	7.0556577	7.19609651
7.9614387	7.33913649	8.99760122	7.3001045	10.8987095	10.6618805	10.3294466
5.85642852	5.71477523	6.04739923	5.9547963	6.68578461	6.76866338	6.61058816
7.37862866	7.17294749	8.61794675	7.01760684	10.0003015	9.90857906	9.36113362
6.417865	6.31383536	6.41205763	6.10719238	7.5436691	7.49868967	7.53236606
6.53963746	6.25793238	6.62241486	6.11574929	8.39678029	7.57861045	6.93697119
7.00322442	6.39652961	7.32307729	6.84666506	7.77290638	7.67600077	9.19784205
10.275773	10.2668416	12.5631121	10.7756597	12.7168373	12.4221116	13.7658807
6.59495321	8.17535942	6.62064036	7.38043769	8.79111599	7.05849076	6.71612529
10.2581573	9.73304465	11.1836912	10.1388349	12.5369548	12.3420477	12.6029422
6.04021497	5.9376144	5.60666667	5.93153675	6.76848739	6.95240929	6.14353567
7.75518269	7.31981209	8.67162846	7.81874833	9.4298536	9.2963475	9.68144013
13.2546434	12.5335259	13.897143	13.2326076	14.5911372	14.180164	14.5424793
7.26548284	7.09625026	7.71310465	6.71490489	8.91399608	8.53461299	8.64325266
11.7184935	11.50446	11.7765379	11.9124676	13.6598867	13.8289199	13.085211
8.74432662	8.49497755	9.32346037	8.87024572	10.5482526	10.3093081	10.8065955
11.0967501	10.7488813	11.9914684	10.7681173	13.1589342	12.9119284	13.1575213
8.6394847	8.38537452	9.6637415	9.31521366	10.4690485	10.2048462	10.4187255
7.96242987	8.04643977	9.11413143	7.78650031	10.1470115	10.1635981	11.0680353
6.06627135	6.38300576	8.0819675	6.47547275	8.30991239	8.3669774	7.88936524
9.56849238	9.04929641	10.1550434	9.2322108	10.5965877	10.7558006	10.6016807
5.42166328	5.20201174	5.27787059	5.03248263	6.67871421	6.69108884	6.27764207
11.5168519	10.8947556	10.1070156	11.9610401	12.8552707	12.5955279	13.1241283
5.89469701	5.66765516	6.58257241	5.66465918	8.04880726	8.26758636	7.94734472
5.8543054	5.49021776	5.79935805	5.67325055	7.36470987	7.19535646	6.58046662
11.2997152	10.6195066	11.809482	10.4809033	12.387302	12.0726703	12.0397567
6.35661342	6.16863462	7.2534022	6.09861978	7.89734632	7.91229418	8.24679142
8.874702	8.43247926	10.0656221	8.44043692	10.9599042	10.3527519	10.1410419
9.15662869	8.53977377	8.82715309	8.83833634	10.0568599	9.92533917	9.06402461
6.09366686	6.06940143	6.93333776	6.4528056	7.50550274	7.70856869	7.9064024
8.86782513	8.83305705	9.04507214	8.58831062	10.5177757	10.4635509	10.132663
6.47884629	6.35313059	6.8622096	6.56879537	7.88470536	7.77147164	7.7436146
12.3533806	11.8098176	12.2722396	12.1945833	13.9788945	13.8892815	13.4623987
8.3738074	7.99409769	8.88858771	7.93693528	9.75783917	9.99366587	9.26751777
5.44349327	5.37168117	5.82131225	5.64621768	7.39905827	7.1146856	7.49593016
7.82727347	7.48501699	7.79545203	7.26391797	9.01641665	8.93887826	8.39987731
6.01486653	5.98202796	6.84938884	6.16076997	7.88248104	7.38572093	6.84175303
11.785589	11.1097347	12.80756	11.7606734	13.2079073	13.2156687	13.778803
6.30222509	6.15554181	7.1028817	6.47170691	8.26205161	8.29236995	8.40427686
7.10255526	6.79806348	8.11058249	6.77644751	9.84468254	9.87726803	9.06044925
7.12052509	6.91832541	7.6233343	6.88044555	9.01838409	8.89902877	8.72373704

6.45304193	6.17923076	6.65836003	6.42720556	8.68685626	8.71481287	7.97111528
5.97993456	5.87277829	6.64548552	5.68385868	7.27606484	6.85541669	7.01649521
5.65811292	5.74569637	6.1752653	5.70979764	7.30536958	7.27073449	6.84635165
7.12310021	6.7121404	7.06939606	6.61614035	7.94319992	7.73161229	7.81761686
9.62524583	9.19418779	9.83115647	9.51457323	11.794058	11.7856838	11.0843905
8.68312726	8.18512321	8.93950517	8.15919876	9.42648701	9.49679647	9.48441046
11.3198335	11.1128291	11.2590936	11.1773211	12.8700979	13.1153032	12.4129876
8.09985589	7.95207201	8.10951728	7.69860814	8.87753384	9.07629667	8.60954482
6.50032421	6.15232947	7.04387706	6.76920444	7.40226001	7.96726143	7.13141766
7.71583847	7.62976533	7.96561692	7.86566833	9.15778448	9.67191607	7.82387225
7.39867615	7.19664638	7.79946944	6.63574032	8.74766231	8.56406563	7.8707913
10.775723	10.4268989	10.7515357	10.488746	12.7755586	12.8469146	12.3033635
6.24343279	5.84871081	5.90630883	5.9139935	7.15227267	6.87814535	7.02077331
12.961237	12.6533348	13.0787579	12.861602	14.5584016	14.4826926	13.9290364
6.37817154	6.13406175	6.72736096	6.35610207	7.68665179	8.03349789	7.32293042
8.86069996	8.37350032	8.67631079	8.61875329	10.7741636	10.768793	10.337371
8.41473428	7.58110264	8.77017262	7.43339599	9.97907216	9.59532574	9.46318042
5.86921035	5.92170243	6.32285239	5.99144112	7.20510607	7.10199562	6.44654855
7.21446581	6.89526142	8.35470736	7.44900416	9.30174036	8.44200254	7.86677077
5.60664446	5.64119283	5.65067066	5.90687425	6.6209057	6.72326229	7.0462946
6.78611408	6.37963171	7.36668241	7.06864502	8.13414083	7.77655476	6.97853773
5.40657749	5.39735599	5.5326137	5.53627457	6.31074273	6.33829515	6.36806656
5.70543057	5.58645102	5.77359869	5.63796462	6.86331531	6.90651304	6.38901038
6.00609736	5.7272218	6.05663663	5.90233556	7.58067162	7.13478567	6.83221154
5.71025762	5.60257031	6.14548997	5.87672075	7.24987194	6.94960146	6.52992118
5.48539913	5.2765886	5.52375608	5.34246692	6.29783227	6.3200994	6.22112326
5.66106348	5.66844367	6.27628616	6.01968279	7.32542869	7.13394055	7.3561667
7.05569674	6.55558105	7.98516283	7.06496151	8.67065944	7.96689416	8.73075882
5.77144272	5.75654618	5.6414417	5.74820734	7.15278171	6.62453127	6.73266266
6.05123896	5.71990516	6.71739403	5.89542672	7.77486413	7.32374385	7.00797361
5.27657185	5.22576657	5.46532739	5.38994017	6.34731964	6.35502966	6.09542319
5.82931505	5.5706296	5.69011059	5.79237802	6.71567377	6.49842146	6.37626972
6.19479329	6.04669033	6.44776233	5.79886239	7.52956018	7.50405305	7.30808688
5.33416636	5.38521013	5.53132968	5.41670972	6.57790597	6.52900725	6.28783518
6.58667393	6.2590587	7.52326555	6.90128205	8.2530698	8.01369059	8.09410197
5.6106605	5.34595339	5.97959965	5.4982164	8.27984919	7.82819933	7.13305382
6.53250508	6.29723636	7.32513992	6.62256978	8.4414673	7.94025839	7.58917922
5.62205153	5.65815006	6.23324923	5.86339114	6.99695021	6.80768464	6.469219
5.58493665	5.55579315	5.740552	5.69920819	6.55039065	6.53621146	6.35458333
6.9942018	6.85368009	7.70272887	6.82798919	8.88148201	8.86894691	9.54694576
6.32522233	6.15231263	6.93168069	6.65747995	8.04773959	7.68600811	7.43205828
6.57906789	6.31161773	7.12240457	6.6694662	7.88478472	7.51129962	8.00864039
6.83360233	6.53146053	7.31574826	7.10412695	7.74914002	7.6696476	7.79531045
5.66738437	5.53306181	6.27305518	5.78873921	6.93019918	6.88737417	7.39967014
5.10393945	5.07158936	5.40412892	5.24075966	6.21897679	6.07729193	5.92243431
5.97347145	5.87237249	6.14136106	5.69689911	7.51172894	7.41076169	7.00555157
5.48903806	5.32043305	5.48624552	5.68324337	6.37267338	6.35324088	6.47026841
6.01935254	6.1687782	7.8149131	5.98368636	8.46530029	7.87539674	7.81479713

5.30901862	5.24747261	6.27235676	5.42795407	7.03534018	6.62717154	6.84978039
5.76652414	5.73941177	5.84791425	6.0616006	8.36210821	7.79378081	7.27131455
6.08843379	6.12063395	6.05237611	5.86025518	7.46164069	7.14773814	7.73888806
5.40624039	5.2584149	5.76945863	5.52267348	7.17073128	7.13312474	6.9028478
6.09395604	5.89305709	6.53511049	5.68018762	7.51231381	7.6003493	7.4968418
7.00155232	6.03641586	7.37136545	6.32107449	8.03193604	7.69452156	8.23625055
7.34523934	7.00931134	7.85225546	7.15039416	9.85222908	9.90590803	9.29568216
6.76800828	6.53154506	7.78250096	6.62786798	8.42813569	8.12766887	8.25545541
5.37375434	5.31613633	5.48455741	5.55612619	6.27868362	6.3360525	6.16223824
6.1061454	5.90372426	6.44173162	6.9729706	7.47207749	6.97382133	8.44912305
5.41209388	5.41026342	6.13619098	5.4522386	7.39839569	7.58332502	7.15303819
5.97840387	5.81029271	6.80597984	6.25621624	7.5910226	7.2324948	7.21407331
7.53715005	7.22221819	7.79342969	7.82206217	9.10464828	8.66045056	8.73059309
5.73700824	5.69543405	5.65542425	5.8109199	7.02382642	6.65453865	6.52279634
6.21266758	5.91497489	6.84507851	6.20542399	7.64347166	7.26842129	7.44801551
6.52000683	6.439674	7.28912438	6.91295878	8.37809323	7.86491412	8.17511986
5.51158695	5.40335806	6.23222424	5.65533408	7.13130858	6.64416345	6.96176222
6.19567516	5.70256696	6.21481723	5.99464014	7.6555987	7.43975111	6.80602755
5.62633867	5.76783989	5.78017254	5.64281101	6.94559717	6.524705	6.44103456
5.2626564	5.09087206	5.29163339	5.21574343	6.04687866	6.09119882	6.05125506
5.42645498	5.34412402	5.45787135	5.17806205	6.11835848	6.19000498	6.10202251
6.16244571	6.07595314	6.9874971	6.118016	7.57708058	7.62268808	7.46596222
5.44306189	5.24875607	5.36648798	5.15623439	6.30064267	6.27374637	6.13116307
5.91023593	5.82785763	5.80892995	6.02257333	6.95137681	6.99705751	6.34780064
5.43348095	5.38068997	5.74329603	5.37632164	6.74118246	6.20765765	6.3454881
5.31428795	5.20649593	5.62860243	5.09070314	6.18454572	6.14022384	6.06304966
5.98981091	5.73474294	7.45219264	5.91983202	8.13243649	7.79764207	8.15202721
5.94923889	5.85161089	6.41105163	5.95526072	7.69197936	7.07626727	7.25574148
5.58982939	5.53810045	5.64475096	5.51510246	6.69891159	6.57131017	6.45222427
5.99625993	5.93152635	6.92045767	5.78116192	7.95473082	7.57967044	7.33175626
6.271717	5.8078433	6.73176976	6.41667662	7.98032069	7.65084568	7.66084664
6.25554322	6.02889421	6.31736775	6.16311406	7.09421284	7.0894865	7.37267269
7.86790388	7.41945799	8.14779918	7.01854182	9.84631278	9.62810374	9.78412163
5.784482	5.6430853	6.45261438	5.8060064	6.87143463	6.96695067	7.70784294
6.54872837	6.32121198	6.31547113	6.41722232	7.25632099	7.47898474	6.84656477
5.79475999	5.50111299	6.33727008	5.72088913	7.318494	6.95984665	7.61267686
5.85263337	5.60584157	6.08160678	5.67719419	7.34339805	6.80321929	7.55602393
7.16619924	7.02482496	8.31698653	7.11185032	10.3917548	10.8297338	9.8414527
6.67037583	5.74827257	6.85201882	6.1443605	8.13066322	6.69371415	7.6387523
6.1629393	6.12764545	6.75967917	6.71436642	7.56339307	8.60245948	7.37550867
5.31264848	5.39398346	5.76541699	5.33975988	6.84830245	6.47044153	6.44318724
5.66888272	5.73941042	6.02276994	5.86762346	7.85591061	7.08397175	7.12454734
11.5911329	11.7986994	12.6601185	12.0371893	13.141405	12.9453057	13.3431232

C036_NS	C030_NS	C108_NS	C119_NS	C056dup_NS	P008dup_NS	C056_NS
13.5154399	13.922275	13.7668291	14.1381006	12.8434114	12.6240243	13.0998273
7.41168554	8.17614173	6.9200765	7.19321387	7.48314102	7.14349954	7.47058404
7.61636578	7.03870614	7.73981031	7.80498348	7.50979717	6.89762646	7.44644483
7.92847219	7.94091845	7.25197183	8.00081974	6.67123817	6.99280015	6.46873188
7.47822034	7.35543303	7.92463854	8.58112901	6.20948086	6.18470975	6.08029567
9.3356514	9.10862717	8.43156906	8.36776069	6.89684944	6.65685099	6.97633958
7.82148254	7.65599861	7.68098183	8.01205014	6.65534467	6.11011932	6.58891098
6.7840642	6.45658112	6.8103287	6.98220415	6.17651327	5.88351232	6.02547691
8.53498486	8.04859858	8.43155026	8.92860661	7.13111717	7.26131172	7.00409909
6.88034024	6.48372373	6.90641563	6.9677381	6.55827223	6.37757409	6.61576072
8.09957768	7.55700871	7.90505721	7.81323415	7.21060316	7.10251852	7.19818472
9.5483821	8.98953472	8.62192549	9.15678845	8.19667048	7.3883368	7.99311615
7.3651816	7.57703827	7.30667784	7.59773526	7.63354601	7.76239533	7.7414326
8.16140855	7.69546634	7.99735169	8.06907162	7.5801485	7.10523492	7.45673552
8.23860011	7.11961556	7.06269811	7.64958816	6.59141329	6.69203517	6.62275806
8.4107405	7.63699503	7.66031828	7.71264599	7.26733635	7.03728337	7.27434833
7.62632232	7.57039993	7.75104857	8.61590546	7.72783607	7.0297179	7.58070252
7.93057	7.49069367	7.66115957	8.27346787	8.04473563	7.66240349	7.9714763
6.39603187	5.98250651	6.52200975	6.33494274	5.74980589	5.67771401	5.64633204
7.86825586	7.1670297	7.46644522	7.15297737	6.43063286	6.23922827	6.39756135
8.49809566	8.58066258	9.0644445	8.60398011	7.76339896	7.96382031	8.1166096
6.71123195	6.35634333	6.44572224	6.56827248	5.93211008	5.62205153	5.61616858
9.63779262	9.01021534	8.87561913	9.071221	7.60965229	7.65431595	7.85511639
13.0319107	13.263249	12.5602934	12.7465234	11.8876437	11.356239	11.9959932
13.2940434	13.2498764	13.6059343	13.5363869	11.4338885	11.6118608	11.8003438
6.56774348	6.55334729	6.76594708	6.7933575	6.08975618	5.64003839	5.99033205
14.2192343	14.1265666	14.8025184	14.655353	13.633262	13.5608857	13.8840656
9.59662865	8.59209856	8.48845476	8.71117756	7.67331756	8.00080735	7.58715268
8.96559528	9.10759242	8.62870179	9.01136956	6.90976711	6.62306677	7.16185947
10.9265036	10.9521027	11.2382142	11.374307	9.66146637	9.57796804	9.36676545
14.7300657	14.483036	15.2731542	14.9537395	13.8034914	13.6531642	14.0973894
13.3201744	13.3239808	13.4511081	13.412026	12.5187756	11.8793037	12.6621329
7.48895438	6.89104121	6.98580324	6.99462498	6.11619235	6.01232843	5.64836506
8.31356513	8.93069417	8.45663738	9.00753191	7.95386759	7.75314052	8.28420271
6.22086119	5.75819736	6.354358	6.23680944	5.75311885	5.39126422	5.43476432
12.9799719	12.9926571	12.9878301	13.2275123	11.9077169	11.2850438	12.0411417
10.0168612	10.2432418	9.93447463	10.6823828	9.19910202	8.74887176	9.81084007
10.8152744	10.4851028	9.99154594	9.88775951	8.79536598	8.18335947	8.94978091
6.73945795	6.88535716	6.81967712	6.98174308	6.65291379	5.88408967	6.4832739
7.33655922	7.75176564	7.61185475	7.80155511	6.80588443	6.72705594	6.97107906
10.3699888	10.5592012	9.89443097	10.3844524	9.33665674	8.94242595	9.34006884
9.25652079	9.46277443	9.05075553	8.58803697	8.7135642	8.43159998	8.75531424
7.99452316	7.06279091	7.40604595	7.03686924	6.54774517	6.41418324	6.45781555
8.12238593	8.27735715	7.15926487	8.25258455	7.79701233	7.19448306	7.685889
7.25580095	6.94746629	6.94045456	6.98486957	6.34271296	6.07881048	6.08081327
10.3275538	9.99884425	9.8259527	10.0999066	9.29701919	8.83392737	9.37621049
7.76412451	7.76187564	7.42402768	8.00481484	7.86168682	7.1400557	7.94306861

7.83449317	7.9012549	7.6157363	8.04928007	7.32201916	6.80993015	7.34424353
7.89030563	7.60617866	7.29009444	7.90521309	6.98894367	7.24471109	6.78720389
10.8659026	11.2457161	11.2016032	10.8611055	10.1129777	9.16341042	10.1084905
11.4421126	11.5568427	11.3105185	11.4497099	10.3290866	10.1151269	10.4257687
8.12415285	7.81278763	7.84264514	7.70166341	7.49830114	7.25095116	7.2785842
6.76441384	7.12534734	6.88675132	7.33983219	7.19230071	6.22361755	6.77917566
15.1663997	14.8414892	15.2433332	15.2742777	14.231477	14.3069802	14.3288243
8.02160187	7.99425782	8.65899654	9.26833801	7.2465525	6.97416217	7.34959086
6.32786248	6.20637246	6.50700538	6.5194032	6.50124502	6.25107712	6.32394087
9.94828838	9.99834502	9.87700937	10.0627154	9.35804477	8.32641511	9.50359958
6.76736348	6.40767464	6.64860062	6.57344103	6.11434578	5.77323971	5.89865362
8.91531384	8.56600531	8.76346204	8.49419877	7.69806008	7.56999759	7.80769752
10.8176137	10.1666929	10.2377167	10.8710412	9.457646	9.1206381	9.43346464
9.19704808	8.24513909	8.66312185	8.51877077	7.51639737	6.5689016	7.75325085
8.32437769	9.06300348	8.05089997	9.07470639	7.59165623	7.34779763	7.57314045
10.3868918	10.3826424	9.69903327	9.80695925	9.36412415	8.38670578	9.34400312
8.51979978	7.36872296	8.09739589	8.172881	6.85703049	6.69718861	6.88201204
7.17205241	7.30272299	6.67397753	6.95012076	6.30746423	5.86357005	6.07732676
8.33001917	8.13826873	9.00455476	8.36385397	7.07254639	7.04600205	7.33001514
9.82141542	10.0193572	10.1877059	10.3587896	9.19623302	8.63335165	9.10291581
7.80729374	8.15457562	7.63614969	7.95464417	6.7844608	6.58863144	6.99812779
12.5690066	12.7921566	12.3961638	12.6815714	11.8063249	11.3343817	11.5799513
9.86984951	9.75561618	9.77249405	9.73447593	7.87325154	7.79757204	8.04560351
9.33958682	9.38462814	8.65252199	8.72311618	8.41119191	7.86716938	8.43667574
10.2042789	9.63284256	9.52887113	9.94352052	8.35277176	8.17522867	8.62858917
8.46361224	8.85792005	9.67871104	9.73867125	9.15987941	8.87781816	9.19387029
8.61244208	8.11998691	8.40906842	8.49448802	7.10451803	6.9690434	7.20836411
9.52049631	9.23818187	9.26632555	8.29254941	8.57636605	7.89135964	8.55470587
10.7495326	10.7690849	10.309266	10.2469828	8.95037218	8.87507845	9.09632195
7.10601847	8.94604098	7.00683321	6.95759776	6.21776852	6.16663612	6.10834233
9.67614238	9.64842661	9.12875221	9.1629839	8.18940058	7.72544602	8.161147
9.88125639	10.136117	9.84044451	9.84911184	7.96393876	7.81035505	8.48829126
10.7315498	10.5582791	10.1871702	10.2841092	8.96623116	8.56524606	9.10218858
6.67323698	6.90817643	7.88567014	7.60828218	7.02924968	6.92698263	6.86981713
9.55932642	9.55482365	9.34111492	9.59024439	8.60252963	7.61158922	8.41541897
6.54589132	6.40851657	6.66133204	6.63350924	5.99532939	6.04259531	5.62053656
8.82876548	7.69686184	7.70343367	8.06030251	6.90855234	6.77610794	6.82626628
8.13908112	7.61907475	8.44501692	8.06896364	8.13608934	7.41255969	8.14398466
14.3536086	14.2052602	14.1210737	14.0975787	12.7320347	12.4484351	13.0704142
11.3139646	10.9075314	10.7719153	10.5698575	9.98782961	9.11279317	9.98006673
6.1593222	7.91672384	8.45662588	8.07761741	7.53436203	5.4678142	7.53530104
10.0054267	9.72796333	9.81408621	9.83905069	8.30053835	8.2373988	8.57737784
7.59773079	7.69919625	7.41172536	7.79904974	6.86160928	6.48491084	6.72027847
8.93370157	8.55735418	9.59565676	10.1094977	9.25918467	8.92180435	9.10065787
9.14862733	9.29194791	8.76498999	9.20791774	8.09305411	7.89016516	8.22879705
8.74377682	8.47140534	7.53644539	7.67684989	6.42164931	6.24219586	6.12701621
10.436014	9.48613311	9.03605957	9.82354016	8.131697	8.80687953	8.18036024
6.81620673	7.08376013	6.57316136	6.79308173	6.29162437	5.8520905	6.16142478

8.36142054	8.33116104	8.69242371	8.68333503	7.30245615	7.31509521	7.62752951
10.0598528	10.0564372	10.4913531	10.4004846	8.32436014	8.39959025	8.84552643
8.21319562	7.26610906	8.41377304	8.44760771	7.34273429	7.37334892	7.45526885
9.90480876	10.1234606	9.7396284	10.102208	8.75436476	8.13522189	8.72238883
13.2963413	12.5271217	13.0741609	13.118417	12.0463615	11.493185	11.9594626
6.61176669	6.34445144	6.71341676	6.96576928	6.45156193	6.29365906	6.50366199
8.40863078	9.18100562	8.48743329	9.23987845	9.3074365	8.66207266	9.23267634
6.82641375	6.46340748	6.87105015	6.97515783	6.51294774	6.24769344	6.39715419
10.5528641	9.74551417	9.04430094	9.37246854	7.86305693	6.8204011	7.83972281
12.3225521	12.9650611	12.727044	13.1896396	10.3357655	10.453035	10.5283721
9.11465983	9.09810106	9.39090108	8.95739026	7.70303285	7.49809925	7.89109044
11.9703594	11.8525494	11.5560332	11.8197692	10.8204822	10.4099796	10.7319308
12.2287688	12.208467	11.7039745	12.1812995	11.0978465	10.3722502	11.1495465
6.69066712	7.4996498	7.36886174	7.60878116	7.04288287	6.1590737	7.07497633
8.78563196	8.1861509	7.77941798	7.5797632	7.17893668	7.83688606	7.08953417
9.75783317	9.9334524	9.40081007	10.233084	9.19849391	8.93837813	9.45141967
6.32999738	6.15296075	6.64472155	6.75417667	6.37113192	5.84785939	6.21164421
14.327147	14.2998248	13.5730436	13.7614567	12.7071167	12.1277729	12.7973871
6.70186922	6.42912416	6.53091816	6.38318984	5.8642649	5.94957368	5.4390925
12.6173942	11.8880415	11.9169625	12.085889	9.53491466	11.6451504	9.81505821
11.4786923	11.7837992	11.3277025	11.9136958	10.6721973	10.1580416	10.9746641
12.2029839	12.6068223	11.8955011	12.0343801	11.3176868	10.7732785	11.4135851
7.45823024	7.56324967	7.61563332	7.61995143	6.89740623	6.45982159	6.88727546
9.82673469	8.77709188	9.31351411	9.32778211	7.37844833	7.62373959	7.45322371
6.77693961	7.02220134	7.04896088	7.27984548	6.87747556	6.67152691	7.05029817
12.175708	12.0713692	11.4980454	11.6254107	10.4305874	9.76033939	10.2852045
12.0107753	11.904886	11.8369206	12.2384785	10.5341414	10.0837585	10.6818112
9.66108419	9.05492524	8.6914888	8.99727575	7.55633438	7.22344862	7.52682863
9.96938796	8.2889837	7.72119354	8.41580218	7.65753283	7.23030967	7.74662382
11.2068489	11.7995245	11.1442599	11.9655646	11.0543411	10.7837988	11.2004793
7.61740281	7.22711531	7.54277188	7.52517624	6.56734024	6.4621541	6.48832653
7.47831743	8.06443668	8.70993229	8.32052845	8.19429394	7.52775692	7.95877715
12.694354	11.9910757	12.138499	12.532998	10.4967969	9.88960435	10.7962287
8.44122575	8.82106856	8.64574849	8.50037504	7.30468535	6.80746956	7.36401556
8.99781514	8.32465717	8.52635487	7.92519777	7.29919886	7.00242237	7.38611771
12.7416998	13.1970961	13.063239	13.4252067	11.7974629	11.6717281	12.0792251
7.49583105	8.35305638	9.31037034	8.23010212	7.06045384	7.74493405	7.18995697
7.79232569	8.06440057	8.36186265	8.01179192	8.23529578	7.08362293	8.18723032
13.0293174	12.3890613	12.5699325	12.7231688	11.5366023	11.1970901	11.4274511
8.50248496	7.83267802	8.11203492	8.06833707	7.17528506	6.71134485	7.26205819
8.33895026	8.4353869	8.90638677	8.90740867	6.97466123	6.70510981	6.91838981
8.05466184	7.86572585	8.4659965	8.4759251	6.70537804	6.66152445	6.82129183
10.1690168	10.4708022	11.4115094	11.4920339	10.5609528	9.73643542	10.6735467
14.5527111	14.4153958	14.7381456	14.9874839	13.2559944	13.4126982	13.6302125
8.36433605	8.76919386	8.50428232	9.35192266	8.31513054	7.98800692	8.52969291
10.8110832	10.8111973	10.34624	10.8061162	10.0022904	9.12242243	10.0073329
7.47943268	7.43743916	7.36172538	7.87642424	6.44512314	6.33910157	6.61625461
7.14404535	7.25798837	7.0535936	7.15505095	6.69034725	6.02037752	6.73498323

6.90742069	7.09284909	6.97733832	7.34929362	6.07762905	6.26833034	5.84642361
9.68336662	10.2844991	9.84900015	10.2660627	9.12384826	8.9256473	9.33072443
11.5322614	12.4519606	11.8065158	12.0465374	11.8303496	11.178892	11.7253867
13.9424394	14.1249888	13.8360385	14.5418541	13.6154388	13.1804952	13.8325963
8.82254183	9.5500624	9.53228773	9.62100072	8.86287598	7.73833821	8.87286692
9.6168335	8.55507338	8.90987239	9.01421747	7.02845423	7.28350883	6.97317271
6.24012978	6.52945898	6.56452115	7.03301106	6.53884045	6.21889448	6.65290232
12.4084141	12.8233123	11.7996819	12.8210909	11.7317531	10.9821608	12.232214
10.7986127	10.8868649	11.2975732	11.2795276	9.98813079	9.39390952	10.0243377
7.33810792	7.23299837	7.38289806	7.29982161	6.49460602	6.28821883	6.26731294
11.9096246	12.2276436	11.4016465	12.0614563	10.9970418	10.5413745	11.2835763
8.71371471	8.80016802	8.45913403	8.76458269	7.86105278	7.84831935	7.77017691
9.49999812	9.9321322	9.239804	9.79986295	8.77216081	8.50600602	9.30915708
6.33062616	6.40928163	6.99951977	6.97196308	6.58579632	5.85264011	6.43323022
15.0533855	14.9510042	15.296747	15.2911214	14.4255202	14.3391527	14.5988155
8.01982143	7.96384689	7.69219353	7.70856318	6.65358172	6.18036531	6.77744996
6.77766562	7.77010818	6.80172646	7.94202238	7.24446945	6.84649332	7.42765226
8.55049747	7.90688098	7.89919739	7.78747687	6.85129055	6.61440685	7.0057907
9.31308165	10.5033577	8.82166462	10.0807922	8.96570378	9.41964528	9.18852901
6.26703384	6.22940142	6.45962424	6.66488527	6.50616427	5.77143295	6.15110769
12.536938	12.5776236	12.130345	12.1411466	10.8806442	10.3151892	11.1487543
9.5357746	9.88018348	9.65368651	9.82588148	8.89151762	8.1487806	8.77997734
8.28247965	7.83203743	8.27662507	8.48374122	7.77504066	7.38742286	7.74296823
7.42241888	7.57390693	7.14123045	7.46651619	6.80390766	6.49724893	6.81887356
6.60641809	6.82150742	6.75761936	7.08883911	6.95012776	7.42605672	7.18115027
7.61535597	7.5777045	8.52444886	7.99283526	7.80628023	7.47590282	7.71218924
14.8748157	15.0321422	14.9447972	15.3263607	14.2335613	13.9007234	14.4149554
6.51390916	6.17504109	6.92798176	6.56982068	6.23814121	5.70968903	6.07889671
7.29851902	7.22257854	6.96749604	7.4852964	6.9258962	6.58699887	6.90469724
6.37263595	6.82976205	6.95916852	6.95007993	6.22052305	5.70811825	6.20582159
12.9808702	13.1010466	13.0502041	13.7281299	12.327618	11.7485304	12.6385346
9.0240412	8.99651134	9.03980957	8.96327586	8.18664664	7.75645758	8.29400127
8.59552902	8.07712227	8.99416814	9.50672034	7.44832283	7.17163919	7.55981681
6.22570161	7.1043094	7.17685132	7.16510296	6.40085007	5.74070256	6.09809758
6.75524012	6.67270113	6.80143547	6.87838694	6.06864588	5.81868775	5.85205404
13.0766954	12.582162	12.3055984	12.3303875	11.3196586	10.6291872	11.3980596
8.34812351	8.81131528	9.27926397	9.36805285	8.5188455	7.5027801	8.64269291
10.6689683	10.7010604	10.9017878	10.806237	9.46487223	9.22258144	9.72011717
11.2458522	11.8842698	11.5553958	12.134356	9.48135223	9.29131439	10.0880422
7.08630716	6.77451431	6.70713132	6.76709064	6.07994573	5.77456886	5.87935289
7.04360589	7.15438574	7.12637615	7.24909828	6.40205121	6.06058181	6.39405397
8.72847089	8.67970599	6.97328868	8.40835989	7.46278199	7.80325223	7.41243859
10.2601104	10.4751903	9.77223341	9.84669373	9.97953095	8.74667185	9.95255533
8.02730724	7.94632499	7.49838492	7.65408534	7.19781233	6.85535813	7.22125166
10.0204953	9.86118014	10.5668542	10.2121231	8.86272258	8.75685684	8.9532437
9.2839588	9.31618603	9.64363328	9.44621197	8.31606109	7.98419285	8.54806434
7.76457241	8.09722943	7.81005166	8.38807758	7.80488154	7.51070879	7.85931611
6.4892963	6.2185828	6.59575949	6.50169884	5.91369008	5.70494913	5.83919144

7.64219614	7.40308789	7.36313515	7.79431059	7.11270535	6.97705663	7.01266253
13.6840637	13.3792416	13.3209345	13.4809972	11.3797087	11.3756032	11.7704562
10.8876966	11.3375712	11.1645286	11.4913085	9.91674518	9.86110645	10.1164466
14.6472943	14.641069	14.2461156	14.7440402	13.8950025	13.6305242	14.1878662
13.4020128	13.0230311	12.8837207	13.027306	11.5048587	11.1340059	11.9313865
11.7423625	11.9006623	12.2180785	12.0375696	9.8833132	9.96930017	10.4377916
13.376352	13.2198474	13.1910899	13.2840256	12.2900125	11.7951212	12.2722271
8.30839364	7.72165018	7.87124117	8.10918502	6.95852256	6.72919382	6.84792051
7.57612906	7.47950706	7.31444911	7.64523763	6.92874931	6.72683258	6.7562067
9.80397835	10.4889538	9.0691283	9.53849393	8.68469042	6.71805607	8.54612855
9.59215987	9.88145845	9.04402165	9.86540867	8.64952277	8.35804323	9.1020589
12.6324816	13.3475729	13.5534337	13.7575171	13.0658348	12.1504832	13.0121631
9.68406498	9.85561057	9.94395545	10.5070592	9.41960525	8.91566072	9.59923279
6.61904451	6.59775433	7.00161874	6.9595905	6.7119794	5.90781555	6.55332471
7.37129574	8.1039665	7.99382435	8.55913423	9.55845286	8.55660032	9.4785579
8.2001095	7.71447427	7.45660658	7.34306665	7.00994839	6.15202092	6.94567207
13.8692989	13.8820077	13.8501214	13.9621875	12.6648643	11.7174708	12.6802747
6.38008906	6.28751219	6.59039363	6.56328354	6.00954067	5.81085565	5.81224714
8.09059147	8.55509524	8.66038852	8.75493459	8.15551347	7.6315576	8.17160016
9.97037772	10.1317657	9.71626336	9.79827316	8.87916662	7.9098037	8.79823979
6.69483702	6.49827688	6.76784677	6.91837908	6.99192087	6.41754791	7.02628551
6.79176069	6.78470812	8.65195937	6.61693341	5.98250834	6.05893747	5.87077626
9.96085889	9.49424899	8.78874574	8.30876131	7.86036693	7.45803333	7.60431378
8.36144598	7.60750121	7.82145831	8.11648277	6.52654775	6.42519794	6.73217964
8.01629128	7.77215157	7.61506827	7.91091371	7.3244479	6.714431	7.28426519
11.4722568	11.2887052	11.0794988	11.8133299	10.6731589	9.70350531	10.9098653
10.5659065	11.2565012	10.0949789	11.1505762	10.5020441	9.64345561	10.6616864
11.2267409	11.3091836	10.3237236	11.3803821	10.2265001	9.69733146	10.6560646
9.62860174	9.30342711	9.423825	8.79203345	8.50348669	7.93731614	8.46673763
8.65532929	9.02342559	8.5530425	9.22076549	7.74021926	8.16772572	7.94577389
12.7832567	12.4788798	12.2748566	12.4063108	11.1740468	10.6617612	11.2144333
6.43956762	6.38297153	6.63210765	6.69655726	6.26057899	6.02795398	6.10397762
10.0355511	10.0762785	9.95110371	10.0571483	9.19348766	8.1521961	9.37462699
9.09209536	9.42193248	9.55889592	9.51901501	8.87118017	7.8506613	8.90068057
7.72942361	7.42166008	7.4890114	7.61031619	7.12245634	6.5070931	7.06258263
7.34615556	7.20645276	7.02456348	7.15890855	6.5005986	6.27747748	6.27864812
10.6532956	10.1796914	10.1110248	9.81434518	8.88674248	8.59504447	8.97524526
12.6768192	12.1705088	11.6429204	11.8643645	9.6832945	9.75914401	10.0705248
14.9437826	14.8008761	15.1384094	15.3506126	14.0686011	13.8202388	14.325862
11.7763816	11.1982081	11.2193452	11.4819694	10.9437507	10.1735535	10.7861634
6.82261544	6.86168248	6.95567315	7.32913526	6.83279383	6.36287785	6.59112002
9.80543741	9.75421927	9.80096136	9.97775073	9.30312134	9.37716454	9.16607243
7.40913123	7.38864591	7.51802677	7.55578395	7.39038911	7.16148665	7.25513152
14.7547544	14.4422185	14.2206496	14.4486061	13.3961927	12.8918014	13.5071319
9.61342115	9.33402339	8.85177215	9.56287108	9.74078087	9.09183125	9.72791042
10.1191785	10.3607242	10.4211812	10.7279672	10.2026479	9.64887668	10.3235221
7.2408333	7.59735984	7.35679175	7.1732134	6.52132169	6.20062048	6.47007151
11.5681913	11.5868374	11.3264508	11.4133852	9.63276624	9.34425862	9.93528294

7.19815918	7.65703113	7.85282723	7.58348373	6.69073695	6.43880832	6.51907982
15.0585093	14.9066962	15.2552381	15.3938141	14.6052891	14.1258913	14.8643937
11.1080049	11.4806271	11.7970633	13.5194593	12.2470505	8.06820485	12.3237168
12.0151087	12.0797098	11.9974043	12.3622299	10.9918187	10.8695769	11.315436
9.93234336	8.6382194	9.25645875	9.67458847	9.341459	8.67457293	9.32007056
10.4147254	10.3364664	10.1793568	10.5222323	8.06776777	7.78232862	8.14625248
7.88995716	8.17582646	7.79637128	8.43339057	7.23713537	7.0849221	7.21570869
11.8326654	12.1792722	13.3650415	12.4653368	12.0826411	11.9008619	12.0335813
8.39956975	6.33682091	6.76817374	6.80609588	6.16282674	5.8303712	6.07174573
6.95164957	7.04909271	7.18593888	7.3141672	6.848494	6.36713374	6.66921248
6.36182099	6.40687999	6.47690413	6.7879352	6.93518038	6.45297326	6.90353966
6.52490288	7.06419522	6.56197508	7.21388479	7.43348529	7.1114247	7.46909801
13.080441	13.7218554	12.2281925	13.3193758	12.6719877	12.1972984	12.4476562
8.32181042	9.23967657	9.21340707	9.50096812	9.27247583	8.89461529	9.19953862
9.43666803	9.49080716	8.89913725	9.49079193	7.57059347	7.69586301	7.66491513
11.3672067	11.1262857	10.2307002	11.0910175	9.532323	9.30903154	10.0447848
12.209952	12.6718646	11.6816048	11.9757355	11.3387392	9.97126189	11.2820256
7.09461929	6.75503978	7.31060006	7.28715089	7.08827519	5.93336939	7.03916691
10.7977045	10.6654123	10.1234024	10.5300229	8.73869962	8.54330641	9.05166275
6.85793622	7.05307114	6.93559411	7.08039012	6.36401818	6.08968288	6.5223692
14.9960219	14.759263	15.0328563	14.9456249	13.7550461	14.0405569	14.1218807
9.03324733	9.34363056	8.92228914	9.43604188	7.9717746	8.11091129	8.59284793
6.72638984	7.46978425	6.839748	6.95698244	6.21828482	5.93885434	5.91383701
7.86751845	7.57288588	7.55703552	7.71916632	7.00955992	6.87942764	7.03926892
14.0800922	13.8225469	14.4985607	14.3347879	12.4502117	12.4689226	12.8855189
8.95746804	10.0896319	8.18013443	9.69798974	9.42313503	9.26097715	9.29809305
6.87573092	6.81011787	6.66246046	6.86280595	5.91003129	6.08875878	5.72663915
6.28185698	7.42365968	7.35949833	7.63047974	6.70961598	5.67328872	6.52886508
9.50258421	9.25711106	9.50847104	9.58529646	7.5396753	7.40724737	7.86618967
8.93281681	9.62637452	9.13375487	9.23861134	9.25793182	8.91905548	9.05183402
8.70558643	9.48850657	8.27586935	9.31040397	9.33405245	8.6284645	9.04912239
6.38695369	6.62060694	6.70308547	6.89921552	6.22257371	5.73717757	6.22909605
9.52275041	10.1313541	9.88024227	9.39153403	9.07986421	8.52857991	9.12031202
7.74691466	7.54102303	7.94513654	7.58218715	7.27200452	6.56985181	7.48871262
8.81159786	8.82945542	8.80004762	8.77333606	7.7684113	7.59586539	7.67210282
7.2975537	7.47515624	7.30181991	7.89976596	7.2210109	6.78437965	7.19863755
6.43455367	6.22669406	6.88727546	6.91114627	6.45318354	6.2368961	6.54679895
7.62406945	7.19402498	7.33663688	7.2399956	6.895923	6.98225208	6.69680482
10.451923	10.476731	10.5885591	10.5476311	9.83411705	9.12147576	9.65474235
11.4721039	10.7896608	11.8619608	11.6587515	9.81917958	9.53724927	9.98545916
6.89623915	7.16287064	6.86068848	7.20828314	6.36654551	6.0536466	6.47074346
6.50521768	6.70505589	6.71688529	7.09821636	6.35910541	6.10634895	6.33118783
7.97626797	7.7429561	8.2462152	8.05273704	7.02885753	6.64669838	7.04771832
7.4478767	6.56847543	8.1827062	8.13558515	6.57224819	6.18150691	6.73168696
6.92890324	6.37593849	6.59331059	6.69444565	5.812768	5.66301978	5.64295105
12.8097336	12.7189877	12.903127	13.3867274	11.4613577	11.131185	12.1614617
11.06547	11.7445526	10.4386276	10.7688179	8.06702341	7.84488023	8.5150071
10.137793	9.83645296	10.2189603	10.0497628	9.09676179	8.673838	9.00042119

9.59141089	9.57222594	10.0105435	9.7681964	9.20798507	8.1237562	9.24505469
11.5599841	11.9588097	11.7971022	12.1373021	11.0875809	10.7796381	11.2409827
7.49766825	7.20323738	7.26780263	7.25769615	6.25807584	6.11021121	6.18156217
9.71963627	9.69192982	9.41953489	9.80238453	9.25545423	8.2660144	9.11064277
8.21623707	8.73112655	8.61451698	8.72209984	7.73876378	7.09669091	7.68469533
6.53179924	6.35991872	6.68478508	6.64786132	5.74086875	8.7346954	5.86810594
6.95732264	7.91240976	7.56325882	8.04891349	7.01323333	6.91362427	7.12653206
10.9358494	10.7242751	10.7381142	10.4631851	10.3139192	9.66718731	10.1362298
9.57049063	9.41765656	9.80479961	9.85573016	8.84217903	8.43684935	8.90709556
8.27743434	7.71622203	7.57238342	7.29918878	6.68173598	6.52020614	6.50998904
8.10954183	7.7077836	7.56032978	7.74359912	6.60144263	6.37703262	6.67815644
6.75115974	6.96136519	6.62144854	7.05154938	5.95428938	5.96171384	6.09824184
11.2453376	11.5607355	10.9123512	11.7767324	10.0236698	9.71768842	10.0170241
8.74122292	8.78447388	8.81057196	9.2498928	7.85294267	7.45905942	8.34221115
11.2739774	11.2499987	11.7060401	11.4285743	8.92974633	9.27487384	9.5295471
6.22860073	7.06758279	7.36981979	7.09632722	6.68781516	5.57512018	6.71213902
6.56249216	6.92255348	6.98473744	7.90618892	6.38397121	6.38637124	6.66252882
11.9231165	11.8271378	12.241842	11.4058334	10.697077	10.2683139	10.8435631
10.0150958	9.68633401	9.64179672	9.76729295	8.99530191	8.03894532	9.07746306
13.0389091	12.5756291	12.6596078	13.0209834	11.5704625	11.3720526	11.4510236
6.16420079	5.83069494	3.28703842	3.3314358	6.45995024	6.21419923	6.31623193
6.68706629	6.2234741	6.64675022	6.42780074	5.83888346	5.88247261	5.62948194
14.5320308	14.5015107	14.2661138	14.7247568	13.7568669	13.5540696	14.1405119
14.9799583	14.6296367	14.76334	14.8655627	14.0603202	13.8117078	14.2837533
11.1518379	10.6660953	10.4128217	10.9481022	8.22312518	9.58176983	8.24471442
6.66215707	6.63620538	6.78504699	6.93206826	6.69534384	6.26057522	6.51256065
7.82597384	8.18139927	8.28917695	8.45972955	7.0375173	7.00093182	7.18345277
7.54703718	7.52470782	7.40674073	7.73150301	7.08576243	6.56224578	6.86107201
8.17353373	7.83451277	9.0111006	8.64253408	7.75062526	7.62448477	7.98613557
6.62555251	6.78758265	6.91140637	7.6398146	6.27921219	6.37397388	6.22355191
13.3808533	12.8211302	13.0622956	13.1135665	12.0603166	11.5861755	12.1502011
11.5943689	10.1591978	11.1404144	11.1477582	10.1643501	9.33308467	10.0399143
10.6499486	11.1954633	10.8652152	10.7754802	9.54474792	9.50382534	9.74559652
8.71513147	8.92065863	8.65203504	8.89155832	8.25442917	7.84413454	8.38285916
9.82287017	9.55164713	9.5203087	9.53470246	7.77968128	7.59768163	7.81624449
7.92472701	8.18806656	8.34404108	8.26947457	7.04795275	7.0401823	6.93461203
8.32096613	8.43958823	8.0223212	7.9411339	7.55887985	7.07818284	7.51494755
9.76432658	10.0903731	8.85705787	9.52186462	7.5541744	8.05097233	7.84363405
7.07621275	6.92333699	7.29001424	7.57003555	6.91697338	6.82350099	6.92478757
8.7192656	8.41833755	8.98704965	9.36944729	7.06710195	7.05152328	7.32488619
10.7590285	9.97414352	10.5975498	10.2727709	8.94369867	9.16816339	8.96209898
12.6485582	12.7244913	12.5456861	12.5700986	10.7636863	10.3364865	11.3940548
12.3347805	12.005606	12.1854952	12.371172	11.5462535	10.8704742	11.7176657
9.59414076	10.5614254	9.34089936	9.23559233	8.54640795	8.7483616	8.81028467
6.66508431	6.45127442	7.01100916	6.78188424	6.26298895	5.98371805	6.05722215
7.23831015	7.55087297	7.8712387	7.55962182	7.80868043	7.1543665	7.61913492
10.5982489	10.949667	11.9304934	12.1769199	9.78476684	11.031901	10.3710164
8.31722899	8.9621901	8.73207718	9.00528545	8.05241209	7.1424645	8.08639156

9.22323694	8.49000727	8.46211573	8.39790305	7.76813668	7.37491254	7.76002795
6.50898287	6.56752811	6.64012113	6.79580071	6.63188786	6.37173117	6.58247185
7.2465297	8.10950317	7.54873176	8.64284884	8.46279385	8.55384967	8.49206027
8.87388055	8.78657219	8.71801358	8.95338922	8.84291882	8.50343461	8.89582124
9.66754517	8.41186389	8.67337834	9.06812905	8.02441997	7.02090218	7.85844358
12.819186	12.6749677	12.3032979	12.4026369	11.5487945	10.905666	11.4176764
6.43661161	7.58289015	7.99446376	7.96670904	7.73945052	5.51539992	7.63822592
9.01629351	7.67637109	7.74243475	8.65882593	7.92408385	7.03666377	7.94177538
12.3833474	12.640838	12.1685324	12.8423016	11.5860793	11.1189372	12.0558755
13.2010527	12.9041791	12.895608	13.1901656	12.5309196	12.2856421	12.4558797
6.45085889	6.93204463	7.80549819	6.93697237	8.42726423	7.68643259	8.45757172
7.11891097	7.17384101	7.01408521	7.4700471	6.77666332	6.15894731	6.6323927
8.4518919	7.91649217	7.78064737	8.41209854	7.10216364	6.54076443	7.06451105
6.1970765	7.24313113	6.7979831	6.85086817	6.48180266	5.42041402	6.28155082
10.7461624	11.2656906	10.8436102	11.2681648	9.92241849	9.73097563	10.0971196
11.1622516	11.0079068	10.8807659	11.0171543	10.0669758	9.89081146	10.1276101
10.4343832	10.6709448	10.6450108	10.8500063	9.2915932	9.0155667	9.62227187
10.5314	9.83454421	10.0548715	9.65690943	9.24112919	9.280893	9.21864872
9.40048712	9.93335871	8.79858391	9.71871866	8.49348723	8.37552039	8.9502281
13.0795988	13.3131268	13.1248485	13.4981237	12.1460563	12.0726448	12.5654023
7.00632159	6.66623395	7.52126436	7.70214705	6.65905114	6.56699928	6.49222222
7.37598383	6.98086617	7.47232124	7.73106374	6.43928387	6.19533027	6.62421219
7.46481936	6.93599477	7.43879518	7.63232348	6.31642527	6.76705752	6.25962125
6.61835687	6.56516897	6.64547975	6.86168	6.77592101	6.09426943	6.60714682
6.93314534	7.10599858	6.77685805	7.06251787	6.51853215	6.13987915	6.61942812
9.41398856	8.79524778	8.76211058	8.6658088	7.89464802	7.30756605	8.03003861
11.1324432	10.9772506	10.2394195	10.3723906	7.96374923	7.95080782	8.5529361
8.31502952	8.12482284	8.05477957	8.38237632	7.08435452	6.58111274	7.4013429
14.1500765	13.6841985	13.3875489	13.514597	11.8939943	11.7843735	11.9427102
9.44207845	9.31771311	9.3348717	9.08962606	8.38438488	8.02851059	8.27011028
8.81768401	8.56054187	7.95009043	7.92712846	6.77878549	6.70556742	6.90604036
8.44163079	7.50551465	7.54036288	8.08744269	7.44434571	6.78801353	7.32091876
10.4419835	10.3838015	10.1125646	10.6644615	8.34136893	7.94926178	8.47146753
7.21998743	7.26086121	8.92778803	8.72176876	7.60477708	7.00543704	7.69741313
9.13522138	9.35252351	8.34862902	9.30713864	8.29345923	7.95706372	8.50634845
9.90802117	9.63317689	9.64727997	9.53945624	8.92390534	8.43165346	8.86666848
6.88565855	6.93771985	7.20359859	6.41817544	6.5147464	5.93779846	6.28393529
8.58363266	7.980917	7.5842297	8.00907503	7.27417685	6.54898416	7.18300706
11.1012121	11.1251765	9.82037078	10.8159628	9.93788092	9.43354121	10.1866256
8.92017567	9.29620225	8.33649763	8.72570878	8.03673574	7.09348834	7.97476963
9.73722097	9.07403627	9.12885272	9.13069823	7.94762116	7.44387017	7.89018644
7.32669553	7.569128	7.25276192	7.35704169	6.80811214	6.58353277	6.86284437
9.69932016	9.82616329	9.83037006	10.4116423	9.25965171	9.33633918	9.5826625
10.9897273	10.7320987	10.4182005	10.2389409	9.55670913	9.18236977	9.59854144
9.08013105	9.07250432	9.47480612	9.3616245	8.01607673	7.70042377	7.89912547
7.06246175	7.32058757	7.37826364	7.42746119	7.03040119	6.79712066	6.88204384
6.38177428	6.48294409	6.74584774	7.05756291	6.90871445	6.23711682	6.88970959
8.99692282	8.63412819	9.28287877	8.79835936	7.7176682	7.77585518	7.76798644

7.57975113	8.60905386	8.61361121	8.78428705	8.84709588	8.44735025	8.7187767
12.4234718	12.2312671	11.7077702	11.1178671	10.9360851	9.63282475	10.9722827
6.22087976	6.10177213	6.67871421	6.62200949	6.68125669	6.4669566	6.50795365
10.5329715	11.1399996	10.70566	10.8343691	10.5380172	9.33279564	10.5024002
9.32467764	8.98336903	9.32947032	9.11803288	7.95755249	7.61312938	7.86706247
8.34681377	8.03407152	7.78012896	8.19795432	6.96993788	6.55205408	6.89037251
9.28071858	8.80235092	9.67260938	9.24542483	7.78723916	8.11857932	7.91224866
6.94489943	6.93692292	7.12916966	7.43343939	6.07551316	5.93452759	6.05747865
10.4569396	10.4953624	10.4540957	10.442929	8.56021092	8.47149558	8.80381534
6.75363529	6.52875258	6.64520016	6.88288878	6.35750309	5.97947504	6.15728286
9.73811726	10.0186564	9.77237363	9.53218973	7.64017985	7.81258165	8.21289126
7.68006761	7.37855237	7.40385225	7.30077169	7.08456181	6.66517529	6.92700279
7.86285925	6.67882687	7.13505358	7.29838614	7.03156392	6.46430886	6.71802866
8.27614628	7.65072148	7.50919148	7.62657931	7.29099752	6.22085616	7.35913634
11.9612739	12.6462288	12.1057815	12.1724428	12.5754161	10.4119579	12.5148405
6.75605855	8.33717765	7.92662809	8.92289506	7.77190538	6.98058632	7.65609732
12.1973193	12.408201	12.5021294	12.14234	10.7522602	10.1312101	11.1010249
6.50077959	6.66888073	7.67341509	7.01480272	6.38528866	5.77645751	6.1393686
9.49596013	9.20929812	8.86909224	8.98178816	8.4785395	7.90761056	8.51567776
14.7936963	14.4936197	14.172965	14.4604975	13.5434173	12.5104245	13.4092835
8.59685652	8.75980764	8.78128987	8.2975514	8.35401307	7.65017073	8.20297058
12.6196725	13.1902651	12.460848	13.1957459	11.8812532	11.5076331	12.5805288
10.4890833	10.5274379	9.19922426	9.94652019	9.07125184	8.23423209	9.1419615
13.1838737	12.9404045	12.6041246	12.8099457	11.258129	10.6328217	11.1695167
10.5249139	10.0268001	9.79030239	10.2230822	9.5266479	8.19467512	9.63262466
9.69210179	9.6032276	10.3758486	9.79765828	8.67281567	8.67295989	8.91539571
7.27356904	7.96441712	7.72743176	7.91775098	8.75102178	6.74112313	8.60954334
10.7399013	10.3066712	10.1180586	10.5588349	9.87706798	9.47729564	9.8018401
6.0619846	5.99227741	6.23403488	6.62289229	6.39776156	6.01156519	6.26367859
14.1091777	13.5744682	12.6501659	12.603615	11.6412295	11.2773234	11.7929428
7.65590705	7.81819165	7.98831886	7.64405815	6.42872162	6.37243954	6.65855997
6.56978697	6.73654941	6.7398832	7.29322148	7.12626669	6.62980146	7.25817498
12.3011801	12.0983411	11.7524271	12.1707091	10.8720561	10.3164411	11.12684
7.42111219	7.72937603	7.8959551	7.90868443	6.86642585	6.8043195	6.78099375
9.85661694	10.4348575	9.07074674	10.2701902	9.73202832	9.00306388	9.83648452
9.13621523	10.125314	9.19785458	9.72343945	9.49149719	9.39212634	9.31268264
7.95909605	7.84739754	7.59574159	7.53429821	6.41793047	6.12042079	6.64895
10.4039323	10.7448448	10.6693165	10.2338042	8.80483154	8.77252309	8.88476854
7.711956	7.74080414	7.89559467	7.94819141	6.83275333	6.74726923	6.71710334
13.101764	13.5945975	13.1967974	13.5722869	12.3242838	11.9793838	12.6739775
9.72224366	9.75866812	9.72918379	9.80621432	9.18346766	9.34483005	9.0073183
6.89417022	6.81725358	7.12082158	7.19001328	6.0886761	5.89679496	5.97904855
8.11233629	8.15260377	8.52994936	8.73911057	7.45537819	6.99858366	7.56003624
6.88451734	6.78472252	6.66698377	7.39554956	7.61774955	6.89024603	7.71936555
12.8150752	13.152524	12.4071987	12.9474118	12.4843094	11.2096197	12.4252353
7.319785	7.26691956	7.29030736	7.82499437	7.32250506	6.34945976	7.4050955
8.88330159	8.90116739	8.4043897	9.21123395	7.86062703	7.62085323	8.36790569
8.85415371	8.41671225	8.85596012	8.83974926	7.98021442	7.82051853	8.18387106

7.79047231	8.10230854	7.85284408	8.31317555	7.3644525	6.87338259	7.60096028
7.28271038	6.67412305	7.35008613	7.06235381	6.64729868	6.37127639	6.51824179
7.11665378	6.98884485	6.88141868	7.05315914	6.49822613	6.11851318	6.44771605
7.65765072	8.00801564	7.90101711	7.8089809	7.54032413	6.89930858	7.47328769
10.6797334	11.1293957	10.729399	11.2785087	10.3089351	9.85948525	10.3329373
9.90839367	9.92740361	9.63613246	9.58895575	8.63072107	8.18183505	8.47241263
11.8852651	12.4563755	12.1997754	12.4617194	11.3799133	11.2136693	11.6028065
8.30056077	8.69247637	8.94890303	8.83255904	8.33632133	7.9066381	8.21775806
8.32989319	7.90347398	8.11160571	8.00093632	7.83446663	7.15844105	7.6742855
8.67982701	9.10278199	8.34270629	8.7377307	8.66060316	8.14219898	8.60639781
8.17886749	8.52879321	8.08909821	8.481024	8.49097679	7.50929363	8.24337871
11.6787885	12.1860975	11.9780998	12.3390076	10.7073643	10.6388323	10.8804919
6.78163511	6.69000356	7.1191009	7.03777974	6.22563008	6.1479471	6.04114834
13.8082654	13.7597873	13.7077139	14.1665813	12.8031945	12.5109815	13.3562346
7.35702761	7.62856219	7.27662693	7.61380861	7.0744744	7.08827731	6.99083917
9.73059411	10.1873471	9.86490159	10.2569576	9.01468139	8.96735712	9.46840452
9.10776501	9.31571362	9.19655787	9.6195005	8.99879998	8.67766362	9.01099657
6.47419087	6.50321753	6.6909911	6.79822034	6.80560459	5.90231698	6.70052295
9.705171	9.77063945	8.00722074	8.64398819	8.6378656	8.53705723	8.64482391
6.5265598	6.42984206	6.83942805	6.78409038	6.41472565	6.07148339	6.21413277
7.06286968	7.15308178	7.29906695	7.80289177	7.81846011	7.58428157	7.66791766
6.21807069	6.16771278	6.4931282	6.49687247	5.93407721	5.9464291	5.76728878
6.63434815	6.72695107	6.88682934	6.8802092	6.02403505	6.12359833	5.84042647
6.432148	6.49524553	6.80609201	7.54236021	7.35935688	6.46442192	7.05653801
6.46314309	6.81115888	6.47168692	6.77733423	6.94602548	6.22732656	6.59931229
6.2510883	6.26240666	6.48291345	6.4486514	5.7106855	5.65678894	5.5537024
7.3082389	6.90258977	7.03262875	6.93345343	6.43730299	6.05003451	6.32048179
8.23367815	8.0308034	7.78939018	8.31268332	7.68438396	7.29647393	7.57753835
6.63129581	6.43062249	6.77815337	6.63515435	6.45005517	6.33029235	6.52359677
7.67632595	7.66938399	7.2934836	7.76651566	7.49581746	7.10483774	7.47092644
6.50491224	6.40998317	6.55625328	6.30486839	5.91016444	5.75068716	5.68123448
6.85702925	6.49249736	6.55734943	6.72473348	6.18985934	6.12830041	5.97235537
7.27759335	7.18622817	7.33263454	7.14459503	6.79980744	6.3084437	6.69552063
6.46826692	6.07661125	6.63993337	6.52460095	5.78333171	5.76199102	5.85621934
8.03304025	7.74020171	7.90608787	7.89862073	7.71961265	6.83815257	7.63548742
7.06168981	7.11254898	6.89688455	7.82942212	6.70024133	6.28861566	6.9789415
8.11001813	7.94720209	7.44503306	8.20354622	7.4917994	7.25922634	7.56340832
6.87052378	6.5787401	6.68955916	7.01726832	6.75410048	6.42106791	6.67932104
6.51452297	6.3740151	6.55073278	6.54418026	6.04166112	5.70236942	5.69708071
9.0818011	8.393027	8.37962616	8.96295498	7.62156913	7.2443429	7.65474485
7.77430213	7.56900949	7.27751152	7.75493626	6.65362185	6.43506697	6.82315703
7.61115098	6.85294767	7.64874009	7.99603843	7.32452081	6.7746474	7.3400753
7.85542167	7.7852916	7.6139596	7.59189704	7.08337082	6.89536079	7.02934797
7.3734368	6.81156094	6.99453108	6.8201879	6.32394736	6.25105723	6.13095722
6.1839551	5.97021635	6.53890841	6.24002404	5.72716325	5.51332872	5.46588046
6.76484241	6.89348114	7.36913055	7.18343193	6.57429607	6.66236361	6.47243984
6.24261946	6.15770264	6.52246274	6.47565546	5.9503317	5.70236388	5.7243575
8.19979894	7.27210253	7.13072087	7.79516496	7.65830576	6.65429805	7.51057905

6.52706502	6.35495211	6.71170964	6.59472037	6.32248397	6.24178643	6.20247551
7.26970925	6.83934742	7.01076418	7.58304289	6.77960906	6.48586279	7.00737044
7.15314463	7.04552488	7.06720523	7.32018679	6.58534281	6.32652207	6.39465321
6.70912656	6.74202778	6.74382057	6.98391022	6.38609632	6.09535904	6.45864858
7.60254447	7.30465067	7.47837487	7.5976429	6.54210123	6.55628486	6.51586239
7.93324922	7.94471148	7.67169745	7.96837189	6.99508874	6.6015493	7.1770577
9.11772387	9.50153011	8.43564609	9.3639225	8.40933195	7.96909983	8.52791991
8.09129362	8.08947061	7.95323089	8.23601515	7.59994185	7.30147882	7.51013356
6.26657349	6.21232478	6.5198155	6.46420003	6.00236048	5.75612822	5.71813555
7.6894718	7.82331819	7.52138687	8.17680047	6.60397982	7.27611882	6.36180292
7.05070738	6.98463264	6.72588973	7.20656606	6.44191832	5.96112483	6.46841714
7.45392659	7.31341185	7.31167113	7.61079454	6.9531884	6.26259101	6.93178823
8.50233975	8.61649885	8.47585338	8.91058038	8.68156909	8.16572255	8.48331921
6.77771559	6.44614091	6.6725894	6.90906622	6.38141213	6.52405176	6.23261663
7.35774992	7.12675815	7.38490287	7.63321685	7.04506067	6.6687758	6.86085357
9.05465587	8.00693529	7.99451015	7.7118459	7.39102185	6.89796643	7.10233059
7.18223841	6.51388642	6.83071421	6.72406085	6.24861737	5.86747249	5.97397099
6.76490343	7.01585323	6.71459584	7.26743467	6.92192422	6.4478848	6.90924147
6.28233249	6.40048362	6.55148372	6.72614866	6.50554561	6.04847031	6.28276356
6.21780283	5.99901841	6.4083559	6.41132285	5.63966225	5.61844835	5.39822209
6.20185848	6.24254687	6.60159342	6.44238488	5.56859825	5.79893469	5.4731206
8.0166824	7.86220402	7.43976939	7.55276745	6.62051559	6.2636078	6.53261214
6.41573653	6.15390584	6.53692541	6.55522319	5.74760653	5.38401733	5.52082388
6.49814538	6.68172333	6.51985512	6.80400321	6.68755625	6.11937568	6.38093806
6.32529358	6.26927314	6.36632134	6.5216375	5.87606061	5.62213003	5.57767565
7.12727937	6.72694154	6.35313412	6.41364621	5.94820111	5.62138905	5.6693307
8.82902119	8.17050647	7.73478277	8.40776572	7.00354939	6.50140045	6.89601143
7.80986972	7.03923273	7.36825762	7.42165588	7.17281451	7.56911965	6.94748382
6.46526175	6.2311096	6.65321626	6.65185679	6.09210382	5.87694147	5.84617335
7.42385462	7.32862557	7.62312282	7.34556137	7.41057431	6.89315103	7.31137687
7.00351229	7.53659226	7.33998625	7.59023897	7.34139784	6.40170367	6.97715626
7.33826395	6.8125547	7.06782369	7.31061006	6.34953991	6.16627598	6.4955308
9.86779101	9.32627555	9.19255765	9.47818656	7.86889785	7.83898579	8.03783903
7.24891523	6.72127011	7.24216041	7.25307406	6.71355698	6.23196744	6.46026011
6.85815137	7.04460177	7.07372706	7.12833052	7.0870618	6.61731685	6.96354117
7.46036825	7.17291949	6.91094248	7.04869829	6.56977375	6.2040098	6.50151367
6.93753861	6.66018848	7.30007063	7.12735883	6.76272087	6.22796937	6.56013165
9.45087575	9.99269622	8.87963869	9.86486832	8.42334874	8.14656104	8.92666515
8.53391954	8.03005572	7.49274248	7.84683301	6.74617301	6.57617987	6.63157928
8.62015273	7.3840144	7.21597266	7.28874429	6.64652847	6.11635519	6.60444722
6.43825525	6.34589167	6.52436039	6.62442102	5.72253597	5.71412598	5.59791578
8.73346179	8.31101713	7.04444112	7.39388293	6.53670337	6.59644547	6.46684113
12.9431853	13.2217311	12.7721746	12.9880498	12.2963097	11.616898	12.2362343

P008_NS	P009_NS	C029_1_NS	C029_2_NS	C048_NS	C099_NS	C170_NS
13.0187699	15.0056991	14.7826804	14.7851556	13.1802989	13.3851991	13.217156
7.39418485	9.05666643	9.01939057	9.07774797	7.51286211	8.0292563	7.78783597
7.24353199	8.03600378	8.18431055	8.33767497	7.26704508	7.45592144	7.54403865
7.02581617	8.78943811	7.66276663	7.66829644	7.60405501	7.23863498	7.46639954
6.23296768	9.57070446	7.65154758	7.67404887	7.50544478	7.35143664	7.05084231
6.80477638	8.54617293	8.73561255	8.83619051	7.68153782	7.46895877	7.20501122
6.6569811	8.95282918	7.96885914	7.85041815	7.06401625	6.95578589	6.70764626
6.02375103	6.77817572	7.00949386	6.74801837	6.16353577	5.88576543	6.74150744
7.28680169	9.69479002	8.82574048	8.83941451	7.40386588	7.55696044	7.89156561
6.30396445	7.36231909	7.32661375	7.24585975	6.13045875	6.44476214	6.933287
7.05979653	9.12679325	8.8566101	8.83337115	7.01808999	7.50163977	6.58520624
7.45712556	9.71011333	9.52558482	9.37727301	8.56418608	8.40295917	8.4011082
7.69397614	8.30784333	7.65954429	7.73506856	6.72796945	6.98591364	6.95394033
7.26172637	8.95745498	8.81174556	8.81303978	7.24097064	7.34329322	7.36730641
6.81367475	7.99862202	7.70942161	7.71577534	7.3044718	6.67646771	7.42944859
7.23897693	7.84512055	8.09326596	8.02044164	6.93398919	6.93715604	7.27204839
7.06543768	8.91140907	9.41879502	9.44517339	7.16933779	8.10271534	7.87644205
7.74187479	9.30712953	9.73403638	9.68519679	7.41632295	7.75619268	7.56254315
5.84800317	6.42855129	6.41994855	6.52317536	5.41306157	5.41480564	5.64853303
6.37969962	7.77135807	7.46256935	7.39347541	6.4379754	6.32689665	6.42369985
8.4728553	10.1059452	9.81217923	9.69243539	7.3754044	7.92933476	8.77164265
5.71222956	7.09112433	7.40485715	7.23550114	5.9278282	5.65103447	6.20888514
8.08941605	9.76858548	9.58229086	9.58407783	7.80848223	7.94828371	8.14083642
11.6737693	13.4926656	13.1916396	13.1321543	12.7312541	12.0587474	12.1970732
11.9440096	13.824569	13.6596648	13.684341	10.2778418	11.6069294	11.2123882
5.74521528	7.04303494	7.17477891	7.03356726	6.01322663	5.97645827	6.46555743
14.0324427	15.3392254	15.3220401	15.3591185	13.3972053	13.8145394	13.9309212
8.12221663	10.1041466	9.43047346	9.3714896	8.64630417	7.99154339	8.20433589
6.80241943	8.91570135	8.37651613	8.29485413	7.53498059	7.38075542	7.38866571
9.35196903	11.8577859	11.3839516	11.3715997	9.86993724	9.45250319	9.83099397
14.0818554	15.1313779	15.1605183	15.0553807	13.2612484	13.640037	13.7755784
12.1037501	14.3829293	13.9488463	13.9160409	12.9313113	12.6462237	13.0180381
5.89433784	7.09872588	6.97338971	6.84420862	5.83990342	6.07035713	6.17062334
8.18028912	9.78902609	9.68932153	9.624087	7.68833984	8.31650013	7.93231871
5.46347468	6.14848329	6.19018381	6.40348963	5.09069383	5.13268911	5.45333682
11.8134826	14.532849	13.759962	13.7571441	13.0304308	12.3983677	13.013977
9.46102344	11.4014758	11.2084734	11.1552167	8.83083936	9.2458329	9.16402143
8.44450681	9.69283498	10.7817728	10.6659878	9.4383517	9.16828977	9.17142077
6.00816662	7.06651763	7.69456892	7.62711506	6.36820768	6.12549613	6.17909638
7.39687788	8.3698224	8.18006678	8.13831942	6.60742042	6.93304971	6.85246584
8.97348443	12.2148418	11.0055158	10.9863427	9.73157768	9.45213744	9.6510429
8.69833969	8.87087142	9.90051484	9.74558895	8.30993194	8.2113369	8.50177828
6.55046207	8.16537937	7.63234529	7.52769672	6.68774519	6.68739388	6.37548729
7.43438971	9.22585958	9.22325939	9.30084215	7.99352889	7.64339445	7.47981259
6.14559187	7.33477473	7.18517575	7.10534389	6.6442774	6.64728572	6.46969765
9.1104452	10.8777297	10.6604185	10.734774	9.4689665	9.44886668	8.9722044
7.28630362	8.5057163	8.59716762	8.34960634	7.41869258	7.1935914	7.63384385

7.10464278	8.52211108	8.85736305	8.89780039	6.66477294	7.34040293	7.30076986
7.39746329	7.88528882	8.52550006	8.59139107	6.90308167	6.75828985	7.17593871
9.46368416	9.85721669	10.4907165	10.5143847	9.90367085	10.030543	9.70688155
10.4515089	12.2430314	12.0825483	11.9722468	10.9649855	11.0289041	11.0681495
7.36773973	8.42966324	8.999774	9.20156795	6.48580355	7.54528886	7.27342917
6.01543802	7.90774154	7.95267137	7.99199908	6.31447358	6.80752237	6.85740256
14.5086571	15.1441978	15.7175118	15.6206062	13.7025634	14.1886723	14.3368831
7.09372601	8.64702951	8.87014491	8.90463703	7.02290928	7.06271321	7.05150479
6.43368842	6.99575698	7.43219277	7.4469096	5.63508512	6.05206283	6.34594328
8.67285031	10.8754553	10.9432027	10.8832413	9.4249139	9.37062137	9.60583617
5.76398567	7.25112258	6.80662695	6.78606048	6.34425684	5.67276759	6.11369279
7.79738463	9.84260271	9.40168443	9.35809335	7.92405058	7.89676469	8.00112948
9.36668394	11.7263996	11.0932532	10.9695391	9.69582648	9.87718243	10.4107524
6.57184106	9.37115653	9.12995037	9.15036851	8.58834548	7.64445695	8.26412635
7.44294515	10.1790208	9.06218082	9.10178242	7.73566568	7.45762876	7.42425869
8.57848969	9.72683581	10.7302281	10.703792	9.81143362	9.40821615	10.1091673
6.86182389	7.20004328	8.27986126	8.26809702	8.37587352	6.92473176	6.61050917
6.05859682	7.2446854	7.6247728	7.55030968	6.57499898	6.01860591	6.79522537
7.35304182	9.40788508	8.73334417	8.73130783	7.16759176	7.63215332	8.00538314
8.81279052	11.0667888	10.6274181	10.540439	9.89028176	9.77737796	9.75453063
6.92048387	7.39220406	7.65630472	7.70225374	6.75345746	6.74188625	7.40625859
11.390737	13.4115984	12.9852149	12.9399718	11.9280438	11.7726107	11.9655643
8.1310971	10.0790314	9.69055119	9.66546188	8.24881706	8.20978135	8.83790793
7.97020255	9.29978704	9.73145328	9.58127253	8.74843976	8.20523219	8.10705174
8.52970931	11.4115073	10.3470688	10.3066496	9.10922211	9.11209202	9.22870616
9.33605616	9.68972198	9.81224406	9.84793225	8.32822926	8.75219898	9.21676356
7.27972943	9.57924729	9.14117628	9.0996805	7.18910402	7.5260422	7.53500548
8.09562383	9.47726245	10.0982098	10.0199493	7.90108953	8.21615217	8.88440835
9.13206756	11.1697797	10.633661	10.5653097	9.42998433	9.02013407	9.31320118
6.16983463	8.16251973	6.79926349	6.79565787	5.67360193	7.88213435	6.37670244
7.90345771	8.79199082	9.9716542	9.91535493	8.17078836	7.70964837	8.12639887
8.27015841	10.5302805	10.4442705	10.3431357	8.53238866	8.67929606	9.16809493
8.95515062	11.1405001	11.0766773	10.9938965	9.87810749	9.5709027	9.53516902
6.96740035	6.82634001	7.4639992	7.27257567	6.20202389	6.35413017	6.7490059
7.90680523	9.55133375	10.2504038	10.2359364	8.96097875	8.25325232	8.92575321
6.08984832	6.83418912	6.75131241	6.74810025	5.49479992	5.82525393	6.0846681
6.97669471	8.51141348	8.3363865	8.26250645	7.45111713	7.05662905	8.03593617
7.70529992	8.90221772	9.29828348	9.26459601	6.68918313	7.68064083	7.94195198
12.6875028	14.9594528	14.9144192	14.8354114	13.4161642	13.2723717	13.7506452
9.38440085	11.6134078	11.651319	11.58507	10.4309062	9.86459444	10.2505827
5.53790421	9.27468891	8.60604352	8.67101307	5.28620953	7.77604607	5.77207726
8.51290595	10.6960147	10.1746786	10.1477989	8.94037997	8.82016779	9.29732671
6.63426595	8.19619096	8.25828241	8.11740882	7.18462248	6.77586703	6.88779331
8.93479482	10.1693877	10.3088202	10.2344917	9.14758323	9.18883261	9.50128089
8.09825532	9.76320971	9.63922203	9.68002857	8.12731549	8.23006993	8.95877889
6.28602892	7.80326385	7.21351254	7.12440052	7.04709337	6.72014574	6.61718342
9.15049393	10.4633987	10.7931595	10.7076292	8.82830058	8.58490577	9.47174857
5.96000054	8.13412389	7.57481299	7.44771308	7.01830813	6.59542056	6.3594711

7.73445967	9.30799015	9.12506367	9.08525016	7.81522637	7.90075392	8.13675175
8.75458424	11.2295351	9.95075753	10.0719383	8.80682154	8.80502638	9.42724055
7.80361969	8.72799021	9.50980449	9.37080029	7.98066	7.79580201	7.34123237
8.18324433	10.7339457	10.3754848	10.4071583	9.13574391	8.97209466	9.43944217
11.8645375	13.6892424	13.4194112	13.3230773	12.4984708	12.2546829	12.6817018
6.75538702	7.31660737	7.23378359	7.06792047	5.57293271	5.94803567	6.30319022
8.96784298	9.89703224	9.82488191	9.69344218	9.32237459	8.95955254	9.17715988
6.2400376	7.98275976	7.73852399	7.82470931	6.02456592	6.52673772	6.71871095
6.96007493	10.6140923	9.56866736	9.46146881	9.78589935	8.15532727	9.67776456
10.743398	13.5205978	13.2580756	13.3564245	10.1863966	11.1886989	10.8299875
7.44570602	9.79356381	9.68318132	9.60855146	8.43523812	8.52201493	8.50207238
10.698439	11.7544291	12.6080868	12.525023	10.558612	10.291691	11.4422412
10.6145148	12.9204104	12.9387166	12.8119857	12.3862702	11.17642	11.6488375
6.31554214	7.04410227	8.65050753	8.49541736	6.70520798	6.45146764	7.36493481
8.05848481	8.11646873	8.14332198	7.99342246	7.31452433	7.3797691	7.82606539
9.19851208	11.2252936	11.2192042	11.0659337	8.74871682	9.39557633	9.31214418
5.89036984	7.27477322	7.84496431	7.76900206	5.65600977	6.28192039	6.38565864
12.3745187	14.3131863	14.5385667	14.3970816	13.5701498	13.1015595	13.6417233
5.91597591	6.29405275	6.80472992	6.5703301	5.25364065	5.52261135	6.65095551
12.110575	11.7189318	11.385554	11.2585378	9.67078547	10.6370867	11.1760653
10.6149012	12.0945452	12.6570884	12.6350925	10.1130272	10.6727506	10.6373884
10.9722575	12.8375345	12.729972	12.5661701	12.108904	11.4233928	11.5572162
6.65219965	8.17624996	8.27858606	8.13596318	7.27186823	6.77691725	6.92142681
7.48488733	10.1953648	9.70769423	9.67675382	8.30284269	8.13293614	8.4315373
6.98011787	7.7648444	8.14712401	8.17614671	6.65713263	6.70262015	6.95552089
9.96185568	12.5611801	12.2377812	12.1245595	11.0988881	11.0313221	11.1085395
10.6359403	12.7491536	12.7163074	12.6334026	11.0557915	10.6907462	11.1325813
7.61760631	10.4926684	9.67272499	9.60734937	8.40969896	8.25091043	7.61306969
7.37880031	9.45922094	9.34189393	9.23969614	9.21647742	7.63590309	7.73818416
10.9848442	12.9873091	12.4429401	12.3255986	11.3883171	11.3427608	11.558172
6.64824108	7.62764842	7.34901313	7.25289625	6.36882125	6.29613855	6.3791697
7.68914957	8.92810172	8.18827382	8.27182996	7.7558116	7.76797982	8.33826395
10.1608698	13.5122306	12.2916697	12.3185815	11.778602	11.1054371	11.6460501
6.94095837	8.63355763	9.14201896	9.06000684	7.67279021	7.48478423	7.9823348
7.1956232	9.28721093	9.33787296	9.25646583	7.8243058	7.71342845	7.60789249
12.2303616	14.2468749	14.1516397	13.9975103	11.6525855	12.2646493	12.0133331
7.89128247	9.77366392	9.60730128	9.56104808	7.51645174	7.35882084	7.53153196
7.323189	10.1166961	9.36089404	9.3237049	8.48993905	7.69067969	8.48238487
11.4844191	13.3818606	13.2054496	13.1348322	11.785076	11.9306353	12.1766354
6.86450313	8.47848206	8.36474619	8.39750479	7.4050589	7.04101404	7.31086358
6.92651068	8.76353773	8.56200081	8.55086912	7.03427295	7.32938107	7.438213
6.84885826	8.70815309	8.02320877	8.10152087	6.50115982	6.71513422	7.06980634
10.1741405	11.2878959	12.0837256	12.0929371	10.4110491	10.2222395	11.7547414
14.1309598	15.2451352	15.3660227	15.2745431	13.3096426	13.5697275	13.6953447
8.34444106	9.91767849	9.90108154	9.95175752	7.73134619	8.28658225	8.02230067
9.34436937	12.0993814	11.909391	11.6753277	11.0607736	10.2406423	9.97355531
6.61536156	8.20067109	7.61733596	7.64773471	6.90277666	6.52003874	7.13073014
6.33487375	7.19237863	7.99040751	7.8181322	6.76649048	6.7843862	7.02138645

6.41086428	7.46396488	7.56495123	7.6015747	6.43280311	6.44821082	6.65462601
9.41283677	10.7887901	10.8379409	10.7599639	8.44261346	8.89884894	8.99800223
11.4785629	12.6231584	13.0002009	12.9151132	11.4298199	10.8978629	12.3431191
13.7163919	15.0256185	15.31878	15.2321811	13.498312	13.6991614	13.8976309
7.91718528	9.98046236	10.4143311	10.4229278	9.59195687	9.00776427	9.53598314
7.23641225	9.91233146	9.39124638	9.42542511	7.7889845	7.89294468	8.08163043
6.42096824	6.75623472	6.70132587	6.59670069	6.08078343	6.45255969	6.11030206
11.6797787	13.3818836	13.9434416	13.9314061	10.9576179	11.7552949	11.666643
9.69189947	11.7398743	11.3966781	11.3804703	10.2999338	10.1389514	10.4825357
6.24763648	8.18628958	7.80405033	7.68797119	6.32534899	6.2970296	6.30168184
10.9552474	12.3678133	13.2688597	13.1466349	10.5041635	11.21753	10.9903692
7.89946146	9.48303155	9.35782798	9.13207065	8.0774636	7.8626324	8.53013397
8.98905639	10.623278	10.6238495	10.5516487	8.32867224	8.84428239	8.65427692
5.71239851	6.9687693	7.16883756	7.17234149	5.69810483	6.31572979	6.19527692
14.5014454	15.4725779	15.5442285	15.4384394	13.613824	14.0644127	14.0842802
6.25661896	8.94419374	8.24018551	8.12493241	7.42298897	7.48032653	6.8957643
7.19752437	8.60655253	8.61366535	8.64396222	7.2535289	7.58318735	7.26969429
7.04256445	9.24467946	8.61696671	8.66860457	7.33137829	6.97388331	7.73238928
9.43857273	10.9880058	11.0714952	11.1563562	9.22981164	9.64112493	9.75510656
5.69379526	6.87423384	6.64016278	6.4948671	6.36383496	6.15629953	6.24690569
10.6093764	13.0235785	12.8967294	12.9682919	11.9076531	11.4394933	11.7459932
8.32693502	9.82435975	9.97195734	10.0233801	9.2110862	8.53847841	8.9047773
7.61446398	9.13542995	9.04792195	9.05771859	7.95642437	7.6400287	8.03440392
6.83574221	7.54899602	8.13253058	8.19852608	6.01891164	6.69271871	6.63262506
7.65020377	8.53065525	8.73092079	8.6961907	5.68424901	6.93354903	6.63875896
7.47392893	8.48731872	8.00221588	8.06433159	7.16328638	7.44580118	7.71545481
14.2714601	15.6198749	15.5022945	15.5688524	14.2650656	14.0925045	14.2885157
5.75347832	6.87037952	6.82557195	6.78839469	7.16550801	5.51588178	9.41201044
6.7010458	7.68374136	7.88578116	7.90025338	6.43752538	6.96104099	6.86252702
5.77168067	7.59256094	7.12800567	7.24497551	5.40992175	6.14668556	5.96031547
12.6240879	14.5427018	14.5122078	14.4822256	12.5291863	12.9141332	12.63037
7.91939133	10.2075354	9.96736894	10.04563	8.99136708	8.78803834	9.07496937
7.29076812	10.1304637	9.44597763	9.45209933	7.60082056	8.08888097	8.29502639
5.76684928	7.59780229	7.58040255	7.57189899	5.52409377	6.21281113	5.73372365
6.12100442	7.14707668	7.01540502	6.98814386	5.71887011	5.91745825	6.15359378
10.9376644	13.1497955	13.2076403	13.2599586	11.7791858	11.375197	11.6870699
7.6045384	10.456376	9.41086597	9.43615913	8.19444169	8.33091867	8.33740559
9.50374883	11.3546373	11.0599244	11.0640549	9.87071364	9.74212094	9.95686791
9.65731899	11.2502582	11.4617547	11.4964569	9.99691125	9.99788513	9.63705573
5.89408144	6.69136945	6.95800395	7.04080368	6.49457418	5.94466662	6.01503274
6.19355986	7.52362154	7.77308513	7.69062941	6.18894425	6.24337566	6.3409601
8.05695846	8.67672685	9.53507354	9.5861406	7.98644109	7.44460247	8.05750605
8.8854981	11.7482134	11.1946122	11.1291284	10.2060034	9.42436766	11.3184377
7.1753719	8.1377863	8.51337586	8.5032235	7.01604162	6.65317756	7.20333724
8.92448148	10.8217804	10.6153436	10.5161156	9.0278521	9.21296152	9.63096582
8.35457117	10.4682106	10.101436	10.0057579	8.89705433	8.91175509	9.23374669
7.86969624	8.54304458	8.82884004	8.80904459	6.83963464	7.56895554	7.61962869
5.76445922	6.66350545	6.58572332	6.74078997	5.50965911	5.6392339	5.81668341

7.20930421	8.44228211	8.37692717	8.34063659	6.31690817	6.80377337	6.9982587
11.9374102	14.2493557	13.8879079	13.763423	12.7585634	12.5649835	12.5643906
10.4195484	12.5537891	12.11269	12.1815247	10.0010042	10.5364454	10.0686668
13.9455591	15.1509559	15.1217889	15.0705441	13.4726337	13.7321982	13.9759142
11.704993	13.9748698	13.7087175	13.6206826	12.4891608	12.3747442	12.2608058
10.5417926	11.513625	11.8206309	11.7073872	9.57154485	10.1496074	9.88790311
11.9336667	13.7979725	13.6862762	13.5550408	12.4357057	12.2613439	12.4238219
6.91139678	8.04701209	8.26196467	8.18364531	7.19601583	7.18524514	7.75577088
6.9019445	8.23281615	8.09488685	8.16652013	6.34642544	6.79010605	6.96209898
7.05056264	10.6032239	10.4292488	10.4700823	9.75758069	8.82261449	9.60509314
8.68709674	10.3572826	10.6297792	10.641248	8.46451512	8.87882893	8.77409387
12.4806176	13.0414177	14.2780545	14.3189544	13.1133425	12.9865594	13.2445329
9.18838337	10.9865681	11.4620856	11.4349956	9.21910655	9.78941969	9.64970096
6.01457512	7.68279519	7.74918093	7.81241158	6.09147537	6.32345582	6.362136
8.6741015	8.79341343	10.2986285	10.1621698	8.26159663	8.24564635	7.78291764
6.49519835	8.14229345	8.34050753	8.372637	7.64688051	7.17954688	7.19837923
11.8759252	14.7628071	14.441497	14.3924147	13.4841117	13.3213052	13.5853499
5.81940506	6.737826	6.87487933	6.92033499	5.46471727	5.89099672	6.23447354
7.89587212	9.29576985	9.31741126	9.25383047	8.10192999	8.22053698	8.75026582
8.30083986	10.5297556	10.4584804	10.3926094	9.19208022	8.87619921	9.08369924
6.66818296	6.99543703	7.14072549	7.40112015	5.77049449	6.12549179	6.35203124
6.07931218	6.67985163	9.3494303	9.32403222	5.79708848	5.79266642	6.13033106
7.73628083	10.050635	9.83513773	9.63459145	8.5207216	7.6148254	7.98025785
6.53768884	8.6940371	7.67722844	7.59205632	7.61867181	7.37592911	7.21105212
6.85563222	8.06442968	8.59300598	8.53354933	7.02246769	6.92580721	7.49248534
10.3664643	12.3555584	11.8288458	11.7450436	11.6966942	11.0947467	11.2573566
9.99855094	11.7596556	12.0585313	11.9976671	10.0027602	10.3690695	10.1098881
10.1672676	11.994882	12.6687944	12.589528	9.59487918	10.3363704	10.3180072
8.19753911	10.5074359	9.88257779	9.98973083	8.91023287	8.62453339	8.89487474
8.40138387	10.7811269	9.93491333	9.80258503	7.61738004	8.22038945	8.28798889
11.073525	13.1837259	12.9278689	12.8379109	11.8375231	11.6031766	12.2581523
6.0802076	6.97912448	7.68213292	7.7604066	5.61752271	6.16746291	5.94635096
8.40212568	10.9861755	10.8913121	10.9194757	10.1420317	9.76382136	9.85782801
8.01245079	10.3622189	9.84784255	9.83424934	9.05047939	8.59092345	8.90422034
6.70962563	8.79130943	8.80114831	8.846141	6.76847548	7.45490051	7.01836155
6.17768264	7.19460027	7.44661603	7.30731467	6.72326366	6.54361203	6.59366331
8.9975984	9.85123932	10.7649089	10.7250467	9.33084765	9.2449125	9.18913071
10.2291865	12.6779486	12.3783344	12.2786797	11.1355964	10.94361	10.9430452
14.313496	15.3581841	15.3878164	15.3907151	14.1037705	14.3379389	14.6476411
10.3663364	13.467985	12.5142118	12.4261311	11.676442	11.3801304	11.630507
6.28527385	7.53619126	7.80261717	7.78618205	6.13013808	6.41245519	6.68385363
9.52577272	11.5819971	11.3956835	11.199749	9.65076007	9.73849089	9.53834784
7.34529967	8.22891054	8.28147571	8.10937101	6.83201781	7.03392841	7.52454877
13.0799612	14.944734	14.9144627	14.8755862	14.1235605	13.2930491	13.6645106
9.2954558	10.3693291	10.5042241	10.5477236	10.2086563	9.72404584	9.7418657
9.91005624	11.312103	11.7627603	11.7440223	9.67334106	9.25720184	9.76749855
6.28838533	7.23220025	7.67915986	7.5421613	6.45399884	6.69160954	6.66200464
9.9059978	11.9627337	11.628657	11.5408081	10.3206652	10.1928032	10.4440044

6.62549903	7.88025268	8.45202786	8.30516434	7.08213201	6.89335008	6.61229411
14.5303527	15.6376033	15.5908396	15.3759086	14.2187176	14.2539575	14.7709777
8.36241203	12.1735347	11.8507147	11.7068456	11.2031761	11.2596521	11.02048
11.3375221	13.1300789	12.9721764	13.0891668	10.9822321	11.3857238	11.2211346
8.79010409	9.90150803	9.84487568	9.78966911	9.71111801	9.35602537	9.03185336
8.22500735	10.9428539	10.0118338	10.1752402	8.67122083	8.32913571	8.74666312
7.32002158	8.57576877	8.60390521	8.54914778	6.70591572	7.38057795	7.28124942
12.4163183	11.7761003	12.6601562	12.6001057	10.7260964	11.5505559	11.4996172
5.84863471	7.98434611	6.41528967	6.52346332	6.77653963	5.91445306	6.53923792
6.4574697	7.70599656	8.19780545	8.18154637	6.30555964	6.58573669	6.71869178
6.55005366	6.46818985	7.50527565	7.52700307	5.46653511	5.67949164	6.29223677
7.34205688	8.28977223	8.61963016	8.52764746	6.34634158	6.96105489	6.65553788
12.4837898	13.8046988	14.2186419	14.1659708	12.7012462	12.2315974	12.9219227
8.96305125	10.4113616	9.78664504	9.65379827	8.70950605	8.42148126	9.95291753
7.9274728	10.0445799	9.39018164	9.44067257	8.11199216	7.96416987	8.7775864
9.93733086	11.3922171	12.0142838	11.8987083	9.36012084	9.83216833	9.90425076
10.3931502	12.643968	12.6731436	12.6606248	11.8549241	11.2932245	11.0291899
5.96140339	7.92142086	7.70456353	7.59622405	7.04788515	6.8323508	7.18353515
9.09924944	11.8550713	11.2820563	11.1871071	9.83475972	9.66516481	9.95280443
6.27589877	7.22216892	7.5945911	7.37163378	5.81456298	6.29901968	6.41756969
14.3974079	15.3185622	15.4052834	15.398245	13.5287789	13.8962482	13.9724351
8.73631804	10.5046363	10.0782242	10.0162191	7.97876136	8.59904287	8.27735715
5.99759388	7.91061695	7.15378913	7.02565341	6.3652985	5.89802063	6.32428714
6.96864145	8.47484444	8.4983901	8.57142312	7.1192576	7.07572729	7.26510116
12.778018	14.4632338	14.3585372	14.2285128	11.8818221	12.552201	12.3430816
9.5015721	9.87438033	10.4949896	10.5144044	8.96337442	9.26287167	8.59335489
5.95753183	6.88234103	6.87303924	7.02341394	5.76091551	5.94772693	6.05604545
5.7910149	8.28369309	7.77743879	7.67094087	5.33080648	6.47511424	5.80276424
7.58987881	10.2328166	9.41719071	9.51349588	8.02281442	8.14458993	8.369733
9.18581527	10.6877072	10.2879559	10.2166816	8.66321438	8.83985286	9.41045093
8.87190924	9.95366306	10.409026	10.2485586	8.60804852	9.08166425	8.42858085
5.89308136	6.60460838	7.10805394	6.95303354	5.98832817	6.00033539	6.20211285
8.82637561	9.82907718	10.8964588	10.8666129	8.89772145	8.96981934	9.21055812
6.72520536	8.6517847	8.91437078	8.99983036	7.67385813	6.88189706	7.74917422
7.78152561	10.0699975	9.38607394	9.34073472	8.21352712	8.04249332	8.35472543
6.94328491	8.25041355	8.38228595	8.41484672	6.58461441	6.87769272	6.88574029
6.28900778	8.93450471	7.97822252	8.21643643	6.52454501	6.66274812	7.07089268
7.11681906	8.53223045	8.19705349	8.16175419	6.79116289	6.65085358	6.89672232
9.40464984	10.7920134	11.4219191	11.4570735	10.0786459	10.0278383	9.94349532
10.1858517	12.4870557	12.1829821	12.0129178	10.5433915	10.4220543	10.7707919
6.56596892	7.35408008	7.88382606	7.80122722	5.75780307	6.31563941	6.30055958
6.12401706	7.22898315	7.58561382	7.39809979	5.76803475	5.97442928	6.24838569
6.8715332	9.08276725	8.33880367	8.39363972	7.46203759	7.21911139	7.49684499
6.6480023	8.09980277	8.05863849	8.04196627	5.97577599	6.50454333	6.65548779
5.5879224	6.63354368	6.90313953	6.91310243	5.57374867	5.79550463	5.81070684
11.8569939	13.9977881	13.9812123	14.0186166	12.2989598	12.4292605	12.227742
8.36939407	11.8708344	10.5089842	10.5034332	10.0942849	8.86168279	9.58723934
8.91568641	11.1158955	10.4259491	10.3792051	9.21300893	8.84069485	9.38046669

8.40041369	10.4527694	10.5140157	10.3610599	8.96942739	9.04774477	9.21252435
11.2442401	13.0093299	12.8084828	12.7493149	10.7876169	11.2073322	10.8894553
6.03243723	7.76701646	7.5174387	7.63632953	6.65066118	6.52182968	6.97608749
8.51391981	10.3885835	10.1996858	10.0422517	9.77866428	9.21404246	9.65421641
7.29391577	9.22411699	9.37428247	9.39128205	8.54360724	7.66888286	8.88434302
8.83587356	6.76753828	6.87320416	7.02540316	5.65588959	5.90885589	6.1143083
7.11673798	8.52602498	7.80628345	7.70674258	6.58830958	6.89303936	7.01334724
9.68031135	10.3625093	11.6392299	11.4910003	10.3104526	9.83080641	10.363861
8.81110588	10.7047112	10.5009086	10.5095355	9.20645105	9.03477558	9.21488079
6.78223294	7.20737458	7.95188965	8.06640784	6.59656116	6.60611572	6.65524448
6.4789896	7.91601319	7.88278426	7.99507234	6.79588121	6.69575863	7.15111297
5.69165113	7.32387352	7.37285026	7.40221992	5.59861205	5.84544037	6.04864293
9.81252711	12.3697814	11.6223305	11.533558	10.811015	10.454874	10.7977231
8.0652287	9.56677349	9.97123314	10.0086667	7.62248749	8.23651797	8.16513613
9.64887129	12.001932	11.5098863	11.5217648	8.43288719	9.547069	9.57249503
5.49520843	8.21369042	7.73068682	7.77920981	5.2090006	6.51193083	5.63411374
6.48155195	6.84099313	6.94879005	7.09732741	5.60921125	5.93456132	5.92458498
10.61051	12.6260955	12.1199206	12.0270428	11.1598808	10.9692642	12.0588718
8.42897104	9.94725733	10.4239584	10.3236279	9.67201581	9.0174838	9.11476575
11.6341113	13.5342263	13.3271526	13.1481362	12.4162832	11.956142	12.480985
6.26210537	6.77007844	7.0625427	7.24260536	6.49430728	5.74564313	7.33871321
5.70013783	6.64639886	6.69325137	6.71343601	6.07306399	5.60298148	5.89264384
13.9880615	15.1287173	15.4308325	15.3586897	13.7778676	14.1036443	14.1551313
14.0780657	15.3822397	15.1744194	15.1162863	14.3394555	14.0908154	14.3350693
9.81379546	11.2596032	11.308934	11.1693006	8.91436689	9.25753682	9.23990088
6.48096255	7.56187522	7.77585781	7.80881041	6.1105946	6.5552416	6.22994221
7.0660655	9.35704521	8.85583775	8.88393265	7.36421171	7.5723008	7.89947838
6.71260954	8.56699015	7.97845307	7.91947655	7.04103595	6.53142638	6.96388907
7.89576975	10.2549967	8.72688161	8.73377055	8.14010172	8.24029429	8.63915654
6.26950168	7.84646072	7.26354996	7.2308649	5.89307966	6.27454384	6.58721285
11.9298102	14.1207271	13.8060098	13.7266397	12.7939453	12.2665386	12.8924872
9.43329416	11.9799249	11.6509916	11.5489587	10.9997076	10.7525429	10.4966291
9.7649031	10.2139487	11.5320612	11.3956755	9.91226977	9.54421933	8.81045499
7.84006537	9.45060382	9.75296965	9.59150645	8.48454581	8.07504588	8.76311209
7.76046249	9.73098021	9.31852338	9.26129315	7.44620163	7.77024234	8.22605762
7.03240835	8.63161435	8.30430203	8.18830844	7.0840313	7.17843579	7.15330172
7.29865648	8.07175087	9.02329001	8.98478386	7.5319327	7.15151385	7.59889293
8.5159919	10.4379403	10.1415209	10.1754547	9.55123645	9.28796419	9.15707555
7.09360665	8.50148413	8.09985589	8.059816	6.74650492	7.22393412	7.14203045
7.3308649	8.75757702	8.24057288	8.43913232	8.02520048	7.35691937	7.6305853
9.60578284	11.5062565	10.9376291	10.9482241	9.43722566	9.62168231	9.97827573
11.160849	14.0099598	13.5935429	13.5521443	12.0842766	11.9959424	12.4252899
11.4779252	13.3210402	12.8750682	12.8246637	12.1083082	11.6965946	12.1076153
9.26198323	11.3818165	11.0148211	10.8947587	9.41075597	8.86657143	10.1625751
6.03455579	7.05904372	7.18325624	7.13714076	5.59385122	5.89173316	6.45223168
7.304407	8.05979221	8.62298597	8.49211156	7.45743998	6.63254073	8.24092582
11.4383818	11.0724227	11.5500132	11.4803752	9.27190021	10.1614406	10.5161718
7.37734243	8.95541249	9.04656964	9.04842421	8.3165137	7.70578373	8.60221597

7.64139141	10.2517025	9.55005047	9.40602702	8.40739524	8.1218531	8.50730864
6.65809292	6.83827487	7.05090759	6.89666541	5.73476624	5.96730074	5.97226415
8.63509375	8.78281905	9.33890393	9.37852485	7.06167901	7.96069994	7.7889271
8.65223658	10.8505018	10.668026	10.5974623	8.87494509	9.29315917	9.03390969
7.19204125	9.64499547	9.13646032	9.08433937	9.22100123	8.22435193	9.07824289
11.3171576	12.8659323	13.0737158	12.9445443	11.8867982	11.6554963	11.9666259
5.68731269	8.71164185	8.95188149	8.91621179	5.45041222	7.20945434	5.78946609
7.40202716	9.349139	8.55475844	8.66362926	8.13845357	7.52708989	8.06124697
11.6523745	13.2704957	13.785103	13.9326466	11.2879501	11.9988802	11.7785449
12.5095135	14.2498209	14.2127973	14.1402706	12.6335879	12.6093124	12.7942317
8.06207367	9.69568522	8.44093435	8.29568308	6.29369805	7.3051908	7.75202666
6.20090606	7.12622539	7.63394626	7.6453133	6.97920224	6.49447418	6.87130525
6.66837446	9.05638763	8.7917392	8.72458138	7.7599208	7.36664394	7.5633023
5.36336209	7.7531024	7.60539655	7.47417561	5.03855031	6.10931417	5.55000116
10.1816269	12.0607284	11.8015576	11.8534207	9.75090676	10.2008704	9.9693635
10.1991693	12.0188004	11.6289677	11.5304595	10.2640919	10.7066281	10.8959554
9.12310848	12.0632431	11.216998	11.2097391	10.2181672	9.95162874	10.4513811
9.40321105	10.7893519	10.3354417	10.425817	8.82018886	8.99941379	10.1706324
8.94992357	9.91604604	10.3756346	10.2549896	7.91693637	8.36077511	8.41394267
12.4602777	14.4560823	14.4881885	14.414818	12.1337571	12.6744357	12.5253233
6.66137908	9.02666784	7.76081241	7.681769	7.47155654	6.94822471	7.30565047
6.29975312	7.93736794	8.1788187	8.28331991	6.69015586	6.3941303	6.59394904
6.93067466	8.13190582	7.96591412	7.9093657	6.81030942	6.80032659	7.01051916
5.96195688	7.76319045	7.87472259	7.77108863	6.00793012	6.64358133	6.59824103
6.40214453	6.95647838	7.40929928	7.45859937	5.95208308	6.2573043	6.5151911
7.49331749	9.27126715	9.27429545	9.21578924	8.39376454	7.80285236	8.33413426
8.53539825	11.1674332	10.4466346	10.3532549	9.29139566	8.87353147	9.03980134
6.90750481	8.61434103	9.13980483	9.08157052	6.7655122	7.2903175	7.00905487
12.1625443	14.1381998	14.0114972	13.8752934	12.7147369	12.4613779	12.6117752
8.16937838	10.1613473	9.89771267	9.8752098	8.75722364	8.32845952	8.29172096
6.98342347	6.05730513	8.25052582	8.02106714	5.10630853	7.41224126	7.83637611
7.07855753	9.24991436	8.57190582	8.5985822	6.87337767	6.39912939	6.99845955
8.32609372	10.0689099	10.3856467	10.0901932	8.96542847	8.53430094	9.14565912
6.95030977	8.22919086	7.60093724	7.75594376	7.66348197	7.13122008	8.25821786
8.17690416	10.1398097	10.1686746	10.0482989	8.10637449	8.22578729	8.36183677
8.68994313	10.3196439	10.177995	10.1348858	9.57885986	8.7076818	9.25664981
5.89193823	7.10718778	7.15309496	7.17272152	5.8798954	5.89067213	6.52722488
6.54945417	8.90835239	8.31264067	8.192854	7.89196046	7.372024	7.21452895
9.99399413	11.6754843	12.1038526	12.0160227	9.34678629	10.0096369	9.87620013
7.18368599	8.43426417	9.96694086	9.85788461	9.30369948	8.18636932	9.89099365
7.70562202	9.67221882	9.69154638	9.6773428	8.31710957	8.42776329	9.17875198
7.14220306	7.96694778	7.81694652	7.75694722	6.17445815	6.44921568	6.4579946
9.30560602	11.4901815	10.7201861	10.5893188	9.13396623	9.35973814	9.86565425
9.37946241	11.6621249	10.6615448	10.6469493	10.1486177	9.98382018	10.1676991
8.00550273	9.30116281	9.77713546	9.7782738	8.65194825	8.71863014	8.78653331
6.95083464	8.70506487	8.11921298	8.05975004	6.4041463	6.62338742	6.98793134
6.46455964	6.54601067	7.24979232	7.17595966	5.9325195	5.97388652	6.00474002
7.9630764	9.56275567	9.08716048	9.06896445	7.34085954	7.44771969	8.17246101

8.62914749	9.43094152	9.35175464	9.27916227	7.93871216	8.48284748	8.23922713
9.76119301	11.7360805	12.0604214	12.1194739	11.5732013	10.8351914	11.2165003
6.59190721	7.04991932	6.28765587	6.2662712	5.97708412	5.73238155	6.33550291
9.73242999	11.3006952	11.3956241	11.3329932	10.5010062	9.56017193	10.2192545
7.81986358	9.72156696	9.00166124	9.12651606	8.72870012	8.05372337	8.82014162
6.62659523	9.13607601	8.89850798	8.90608276	8.20906925	7.49454682	7.56246606
8.19437717	10.1333131	9.39497044	9.37373564	7.55496713	7.8389253	8.17336633
5.79144684	6.95918475	7.22232544	7.29920619	6.02681555	6.08884377	6.34436764
8.95232862	10.4340089	10.3563981	10.2123201	9.14591075	8.86943407	9.54563792
5.91761459	6.83814122	7.1106994	7.03174806	5.91697935	5.97524887	9.20118908
8.30989966	10.490168	9.97325538	10.0038749	8.24967714	8.65540693	8.4943348
6.98689213	7.70881904	8.55060251	8.49172205	6.53757392	6.60289881	7.01002451
6.49064634	7.62910668	8.11107358	8.11271108	5.98813795	5.84055085	7.06507895
6.30462256	8.4397856	8.49669602	8.34227161	8.23601276	7.33884422	7.65821791
10.6104085	12.4481042	14.0567342	13.9113424	12.2304867	11.1181384	11.5766733
7.202548	9.02703404	9.45623304	9.34525709	7.99374851	7.71747697	7.40970405
10.7992355	13.6504033	13.3472075	13.306741	11.3126051	11.5027269	11.6050118
5.6742872	7.3294313	7.47761176	7.41968299	5.25358922	6.11563653	5.47671543
8.20767034	10.2676997	9.8447577	9.70967094	9.09006689	8.49461802	9.30529433
12.6143751	14.8698497	14.6877382	14.6947643	13.8730076	13.0981009	13.4884327
7.76140097	9.07736933	9.39216821	9.34232695	7.5016342	7.89297321	8.93363489
12.2259497	13.8347713	14.1611579	14.1754521	11.5561923	12.3236971	12.082187
8.46969635	10.9426553	10.6007009	10.5703956	10.2855594	9.16701313	9.60438069
10.9773329	12.8740884	13.330627	13.2730406	12.2653528	11.8244512	11.2648021
8.41857874	10.873551	11.2223379	11.1362542	9.94312225	9.18041817	10.045174
8.87415144	10.5858414	10.4408007	10.4583984	8.89255769	8.74989691	9.0520194
6.71623095	8.95283354	9.28832626	9.32259541	7.09809632	6.98888574	8.34048082
9.78092914	11.2722402	11.0166036	10.9870032	10.1025309	9.65287419	10.020123
5.99291342	6.46158471	6.08635791	6.01427517	5.47557554	5.76860119	5.80252748
11.6681635	12.9806191	14.0897724	13.9742963	12.8531994	12.8060378	11.761884
6.38027525	7.65649208	8.18551376	8.18263917	6.68657896	6.69475904	6.80715267
6.88361715	7.47695353	7.12742074	7.12481922	5.96062847	6.56742726	6.28136554
10.8802937	13.0866743	12.7736544	12.7065811	11.7975825	11.5476339	11.4096938
6.94711908	8.98451046	8.41604211	8.45194299	7.08856354	7.26031082	7.53494791
9.22764217	11.2803793	11.2087824	11.202073	10.1968541	9.56412433	9.69317713
9.46021444	10.2743479	10.5533	10.4756149	9.23466322	8.91834389	9.12283084
6.56935194	7.75844843	7.78244264	7.73974889	7.37666546	6.92879667	6.99532509
9.05359903	11.1388017	10.7279689	10.7803142	9.76817506	9.67756339	9.18292391
6.81683263	8.27659297	8.31927967	8.23554087	7.05227024	7.02652566	7.08070243
12.3391031	14.2908216	14.3178747	14.2589119	12.2624989	12.8339106	12.5341168
9.34477037	10.3484958	11.0573887	10.9812269	8.84765373	8.86226757	8.90279867
5.85081067	7.86425054	7.36098751	7.40942064	5.84440976	6.04621536	6.10012994
7.11499578	10.2485028	9.57521683	9.63310203	8.10510027	7.56555605	7.9470273
7.04974837	8.08283562	8.18134908	8.15216364	6.45930817	7.16994905	6.76680185
11.4450526	14.4180002	13.9237267	13.8374634	12.8052392	12.283543	12.9578811
6.42657054	8.15943846	8.93438794	8.89027249	7.25942974	6.81273309	7.78357648
8.23383295	9.74326755	10.1609404	10.1871751	7.80183573	8.20413729	8.19605962
8.09009918	9.79255395	9.14210323	9.03217194	8.11015598	8.00461543	8.14660431

7.27108293	8.58632453	8.76474125	8.83337526	6.70136192	7.34587624	7.31703448
6.33427221	8.14534731	7.55925242	7.55275362	6.19469186	6.63997836	7.04949355
6.36928919	7.11728881	7.65586126	7.37747084	6.01177851	6.08581043	6.20376281
7.20050141	7.72715808	8.08070776	8.08892071	7.45148231	7.23388039	7.37735718
10.1372742	11.5382767	12.1490005	12.1627891	9.6644256	10.4344291	10.166718
8.28286001	10.1834605	9.66726769	9.73305855	9.28101336	8.82612992	9.00757844
11.5227095	13.346342	13.1073569	13.1609441	10.9331359	11.4991498	11.1644218
8.16580293	9.65819345	9.42671783	9.53671211	7.8948111	8.09461718	8.4007215
7.33839143	8.04249989	8.02971514	8.10426222	7.05803287	6.82618237	6.56161559
8.42457531	9.25495183	9.60748345	9.69425271	8.551927	8.06047276	9.51818457
7.47237567	9.36999313	9.28140314	9.36089996	7.36866796	7.82215397	8.28468578
11.0992314	13.1900602	12.9610539	12.8756277	10.742653	11.3205524	10.8952011
6.17975188	6.73291903	7.17055104	7.32337097	6.53795839	5.97601965	6.6330625
13.1795527	14.9591602	15.0397219	14.9600169	12.825537	13.3876781	13.2451939
7.29712629	8.0529826	8.13612011	8.04728863	7.06400439	6.88495074	7.59621809
9.34996874	11.0682986	10.7786587	10.7936984	8.70109676	9.276452	9.13741361
8.80817007	11.0687481	10.2647182	10.2257762	8.94180002	8.87466942	8.79758631
6.15101268	7.25922823	7.18316491	7.13531836	6.0151552	6.50073068	6.65707688
8.9447715	10.6852709	10.7013351	10.5498896	7.81554341	9.07011075	8.79573643
6.01337963	6.53357114	6.83683431	6.99299837	5.73441536	6.05310786	6.042594
7.52030516	8.82541518	9.04866805	8.95643134	7.00825493	7.61959935	7.5027093
5.76774165	6.68638287	6.62876106	6.67713751	5.369545	5.5826704	5.95075277
6.17049455	6.92919093	7.14885684	7.13430207	5.78541166	5.77344071	6.01151487
6.4937911	7.34950152	7.32147538	7.31989697	6.00307665	6.58381961	6.28696984
6.25493828	7.74749952	7.78127118	7.78911235	6.10811083	6.59143932	6.47881928
5.63051643	6.15206333	6.32800561	6.34009293	5.34452282	5.52007583	5.7070424
6.07817729	7.37396657	7.73209211	7.60200249	6.19045466	6.23643388	6.59654535
7.53134481	9.22351354	9.03864716	8.93591405	7.31254447	7.96045495	7.48889414
6.47812308	7.49774967	7.53333596	7.54014199	5.73490088	6.17009271	6.08849654
7.382324	8.88054105	8.72020971	8.71954284	6.82722836	6.97643811	6.9291495
5.95094869	6.51954112	6.75105795	6.67147738	5.27737296	5.61270753	5.65229116
6.04538181	6.83368834	6.86207195	6.76762037	5.69535052	5.94082098	6.08648721
6.36208865	8.1042014	8.01930105	7.97130155	6.49458298	6.53200254	7.17504449
5.81209437	6.67898035	6.67261769	6.57831608	5.4234306	5.57155426	5.81241351
6.94332594	9.39161415	8.98794043	9.14419885	7.60613719	8.00394061	7.60574099
6.59757572	8.70643662	8.31052372	8.24339013	5.72737757	6.44675315	6.22533606
7.26433469	8.50655593	8.74839045	8.77990579	6.83280649	7.39258704	7.19376386
6.55811745	8.05399645	8.29002207	8.14351485	5.96651053	6.57293543	6.50415907
5.77695724	6.47754555	6.79047231	6.61735976	5.59261371	5.70974444	6.09365207
7.51370154	9.83131548	8.76761408	8.93990033	8.41248178	7.88622089	8.01563026
6.89912245	8.15979214	8.74889021	8.70851559	7.02010767	6.75244488	6.70179716
6.67037583	8.49903305	8.27243337	8.31498241	7.00852611	7.28033743	7.15868744
6.91775038	7.58815544	8.44200212	8.44550532	6.89083334	6.89121016	6.96068777
6.35547796	7.88597696	7.60257714	7.59619199	6.00246288	6.35046648	6.27939255
5.58508693	6.4239882	6.13768344	6.27266431	5.28884652	5.33452323	5.7071871
6.86511586	7.88378697	8.00824933	7.74979838	6.19940596	6.56857925	6.51777472
5.61801718	6.25671688	6.61071733	6.49928368	5.41348678	5.83089873	5.42953429
6.67117871	8.55278897	8.25581322	8.30943361	7.44913379	7.53546591	7.53140798

6.08797088	6.99414409	7.51971566	7.52831892	5.37421843	5.87840386	6.03011688
6.44790991	7.83104053	8.17412624	7.96473247	6.10512175	6.43659995	6.45867368
6.47646626	7.21914335	7.29978223	7.22217085	6.7375367	6.16283923	6.20400706
6.34148324	7.02477843	7.32895222	7.22784117	5.50432451	5.93078393	6.13044207
6.61875255	8.14578188	7.64120208	7.54944816	6.47626681	7.04637974	7.39449442
6.84036504	7.88380285	8.65804971	8.52742383	7.66562869	7.29322608	7.09127669
8.29153219	9.474004	10.2136948	10.1387927	7.70677849	8.10197305	7.99015673
7.38512987	9.27444502	8.61520992	8.60764804	7.94320461	7.73492161	7.85053254
5.80051375	6.54966301	6.56053338	6.63049022	5.48068262	5.71093396	5.97103835
7.17920747	8.65173699	9.79681528	9.76272452	5.72726156	6.7000124	6.98227034
6.20223708	7.24580747	7.77477191	7.70328208	5.75813605	6.21170825	6.3560783
6.34580405	8.58560256	8.22408708	8.17213844	6.57457582	7.37614884	7.38198672
8.18853049	9.62604501	9.93703154	9.84352618	8.14018048	8.52550084	8.79311783
6.41473123	7.48912222	7.66123939	7.63591978	5.75330689	6.14715546	6.14511555
6.51099356	8.57313742	8.32055688	8.31430136	6.45266429	7.18934226	7.26606033
7.05036021	7.72704641	8.61372354	8.57739257	7.32201826	7.11889021	6.75777401
5.94978657	7.50279521	7.24532832	7.31191267	6.43124903	6.27260683	6.3342504
6.55962977	8.12763999	8.43001591	8.26421316	6.50651705	7.22641508	6.80349958
5.99084234	7.28838164	7.0641025	6.93534423	5.72556803	6.04927114	6.03872786
5.57016932	6.10727735	6.40104043	6.17634811	5.21283194	5.4409884	5.44714904
5.63818711	6.36274322	6.49269346	6.43317179	5.41402354	5.50447103	5.67881448
6.29465806	7.9771637	7.86829414	7.9402901	6.95768365	6.82215015	6.88299146
5.58455969	6.64685964	6.41531874	6.33054549	5.29137259	5.41854282	5.60011956
6.15681322	7.79247928	7.5531631	7.52956408	5.67801527	6.5674662	6.47243594
5.6808183	6.49634186	6.57620285	6.44533903	5.68203752	5.64965512	5.67947813
5.49806477	7.41297963	6.97270529	6.70106244	5.29643171	5.58512329	6.46994994
6.78862307	8.9608476	8.30501838	8.16229565	6.78658852	6.91874042	7.595488
7.67340731	8.23498312	8.32213862	8.34604251	5.73222903	6.67534737	6.71731999
5.99314967	6.71336864	6.90146549	6.72163378	5.66977741	5.94787254	5.8747146
6.9497742	8.56606317	8.65680953	8.49152044	6.55963895	7.03478356	7.50269101
6.58600853	8.47373253	8.51762294	8.42939712	6.62150701	7.11092277	7.20485574
6.17729295	7.21002935	6.86668918	6.87960163	6.51002276	6.07503582	6.49548539
7.82123825	10.5892289	9.91079576	9.71067679	8.66699264	8.53852885	8.64855784
6.13005095	7.70902728	7.83771938	7.73079889	6.3406908	6.76751974	6.11243575
6.79529811	7.93116181	8.13608011	7.98805976	5.94390797	6.83686586	6.82944622
6.44305708	7.43183103	7.64960253	7.52175981	6.06018577	6.20270901	6.14969165
6.24430484	7.23711721	7.97372205	7.83320952	6.11876711	6.5167026	6.53960675
8.66887825	9.93336682	10.4816673	10.4320982	7.81891957	8.25960581	8.57896437
6.71659863	8.13450487	8.61314485	8.57881043	6.53711867	6.98125445	7.12948189
6.16426516	8.57169994	9.29210003	9.14972096	7.04820239	7.56022582	7.00920943
5.64181969	6.56487626	6.69783497	6.754798	5.80885223	5.82493304	5.86734596
6.71466315	10.0274914	9.57710966	9.58691464	6.30398855	7.3315234	6.76509842
11.9081967	13.629936	13.2531192	13.1157343	12.8692281	12.302706	12.3042144

C069_NS	C129_NS	C184_NS	P011_NS	C147_NS	C174_NS	C182_NS
12.9578457	13.78137	13.4527612	13.3881922	13.4081665	13.7249977	13.7914031
6.98056918	8.25633703	7.44378565	7.35967929	7.36026506	6.71089871	7.1586935
6.7931858	8.01637709	7.4073842	7.40251328	6.85686745	6.92460707	7.2797907
6.44890525	7.83956599	6.99493945	6.5806145	7.10891644	5.91872993	6.42297603
7.20968242	7.53293484	7.05970354	6.55180299	6.41900857	6.61890419	6.68362769
6.75976304	7.99859833	7.36745232	6.96121352	7.45675277	7.22919567	7.11684921
6.23685076	7.44633399	6.87117709	6.6407233	6.60581343	6.82521576	6.97863496
5.57202258	6.44136246	6.15189372	5.56130872	5.89481073	5.86645255	5.71386295
7.02797675	8.26980695	7.27418524	6.66947045	6.7918987	7.26720149	6.94115093
6.00305438	6.91469845	6.49869239	6.37078905	5.99175107	6.44576312	6.83060267
6.55553458	7.90430285	7.00897646	7.25400628	7.12626773	6.80318441	6.61837699
7.75025142	8.84806265	8.44823486	7.97598034	7.81988976	7.64099827	7.67014281
6.40925515	7.44034181	7.36754842	7.18979294	6.95830446	6.25223322	6.22445998
6.70716446	7.79885928	7.38211127	7.78060735	7.1384039	6.92258559	7.16282835
6.63071284	7.27037102	6.9915774	6.4828728	7.42339413	6.06921	6.06431854
7.35111415	7.73603859	7.90779861	7.4430405	7.16110467	7.12948086	7.10370639
7.10108379	8.23062862	7.28098043	7.21783753	7.22161612	7.10155133	7.97440484
7.0358713	8.09678683	7.58000299	8.05280115	7.4414204	7.24713666	7.70894385
5.3644215	5.76155363	5.73436171	5.45615068	5.58533154	5.27899725	5.35628187
5.92011653	6.7382747	6.46569308	6.57376336	6.10948259	6.00078065	6.14834743
8.3169725	9.28615967	8.52586138	8.92847692	8.10920748	8.11908326	7.79585589
5.11459024	6.06241555	5.73550214	5.41317296	5.62950409	5.07575509	5.25239409
7.28502855	8.24777374	7.73772326	7.88290711	8.15274623	7.42584866	7.57457082
11.4435611	12.7358636	11.9647405	11.8403452	11.9029641	10.7688551	11.3643071
11.5045603	12.4799285	12.462754	12.3437132	11.7589219	12.5596383	12.3081041
5.70575291	6.61044258	6.19354448	5.58693734	5.59560689	5.68750222	5.57719858
14.1566678	14.4304107	14.3809304	14.3450254	14.2672132	14.072115	13.9566756
7.85248082	8.78311906	8.13136363	7.94347305	8.37274929	7.21060609	7.55776083
6.38019073	7.8378764	7.23549253	6.64655007	6.91727898	7.07652489	6.86003786
9.39474756	10.4298576	10.3013887	8.67782146	9.20971995	9.29523331	9.12622642
13.4900712	14.6193339	14.2593387	14.3436021	13.9048801	13.7518181	13.6671781
12.2470917	13.4206932	12.7290255	12.8474806	12.701642	12.4877546	12.3168113
5.73084208	6.4735439	6.32229972	6.11874095	5.90239803	5.60343704	5.50114102
7.75617133	8.60342346	8.12242476	8.07824529	7.84631466	8.11865562	8.37112015
5.1077021	5.64543219	5.45797671	5.14497591	5.195448	4.93670719	5.22586449
11.3259036	13.3021062	12.4594647	12.2491078	12.4572193	11.5313371	11.1478924
8.82447002	9.66380624	9.11220855	9.19249355	9.07808648	9.77752673	10.1744027
8.57700464	9.36331785	8.83013266	8.99813259	8.82275213	8.81568814	8.9892713
5.35686322	6.53553636	6.43346593	6.1161622	5.92073919	5.78302127	5.89449476
6.40254688	7.20248727	6.76588344	6.60423667	6.64382012	7.14978776	7.41891853
8.87626921	10.1119064	9.91898857	9.00849193	9.25131529	8.70389492	8.75120695
7.49264636	8.69429067	8.48272407	8.25527648	7.9421356	7.55709451	7.60475113
6.58566054	6.83863416	6.74225149	6.75378101	6.75141819	6.12741888	6.71150179
7.43256779	8.39449742	7.35689209	7.61802203	7.05983114	7.43398763	7.30768079
5.43889296	6.82652561	6.52151501	5.92389554	6.02309675	5.95957196	5.88278682
8.35766944	9.76515115	9.31144113	9.10610147	8.84375087	7.97450866	7.85056317
6.6027146	7.61570982	7.10763557	6.80288208	6.98198162	6.0456675	6.70435188

6.81969884	7.77215091	7.17163218	7.56197065	7.07402369	7.04089134	7.40642187
6.2837605	7.26617748	6.75388528	6.90273687	7.25882322	6.42666228	6.27330906
9.87364082	10.8564185	9.99949976	9.81936961	10.3209912	9.24045554	9.8316594
10.5693069	11.6806795	11.1211547	10.7908445	10.6449495	10.1036419	9.80157516
6.87910411	7.59763843	7.02290928	7.60584098	6.94466522	6.42286539	6.71552551
6.53954425	6.97021175	6.63572828	6.62856773	6.68688986	6.23144143	6.37680591
14.0471418	14.9768013	14.7141677	14.6654397	14.3971478	14.1880858	14.0299273
6.0638103	8.64582701	7.69587901	7.50828444	7.5062195	6.87637083	7.02278059
5.93878496	6.54260135	6.37948009	6.31963564	5.74187574	6.17487317	5.82041718
8.65169179	9.73779288	9.27400348	8.82430007	8.63328044	8.65947186	9.22730668
5.35355408	6.23821555	5.8224862	5.52203	5.68223421	5.29664792	5.66616093
7.0743074	8.54988976	8.13841261	8.04270343	7.61267834	7.09986746	6.85974846
8.60968221	10.5925234	10.0131286	9.35845758	9.9340094	8.82613755	8.57894022
6.95523491	7.8323527	7.62367961	7.68672252	7.21421905	6.9150667	6.67311541
6.60621242	7.99193561	7.60060725	7.61953406	7.08826671	6.7240977	6.98945922
8.55032469	9.77362552	9.0974059	8.69844388	9.27673928	9.30640603	9.10574512
6.32279273	7.4682982	7.24312923	6.51770592	7.07018536	6.94737628	6.87093429
5.57679269	6.74909979	6.45203198	5.86171473	5.56525504	5.83339588	5.73142032
6.79841737	8.20792798	7.5487256	7.5850181	7.20118785	6.6894697	6.58658327
9.51058202	10.0447589	9.85834815	9.64886931	9.53498059	9.42869809	8.97929257
6.25182739	7.36813363	6.46973411	6.00810161	6.21942373	6.06058008	6.0716788
11.2849027	12.3636042	12.1107355	11.5652868	11.5576623	11.3011143	11.0384094
7.91435643	8.85833945	8.27580372	8.15097847	7.99001998	8.27831796	8.19999961
7.74297092	8.7508664	8.0963035	8.14565606	8.04943691	7.27811767	7.73546464
8.10934384	9.720786	8.67423535	8.54019044	8.27701906	8.19856389	8.07628117
8.71191127	9.6398381	9.50933896	9.15242201	8.72013753	8.55188549	8.21688309
6.82504145	8.02646369	8.0311702	7.09571876	7.13994011	7.03382272	6.95051274
8.10169314	9.03858077	8.56148118	8.37622091	8.5832227	7.7581066	7.71313559
8.68101769	10.0279627	9.56626948	9.23618955	9.173846	9.17520371	8.59715347
5.47113542	6.74067801	7.51175107	6.40676556	5.82801078	6.62296299	7.12044733
7.50930313	8.19273419	7.78249899	7.8061829	7.54815607	7.27440611	7.67257668
7.87820299	8.9664371	8.37546609	8.36615258	8.47503542	7.8085646	7.98599047
8.36906597	10.0146454	8.97663744	8.64763111	8.96736836	8.56598971	8.68381153
6.6675473	6.84260303	7.10504316	6.81339386	7.23974458	6.56363514	6.32025378
7.74599291	8.85223642	8.34036198	8.15146412	7.64643559	7.80940222	8.42888313
5.43677816	6.04984332	6.47949897	6.02075464	5.81135928	5.68003314	5.43211024
6.88485674	8.10431045	7.20925352	6.47918011	6.80375529	6.97383166	6.82445856
7.27494464	8.42886806	8.29941594	8.52338122	7.39956676	7.18755597	7.4898279
12.6721701	14.053012	13.4435704	13.3229112	13.3916692	12.4012907	12.199868
9.32072723	10.4536482	9.94407116	9.56180745	9.42409153	9.24402745	9.37167756
7.61752624	8.68605154	8.5509399	8.11877344	7.84322513	7.44394143	6.90944791
8.04194821	9.59954338	8.92906278	8.67828146	8.63595713	7.96386711	7.65706902
6.46695366	7.35273999	6.84815595	6.46768363	6.69335453	6.28328953	6.10574805
8.67149047	9.63421262	9.14904948	9.8000494	9.60972837	8.741619	8.52213777
7.7073543	8.82318856	8.12866201	7.87596186	7.77736316	8.27552952	8.09419172
5.74938785	7.07809315	6.45634669	6.58961909	6.55719578	6.04141779	5.71057035
7.39751378	9.14946084	8.47004303	7.40046193	8.37425964	7.95515701	7.08682303
5.70970667	6.87910411	6.25382121	5.78265546	5.85772626	5.6333414	5.18003604

7.93923422	9.12523574	8.85179026	8.51274639	8.33039123	8.09429625	7.16790181
7.85761281	9.6531333	8.97226731	8.24837639	8.72690511	8.48924186	8.32982145
7.60743611	8.34074829	7.64387999	8.35064089	7.79081699	7.01404391	7.58381014
8.59337768	9.57670428	8.89064155	8.72817665	8.30401677	7.59423726	7.86301913
11.3480476	12.7058302	12.1633978	12.3182742	12.1095591	11.3118998	11.0955124
5.82453977	6.35732591	6.21858435	6.4562279	6.30266524	6.20915758	6.14450183
8.24832515	9.16514292	8.57162031	8.51394112	8.0501719	8.73598851	8.83860769
5.71504771	7.17648335	6.19866761	6.2477335	5.98460895	5.87612202	5.91246281
7.54495285	8.22475081	7.78458512	7.19485533	8.77931554	7.62478376	7.404256
10.9710348	11.540764	11.496722	11.9941105	11.5408671	11.4865676	11.0613935
6.94132585	8.70289991	8.18300408	7.47871947	7.5467363	7.18376141	6.95846456
10.3238418	11.312917	10.8053408	10.2353202	10.1001078	10.9153446	10.9750512
10.0524159	11.8997848	10.9519935	10.3637964	10.4972162	10.2407472	10.5455157
5.99243627	6.71899994	6.48320472	6.57744046	6.26777024	6.17632756	6.35250184
6.80411682	8.27914021	7.36713601	7.49865936	7.61440287	6.19145428	5.95732705
8.89271857	9.75841612	9.12850478	9.5815983	9.06915516	9.07339423	9.18091216
5.36136097	6.45363215	5.90622897	5.8100775	5.91155809	5.50508268	5.9997439
12.1822683	13.5515881	13.0416472	12.9502601	12.4165875	12.5757111	12.3145728
5.51913645	5.9524256	5.76661636	7.24967667	6.28959012	5.53988649	5.34016736
9.82175135	11.2293163	11.6317259	10.9444844	12.8709272	10.1565749	10.4003234
9.94326	11.0718049	10.5461951	10.7830359	10.3095389	10.4785563	10.7640298
10.8227341	12.1911231	11.3370158	11.2834305	11.3689336	10.0961722	10.7928692
6.70232509	7.55070601	7.40707313	6.82815299	6.79254305	6.68868533	6.57181922
7.47180119	8.70124026	7.99160347	7.27281078	7.32699118	7.06347598	7.39031343
6.20935549	7.51802756	6.97216073	6.73676315	6.36743467	5.91639567	6.29143025
10.1671321	11.6273004	10.7569616	10.3108059	10.4185958	10.1119624	9.83212037
9.89342122	11.4495036	11.1277474	10.6278813	10.6499163	10.4209308	10.2302907
7.58158851	8.45827208	8.089326	7.82832947	7.58300151	7.90187212	8.04601187
6.60497525	7.96067561	7.40633683	7.19994417	7.28338288	6.84890832	7.0005871
10.4037705	11.9735765	11.1377411	10.8435756	10.8678818	10.2837933	10.174862
6.03131645	6.72364866	6.57058588	6.48567413	6.44428027	6.02930689	5.98529959
7.30452747	8.18976725	7.9992001	7.23035194	7.19641328	7.5342593	7.19743886
9.9216623	11.8983584	11.0468655	10.8735118	11.1334556	11.0684095	10.7849006
7.02294921	7.56677483	7.18000858	6.72941682	7.14271159	7.12933248	6.84812589
6.47622014	7.94582129	7.20918137	7.27349071	7.03610766	6.50635376	7.17160717
11.7422795	12.71439	12.1357074	12.1769071	12.1345483	12.7248236	12.9222876
6.43382777	8.27920197	8.5008825	8.82662793	8.08555425	6.62846975	7.92455897
6.89936659	8.30752598	7.54490186	7.50594967	7.69633121	6.9567374	6.97624218
10.9197066	12.615651	12.031216	11.7116807	11.7464782	11.5723251	11.3157168
6.7135776	7.56725718	7.1251324	6.77991236	7.08576243	6.57673481	6.94502706
6.52475091	7.45076669	7.03417499	6.39960623	6.82238218	6.38039284	6.69402073
6.38051613	7.25153543	7.02591691	6.75042294	6.91343399	6.77883936	6.54547143
10.9184764	11.2282692	11.0276545	10.6788175	10.3155572	10.3798614	10.3720412
13.5936736	14.2799674	14.2487089	14.4639317	14.1815941	13.6481935	13.5137917
7.58526453	8.5293837	7.96053372	8.2619783	7.75223746	8.30388803	8.41064554
9.67587343	11.1804301	10.2330756	10.336781	10.8191261	9.5365435	10.4354723
6.38155333	7.61903365	6.75890808	7.14580175	6.49871648	6.49844204	6.21727144
6.41301586	7.11908222	6.81897068	6.62590723	6.42627784	6.18636367	6.31638545

6.27111805	6.52276779	6.4615595	6.20904467	6.31250418	6.43971322	6.31755263
8.31033921	9.22426317	8.79894479	8.50052523	8.55221182	8.94571743	9.29561261
11.7822288	12.19639	12.0885279	11.0977839	10.9588999	10.5915887	10.7734013
13.3704667	14.0175122	13.7711717	14.0969984	13.7893034	13.3722815	13.7144173
8.73291597	9.40486503	8.77838273	8.74716448	8.58220334	8.45140854	8.38417504
7.29184756	8.51368339	7.56499161	7.142152	7.02559916	6.97150332	6.8770129
5.78954486	6.87455846	6.77667779	6.82943988	5.7074508	6.95563364	6.15971798
11.087193	12.5490822	11.5250779	12.0408541	11.4828957	11.5554179	12.0117916
9.7735164	10.6219246	10.3165418	10.1504416	9.68441341	9.77923969	10.15058
6.16253413	6.65666082	6.63966036	6.24845988	6.33897023	6.22655516	6.5474398
10.6473181	11.7135631	11.1179132	11.2957024	11.0992748	10.8071424	11.1726169
7.43798588	8.33284038	8.25253251	8.38339067	8.11294456	7.62445699	7.4576739
8.18718577	9.42388339	8.82296145	8.66201176	8.46539458	9.17698916	9.39356357
5.42637782	5.98395125	5.76927586	5.66059211	5.49763887	5.4523111	5.46397975
14.0662347	14.8963031	14.6725082	14.6993838	14.3282058	14.2634181	14.2072599
6.4710816	7.66564645	7.00429127	6.50710579	6.84524916	7.20836021	7.1636044
6.98063429	7.90881051	7.14986497	6.83821814	6.99856899	7.15046524	7.38079351
6.15022841	7.62142554	7.16914333	6.6165484	6.94383344	6.37324607	6.0740042
9.01810446	10.4787009	9.63462049	9.59385309	9.49519387	9.33089291	9.08630955
5.50399392	6.19766472	5.98223565	5.39793504	5.63678556	5.58801838	5.61238936
10.3205795	12.0734665	11.3240776	11.1223667	11.0701082	10.4969955	10.2230544
8.06265764	9.09293232	8.52060218	8.4094741	8.5125951	8.16957881	8.33804194
7.35024086	8.18406255	7.81095523	7.26223582	7.24243674	6.68216594	6.71445023
6.17694424	6.88534496	6.54179638	6.55052286	6.38090482	6.3076522	6.66656914
5.38554217	7.16640414	6.80958165	7.6118636	6.35224475	6.59238064	6.23860995
7.20503273	8.12202193	7.68340031	7.84308879	6.85303503	7.33004742	7.27760357
13.5705009	14.7516654	14.4344588	14.327399	14.0497371	13.678895	13.7999362
5.94941821	6.72716488	6.14916708	5.4094405	5.45367001	5.51947617	5.50011782
6.52722614	7.128645	6.7656448	6.91074825	6.73947685	6.85741251	7.09290933
5.89608872	6.80628925	6.51588462	6.37083542	6.08700959	5.85527166	5.56862956
12.4042744	13.0720896	12.5603957	12.7679825	12.6602854	13.0814373	13.3428275
7.58514283	8.65025979	8.31948156	7.4679617	7.83660649	7.64451535	8.06224424
6.88061084	8.24959324	7.08947802	7.52558032	7.69958286	6.78324841	7.08209474
5.86731977	6.83444956	6.46247152	6.21540583	6.15490092	5.88239899	6.10641281
5.58983268	6.55607268	5.93622097	5.84544614	5.70713519	5.86537424	5.66956739
10.1288708	11.6449013	10.9958526	10.6438192	10.8959812	10.4991722	10.9703648
8.24406837	8.28826442	9.35086495	8.36875002	8.6341104	8.0356624	7.90932309
9.2328056	10.4993785	10.2048523	9.57038988	9.62554046	9.03334288	8.93129421
8.92592141	10.2065932	9.55726648	9.51498048	9.57009268	9.51315119	9.60623178
5.52860945	6.0576385	6.11571705	5.51790398	5.72607426	5.69893881	5.76735447
5.70129121	7.05435035	6.56331908	6.39940953	6.22705734	6.0726122	6.18213072
6.65372788	8.27980927	7.98283105	7.91676504	8.28464183	7.30304095	6.25270195
9.27836768	10.3503271	9.79851959	9.36992009	9.0192713	8.7691641	8.60968886
6.58544662	7.56916219	7.46057467	7.15634302	6.9748287	6.20582941	6.43767368
8.66606845	9.82713641	9.43845858	9.11437301	9.11907963	7.6963799	7.84665879
8.47210024	9.54511912	9.01285133	8.54021989	8.59393747	8.2168923	7.79855151
7.04969611	7.96672635	7.92268013	8.13918449	7.15417913	7.59260205	7.24102024
5.64470828	6.04694521	5.92216502	5.78289314	5.65419205	5.49290748	5.57591555

6.88132201	7.54865548	7.34888038	7.25643422	7.08506768	6.71901638	6.59222439
11.3554519	13.0100973	12.4537429	11.777843	12.3228208	11.4765381	11.5666568
9.8447759	10.9788241	10.2886262	10.3351715	10.3481395	10.8720654	11.2123249
13.383819	14.3019441	13.9135916	14.1650546	13.8309578	13.6367141	13.6190301
11.5294389	12.7844312	12.2914631	12.3323295	12.1422242	12.1457682	12.1714617
10.038443	11.2238473	10.9185346	11.2002654	11.0293224	9.36868564	8.84718673
11.6627565	12.7083638	11.9480069	12.0606446	11.9363781	11.3692151	11.5072283
6.57952262	7.51567225	7.32826921	6.89120286	7.12619544	6.22427301	6.5199169
6.90395533	7.36244709	7.11838361	7.1420376	6.83671062	6.52304736	6.54434149
7.89773355	8.18634753	8.81591416	8.4900803	7.22483617	7.81898154	8.71593111
8.37946675	9.28080835	8.83922426	8.94992269	8.54551089	8.83390397	8.8840154
12.3270218	13.3475895	12.6766872	12.7638925	12.8211041	12.1527529	12.3639811
9.02536523	9.94523649	9.37493644	9.75570914	9.35092968	9.71837875	9.95725327
6.00405099	6.44459452	6.22017941	6.47941829	5.67276504	5.70186922	6.20146626
7.35092062	8.2452784	7.7172055	7.90250174	7.15127739	7.30418611	7.72194951
6.06709851	7.17303846	6.82635654	6.54033095	6.87605153	5.94714176	5.7991513
12.7451793	13.3251227	12.7795731	12.8185834	12.8575112	12.4318313	12.6814596
5.55832642	6.59183602	6.41682049	5.75124839	5.63325014	5.31714666	5.69583879
7.83899713	8.44600101	8.1809395	7.79516302	8.04368845	7.22059695	6.97821165
8.21605368	8.86507656	8.70350289	8.18664317	8.39334197	7.76745022	8.30467029
6.11103528	6.85112436	6.35207327	6.4736676	5.84220047	6.05241089	6.21765435
7.13116348	6.19886382	6.17167641	5.93328511	5.86691045	5.90334635	5.67664259
7.24172103	8.71280554	8.03173042	7.98566379	8.19046227	6.69834629	6.46656986
6.31970806	7.68937046	6.93204345	6.43933407	6.5351654	6.59019659	6.6621414
6.79648614	7.65640341	7.05113279	6.78840905	6.54767639	6.54379312	6.77762618
9.19672924	11.4724441	9.89633907	9.14878771	10.6464795	10.2305921	10.4575738
9.6136486	10.8013721	10.0336377	9.81289494	9.65810274	9.93216868	10.0573054
9.57552185	10.5833649	10.0570156	10.572919	9.8527684	9.85251203	10.4040803
7.67234118	9.19869869	8.71555709	8.19762708	8.4901461	7.89034394	7.54618991
7.73334688	8.94577038	8.36858896	8.17937072	8.0885699	7.47551944	7.27576136
10.9678394	12.2861407	11.8799818	11.386423	11.7466369	10.6129054	10.2950089
5.74638318	6.66932446	6.23248904	6.63796389	5.89896554	5.81391455	5.94425642
9.31939507	9.92797821	9.21844288	9.27167195	9.36847721	9.23183342	9.40936526
7.46926573	9.23999751	8.59572966	7.68523096	8.18486295	7.45845583	7.76335447
6.6088289	8.0006164	6.94165684	7.41888313	6.90959792	6.24187736	6.46840769
6.24430655	6.96491263	6.65121533	6.12438956	6.08584739	6.10494087	5.93913464
8.25555644	9.30500013	9.25751655	9.17440917	8.50565716	8.51951596	8.55032892
9.98302636	11.522067	10.8802179	10.4469944	10.6074025	10.3330547	10.5414693
14.000574	14.6013762	14.5425622	14.5705875	14.4720822	13.7682154	13.7496704
10.3035057	12.0404808	10.9449177	10.6956285	10.8249245	9.76577606	9.77867939
6.24529637	6.69969737	6.73451725	6.41047069	6.30973517	6.34461193	6.34563269
8.95814464	10.3365534	9.75059629	9.29527418	9.28826927	9.29732533	9.18197738
6.86047123	7.61056498	7.34752482	7.16279613	6.96817256	6.60946865	6.44780299
12.9990238	14.4266526	13.6460366	13.0551779	13.3790588	12.6584945	12.73803
7.83324748	10.1349089	8.97230178	7.86190079	8.58514508	8.26988033	8.37258172
8.92656375	9.89802208	9.45315682	9.60772756	8.81495571	8.26952646	9.15472167
6.26944465	6.54937561	6.1216222	6.09991668	5.89073243	5.91326403	5.99628547
9.37693715	10.4607395	10.1327735	10.0799834	10.0860008	9.84990906	10.0795741

6.2179106	7.39885568	6.23376499	6.08816514	6.19840123	6.58365206	6.54049865
14.2280618	14.7158312	14.5442062	14.6677281	14.4886498	14.1848684	14.2564195
10.3903625	11.392487	10.8474567	9.87021644	10.252948	10.8971444	10.7431985
10.91435	11.6683758	11.1594192	11.3891694	11.2698251	11.5781637	11.8034733
7.99712414	9.47067229	8.24824876	8.72267333	8.27064994	8.11691521	8.5146537
7.60775566	8.8153154	8.44097877	7.91419077	8.14876484	8.08152711	7.82161137
6.85291647	7.43164134	6.90302501	7.11471266	6.9388668	7.46387172	7.57469343
12.0610518	12.4585819	12.879754	12.7914706	12.9526415	11.1208126	10.8365814
5.30220315	5.85803671	5.96597817	5.44783142	5.90624412	5.44841072	6.54837682
6.49547388	7.16311526	6.77394755	6.73926486	6.48783457	6.36286417	6.27982134
6.33570387	7.31940478	7.28693288	6.68302969	5.97198492	6.2140152	6.14379237
6.67375427	7.5717989	6.97853201	7.42331428	6.82372516	6.49992416	6.88525587
11.9554625	12.9824774	12.6269873	12.5062032	12.5018857	11.9420548	11.800508
8.73915514	9.40765463	9.05083387	8.74515765	8.54721845	7.42571273	7.65754498
7.54288948	8.84473997	8.72561302	8.39867102	8.32574123	8.43868832	7.88128714
9.10263058	10.3744809	9.79150122	10.203091	9.80181957	9.34966162	9.59286662
10.3778298	11.7140411	10.9774467	10.2153486	11.089473	9.52646755	9.82365687
5.99132025	6.38245076	6.40486226	6.71978307	6.12656634	6.01326282	5.88359785
8.94238255	10.480355	10.0102232	9.61498842	9.66832623	8.91044066	8.73814125
5.9502316	6.50135984	6.24440646	6.18616219	6.07072635	6.59029886	6.39037999
13.7306867	14.6070794	14.4554903	14.5733892	14.2515068	13.8141478	13.5990027
8.11975923	8.8480661	8.37857101	8.33918639	8.13209199	9.05520406	9.45471631
5.64194249	6.48545259	6.28942026	6.28959603	5.81770537	5.70610171	5.50365149
6.98288066	7.57582284	7.27946851	7.28962701	7.17613025	6.75364331	6.88238261
12.247182	13.4212062	13.0940887	13.3888232	12.8610101	12.7971857	12.7740334
8.32628386	9.73764705	9.02396111	8.71944735	8.32612204	8.46943221	8.55592733
5.60647747	6.35882981	5.97596739	5.94335783	5.85285181	5.56866239	5.59468977
6.36519297	7.16028895	6.98557557	6.90586712	6.71211012	6.27100839	6.01268889
7.30053194	8.83254037	8.15803755	7.97492848	8.07291023	7.43019744	7.30751141
8.78475327	9.63935681	9.74621215	9.03068589	8.48755147	8.00724541	8.03328864
8.55372067	9.38987652	8.77308908	8.7721717	8.46054395	8.41215783	8.39068961
5.63526161	6.37532968	6.11961078	5.81125137	5.74842286	5.70245586	6.05261543
7.85284408	9.09737772	8.74279379	8.40911001	7.93972025	7.95229279	8.61607828
6.42602552	7.38682043	6.71583024	6.87408503	6.83862912	6.70128567	6.73649529
7.44241524	8.74163686	8.35322712	8.15118148	7.71610402	7.20190042	7.1100552
6.58227569	7.15445663	6.85270802	6.95828706	6.70810031	6.92634344	7.08727931
6.00605359	7.42432504	6.78864526	5.83863517	5.78828296	6.21834295	6.21427871
6.06100022	7.35616847	6.74416917	6.23261951	6.27225167	5.61515348	5.59440762
9.72246398	10.2276424	9.96821894	9.33731928	9.5555819	9.03368619	8.86557214
9.56021646	11.2757689	10.4210044	10.7424939	10.6251718	9.68515175	9.46262537
6.20591052	6.53204323	6.03183274	6.18584812	6.19900541	6.05396258	6.23533436
6.07303528	6.75196174	6.57030308	6.65115935	5.89111462	6.01104428	5.87791059
6.68679464	7.40485715	6.97690546	7.19195047	7.19075904	6.71433895	6.83243562
5.9311741	6.82088609	6.44665672	5.99739494	5.94427376	6.21932324	6.54600898
5.30937412	5.97051341	5.99488222	5.49426263	5.43529255	5.36925499	5.38580754
11.7093803	12.7033352	12.3249231	12.4623913	12.5575401	11.9732658	12.0514486
8.31886408	9.45009816	8.78006038	8.39139913	8.30973145	7.8407666	8.26016966
8.64708349	9.79269465	9.06540402	9.0245488	8.61595547	8.27591543	8.41863608

8.27488222	9.58156554	8.96984092	8.81442866	8.63417032	8.25582502	8.82012534
10.7837816	11.7472506	11.200605	11.1609479	11.0867168	11.7990209	11.8959649
5.96257077	7.0477892	6.65095264	6.35758719	6.02211787	6.0584492	5.86002171
7.8680385	9.64691579	8.81549153	8.49981082	8.37861306	7.87886816	7.78293009
7.25751513	8.42779597	8.01479826	8.12052561	7.42835766	7.00362472	6.66824254
5.5387157	6.3159663	6.1358726	6.0910454	6.11948504	5.68840783	5.76378097
6.41034976	7.93482961	7.54591433	7.09208796	6.78781247	6.54825027	6.24552457
9.23864477	10.5361297	9.72661583	9.47052751	9.12708329	9.35207985	9.78429441
8.1932326	9.68440114	9.01415664	8.75865496	8.29585203	7.61142471	7.86441894
6.39313386	7.28909947	6.77017361	6.69526727	6.46072078	5.92576686	6.00345495
6.1012248	7.09259648	6.7915927	6.68414267	6.88081652	6.36211443	6.63059302
5.72755892	6.39952848	6.34227116	6.06222211	6.01507378	5.7941	5.91175772
10.0946161	11.11603	9.80634533	10.2080953	10.428222	9.98961873	9.79828872
7.6504903	8.63294977	8.11358018	8.26569755	7.88504595	8.30558162	8.58186302
9.33076633	10.3071632	10.0081933	10.1073583	9.82178211	9.72344679	9.55961245
6.27494762	7.26918934	7.05368808	7.00290276	6.1589992	6.38238367	6.0299599
5.33982649	6.11351382	5.81345594	5.7151864	5.67058572	5.68080171	5.74257703
10.316139	11.5445796	11.3224808	10.6383291	10.6664948	10.1942632	10.6696788
8.58200495	9.67981452	9.01316576	8.76108145	8.86229826	8.4767134	8.21550291
10.9518828	12.8801085	12.3855308	12.1030855	12.1854485	10.9165259	10.4653174
5.88770462	6.51523369	6.25531019	6.26049242	6.44905122	6.53936	5.6678785
5.57591857	5.95524119	6.11516728	5.58877789	5.6239479	5.38691852	5.6113893
13.5590018	14.4860355	14.0024336	14.2988874	14.0968057	13.8666772	13.9220315
13.6437469	14.7877633	14.1858062	14.2580338	14.0973079	13.1455944	13.3392376
9.52458853	10.453531	9.68808312	10.3693651	10.6644553	10.4948076	9.33957524
5.9241436	6.68624838	6.13223299	6.60785995	6.14501464	5.87557563	6.10477716
7.05620958	8.27273148	7.86758455	7.70965044	7.46978995	7.67612281	6.94874567
6.54192799	7.49449802	7.19246741	6.88646357	6.67154956	6.60877465	6.21066764
6.86145793	8.40694307	7.62737994	7.47879227	7.05557962	6.90786409	7.27348698
5.93390471	6.76933934	6.41111147	6.02716489	6.08451397	5.8680348	5.61244952
11.5076311	12.7955263	12.1194282	11.8708714	11.8393495	11.8094977	11.5049679
8.46954455	10.5817166	9.46321496	10.2471728	10.5327377	8.11242439	9.31746711
8.66897006	9.3600609	9.32378685	9.53981408	8.49448842	8.25479526	9.34671428
7.87849423	8.54184585	8.37284765	7.9792211	8.04325099	7.42754751	7.49637127
7.56863488	8.41994122	8.0824482	7.92219606	7.66634403	7.57296089	7.36115424
6.9872697	7.76711978	7.43496009	6.97565311	7.4930156	6.6780071	6.56305087
6.69221515	7.41844887	7.04868195	6.98546855	6.44308295	6.3858436	6.62699464
8.10013667	9.90263966	8.78274338	8.55493222	8.8172523	7.73425371	7.94686067
6.17282871	7.08117876	7.2056271	6.88621236	6.44881161	6.18302811	6.01794328
6.35709344	7.80595728	7.37311049	6.66547521	6.90478633	7.50433039	7.44681781
9.00368543	10.5379774	9.85857723	9.48654082	9.58209681	9.07676296	8.78148463
11.2653636	12.7674299	12.0322672	11.9684537	11.8387816	11.2263983	11.2746455
10.8505385	12.419699	11.727041	11.5217452	11.1231055	11.552465	11.6141609
8.43937921	9.80692382	9.82790967	9.38513764	9.10714435	8.65000841	7.61883916
5.81517013	6.49145245	6.04986706	6.27802937	5.73417633	5.88296872	5.76381628
6.68052972	7.96768047	7.93176164	7.42676961	6.30544035	6.67939283	6.31771089
10.7025502	11.5555785	10.7806735	11.51922	11.6531689	10.1993313	9.42840519
7.21021545	8.05808321	7.87498135	7.37321579	7.89645109	7.0270722	7.10159545

7.19245754	8.79623271	8.23541498	7.92101435	7.76992708	7.12575337	7.10143052
5.5532794	6.49439273	6.35860444	6.09543732	5.63781685	5.81897835	6.2038045
7.47545459	8.31703086	8.34094497	8.14283869	7.63754577	7.1052674	7.38238799
7.93124751	9.22429959	8.44836042	8.92152768	8.52488526	9.00295084	9.32273938
7.19410479	8.41696047	7.71119615	7.47784001	7.81465552	6.77552732	7.16625148
11.2324611	12.274665	11.8081191	11.5247542	11.5532688	10.9014847	10.9428062
7.32331242	8.16085012	7.92901039	7.90030652	7.45521049	7.09651382	7.0983427
6.69037379	8.24564207	7.34023823	7.50166445	7.43612519	6.96678805	6.61473207
11.2803445	12.3025601	11.749554	11.9518872	11.4855783	11.8516495	12.175621
11.6063187	12.9196835	12.2776254	12.4752985	12.7478707	11.8287157	11.8507647
6.69820042	8.44683104	9.09556159	7.49119732	6.20035683	5.9429184	8.19272679
5.95507911	7.1087722	6.50033234	6.51835873	6.20471179	6.1921036	5.90305828
6.27734525	7.69068806	6.74454191	6.68591072	7.05667998	6.09136641	6.25610072
6.24474134	7.3454771	6.97056035	6.98270957	6.3807781	6.1099163	5.67981955
9.84349917	10.5369786	9.97781368	10.1396984	10.0293024	10.764588	11.0079341
10.0436056	11.1567499	10.412519	10.421459	10.1032668	10.2224206	10.1839951
8.78812645	10.3403798	9.55567047	8.95518085	9.5240623	8.20021546	8.16083903
9.22968453	9.80075077	9.49859574	9.29029238	8.61499504	7.83403739	7.93694882
7.94942696	8.91273099	8.54258347	8.49056091	8.31329937	8.18263172	8.66764948
12.1142444	13.1822989	12.6935582	12.9108578	12.6447292	12.8495969	13.1010754
6.20080996	7.77376957	6.67523021	6.54781441	6.09874525	6.53517038	5.8840198
5.92713035	6.89870416	6.67821701	6.54111598	6.14659882	5.8010342	6.03436584
6.11343526	6.47238103	6.31919557	6.37803192	6.44995682	5.95414488	6.10638266
6.0156753	6.79430669	6.37862762	6.26128182	6.20222415	5.77049581	5.85863006
6.22697336	6.68683105	6.29733689	6.41209286	6.16376982	6.19919333	6.35224528
7.43553359	8.76031677	8.2139844	7.88318824	7.63845691	7.27230319	7.12684486
8.40749849	9.56843879	9.54250319	9.32910969	8.87907653	8.06654615	7.75776135
6.51183439	7.47435816	6.66531886	7.04377647	6.99207388	7.02315331	6.94153244
11.3364379	12.8780519	12.3548317	11.8997497	12.2565717	11.6324368	11.8331728
8.05150914	9.20573559	8.99535873	8.8452821	8.50860415	7.64376025	7.56309634
5.38162531	7.38923639	7.8373441	5.25225101	7.05696713	5.24619524	5.05750963
7.54474811	8.43639341	8.63077967	6.43059539	7.7250349	7.30172573	7.63234457
8.18799285	9.25010131	8.44511502	8.86032875	9.02833045	9.10548035	9.12204938
7.12502801	8.24201937	7.84725596	6.73017257	7.4713899	7.69800242	6.85551262
8.04269522	8.81388282	8.35927649	8.76705951	8.19560794	7.7818272	7.80784433
8.10660534	9.56108723	9.04815552	8.81309815	8.48320149	8.11839347	7.89998583
5.85657742	6.25912746	6.2724846	5.51859682	5.93470449	5.44872754	6.03649256
5.99857057	7.69488924	6.86523095	6.56777982	6.67114049	6.14817067	5.92502456
9.48614156	10.5191104	9.9129682	10.4737027	9.84764276	9.32844695	9.56438655
7.08351762	9.24274135	7.81141965	6.55951292	7.70713822	8.5097564	8.19212609
7.41411692	8.53353415	8.05203489	7.45344021	7.84402968	7.19673883	7.13620369
6.090469	6.69638755	6.40761448	6.22567751	6.1010117	6.52200614	6.76408469
8.64945954	10.4434057	9.70269858	9.53287057	9.10374992	8.90382191	8.48876523
8.90083723	10.4046371	9.79710931	9.70419987	9.43403289	8.49655383	8.5670339
8.32623127	8.65666905	8.31478896	7.93421925	8.00963326	7.93702182	7.44518788
6.75896669	7.47010568	7.32514622	7.04859695	6.62540289	6.56600927	6.20382251
5.64798418	6.41164318	6.11199237	6.01433387	5.49985705	5.51069614	5.73480119
7.20732187	8.60256229	8.37121557	8.22654128	7.73577804	7.46925596	7.25612658

7.81410945	8.9542635	8.62670414	8.44799444	7.67300513	7.67539466	7.43673652
10.1926528	11.1109335	10.1100461	10.108284	10.5944049	9.69762737	10.1464338
5.88526515	6.42479262	6.32096225	5.8670778	6.14343526	5.7640985	5.47374016
8.80068542	10.2397095	9.75400275	9.67997317	9.24714592	8.77908239	9.38733195
7.20672722	8.723406	8.08529611	7.74291031	7.72427509	7.23695558	6.99969001
6.04668771	8.34564387	7.011859	7.02206482	7.25115667	6.19699902	6.89423936
7.42875963	8.62364742	8.3677406	8.18587447	7.70987165	7.40887232	7.3539725
5.74720369	6.61729275	6.23170984	6.13462695	6.25291346	6.03173748	5.8234024
8.41815916	9.79470584	9.26003295	9.07455066	9.12328803	8.37394265	7.82007171
5.58727707	9.1220007	6.32458868	5.86978654	5.96659358	5.84165794	5.8546859
8.26274321	9.38803171	9.08800375	9.01053874	8.76672799	7.88573358	7.95208716
6.38450766	7.10762929	7.10580168	6.98616174	6.40260196	6.53288638	6.54241671
7.22853782	7.11661428	8.13739952	8.34621009	6.0274175	6.89739292	6.59017488
6.32920488	7.5187279	7.0297168	6.34133468	6.7208202	6.5040672	6.87264287
10.4713491	10.770037	10.5602239	10.9064893	10.7481568	9.36124703	10.5502158
6.21750527	7.98412106	8.36840562	7.41328266	7.57955806	7.84875375	7.56330612
9.85820811	12.0220213	10.9256605	10.9750161	10.7914087	10.5203535	10.7591833
5.94594802	6.7709638	6.65233304	6.27265106	6.10726186	5.86434663	5.77632248
7.75853504	9.11794953	8.43235231	8.12502594	7.87458367	7.58369357	7.28621674
12.927009	13.9248403	12.9222493	12.9127256	13.3447453	12.426694	12.8860968
7.92965728	8.21400577	8.1639822	7.63923723	7.62502276	7.36959124	6.91712261
11.4315545	12.9125576	12.238723	12.2836634	12.0756794	12.273228	12.6187549
8.21001717	10.0764295	9.34450653	9.0859581	8.90019722	9.01456117	8.70833693
10.8317672	12.1532306	11.262459	11.1783605	11.492408	10.9118024	11.3808961
7.21041127	9.13830176	8.56384148	8.66585001	8.60341605	8.31153899	8.63870452
7.68732109	9.47712743	8.58137083	9.1531355	8.77786975	8.13925102	8.71461302
8.00473115	6.85876804	7.10996956	6.40565155	7.55105761	6.15701217	6.60872779
9.16575294	10.2950652	9.72398306	9.49743094	9.34451673	9.38073681	9.22789772
5.26472407	6.12913668	5.63032334	5.55887343	5.34705472	5.55934956	5.33116453
11.113694	13.3294754	12.041115	10.9923535	9.11893043	12.2916338	11.9752773
6.11987593	6.78482065	6.33929366	6.55695523	6.3049724	6.29286456	6.16000511
5.81629045	6.53396975	6.89695597	6.63074662	6.314206	5.94134134	6.01084025
11.0129003	11.7774308	11.379916	11.4953339	11.474648	11.588739	11.1598865
6.59050531	7.50988345	7.29602876	7.0720018	7.07681346	6.6455446	6.82124591
9.05497732	10.4025868	9.62437163	9.66566813	9.52263881	8.31300363	8.37467567
8.95179496	9.66310922	9.72792777	9.79928162	9.30977987	8.49229666	8.67917851
6.5165113	7.26599659	6.59368049	6.59511274	6.40560867	6.53545782	6.63632083
9.09090158	10.2466919	9.99040893	9.63872099	9.58204354	9.16199195	8.85647875
6.59856947	7.37092362	7.35341063	7.06962269	6.651577	6.34234193	6.47062911
12.0618272	13.2230389	12.5537382	12.6155747	12.5704615	12.8462896	13.1718276
8.62876157	9.40930904	9.35176656	9.32081589	8.87232736	7.71181149	7.74604869
5.56929847	6.46212759	5.973693	5.95502399	6.00126203	5.88559389	6.0122017
7.23537285	8.25622851	7.74462465	7.22475419	7.41123725	8.06228634	7.80410843
6.32906456	7.47245609	6.92229777	7.57576462	6.71388136	6.11638971	6.50052419
11.8119406	12.4960041	11.4278246	11.3190195	11.4746936	11.6324836	11.868855
6.43808705	7.46467153	7.00347968	6.58338098	6.72783062	6.01326862	6.51250535
7.76166625	8.59619348	8.1214527	8.81099667	8.2213933	7.75697723	7.9972591
7.29634269	8.59387624	8.21330694	7.88809054	7.58675199	7.57727624	7.4721498

6.80138374	7.35120163	6.94245117	7.08143657	6.92254278	6.96250275	7.00111878
6.18364848	7.01554998	6.9982508	6.59664434	6.46014279	6.00674167	6.0117756
5.88354702	6.5552301	6.3515778	6.11253209	5.95262851	5.69943947	5.83675329
6.85940806	7.68634294	7.49093108	6.86176187	6.84843641	6.47569939	6.4080272
9.80178045	10.6994857	10.0165855	10.5297341	10.23143	10.481747	10.7890698
8.53686341	9.89309808	9.12450365	8.76169948	8.77230963	8.4559009	8.30375518
11.1522196	11.9300524	11.6041302	11.5617629	11.3237692	11.7287488	11.9397111
8.36358812	8.88460097	8.98535669	8.18801512	8.08894297	8.04602279	7.86246503
6.17559186	8.18594879	7.44675662	7.40256188	7.19551198	6.98098152	6.23461954
7.48280392	8.7792305	8.54982548	8.35832768	8.44993521	7.29359488	7.56612865
7.39609354	8.60750084	8.0908148	7.74278369	7.2802836	7.72713765	7.03729875
10.644845	11.5431121	11.1096335	11.1631842	11.1820343	11.588717	11.9947293
5.64892355	6.47953923	6.47577428	5.82696204	5.78169858	6.12219291	5.92028401
12.687207	13.9140674	13.4107389	13.5215698	13.2939041	13.3810454	13.6776029
6.4564157	7.38160005	7.19945642	6.37806522	6.21893845	6.51857715	6.19471431
8.8214041	9.60079678	9.0661258	9.24899443	8.85172062	9.49702729	9.85948804
8.10169577	9.41813807	8.77267483	8.6831227	8.77338519	8.02600381	7.86949391
6.05198586	6.48127504	6.25207083	6.22666805	5.8443284	6.02755927	5.93962129
8.06665378	9.53518185	8.30476656	8.64640966	8.50457352	8.23964674	7.84068478
5.58757862	6.013433	5.75420767	6.20923968	5.72376004	5.74234151	6.00176892
6.97040619	7.59111613	7.07939518	7.11878745	7.02392397	6.69562502	6.7877276
5.43518919	6.06669693	5.90155479	5.56822094	5.63343586	5.71141066	5.74642188
5.75072949	6.21904884	6.01495845	5.69360209	5.77530555	5.68208192	5.72669773
6.00109986	6.70139103	5.99002134	6.37996867	5.97855695	5.76035989	6.12292633
5.87613013	6.93729142	6.2771477	6.55410148	6.16439048	5.5955702	5.77516646
5.35938482	5.82427347	5.6358603	5.36674884	5.4063353	5.32452585	5.26076865
6.08485451	6.67921406	6.34346183	6.27650988	6.17142843	6.02103681	6.38011712
7.29880401	8.12033742	7.65287221	7.74927279	7.83314626	7.07057073	7.19162878
6.02315464	6.82068701	6.21181823	6.34641764	6.02557037	5.69182279	5.90089385
6.73692006	7.8284507	7.2884084	7.79663993	6.84875437	6.38577804	6.35587293
5.52475451	6.04657597	5.75376577	5.90990676	5.43138829	5.4569244	5.48739534
5.51646781	6.22810756	6.38389054	5.55092744	5.71807909	5.79609855	6.11304763
6.37797242	6.92810379	6.63553835	6.70970282	6.28355791	6.41557903	6.1573528
5.44116274	6.0107729	5.91132056	5.44389967	5.57668659	5.47400408	5.39551254
7.09255314	8.32597593	7.6365949	7.68623157	7.25268152	6.66608621	6.79952642
5.92655266	6.74173797	5.94108541	6.44060543	6.03929876	5.86645799	6.46671472
7.00010933	7.84583877	7.1697677	7.27942208	6.83510551	6.63201674	6.69486348
5.94120657	6.79399859	6.48044561	6.51553207	6.3327176	5.86427209	6.05898833
5.53455284	6.26891192	5.97061326	5.7369839	5.6048921	5.53592355	5.52473477
6.71821228	8.34966914	7.5629964	7.2391641	7.24453985	7.43715615	7.18859973
5.79977222	6.97359521	6.77183541	6.56029095	6.65616169	6.14273793	6.32846742
6.66918696	7.41581358	7.01759125	6.97324619	6.8327065	6.36883521	6.56827735
6.73020926	7.34549839	7.24773862	7.05636785	7.09720098	6.68366558	6.68288506
5.91311321	6.7899835	6.28293421	6.22671178	6.10688261	5.83947819	5.77371657
5.17930841	5.73805766	5.67471741	5.26194371	5.18166353	4.99717316	5.24943245
6.08724748	7.29611505	6.9567223	6.90527869	6.66119235	5.87797389	5.82262742
5.32185812	6.17319761	5.61750831	5.28294644	5.32783069	5.68395438	5.4367182
5.84644793	7.28383568	6.03904822	6.38635072	6.9394605	6.00157889	6.53100163

5.43243667	6.22574074	5.86338792	6.32333206	5.7435699	5.23432159	5.64363631
5.99205529	6.84959776	6.23093098	6.16628824	5.8613001	5.96721012	5.85787229
5.95296368	6.66373598	6.4156882	6.16288151	6.25171663	5.87742304	6.1751567
5.88455616	6.00813411	5.97823888	6.11048143	5.64664079	5.61024915	5.87540534
6.16294353	7.62421278	7.07082186	6.71863288	6.40952111	6.37188413	6.12040316
6.41979067	7.61762908	7.0547302	6.51732027	6.94936684	6.76242855	6.92708578
7.60906199	8.6232893	7.93066225	8.13858463	7.80152342	7.86446785	7.95673392
6.97102731	8.20045776	7.69686393	7.52311026	7.02523814	6.64546102	6.60624485
5.43331102	5.96573373	5.95904051	5.813029	5.69921318	5.53711246	5.51724829
6.51280082	7.2758647	6.51493887	6.83588493	7.50764878	6.31611207	6.65363761
5.95314113	6.26386165	6.05005759	6.2967228	6.0555306	5.82990891	5.78403828
6.38604595	7.8314276	7.08964221	6.73316857	6.86621316	6.32445313	6.38501301
7.98189431	8.99991518	8.45444407	8.02571099	7.93759275	7.52553725	7.51841554
5.52032072	6.46192107	6.06250751	6.18505381	5.58463636	5.52961983	5.75786893
6.71838081	7.61362226	7.01337516	7.07628865	7.0630995	6.22029919	6.20186691
6.03483659	7.63769791	6.35637186	6.9685781	6.18556708	6.03310293	6.405231
5.36958967	6.60397596	5.83917304	5.57048631	5.72125863	5.45452007	5.5703175
6.06498931	7.33479708	6.64018969	6.60472564	6.32554814	6.15478713	6.39747117
5.72038956	6.35421288	6.0352137	5.81928268	5.88741609	5.48817692	5.61241443
5.31336486	5.79547242	5.63067485	5.23331634	5.29536378	5.02893774	5.17761328
5.342133	5.78145965	5.74190755	5.56984103	5.40951653	5.2965679	5.47238606
6.38594814	7.14115993	6.73429233	6.00001195	6.43445543	6.21018038	6.30913598
5.32224581	5.92497636	5.61418096	5.38761241	5.42632481	5.4317834	5.43692171
5.93683814	6.86775565	6.33990789	6.26099422	6.14174442	5.70936426	6.18454254
5.45820481	5.96906505	5.67730978	5.50677774	5.66091037	5.41504167	5.56358923
5.35256434	5.68994907	5.66479171	5.25886373	5.50529646	4.98697243	5.28183695
6.57483357	7.37827751	7.40141715	6.84185742	7.1325388	6.57981206	6.17849854
6.07513404	7.09558691	6.65565665	7.31916449	6.44835852	5.96110862	6.02173577
5.81272924	6.18003684	6.05998943	5.62453639	5.75840579	5.54573645	5.41825405
6.57510898	7.61555902	7.47353286	7.80824281	6.76783883	6.3507626	6.25386771
6.64139921	7.82025173	7.06167793	7.07770552	6.8774866	6.24116557	6.384136
5.60956025	6.73568328	6.33857132	6.04503008	5.87982215	5.97030472	5.86417128
7.60135779	8.91563981	8.4620425	8.11122124	8.19224053	7.75408712	7.83615895
5.752204	6.55071401	5.96998069	6.20428238	5.97307189	6.16146307	6.6125399
6.68009505	7.2965088	7.06232143	6.93237895	6.32102072	6.70387728	6.88071124
5.99503423	6.50087581	6.29681015	6.58053521	6.21078081	5.699953	5.59946777
5.72091539	6.72892455	5.98858381	6.56414873	5.73712455	5.45822482	5.93190687
7.98991954	8.90444526	8.35019002	8.75860533	8.32064714	8.1949597	8.48349456
6.47331205	7.00691286	6.82926861	6.21205392	6.61827772	6.17790384	7.07347537
6.27329358	7.23157737	6.84920991	7.21782396	6.35473406	5.99907435	7.07229878
5.56472203	6.17460596	5.83182025	5.84972435	5.86421116	5.27658339	5.45950933
5.85628882	7.32116507	6.20403563	6.38850109	6.02187721	5.88760788	6.03005462
11.6076087	12.8646884	12.1888985	11.9793209	11.9851574	11.1476215	11.8484645

C289_NS	C139_NS	C120_NS	C063_NS	C019_NS	C269_NS	C284_NS
13.2477475	13.757916	13.5709183	13.6264836	13.8617548	13.0012666	13.2040693
6.59928536	7.57733214	7.90435283	8.0113307	7.20382858	6.39430663	6.56459629
6.51846495	7.3070669	7.29080866	7.10529779	7.2434187	6.47646562	7.04226457
6.36595913	7.39293473	6.82305	5.82649663	7.13659541	5.84396601	6.48265567
7.06749137	7.37265441	7.12439121	7.13794405	7.45540449	7.03911755	6.10511693
7.35340799	7.89085644	8.15842136	8.11571736	7.62380103	6.80584574	6.69563198
6.91244988	6.86224184	7.26589348	6.87125103	7.27361752	6.16686083	6.20065108
5.56875388	6.09021746	6.37324155	6.31181424	6.0881346	5.94680711	6.12413807
6.9740302	7.96347644	7.88018165	7.52624178	7.60035971	6.58802663	6.64765845
6.06439098	6.76182778	6.70237496	6.75662837	6.70728182	6.15893358	6.51406386
6.37660035	7.31455877	7.32110011	6.91621388	7.13578725	6.33583319	6.92110423
7.56450088	8.72836544	8.43361215	8.11478553	7.98015043	7.27103249	7.87375969
6.18783523	6.64030525	6.88757645	7.20922622	6.73260568	5.96721104	5.83679266
6.67671733	7.9188847	7.58575802	7.36235153	7.25960111	6.42352103	7.3438244
5.80366231	7.52631222	7.26771182	8.09033688	7.65276468	6.43273514	7.46666219
6.95744685	7.48795336	7.32717356	6.95324778	6.88783717	6.64591489	6.92495973
6.69442893	8.27208899	7.57872441	7.20277815	7.37685817	6.41331109	7.67960958
6.81997084	8.17787047	7.8518015	7.43096096	7.80322058	6.46158094	7.81717362
5.33276593	5.61637711	5.73465652	5.85009255	5.83147903	5.51692033	5.46646692
5.68428155	6.3664635	6.49230876	6.22178159	6.2428277	5.97589702	6.13163352
7.14090133	7.42589061	8.77061666	8.67807895	8.72759105	7.90924868	6.76762435
5.13787563	5.96529045	6.01865687	5.83211531	5.61788029	5.36878127	5.39800245
7.15042868	8.96248077	8.2833727	8.37633423	8.11576157	7.02706224	7.267621
11.7709558	12.2153407	12.5677448	11.9283896	11.7851332	11.3925187	11.2311888
11.9103538	12.1140364	12.437652	12.6678001	12.7254562	12.0800707	10.7177475
5.66658761	5.95518933	6.14771474	6.08519709	6.14786287	5.70548835	5.66453774
13.6351059	13.9622797	14.1030665	14.2569738	14.1765191	13.7356597	13.4822193
7.53270112	8.33168014	8.20874257	8.26399119	8.20102902	7.70706849	7.1386179
7.29333094	7.40744623	7.74491051	7.71477304	7.70884248	6.42463426	6.03501415
9.35603704	10.0171341	9.90589314	9.91675324	9.83445556	8.71161914	7.95602993
13.5463302	14.0432324	14.1900129	14.0513169	13.6437333	13.756352	13.4545679
12.4201205	12.7348821	12.8867959	12.8499236	13.1851429	12.3025927	12.4243479
5.34417694	5.99324027	6.3287496	6.36665513	5.74941547	5.49203519	5.55548458
7.86944024	8.7930375	8.720667	8.32854434	8.23750492	7.34104997	7.52265999
5.11935286	5.5962998	5.67297233	5.63113048	5.21428104	5.26194145	5.15333638
11.7384077	12.5923503	12.4541952	12.1154654	12.4612546	11.0079453	10.8929806
9.29575149	10.1842233	9.94778624	9.89503127	10.343621	8.76564248	8.66811239
9.23513778	9.98306058	9.74579666	9.3710805	9.2207152	8.63689356	9.0496501
5.85169108	6.61524737	6.42519928	6.69486488	6.24839802	5.76718309	5.89048147
6.65472767	7.67642539	7.16043015	6.991159	7.36572753	6.46705217	6.53603331
8.89481564	9.32290046	9.72109081	9.30693639	9.3158078	8.35398264	7.98589828
7.74688847	8.0185702	8.09637256	8.03827978	7.86439975	7.10827353	7.54106098
5.96388607	7.18808041	7.41190752	6.5746288	7.2267898	7.01948843	6.30697233
7.05169728	8.02494848	8.06717511	7.19375795	7.23658924	6.53152541	6.85661725
5.77002583	6.03390463	6.46741791	5.96479968	6.48464328	5.75794651	5.77710537
7.36763403	9.42260683	9.43204052	8.91057498	8.2792159	7.13184153	7.51361154
6.29583504	7.63666232	7.16744324	6.93277105	6.78633367	6.11872351	6.83428395

6.68969613	7.94968783	7.6895927	7.3982888	7.51035434	6.58341318	6.64737784
6.09852525	6.77312734	7.74525952	7.28767083	7.18289089	6.74877652	6.87938843
9.45355791	9.87807268	10.0436357	9.63942555	9.45864386	9.25662575	9.50390185
10.0390068	11.0432625	10.8488928	10.9429418	10.4914543	9.83081259	9.62323094
6.1642034	7.75243016	7.97115035	7.35335594	7.12389688	6.09609926	6.86652847
5.85795015	7.01572614	6.79820608	6.55363605	6.5447104	6.19511074	6.63578834
13.8071456	14.265522	14.4242431	14.4741026	14.6669629	13.9015127	14.0087394
6.66146316	7.96963345	8.26281319	7.22703249	7.64018853	6.28115612	6.19923751
5.58073734	6.27445419	6.28147831	6.10999421	5.95478374	5.66188952	6.08746072
8.65686208	10.244547	9.75016866	9.49531322	9.49156232	8.8169238	8.64986906
5.62917299	5.92757129	6.13046822	5.82273525	5.88711726	5.57335877	5.53003421
6.45478572	7.91841665	7.93851869	7.78573428	7.73356381	6.89314982	6.96041904
8.97615596	10.1195442	10.0467993	9.62749339	9.52371602	8.54194494	8.3811514
7.30251007	8.43859976	8.10044789	7.65330943	7.34092183	6.68361646	6.57815649
6.63002288	7.49611237	7.74314533	7.47556646	7.79087302	6.44801237	6.2567482
9.41395345	10.1181897	10.5924757	9.80082388	8.99823444	8.4884656	9.59075713
7.0576019	8.03101687	7.74708927	6.8836648	7.28388937	6.10563953	6.37253462
6.32303061	6.46677344	6.85725822	6.299315	6.3158926	5.51824745	6.12823277
6.61173483	7.16238529	7.32906617	7.49982915	7.46646398	6.55906714	6.39760225
9.01709401	10.1513553	9.95345077	9.97733717	9.74416059	8.96292983	8.82904911
5.99404631	6.95793549	7.06117459	7.02366787	6.73076493	6.13901613	5.97890833
11.2543596	11.9258841	12.0467369	11.9308955	11.8429812	10.9628078	11.3495041
8.21435944	8.91986681	9.18210479	9.00784414	8.91628883	7.82288371	7.0629409
7.46379164	8.29859784	8.13454645	8.19640246	7.99070305	7.4947428	7.75055358
7.79472517	9.20785479	9.2350085	9.13859999	8.8209282	8.05777354	7.60474298
8.33884333	8.79707071	8.9637761	8.95913026	8.62482542	8.10376041	8.52033031
6.669401	7.74473766	7.64235862	7.58626034	7.58840958	6.69881716	6.22347758
7.22318118	8.12492931	8.24798826	8.27047989	8.80146005	7.33154938	7.55862734
9.07306506	9.45506291	9.74906072	9.5462505	9.5209119	8.68716184	8.64784873
5.43701496	8.13782471	7.81877708	7.03759747	7.48603131	5.73471558	6.48784341
7.4297532	8.74294162	8.58898479	8.59988533	8.37163161	7.08813311	7.3917514
7.92928268	8.67296166	8.95534623	9.05079253	9.14708635	7.9018691	7.13181581
8.42719382	9.83715942	9.63525033	9.10594229	8.98751813	7.48893751	8.1086567
6.3217867	6.65926669	6.87340228	6.86834724	6.72230344	6.1920886	6.37983372
8.39892621	9.04948919	8.94883822	8.61203322	8.85151357	7.66711935	8.76894121
5.49169792	5.79883543	5.87569112	5.85811879	5.89258556	5.80553598	5.62963027
7.38772613	7.40093748	7.79290675	7.68203809	7.45714527	6.57331469	6.81876111
6.51074771	7.47702479	7.68472548	7.52821341	7.63030062	6.81699642	7.48507014
12.2488538	13.5047669	13.4019821	13.479611	13.2726329	12.0516831	11.8397396
9.29042072	10.4193166	10.5488672	10.2144891	9.94826253	9.04223912	9.45405433
6.5595634	7.31356603	7.77221559	5.69465319	6.09703849	7.15301184	6.99998986
7.71256345	8.74225924	8.56315546	8.86922954	9.20910727	7.94222652	7.34810315
6.08550008	6.92646917	6.90399147	6.76780176	6.63257432	6.24526899	6.41374266
8.2656413	8.88154043	8.61743441	8.26336358	8.80796472	8.13816734	8.22377922
7.74710001	8.36682183	8.7017975	8.63524833	9.11134828	7.37883325	6.89705645
6.23229965	6.27283914	6.52278944	6.74515261	6.62029022	5.77921322	5.77710142
7.17047092	8.98065371	9.27621376	8.52076442	8.69799895	7.88792793	7.30491346
5.42019773	6.34400556	6.43237754	6.3112933	5.84991472	5.3835792	5.34180149

7.42517142	8.22954707	8.34595516	8.80173358	8.37700919	7.91642051	7.03970321
8.48431298	9.12692486	9.27325128	9.10953869	9.13608242	8.06043547	7.11647601
6.95577194	7.9145143	8.0241828	7.4939699	7.64481815	6.93270492	6.92265932
8.22163255	8.94933532	8.91746219	9.01422305	8.87304724	8.01374474	8.58378119
11.3985588	12.3496274	12.1200778	12.1226433	11.7979707	11.0474014	11.4379258
5.6546956	5.97082352	6.25420947	6.01110848	5.99657766	5.89436597	6.28855716
8.25973008	9.50889447	9.19641328	8.59769727	8.51340665	7.38939903	8.23545615
5.57357751	6.30695575	6.49731856	6.13967249	6.34797174	5.80131156	5.82651722
8.81135125	9.55346899	8.66451235	8.43018406	7.80095812	7.63660505	7.91808326
10.8315147	11.5608907	11.770782	11.9288988	12.3123773	11.0658234	8.9108109
6.45379855	8.17898298	8.16665018	8.24954347	7.87623698	6.50593522	6.72690749
10.9472658	11.5059658	11.7779149	11.9667174	11.0681824	9.96757356	10.4383844
10.550209	11.8962601	11.5057208	10.8657511	11.0792661	9.96231418	10.2829112
5.85376665	6.74032991	6.94959213	6.9662078	6.51453181	6.21971451	6.27709469
5.78958816	6.43713667	6.45434026	7.10718778	6.00178761	5.72873385	6.10277863
8.561037	9.75534011	9.65706812	9.78794026	9.86800238	8.39557912	8.39947618
5.2534508	5.95255676	5.97356697	5.87218679	5.82812125	5.34915329	5.8666877
12.3793946	13.5390825	13.5360869	12.9071532	13.2404408	12.1772218	12.080004
6.13155018	5.90852689	5.88455812	5.99609494	6.02845069	5.50685484	6.64368969
9.66311366	11.7731524	11.9847374	11.7057171	11.7667587	10.5746966	8.84600306
10.1004652	11.647185	11.4329803	11.2202634	10.8477331	9.32745205	9.27530067
11.0662197	11.7037366	12.0551729	11.2405248	10.9502234	10.7470307	10.2452259
6.69094642	7.46686282	7.36088482	7.26799827	6.89404286	6.58169727	6.64007672
6.91470563	8.86838798	8.24835172	8.18141418	7.90435464	6.77456306	6.76655276
5.81030145	6.60127027	6.6390689	6.64159892	6.38326382	6.11978693	5.89539279
9.82745608	10.8774889	11.1019921	10.8366769	11.1102954	9.78704828	9.60363599
10.1964903	10.9091731	11.1741361	11.2353352	10.9638498	10.2537317	9.50822382
7.98268904	8.68912895	8.59262447	8.41658644	8.06592984	7.93077932	7.21818832
7.12171067	8.69589501	7.54467162	7.11099896	7.07748336	6.43573709	7.22543594
10.0363715	11.3529863	11.0497301	11.3517481	10.951335	9.76763725	9.69626061
5.97241351	6.41686085	6.5079045	6.75743402	6.38640729	6.14929229	5.85532175
7.49586302	7.63528425	7.6205065	7.93776633	7.58498129	6.99111931	7.0868952
11.1113365	11.6137508	11.6266572	11.1088341	11.4519791	9.97987816	9.28762951
6.99362682	7.08407171	8.13081661	7.57445048	7.30879953	6.49760199	6.73343434
6.39596317	8.02679951	7.81701113	7.49245249	7.13312371	6.10673168	6.35070692
12.3012084	13.1496027	12.7933267	12.4867694	12.7988367	11.6067957	12.1691621
6.57963152	7.93338674	7.94411173	7.76216807	7.0763699	6.33964463	6.05385748
7.17389796	7.98568144	8.14714895	7.72181079	8.00774942	6.63945214	6.88395066
10.664209	11.721227	11.8678305	11.6052647	11.8311251	10.8612506	11.4009584
6.84681547	7.43646504	7.39920784	7.38755555	7.45898973	6.69464622	6.95487445
6.26476366	6.94087383	7.15392792	7.26593379	7.92040348	6.43782996	5.82895445
6.51017344	7.00259002	7.17479489	7.29496254	7.34076342	6.28364661	5.87570537
10.0136409	10.473037	10.4248875	10.0523385	11.2810613	10.6166674	11.0588076
13.4329372	13.9248301	13.7818535	13.8358126	14.3516729	13.6339216	13.2039563
7.86117502	8.90092928	8.69408935	8.21371374	8.42463661	7.48910455	7.68298967
11.9121382	11.1886939	10.983994	11.4512194	10.1903173	9.50450043	10.1841724
6.72676992	6.6499215	6.99469625	6.92723989	7.28165282	6.20219672	6.1838785
6.28423076	6.97121244	6.92676446	6.54000649	6.71477716	6.12897425	6.25157728

6.00077096	6.82601834	6.53162553	6.42863319	6.63827473	6.06583379	6.23049106
8.73521617	9.53674085	9.5255537	9.27460133	9.49036295	8.34309064	8.31663136
10.2232596	11.4070585	11.9001834	12.3964681	12.3243575	10.9119559	11.0442288
12.9576069	13.9580714	13.7847698	13.6284296	14.0307309	12.8758655	13.3019177
8.4484393	9.4425241	9.61763808	9.31121495	9.05721549	8.20126334	8.79282998
6.69073136	8.54237569	8.1048346	7.84841888	7.69270825	6.73745558	6.30783177
6.3343389	6.27610021	6.40348878	6.16794936	5.99518306	5.74442081	5.73287128
10.7414181	12.7347183	12.4809194	12.1834481	12.059288	10.385995	10.7779449
10.0785231	10.9180776	10.4101884	10.4002401	10.4359713	9.83995864	9.80952008
6.72321041	6.75977236	6.97723412	6.56410863	6.6724805	6.16869318	6.18003365
10.2632457	11.9577996	11.807456	11.4464212	10.9610459	9.43146062	10.1935488
7.87290754	7.83007528	8.19281406	8.3342706	7.85741935	7.87379475	7.13924129
8.66527409	9.60972136	9.63159198	9.3066423	9.3241981	8.36247778	8.59187797
5.66868335	6.39062986	6.33797952	6.24956423	6.08621012	5.56728745	6.22475708
14.0350293	14.3970669	14.4390244	14.5930228	14.2169269	14.3338416	14.0191494
6.88529371	7.20252841	7.82662793	7.22583693	7.32290789	6.88665501	6.5026856
7.28067985	8.00290501	8.0187421	7.45052753	7.40900051	6.79076813	6.53197651
6.12452025	6.97083985	7.17923236	7.01989536	6.93546564	6.13341968	6.04616623
8.56677635	9.61031564	9.62351849	9.73119733	9.72549339	8.23796948	8.27024626
5.70045026	6.09038304	6.35281236	5.89951958	5.6632094	5.38646644	5.73126608
10.7711164	11.5931245	11.8946958	11.3877809	11.4587803	9.8490793	9.58763221
8.9468364	9.32487022	9.46260942	8.85623471	8.60014559	8.07607857	8.49234032
6.53462878	7.49767064	7.94154241	7.7222563	7.54747297	6.80300612	6.82590517
6.14801932	7.2005838	7.37678872	7.04671914	6.69540092	5.85766979	6.31841736
6.39442325	6.67886066	6.80287175	6.86847565	6.14989199	6.35536431	5.99847783
7.11377654	7.06929938	7.47675673	7.10825576	7.22499337	7.29729873	7.12039031
13.8183495	14.3929828	14.2099681	14.1430898	14.4494279	13.5797272	13.8532306
5.33709164	6.39656711	6.47696957	7.70907002	7.51279655	6.11164441	6.10729702
6.53584006	7.32589726	7.37256474	7.27042989	7.16118732	6.30565412	6.37348257
5.41650334	5.98707001	6.11188912	5.7978091	5.99293743	5.83822974	5.68813055
12.8628249	13.4964032	13.3711368	13.1242992	13.4439679	12.0684387	12.7679186
7.98633641	8.98014243	9.27388999	9.02338733	8.58048998	6.97449375	8.22673297
6.50765924	8.3218537	7.40284324	7.48933018	7.53332973	6.70502546	6.05881888
5.80919627	6.66478432	6.72401172	5.9994873	6.01267415	6.02836449	6.02247257
5.56112875	5.93365147	6.24408616	6.39316682	6.11658995	6.15316307	5.83752324
10.9800982	12.0284569	11.9541421	11.5637988	11.2133552	9.58963224	10.3790913
7.84048086	8.40795559	8.90352007	8.22940238	8.65662972	8.03784507	7.07368744
9.18713157	9.68607448	9.91307954	9.81047138	9.63071707	9.02231926	9.03195478
9.54864431	10.4535001	10.3998654	10.1656658	10.4849256	8.58626898	7.91420452
5.44083795	6.05756746	6.46170699	5.98236618	5.91886228	5.66625412	5.65687616
5.77638987	6.41259136	6.63434365	6.50870008	6.41720105	6.02381067	6.04536084
6.23316082	7.14187417	8.82869727	7.90847849	7.75517468	7.22341772	7.22675512
8.7158347	9.68694605	10.0187552	10.3585292	10.3056617	8.67222554	8.77535744
6.11641591	7.04078396	7.49280977	7.32671081	6.63730521	5.9022521	6.48766385
8.04285388	8.91402539	8.725536	9.15040025	8.61393713	8.37145867	7.57793784
7.82404862	8.43597398	8.68558945	8.57115459	8.809381	7.78655454	8.03282322
6.83763046	7.81522253	7.64612593	7.47626502	7.75163243	7.08280689	6.94216024
5.30830881	5.83208694	6.02902258	5.93766689	5.67672072	5.57548721	5.7553109

6.1431048	7.46332573	7.44234805	7.31829515	7.23034041	6.45267252	6.51481866
11.6867671	12.682205	12.4423853	12.1332501	12.479291	11.2140355	10.9697398
10.4341059	11.464951	11.053442	10.5742927	11.1624398	9.76730736	10.2028915
13.11068	14.0239302	13.8258234	13.7779868	14.1906396	12.8055462	13.35702
12.2499262	13.1089783	13.0281792	12.8252613	12.5388633	11.6687569	11.2508781
9.09516332	9.61495953	9.9230092	10.0658375	10.2494762	9.1538565	8.2916321
11.9726123	12.6753367	12.7935771	12.5119283	12.1512081	11.267482	11.6224308
6.35511408	7.58179185	7.10109745	7.21045511	6.7697572	6.34981552	6.44379278
6.40829866	7.20384326	7.52294633	7.4882555	7.04584265	6.65812578	6.67366383
8.72026205	9.60141536	9.98871025	8.4105777	9.22103459	8.38064461	8.20819338
8.23323043	9.34908414	9.47783677	9.09991531	9.19982986	7.78805922	8.0607143
12.178869	13.0587878	13.1710747	12.9542608	12.4233282	11.1978887	11.9727278
9.33440336	10.3628457	10.2073658	9.76828574	9.79201197	8.55504423	9.16570974
5.77165453	6.53733102	6.55408443	6.23203654	6.13633517	5.57138709	6.08352698
8.12647579	8.74575633	8.56385292	7.95591082	6.7596805	6.79004608	7.10614414
6.04931253	6.71473046	6.82901358	6.73735958	6.67269405	5.77198802	5.93185937
13.0131047	13.7300689	13.414602	13.5464966	13.0130597	12.6715528	12.2525283
5.31901343	6.01456151	6.13766213	6.14079072	5.94452467	5.97108987	5.70705289
7.0239916	8.4795596	8.17136197	8.41500735	8.19647623	7.2368542	7.3096928
8.21499562	9.78863743	9.80604995	9.55110357	8.7264641	7.71178603	7.89016211
5.71947179	6.22572532	6.32169202	6.63845155	6.20053535	5.77612136	6.13654968
5.50094797	6.00015125	8.52009061	6.29654551	6.13384892	6.04320778	5.87291493
6.41124098	6.9194843	7.11823201	7.44906278	6.94925477	6.68545525	6.9240488
6.52058615	7.5874235	7.34665379	7.00718765	7.1807476	6.56005505	6.29221008
6.54779837	7.65064538	7.56895554	7.39116109	7.00059724	6.37804024	6.618977
10.4292728	11.9542509	11.1112009	10.886413	10.7120601	10.1977106	10.0529973
9.69209674	11.1614796	11.0707482	10.4570545	9.98784382	9.2020234	9.78333747
9.51543552	11.3443503	11.0078753	10.8630572	10.3737961	8.73092181	9.21494464
7.5878915	8.49012805	8.69504277	8.37859052	8.26739956	7.40740119	7.70951328
7.29530426	7.74453518	8.17725657	8.12337909	8.00950674	7.18965162	6.84942262
10.4194525	11.546663	11.7479932	11.7020489	10.8761578	10.122322	10.4216241
5.46894785	6.16504767	6.25482171	6.11319728	6.22261081	5.66856139	5.50186584
9.61502356	10.6153988	9.91602603	9.65755962	9.32763707	8.74416009	9.20824631
7.99413221	8.35435078	8.70664037	8.20348162	8.59525674	7.69443102	7.908637
5.86649607	7.30676623	7.13653389	7.00148138	6.84573341	6.04478141	6.21401559
5.93849588	6.38934137	6.70736604	6.42318871	6.63446316	5.98887802	5.94459775
8.56486018	9.82780887	9.90091012	9.44096694	9.08882745	8.53505682	8.55681717
10.7138968	11.2450161	11.7592541	11.4943466	10.9382865	10.1214791	10.0941582
13.694408	14.0648025	14.0525867	13.8967959	14.6738523	13.8601908	13.6803584
9.83708749	10.9578036	10.806341	10.7717585	10.7411939	9.08086042	9.63923108
5.81534359	6.80677515	6.93226437	6.63691023	6.56882592	5.86685433	6.04777502
8.86693146	9.22751535	9.36442273	9.35354892	9.24879809	8.51426392	9.02337707
6.12543269	6.87838571	7.20505718	6.98756531	6.86256669	6.38982095	6.58338775
13.1941023	14.0684945	13.7953244	13.8828178	13.8019559	12.5122254	13.1255727
8.41128641	9.47973116	9.86375374	9.18268733	8.46348636	7.3343752	8.12067127
9.04504018	9.3468902	9.47489655	9.3056735	9.7353223	8.61932607	9.18562129
5.78323374	6.94173195	6.65115217	6.36809311	6.21498659	5.78183232	6.27398969
10.1393339	10.9380637	10.9038879	10.6839368	10.7062517	9.87856918	9.5787361

6.59520391	7.06686631	6.98733906	6.45852096	7.00268227	5.8629884	6.03568923
14.2458773	14.3141244	14.34987	14.2854051	14.9443049	14.2035575	13.89228
10.3144643	11.6794335	12.1347132	10.7062905	10.7247517	9.98967833	10.1720249
11.1595018	12.0164688	11.6696084	11.4973803	11.8334871	10.6662213	11.1927551
7.72316125	9.33101074	8.47852898	7.90050518	7.84370188	7.41644119	8.50743194
7.62669392	8.46321659	8.67630232	8.5990358	9.15039416	7.57939288	6.61331889
7.06293982	8.07052243	7.90586833	7.93066047	7.85474935	6.93630696	7.0962492
10.5656162	10.8070788	11.2846136	11.5962317	10.9969098	10.4378103	10.8382514
6.22791451	5.88842229	7.21374873	5.79754637	5.88672743	5.66833956	5.40398446
5.88688615	6.83010952	6.69304084	7.18141667	6.76952052	6.15502645	6.41692552
5.5191267	6.41867605	6.18449553	6.05638671	6.15789864	5.99801877	5.71266126
6.09811464	7.39299052	6.87644205	6.71214865	6.49865234	5.93356863	6.85239842
11.7175501	12.57994	12.6602727	12.4847006	12.2940245	11.3742358	12.0915494
7.70347797	8.79507857	8.78401347	9.32696736	9.03319199	7.6862736	8.30147196
7.81169515	8.49554687	8.940747	8.17308845	8.74497306	7.20162014	7.05885005
8.77791511	10.9227375	10.4057574	10.2709564	10.1295677	8.90306389	8.60032142
9.89728264	11.7367985	11.8148608	11.2355326	10.1075022	9.15304934	9.24489633
6.15746922	7.44616358	6.2967439	6.22874906	6.32854946	5.88216028	6.52186909
8.41576458	9.56511122	9.67107749	9.69152073	9.42265539	8.55273057	8.26647923
5.9389223	6.93180005	6.77902985	6.56213449	6.64350716	5.96460864	6.09358595
13.3710619	14.1257573	14.1228426	14.1669845	14.1702866	13.7164926	13.2534464
8.59382471	9.453305	9.18100637	8.88634864	9.34265074	8.10910612	8.13138679
5.4635453	6.21708625	6.23191735	6.09402299	5.94958512	5.58927182	5.55399845
6.49011151	6.95754088	6.94160873	7.15793703	7.36133149	6.70529647	7.12423403
12.4276182	12.9752651	13.1087571	13.1068168	12.7945938	12.3970411	11.8944775
8.13242467	8.92424778	8.99036553	8.90353905	8.79383282	7.68028989	8.3344114
5.7912064	6.02404658	6.394944	7.76585627	6.34330406	5.64397535	5.80515984
5.80699627	6.67821701	6.86321742	5.78773387	5.67045203	6.017628	6.14738832
7.19468792	7.94402034	8.08271696	8.03861451	8.24682656	7.1827489	6.59285272
7.65105169	8.37645666	8.66864251	8.78578527	8.9995784	8.14833218	8.20248335
8.00353422	9.28562369	9.27217113	9.19503872	8.37300301	7.51442845	8.09036123
5.7112314	6.21304452	6.15000638	6.2831882	5.82626399	5.55494181	5.83109819
8.34506632	9.00087155	8.89186723	9.42791976	9.36571134	8.65853962	9.04462963
6.9911023	7.30134346	7.72866543	7.30925624	7.10099446	6.37161427	6.78291305
6.70805201	7.73463715	7.90824069	8.0620904	8.32306377	7.28465848	6.5488404
6.46404136	7.49285382	7.45695806	7.44126684	7.16296729	6.3395748	6.447822
5.81287246	6.43181248	6.34603001	6.48580275	6.07253975	5.57462471	6.08072587
5.66565654	6.3348137	6.14311317	6.38478789	6.25117921	5.52080629	5.56370606
8.90202834	9.93305251	9.83195449	9.66203829	9.33301353	8.78291698	8.69114992
9.21441335	10.424139	10.2727348	10.2394685	10.4324438	9.21262454	8.64984356
5.67389162	6.85535689	7.07118667	7.02062887	6.63875403	5.74602597	5.73721002
5.31457001	6.17199739	6.32627847	6.22167412	6.20115256	5.81706858	6.03440766
6.71579593	7.70615132	7.3220426	7.25135743	7.06269703	6.56926869	6.87329522
5.82701236	7.23772909	6.79590069	6.62624769	6.67068032	6.08473463	6.03413162
5.30229309	6.2395382	6.17241491	6.05518177	5.84381907	5.5874397	5.70841819
11.9184641	12.8606903	12.7734333	12.5993397	12.7298648	11.224021	10.8503119
8.81122921	10.7863472	10.0870239	10.2075439	9.22469439	7.94358382	6.31685641
8.21636319	9.28259595	9.02455461	8.91305245	9.22508425	8.13773456	8.1751528

8.51232685	9.71134502	9.19490899	9.25052132	8.93509844	8.88914191	8.82624371
11.2216143	12.061009	11.777737	11.4988866	11.7906491	10.9236053	11.2740549
5.7487301	6.61355316	6.78784381	6.6170704	6.49049777	5.98692671	5.67091396
8.27089985	9.45189726	9.39647891	8.8702038	8.68846259	7.95809098	8.02819838
6.89681675	8.04827161	8.41777405	8.41174739	7.73393998	6.85359152	6.867684
5.53255791	6.07850779	6.13976989	6.41789132	5.96795224	6.00260802	5.83383303
6.20346488	8.0059776	7.82661077	7.54577768	6.86414776	6.23644459	6.13167817
9.58218697	10.5254004	10.1529971	10.1263947	9.9862616	9.34357527	9.92405519
7.67196205	8.95771266	8.7109383	8.83523375	8.72374967	7.92670398	7.56871467
5.75029832	6.6275689	6.6535201	6.66976664	6.51211277	6.02252184	6.32749031
6.5533006	6.93802697	7.31604887	7.42208496	7.11544739	6.54464318	6.44160299
5.39128621	5.98701315	6.05597021	6.17272972	6.34445748	5.68746611	5.74312931
9.79817772	10.6024725	10.4988243	10.647149	10.6032424	9.60491641	8.78335483
7.94389086	8.73864828	8.72817971	8.57096264	8.78520461	7.70989163	7.77264119
9.2613973	9.46855725	9.94295209	10.1725138	10.6448522	9.04655381	7.7733021
5.75268973	6.50047591	6.77037582	5.6654789	5.89431722	6.38157981	6.246562
5.65309643	6.45957162	6.34712543	6.57810983	6.26812163	5.53332849	5.40016047
9.98956338	10.2644391	11.2428746	11.5485339	11.684451	10.2998502	10.4743428
8.46163415	9.54811059	9.2337359	9.03029276	8.66963343	8.04477551	8.172378
10.7639792	12.1835876	11.7599219	11.570184	11.7320847	10.9202382	10.7984426
5.87629887	6.03701052	6.24396358	6.26360705	5.81445173	5.76483604	5.96052399
5.40675774	5.97580808	6.1013181	6.12701806	5.95107112	5.9189858	5.79600248
13.2965033	14.1373114	13.9747901	13.9463352	14.2905978	12.9264135	13.564783
13.3735014	14.3185944	14.142044	13.9220501	14.267399	13.4575297	13.450041
8.97011855	9.64670468	10.1309592	10.0107309	10.0397471	9.87962766	9.27757684
5.68560718	6.57033951	6.33766347	6.15422471	6.19141499	5.79373855	5.90047342
7.0235703	7.65348428	8.15852335	7.97497435	8.21609589	7.33821937	6.57984524
6.29508179	6.75632681	6.97434231	7.14415959	7.07002431	6.69212586	6.35952223
7.04256664	8.08190201	7.77218523	7.77156936	7.90244506	6.87424737	6.48401516
5.48824409	6.03289891	6.24941216	6.17307005	6.12784723	5.70638744	5.47147005
12.0172883	12.6963503	12.8112021	12.7914645	12.4540476	11.4758464	11.6867286
8.84542858	10.6841122	9.59726727	9.29173454	8.80090119	8.0752882	8.27251782
9.33507208	10.3684792	10.1201562	10.2717361	10.1369168	8.34582302	8.57847761
7.60527282	8.50382614	8.58100959	8.51489273	8.35014758	7.50226058	7.57561412
7.29723636	7.92982058	8.19613587	8.29954919	8.26297852	7.19010418	6.45955523
6.57395993	7.2696812	7.30013013	7.26444917	6.89699592	6.3812877	6.60862771
7.09430153	7.67063501	7.74020171	7.53127852	7.1499696	6.58700052	7.19862478
7.5270805	9.25561309	9.17319441	8.95088129	8.2356098	7.78783924	7.50478007
6.1996537	7.36843924	7.35000654	7.07949438	6.89503597	5.66700791	6.34194466
7.85848274	7.70981308	8.21572373	7.97156195	7.96206774	6.7950409	6.59979445
8.45964309	9.18107024	9.51119938	9.44777258	9.60345814	8.8209869	8.47009226
11.1629187	11.9075982	11.8867685	11.8148248	12.4066188	10.9789778	10.1924321
11.685348	11.9359948	12.1363693	11.7777957	11.8408499	11.2305861	11.3558334
7.92043206	8.78035505	9.40676858	9.26129456	9.72364729	7.38767703	7.47315292
5.52417748	6.22851531	6.41013993	6.36190115	6.19285361	5.70716777	5.85030863
6.25401611	7.68017102	7.21008977	7.29913291	6.99043304	6.23953667	6.40577015
9.48513133	8.8120563	9.51710046	10.3046251	10.777229	9.73655617	8.55748361
8.07387003	8.0062565	8.76855322	8.37372215	7.88575371	7.27596617	7.50347913

6.85018434	8.01429001	8.17722318	8.15650027	8.17128941	7.0448957	6.72053431
5.63225047	6.11869403	6.22182141	5.86577261	5.7506668	5.68539329	6.23069336
6.55737072	7.23256099	7.35073326	7.33535016	7.46807659	6.65145358	7.24825683
8.69229433	9.0291638	9.25406653	9.03836786	9.08954847	8.08314155	8.90085564
7.33309719	9.39376026	7.91225046	7.91575571	7.51087491	7.05807076	7.50675505
10.9214029	12.0222981	11.9188305	11.6886345	11.7088946	10.9177965	10.9554363
6.19444977	6.98546969	7.13778527	5.90442942	6.22012755	7.04792222	7.37308613
7.20579032	8.5634476	7.87911146	7.91711724	7.24716705	7.03457119	6.7446307
11.2214379	12.8467824	12.4753398	12.4044066	11.9207217	10.3326196	11.0664761
11.0861103	12.801263	12.5782404	12.2948984	12.555663	11.4986667	11.6585699
6.58335345	6.18219132	7.26488543	7.10394028	6.76232755	6.68825311	7.15376886
6.22354747	6.90638677	7.26492764	6.83671189	6.54579682	6.21275336	6.46477134
6.30773834	7.72791977	7.75694655	7.16780449	6.81361704	5.93298973	6.30468078
5.45724362	6.27418878	6.85303253	5.41463991	5.73554222	5.86225871	5.82256344
10.2091353	10.9605313	10.7166437	10.4340558	11.0627842	9.88404991	10.0272301
9.35715917	9.58162128	10.7953709	10.7075662	10.5115016	9.8997578	9.85010915
8.1822086	9.75180295	9.61087203	9.64327431	9.9380127	8.04383551	7.55237015
7.28813612	9.05310156	9.22135463	9.75059897	9.53340255	8.30853198	8.88743419
7.82283338	8.99474122	8.92845412	8.77009661	9.04562289	7.92311708	7.83772316
12.433654	13.2665722	13.1216758	12.7057898	13.1842876	11.9724568	12.3054396
6.39341208	6.74203721	6.87403461	7.00504623	6.94444385	6.23925864	6.0910073
5.80340142	6.76353109	6.56109163	6.53907225	6.61037614	5.77131488	5.60560041
6.31045592	6.60414666	6.60227292	6.37825306	7.21266174	5.91486729	6.1906115
5.58213385	6.45483144	6.41307427	6.08821009	6.0942196	6.01591231	6.197012
5.68186301	6.30699055	6.55331519	6.56603927	6.54691313	5.94189178	6.12517249
7.12202452	8.10906746	8.28718737	8.00721289	7.82023833	7.30638256	7.35913019
7.73534819	8.83088847	9.12323034	9.2519846	8.81714484	7.23939804	6.61587413
6.33396858	7.76888234	7.64070551	7.38110424	7.14955813	6.12725295	6.25099757
11.9590083	12.7619237	12.4809078	12.5677731	12.5551367	11.4142148	11.5705644
7.49357929	8.21871531	8.02375934	8.60418848	8.2173805	7.71693741	7.90655994
7.8589607	8.90612998	8.02237281	7.70715963	5.55713465	7.3184226	6.85558986
6.2605453	6.64487437	7.80477315	7.05281473	7.32822253	7.29539243	7.64381435
8.80009099	9.63364554	9.42907381	9.04912811	9.26310274	8.55200811	7.52527414
7.16525977	7.14730365	7.80127767	8.06557608	8.00799603	7.31728869	7.10101233
7.24611351	8.98339439	8.94190328	8.73196148	8.33623473	7.16392335	6.87024757
7.81573936	9.0876437	9.10316924	8.83366399	8.86999876	7.82964537	8.10663622
6.23736046	5.98527977	6.7196941	6.82687385	6.26265753	5.97576063	5.80895105
6.42692036	7.28050633	7.04298352	7.21112222	6.85356282	6.09210614	6.09465687
8.66378472	10.7157553	10.5483885	10.3480288	10.1577953	8.41979547	8.45184741
8.98175421	8.35849473	9.58620404	8.13113106	8.37249291	6.88794803	7.90956552
6.91586385	7.92504225	8.7016693	8.34058185	8.40704848	6.39130546	7.09817003
6.25378169	7.03094848	6.99755436	7.04301963	7.01034236	6.1792003	6.08075913
8.17797159	9.13553256	9.22443442	9.17732336	9.62716158	8.62802187	7.86109746
8.40917664	9.73991509	10.0868262	9.82154042	9.78217838	8.27861347	8.9164223
7.63822665	8.37802689	8.88537608	8.65997271	7.95519247	7.5058822	7.43414864
5.87267759	6.49133684	6.65597858	6.81517448	6.40608627	6.32481582	5.93302279
5.49771965	6.16578133	6.05961659	6.09073003	5.95705606	5.61138576	5.82089911
6.9968463	7.33795188	7.78813624	7.65978331	7.82983434	6.95157731	6.74239029

7.48922821	8.0528533	8.1859146	8.15699115	7.72157294	7.50357136	7.35792055
10.2503813	11.8956181	11.0540019	11.0780487	10.4078912	10.3101288	10.3793762
5.49065965	5.85801632	5.97958525	6.00691129	5.67737801	5.68692963	5.51539802
9.3000553	9.82434002	10.0704913	9.77486842	10.3809956	9.12494843	9.40769477
6.89796038	8.06434668	7.74998538	7.85135428	8.31450258	7.38716606	6.98184695
6.41665551	8.18873521	8.14680896	6.75190954	6.94771407	6.1260587	6.59950898
6.86055813	7.57262065	7.5895021	7.83533925	8.20236336	6.97243189	6.61850693
5.53234533	6.51863726	6.57319848	6.53682969	6.28947153	5.70187809	5.7584948
7.9970592	8.9476121	9.13341916	9.07016362	9.02099688	7.80184737	7.34491014
5.72963189	6.06311568	6.37450658	7.9916851	5.88484258	5.80436855	6.05417363
7.86460156	8.7487071	8.85018871	9.09074098	8.39831745	7.86964072	7.07367566
6.14534283	7.16879545	7.05680135	7.06751396	6.68538796	6.21145187	6.50165713
5.85016508	7.3990805	6.85684256	7.42887769	8.43273439	6.39997034	6.52546866
7.0040103	7.94601787	7.49831789	7.02890062	7.0281304	6.73529877	6.90336973
11.2245022	12.398445	12.3135732	11.5370315	10.8583657	9.22710688	10.6641797
7.50228923	8.83567085	9.30355908	7.25864329	7.96195202	7.4173901	6.39493543
10.5479462	11.6767113	11.5644782	11.3932537	11.6856638	10.2211883	9.89248635
5.47683918	6.17791859	6.54218034	5.67016335	5.82907753	6.18712736	5.88740098
7.37037791	8.58210773	8.4412548	8.64003846	8.56082269	7.36010788	7.4915974
13.275034	14.1668127	13.9115933	13.3673956	13.3803961	12.7387674	13.0640116
6.75662971	7.75311644	8.35739761	8.49803141	8.66682546	7.29265661	7.36340064
11.7098562	13.0531553	12.7352338	12.3149675	12.2987222	11.2031479	11.9999172
9.16954374	9.47168578	9.84858679	9.15231907	9.286119	8.49714648	8.49635848
11.4074261	12.4762814	12.5874149	12.1311399	11.6737172	10.5053392	10.6551434
9.10102179	9.80804905	10.1278461	9.5106726	9.2291322	7.85090316	8.78336269
7.8761418	8.97296285	8.40028431	8.80392735	9.09027521	7.70465828	7.87937372
6.64988989	7.05600902	7.70407032	7.74982519	7.41185753	6.51957068	7.16474606
9.31008173	10.4729863	9.82362439	9.79148461	9.47020922	8.73116763	9.21135832
5.33442025	5.55980626	5.78452178	5.85739783	5.67120391	5.29359672	5.35388501
12.4357166	13.1338878	13.4188435	13.1599716	12.1388628	11.6730007	11.0766479
6.33038684	6.44247264	7.02050109	6.95335022	6.74977425	6.14061998	6.08980405
5.81544504	6.29654312	6.38557634	6.61427697	6.44981572	5.81501459	5.93445399
11.3951924	12.6060415	12.3976196	12.1560056	11.8468482	11.0343244	10.4875024
6.78826939	7.11057309	7.44201954	7.55344806	7.3203547	6.69627487	6.27143091
8.44826418	9.99295674	10.0989795	9.64935319	9.09085264	8.16191488	8.54102225
8.26859908	8.89041812	8.83332687	9.0110813	9.18191851	8.8850374	8.68425595
6.29799546	6.81761814	7.14075208	6.94633786	6.73797062	6.44464968	6.18491025
8.87310262	10.032842	9.79096503	10.0452327	9.43819345	8.93655432	8.69210214
6.25700156	7.16672448	7.2914553	7.17600156	7.22919086	6.40430829	6.36970866
12.4719858	13.4726997	12.924568	12.8021433	13.1069129	12.0634369	12.4504734
7.7280933	8.29820786	8.47059909	8.79335328	8.25248851	7.28229801	8.04090339
5.71483484	6.15537564	6.27664517	6.04408959	6.24495098	5.69852938	5.49688253
8.19905833	8.56150295	8.67250455	8.4799714	8.07939838	7.54411054	7.25939679
5.83123554	6.91111631	6.83058999	6.42481748	6.46027387	5.82631154	6.32106511
12.1286114	12.882358	12.9747385	12.2569676	12.230756	11.4682554	11.6306544
5.96482417	7.39267118	7.06009279	6.639121	6.82191553	6.01276799	6.50756252
7.04318372	8.81550594	8.6964192	8.58777639	8.40856879	6.97867958	7.08826353
7.34626462	8.26722912	8.39963938	7.99037801	8.04357691	7.00944795	6.87093552

6.53234767	7.73626797	7.70442451	7.84298951	7.34301778	6.3751724	6.46604203
5.63644018	6.39318913	6.60288545	6.17561661	6.74845721	5.91937012	5.99800635
5.4912836	6.53899623	6.69585605	6.3499717	6.19292243	5.67871421	6.20649984
6.27403294	7.27586284	7.46824199	7.27939886	6.76251094	6.45488423	6.75670042
9.93007585	11.1320916	10.8472477	10.5981623	10.3498175	9.37421704	9.61660269
8.47462302	8.65086004	8.63267392	8.99214867	8.49650749	7.96944984	8.2128441
11.3627603	12.2579644	11.9186199	11.576653	12.1180381	10.9638967	11.4420198
7.7286287	8.26180628	8.2275346	8.36517722	8.31514684	8.03788734	7.7330933
6.0761687	7.38984125	7.85523478	7.79703633	7.00954873	6.79694032	7.03550625
7.37987651	8.19334301	9.10280744	8.40429475	8.93134533	7.39918562	8.44402056
6.84058408	7.53835813	7.63115522	7.65868992	7.78848996	7.39364787	7.10359625
11.2159873	11.9483318	11.7070882	11.3194158	12.0971644	10.8872686	10.9727673
5.87068627	6.20888124	6.74459034	6.24620835	6.23612233	6.05341071	6.06061661
12.9708799	13.7604923	13.4898191	13.323871	13.7332505	12.3744475	13.1299672
6.31961956	6.48109014	6.9867727	6.99398564	6.98268904	6.2312955	6.37167612
9.10490718	10.069435	9.83587024	9.58589526	10.1437327	8.74414125	8.78685933
8.06114434	9.00136175	8.91256963	8.88343265	9.11146722	8.08738752	7.18373263
5.78773413	6.18178656	6.03744394	6.44282211	6.01151375	5.86635215	5.93357028
7.26548753	8.1250275	8.7015643	8.10916464	8.19066424	6.67140519	8.08509637
5.33794332	6.0364985	6.2968918	5.89467979	6.33880839	5.71806484	5.75649494
6.46846863	7.83461579	7.48808113	7.16666223	6.98207635	5.99762301	6.82971766
5.21142422	5.99704215	5.89771237	6.04171522	5.91018291	5.5151157	5.76255771
5.38511658	6.14488355	6.1591165	6.24441255	5.98831226	5.99159463	5.78718508
5.65730873	6.46178997	6.32503556	6.06394595	6.3193486	5.94436724	6.06540515
5.42655394	6.46860906	6.48180605	6.24612207	6.14139968	5.70567772	5.88266603
5.21752117	5.6835446	5.85460039	5.75092829	5.65450657	5.60218308	5.42818495
5.99585283	6.64818355	6.83680276	6.58166097	6.2631287	5.79125381	6.17409937
6.80446406	7.93060725	7.94293201	7.35513787	7.78145086	6.41525721	7.22613567
5.53541318	6.30088826	6.27910816	5.96897753	6.33913293	5.6692998	6.13224574
5.75660409	6.94410177	7.19326513	7.04010009	6.70292484	5.75158583	6.10809556
5.18138963	5.79713104	6.02431835	5.87141393	5.65358774	5.48069102	5.62563901
5.62239129	6.00615819	6.22707332	5.96853594	5.83705339	5.72518873	5.84691749
6.03098973	7.06185503	6.89738203	6.7050144	6.90290205	6.26222398	6.40654347
5.32317981	5.82297279	5.86421339	5.77723901	5.7496965	5.61301737	5.56821638
6.44664084	7.72043512	7.77554707	7.30887687	7.28722016	6.29297403	6.77191594
5.59655773	6.66828651	6.44867915	6.29367819	6.23881934	5.53871787	5.74515879
6.19511547	7.44634888	7.50264885	7.09386754	7.25398832	6.03014668	6.87667656
5.72020595	6.31610337	6.46224983	6.16307882	6.53715704	5.76971753	6.01044687
5.47884823	5.90797437	6.12868005	6.1996537	5.84277297	5.73446305	5.73339366
7.15009861	7.79174506	8.17808168	7.96583507	7.6090073	6.56692229	6.72099799
6.70968765	7.8246024	7.35044774	7.27210159	6.534063	5.67221911	6.1669526
6.17642431	7.29976758	7.17392593	6.90531118	7.00100503	6.00919218	6.82684589
6.78001213	7.16021431	7.56895326	7.36293271	6.93964033	6.14971461	6.94535604
5.48257856	6.28417022	6.47653415	6.16150439	6.19894375	5.70045137	5.9715224
5.02010345	5.46591147	5.73358646	5.52527524	5.53284026	5.37666893	5.45501119
5.55025549	6.38796199	6.4285796	6.52832595	6.40295457	5.87383214	6.19552379
5.30445482	5.46123681	5.62142305	5.75460214	5.50616681	5.35323859	6.07824476
6.05708999	7.92121911	6.71220507	6.4850434	6.05149348	5.86952451	6.24746103

5.13620246	6.47301423	5.96882827	5.82408987	5.87578301	5.26771594	5.48226199
5.87907813	6.46637148	6.45722047	6.44084974	6.28336732	5.83005246	5.81585846
5.86727429	6.36463826	6.42888004	6.23049298	6.62721241	6.11615887	6.14564913
5.56798101	6.0509733	6.15421944	6.08737925	6.11077268	5.39193815	5.45818348
5.96534841	6.61609579	6.66651802	6.6422282	6.86072076	6.25432986	6.07823494
6.84531692	8.26787799	8.10084838	7.57222272	6.55093529	5.98701337	6.54846452
7.32259699	8.82259824	8.72058014	8.58538661	8.37934414	7.02637626	7.39457845
6.28058353	7.70651605	7.74650156	7.66732802	7.16110265	6.78900929	6.48501215
5.2162263	5.69029305	5.94759837	5.75721824	5.72182213	5.68648932	5.58187032
6.50262769	6.64227326	7.48953491	7.56366762	7.39216885	6.19410301	6.62881631
5.44372267	6.55150787	6.55438502	6.64485563	6.25683329	5.68480107	5.74442512
6.03050581	7.34987212	6.98353292	6.69002871	6.5485758	5.60518584	6.55299133
7.11956576	8.37369562	8.77267219	8.33394783	8.0898122	6.86048488	8.27280845
5.39419445	6.05591795	6.10644736	6.04704362	6.02444668	5.65036018	5.83091141
5.78317715	7.01804213	7.24490323	6.86162045	6.53959838	5.94382336	6.23325901
6.28576044	7.37488994	6.55344929	6.98042409	6.36008505	5.79484809	6.5545511
5.33498617	6.02473455	6.2586497	5.95925409	5.92404334	5.55036171	5.67647814
5.98488893	6.91360632	7.10266758	6.36200622	6.29731231	5.78613499	6.24033837
5.40742958	6.18587447	6.30281327	6.28598787	6.13313831	5.64233623	5.7288443
5.08810131	5.67927008	5.72534472	5.64662754	5.61556696	5.23792886	5.34020511
5.15506957	5.73611939	5.85757027	5.82025888	5.61273347	5.46756414	5.55561219
6.35550545	6.95465348	7.26267463	6.94505751	6.62762668	5.93676324	6.15910075
5.27569842	5.56514962	6.07392432	5.74528063	5.53207192	5.28628384	5.39419445
5.61027603	6.50250899	6.54300413	6.28810843	5.95612056	5.61252914	6.30086685
5.14650453	5.98759214	6.11233316	5.96626364	5.70222201	5.48022052	5.41536353
5.03411885	5.84224599	5.68208389	5.4602357	5.6979567	5.51461465	5.28542478
6.2651081	7.09019501	7.67450161	7.24919406	7.50496275	6.2968274	6.16824709
5.44723369	6.68127215	6.64222936	6.16907937	6.58471271	5.62619205	6.20372445
5.26815102	6.22246977	6.34776362	6.64928349	5.88157041	5.84318994	5.82921989
5.77791307	7.31675488	7.38762534	7.3964772	7.18244997	6.04878307	6.46411098
5.96721288	7.16333368	7.16210528	6.84662745	6.52048825	5.93033191	6.44709629
5.81777443	6.31534686	6.32943938	6.25778477	6.34725904	5.82781496	6.31123916
7.45917175	8.38284879	8.57727133	8.45751919	8.0911365	7.73203511	7.23148905
5.79874991	6.95776953	6.51562999	5.95386399	5.91772294	5.55910539	5.86826969
6.26590267	6.86993555	6.8828411	6.70809341	6.73875028	6.27949024	6.61321293
5.51287665	6.45228161	6.86284933	6.24605763	6.1016423	5.58283777	5.74890235
5.59833662	6.73978736	6.62344083	6.41703864	5.8323546	5.55631721	5.83318131
7.60219704	9.27946178	9.03151402	9.02994423	8.72317046	7.44847812	7.26699825
6.65277472	7.98291887	7.21431621	7.38385719	6.86332399	6.06497056	6.12711384
6.75751002	6.67658477	6.62552051	6.83966361	6.17665089	6.19766	5.95070915
5.37281492	6.00622306	6.03430465	6.13663355	5.67982602	5.53794302	5.73553626
5.54337803	6.99391207	7.06486884	6.40849721	6.32533658	5.53982106	5.72435887
12.118375	12.2734815	12.6659734	12.1092917	11.7503059	11.6055573	11.8265548

C194_NS	C005_NS	C053_NS	Species	Source	Search_Key	Transcript
13.2013326	13.7033272	13.6871515	Homo sapiens	RefSeq	ILMN_35460	ILMN_35460
6.97829861	8.45249907	7.96239401	Homo sapiens	RefSeq	ILMN_33714	ILMN_33714
6.88444773	7.57270098	7.59455676	Homo sapiens	RefSeq	ILMN_35526	ILMN_35526
6.23306818	8.60294192	7.73543485	Homo sapiens	RefSeq	ILMN_39212	ILMN_39212
7.14543189	7.30868305	7.14318217	Homo sapiens	RefSeq	ILMN_36365	ILMN_36365
6.57540885	8.0743422	9.0137146	Homo sapiens	RefSeq	ILMN_36463	ILMN_36463
5.95923577	7.54389486	7.70063461	Homo sapiens	RefSeq	ILMN_33930	ILMN_33930
5.9235457	6.58003978	6.1876773	Homo sapiens	RefSeq	ILMN_35501	ILMN_35501
6.67471063	7.97035039	7.75828319	Homo sapiens	RefSeq	ILMN_45221	ILMN_45221
6.5657245	6.76916079	6.68868672	Homo sapiens	RefSeq	ILMN_37061	ILMN_37061
6.73432485	7.43161878	7.65849428	Homo sapiens	RefSeq	ILMN_35497	ILMN_35497
7.35896234	8.36038623	8.59716837	Homo sapiens	RefSeq	ILMN_34363	ILMN_165204
6.1359961	7.64765053	7.63820709	Homo sapiens	RefSeq	ILMN_25861	ILMN_25861
7.05584964	7.85984162	8.14142666	Homo sapiens	Unigene	ILMN_123613	ILMN_123613
6.3678297	7.66356807	8.88187657	Homo sapiens	Unigene	ILMN_85632	ILMN_85632
6.51350527	7.90703486	8.30621256	Homo sapiens	Unigene	ILMN_102473	ILMN_102473
7.87907469	7.46649498	7.67458282	Homo sapiens	Unigene	ILMN_95916	ILMN_95916
7.92989868	8.36866666	7.95208657	Homo sapiens	Unigene	ILMN_110121	ILMN_110121
5.3985662	5.79797817	5.65262875	Homo sapiens	Unigene	ILMN_84001	ILMN_84001
6.0979828	6.67077095	6.98592389	Homo sapiens	Unigene	ILMN_122060	ILMN_122060
6.54328104	7.40899881	5.92728043	Homo sapiens	Unigene	ILMN_71317	ILMN_71317
5.42073603	6.15489627	6.23717649	Homo sapiens	Unigene	ILMN_115289	ILMN_115289
7.45722244	9.00949834	9.1704241	Homo sapiens	Unigene	ILMN_92999	ILMN_92999
11.4964983	12.6740022	13.0815563	Homo sapiens	ILMN_Controls		ILMN_160456
10.5280106	10.8605183	10.800142	Homo sapiens	RefSeq	ILMN_992	ILMN_992
5.51158126	5.88647211	6.21320378	Homo sapiens	RefSeq	ILMN_4884	ILMN_4884
13.3059392	13.7497385	13.5994631	Homo sapiens	RefSeq	ILMN_3511	ILMN_3511
7.2764064	7.68685976	8.97319309	Homo sapiens	RefSeq	ILMN_438	ILMN_438
5.83829454	7.58847029	7.82470231	Homo sapiens	RefSeq	ILMN_32918	ILMN_34149
8.21161725	10.1118803	9.62201008	Homo sapiens	RefSeq	ILMN_6684	ILMN_6684
13.0516547	13.8317842	13.5107234	Homo sapiens	RefSeq	ILMN_37525	ILMN_32190
12.3905514	13.0685128	13.1960162	Homo sapiens	RefSeq	ILMN_12810	ILMN_12810
5.67178278	6.36101612	6.3061957	Homo sapiens	RefSeq	ILMN_4020	ILMN_181875
7.18610532	8.3874724	8.84073985	Homo sapiens	RefSeq	ILMN_21452	ILMN_170581
5.09489645	5.29867628	5.29161719	Homo sapiens	RefSeq	ILMN_16049	ILMN_32013
11.0318783	13.3454704	13.3168133	Homo sapiens	RefSeq	ILMN_21166	ILMN_170702
8.63973177	9.66964672	9.89129827	Homo sapiens	RefSeq	ILMN_30796	ILMN_30796
9.01371767	9.5108668	10.3206844	Homo sapiens	RefSeq	ILMN_9004	ILMN_9004
5.96335667	6.16245196	6.50413412	Homo sapiens	RefSeq	ILMN_24167	ILMN_180500
6.37378844	7.05222024	7.17981855	Homo sapiens	RefSeq	ILMN_1648	ILMN_10378
8.5529457	9.71155066	9.85661103	Homo sapiens	RefSeq	ILMN_16610	ILMN_176850
7.52773815	8.60823375	8.77688041	Homo sapiens	RefSeq	ILMN_24367	ILMN_162780
6.44250896	6.99449261	6.79123323	Homo sapiens	RefSeq	ILMN_4217	ILMN_4217
6.95903982	7.93297592	8.57604098	Homo sapiens	RefSeq	ILMN_13023	ILMN_13023
5.8965901	6.24850144	6.88804547	Homo sapiens	RefSeq	ILMN_19660	ILMN_182039
7.85970374	9.70263642	9.3846467	Homo sapiens	RefSeq	ILMN_13156	ILMN_13156
6.95603225	8.0978025	7.74167394	Homo sapiens	RefSeq	ILMN_37703	ILMN_181618

6.61777849	7.56945843	7.6978711	Homo sapiens	RefSeq	ILMN_39093	ILMN_39093
7.26726986	6.64124327	6.25442114	Homo sapiens	RefSeq	ILMN_42161	ILMN_176099
9.87266534	11.3347962	10.6744942	Homo sapiens	RefSeq	ILMN_26733	ILMN_26733
9.36158613	11.1143488	11.1275823	Homo sapiens	RefSeq	ILMN_8122	ILMN_8122
6.75068957	7.89519112	7.46219468	Homo sapiens	RefSeq	ILMN_30967	ILMN_30967
6.37035123	6.95239298	6.77246238	Homo sapiens	RefSeq	ILMN_138909	ILMN_138909
13.5013521	13.5853663	13.610184	Homo sapiens	RefSeq	ILMN_33193	ILMN_5932
6.15300251	7.08614366	8.03709664	Homo sapiens	RefSeq	ILMN_2573	ILMN_2573
5.78283523	6.03536218	5.85858978	Homo sapiens	RefSeq	ILMN_45580	ILMN_45580
8.74124315	10.257211	9.68798938	Homo sapiens	RefSeq	ILMN_137685	ILMN_7619
5.64326254	5.87505337	6.67053162	Homo sapiens	RefSeq	ILMN_31490	ILMN_31490
6.83528492	7.96934853	8.01523551	Homo sapiens	RefSeq	ILMN_15612	ILMN_15612
8.592966	10.2916968	10.1755982	Homo sapiens	RefSeq	ILMN_23821	ILMN_23821
6.99648366	8.66379646	8.71873356	Homo sapiens	RefSeq	ILMN_931	ILMN_931
6.56549969	8.09396205	7.76350254	Homo sapiens	RefSeq	ILMN_19186	ILMN_19186
9.33957079	10.0513232	10.7808096	Homo sapiens	RefSeq	ILMN_28738	ILMN_28738
6.58819187	7.52174097	8.37849342	Homo sapiens	RefSeq	ILMN_12534	ILMN_12534
5.55153417	6.70577337	7.04582845	Homo sapiens	RefSeq	ILMN_9310	ILMN_9310
6.15589654	7.45540285	7.39284889	Homo sapiens	RefSeq	ILMN_18159	ILMN_168272
8.81185728	10.2973462	9.78694115	Homo sapiens	RefSeq	ILMN_9157	ILMN_9157
6.05853254	6.72349167	6.99254413	Homo sapiens	RefSeq	ILMN_23073	ILMN_40991
11.0395456	12.1974824	12.0420383	Homo sapiens	RefSeq	ILMN_22938	ILMN_22938
6.98046293	8.57382401	9.06376986	Homo sapiens	RefSeq	ILMN_674	ILMN_5512
8.38907502	8.91145341	9.17598685	Homo sapiens	RefSeq	ILMN_3697	ILMN_3697
7.89018522	9.91679713	9.2526801	Homo sapiens	RefSeq	ILMN_12451	ILMN_12451
8.22729055	8.60421851	8.81140424	Homo sapiens	RefSeq	ILMN_1064	ILMN_1064
6.1801789	7.3295802	7.69447699	Homo sapiens	RefSeq	ILMN_9667	ILMN_9667
7.1827638	9.20315832	8.78699978	Homo sapiens	RefSeq	ILMN_11812	ILMN_11812
8.59293723	9.3848185	9.7848007	Homo sapiens	RefSeq	ILMN_11650	ILMN_167824
5.78694497	6.88704998	6.19907257	Homo sapiens	RefSeq	ILMN_434	ILMN_168083
8.07353589	8.23368151	8.75077163	Homo sapiens	RefSeq	ILMN_23233	ILMN_23233
7.33198999	8.82990536	8.62192842	Homo sapiens	RefSeq	ILMN_18271	ILMN_16784
8.24156708	9.72827224	10.5393769	Homo sapiens	RefSeq	ILMN_23042	ILMN_23042
6.37162316	6.66353533	6.85711512	Homo sapiens	RefSeq	ILMN_42138	ILMN_167196
8.39300082	9.19053264	9.33043314	Homo sapiens	RefSeq	ILMN_29466	ILMN_29466
5.93021787	5.86614416	5.64084243	Homo sapiens	RefSeq	ILMN_42457	ILMN_42457
6.41053446	7.89942218	7.41171011	Homo sapiens	RefSeq	ILMN_18158	ILMN_18158
7.07867708	7.40920677	7.53448498	Homo sapiens	RefSeq	ILMN_3430	ILMN_3430
12.058417	13.8808577	13.803895	Homo sapiens	RefSeq	ILMN_4451	ILMN_4451
9.98269274	10.6068308	10.5793716	Homo sapiens	RefSeq	ILMN_18757	ILMN_18757
6.65818577	7.71403666	5.50190914	Homo sapiens	RefSeq	ILMN_18302	ILMN_18302
7.02886195	9.60633451	9.14318217	Homo sapiens	RefSeq	ILMN_16228	ILMN_16228
6.54405998	7.33254773	7.05249306	Homo sapiens	RefSeq	ILMN_6400	ILMN_169896
8.25275767	9.40656877	10.7748859	Homo sapiens	RefSeq	ILMN_13735	ILMN_13735
6.58032792	8.5692769	8.31318371	Homo sapiens	RefSeq	ILMN_1026	ILMN_1026
5.71885587	7.1485437	7.68414758	Homo sapiens	RefSeq	ILMN_4100	ILMN_4100
8.05628113	9.50236502	9.42255743	Homo sapiens	RefSeq	ILMN_26470	ILMN_176188
5.6157944	6.56173044	6.55717647	Homo sapiens	RefSeq	ILMN_34866	ILMN_34866

7.33669043	7.4510454	8.30412677	Homo sapiens	RefSeq		ILMN_28428	ILMN_28428
7.19393433	8.86731118	9.22375292	Homo sapiens	RefSeq		ILMN_12219	ILMN_12219
7.74845385	8.05346382	7.27347673	Homo sapiens	RefSeq		ILMN_18016	ILMN_18016
8.54523209	9.37628257	9.80419557	Homo sapiens	RefSeq		ILMN_12074	ILMN_182200
11.1098953	13.1593089	13.0591104	Homo sapiens	RefSeq		ILMN_546	ILMN_546
5.9543208	6.23234955	6.02375835	Homo sapiens	RefSeq		ILMN_46756	ILMN_46756
7.9089966	8.89306607	9.05350455	Homo sapiens	RefSeq		ILMN_13904	ILMN_13904
5.89176742	6.27076327	5.98060985	Homo sapiens	RefSeq		ILMN_3397	ILMN_180235
7.53171675	10.4684387	11.0236885	Homo sapiens	RefSeq		ILMN_30135	ILMN_183098
8.61368524	11.7623218	10.4859621	Homo sapiens	RefSeq		ILMN_43918	ILMN_166057
6.61669792	8.77562378	9.03705325	Homo sapiens	RefSeq		ILMN_29986	ILMN_176524
10.5027175	11.3410089	11.5803401	Homo sapiens	RefSeq		ILMN_21668	ILMN_21668
10.7587046	11.6819385	11.9170069	Homo sapiens	RefSeq		ILMN_7361	ILMN_7361
6.24310999	6.61516378	6.63826604	Homo sapiens	RefSeq		ILMN_9777	ILMN_9777
5.94785758	7.51115964	7.68777318	Homo sapiens	RefSeq		ILMN_18165	ILMN_18523
8.05025843	9.89837132	9.7578245	Homo sapiens	RefSeq		ILMN_21987	ILMN_21987
5.60015853	5.71188416	5.9010095	Homo sapiens	RefSeq		ILMN_3184	ILMN_177243
12.4208276	13.977457	14.0092469	Homo sapiens	RefSeq		ILMN_16565	ILMN_24337
5.99186646	5.68470431	6.23732834	Homo sapiens	RefSeq		ILMN_23096	ILMN_23096
9.82680618	10.7189994	11.500086	Homo sapiens	RefSeq		ILMN_33374	ILMN_33374
9.31364697	10.8623468	11.1564326	Homo sapiens	RefSeq		ILMN_24018	ILMN_172961
10.7675265	12.0209515	12.5856924	Homo sapiens	RefSeq		ILMN_16294	ILMN_16294
6.59180222	6.96934277	7.85902595	Homo sapiens	RefSeq		ILMN_24835	ILMN_24835
7.10042371	8.70478345	7.97697017	Homo sapiens	RefSeq		ILMN_30669	ILMN_30669
6.06640483	6.60293398	6.71551179	Homo sapiens	RefSeq		ILMN_17938	ILMN_17938
9.82125611	11.3611976	11.2427835	Homo sapiens	RefSeq		ILMN_19052	ILMN_19052
9.61553916	11.4276104	11.3679694	Homo sapiens	RefSeq		ILMN_6282	ILMN_28535
7.46506352	8.4672807	8.60188405	Homo sapiens	RefSeq		ILMN_7500	ILMN_7500
7.32361596	9.99077054	9.74347474	Homo sapiens	RefSeq		ILMN_12195	ILMN_178116
9.60201159	11.7378735	11.1299115	Homo sapiens	RefSeq		ILMN_2086	ILMN_2086
5.88305819	7.03134336	6.61285258	Homo sapiens	RefSeq		ILMN_4671	ILMN_174333
7.61796108	8.50700808	9.06970915	Homo sapiens	RefSeq		ILMN_26236	ILMN_26236
9.19515637	12.5211138	12.6482079	Homo sapiens	RefSeq		ILMN_12367	ILMN_172296
6.44297119	7.52554508	7.23128743	Homo sapiens	RefSeq		ILMN_16615	ILMN_16615
6.43213146	8.35778906	8.5791726	Homo sapiens	RefSeq		ILMN_17968	ILMN_174251
11.9098938	12.5487097	12.7231366	Homo sapiens	RefSeq		ILMN_27890	ILMN_163725
6.04132688	8.52091721	8.25175317	Homo sapiens	RefSeq		ILMN_4882	ILMN_182705
7.14457362	7.89796401	8.25343482	Homo sapiens	RefSeq		ILMN_17467	ILMN_167454
10.9945387	12.6764523	12.2374203	Homo sapiens	RefSeq		ILMN_11399	ILMN_11399
6.71827531	8.21878407	7.71209361	Homo sapiens	RefSeq		ILMN_25109	ILMN_25109
5.86776355	7.00953977	6.86171969	Homo sapiens	RefSeq		ILMN_18271	ILMN_18271
5.89955149	7.16759176	6.85556868	Homo sapiens	RefSeq		ILMN_24867	ILMN_24867
10.3311409	11.5159974	11.0492462	Homo sapiens	RefSeq		ILMN_9411	ILMN_9411
13.4888494	14.4166432	14.0007448	Homo sapiens	RefSeq		ILMN_34885	ILMN_33374
7.41048527	8.40576947	8.43172825	Homo sapiens	RefSeq		ILMN_137656	ILMN_137656
10.161603	10.4182701	11.6562872	Homo sapiens	RefSeq		ILMN_19677	ILMN_19677
6.0518058	6.76850856	6.74656269	Homo sapiens	RefSeq		ILMN_27829	ILMN_27829
6.28464331	6.95589513	7.13727809	Homo sapiens	RefSeq		ILMN_11654	ILMN_11654

6.62917562	6.99823274	6.81139268	Homo sapiens	RefSeq	ILMN_21696	ILMN_21696
8.31725341	9.08678244	9.29944158	Homo sapiens	RefSeq	ILMN_6355	ILMN_6355
10.2961993	12.812851	11.5107879	Homo sapiens	RefSeq	ILMN_11054	ILMN_11054
13.2987113	14.1669766	14.0217573	Homo sapiens	RefSeq	ILMN_137940	ILMN_162123
8.5334006	10.0293397	10.2024275	Homo sapiens	RefSeq	ILMN_24538	ILMN_24538
6.66568838	8.2883198	7.73010394	Homo sapiens	RefSeq	ILMN_30799	ILMN_30799
5.84348243	6.27153642	7.00152192	Homo sapiens	RefSeq	ILMN_18742	ILMN_18742
10.5140867	12.0814958	12.1282	Homo sapiens	RefSeq	ILMN_20742	ILMN_183744
9.63242673	10.4244991	10.974454	Homo sapiens	RefSeq	ILMN_13829	ILMN_13829
6.43115575	6.5544719	7.10233479	Homo sapiens	RefSeq	ILMN_8242	ILMN_8242
9.95980521	11.5640085	11.7285864	Homo sapiens	RefSeq	ILMN_25184	ILMN_25184
7.29108872	8.45378711	8.6613887	Homo sapiens	RefSeq	ILMN_17355	ILMN_17355
8.59174224	9.16846278	9.4800114	Homo sapiens	RefSeq	ILMN_12918	ILMN_12918
5.94053795	5.92179168	6.30909595	Homo sapiens	RefSeq	ILMN_11187	ILMN_11187
13.7644507	14.2664381	13.9947565	Homo sapiens	RefSeq	ILMN_15031	ILMN_15150
6.54745106	7.71437879	7.96813281	Homo sapiens	RefSeq	ILMN_41055	ILMN_41055
6.49966893	7.24510581	7.51384838	Homo sapiens	RefSeq	ILMN_30159	ILMN_30159
6.16120164	7.76669189	7.87278691	Homo sapiens	RefSeq	ILMN_24062	ILMN_24062
7.6159768	10.0048763	9.60981645	Homo sapiens	RefSeq	ILMN_40016	ILMN_183964
5.77211554	5.90277594	6.46223641	Homo sapiens	RefSeq	ILMN_21688	ILMN_21688
10.0907635	11.8546791	12.1563928	Homo sapiens	RefSeq	ILMN_22627	ILMN_22627
8.77262733	9.70064813	9.85619922	Homo sapiens	RefSeq	ILMN_26395	ILMN_26395
6.92745088	8.20719934	7.74136519	Homo sapiens	RefSeq	ILMN_14046	ILMN_14046
6.08213776	6.77149079	6.72222146	Homo sapiens	RefSeq	ILMN_25402	ILMN_25402
6.04755562	6.51673206	6.31353901	Homo sapiens	RefSeq	ILMN_44907	ILMN_44907
6.83848541	6.75807794	7.20646936	Homo sapiens	RefSeq	ILMN_15994	ILMN_15181
13.6547289	14.622816	14.6640189	Homo sapiens	RefSeq	ILMN_35496	ILMN_177367
5.76119377	6.39055454	6.0976799	Homo sapiens	RefSeq	ILMN_1751	ILMN_1751
6.43122162	6.90784607	6.97013236	Homo sapiens	RefSeq	ILMN_30583	ILMN_30583
5.67033843	6.1823584	5.74994101	Homo sapiens	RefSeq	ILMN_15769	ILMN_183654
12.3451726	13.073193	13.2527543	Homo sapiens	RefSeq	ILMN_32160	ILMN_174488
8.1885033	8.52416361	9.06119728	Homo sapiens	RefSeq	ILMN_3417	ILMN_3417
5.9899907	8.49095514	9.00098391	Homo sapiens	RefSeq	ILMN_138567	ILMN_31250
6.08181063	7.26917438	5.80481896	Homo sapiens	RefSeq	ILMN_15744	ILMN_15744
5.86357179	6.09396638	5.95579728	Homo sapiens	RefSeq	ILMN_1531	ILMN_1531
10.2864861	12.5287586	12.4803199	Homo sapiens	RefSeq	ILMN_8851	ILMN_8851
7.92595967	8.81944881	8.55818305	Homo sapiens	RefSeq	ILMN_19305	ILMN_19305
8.79318743	9.82913317	10.0279253	Homo sapiens	RefSeq	ILMN_12279	ILMN_12279
8.05654343	10.3114872	10.5697531	Homo sapiens	RefSeq	ILMN_31523	ILMN_31523
5.76318102	6.07136153	6.17545514	Homo sapiens	RefSeq	ILMN_15938	ILMN_15938
6.1393422	6.48752429	6.47511164	Homo sapiens	RefSeq	ILMN_24447	ILMN_24447
7.72865455	7.27269883	6.4778269	Homo sapiens	RefSeq	ILMN_42231	ILMN_176099
8.55487966	10.9623444	10.5746966	Homo sapiens	RefSeq	ILMN_20636	ILMN_168435
6.33627714	7.49249495	7.80071939	Homo sapiens	RefSeq	ILMN_15535	ILMN_15535
7.85899736	9.37486799	9.02749358	Homo sapiens	RefSeq	ILMN_22340	ILMN_22340
7.44936741	8.69201459	8.51930758	Homo sapiens	RefSeq	ILMN_995	ILMN_995
6.82117446	7.23306358	7.6858869	Homo sapiens	RefSeq	ILMN_9404	ILMN_182113
5.54807868	5.8915424	5.92268358	Homo sapiens	RefSeq	ILMN_34248	ILMN_34248

6.1939903	6.8981382	6.91484074	Homo sapiens	RefSeq	ILMN_42592	ILMN_42592
11.1713676	13.1413659	13.2033448	Homo sapiens	RefSeq	ILMN_26119	ILMN_26119
9.68366154	10.6716269	10.9023118	Homo sapiens	RefSeq	ILMN_42664	ILMN_42664
13.2587677	14.2716636	14.2069304	Homo sapiens	RefSeq	ILMN_37216	ILMN_37216
10.9224692	12.9381742	13.2282947	Homo sapiens	RefSeq	ILMN_29191	ILMN_29191
8.21526215	10.7422008	10.3238226	Homo sapiens	RefSeq	ILMN_36201	ILMN_36201
11.5818102	12.7677903	12.9685854	Homo sapiens	RefSeq	ILMN_17455	ILMN_17455
6.22960311	8.41023679	8.0308194	Homo sapiens	RefSeq	ILMN_14893	ILMN_171466
6.53664152	6.95810374	6.81283319	Homo sapiens	RefSeq	ILMN_40864	ILMN_43403
8.18971191	8.57455152	8.96336285	Homo sapiens	RefSeq	ILMN_26249	ILMN_26249
7.7919612	9.12730104	9.08543528	Homo sapiens	RefSeq	ILMN_12388	ILMN_178719
12.1973015	13.3677697	13.6779211	Homo sapiens	RefSeq	ILMN_13161	ILMN_13161
8.56675238	9.85024061	10.026226	Homo sapiens	RefSeq	ILMN_6379	ILMN_6379
6.2196274	6.33709664	7.55480294	Homo sapiens	RefSeq	ILMN_139245	ILMN_162831
7.78687599	7.61825775	9.09785647	Homo sapiens	RefSeq	ILMN_1388	ILMN_1388
6.13249767	7.65115576	7.78868375	Homo sapiens	RefSeq	ILMN_3109	ILMN_172627
12.793107	13.8746539	13.9223029	Homo sapiens	RefSeq	ILMN_25646	ILMN_25646
5.61021105	5.74475366	5.79724208	Homo sapiens	RefSeq	ILMN_33035	ILMN_33035
7.62880129	8.38505953	8.40343944	Homo sapiens	RefSeq	ILMN_2433	ILMN_162668
8.33250924	8.72076651	9.25371845	Homo sapiens	RefSeq	ILMN_29405	ILMN_29405
5.76909704	6.46949644	6.35823044	Homo sapiens	RefSeq	ILMN_25839	ILMN_38096
6.0564177	9.62960925	5.97930629	Homo sapiens	RefSeq	ILMN_3298	ILMN_3298
7.15526452	8.96222163	9.02775675	Homo sapiens	RefSeq	ILMN_27136	ILMN_27136
6.30553156	7.49203663	7.89883172	Homo sapiens	RefSeq	ILMN_10110	ILMN_177429
6.30448385	7.15897114	7.26752082	Homo sapiens	RefSeq	ILMN_769	ILMN_769
10.1019678	11.8365999	12.1586564	Homo sapiens	RefSeq	ILMN_6780	ILMN_6780
9.96064854	11.2012822	10.7853642	Homo sapiens	RefSeq	ILMN_137685	ILMN_137685
9.00824764	10.9315568	10.662599	Homo sapiens	RefSeq	ILMN_25069	ILMN_25069
7.56343044	9.00873786	8.7370543	Homo sapiens	RefSeq	ILMN_15990	ILMN_167378
7.01142743	7.88518448	7.48701356	Homo sapiens	RefSeq	ILMN_14182	ILMN_14182
10.4197959	12.4262207	12.248676	Homo sapiens	RefSeq	ILMN_12219	ILMN_183064
5.54509668	6.2865598	5.88588791	Homo sapiens	RefSeq	ILMN_34579	ILMN_172509
9.15095665	10.7765548	10.6961225	Homo sapiens	RefSeq	ILMN_4224	ILMN_168866
8.11911491	8.68143416	8.87682111	Homo sapiens	RefSeq	ILMN_5706	ILMN_164962
6.62459575	7.56498933	7.56059039	Homo sapiens	RefSeq	ILMN_3279	ILMN_3279
6.09956916	6.80518281	6.77417032	Homo sapiens	RefSeq	ILMN_18423	ILMN_171627
8.73148128	9.1761916	9.44255375	Homo sapiens	RefSeq	ILMN_1770	ILMN_1770
9.37080988	11.8023504	11.5807009	Homo sapiens	RefSeq	ILMN_25195	ILMN_25195
13.5248514	14.675817	14.5569562	Homo sapiens	RefSeq	ILMN_19648	ILMN_19648
9.92613496	11.4941826	11.3737232	Homo sapiens	RefSeq	ILMN_25641	ILMN_178109
5.93495377	6.67644091	6.58753602	Homo sapiens	RefSeq	ILMN_23234	ILMN_23234
9.20518477	10.0784404	9.80408487	Homo sapiens	RefSeq	ILMN_901	ILMN_901
6.46632889	7.32717087	7.24311208	Homo sapiens	RefSeq	ILMN_28103	ILMN_28103
12.982615	14.165795	13.9227693	Homo sapiens	RefSeq	ILMN_8741	ILMN_8741
8.55960538	9.11777866	10.0122865	Homo sapiens	RefSeq	ILMN_19827	ILMN_19827
9.71594844	10.4448234	10.1652826	Homo sapiens	RefSeq	ILMN_19124	ILMN_168791
6.06711766	6.38765515	6.14283042	Homo sapiens	RefSeq	ILMN_778	ILMN_7072
9.64763471	10.7615562	10.9435573	Homo sapiens	RefSeq	ILMN_22886	ILMN_22886

6.57090293	6.58751411	7.32557171	Homo sapiens	RefSeq	ILMN_6505	ILMN_6505
14.2421685	14.7880326	14.5089405	Homo sapiens	RefSeq	ILMN_13072	ILMN_13072
10.3017728	10.7551213	13.4331029	Homo sapiens	RefSeq	ILMN_2381	ILMN_183928
10.837512	11.651415	11.6826865	Homo sapiens	RefSeq	ILMN_7263	ILMN_7263
8.59380492	9.19428876	10.1202029	Homo sapiens	RefSeq	ILMN_4868	ILMN_4868
6.80256031	9.14200313	8.85498523	Homo sapiens	RefSeq	ILMN_24216	ILMN_169443
6.75530689	7.18166612	7.3755078	Homo sapiens	RefSeq	ILMN_23294	ILMN_176692
10.1476883	12.1618184	11.9097731	Homo sapiens	RefSeq	ILMN_139337	ILMN_10538
6.14605112	7.27352148	7.39784146	Homo sapiens	RefSeq	ILMN_16361	ILMN_16361
5.95162276	6.78227095	6.48853108	Homo sapiens	RefSeq	ILMN_570	ILMN_570
5.88328038	5.5029123	5.83596221	Homo sapiens	RefSeq	ILMN_32065	ILMN_32065
6.49442793	7.2409859	7.29403069	Homo sapiens	RefSeq	ILMN_29403	ILMN_29403
12.0424754	13.3828374	13.4280631	Homo sapiens	RefSeq	ILMN_17961	ILMN_17961
7.55427726	8.86560863	8.46877146	Homo sapiens	RefSeq	ILMN_6966	ILMN_178207
7.24234336	8.57458672	9.04636173	Homo sapiens	RefSeq	ILMN_39734	ILMN_39734
8.50458147	10.4850464	10.3690466	Homo sapiens	RefSeq	ILMN_2794	ILMN_2794
10.7177535	12.0170172	11.9036959	Homo sapiens	RefSeq	ILMN_20221	ILMN_20221
6.35945071	7.0560925	8.60265099	Homo sapiens	RefSeq	ILMN_17425	ILMN_17425
8.38449238	10.3903818	10.0666557	Homo sapiens	RefSeq	ILMN_7707	ILMN_7707
5.9269179	6.51159944	6.46227502	Homo sapiens	RefSeq	ILMN_12259	ILMN_12259
13.0120403	14.1587051	13.9141347	Homo sapiens	RefSeq	ILMN_15150	ILMN_15150
7.95791866	8.76156985	8.78357746	Homo sapiens	RefSeq	ILMN_15131	ILMN_15131
5.69030618	6.09537191	6.25772462	Homo sapiens	RefSeq	ILMN_46214	ILMN_46214
6.9372561	7.78065656	7.54643458	Homo sapiens	RefSeq	ILMN_3191	ILMN_3191
11.5763955	12.4517564	12.215681	Homo sapiens	RefSeq	ILMN_40380	ILMN_40380
8.40402134	8.97032969	8.93731967	Homo sapiens	RefSeq	ILMN_21163	ILMN_21163
5.6136339	7.29236489	6.42858814	Homo sapiens	RefSeq	ILMN_14457	ILMN_14457
6.02208768	7.46381207	5.45146698	Homo sapiens	RefSeq	ILMN_29309	ILMN_163189
6.70744196	8.27952051	8.26543971	Homo sapiens	RefSeq	ILMN_6014	ILMN_6014
8.03060645	9.18869738	8.78722969	Homo sapiens	RefSeq	ILMN_30338	ILMN_30338
7.9868	9.02375796	9.16607895	Homo sapiens	RefSeq	ILMN_22111	ILMN_22111
5.93378435	6.6165434	7.25534871	Homo sapiens	RefSeq	ILMN_6021	ILMN_6021
8.90378998	8.97479027	8.9581664	Homo sapiens	RefSeq	ILMN_3784	ILMN_3784
6.99708462	8.02234673	7.88361104	Homo sapiens	RefSeq	ILMN_1253	ILMN_1253
6.54171585	8.11854817	8.04760927	Homo sapiens	RefSeq	ILMN_23658	ILMN_23658
6.30006953	7.09994949	7.01231055	Homo sapiens	RefSeq	ILMN_46774	ILMN_46774
5.87725223	6.17410436	6.11412086	Homo sapiens	RefSeq	ILMN_137381	ILMN_38635
5.79650016	6.62280402	6.79205233	Homo sapiens	RefSeq	ILMN_8921	ILMN_8921
8.92376721	10.6045847	10.0430437	Homo sapiens	RefSeq	ILMN_24358	ILMN_24358
8.48724716	10.9535199	11.5308399	Homo sapiens	RefSeq	ILMN_17672	ILMN_167369
5.6278531	6.57237569	6.59659201	Homo sapiens	RefSeq	ILMN_38817	ILMN_38817
5.93189647	6.198281	5.98902726	Homo sapiens	RefSeq	ILMN_27791	ILMN_179289
6.92195159	7.64988702	8.06009441	Homo sapiens	RefSeq	ILMN_7648	ILMN_7648
5.95765811	6.34953725	6.5548413	Homo sapiens	RefSeq	ILMN_22044	ILMN_22044
5.49342766	6.23688366	6.13759287	Homo sapiens	RefSeq	ILMN_22616	ILMN_22616
11.0269847	12.956042	13.0088409	Homo sapiens	RefSeq	ILMN_33107	ILMN_168984
7.21766598	9.48784767	10.3110296	Homo sapiens	RefSeq	ILMN_19255	ILMN_19255
8.32153536	9.3093447	9.25412513	Homo sapiens	RefSeq	ILMN_29038	ILMN_180494

8.96101986	9.75210613	9.36159007	Homo sapiens	RefSeq	ILMN_5061	ILMN_7619
11.0026765	11.5203997	11.5176153	Homo sapiens	RefSeq	ILMN_35801	ILMN_175200
5.64942002	6.83187977	7.00571886	Homo sapiens	RefSeq	ILMN_29296	ILMN_29296
8.44302682	9.36500592	9.41632697	Homo sapiens	RefSeq	ILMN_25681	ILMN_25681
6.98561314	8.00183658	8.05792135	Homo sapiens	RefSeq	ILMN_12744	ILMN_12744
5.95315021	6.21523768	6.14981336	Homo sapiens	RefSeq	ILMN_39876	ILMN_166286
6.12541574	6.81140938	7.17537589	Homo sapiens	RefSeq	ILMN_8732	ILMN_8732
10.0400225	9.7400509	9.81702201	Homo sapiens	RefSeq	ILMN_14838	ILMN_14838
8.01899296	9.03954174	8.72948344	Homo sapiens	RefSeq	ILMN_18313	ILMN_18313
6.09599191	6.94797117	7.06461344	Homo sapiens	RefSeq	ILMN_26138	ILMN_167082
6.76006789	7.7188219	7.35081987	Homo sapiens	RefSeq	ILMN_21751	ILMN_180252
5.76923751	6.02265189	6.12495752	Homo sapiens	RefSeq	ILMN_17623	ILMN_17623
8.47676686	10.8707315	11.3102742	Homo sapiens	RefSeq	ILMN_21130	ILMN_21130
7.57315106	8.70655645	8.36261629	Homo sapiens	RefSeq	ILMN_10077	ILMN_164547
7.21764272	8.6855926	9.07121269	Homo sapiens	RefSeq	ILMN_137528	ILMN_8866
6.07901024	6.3479643	5.56099216	Homo sapiens	RefSeq	ILMN_15605	ILMN_182614
5.38512935	6.18662832	5.91761339	Homo sapiens	RefSeq	ILMN_46634	ILMN_46634
9.95551188	12.0431644	12.468247	Homo sapiens	RefSeq	ILMN_18627	ILMN_171368
8.13106622	9.00161114	9.97848024	Homo sapiens	RefSeq	ILMN_11407	ILMN_175392
11.1021614	12.5996077	12.5414788	Homo sapiens	RefSeq	ILMN_25325	ILMN_25325
6.36764731	6.20693894	5.99035248	Homo sapiens	RefSeq	ILMN_437	ILMN_437
5.82785941	5.95699312	6.05287601	Homo sapiens	RefSeq	ILMN_21456	ILMN_169410
13.5092851	14.4748708	14.3844728	Homo sapiens	RefSeq	ILMN_46346	ILMN_46346
13.4394245	14.643027	14.654947	Homo sapiens	RefSeq	ILMN_2142	ILMN_2142
8.2980039	8.68549029	8.34397182	Homo sapiens	RefSeq	ILMN_19998	ILMN_19998
5.99122341	6.5985385	6.73979006	Homo sapiens	RefSeq	ILMN_1700	ILMN_1700
6.46612085	7.30875585	7.74677227	Homo sapiens	RefSeq	ILMN_11669	ILMN_11669
6.22151772	7.21461929	7.42242056	Homo sapiens	RefSeq	ILMN_13531	ILMN_13531
6.2956604	7.98996267	8.44860862	Homo sapiens	RefSeq	ILMN_16330	ILMN_166425
5.56227021	6.30518003	6.29290559	Homo sapiens	RefSeq	ILMN_24057	ILMN_24057
11.8699813	12.9003919	12.4969084	Homo sapiens	RefSeq	ILMN_26505	ILMN_171578
8.84721116	11.2552659	11.982512	Homo sapiens	RefSeq	ILMN_14503	ILMN_167991
9.62021433	9.56831756	9.85754237	Homo sapiens	RefSeq	ILMN_20088	ILMN_20088
7.81539226	8.74146294	8.94530219	Homo sapiens	RefSeq	ILMN_32712	ILMN_172960
6.53120349	9.28045738	8.62018463	Homo sapiens	RefSeq	ILMN_19556	ILMN_169784
6.62139828	7.74380778	7.52863773	Homo sapiens	RefSeq	ILMN_13107	ILMN_180143
7.37876997	7.30868305	7.34594452	Homo sapiens	RefSeq	ILMN_7503	ILMN_7503
8.07671033	9.50236422	8.91722258	Homo sapiens	RefSeq	ILMN_138771	ILMN_19611
6.04097635	7.03143159	7.14677129	Homo sapiens	RefSeq	ILMN_20446	ILMN_20446
6.75323146	7.78938366	7.89534262	Homo sapiens	RefSeq	ILMN_29106	ILMN_29106
8.29410101	10.1075218	9.44421297	Homo sapiens	RefSeq	ILMN_18687	ILMN_18687
10.2460624	12.4535235	12.4617368	Homo sapiens	RefSeq	ILMN_13489	ILMN_13489
10.7959349	12.1979391	12.3174474	Homo sapiens	RefSeq	ILMN_19743	ILMN_19743
7.13342173	9.83039399	9.48174112	Homo sapiens	RefSeq	ILMN_11925	ILMN_177943
5.76584473	6.25230892	6.11438285	Homo sapiens	RefSeq	ILMN_11712	ILMN_11712
6.41815267	7.29062069	7.09738957	Homo sapiens	RefSeq	ILMN_138991	ILMN_37228
8.16088642	8.82519413	10.2302703	Homo sapiens	RefSeq	ILMN_45021	ILMN_33467
7.73565485	8.08031869	8.19426685	Homo sapiens	RefSeq	ILMN_26226	ILMN_181539

7.06142736	8.14313522	7.83448116	Homo sapiens	RefSeq		ILMN_6262	ILMN_162583
5.75865203	6.03328996	6.0759463	Homo sapiens	RefSeq		ILMN_31151	ILMN_31151
6.9382305	8.01859913	7.69660524	Homo sapiens	RefSeq		ILMN_30967	ILMN_30967
8.70941334	9.44059285	9.44480308	Homo sapiens	RefSeq		ILMN_13488	ILMN_13488
7.53931626	8.9702293	9.90079935	Homo sapiens	RefSeq		ILMN_136933	ILMN_41474
11.1240547	12.2940538	12.5155589	Homo sapiens	RefSeq		ILMN_699	ILMN_699
7.04636882	7.78460213	5.62112849	Homo sapiens	RefSeq		ILMN_139242	ILMN_183214
6.96005871	8.70822449	8.8796978	Homo sapiens	RefSeq		ILMN_23642	ILMN_23642
11.0369717	12.1935916	12.2577585	Homo sapiens	RefSeq		ILMN_22327	ILMN_22327
11.4270603	13.168473	13.4538545	Homo sapiens	RefSeq		ILMN_6745	ILMN_6745
6.69023549	5.9461502	6.2678294	Homo sapiens	RefSeq		ILMN_14042	ILMN_174401
6.43645671	7.14917298	7.28892789	Homo sapiens	RefSeq		ILMN_20343	ILMN_20343
6.29701364	8.58293266	8.52114342	Homo sapiens	RefSeq		ILMN_20608	ILMN_20608
5.58327807	6.66979923	5.28791254	Homo sapiens	RefSeq		ILMN_3531	ILMN_173170
9.52166953	10.4823734	10.5189452	Homo sapiens	RefSeq		ILMN_46177	ILMN_46177
9.56922258	10.4720044	10.2040918	Homo sapiens	RefSeq		ILMN_30057	ILMN_30057
7.75190017	10.3067863	10.1628294	Homo sapiens	RefSeq		ILMN_137748	ILMN_21291
8.06294845	10.3625324	9.53251568	Homo sapiens	RefSeq		ILMN_29455	ILMN_166212
7.70486367	8.81663522	8.91854337	Homo sapiens	RefSeq		ILMN_136990	ILMN_136990
12.0504544	13.0323437	13.0402443	Homo sapiens	RefSeq		ILMN_9174	ILMN_167736
6.03287952	6.5922836	6.63119889	Homo sapiens	RefSeq		ILMN_23418	ILMN_183042
5.67898063	6.83787766	7.13795634	Homo sapiens	RefSeq		ILMN_29669	ILMN_162233
5.8207549	6.54840087	7.15078508	Homo sapiens	RefSeq		ILMN_28694	ILMN_28694
5.92300985	6.61684404	6.94555855	Homo sapiens	RefSeq		ILMN_42790	ILMN_42790
5.96193165	6.46999421	6.37085093	Homo sapiens	RefSeq		ILMN_33488	ILMN_33488
7.13067352	8.88304585	8.56274461	Homo sapiens	RefSeq		ILMN_11223	ILMN_11223
6.90098029	9.45707856	8.89485565	Homo sapiens	RefSeq		ILMN_29847	ILMN_29956
6.15554889	7.57958446	7.98556418	Homo sapiens	RefSeq		ILMN_138041	ILMN_168110
11.7431232	13.3141173	13.2551272	Homo sapiens	RefSeq		ILMN_30243	ILMN_30243
7.90877749	8.94070091	8.91868587	Homo sapiens	RefSeq		ILMN_24567	ILMN_165486
5.25637213	5.39097922	8.26576599	Homo sapiens	RefSeq		ILMN_6666	ILMN_9927
6.3092828	6.72366914	6.62704734	Homo sapiens	RefSeq		ILMN_16752	ILMN_16752
7.5650297	9.23892225	9.85247099	Homo sapiens	RefSeq		ILMN_20363	ILMN_20363
6.93477949	7.93649425	7.50017584	Homo sapiens	RefSeq		ILMN_14733	ILMN_14733
7.11240927	8.99939885	8.81952322	Homo sapiens	RefSeq		ILMN_32472	ILMN_32472
8.14599887	9.12760439	10.3136914	Homo sapiens	RefSeq		ILMN_9232	ILMN_9232
5.95244703	6.99944874	6.8910169	Homo sapiens	RefSeq		ILMN_10766	ILMN_170754
6.21862426	7.5804327	7.83903557	Homo sapiens	RefSeq		ILMN_16789	ILMN_16789
8.49188556	10.6694529	10.4149484	Homo sapiens	RefSeq		ILMN_3851	ILMN_3851
7.52220409	8.19462587	8.3831971	Homo sapiens	RefSeq		ILMN_10351	ILMN_10351
7.82937835	7.78850301	8.58415752	Homo sapiens	RefSeq		ILMN_21404	ILMN_176421
6.0490792	6.81061148	6.86708843	Homo sapiens	RefSeq		ILMN_947	ILMN_947
7.65415983	8.80725284	9.29973737	Homo sapiens	RefSeq		ILMN_3908	ILMN_3908
8.37827535	10.1799079	10.0714262	Homo sapiens	RefSeq		ILMN_1690	ILMN_1690
7.77788717	8.73785979	8.97293701	Homo sapiens	RefSeq		ILMN_17166	ILMN_17166
6.13921448	6.47609568	6.53681105	Homo sapiens	RefSeq		ILMN_12799	ILMN_165929
5.69856216	6.32748366	6.13773548	Homo sapiens	RefSeq		ILMN_18612	ILMN_18612
6.55731374	7.82732619	7.74494683	Homo sapiens	RefSeq		ILMN_5095	ILMN_5095

7.47660041	8.14213004	8.23969972	Homo sapiens	RefSeq	ILMN_11642	ILMN_11642
10.7630605	12.1658523	11.8868626	Homo sapiens	RefSeq	ILMN_7921	ILMN_7921
5.44263967	6.12051244	6.18417177	Homo sapiens	RefSeq	ILMN_44054	ILMN_44054
10.1432454	10.3250952	11.4883292	Homo sapiens	RefSeq	ILMN_4432	ILMN_182487
6.80479573	8.72604067	8.88727607	Homo sapiens	RefSeq	ILMN_28389	ILMN_28389
6.56522595	7.74302951	7.93079883	Homo sapiens	RefSeq	ILMN_2176	ILMN_179020
6.46414595	7.97471228	7.69234699	Homo sapiens	RefSeq	ILMN_8616	ILMN_174337
5.80468424	6.53196388	6.21979407	Homo sapiens	RefSeq	ILMN_10243	ILMN_18648
7.19850103	9.39704865	9.59090175	Homo sapiens	RefSeq	ILMN_138444	ILMN_175751
6.02377586	6.44020575	6.33897539	Homo sapiens	RefSeq	ILMN_40991	ILMN_40991
6.79444446	8.85689857	8.77211626	Homo sapiens	RefSeq	ILMN_19298	ILMN_19298
6.28937488	7.11488233	6.84492164	Homo sapiens	RefSeq	ILMN_17030	ILMN_17030
6.56295138	6.6553733	6.78352737	Homo sapiens	RefSeq	ILMN_7286	ILMN_174352
7.40320039	8.1761926	8.44276356	Homo sapiens	RefSeq	ILMN_16356	ILMN_16356
11.5678387	12.1511488	12.6771296	Homo sapiens	RefSeq	ILMN_28136	ILMN_166624
7.40315266	9.13887896	6.56280733	Homo sapiens	RefSeq	ILMN_11562	ILMN_11562
9.80641268	11.8570576	11.9640108	Homo sapiens	RefSeq	ILMN_8146	ILMN_8146
5.91669733	6.94386742	5.73199263	Homo sapiens	RefSeq	ILMN_9065	ILMN_9065
7.75258336	9.40231821	9.00914979	Homo sapiens	RefSeq	ILMN_4662	ILMN_165570
13.4084359	14.2533667	14.3859958	Homo sapiens	RefSeq	ILMN_3192	ILMN_3192
7.01203002	8.49933527	7.31517946	Homo sapiens	RefSeq	ILMN_12611	ILMN_13173
12.0130089	12.4914255	12.7418356	Homo sapiens	RefSeq	ILMN_11171	ILMN_16074
8.53037551	10.1701554	10.7000998	Homo sapiens	RefSeq	ILMN_19425	ILMN_19425
10.9656602	12.4339701	12.8920443	Homo sapiens	RefSeq	ILMN_8829	ILMN_8829
9.05136476	9.87390115	9.61277397	Homo sapiens	RefSeq	ILMN_12517	ILMN_12517
7.64959893	9.47157219	9.33444984	Homo sapiens	RefSeq	ILMN_9298	ILMN_179207
7.27916946	8.93435874	7.75690987	Homo sapiens	RefSeq	ILMN_7804	ILMN_7804
9.26028517	10.3441838	10.3582446	Homo sapiens	RefSeq	ILMN_21292	ILMN_164727
5.47711646	5.50914602	5.39452889	Homo sapiens	RefSeq	ILMN_45279	ILMN_45279
9.54392772	12.8809778	13.5569155	Homo sapiens	RefSeq	ILMN_23476	ILMN_23476
6.00082255	6.57540461	6.48576943	Homo sapiens	RefSeq	ILMN_17742	ILMN_17742
5.87419203	6.68151673	6.45664502	Homo sapiens	RefSeq	ILMN_9041	ILMN_166025
10.6973056	12.1960992	12.4550963	Homo sapiens	RefSeq	ILMN_28796	ILMN_28796
6.11374883	7.74810561	8.66813012	Homo sapiens	RefSeq	ILMN_13325	ILMN_13325
8.88433783	10.4517211	11.1148563	Homo sapiens	RefSeq	ILMN_18970	ILMN_18970
9.13093988	9.86079508	9.68253028	Homo sapiens	RefSeq	ILMN_6070	ILMN_6070
6.32530294	7.03278521	7.56126656	Homo sapiens	RefSeq	ILMN_15080	ILMN_15080
8.34775069	9.4023568	9.99042453	Homo sapiens	RefSeq	ILMN_38143	ILMN_39285
6.32249082	6.94447664	7.21137827	Homo sapiens	RefSeq	ILMN_12235	ILMN_163778
12.1448542	13.0201605	13.1468219	Homo sapiens	RefSeq	ILMN_37107	ILMN_37107
7.77325857	9.20512929	9.10324217	Homo sapiens	RefSeq	ILMN_3472	ILMN_3472
5.60731788	6.07013297	6.16978213	Homo sapiens	RefSeq	ILMN_34080	ILMN_34080
7.47569128	8.2680914	8.23323475	Homo sapiens	RefSeq	ILMN_11126	ILMN_11126
6.33303725	7.31080543	7.46615397	Homo sapiens	RefSeq	ILMN_14436	ILMN_175775
11.9295957	12.8262776	13.0895258	Homo sapiens	RefSeq	ILMN_26264	ILMN_26264
6.7447168	8.11050576	7.51891984	Homo sapiens	RefSeq	ILMN_25018	ILMN_183093
6.8399004	8.62482104	8.64773651	Homo sapiens	RefSeq	ILMN_14371	ILMN_162663
6.80666175	8.21699413	8.1548176	Homo sapiens	RefSeq	ILMN_17714	ILMN_28998

6.41694291	7.11535791	7.32205523	Homo sapiens	RefSeq	ILMN_7082	ILMN_7082
5.83204617	7.15401504	6.58568397	Homo sapiens	RefSeq	ILMN_139301	ILMN_12834
5.9507572	6.60119003	6.60960472	Homo sapiens	RefSeq	ILMN_603	ILMN_172522
6.84474468	7.09514908	7.22884081	Homo sapiens	RefSeq	ILMN_26589	ILMN_172304
9.49874093	10.4078881	10.6662498	Homo sapiens	RefSeq	ILMN_12735	ILMN_12735
8.10268595	9.41523325	9.58903612	Homo sapiens	RefSeq	ILMN_23452	ILMN_163689
11.1344751	11.81807	11.8633976	Homo sapiens	RefSeq	ILMN_138365	ILMN_138365
7.8003596	8.60400829	8.21552087	Homo sapiens	RefSeq	ILMN_631	ILMN_631
6.45181659	7.02112878	7.20948066	Homo sapiens	RefSeq	ILMN_6117	ILMN_6117
8.09604148	8.7463682	8.57771885	Homo sapiens	RefSeq	ILMN_2801	ILMN_173774
7.0596592	8.06153213	8.0161241	Homo sapiens	RefSeq	ILMN_20456	ILMN_26347
10.541932	11.3637345	11.5165023	Homo sapiens	RefSeq	ILMN_31021	ILMN_31021
6.16944583	5.89546186	5.51038219	Homo sapiens	RefSeq	ILMN_8145	ILMN_8145
12.9273945	13.7247334	13.7618452	Homo sapiens	RefSeq	ILMN_6782	ILMN_6782
6.67172363	7.17625295	7.09090846	Homo sapiens	RefSeq	ILMN_407	ILMN_179898
8.40839386	9.4224542	9.542739	Homo sapiens	RefSeq	ILMN_9587	ILMN_166150
7.62536548	9.27986846	9.0571188	Homo sapiens	RefSeq	ILMN_16570	ILMN_16570
6.10876969	6.41044677	6.33187501	Homo sapiens	Unigene	ILMN_71475	ILMN_71475
7.03630002	8.57814245	8.6109403	Homo sapiens	Unigene	ILMN_110256	ILMN_110256
5.76897031	6.02158411	6.45944211	Homo sapiens	Unigene	ILMN_83667	ILMN_83667
6.80473637	7.62913656	7.59877534	Homo sapiens	Unigene	ILMN_88124	ILMN_88124
5.56277757	5.68174104	5.51213427	Homo sapiens	Unigene	ILMN_72534	ILMN_72534
5.93081751	6.01640049	6.09168714	Homo sapiens	Unigene	ILMN_72439	ILMN_72439
6.17492928	6.8155242	6.69190679	Homo sapiens	Unigene	ILMN_108071	ILMN_108071
5.91663404	6.5062714	6.40411223	Homo sapiens	Unigene	ILMN_122129	ILMN_122129
5.5753677	5.6530216	5.68293196	Homo sapiens	Unigene	ILMN_127638	ILMN_127638
6.14682993	6.99273671	6.65014847	Homo sapiens	Unigene	ILMN_79879	ILMN_79879
7.20904584	8.69088951	8.4535077	Homo sapiens	Unigene	ILMN_77737	ILMN_77737
6.19957734	6.40525977	5.88512187	Homo sapiens	Unigene	ILMN_79403	ILMN_79403
5.85089216	7.15976137	7.15601733	Homo sapiens	Unigene	ILMN_109663	ILMN_109663
5.41209862	5.61590325	5.67415667	Homo sapiens	Unigene	ILMN_129862	ILMN_129862
5.66938626	6.16051587	6.51072794	Homo sapiens	Unigene	ILMN_78641	ILMN_78641
6.09696789	6.87201117	7.01254856	Homo sapiens	Unigene	ILMN_77463	ILMN_77463
5.61851178	5.92164317	5.76506235	Homo sapiens	Unigene	ILMN_103202	ILMN_103202
6.92748288	8.01268152	7.80938229	Homo sapiens	Unigene	ILMN_126852	ILMN_126852
5.73611478	6.78651273	7.0464867	Homo sapiens	Unigene	ILMN_89339	ILMN_89339
6.70335961	7.83953828	7.89070082	Homo sapiens	Unigene	ILMN_87950	ILMN_87950
6.02045331	6.65250514	6.46199275	Homo sapiens	Unigene	ILMN_88401	ILMN_88401
5.68810033	6.01200834	6.03536438	Homo sapiens	Unigene	ILMN_116097	ILMN_116097
6.91426672	8.90782083	8.70106729	Homo sapiens	Unigene	ILMN_92403	ILMN_92403
6.54124533	7.49441561	7.54890666	Homo sapiens	Unigene	ILMN_79788	ILMN_79788
6.92426738	7.50718573	7.20997479	Homo sapiens	Unigene	ILMN_105449	ILMN_105449
6.72490534	7.67033333	7.66126291	Homo sapiens	Unigene	ILMN_117456	ILMN_117456
5.97373272	6.82197164	6.31094172	Homo sapiens	Unigene	ILMN_109018	ILMN_109018
5.38814234	5.6400274	5.51004982	Homo sapiens	Unigene	ILMN_95052	ILMN_95052
5.72661245	6.40794242	6.38140175	Homo sapiens	Unigene	ILMN_99761	ILMN_99761
5.61538481	5.54358682	5.66604473	Homo sapiens	Unigene	ILMN_114283	ILMN_114283
6.25507069	8.29989577	8.24923484	Homo sapiens	Unigene	ILMN_92383	ILMN_92383

5.41443527	6.40685857	6.45951883	Homo sapiens	Unigene	ILMN_73408	ILMN_73408
5.99587522	6.57032373	6.5101899	Homo sapiens	Unigene	ILMN_99800	ILMN_99800
6.03929678	6.38247653	7.20121628	Homo sapiens	Unigene	ILMN_121484	ILMN_121484
5.61851442	6.1781294	6.12093228	Homo sapiens	Unigene	ILMN_109150	ILMN_109150
5.9236479	6.87604047	6.95389843	Homo sapiens	Unigene	ILMN_84672	ILMN_84672
6.62531857	8.2508588	8.16564719	Homo sapiens	Unigene	ILMN_78063	ILMN_78063
7.48491068	8.50950425	8.53226474	Homo sapiens	Unigene	ILMN_127215	ILMN_127215
6.75467108	8.49404953	7.8014303	Homo sapiens	Unigene	ILMN_74776	ILMN_74776
5.58783361	5.57974012	5.67440726	Homo sapiens	Unigene	ILMN_74698	ILMN_74698
6.14740867	6.85517247	7.14860979	Homo sapiens	Unigene	ILMN_95926	ILMN_95926
5.74183611	6.49237109	6.47857792	Homo sapiens	Unigene	ILMN_123922	ILMN_123922
6.41141642	7.18474545	7.44757259	Homo sapiens	Unigene	ILMN_104197	ILMN_104197
8.10830385	9.16661528	8.3070546	Homo sapiens	Unigene	ILMN_88069	ILMN_88069
5.89651309	6.31232718	6.04533615	Homo sapiens	Unigene	ILMN_115613	ILMN_115613
6.20224511	7.59002933	7.27089051	Homo sapiens	Unigene	ILMN_87223	ILMN_87223
6.01642055	7.09681739	7.56708906	Homo sapiens	Unigene	ILMN_82381	ILMN_82381
5.8587755	6.39020766	6.98962611	Homo sapiens	Unigene	ILMN_113840	ILMN_113840
6.08754664	6.91100122	6.91031174	Homo sapiens	Unigene	ILMN_117493	ILMN_117493
5.90611638	6.48274989	5.99353036	Homo sapiens	Unigene	ILMN_84598	ILMN_84598
5.33306803	5.50428256	5.43861423	Homo sapiens	Unigene	ILMN_83745	ILMN_83745
5.41592257	5.61916882	5.60920268	Homo sapiens	Unigene	ILMN_114953	ILMN_114953
6.11630611	7.19650279	7.31186636	Homo sapiens	Unigene	ILMN_92557	ILMN_92557
5.41922234	5.54791494	5.43992361	Homo sapiens	Unigene	ILMN_105493	ILMN_105493
6.19599792	6.22783501	6.34136048	Homo sapiens	Unigene	ILMN_115487	ILMN_115487
5.50281176	5.72124277	6.05887017	Homo sapiens	Unigene	ILMN_110945	ILMN_110945
5.74270609	5.9758255	5.36278231	Homo sapiens	Unigene	ILMN_125443	ILMN_125443
5.88200617	7.5932447	6.91074825	Homo sapiens	Unigene	ILMN_93354	ILMN_93354
5.98193437	6.69571966	6.45524288	Homo sapiens	Unigene	ILMN_71784	ILMN_71784
5.67313237	6.06788864	6.09339982	Homo sapiens	Unigene	ILMN_86767	ILMN_86767
6.01742022	7.27328742	7.31751572	Homo sapiens	Unigene	ILMN_93814	ILMN_93814
6.55833469	7.25028989	7.28333195	Homo sapiens	Unigene	ILMN_122524	ILMN_122524
6.2408871	6.74431989	6.58973323	Homo sapiens	Unigene	ILMN_94830	ILMN_94830
7.40017499	9.16748037	8.75643023	Homo sapiens	Unigene	ILMN_76886	ILMN_76886
5.90778936	6.40991259	7.22441754	Homo sapiens	Unigene	ILMN_124625	ILMN_124625
6.42576123	6.69258342	6.60469481	Homo sapiens	Unigene	ILMN_134294	ILMN_134294
6.23599639	6.87516079	6.53738849	Homo sapiens	Unigene	ILMN_96448	ILMN_96448
5.92923615	6.55106453	7.07721095	Homo sapiens	Unigene	ILMN_113743	ILMN_113743
7.16264208	8.97357081	8.96305125	Homo sapiens	Unigene	ILMN_79925	ILMN_79925
6.35363258	7.23498025	7.15138801	Homo sapiens	Unigene	ILMN_134444	ILMN_134444
6.28148851	7.01563806	7.54175797	Homo sapiens	Unigene	ILMN_123332	ILMN_123332
5.5156929	6.02768797	6.03937071	Homo sapiens	Unigene	ILMN_91878	ILMN_91878
5.85297512	7.06959369	6.33078282	Homo sapiens	Unigene	ILMN_77095	ILMN_77095
12.0845237	12.771378	13.2017821	Homo sapiens	ILMN_Controls		ILMN_160472

ILMN_Gene	Source_Reference_ID	RefSeq_ID	Unigene_ID	Entrez_Gene_ID
DTX2P1-UPK3BP1-PMS2P11	XM_933516.1	XM_933516.1		643862
LINC00243	XM_936105.1	XM_936105.1		642035
LINC-PINT	XM_935575.1	XM_935575.1		641825
LINC00639	XM_378454.3	XM_378454.3		283547
AC068587.4	XM_943677.1	XM_943677.1		654053
AL445524.1	XM_933693.2	XM_933693.2		440731
DNAJC3-DT	XM_943048.1	XM_943048.1		647834
AL162377.1	XR_000601.1	XR_000601.1		283521
AC108134.3	XM_941963.1	XM_941963.1		649754
LINC-PINT	XM_930545.1	XM_930545.1		378805
AC114980.1	XR_001261.1	XR_001261.1		80042
LINC00921	XM_370932.4	XM_370932.4		283876
SCARNA9	NR_002569.1	NR_002569.1		619383
AC113143.1	Hs.571432		Hs.571432	NA
LINC02555	Hs.306876		Hs.306876	NA
HDAC4-AS1	Hs.534680		Hs.534680	NA
AC123595.1	Hs.482814		Hs.482814	NA
FOXP1-IT1	Hs.549989		Hs.549989	NA
AC004803.1	Hs.270094		Hs.270094	NA
NDUFV2-AS1	Hs.569879		Hs.569879	NA
PRKCQ-AS1	Hs.13262		Hs.13262	NA
RNU6ATAC35P	Hs.562110		Hs.562110	NA
MIR29B2CHG	Hs.445414		Hs.445414	NA
TXN	NM_003329.1	NM_003329.1		NA
RPS28	NM_001031.4	NM_001031.4		6234
GADD45G	NM_006705.2	NM_006705.2		10912
RPS16	NM_001020.4	NM_001020.4		6217
ABCC5	NM_001023587.1	NM_001023587.1		10057
GNG10	NM_001017998.2	NM_001017998.2		2790
LMBRD1	NM_018368.2	NM_018368.2		55788
LOC647361	XM_936430.2	XM_936430.2		647361
C9ORF19	NM_022343.2	NM_022343.2		152007
RPS6KA3	NM_004586.2	NM_004586.2		6197
RAXL1	NM_032753.2	NM_032753.2		84839
LOC390033	XM_372345.3	XM_372345.3		390033
PBEF1	NM_005746.2	NM_005746.2		10135
LOC644250	XM_929199.1	XM_929199.1		644250
TMPIT	NM_031925.1	NM_031925.1		83862
PLAU	NM_002658.2	NM_002658.2		5328
PPP2R3A	NM_181897.1	NM_181897.1		5523
ZYG11B	NM_024646.1	NM_024646.1		79699
CD82	NM_002231.3	NM_002231.3		3732
POLR2J	NM_006234.4	NM_006234.4		5439
LOC284757	NM_001004305.1	NM_001004305.1		284757
KIAA0319	NM_014809.2	NM_014809.2		9856
RPS6KA5	NM_004755.2	NM_004755.2		9252
MEGF9	NM_001080497.1	NM_001080497.1		1955

LOC644852	XM_934218.2	XM_934218.2	644852
LOC653635	XR_017611.1	XR_017611.1	653635
HIST2H2AA3	NM_003516.2	NM_003516.2	8337
TXNL5	NM_032731.2	NM_032731.2	84817
LOC653158	XM_926814.1	XM_926814.1	653158
PAK2	XM_936511.1	XM_936511.1	5062
RPS27	NM_001030.3	NM_001030.3	6232
GPR177	NM_001002292.1	NM_001002292.1	79971
LOC653352	XM_934316.1	XM_934316.1	653352
CSF2RA	NM_172246.1	NM_172246.1	1438
LOC646424	XM_941267.2	XM_941267.2	646424
ZMYM2	NM_003453.2	NM_003453.2	7750
PPP2R3C	NM_017917.2	NM_017917.2	55012
GPR160	NM_014373.1	NM_014373.1	26996
FLJ11151	NM_018340.1	NM_018340.1	55313
RGR	NM_153615.1	NM_153615.1	266747
EFCBP2	NM_019065.2	NM_019065.2	54550
CACNA1E	NM_000721.2	NM_000721.2	777
DLD	NM_000108.3	NM_000108.3	1738
UNC119	NM_005148.2	NM_005148.2	9094
LOC653604	XM_497711.2	XM_497711.2	653604
TMBIM4	NM_016056.2	NM_016056.2	51643
WSB1	NM_015626.8	NM_015626.8	26118
SH3BP5L	NM_030645.1	NM_030645.1	80851
USP15	NM_006313.1	NM_006313.1	9958
LOC390466	XM_372521.1	XM_372521.1	390466
AKTIP	NM_022476.2	NM_022476.2	64400
TRIM56	NM_030961.1	NM_030961.1	81844
CDC123	NM_006023.1	NM_006023.1	8872
NKX3-1	NM_006167.2	NM_006167.2	4824
LPPR2	NM_022737.1	NM_022737.1	64748
SULF2	NM_198596.1	NM_198596.1	55959
IFRD1	NM_001550.2	NM_001550.2	3475
LOC728825	XM_001128769.1	XM_001128769.1	728825
NTNG2	NM_032536.1	NM_032536.1	84628
LOC391475	XM_937277.1	XM_937277.1	391475
DHRS12	NM_001031719.1	NM_001031719.1	79758
PDE7A	NM_002604.1	NM_002604.1	5150
MNDA	NM_002432.1	NM_002432.1	4332
ARID3A	NM_005224.2	NM_005224.2	1820
CYORF15A	NM_001005852.1	NM_001005852.1	246126
CIR	NM_004882.3	NM_004882.3	9541
CLCC1	NM_001048210.1	NM_001048210.1	23155
TOB1	NM_005749.2	NM_005749.2	10140
SP110	NM_004510.2	NM_004510.2	3431
LMNB1	NM_005573.2	NM_005573.2	4001
F2RL1	NM_005242.3	NM_005242.3	2150
LOC652578	XM_942097.1	XM_942097.1	652578

C9ORF95	NM_017881.1	NM_017881.1	54981
HSD17B11	NM_016245.2	NM_016245.2	51170
LOC441268	NM_001013725.1	NM_001013725.1	441268
SIRPB1	NM_006065.1	NM_006065.1	10326
IFNGR1	NM_000416.1	NM_000416.1	3459
NBPF9	XM_934022.1	XM_934022.1	440670
CUEDC1	NM_017949.1	NM_017949.1	404093
Septin 2	NM_001008491.1	NM_001008491.1	4735
CLEC4D	NM_080387.4	NM_080387.4	338339
LOC731706	XR_016005.1	XR_016005.1	731706
PTGS2	NM_000963.1	NM_000963.1	5743
RAB24	NM_130781.2	NM_130781.2	53917
MME	NM_007288.2	NM_007288.2	4311
UPB1	NM_016327.2	NM_016327.2	51733
TAF15	NM_139215.1	NM_139215.1	8148
BCYRN1	NR_001568.1	NR_001568.1	618
RAPGEF1	NM_198679.1	NM_198679.1	2889
VNN2	NM_078488.1	NM_078488.1	8875
SCGB3A1	NM_052863.2	NM_052863.2	92304
LOC651894	XM_941155.2	XM_941155.2	651894
USP49	NM_018561.3	NM_018561.3	25862
TXN	NM_003329.2	NM_003329.2	7295
ACP6	NM_016361.2	NM_016361.2	51205
LOC650919	XM_944417.1	XM_944417.1	650919
PRKAR2A	NM_004157.2	NM_004157.2	5576
NT5C2	NM_012229.2	NM_012229.2	22978
UBE2D3	NM_181889.1	NM_181889.1	7323
PBX2	NM_002586.4	NM_002586.4	5089
PFKFB2	NM_006212.2	NM_006212.2	5208
TMEM154	NM_152680.1	NM_152680.1	201799
THRAP2	NM_015335.2	NM_015335.2	23389
ASAH1	NM_177924.2	NM_177924.2	427
ACSL1	NM_001995.2	NM_001995.2	2180
PSG11	NM_002785.2	NM_002785.2	5680
TMCC3	NM_020698.1	NM_020698.1	57458
C15ORF51	NR_003260.1	NR_003260.1	196968
THBS1	NM_003246.2	NM_003246.2	7057
SLCO4C1	NM_180991.4	NM_180991.4	353189
MGEA5	NM_012215.1	NM_012215.1	10724
HLX1	NM_021958.2	NM_021958.2	3142
SULF2	NM_018837.2	NM_018837.2	55959
RBM7	NM_016090.2	NM_016090.2	10179
IFI6	NM_002038.3	NM_002038.3	2537
LOC651894	XM_941155.2	XM_941155.2	651894
PTP4A2	XM_944915.1	XM_944915.1	8073
CAMP	NM_004345.3	NM_004345.3	820
HIST1H2BH	NM_003524.2	NM_003524.2	8345
TNFRSF12A	NM_016639.1	NM_016639.1	51330

C19ORF40	NM_152266.1	NM_152266.1	91442
PTPLAD2	NM_001010915.1	NM_001010915.1	401494
STAT1	NM_139266.1	NM_139266.1	6772
PSMD12	XM_001134072.1	XM_001134072.1	5718
PLAUR	NM_001005376.1	NM_001005376.1	5329
LOC646064	XM_933375.1	XM_933375.1	646064
IL4R	NM_001008699.1	NM_001008699.1	3566
ZNF14	NM_021030.2	NM_021030.2	7561
BCKDK	NM_005881.1	NM_005881.1	10295
CMTM1	NM_181301.1	NM_181301.1	113540
ALPP	NM_001632.3	NM_001632.3	250
GADD45A	NM_001924.2	NM_001924.2	1647
ICA1	NM_022308.1	NM_022308.1	3382
CLTCL1	NM_007098.2	NM_007098.2	8218
RPS29	NM_001032.3	NM_001032.3	6235
LOC653610	XM_928387.1	XM_928387.1	653610
SRPK2	NM_182692.1	NM_182692.1	6733
CLK4	NM_020666.2	NM_020666.2	57396
LOC642333	XR_019071.1	XR_019071.1	642333
MME	NM_000902.3	NM_000902.3	4311
PYGL	NM_002863.3	NM_002863.3	5836
ROPN1L	NM_031916.2	NM_031916.2	83853
TLE4	NM_007005.3	NM_007005.3	7091
FARSLB	NM_005687.2	NM_005687.2	10056
LOC649841	XM_938906.2	XM_938906.2	649841
RBM3	NM_001017431.1	NM_001017431.1	5935
LOC440926	XR_017837.1	XR_017837.1	440926
IFIT1	NM_001548.2	NM_001548.2	3434
LOC442609	XM_935827.1	XM_935827.1	442609
USP9Y	NM_004654.3	NM_004654.3	8287
LOC400721	XR_018616.1	XR_018616.1	400721
CLEC7A	NM_197953.1	NM_197953.1	64581
SAP30	NM_003864.3	NM_003864.3	8819
UTY	NM_182659.1	NM_182659.1	7404
PFN2	NM_053024.3	NM_053024.3	5217
MXD1	NM_002357.2	NM_002357.2	4084
HSD17B12	NM_016142.1	NM_016142.1	51144
SF3B14	NM_016047.3	NM_016047.3	51639
LOC347376	XM_937928.1	XM_937928.1	347376
LOC283152	NM_001033658.1	NM_001033658.1	283152
CAMK1D	NM_020397.2	NM_020397.2	57118
LOC653635	XR_017611.1	XR_017611.1	653635
EIF2AK2	NM_002759.1	NM_002759.1	5610
BIN3	NM_018688.4	NM_018688.4	55909
GOLPH3	NM_022130.3	NM_022130.3	64083
EXOSC3	NM_001002269.1	NM_001002269.1	51010
RUNDC2B	NM_001012391.1	NM_001012391.1	400509
LOC647499	XM_942939.1	XM_942939.1	647499

LOC644591	XM_927706.2	XM_927706.2	644591
RGS2	NM_002923.1	NM_002923.1	5997
LOC653489	XM_934113.1	XM_934113.1	653489
LOC645895	XM_928866.1	XM_928866.1	645895
GMFG	NM_004877.1	NM_004877.1	9535
LOC644969	XM_928047.1	XM_928047.1	644969
CKLF	NM_016326.2	NM_016326.2	51192
NUDT16P	NR_002949.1	NR_002949.1	152195
LOC653505	XM_927767.2	XM_927767.2	653505
NQO2	NM_000904.1	NM_000904.1	4835
PBLD	NM_022129.3	NM_022129.3	64081
S100A6	NM_014624.3	NM_014624.3	6277
ZNF786	NM_152411.1	NM_152411.1	136051
FAM101B	XM_001126665.1	XM_001126665.1	359845
FAM101B	NM_182705.2	NM_182705.2	359845
ZNF354A	NM_005649.2	NM_005649.2	6940
S100A9	NM_002965.3	NM_002965.3	6280
LOC440160	XM_498571.2	XM_498571.2	440160
OGFRL1	NM_024576.3	NM_024576.3	79627
RBP7	NM_052960.1	NM_052960.1	116362
C22ORF34	NM_001039473.1	NM_001039473.1	348645
PVRL2	NM_002856.2	NM_002856.2	5819
FLJ31951	NM_144726.1	NM_144726.1	153830
HAL	NM_002108.2	NM_002108.2	3034
ARSG	NM_014960.2	NM_014960.2	22901
USP10	NM_005153.2	NM_005153.2	9100
CSF2RA	XM_942501.1	XM_942501.1	1438
DKFZP564K142	NM_032121.3	NM_032121.3	84061
EXOC8	NM_175876.3	NM_175876.3	149371
PPARBP	NM_004774.2	NM_004774.2	5469
DHRS8	XM_001132265.1	XM_001132265.1	51170
LOC728575	XM_001127825.1	XM_001127825.1	728575
LTB4R	NM_181657.1	NM_181657.1	1241
HK2	NM_000189.4	NM_000189.4	3099
LOC402176	NM_001011538.1	NM_001011538.1	402176
LMTK2	NM_014916.2	NM_014916.2	22853
TACC3	NM_006342.1	NM_006342.1	10460
LY96	NM_015364.2	NM_015364.2	23643
B2M	NM_004048.2	NM_004048.2	567
C12ORF35	NM_018169.2	NM_018169.2	55196
HECTD2	NM_173497.1	NM_173497.1	143279
TNRC6B	NM_001024843.1	NM_001024843.1	23112
ZKSCAN1	NM_003439.1	NM_003439.1	7586
FCGR3B	NM_000570.2	NM_000570.2	2215
CREB5	NM_001011666.1	NM_001011666.1	9586
IL17RA	NM_014339.4	NM_014339.4	23765
PTPRN2	NM_130843.1	NM_130843.1	5799
RNF130	NM_018434.4	NM_018434.4	55819

TNNI2	NM_003282.2	NM_003282.2	7136
S100A8	NM_002964.3	NM_002964.3	6279
FOLR3	NM_000804.2	NM_000804.2	2352
ANKRD30B	NM_001029862.1	NM_001029862.1	374860
CDK5R1	NM_003885.2	NM_003885.2	8851
TLR1	NM_003263.3	NM_003263.3	7096
KIAA1618	NM_020954.2	NM_020954.2	57714
RPL26	NM_000987.3	NM_000987.3	6154
ORM2	NM_000608.2	NM_000608.2	5005
CEP152	NM_014985.1	NM_014985.1	22995
LOC653316	XM_933119.1	XM_933119.1	653316
PPM1B	NM_001033556.1	NM_001033556.1	5495
KLF6	NM_001300.4	NM_001300.4	1316
CYSLTR1	NM_006639.2	NM_006639.2	10800
LOC644033	XM_927280.1	XM_927280.1	644033
YRDC	NM_024640.3	NM_024640.3	79693
CYP4F3	NM_000896.1	NM_000896.1	4051
PHACTR1	NM_030948.1	NM_030948.1	221692
PCMTD1	NM_052937.1	NM_052937.1	115294
FLJ34047	NM_173669.1	NM_173669.1	285696
RPS29	NM_001032.3	NM_001032.3	6235
CEP27	NM_018097.1	NM_018097.1	55142
LOC644964	XM_928041.1	XM_928041.1	644964
GOLGA2	NM_004486.4	NM_004486.4	2801
LOC643284	XM_928180.1	XM_928180.1	643284
UPF2	NM_015542.2	NM_015542.2	26019
RN7SK	NR_001445.1	NR_001445.1	125050
UTY	NM_007125.3	NM_007125.3	7404
PPP1R12A	NM_002480.1	NM_002480.1	4659
RAB3GAP1	NM_012233.1	NM_012233.1	22930
C14ORF102	NM_017970.2	NM_017970.2	55051
FCAR	NM_133279.1	NM_133279.1	2204
OSCAR	NM_130771.2	NM_130771.2	126014
KIF1B	NM_183416.2	NM_183416.2	23095
PHF3	NM_015153.1	NM_015153.1	23469
LOC653829	XM_935802.1	XM_935802.1	653829
MDM4	NM_002393.2	NM_002393.2	4194
EPC1	NM_025209.2	NM_025209.2	80314
RAE1	NM_003610.3	NM_003610.3	8480
PSCDBP	NM_004288.3	NM_004288.3	9595
LOC643882	XM_931879.1	XM_931879.1	643882
ZNF346	NM_012279.2	NM_012279.2	23567
JDP2	NM_130469.2	NM_130469.2	122953
SLC2A11	NM_001024938.1	NM_001024938.1	66035
SLC5A9	NM_001011547.1	NM_001011547.1	200010
LOC730740	XM_001128558.1	XM_001128558.1	730740
S100A12	NM_005621.1	NM_005621.1	6283
RCSD1	NM_052862.2	NM_052862.2	92241

CSF2RA	NM_172246.1	NM_172246.1	1438
SDHALP1	NR_003264.1	NR_003264.1	255812
AMN1	NM_207337.1	NM_207337.1	196394
TM6SF1	NM_023003.1	NM_023003.1	53346
C7ORF25	NM_024054.1	NM_024054.1	79020
FLJ90086	XM_371820.5	XM_371820.5	389389
SYNJ2	NM_003898.2	NM_003898.2	8871
STX10	NM_003765.1	NM_003765.1	8677
RFFL	NM_001017368.1	NM_001017368.1	117584
ADCK4	NM_024876.2	NM_024876.2	79934
TRIM25	NM_005082.4	NM_005082.4	7706
ANKRD44	NM_153697.1	NM_153697.1	91526
HEBP2	NM_014320.2	NM_014320.2	23593
EIF2AK4	NM_001013703.2	NM_001013703.2	440275
RPL23	NM_000978.3	NM_000978.3	9349
CYORF15B	NM_032576.2	NM_032576.2	84663
LOC647691	XM_936748.1	XM_936748.1	647691
TNFSF13B	NM_006573.3	NM_006573.3	10673
FADD	NM_003824.2	NM_003824.2	8772
RGS18	NM_130782.2	NM_130782.2	64407
TAP2	NM_018833.2	NM_018833.2	6891
PNMA6A	NM_032882.2	NM_032882.2	84968
LOC643509	XM_928156.1	XM_928156.1	643509
SRGN	NM_002727.2	NM_002727.2	5552
EOMES	NM_005442.2	NM_005442.2	8320
PSMA1	NM_148976.1	NM_148976.1	5682
ENSA	NM_207168.1	NM_207168.1	2029
C10ORF119	NM_024834.1	NM_024834.1	79892
HIF1A	NM_001530.2	NM_001530.2	3091
RBMXL1	NM_019610.3	NM_019610.3	494115
EVI2B	NM_006495.2	NM_006495.2	2124
IRS2	NM_003749.2	NM_003749.2	8660
ANPEP	NM_001150.1	NM_001150.1	290
MBOAT1	XM_001131044.1	XM_001131044.1	154141
HDAC4	NM_006037.2	NM_006037.2	9759
ZNF585A	NM_152655.2	NM_152655.2	199704
OXER1	NM_148962.4	NM_148962.4	165140
MAP3K2	NM_006609.3	NM_006609.3	10746
RAP1A	NM_001010935.1	NM_001010935.1	5906
LOC552891	NM_004125.2	NM_004125.2	552891
RBM25	NM_021239.1	NM_021239.1	58517
ARPC5	NM_005717.2	NM_005717.2	10092
LOC440093	NM_001013699.1	NM_001013699.1	440093
NT5C3	NM_001002009.1	NM_001002009.1	51251
RPL32	NM_001007074.1	NM_001007074.1	6161
ANKRD55	NM_024669.2	NM_024669.2	79722
LOC649821	XM_942212.2	XM_942212.2	649821
BST1	NM_004334.1	NM_004334.1	683

CBX1	NM_006807.3	NM_006807.3	10951
LOC641978	XM_935752.1	XM_935752.1	641978
LOC653158	XM_926814.1	XM_926814.1	653158
FLJ38717	NM_001004322.1	NM_001004322.1	401261
CLEC4E	NM_014358.2	NM_014358.2	26253
C20ORF43	NM_016407.1	NM_016407.1	51507
PRKY	NM_002760.3	NM_002760.3	5616
TLR2	NM_003264.3	NM_003264.3	7097
MCART1	NM_033412.1	NM_033412.1	92014
NFKBIA	NM_020529.1	NM_020529.1	4792
LRRN3	NM_018334.3	NM_018334.3	54674
SLC26A8	NM_052961.2	NM_052961.2	116369
HECW2	NM_020760.1	NM_020760.1	57520
TMSB4Y	NM_004202.2	NM_004202.2	9087
LOC653596	XM_934651.1	XM_934651.1	653596
MPPE1	NM_023075.4	NM_023075.4	65258
LRRK2	NM_198578.2	NM_198578.2	120892
TAP2	NM_000544.3	NM_000544.3	6891
FLJ40722	XM_942096.1	XM_942096.1	285966
IL18	NM_001562.2	NM_001562.2	3606
PICALM	NM_007166.2	NM_007166.2	8301
CHD7	NM_017780.2	NM_017780.2	55636
CDH2	NM_001792.2	NM_001792.2	1000
LOC440456	XM_935186.1	XM_935186.1	440456
MGC12760	XM_930480.1	XM_930480.1	84809
PPP1R3D	NM_006242.3	NM_006242.3	5509
OSBPL8	NM_001003712.1	NM_001003712.1	114882
SLC25A20	XM_001133926.1	XM_001133926.1	788
IL8RB	NM_001557.2	NM_001557.2	3579
ASB8	NM_024095.3	NM_024095.3	140461
BTNL3	NM_006707.2	NM_006707.2	10917
PNMA3	NM_013364.3	NM_013364.3	29944
TUBA4A	NM_006000.1	NM_006000.1	7277
DTX3L	NM_138287.2	NM_138287.2	151636
LOC647389	XM_936461.1	XM_936461.1	647389
OSBPL9	NM_024586.3	NM_024586.3	114883
GPR84	NM_020370.1	NM_020370.1	53831
SUMO1P1	NR_002189.2	NR_002189.2	391257
LMOD3	NM_198271.2	NM_198271.2	56203
TGM3	NM_003245.2	NM_003245.2	7053
NOV	NM_002514.2	NM_002514.2	4856
ALS2CR14	NM_178231.1	NM_178231.1	65068
ADD3	NM_016824.3	NM_016824.3	120
CEP63	NM_025180.3	NM_025180.3	80254
CXCL1	NM_001511.1	NM_001511.1	2919
LINS1	NM_001040616.1	NM_001040616.1	55180
LOC441193	NM_001013722.1	NM_001013722.1	441193
PSIP1	NM_033222.2	NM_033222.2	11168

CBX3	NM_016587.2	NM_016587.2	11335
CRISPLD2	NM_031476.1	NM_031476.1	83716
LOC653105	XM_931214.1	XM_931214.1	653105
CKAP4	NM_006825.2	NM_006825.2	10970
DJ341D10.1	NM_001007535.1	NM_001007535.1	286453
FBXL13	NM_145032.2	NM_145032.2	222235
BTAF1	NM_003972.2	NM_003972.2	9044
MLX	NM_170607.2	NM_170607.2	6945
NPTN	NM_012428.2	NM_012428.2	27020
LOC653604	XM_497711.2	XM_497711.2	653604
COX7B	NM_001866.2	NM_001866.2	1349
POU2F1	NM_002697.2	NM_002697.2	5451
LOC729853	XM_001134256.1	XM_001134256.1	729853
BMX	NM_001721.4	NM_001721.4	660
MMP9	NM_004994.2	NM_004994.2	4318
AYTL1	NM_017839.3	NM_017839.3	54947
NCF2	NM_000433.2	NM_000433.2	4688
TTTY15	NR_001545.1	NR_001545.1	64595
MAP4K4	NM_145686.2	NM_145686.2	9448
ALOX5AP	NM_001629.2	NM_001629.2	241
PSMB9	NM_148954.2	NM_148954.2	5698
MCM8	NM_182802.1	NM_182802.1	84515
SRPK1	NM_003137.3	NM_003137.3	6732
CMTM2	NM_144673.2	NM_144673.2	146225
MOSC1	NM_022746.2	NM_022746.2	64757
IBRDC2	NM_182757.2	NM_182757.2	255488
ANKRD22	NM_144590.1	NM_144590.1	118932
CUGBP2	NM_001025077.1	NM_001025077.1	10659
LOC648226	XM_938919.1	XM_938919.1	648226
S100P	NM_005980.2	NM_005980.2	6286
PSG9	NM_002784.2	NM_002784.2	5678
BRWD1	NM_033656.2	NM_033656.2	54014
ABTB1	NM_172027.1	NM_172027.1	80325
SLC36A4	NM_152313.2	NM_152313.2	120103
ZNF281	NM_012482.3	NM_012482.3	23528
C1ORF108	NM_024595.1	NM_024595.1	79647
ANP32A	NM_006305.2	NM_006305.2	8125
LOC388789	XM_939954.2	XM_939954.2	388789
ABAT	NM_020686.4	NM_020686.4	18
ZNF486	XM_371152.3	XM_371152.3	90649
CHD8	NM_020920.2	NM_020920.2	57680
KIAA0492	XM_944290.2	XM_944290.2	57238
MGC4093	NM_030578.2	NM_030578.2	80776
ANXA11	NM_145869.1	NM_145869.1	311
PADI4	NM_012387.1	NM_012387.1	23569
KIAA0319L	NM_182686.1	NM_182686.1	79932
TDRD1	NM_198795.1	NM_198795.1	56165
ARID4A	NM_023001.2	NM_023001.2	5926

SLC5A8	NM_145913.2	NM_145913.2			160728
FLJ20152	NM_019000.3	NM_019000.3			54463
FLJ14107	XR_017962.1	XR_017962.1			80094
KIAA1706	NM_030636.2	NM_030636.2			80820
PIP5K2B	NM_138687.1	NM_138687.1			8396
RAB35	NM_006861.4	NM_006861.4			11021
AHR	NM_001621.2	NM_001621.2			196
ZSWIM1	NM_080603.3	NM_080603.3			90204
ANKDD1A	NM_182703.3	NM_182703.3			348094
SP110	NM_080424.1	NM_080424.1			3431
CORO2A	NM_003389.2	NM_003389.2			7464
LOC653086	XM_930995.1	XM_930995.1			653086
IL9R	NM_002186.2	NM_002186.2			3581
C21ORF55	NM_017833.2	NM_017833.2			54943
PLAGL1	NM_001080951.1	NM_001080951.1			5325
ZNF223	NM_013361.2	NM_013361.2			7766
GTF2IP1	NR_002206.1	NR_002206.1			2970
HS.16587	Hs.16587		Hs.16587	NA	
HS.550293	Hs.550293		Hs.550293	NA	
HS.257589	Hs.257589		Hs.257589	NA	
HS.371006	Hs.371006		Hs.371006	NA	
HS.38775	Hs.38775		Hs.38775	NA	
HS.36034	Hs.36034		Hs.36034	NA	
HS.544709	Hs.544709		Hs.544709	NA	
HS.569948	Hs.569948		Hs.569948	NA	
HS.575457	Hs.575457		Hs.575457	NA	
HS.162932	Hs.162932		Hs.162932	NA	
HS.136423	Hs.136423		Hs.136423	NA	
HS.157344	Hs.157344		Hs.157344	NA	
HS.547856	Hs.547856		Hs.547856	NA	
HS.577681	Hs.577681		Hs.577681	NA	
HS.148238	Hs.148238		Hs.148238	NA	
HS.133261	Hs.133261		Hs.133261	NA	
HS.537137	Hs.537137		Hs.537137	NA	
HS.574671	Hs.574671		Hs.574671	NA	
HS.389491	Hs.389491		Hs.389491	NA	
HS.369643	Hs.369643		Hs.369643	NA	
HS.374460	Hs.374460		Hs.374460	NA	
HS.563172	Hs.563172		Hs.563172	NA	
HS.441953	Hs.441953		Hs.441953	NA	
HS.161796	Hs.161796		Hs.161796	NA	
HS.540965	Hs.540965		Hs.540965	NA	
HS.564874	Hs.564874		Hs.564874	NA	
HS.545935	Hs.545935		Hs.545935	NA	
HS.470701	Hs.470701		Hs.470701	NA	
HS.520349	Hs.520349		Hs.520349	NA	
HS.560503	Hs.560503		Hs.560503	NA	
HS.441660	Hs.441660		Hs.441660	NA	

HS.66290	Hs.66290		Hs.66290	NA
HS.520591	Hs.520591		Hs.520591	NA
HS.569303	Hs.569303		Hs.569303	NA
HS.546105	Hs.546105		Hs.546105	NA
HS.283402	Hs.283402		Hs.283402	NA
HS.144312	Hs.144312		Hs.144312	NA
HS.575034	Hs.575034		Hs.575034	NA
HS.103173	Hs.103173		Hs.103173	NA
HS.101139	Hs.101139		Hs.101139	NA
HS.482960	Hs.482960		Hs.482960	NA
HS.571741	Hs.571741		Hs.571741	NA
HS.538918	Hs.538918		Hs.538918	NA
HS.370503	Hs.370503		Hs.370503	NA
HS.562530	Hs.562530		Hs.562530	NA
HS.352677	Hs.352677		Hs.352677	NA
HS.213474	Hs.213474		Hs.213474	NA
HS.559770	Hs.559770		Hs.559770	NA
HS.564915	Hs.564915		Hs.564915	NA
HS.282467	Hs.282467		Hs.282467	NA
HS.259391	Hs.259391		Hs.259391	NA
HS.561570	Hs.561570		Hs.561570	NA
HS.443534	Hs.443534		Hs.443534	NA
HS.541035	Hs.541035		Hs.541035	NA
HS.562352	Hs.562352		Hs.562352	NA
HS.552324	Hs.552324		Hs.552324	NA
HS.573262	Hs.573262		Hs.573262	NA
HS.447737	Hs.447737		Hs.447737	NA
HS.22689	Hs.22689		Hs.22689	NA
HS.339693	Hs.339693		Hs.339693	NA
HS.452445	Hs.452445		Hs.452445	NA
HS.570343	Hs.570343		Hs.570343	NA
HS.467627	Hs.467627		Hs.467627	NA
HS.130036	Hs.130036		Hs.130036	NA
HS.572444	Hs.572444		Hs.572444	NA
HS.582113	Hs.582113		Hs.582113	NA
HS.490981	Hs.490981		Hs.490981	NA
HS.559604	Hs.559604		Hs.559604	NA
HS.163752	Hs.163752		Hs.163752	NA
HS.582263	Hs.582263		Hs.582263	NA
HS.571151	Hs.571151		Hs.571151	NA
HS.437283	Hs.437283		Hs.437283	NA
HS.131087	Hs.131087		Hs.131087	NA
TXN	NM_003329.1	NM_003329.1		NA

GI	Accession	Symbol	Protein_Product	Array_Address_Id	Probe_Type	Probe_Start
89025054	XM_933516.1	LOC643862	XP_938609.1	3610465	I	3261
88998653	XM_936105.1	LOC642035	XP_941198.1	360128	S	3
89027574	XM_935575.1	LOC641825	XP_940668.1	3180246	S	121
89037316	XM_378454.3	LOC283547	XP_378454.2	870575	A	4193
89028557	XM_943677.1	LOC654053	XP_948770.1	7400592	S	941
1.13E+08	XM_933693.2	LOC440731	XP_938786.2	270768	A	111
89037157	XM_943048.1	LOC647834	XP_948141.1	2600669	S	2498
89036737	XR_000601.1	FLJ37307		5490440	A	3986
89040587	XM_941963.1	LOC649754	XP_947056.1	70041	A	190
89025371	XM_930545.1	FLJ43663	XP_935638.1	3930180	A	1437
88988357	XR_001261.1	FLJ12078		7150039	A	2413
1.13E+08	XM_370932.4	FLJ39639	XP_370932.2	5420452	A	2341
77020264	NR_002569.1	SCARNA9		840639	S	46
6991367	AW450591			1340204	S	246
10439674	AK026751			4180274	S	757
41120019	XM_374029			1710411	S	382
30023571	AF452720			1110309	S	822
10438414	AK025793			3990600	S	3313
30047319	CB852325			4610437	S	475
15345167	BI520375			4260639	S	351
51468125	XM_498474			2030673	S	1607
27845617	BX103256			2760114	S	199
34528764	AK123264			6270605	S	2211
4507744	NM_003329.1	TXN		4260048	S	72
71565158	NM_001031.4	RPS28	NP_001022.1	7510672	S	329
9790905	NM_006705.2	GADD45G	NP_006696.1	70376	S	923
71482588	NM_001020.4	RPS16	NP_001011.1	130768	S	496
66529092	NM_001023587.1	ABCC5	NP_001018881.1	7610097	I	1804
89941472	NM_001017998.2	GNG10	NP_001017998.1	4590608	S	292
31542670	NM_018368.2	LMBRD1	NP_060838.2	4590301	S	1576
1.13E+08	XM_936430.2	LOC647361	XP_941523.1	3870088	S	27
22095361	NM_022343.2	C9orf19	NP_071738.1	4280253	S	1778
56243494	NM_004586.2	RPS6KA3	NP_004577.1	5890647	I	7247
20127653	NM_032753.2	RAXL1	NP_116142.1	3830382	S	2182
1.13E+08	XM_372345.3	LOC390033	XP_372345.3	2140537	S	108
1.11E+08	NM_005746.2	PBEF1	NP_005737.1	3800243	I	1544
89036604	XM_929199.1	LOC644250	XP_934292.1	940386	S	259
13994299	NM_031925.1	TMPIT	NP_114131.1	5560577	S	1055
53729348	NM_002658.2	PLAU	NP_002649.1	1740349	S	2049
32967585	NM_181897.1	PPP2R3A	NP_871626.1	540041	A	4082
55742692	NM_024646.1	ZYG11B	NP_078922.1	4610019	S	6333
67782352	NM_002231.3	CD82	NP_002222.1	6770594	A	1323
62422568	NM_006234.4	POLR2J	NP_006225.1	4810594	S	627
51972191	NM_001004305.1	LOC284757	NP_001004305.1	870349	S	4094
62988329	NM_014809.2	KIAA0319	NP_055624.1	5910102	S	6659
32528294	NM_004755.2	RPS6KA5	NP_004746.2	2030482	I	3521
1.23E+08	NM_001080497.1	MEGF9	NP_001073966.1	7560615	A	857

1.13E+08	XM_934218.2	LOC644852	XP_939311.1	2360053	I	879
1.13E+08	XR_017611.1	LOC653635		3990128	S	2595
21328454	NM_003516.2	HIST2H2AA3	NP_003507.1	1030039	S	1
21362103	NM_032731.2	TXNL5	NP_116120.1	830047	S	378
88983545	XM_926814.1	LOC653158	XP_931907.1	5820682	I	2213
88971807	XM_936511.1	PAK2	XP_941604.1	6110608	I	1688
68160923	NM_001030.3	RPS27	NP_001021.1	5290523	S	36
50541962	NM_001002292.1	GPR177	NP_001002292.1	540288	I	1848
89040118	XM_934316.1	LOC653352	XP_939409.1	4540653	I	2039
27437033	NM_172246.1	CSF2RA	NP_758449.1	4570168	A	1147
1.13E+08	XM_941267.2	LOC646424	XP_946360.1	520209	S	189
37574604	NM_003453.2	ZMYM2	NP_003444.1	6040079	A	4512
31542241	NM_017917.2	PPP2R3C	NP_060387.2	2760110	S	1264
7657135	NM_014373.1	GPR160	NP_055188.1	6180427	S	1437
8922900	NM_018340.1	FLJ11151	NP_060810.1	7550519	S	2496
23957679	NM_153615.1	Rgr	NP_705843.1	3440253	S	2648
32129213	NM_019065.2	EFCBP2	NP_061938.2	6770669	S	1468
53832004	NM_000721.2	CACNA1E	NP_000712.2	2760452	S	9561
91199539	NM_000108.3	DLD	NP_000099.2	1690671	S	1675
16936537	NM_005148.2	UNC119	NP_005139.1	1570164	A	1267
88943484	XM_497711.2	LOC653604	XP_497711.2	6650593	S	133
1.17E+08	NM_016056.2	TMBIM4	NP_057140.2	5270356	S	610
58331181	NM_015626.8	WSB1	NP_056441.6	6650575	A	2671
24308302	NM_030645.1	SH3BP5L	NP_085148.1	7320112	S	2889
14149626	NM_006313.1	USP15	NP_006304.1	70767	S	4159
41203895	XM_372521.1	LOC390466	XP_372521.1	6130672	S	61
61743931	NM_022476.2	AKTIP	NP_071921.1	2360414	A	1431
30794215	NM_030961.1	TRIM56	NP_112223.1	1780647	S	3115
5174422	NM_006023.1	CDC123	NP_006014.1	6110379	S	1191
19923351	NM_006167.2	NKX3-1	NP_006158.2	4670735	S	2969
12232394	NM_022737.1	LPPR2	NP_073574.1	3140274	S	2354
38327657	NM_198596.1	SULF2	NP_940998.1	5270762	A	3335
55953128	NM_001550.2	IFRD1	NP_001541.2	3780243	A	1957
1.13E+08	XM_001128769.1	LOC728825	XP_001128769.1	3060348	S	35
19387853	NM_032536.1	NTNG2	NP_115925.1	6270382	S	2101
88959061	XM_937277.1	LOC391475	XP_942370.1	2630253	A	601
72534731	NM_001031719.1	DHRS12	NP_001026889.1	4890114	A	562
24429563	NM_002604.1	PDE7A	NP_002595.1	6960221	I	2530
4505226	NM_002432.1	MNDA	NP_002423.1	6380228	S	1318
1.18E+08	NM_005224.2	ARID3A	NP_005215.1	4260446	S	2546
54291711	NM_001005852.1	CYorf15A	NP_001005852.1	2190192	S	1688
40068058	NM_004882.3	CIR	NP_004873.3	2640088	A	1294
1.15E+08	NM_001048210.1	CLCC1	NP_001041675.1	20674	S	4235
22035666	NM_005749.2	TOB1	NP_005740.1	2470477	S	892
17986251	NM_004510.2	SP110	NP_004501.2	70338	A	1274
27436949	NM_005573.2	LMNB1	NP_005564.1	4900048	S	2443
34577051	NM_005242.3	F2RL1	NP_005233.3	5690201	S	2245
89063135	XM_942097.1	LOC652578	XP_947190.1	3180192	S	537

8923529	NM_017881.1	C9orf95	NP_060351.1	7320079	S	1013
56786142	NM_016245.2	HSD17B11	NP_057329.1	2190475	I	465
61966904	NM_001013725.1	LOC441268	NP_001013747.1	3840102	S	2048
5174678	NM_006065.1	SIRPB1	NP_006056.1	1570402	S	3692
4557879	NM_000416.1	IFNGR1	NP_000407.1	1500446	S	1771
88943153	XM_934022.1	NBPF9	XP_939115.1	6270324	I	582
8923664	NM_017949.1	CUEDC1	NP_060419.1	2680725	S	2114
56549635	NM_001008491.1	Septin 2	NP_001008491.1	780431	I	352
37577120	NM_080387.4	CLEC4D	NP_525126.2	3990328	S	1551
1.13E+08	XR_016005.1	LOC731706		4180564	S	466
4506264	NM_000963.1	PTGS2	NP_000954.1	1470682	S	3943
96975134	NM_130781.2	RAB24	NP_570137.2	4730156	A	1124
1.16E+08	NM_007288.2	MME	NP_009219.2	240608	A	5051
56550123	NM_016327.2	UPB1	NP_057411.1	7160367	S	1915
21327700	NM_139215.1	TAF15	NP_631961.1	5960128	A	1913
34850482	NR_001568.1	BCYRN1		2450154	S	57
38373676	NM_198679.1	RAPGEF1	NP_941372.1	6100576	I	65
17865815	NM_078488.1	VNN2	NP_511043.1	5570673	A	1770
50363225	NM_052863.2	SCGB3A1	NP_443095.2	2060543	S	221
1.13E+08	XM_941155.2	LOC651894	XP_946248.1	1570750	S	7
34147686	NM_018561.3	USP49	NP_061031.2	7320088	S	2818
50592993	NM_003329.2	TXN	NP_003320.2	830762	S	43
21359910	NM_016361.2	ACP6	NP_057445.2	3180376	S	1595
89040641	XM_944417.1	LOC650919	XP_949510.1	4250543	S	1385
47157329	NM_004157.2	PRKAR2A	NP_004148.1	4900593	S	2304
20149601	NM_012229.2	NT5C2	NP_036361.1	520647	S	3272
33149315	NM_181889.1	UBE2D3	NP_871618.1	4640110	A	2052
1.24E+08	NM_002586.4	PBX2	NP_002577.2	5550500	S	3106
64762405	NM_006212.2	PFKFB2	NP_006203.2	3190326	I	6815
22749366	NM_152680.1	TMEM154	NP_689893.1	7650379	S	2498
47575843	NM_015335.2	THRAP2	NP_056150.1	1990332	S	9174
1.19E+08	NM_177924.2	ASAH1	NP_808592.1	840161	I	261
40807490	NM_001995.2	ACSL1	NP_001986.2	4880717	S	3553
42560239	NM_002785.2	PSG11	NP_002776.2	3400292	I	413
29789109	NM_020698.1	TMCC3	NP_065749.1	2650152	S	4505
1.18E+08	NR_003260.1	C15orf51		5910131	I	3782
40317625	NM_003246.2	THBS1	NP_003237.2	5910376	S	4947
38679889	NM_180991.4	SLCO4C1	NP_851322.3	770717	S	4454
11024697	NM_012215.1	MGEA5	NP_036347.1	1010402	S	4955
19923769	NM_021958.2	HLX1	NP_068777.1	1400706	S	1612
38327656	NM_018837.2	SULF2	NP_061325.1	1850056	I	2862
31543547	NM_016090.2	RBM7	NP_057174.1	5390326	S	1656
94538326	NM_002038.3	IFI6	NP_002029.3	4390176	A	743
1.13E+08	XM_941155.2	LOC651894	XP_946248.1	5130273	S	131
88947652	XM_944915.1	PTP4A2	XP_950008.1	5360603	I	2364
39753969	NM_004345.3	CAMP	NP_004336.2	6400736	S	644
21166386	NM_003524.2	HIST1H2BH	NP_003515.1	5720433	S	352
7706185	NM_016639.1	TNFRSF12A	NP_057723.1	2190008	S	634

22748622	NM_152266.1	C19orf40	NP_689479.1	7150189	S	676
58219057	NM_001010915.1	PTPLAD2	NP_001010915.1	1470091	S	3665
21536300	NM_139266.1	STAT1	NP_644671.1	3190133	I	2595
1.13E+08	XM_001134072.1	PSMD12	XP_001134072.1	1440131	I	2849
53829378	NM_001005376.1	PLAUR	NP_001005376.1	6130594	I	1107
89039665	XM_933375.1	LOC646064	XP_938468.1	2450014	S	1382
56788410	NM_001008699.1	IL4R	NP_001008699.1	1770678	I	962
38045951	NM_021030.2	ZNF14	NP_066358.2	2340674	S	2358
5031608	NM_005881.1	BCKDK	NP_005872.1	5390056	S	1742
31563433	NM_181301.1	CMTM1	NP_851818.1	4830017	A	704
94721245	NM_001632.3	ALPP	NP_001623.3	4230088	S	2672
9790904	NM_001924.2	GADD45A	NP_001915.1	3140239	S	659
12545396	NM_022308.1	ICA1	NP_071683.1	6560411	I	543
1.09E+08	NM_007098.2	CLTCL1	NP_009029.2	5090026	I	4593
71772593	NM_001032.3	RPS29	NP_001023.1	1990753	A	63
88943486	XM_928387.1	LOC653610	XP_933480.1	5670544	S	713
33188448	NM_182692.1	SRPK2	NP_872634.1	2350072	I	107
47717135	NM_020666.2	CLK4	NP_065717.1	6510110	S	2086
1.13E+08	XR_019071.1	LOC642333		1010296	A	611
1.16E+08	NM_000902.3	MME	NP_000893.2	2360400	I	28
71037378	NM_002863.3	PYGL	NP_002854.3	4610113	S	2494
17572806	NM_031916.2	ROPN1L	NP_114122.2	1050240	S	740
38327621	NM_007005.3	TLE4	NP_008936.2	6290170	S	4048
19923332	NM_005687.2	FARSLB	NP_005678.2	460128	S	2992
1.13E+08	XM_938906.2	LOC649841	XP_943999.2	4830300	S	644
63054841	NM_001017431.1	RBM3	NP_001017431.1	7380612	A	346
1.13E+08	XR_017837.1	LOC440926		4590593	A	143
53759100	NM_001548.2	IFIT1	NP_001539.2	6220673	I	108
89027425	XM_935827.1	LOC442609	XP_940920.1	5360300	A	2603
74319832	NM_004654.3	USP9Y	NP_004645.2	6290398	S	9257
1.13E+08	XR_018616.1	LOC400721		5890162	S	5744
37675384	NM_197953.1	CLEC7A	NP_922944.1	1090170	A	43
91208440	NM_003864.3	SAP30	NP_003855.1	2510133	S	873
33188426	NM_182659.1	UTY	NP_872600.1	5310196	I	4730
94538348	NM_053024.3	PFN2	NP_444252.1	4210246	I	620
70167417	NM_002357.2	MXD1	NP_002348.1	2260239	S	5318
7705854	NM_016142.1	HSD17B12	NP_057226.1	3290411	S	1747
54607092	NM_016047.3	SF3B14	NP_057131.1	1410315	S	199
89060764	XM_937928.1	LOC347376	XP_943021.1	5900072	A	1
75905486	NM_001033658.1	LOC283152	NP_001028830.1	4810324	A	314
62952497	NM_020397.2	CAMK1D	NP_065130.1	3930653	I	1347
1.13E+08	XR_017611.1	LOC653635		2480278	S	2417
4506102	NM_002759.1	EIF2AK2	NP_002750.1	1190349	S	2728
1.1E+08	NM_018688.4	BIN3	NP_061158.1	7550392	S	1578
29550859	NM_022130.3	GOLPH3	NP_071413.1	4780114	S	2120
50511938	NM_001002269.1	EXOSC3	NP_001002269.1	1980743	A	721
59933283	NM_001012391.1	RUNDC2B	NP_001012391.1	4780064	I	1140
89028499	XM_942939.1	LOC647499	XP_948032.1	6560128	I	589

1.13E+08	XM_927706.2	LOC644591	XP_932799.1	1510196	S	3719
4506516	NM_002923.1	RGS2	NP_002914.1	5690500	S	865
88953414	XM_934113.1	LOC653489	XP_939206.1	3890053	I	5975
89028131	XM_928866.1	LOC645895	XP_933959.1	7570673	S	83
4758439	NM_004877.1	GMFG	NP_004868.1	4250452	S	469
89059772	XM_928047.1	LOC644969	XP_933140.1	2600187	S	1
10092611	NM_016326.2	CKLF	NP_057410.1	2000551	A	174
91598583	NR_002949.1	NUDT16P		6420402	S	1354
1.13E+08	XM_927767.2	LOC653505	XP_932860.1	160494	S	3717
4505416	NM_000904.1	NQO2	NP_000895.1	870400	S	621
74316007	NM_022129.3	PBLD	NP_071412.2	1340474	I	2137
52352807	NM_014624.3	S100A6	NP_055439.1	1500553	S	527
22748870	NM_152411.1	ZNF786	NP_689624.1	4200541	S	3091
1.13E+08	XM_001126665.1	FAM101B	XP_001126665.1	2350093	S	5
1.16E+08	NM_182705.2	FAM101B	NP_874364.1	940246	S	292
37537688	NM_005649.2	ZNF354A	NP_005640.2	2850670	S	2312
1.15E+08	NM_002965.3	S100A9	NP_002956.1	830400	S	506
88957838	XM_498571.2	LOC440160	XP_498571.2	1190731	S	115
55770901	NM_024576.3	OGFRL1	NP_078852.3	2750315	S	1038
16418454	NM_052960.1	RBP7	NP_443192.1	4810131	S	421
87080804	NM_001039473.1	C22orf34	NP_001034562.1	5910682	S	1303
1.13E+08	NM_002856.2	PVRL2	NP_002847.1	2570544	S	1789
21389514	NM_144726.1	FLJ31951	NP_653327.1	2490437	S	2719
4809282	NM_002108.2	HAL	NP_002099.1	6660327	S	2301
45430056	NM_014960.2	ARSG	NP_055775.2	5420379	S	1739
1.19E+08	NM_005153.2	USP10	NP_005144.2	1980021	S	2458
89060605	XM_942501.1	CSF2RA	XP_947594.1	5340037	I	1791
40255228	NM_032121.3	DKFZp564K142	NP_115497.3	5700139	S	2093
58331103	NM_175876.3	EXOC8	NP_787072.2	5130491	S	4522
28559038	NM_004774.2	PPARBP	NP_004765.2	6760373	S	5590
1.13E+08	XM_001132265.1	DHRS8	XP_001132265.1	1570553	A	1604
1.13E+08	XM_001127825.1	LOC728575	XP_001127825.1	5270575	A	224
31881791	NM_181657.1	LTB4R	NP_858043.1	3120735	S	2942
40806188	NM_000189.4	HK2	NP_000180.2	4760768	S	6532
58372151	NM_001011538.1	LOC402176	NP_001011538.1	4050056	S	1350
38016936	NM_014916.2	LMTK2	NP_055731.2	4050040	S	5327
5454101	NM_006342.1	TACC3	NP_006333.1	2630544	S	2550
34098964	NM_015364.2	LY96	NP_056179.1	6980474	S	505
37704380	NM_004048.2	B2M	NP_004039.1	6290114	S	402
65507252	NM_018169.2	C12orf35	NP_060639.2	3310446	S	5367
27735098	NM_173497.1	HECTD2	NP_775768.1	2230408	I	1864
67782329	NM_001024843.1	TNRC6B	NP_001020014.1	2600386	A	15415
55769563	NM_003439.1	ZKSCAN1	NP_003430.1	1580187	S	5035
50355969	NM_000570.2	FCGR3B	NP_000561.2	3990360	S	1450
59938775	NM_001011666.1	CREB5	NP_001011666.1	4220026	I	40
94538364	NM_014339.4	IL17RA	NP_055154.3	3870110	S	2932
19743913	NM_130843.1	PTPRN2	NP_570858.1	6580717	A	4200
38176162	NM_018434.4	RNF130	NP_060904.2	5670603	S	1503

50593000	NM_003282.2	TNNI2	NP_003273.1	6350142	S	274
21614543	NM_002964.3	S100A8	NP_002955.2	6280576	S	253
9257219	NM_000804.2	FOLR3	NP_000795.2	2230601	S	759
71143157	NM_001029862.1	ANKRD30B	NP_001025033.1	1690184	I	2942
34304373	NM_003885.2	CDK5R1	NP_003876.1	770484	S	3133
41350336	NM_003263.3	TLR1	NP_003254.2	5080398	S	2736
66529202	NM_020954.2	KIAA1618	NP_066005.2	4570561	I	4774
78190467	NM_000987.3	RPL26	NP_000978.1	2000025	S	57
40807473	NM_000608.2	ORM2	NP_000599.1	6370202	S	473
55775476	NM_014985.1	CEP152	NP_055800.1	3930370	S	4067
88986346	XM_933119.1	LOC653316	XP_938212.1	7320170	I	1162
75813617	NM_001033556.1	PPM1B	NP_001028728.1	3450026	I	1448
56550115	NM_001300.4	KLF6	NP_001291.3	5260397	I	1330
29029598	NM_006639.2	CYSLTR1	NP_006630.1	4810204	S	1218
89027700	XM_927280.1	LOC644033	XP_932373.1	4290669	S	283
58761509	NM_024640.3	YRDC	NP_078916.3	1340491	S	1263
4503240	NM_000896.1	CYP4F3	NP_000887.1	650164	S	4625
54144630	NM_030948.1	PHACTR1	NP_112210.1	3800095	S	1803
24308385	NM_052937.1	PCMTD1	NP_443169.1	240037	S	3666
27734690	NM_173669.1	FLJ34047	NP_775940.1	4850735	S	2829
71772593	NM_001032.3	RPS29	NP_001023.1	5960026	I	194
8922429	NM_018097.1	CEP27	NP_060567.1	3850524	S	1256
89050223	XM_928041.1	LOC644964	XP_933134.1	70221	S	430
47078236	NM_004486.4	GOLGA2	NP_004477.2	5390445	S	3921
89029829	XM_928180.1	LOC643284	XP_933273.1	4230615	S	68
18375674	NM_015542.2	UPF2	NP_056357.1	2230307	I	5
31455612	NR_001445.1	RN7SK		5080673	S	257
33188430	NM_007125.3	UTY	NP_009056.3	5420224	I	6354
4505316	NM_002480.1	PPP1R12A	NP_002471.1	4180280	S	4479
55743117	NM_012233.1	RAB3GAP1	NP_036365.1	6330561	S	3989
39932582	NM_017970.2	C14orf102	NP_060440.2	3140358	I	161
19743870	NM_133279.1	FCAR	NP_579813.1	6860239	I	1361
45580712	NM_130771.2	OSCAR	NP_570127.2	2190609	A	983
41393558	NM_183416.2	KIF1B	NP_904325.2	3170719	I	5646
7662017	NM_015153.1	PHF3	NP_055968.1	2510687	S	6079
89027419	XM_935802.1	LOC653829	XP_940895.1	7100044	A	3711
88702790	NM_002393.2	MDM4	NP_002384.2	4490671	S	2032
24475703	NM_025209.2	EPC1	NP_079485.1	2680010	S	2756
62739174	NM_003610.3	RAE1	NP_003601.1	4850138	A	1662
47933391	NM_004288.3	PSCDBP	NP_004279.3	5390195	S	1719
89035399	XM_931879.1	LOC643882	XP_936972.1	3180482	S	1616
38570153	NM_012279.2	ZNF346	NP_036411.1	3420373	S	2662
31982907	NM_130469.2	JDP2	NP_569736.1	3180379	S	1608
68226419	NM_001024938.1	SLC2A11	NP_001020109.1	2750091	A	1397
58531218	NM_001011547.1	SLC5A9	NP_001011547.1	70270	S	2889
1.13E+08	XM_001128558.1	LOC730740	XP_001128558.1	4920408	S	228
5032058	NM_005621.1	S100A12	NP_005612.1	10279	S	241
31377636	NM_052862.2	RCSD1	NP_443094.2	3140280	S	2692

27437033	NM_172246.1	CSF2RA	NP_758449.1	4150246	A	1244
1.18E+08	NR_003264.1	SDHALP1		6420674	I	10150
46559738	NM_207337.1	AMN1	NP_997220.1	3990719	S	1629
13194198	NM_023003.1	TM6SF1	NP_075379.1	10541	S	964
13129023	NM_024054.1	C7orf25	NP_076959.1	4570397	S	1351
1.13E+08	XM_371820.5	FLJ90086	XP_371820.5	5050181	A	4017
52851404	NM_003898.2	SYNJ2	NP_003889.1	5900592	S	6404
4507284	NM_003765.1	STX10	NP_003756.1	3190681	S	685
62865648	NM_001017368.1	RFFL	NP_001017368.1	5870551	A	3411
21361994	NM_024876.2	ADCK4	NP_079152.3	1690221	S	1907
68160936	NM_005082.4	TRIM25	NP_005073.2	2850576	S	5462
24233529	NM_153697.1	ANKRD44	NP_710181.1	4730377	S	2988
41393567	NM_014320.2	HEBP2	NP_055135.1	5090184	S	804
65287716	NM_001013703.2	EIF2AK4	NP_001013725.2	3370471	S	5290
78190459	NM_000978.3	RPL23	NP_000969.1	4120707	S	321
50355980	NM_032576.2	CYorf15B	NP_115965.1	4730458	S	4976
88987903	XM_936748.1	LOC647691	XP_941841.1	2970600	S	3
23510443	NM_006573.3	TNFSF13B	NP_006564.1	460608	S	634
22219473	NM_003824.2	FADD	NP_003815.1	1940035	S	1558
56682942	NM_130782.2	RGS18	NP_570138.1	1240438	S	1787
73747916	NM_018833.2	TAP2	NP_061313.2	1780528	A	1448
31543155	NM_032882.2	PNMA6A	NP_116271.1	6760270	S	1892
89047114	XM_928156.1	LOC643509	XP_933249.1	70286	A	2564
45935370	NM_002727.2	SRGN	NP_002718.2	650541	S	482
22538469	NM_005442.2	EOMES	NP_005433.2	6760075	S	2643
23110934	NM_148976.1	PSMA1	NP_683877.1	20451	I	233
46389561	NM_207168.1	ENSA	NP_997051.1	1110685	A	236
13376242	NM_024834.1	C10orf119	NP_079110.1	4830192	S	4030
31077212	NM_001530.2	HIF1A	NP_001521.1	2850288	I	2496
61742780	NM_019610.3	RBMXL1	NP_062556.2	5080093	S	3210
20070234	NM_006495.2	EVI2B	NP_006486.2	2030593	S	1401
38683859	NM_003749.2	IRS2	NP_003740.2	6980095	S	6369
4502094	NM_001150.1	ANPEP	NP_001141.1	1770605	S	3149
1.13E+08	XM_001131044.1	MBOAT1	XP_001131044.1	5810731	A	2451
13259519	NM_006037.2	HDAC4	NP_006028.1	5960341	S	8280
40217797	NM_152655.2	ZNF585A	NP_689868.1	460504	I	5
1.16E+08	NM_148962.4	OXER1	NP_683765.1	510491	S	1501
85838510	NM_006609.3	MAP3K2	NP_006600.3	2320296	A	3192
58331201	NM_001010935.1	RAP1A	NP_001010935.1	3060692	I	188
21361096	NM_004125.2	LOC552891	NP_004116.2	5820176	S	337
55741708	NM_021239.1	RBM25	NP_067062.1	840075	S	4100
23238212	NM_005717.2	ARPC5	NP_005708.1	3930243	S	1767
61966848	NM_001013699.1	LOC440093	NP_001013721.1	4730100	S	307
70608079	NM_001002009.1	NT5C3	NP_001002009.1	3780689	A	962
55743129	NM_001007074.1	RPL32	NP_001007075.1	2340373	I	1
90186266	NM_024669.2	ANKRD55	NP_078945.2	4670056	S	1969
1.13E+08	XM_942212.2	LOC649821	XP_947305.1	5550364	A	192
4757873	NM_004334.1	BST1	NP_004325.1	4920243	S	1081

34147635	NM_006807.3	CBX1	NP_006798.1	2450446	S	1745
89026887	XM_935752.1	LOC641978	XP_940845.1	6040114	S	7
88983545	XM_926814.1	LOC653158	XP_931907.1	3460735	A	194
51972215	NM_001004322.1	FLJ38717	NP_001004322.1	990100	S	2559
90577173	NM_014358.2	CLEC4E	NP_055173.1	940754	S	1426
7705482	NM_016407.1	C20orf43	NP_057491.1	4050537	S	1458
89276754	NM_002760.3	PRKY	NP_002751.1	2900048	S	6641
68160956	NM_003264.3	TLR2	NP_003255.2	1260008	S	3246
15529971	NM_033412.1	MCART1	NP_219480.1	1980521	S	1556
10092618	NM_020529.1	NFKBIA	NP_065390.1	4570154	S	992
37059785	NM_018334.3	LRRN3	NP_060804.2	7380181	S	2676
20336283	NM_052961.2	SLC26A8	NP_443193.1	6650717	A	2874
55741472	NM_020760.1	HECW2	NP_065811.1	3140541	S	6706
34328944	NM_004202.2	TMSB4Y	NP_004193.1	4150095	S	1360
88953511	XM_934651.1	LOC653596	XP_939744.1	430209	I	6963
98986451	NM_023075.4	MPPE1	NP_075563.3	780168	A	2114
83722281	NM_198578.2	LRRK2	NP_940980.2	1450523	A	7502
73747914	NM_000544.3	TAP2	NP_000535.3	2970240	I	5257
89026533	XM_942096.1	FLJ40722	XP_947189.1	2600632	I	2233
27502389	NM_001562.2	IL18	NP_001553.1	5890196	S	1038
56788365	NM_007166.2	PICALM	NP_009097.2	1580364	I	1560
54112402	NM_017780.2	CHD7	NP_060250.2	4640750	S	10019
14589888	NM_001792.2	CDH2	NP_001783.2	4780376	S	4032
89041683	XM_935186.1	LOC440456	XP_940279.1	7510424	I	895
88942472	XM_930480.1	MGC12760	XP_935573.1	4490338	I	1659
37622904	NM_006242.3	PPP1R3D	NP_006233.1	6400064	S	3031
51243031	NM_001003712.1	OSBPL8	NP_001003712.1	3830138	A	6974
1.13E+08	XM_001133926.1	SLC25A20	XP_001133926.1	770255	I	561
29171680	NM_001557.2	IL8RB	NP_001548.1	2710437	S	2541
45333920	NM_024095.3	ASB8	NP_077000.1	4280114	S	2124
37574625	NM_006707.2	BTNL3	NP_006698.1	3180082	A	1714
1.09E+08	NM_013364.3	PNMA3	NP_037496.2	2490113	S	3474
17921988	NM_006000.1	TUBA4A	NP_005991.1	380731	S	1391
31377615	NM_138287.2	DTX3L	NP_612144.1	2850100	S	4588
89040690	XM_936461.1	LOC647389	XP_941554.1	6040181	S	837
22547162	NM_024586.3	OSBPL9	NP_078862.2	6020685	A	2397
9966838	NM_020370.1	GPR84	NP_065103.1	2370576	S	1181
95006998	NR_002189.2	SUMO1P1		5340050	S	447
54607115	NM_198271.2	LMOD3	NP_938012.2	4250609	S	3155
39777600	NM_003245.2	TGM3	NP_003236.2	3440754	S	2485
19923725	NM_002514.2	NOV	NP_002505.1	6420367	S	1974
30102945	NM_178231.1	ALS2CR14	NP_839945.1	4900215	S	830
62912451	NM_016824.3	ADD3	NP_058432.1	1690273	I	2132
1.09E+08	NM_025180.3	CEP63	NP_079456.2	1090300	S	2450
4504152	NM_001511.1	CXCL1	NP_001502.1	6130576	S	792
95007029	NM_001040616.1	LINS1	NP_001035706.1	730491	A	1016
61966890	NM_001013722.1	LOC441193	NP_001013744.1	2060113	S	1600
19923652	NM_033222.2	PSIP1	NP_150091.2	1240541	I	3023

20544150	NM_016587.2	CBX3	NP_057671.2	4880020	I	37
13899331	NM_031476.1	CRISPLD2	NP_113664.1	6060224	S	4224
88944406	XM_931214.1	LOC653105	XP_936307.1	3800139	I	1
19920316	NM_006825.2	CKAP4	NP_006816.2	6770348	S	2458
56090219	NM_001007535.1	dJ341D10.1	NP_001007536.1	4180402	S	1152
24432071	NM_145032.2	FBXL13	NP_659469.2	5050653	S	2503
50345994	NM_003972.2	BTAF1	NP_003963.1	1820546	S	6646
38201610	NM_170607.2	MLX	NP_733752.1	5820021	A	2409
88758607	NM_012428.2	NPTN	NP_036560.1	5360338	A	1992
88943484	XM_497711.2	LOC653604	XP_497711.2	730468	S	12
18105038	NM_001866.2	COX7B	NP_001857.1	70722	S	141
42476163	NM_002697.2	POU2F1	NP_002688.2	3850554	S	2380
1.13E+08	XM_001134256.1	LOC729853	XP_001134256.1	5720204	S	1261
42544180	NM_001721.4	BMX	NP_001712.1	5560451	A	1987
74272286	NM_004994.2	MMP9	NP_004985.2	4150224	S	2049
47106078	NM_017839.3	AYTL1	NP_060309.2	5670100	S	1697
67189969	NM_000433.2	NCF2	NP_000424.2	7050360	S	2056
32563547	NR_001545.1	TTTY15		2570082	S	4933
46249360	NM_145686.2	MAP4K4	NP_663719.1	2450170	A	6802
15718674	NM_001629.2	ALOX5AP	NP_001620.2	4180411	S	664
73747924	NM_148954.2	PSMB9	NP_683756.1	6130669	A	767
33469923	NM_182802.1	MCM8	NP_877954.1	6270082	A	3364
47419935	NM_003137.3	SRPK1	NP_003128.3	3460674	S	4153
31563435	NM_144673.2	CMTM2	NP_653274.1	5080300	S	763
33285009	NM_022746.2	MOSC1	NP_073583.2	1980082	S	1680
50284695	NM_182757.2	IBRDC2	NP_877434.2	5910037	S	4431
21389370	NM_144590.1	ANKRD22	NP_653191.1	4200543	S	609
68303646	NM_001025077.1	CUGBP2	NP_001020248.1	6110672	I	2065
89030416	XM_938919.1	LOC648226	XP_944012.1	3780189	S	2404
45827727	NM_005980.2	S100P	NP_005971.1	2640609	S	378
21314634	NM_002784.2	PSG9	NP_002775.2	1050465	S	404
38026951	NM_033656.2	BRWD1	NP_387505.1	430400	I	12653
25777623	NM_172027.1	ABTB1	NP_742024.1	5670424	A	1913
40807350	NM_152313.2	SLC36A4	NP_689526.2	2350195	S	2320
40255235	NM_012482.3	ZNF281	NP_036614.1	6940113	S	2768
13375790	NM_024595.1	C1orf108	NP_078871.1	1690673	A	1613
20127493	NM_006305.2	ANP32A	NP_006296.1	3360546	S	987
1.13E+08	XM_939954.2	LOC388789	XP_945047.2	1440259	A	219
96304477	NM_020686.4	ABAT	NP_065737.2	1030685	A	4303
1.13E+08	XM_371152.3	ZNF486	XP_371152.3	2070471	A	3074
1.14E+08	NM_020920.2	CHD8	NP_065971.2	5310433	S	7016
1.13E+08	XM_944290.2	KIAA0492	XP_949383.1	1090609	A	5727
34147390	NM_030578.2	MGC4093	NP_085055.1	270519	S	895
22165432	NM_145869.1	ANXA11	NP_665876.1	7380450	I	338
6912575	NM_012387.1	PADI4	NP_036519.1	5310653	S	2163
33359218	NM_182686.1	KIAA0319L	NP_872628.1	150392	I	5065
38505160	NM_198795.1	TDRD1	NP_942090.1	270301	S	3989
1.15E+08	NM_023001.2	ARID4A	NP_075377.2	3830228	A	4318

33942075	NM_145913.2	SLC5A8	NP_666018.2	4010021	S	3702
77917615	NM_019000.3	FLJ20152	NP_061873.2	1230022	S	2592
1.13E+08	XR_017962.1	FLJ14107		2900008	S	1503
71043965	NM_030636.2	KIAA1706	NP_085139.2	6250685	S	4375
20336255	NM_138687.1	PIP5K2B	NP_619632.1	6370093	I	1324
38016920	NM_006861.4	RAB35	NP_006852.1	5340243	S	2738
5016091	NM_001621.2	AHR	NP_001612.1	840189	S	4990
60460880	NM_080603.3	ZSWIM1	NP_542170.3	1260082	S	2094
96975022	NM_182703.3	ANKDD1A	NP_874362.3	3290296	S	2807
17986253	NM_080424.1	SP110	NP_536349.1	10546	I	1934
16554582	NM_003389.2	CORO2A	NP_003380.2	4590554	A	1976
88953548	XM_930995.1	LOC653086	XP_936088.1	6860064	I	6448
29171681	NM_002186.2	IL9R	NP_002177.2	290424	A	1990
40254907	NM_017833.2	C21orf55	NP_060303.2	4390136	S	1982
1.24E+08	NM_001080951.1	PLAGL1	NP_001074420.1	2230475	A	2085
33859844	NM_013361.2	ZNF223	NP_037493.2	4760767	S	2321
57163721	NR_002206.1	GTF2IP1		6200438	S	2314
51663330	CR738550			5810274	S	470
21757894	AK097979			5820441	S	2069
16550235	AK055497			2470440	S	3173
4739546	AI655567			2600138	S	157
2079612	AA417811			2320722	S	257
27835509	BX108917			1050543	S	489
14432774	BI026144			2810653	S	145
22665185	BU151653			1170133	S	539
80547114	DA385619			3990497	S	469
81140489	DA572426			1260750	S	447
21985501	BQ777029			7100053	S	344
21733442	AL832858			2100576	S	2072
38512164	BC062546			5570224	S	2313
23238724	BU587445			5130747	S	1
27879393	BX113731			4880541	S	50
19044878	BM723547			6200424	S	592
51656959	CR746297			4250470	S	589
9866586	AV645572			6960445	S	241
15330256	AW020492			4210128	S	313
34530644	AK124771			6510626	S	3322
21178167	BQ439091			5900204	S	544
19016391	BM703133			4250026	S	218
27878684	BX111927			3940719	S	35
21848608	BQ709709			6450368	S	471
1760761	AA179392			6020577	S	30
19722737	BM997836			3850634	S	387
30219749	CB963631			240102	S	279
22364332	BQ948854			4040075	S	765
28277084	BC045657			2030326	S	3657
19760814	BQ025535			7000411	S	315
19029786	BM716528			650431	S	466

27829802	BX099079		3850196	S	533
6660861	AW273831		20070	S	171
24041994	BU857004		4220468	S	174
5100912	AI738931		6200168	S	251
34194422	BC037864		4810082	S	4220
27835769	BX109404		2190176	S	112
12362621	BF945346		770500	S	452
27844508	BX100504		7160598	S	193
80823217	DA395916		4590053	S	550
3183900	AJ227862		670441	S	220
2785000	AA744250		4640685	S	189
34526117	AK129555		6480148	S	405
21733307	AL832727		1500619	S	4727
24732379	CA396207		3610255	S	536
18978757	BM668860		6110762	S	443
27825094	BX092006		1170193	S	293
15332023	BI492679		3390333	S	304
30981510	CD238045		1990097	S	101
55940382	AV651069		7650746	S	216
4888225	AI678043		3840504	S	131
16179735	BI915787		4150327	S	129
27840427	BX117171		1430504	S	334
3096496	AA938457		5690064	S	238
1156211	N35069		830553	S	137
5741141	AI948831		650593	S	209
8907626	BE220308		5560719	S	359
16550218	AK055485		3390348	S	2006
4500184	AL049390		4220301	S	1933
8611465	BE148741		7510523	S	218
8655615	AL359560		1980242	S	1386
1695587	AA134590		3450309	S	479
21753601	AK094521		2900132	S	960
34530059	AK124299		2190259	S	2411
10437827	AK025332		3850020	S	1748
82859048	DR977977		50433	S	10
31874202	BX538075		4220050	S	2335
15948260	BI836710		110138	S	574
1802546	AA204695		6510753	S	212
31811548	CD642055		4890674	S	504
10722614	AV705309		5960528	S	357
10217337	BE796139		5220291	S	813
27878392	BX111162		5050112	S	380
4507744	NM_003329.1	TXN	7610747	S	23

SEQUENCE	Chromosome
GGAGCCCTGATCCTGCCACTGCACTCCAGCCCGGGCGACAGAGTGAGACC	7
GTCTTCACATCTACCTTTCTATGTCACATGTGCAAGATGGTTGCTCTGCC	
GGGAGGAAGGAACGAGGCAAGGAGCTAAAGCAGCGTGCGTTCAGCCCTGG	
GCCTCATGTCTCTGCAGGGTGGACATAGCTCTAACTCTCTGAAGCTGAGT	14
TGGGATTCGGCCTCTGGAAAGTGGTGGTAGTTCCAGATTTATGTGAATGT	
TGGCTAGAATATGGCAGTAACTACAAGGCATGTTCTGCTCTGGCACGAAG	1
TAAGATCATGTCACTGCACTCCAGACTGAGCAACAGAGTGACACTTTATA	
GTCTTCCTTGAATACCCTGGGGGAAGAGAATCCTCCAGTCCTTGGGATGC	13
GGGGTAGAAGTTATGCTTGTGTTTCTCCAGGCCAAAATCAACAGCTGATC	
AGGACAACTTTTAGCCGGCAGCCCAGACCAGCGCGGCACCTGTCTCCGGA	7
GGGGTGAAGTGGAAAATCCAGGGCAAGTTTCGTGGAGGAAGTGATGCCTG	
GCTTTACGTTGCCTCATGTGAGTGCTTCATAGCAGGGTGTGACGCTGGCC	16
GCCAAATCTGAGCATCAGAAGTCTTTCCAGTCTACCTGATGCATGATCTC	11
TGCCTCCTTCCAGTCAGCCTGTTAGTGACCCCAGAGATGGGCTCCGAGTT	
CCCTGGAAAGCTCCCCGACAACCTCCACTGCCATTACCCACTAGGCAAGT	
TTGCGGACCTGGCGGAGATGCGCGTTCGCTTCGATTGTAGGCCATTCCTT	
GAGATGACCTCCACCCAGGTTTCACTTCCGTTGTACCCAGCTCTCGTCTT	
CCGTGTGTCAGGGAGCTGAGTGGAGGCTGAAAGAGGATGGAACACTTCCC	
GCAGGCTTCTCACTGAGGCCACTGTAAATCAGTATTCTCCTCCTTGCAGC	
GCAGCATAGAGGGAGAAGAACCTGTCTGTCAACCCCACACACGCTTTCCC	
GGCTCACCAGAGTACCCAGAAGAATCAGTATGGAATTAGAGGACAGTGGC	
GATGGAAGAGGCCTGTACAACACTCATATAACTTCGGCATTGCCACCTTC	
TCGGTTATGATGTAGGGGGAAAAGCAGCAGCCTCGAAGCCTCATGCCAAC	
GCAGATCGAGAGCAAGACTGCTTTTCAGGAAGCCTTGGACGCTGCAGGTG	
CGCCACACGTAACTGAGATGCTCCTTTAAATAAAGCGTTTGTGTTTCAAG	19
GCCCTGGACTTGGTACAGTTTCAGGAGCGTGAAGGACTTAACCGACTGCC	9
GCGCTCGCTACCAGAAATCCTACCGATAAGCCCATCGTGACTCAAAACTC	19
CCGTCGGAGACCAGATGACCTGTTAGATGGCTAGTCCTGTATAACTCGAC	3
GCCTTCAAGCACAAAGTGATGAATGACTGCCTTCAAGTCTCAAGAAAACA	9
CTGGTTCTTCAGTGCTGCTTACTATTTTGGTAACTGGGCCTTTCTTGGGG	6
CCACGCGCGAAAATTCGGCCAGGGTTCTCGCTCTTGTCGCGTCTGCTCAA	
GCAGCTCCTGAAGGTCTGTGTTGCACTGTCACCAGTCTCAAGCTATGCCT	9
GTACTTATTTTGCATTTTTGGTCATGGGTGGGAATGGGGTGGGTGCCTAC	X
TTGAGACCAGCCTGGGTGACACAGCAAGACCCCATCTCCACAAACGTTTT	19
TGGAACTGAATCTCATGTGAGGCTACCAGGACCAAGACCCAATCGTCCTG	11
CGTCTTCAAGGACCCAGTTGCTGATCCCAACAAAAGGTCCAAAAAGGGCC	
AAGTGGAGGTTGCAGTGAGATCGCGACACTGCACTCTAGCCTGGGCAGTA	13
CCACAAGTTTCACAGTCAGCGGCACGGGAGCAAGAAGGATTGAGGCTGGG	7
GTGGTCTTTCTGGAGAGGTTATAGGTCACTCCTGGGGCCTCTTGGGTCCC	10
CACTCCAGCCTAGGCGACAGAGCAAGACCCCATTCTCTTACAAAGTCTCT	3
GGAAGTTGCCCTTACAGGTGGGACCTTTTGTGTTAATCTGTTTTCTCCCC	1
GGCGCAGGTGGGCTGGACTTCTACCTGCCCTCAAGGGTGTGTATATTGTA	11
TGTGGACTTGAGCAGCGGTGACTTCGCAAGCAAATGGATTGTCAGGCTTG	7
CTGGTAGCCTTCAAGTGGCCATGGTGGGAGCATTGACCCAGGGAAATCCA	20
CAGCCTGTGTGTGCACGCCCCATGAGCCGAAAAGTGGGTCTTATGTTTTC	6
GGGGCATGTTGTGTCATGTAGTCAGCCACTTATGCACCAATGTGAGGAAA	14
GGCTCTGTCAGCCATGTGACTGTAGTCCACATGGAGCTCTCAGCATACCG	

CCCAGCGCTTTGGCAGGCCAAGGCGGGCAGATCGCTTGAGCTCAACAGTT		1
GGGGGCTTGTCACCTCCCCCACCTTCTTCCTGAGTCATTCCTGCAGCCTT		1
CGACTTTCCCGATCGCCAGGCAGGAGTTTCTCTCGGTGACTACTATCGCT		1
CACCTCAAAAACTGGTAGAATCTGAGTGTCTTCAGGCCAACCTGGTGGAA		17
GCTACTCAGGTGGCGGAGGTGAGAGGATTGCCCGAACCCAGGAGTCAAAA		5
AGAGGTGCGGTCGATCGGCGAAGAAACCAGGCCAGACCGTAGTGCCTTAT		
CACGAGAACATGCCTCTCGCAAAGGATCTCCTTCATCCCTCTCCAGAAGA		1
TGTTCAGCGCTTCGAAATATTCCTTCATCAATGACAACGCAGCTTCTGGT		1
AAGTAGACACTTGGTCTTTCCCAGTTGCCAGCATCCTGCAGTCCTCCTGC		16
ATTCACCCCAGAGGAAGGGAAAGGCTACCGCGAAGAGGTCTTGACCGTGA	X	
CAGTGACCAGGTTACCTACAGTAGTGACTGCCATCTGTGCTCCAACATTC		
CAAGTTTACTCCTTCTGTTTTGAGTTTTGTAGCAGTGTACCCACGCTGGG		13
GCTACCATGACCAATGTCTTCTTAGACCGTGTTTCCAGGAGTGTCTCAC		14
TGCCCCCTGACTGATAGCATTTCAGAATGTGTCTTTTGAAGGGCTATACC		3
CAGGTGTAGGTATAGGAGGTCAAGAAAAGGAGTTCGGTAAAGGGCATAGC		16
GCTCTGCACCATCCCTCACCCAGACCGTAGACACCAGGGAACCACATCTA		22
CCACGCATGACCCACACTGACCACACCCTGCCCTCTTCGGTGACATTCTT		16
CACTGCAGATGCGGTCACCTGACTGACCACCTGGGTTTTCTATTACCAGT		1
CCTGGGAGCTTTTGTAGAAGTCACATTCCTGAACAGGATATTCTCACAGC		7
CATGAGGGGCCAAGACACTGCCTAAGGTGTGGGAGGGACTGGCTGAGATT		17
ATGGCCCGTACTAAGCAGACTGCCCGCAAGTCGACCGGCGGCAAGGCCCC		1
GGAGTTGGTCTTAGCCGCTGCAGGAGCCCTTCTTTTCTGTGGATTCATCA		
CTGTTTCTGCAGGGGATGATATTGGTGAGTTGCCAAAGAAGCAATACAGC		17
AGATCTGGGTGCCTTGGGAGAACCAGTCCTTCCTTTTGACCCACCCCAGG		1
GCTGGGGCACATTTCACATATCGACTACCTGAGAAATTGCTTTGTGTCCC		12
AACCACCGCTGCCGCCTCCTTCTTCTGTCACTCCTGGTGCTGCACTGTGT		14
ATGTTAGGAAATGGAGAGGTATAGAGAGAGCAGGTTCCATAGCTCAGCAC		16
GGGCCAAAGAGGTGTTAGGTTCAAAGGCTGCGAAGAGAATAGGCTACTGG		7
ACCGGCTACCCAAGGACTTTGTAGACCTCTCTACTGGGAGGGACGCTCAC		10
CCATCTTGCTTTCCCCATTGGAACTAGTCATTAACCCATCTCTGAACTGG		8
GCTTGCCCCACTTCAGAGGTTTTGGGGTTCAGGGTGCTGTGTCTCCCCTT		19
CAGGACAGAGGCAACGTGGAGAGGCTGAAAACAGTGCAGAGACGTTTGAC		20
GACAAGAGTTCTGGGTACAATTTTGGGATCTAGTTCCCCTGGAAAAGCTG		7
TGCGCGCACCCTCTGCCCTGGTCCATCGCTGCCGCCTCCTTCTTCTGCAG	X	
GGACCCCGACCTCCAGTTGCCTACAATTCCAGTCGCTGACTTGGTCCTGT		9
CCTGCTCGGTGCACTCTCTGCAGGACTTTCCCAATCACGGGACTCAGGTA		
ACCCGGCCATCCATTTTTCTTCCATGCATCCTGGCTGGGCCGACACCCCA		13
GCTGAATCTCCCAGAGAGCTCACACTGGCCAGGAATGCTGAGAGTAGCAG		8
AACTGAGAACAGTTGACCGCAAGCTGAAACTGGTGTGTGGAAGTCACAGC		1
CCACACACTCACCACTCCCAGCTTCTCGTGTCCAGTGAAACCCCTGAACC		
TTGATGCAAGCCCTAAACACTCTTTCGACTCCAGAGGAGAAGCTGGCAGC	Y	
AGGGAGACAAGGAAACGGGCACAGCGAAATCCTGGTGAAGAGCAAAGCAG		2
CTTCTCTGGACATGCGCGTTTGAGGGTGGAGGGGTCCTGTAAGGTGCTTC		
CCAATGGAATGTTCCCAGGTGACAGCCCCCTTAACCTCAGTCCTCTCCAG		17
GGTGGATAAGGTGACTCAAAGGAAAGACGACTCAACCTGGAACTCAGAGG		2
GGGAGGGTAAATAAACCACTGTGCGTCTTGGTGTAATTTGAAGATTGCCC		5
CAGACTGGGAACAGGGCCCAGGAATCTGTGTGGTACAAACCTGCATGGTG		5
GAGATGATGTGTGGTCCCACTGAATGGTATCAGAGTTGTAGTCCTAGCTC		

CTCCCTCTCCAGGGAGCGCATAGATACAGCAGAGCTCACAGTGAGTCAGA	9
ATGCTGGTGTAGTCTATACATCAGATTTGTTTGCTACACAAGATCCTCAG	4
CAGGCCCCATTGCCTGGGGTTGTTACTGCTGAGATTAAGGGATGCTCGTC	7
CTCCTCTCCCATTTGTGGCAGTTTGCGTTTACTACCTCCCTCTGGCTGCC	20
CGGAACATATCCAGTACTCCTGGTTCCTAGGTGAGCAGGTGATGCCCCAG	6
TATTTCTGGCCCCCCAGTGTCAGCCCATTCATGACAAAGCTTGACCAGTC	1
GCAGCAGCCAATTACAGCCCCTTTTGTAGCCGGGCGTTCCTATGGTCAAA	17
TTTTTTGTCTGGAGGAATGGGGACACCAAAACTCATTTGGCAGCAGAGGT	2
GCCAAAGGGCTCACCTTCACATTGTTAGTTCATGACAGACCCAGGTGTGC	12
AGCCTGAGCTGCTGGAAACTATTCCCTATGAATTCATGGCATAATAGGTG	
GCCTATGTGCTAGCCCACAAAGAATATTGTCTCATTAGCCTGAATGTGCC	1
TGGAACGGAGACAGCATTGGGCTGACTGTGGGCATGAGGAGGGATAAGGC	
GAGTCTGCCTCCATGCTGCAGTGTTCGAGTGGATTGTAGGTGCAAGATGG	
GAAATTCTGCCTGAGGACAGCAGCCCAGTGCTTGGCGAGAGTTCCTGACA	22
TTGCCAGAGTTTTGCCTGCTGCTTTCCTCGTGGCCTCTTCTTGGGTAGTG	17
GGAGGATAGCTTGAGCCCAGGAGTTCGAGACCTGCCTGGGCAATATAGCG	2
AAGAGATATCCCCTTTCAGCCAGTGACTCGGTCCTCAGTTGCTGCTCCGG	9
CTCCAGCTCCAGCCTCATTTGCTTGAGACTTTGTGTGTATGGGGGACTTG	6
ATCCCCGTGAACCACCTCATAGAGGGCTCCCAGAAGTGTGTGGCTGAGCT	5
TTGGGCTTCACCCATAACCCACTGCCATGGCCGAGGAACGCATTGCTGTT	
TCCTAGCTACTTGGGAGGCTGAGGTGGGAGAATTGCTCAAGCCCAGGAGT	6
CGTCAGACTCCAGCAGCCAAGATGGTGAAGCAGATCGAGAGCAAGACTGC	9
TGCCGAGAGGTTGCCCTGATGGGCTCTGCCCGCTGGACATGTTCTTGAAT	1
GTAGTCTGCAGATGTGGATCCTAACTCCTGCGAGAAGTAACTCACAGTGA	
GGGTCTGGAGGGTTGCTATCACTTTGTCCAGCCCAAATACCTTCCTGGGC	3
CTTCCATTCTTGTCATTGGTCAATAGGGGAGGGTAGATTAGCTGCTCCAG	10
TGGTGTGGGAGTTGTCATGAGGCTGTGTTGAAGTGACTTATCTATGTGGG	4
GGGAGGCATGGGCCGGGGGCAGTTCTCTCCTCACTTGTAAACTTGTGTAG	
GGGCTGAGCACCTCTGGGTTGAATGGGAATGGGTCAGATTGGGAAGCCTA	1
TCCAACCCTATCAAATAATGAACTTCTCAGAGAGGCATCTGGGGTCCTGG	4
GGGGAAAAGGGTGGGATTGTCCAGCATGCTTGTATGTATATTTCAGAACC	12
GCTCTTCTTTGCCTCTGCTGGAGTCCGGGGAGTGGCGTTGGCTGCTAGAG	
GGGGTCTGTGAGAGTACATGTATTATATACAAGCACAACAGGGCTTGCAC	4
TTCCAATGCATCCCTGCTGATCCAGAATGTCACCCGGGAGGACGCAGGAT	19
GGGCAAACCCAAAGATGGAAAGTGCTTGTTGGGTGGGTAAGCACCACCTG	12
AGCTCACACCACTGCAGTCCAGCCTGGGTGACAGAGCGAAACTCCATCTC	
CAGTCTAATAAGCTGCTCTGCCCCTTGTGCTCAGAGTGGATGTTATGGGA	15
CTCCCACCAAGCCAGACACGTTGTAAATGCTGCTTCCTATTGGAGCCCAG	5
CACAGGTGACTGTGAAACAGATGCCCCTGGTCTTGCTTTCATCACTCTAG	10
CACGGAGCGGACTGAGGGGAGTGAGCGTTCTCTGCACCAAACAACAGTTA	1
GTTTCAGCGTCGAAAGTGGCCAGAAATGAAGAGACCTTCTTCCAAATCAC	20
AGTGCCCAGACTGTGTACAAAGACACATGTAATGGAGATTGTACAGGTTG	11
TGCGCCGACGATGCCCAGAATCCAGAACTTTGTCTATCACTCTCCCCAAC	1
TGGAATTTGCAAAGCTGCCAAAGCCTTAGACAAGCGCCAAGCCCATCTTT	
ACTCTGGCCTGGGTGATAGAGCGAGACCCTGTCCCCTGCCCCGCCAAAAA	
AGAGTCCTAGTGTGTGCCCTACCCTGGCTCAGGCTTCTGGGCTCTGAGAA	3
GCCGTCACCAAGTACACCAGCTCCAAATAAATGGACGCATGTTCAAACCC	6
CAGAAAGGGAGCCTCACGCTGGCTCACACAAAACAGCTGACACTGACTAA	16

GGAGCAGGTGGTCGGACAAGCAGTGGCACAGCAGATCCATGCCTTCTTCA		19
GAGACAGCCTGGGTGACAGAGTGAGACTCTATCTCAAAAGAGAAGACCCT		9
CGCCATCACAGCTGAACTTGTTGAGATCCCCGTGTTACTGCCTATCAGCA		2
TGCAGTGGATCCCAGCACTTTGGGAGGCCAAGGCGGGTGGATCATCTAAG		
TCAGCCATCTCAGCCCAGGCACCAGACAAGTGGGTGAAGAAGCCACCTTG		19
TGTATAGTCTGCAGATGTGGATCCTAACTCCTGCGAGAAGTAACTCACAG		16
GGGGAATCGGCGATGAACAAAGCAGATAGAAATCCCCACTCTTGTGGAGC		16
AGGCCAAGCTTGTTGGCTCATGCCTATAGTCCCAGCACTTTGGGAGGCCA		19
AGCACCAGTTCCGTCATTCTCGTTCCTGGGGAACCCCCACTCTGACCTGT		16
CACCCCCGAGCTCGCATGCTGTCACCCATTCCAGCCTAAATGTGACCATA		16
TCTATGCGCCTGTAGTCCCAGCTACCCAGGAGGCTGAAGCAGGATAATCG		2
CTGCACTGCGTGCTGGTGACGAATCCACATTCATCTCAATGGAAGGATCC		1
GGATCACAGTGACCTATAATGAGCCGTTGTGCTCCAGCCTGGGCGACAGA		7
GTTCAGGTGCATTGCGGCCTATCTGTACAAGGGCAATAACTGGTGGGCCC		
GCGAAAATTCGGCCAGGGTTCTCGCTCTTGTCGTGTCTGTTCAAACCGGC		14
ATGCTGGCCACTCTCAGTCCAGCGTTCCTCAGTAGTGAATAGCGAACCTG		1
CTGGCCATTCAGGCCCGAAAGCGGAGGCCGAAAAGAGAGAAACATCCGAA		7
GGGTGATGTCAGGGTGATAACCAGACATTCATGGAAAGGCATGCAGTTTG		5
GGCCGGGAAGCTAAAAGACAAGTCCAAAACAGACCTGGAGAGCTCCTTGG		
AGATGTGCAAGTGGCGAAGCTTGACCGAGAGCAGGCTGGAGCAGCCGCCC		
ATCCAAAGGCCTGGAACACAATGGTACTCAAAAACATAGCTGCCTCGGGG		14
GGGCCCGCTCGCATCCCCTTCAAGACGTTTTCCTACGTTTACCGCTACTT		5
GGCATGCTCTTTGGGAGCTGCACAGTTATGGGGAGGACTCCCACTGCTGT		9
CAGTCCCAGCTACCTGGGAGGCTGAGTCAGGAAAATCTCTTGAACCAGGA		2
GGACAGTGTCTTTTGACCAACCACCAGGCTTGAAGGCTACAAGAGACGAG		
GAGGTGGTGGGGACCAGGGCTATGGGAGTGGCAGGTATTATGACAGTCGA	X	
CGTACAAAGCAGACTGCCCGCAAATCGACCGGTGGTAAAGCACCCAGGAA		
GATGCAGCAAGAAGGAGCCATCTGCAATCCAGGAAGAAATTCCTTGCCAG		10
TTTTGGAGGTCATGGCGGGAGGATCACCTGAGGCCAGGAGTTTGAGGCCA		
GTGTAGCCAAGAGGACAGAATTACATGAATGACAGTGCCCAGAGTGACAG	Y	
AAGGTCATGCCACTGCACTCCAGCCTGGCTGACAGTGAGACTCTGTCTCA		
CCTAGCCCACATGATTTGACTCAGAGATTCTCTTTTGTCCACAGACAGTC		12
CAGATCTCAAGGTTGATAGTGGTGTTCACTAGGAGACGTGGAATTGAGAC		4
TGCCTTCTGAAACTGCTGCAGTTTCTCTTTGGGGGTATTGGTAGCCATTC	Y	
GGTGATGGGGTATGAACCTGTGTCTCCTTTGTATCCCTCTGTTGGTGGGG		3
TTTTGGGGGGCCATCTTCCTAATGCTACACACAGCCTGACAGGGGAGCAG		2
GTACCACTGAAACCCTGACCCAGAAAAGTGGCTTGCTTGGACACCCAGCT		11
TTCATCAGCCCGCCAAGATGGCGATGCAAGCGGCCAAGAGGGCGAACATT		2
ATACCATGGCTCGTACAAGGTGGACTGCCCGCAAATCTACCGGTGGTATA		
GCATGCCAAAGGGAGGCTGCAGCTGCCCGGGAAGAGGCTGAACAAGCTCT		11
GCATCTGCCGAGCACCTCCTGTTTGCCAGGCGCTTTCTATACTTAATCCC		10
ACACCAGCAATTGTGCCAAGGGCCATTAGGCTCTCAGCATGACTATTTTT		1
CGTTCTCTGCCTCACATAGCTTACAAGCCAGCTGGAGAAATATGGTACTC		2
TTGTGCTGTAACTTAGGCGAGCAGAGGAAACCCCTTCCTGGGCCTGCTGC		
GGGAAGAGGCTTGTGACCAGTACCAATCTTGAGTTTCTTTTTCTGTCCAC		5
CAGGTCAGTTGAGGCAAAAAACTATGGGTTTTTTCAGGTGAACCTCCCCC		9
GAGCTGGGGAGTTCAAGACCAGCCTGGGCAACATAATGAGATTTCAGCTC		16
AGGCCCTTTGCCTTCACAGAGAAGAGCAGACACTGCCATGGACCCGTCTC		

TGGTGATGAGGGTCTGTAGTCCCAGATACTCAAGAGGCTGAGGTGGGAGG		1
CCTCACTGTGTACAGAACGCAAGAAGGGAATAGGTGGTCTGAACGTGGTG		1
TCTGTCACACCACTGTACTCCAGCCTGACGGCAGAGCGAGACTCCATCTC		2
TGTGGCGCGCGCCTGTAGTCCCAGCTACTTTGGAGGCTGAGGCAAGAGGA		8
TCCTCAGGTGACTGGGGACTTGGAACCCTAGGACCTGAACAACCAAGACT		19
CAAAATGGCTGGCAAGCAGGCCATTTCAGCATCAGGCAAGTGGCTGGATG	X	
ACATCGCCCCTTCTGCTTCAGTGTGAAAGGCCACGTGAAGATGCTGCGGC		16
AGAGCCTCCCATTAAAGAGGAGTTAGAGCTGGGCCTTGAGGCTCTTCACT		3
TGGTGATGAGGTTCTGTAGTCCCAGATACTCAAGAGGCTGAGGTGGGAGG		1
CGGGAGGCACGGCCGAGATGTACACGAAGACAGGAGTCAATGGAGATTCT		6
GGAGTTCGAGACCAGCCTGGGCAACACAGCGAGAACTTGTCTCTATTAAA		10
GGAGTATGTCACCTTCCTGGGGGCCTTGGCTTTGATCTACAATGAAGCCC		1
TGGGAGGATCGCCTGAGCCCAGGGAGGTCAAGGCTGCAGTGAGCAATGAT		7
CTCCCACCAGCCTCTTAACTACTTGCCTGAGGAGGAGAGATGAAATTTCC		
CCTGGCTCCCGGCTGTGCGCTGAGGCTTTGTCCCCTGTCCTTTGGCGAAG		
TGTCACGTGATCATCAGAAACAGATATCCGAGTGGGTGGGGAGGTGTGCT		5
GCCTGTTATGTCAAACTGTCTTGGCTGTGGGGCTAGGGGCTGGGGCCAAA		
TCCTGCAGCTGTGTTTGAACAGGTCATTTACCATGCGGTCCTCCAGGTTC		
AAAGGAGAAAGCTCCTGCGGTTCGCCCAGAAACACTACACGCCTTCAGAG		6
GTGCAAACAGACATTCCAGAGAGCCTGATCCACATCCAGCAGCAGAGCCC		1
TGCTTTCTCCTGCAAAGTCACTCGAAAGGTGACTGGCGGAGGTGAGGCTG		22
CTGGACGGCTCCCTCATCTCACGGCGGGCAGTTTATGTGTGACCTGGACA		
GGGTGGGCACTTGGGCCTTGCGGGTGCATTCATGTAATCTGAGACTCTTG		5
GGGCAGTCAGTTTAGCACAAAGCAATACTAGGCTGAAGGAGAGACCTGAG		12
GTTAGCCTTGCATATCCCTTCTGTATCCTGTCCCTCCTCCACGCCGACCC		17
GATCAACCAGTACCAGGTGGTGAAACCAACTGCTGAACGCACAGCCTACC		
CCGTTGCACTCCAGCCTGGGTAACAAACTAAGACCCCATCTCTCTGTCTC		
GATATGAAAATTGGCTGGGCTTGGTGGCTCATGCCTGTGGTCCTAGCTAC	X	
GGAACTTGACTAAAGACCCCCAGTGTTGTAACGTACCTTTGTACCCAGAC		1
GGGTTCAAAATGATGGAAGGCCGAAGAGCAAGGCTTATATGGTGGTAGGG		17
CCTACCCATTGCCACTCTGTTTCCTGAGAGATACCTCACATTCCAATGCC		
AGAATCACCAGGATGGAACACAGGTCTTGCAGGATCACTTGAAACCTTCT		5
TGAGAGCGTGCTCCAGCCTGGCTCCCACAGGCAGCTTTAACCATTAAAAC		14
GAGCACAGAGGGGCTAGGGGCTGGTCCTTCTCGTTTGCTCTAGTCTTGCT		2
ACTGCATCCAAATCAACCATCTACAGGATACCCTCAGCATGTCGGTGCTC		4
GGATCTTCAGTTATTCGAGGGGAATGAGGCAGGTCAAGCCGATGCTAGCC		7
GGAGCAGAAGACTAAAGAGAACGAGGAGCTGACCAGGATCTGCGACGACC		4
TGGGAGCCCAGAAGAAATGCTCTTTTGCTTGGAGTTTGTCATCCTACACC		8
GTGGGATCGAGACATGTAAGCAGCATCATGGAGGTTTGAAGATGCCGCAT		15
ACGGAAGGATGTAAAGCCTCATCCTAGGAAGGAGCAAGCCCCTCTGCAAG		12
ATTGAGTAGGCTGAGGAAGAGGAGGGGTTGGTCTTGCTGTCTCAGGTGGC		10
CTAGGCACTTTAGAGGTGCACTTGCATGGCAGGCTGGGCCCCCTTTTCTA		22
ATCCTCAGGTTGTGGGGTATTTGTTTCTACTCCAGCCTGGCAGGCAAGGC		7
GAGCCCTCTAATGCTAGGAGTAGCAAATGGTCCTAGGAAGGGGACTGAGG		1
GAGCTCTCATGTTCTGCACCTCAGGAGGGAATTCAGCCTCAGTGATGTCC		7
CAGGGGAATCCACACAGCCCGCTCCCAGGAGCTAATGGTAGAGCGTCCTT		22
CCGATTCGCCATCACCCCCTACCCCAGAAATAGGACAATTCACTTCATTG		7
CCTCAGGATGGGGAGCTCACTCCGAGAACAGGAGAAATCAACATTGCAGT		5

GTGAGGGTGCAGAAGACCAGCAAGGAGCTGGAGGACATGAACCAGAAGCT	11
TAACTTCCAGGAGTTCCTCATTCTGGTGATAAAGATGGGCGTGGCAGCCC	1
GCCCCGTCTCGTGGGATTATTGATTCCTGATCCAAGAAGGGTCCTCTGGG	11
CATGCACCTACACTCCAGCCTGGGTGACAGGGCCAGACTCTGTGAAAAAA	18
CCCTCAGTGTGAAGCCTGTCGTGTTCTCTCCCCTTGCACTGGTCATCAGT	17
GTGAGTGTATGATGTAGGTAAAAATATATACCTTCGGGTCGCAGTTCACC	4
CAAGGCAGCAGTGAGCCATGATCACACCACTGCACTTCAACCTGGGGGAC	17
CTTCCCTTTTGCGGCCATCACCGAAGCGGGAGCGGCCAAAATGAAGTTTA	17
GCTGTTCCTTAGGGACACCAAGACCTTGATGTTTGGTTCCTACCTGGACG	9
AGGCAGACTGAGCATGCCCATCAGTTTCCTATTGCTGCTTCCATCCCTCG	15
CAGTGGCTACCACCTGTAATCTCAGCAGTTTGGGAGACCAAAGCAGGACG	5
GTAATCCTAGCACTTTTGTCGCCTGGGCGACACACCAAGGCTCTGTCTCA	2
CCCTTCCGAGCGGCGCCTAAGCCTTTGCCGTGAGCATGCACACTGAGAAT	10
TGCAGAAGTCCGTGGTCATAACCTTGTCTCTGGCTGCATCCAATTGTTGC	X
CAAACCTGTTTGTGAGATCTCCTTCAAATACTACTGTAGACCCCAGTGTT	8
AAGTCTCAGGTGGCTGCGTGTGGTGGCTCATGCCTGTAATCCCAACATTC	1
CTATCTATTCCATGTTGGACCAATACCACACTGCCCTAGTCACTGTTGCA	19
AGATAAGCCGTGGACCCGCCTCACCGCTGCAGACAAAGCTGCCATCCGAA	6
TCTAGCATCAGATCAATCCCAAGAATCCATCAGCAACCTCAGACCAACCC	8
TACTCTTGGAGGCTGAGGCAGGAAGAGCCCTTGAGCCCAGGAGCTGGAGG	5
TGGACTAAATGCTCTTCCTTCAGAGGATTATCCGGGGCATCTACTCAATG	14
AGTCATGATCACGCCACTGCGCTACAGCCTGGGCGACACAGTAAGACCCT	15
CTATGCCATATGTGCCTCTGGCTTATTGCCCAATTAATTGTAGTCTCAGG	19
CCATCTTCTTACACAGAGAGGCAGCTGAGGCAGGACAGTGGGGCTAACTG	9
GGCCAGGGTTCTCGCTCTTGTTGCGTGTGTTCAAACAACCGGCACGGTCT	9
GCTGGAGTTGGTGCTGGGAAACCCGGGGCTAATGTTGACAACAGGCTCGA	10
CGTAGGGTAGTCAAGCTTCCAAGACTGCAGACACATCCAAATGAGGCGCT	6
GCTATACTGTCCTGCTTGTACAACATGGTTTGGGGTGAAGGGGAGGAAAG	Y
ACATCTTTCTGGCACATAACTGTCTCCTTAACCACTGGAACAGTTCAGCC	12
CATGGAGCTCAAGATGTCTTGTGTCTGTGTGGCTAGATGGCCTCTGCTTG	2
GAAGCAGCTCCAGCCCATGTTTCTGAAGGGTTACCGCTGACAAGGAGTCA	14
CTGCACACATTCTTATTAGGATTCCACCTTGTTCTGGTGTTGTAGAGATG	19
AACGGTCCTTAGACCCCGCTGTGCCCTGTGCTGTAGCTTCTTTCCAGGCC	19
GCCCAGGTTGGAAATGGAGCAGGCCAAAACTCCCAGGCTGATCAGTAATG	1
GGAGAAAAGGACAGGGATAGGTACCACAAAGATAGGGACCACACTGACAG	6
GTTTTGGAGGTCATGGTGGGAGGATCACCTGAGGCCAGGAGTTTGAGGCC	
CTTCTGCCCCTCTTCAGACAGTCCTTCAGCTATTTCATGGCTCTCACCCT	1
ACACAGTAGCGATGGAGGTGACGTAGCTTCCTCCGAGTGGAACTGCAGCC	10
CTAAGGGGGTTGAGGTTATTGTAGACGTTAGATTGCGGGCACCGCCAGGG	20
GGGGTTAAGGTTCATGAGAACCATGGAAAGATGTGGTCTGAGATGGGTGC	2
CTACTCGGGAGACTGAGGCAGGAAAATCACTTGAACTCGGGAGGCAGAGA	12
GTGTCCAGTATGGCTTTCCAGGCTAATCACTTAGGGCAGAGACCCAGCCT	5
GCCAGGGCGGGGGCGGGGTGCATTTCCATCCTTGTAAACCCTTCATAGTA	14
TGTCCCACTTCCTCTATGTCCCTTTCCTTGGTGTCTGTGTCTGTGGGGCC	22
CTGTATTTAAGGGTAAGCCCCACAGCGGGCAGCACAAACAGCCTGGGAGC	1
TGTGGCGCTCCGTGAAATTAGACGTTATCAGAAGTCCACTGAACTTCTGG	
TCCAAGGCCTGGATGCTAATCAAGATGAACAGGTCGACTTTCAAGAATTC	1
TGCAAGGGACAGGGGGCCTGACTACCCAGTCTTTGACTTGTATCCTCTCC	1

CCGCCTCCGCGACACGGGGGAACTGTTTTCTTGATGATGCTGTGAACCTT	X	
GATTGTGCCACCGTACTCCAGCCTGGGCGACAAGAGTTAGACCCTATCTC		
CCACGCTTAAACCAACCCCCAAATTTAGCATATTCATTTTGCCATGAGCC		12
GGAGGTCTGGCTCAGGCTCAGTTTTCTCACATTGGTGCATCTCTTCATGC		15
CAGCCCAGAGCACTTACTGAGAGTAAAGAGGCTCTAGCCACCCCCTTACC		7
AAAAGAGTGCTAGGATTGGGCCAGTTAAGAGCAGGGGAGACCTGGCTGGG		6
CAAAGAAGCAGGGGAAAAGTAAGCTCCTCCAAAGTTGCTTGCAGTGCTGG		6
TGGTGTGCCATCGCCGTGCTAGTGGGGGTGCTTCTCCTCGTTCTCATCTT		19
GGGCAGAAGCTGGCTCTCTACTTACAACCTGCTTTCTCTGCTGAAGCCTT		17
GGAACTCCCAACTTCGTGCCCTAGATCCTGCACCTCCCCACTCGAAAGTG		19
CAGCCTGACCTATGGGGCGGGGGACTGGAGGCTGAGTTTAATCATCTGGC		17
CCTACAAGTGCTGACCAAGCTGGGCAGTTTTAGTTAGAATCCTCCTTTTG		2
CGATGAGAAGGTTTACTACACTGCAGGCTACAACAGTCCTGTCAAATTGC		6
ATGGATCATCTGAGCCTCAGGAGGTTGAGGCTGCAGTGAGCTGTGACTGC		15
TCCAGCAGTGGTCATTCGACAACGAAAGTCATACCGTAGAAAAGATGGCG		17
AGCAGTGCCTGTGACGCATTCCTGTGCTGACCTGGATTCTTCCAATATGC	Y	
GCCACAAATAGAAAAGTTTTCTGCAAATGTACCCCATTCCCCTGTGCGAG		
GGCAACTCCAGTCAGAACAGCAGAAATAAGCGTGCCGTTCAGGGTCCAGA		13
CTCTGAGACTGCTAAGTAGGGGCAGTGATGGTTGCCAGGACGAATTGAGA		11
GGGGAAGAAGCAAGGAGTCTAAGGCCCTAGCGATTTGGGCATCTGCCACA		1
GGTTTTCTCCTACATGGACCGACAGCCAAATCTGCCTTCACCTGGCACGC		6
GATGGCGGAAAGAAGGCACCAGGGCACAGTGGACACTCATCCCGTGACAG	X	
GTGGAATGGCCCATGCCTGTAGTCCCAGCTACTCGGGAGGCTCAGCAGCA		18
CCTTAGGTCTCTTGACAGGAATCTGCCCTCAGACAGCCAGGACTTGGGTC		10
GTCCAGGATTGCCTCACTTGAGACTTGCTAGGCCTCTGCTGTGTGCTGGG		3
CCTTGGCCCTCCAAGGCTGGGAAAAGACAATGACAAGTCAAATCCAGACC		11
GCCTGAGAGAGCTGAAGAGGCAAAGCTAAAGGCCAAATACCCAAGCCTAG		1
CAGCGTTAATCCTGTATGGCCAGGAAACTGAGTAGACTCCTGTGTAACCC		10
TTACAGCAGCCAGACGATCATGCAGCTACTACATCACTTTCTTGGAAACG		14
CTCCCCGTCTTGGAAACAAGTTTTAGTTTATTGCTTTGGAGACTAGAGCC		1
CCTCCCAACTCTGATCAAGATCTTAATGAATCCCTGCCACCTCCACCTGC		17
GTAACTCCCCCCAGGTACGATAGGGACTGAATATGGACCCTGCTGAAAGC		13
CTCCAGCCCACGTTCTCTCTGCCTGTGAGCCAGTCTAGTTCCTGATGACC		15
GAGCTGAAGAGGGGCTGCTGTTTTCCGAATGTTTTCCATTTCACAGGCGC		
GGTGATGTATGGCTAAGATTTCACTTTAAGCAGTCGTGAACTGTGCGAGC		2
CGGAAGTGGAAGTGGTCTTCCAAGGCTTTTTTGCCGCTGGTGTCAGGAGT		19
GTGGGTGGCAGGGAGAGAAACCCACCTAGGCCTCTCAGTGTGTCCAGGAT		
GCAGCCTCAAAGAATTACCCCTCCCAAAATGTCAGAAGTGCTGAGGCTGA		2
GGTGATTACCCTGTGTATAAGAGTATGTGTCTCACTGCACCTTCAATGGC		1
AACCGCCTACGAGACACTCAAGGTCTCTCAGGCAGCTGCAGAGCTTCAAC		9
ACACACCTGGAGAGGACATTTGAAAACACTGTTCTTACCCTCGAACCCTG		14
CGAGTAGCTCTAAAACAAACCACCTGACCAAGAGGGAAGTGAGCTTGTGC		1
TTCAACACTGACCTGAGGTTTCAGAGCGCAGTCGTCGGTGCGCTGCAGGA		12
ACTCCCAAGGAGACTTAAGAATGGCAGATGGAGTGGCCAATGTTGAGCAC		7
GACCTCCTGGGATCGCATCTGGAGAGTGCCTAGTATTCTGCCAGCTTCGG		3
CTGCCAACCCCACCAGTGATGAAAATTAACTGTGGGCCACTCGCTGCAGA		5
GGCCTTCTCACCTAACGCCACCAACATGGTGTTCAGGCGCTTCGTGGAGG		
GTAACTGGAAACTGTGTTGCTCTAACCCTCCTCCAGCCCTGCAGCCTCCC		4

AGAGGAAGAGGGTGGTAGAGGAGGTAAGACAGTAGGGAAAGACAAGGGCC		17
CTAATAACAGTGACCTCCCCGCCAGGTCCTGTGTGTTGCCGGCTGAAGAA		
TATAAGATATTAAAGGGTAAGTCTCTCCGGCCCGGTTTCCCTCGGTGTGC		5
TTGCAGGGGCCCGAGACCCCACCATTGCACTCCAGTCCGGGAAACAAGAA		6
GGCTGTAACCAAGTCCACCCATCCCTGGGGCTTCCTTTGCTCTGCCTTAT		12
TCCCTCTGCTACAGCCCTGGGAGGAGCCAGGATCCTTGTTGGTCTAGCTA		20
ATCTGGCCTTGCCCCCAGCAGTACAGTCCTTCCTAATAACGGGGATGCTC	Y	
GCCACAAAAGGCATTCTCTGTCCTACCTAGCTGTCACTTCTCTGTGCAGC		4
CCTAGCTGCTCGGGAGGCTGAGGCAGGAGAATCTCTTTCTTAATTGGCCA		9
GAGGACGAGCTGCCCTATGATGACTGTGTGTTTGGAGGCCAGCGTCTGAC		14
GCTGCCTCTCTCCAGAAATGAACTGTGATGGTGGACACAGCTATGTGAGG		7
GCAGCGTTACTGGCCTATGTATCATCCGTCTATGGCTTCCACCCAGTCTC		6
CTGGGACCCTCAAGAGGAATACCACTTATGTTACACTCCTGCACTAAAGG		2
AGTGGCGTGCCCCTTGGCCTCACAGGCAAAGAATAACTTAAAAGCTGACG	Y	
GTGATGTGTCACGCCACTGTACTCCAGCCTGATGGCAGAGCGAGACTCCA		2
TCGTCACTCACATGCACGACAGTCCTTGTTCCCCCAGGAAGGGCCTGGTG		
CACAGCTAGGAAGCCTTAAAAATGTCATGCTGGTATTGGGCTACAACCGG		12
ACTTGCCTGGGTCACATAGCTAAGGAAGAGGTGGACTTGCCCAGCTTTGC		6
CTGGCGTTCCACACCAGCCTGGCCAACATGACAAAACCCTGTCTCTACTG		
TGTGGTGAGCCGAGATTGCACCATTGCGCTCTAGCCTGGGCAACAACAGC		11
CTGTAGATGCTGTTGATGATGCCATTCCAAGCTTAAATCCTTTCCTCACA		11
TGTCCCGAGGCGCAGCAATAATAAGGCAGCTGTTGAATGTGAAGGGTCCC		8
GCCTCTGTATTGTGTACCAGAATATAAATGATACACCTCTGACCCCAGCG		18
GTCCATCAGCCGAGGAGTGGATAAGCCAAATGTGGTGGATCGAGACAATG		17
GCCGTCGGTCTTGTCCCGCAGGCACTGGCCAACATGGCGAAACCCCATCT		1
GGCCTTCACTGCTACGCCCCTGGCCCCAAAACAGAGAGCAAGACAGTTGT		20
CCTGCAGTGTAAGTACAGCACACTGTCAAATTCTTTTCCTTAAGGTGCAC		12
GTAAGGTTGTCTACTCAGGAGGCTAAGGTGGGAGGATCACTTGAATGCCA		
GGAGGGGAGCATGGGAAGTGACGGTTTAATGGGCACAGGGTTTATGTTTA		2
GGCAGTTCCTACACCTACGGTGTGTGTTCCAGCAGGGAGGAGTTATGGGC		12
CTCCATCCAGCTAAGCGATCTTGAACAAGTCACAACCTCCCAGGCTCCTC		5
CGGGACCTGTGTGGGACCTGTGTCCTGTGGTGGCCGTTTGCAGTTTCTCT		
GAGGGAGAAGAATAAAGCAGCTGCCTGGAGCCTATTCACTATGTTTATTG		2
GGGATTACAGGCGTGAGTGACCATGCCTAGCTCACTTCCAGGTTTAACAG		3
AAATCAAAACTACTATGAGGCCGGGCACAGTGGCTCACGCTTGTGGTCTC		
GCTTCCCTTTTCCCTCTGTGGCAGTTACGATTTTGACTTCAGTCCTGAGA		1
GCATCAACCCTGTGCTCTATGCAGCCATGAACCGCCAATTCCGCCAAGCA		12
GACTTTCCAGTTGGCCCTGATTTTCAACCATGTGATTGTTTCACTCCTGG		
CCCTAGAAATTAGAAAATCAAGCTTTGGGCCAGGTGCGGTGGCTCATGCC		3
TGATCCCCAACCTGCACGGGGCATTCCTGCTTCTCTCTCAGGCCACCACA		20
TGAGGCCCAAAGCACTTGCTTACATCCTCTGATAGCTGTTTCAAATGTGC		8
TTTTCTTTGAGACAGGGTCTTTCTCTGCACTCCAGCCTAGGCAACAGAGC		2
GATGACGCTTCATCTGTTTCACAAATTCAGTCTCAAACTCAGTCACCGCA		10
GGAAGAGGAGGAACTGAGGTCTCATCACATTCTAGAGCGCTTGGATGCCC		
GGCTGGCGGATCCAAGCAAATGGCCAATGAGATCATTGTGAAGGCAGGGG		4
GAAACCATCTTGCATGCTAGAAGTTATTACCTGGCCTATTCAGGCTTTTG		
GAAAACCCTGGGTCTCACATCGAGCTGCTGGAGGAGGTGGCCGAGAAGGT		7
TGAATATTAGGGTCATTTGGCACTTCTCAGCAAGTAGGATACTTCTCATG		9

TCCCCCTTCGGATGTGGCTTGAGCTGTAGGCGCGGAGGGCCGGAGACGCT		7
ACGGCGTTCCTGGCTCTCCTGCCCACAGGATGAACATTTTCGGCTTCCTT		16
TGCCGACTCCATTTTGTCGGTAGAGGCAGAAGGAGAAGGTCGGGTTGTAG		1
CTGCCTCCCTCATGGTGTGCGTGTCGTTCTCTTCCTGACGCATCTGTGAT		12
TCAGGGTCTTCCCATGGTGGTTCAGAATAGATGAGCATAGCAAGGTTTTG	X	
CCCTCCACGTTGGTTTGGCTATGATAGGGAAGGAAACCCTGTTACAGAGC		7
CTGGGCAGCAATAATAATTGGGCATGAGGCTGATTACTCAATGGTGAGGG		10
GAGATGTCACCAGGATAAGACCACAGGGAAGCAAAGAAGGAAGAGAGCTC		17
GGGCTTAACCTGTGACTTTAATAAGCTGGAACAGTCCACTGAATGGGTAT		15
GGACCATGAGGAATTCTCCAGTGCTGTCGGGGTTTCCGATACTGACACAG		1
TCAGCAAACAATGGCAAGGCAGAGCCACCAGAAACGTACACCTGATTTTC	X	
TCCAAGGCACAGTGAGCTGGGCAGAGCTGGGCTGCCAGAAGCCTTTTTCA		1
AGGGCTTATTCTTCCTCAAGTAAGGGGGAACTCCTGCTTTGGGCTGGGAC		1
ACAGGCTTTACCGGCCCCACCTGGCATCGGACACCATCTACCAGATCATG	X	
GCTTCTACTGGCGCGTGAGTTCCCGGAGTGAGTTGAACCAGGTGGACCAA		20
GACAACCCCCTCCACCGCCAGTAATAAAGTCAGCCCTGAAAAGCATGAAG		16
GGGGAGAGGAAAAGTGGATGGAAGTGTCTGGAAAGGGCACGAGAGAGTCT		1
GGCTGCATCATGGGGAGCCATTTTACAAGCAGCACCATGGGTGTTATGGG	Y	
GCAGGGACCCAGTGAAGTTTCTCCGTTAAAGATTGGGAGTCGTCGAAATG		2
TCTATTGGCCATCTGGGCTTCACAGCTTGAGTTAACCTTGCTTTTCCGGG		13
TGGGAAATGAGTGCTCAGGGAGATGGAGCTTAGGGGAGGTGGGTGCTTCC		6
CAGTGAGCCACAATCACACCAATCACTGCACTCCAGCCTGGGCAATAAAG		20
GGCAGCTGTAGATCTTGATCTTCCAGGTACCCCATGTACCTTTATTGAGC		6
CCACTTCAGAGGCAAGAAGGCCAAAAAGCATATGCTGGTTCCTCCTCCAG		16
GGACCCCTGGATCCTTGCCATTCCCCTCAGCTAATGACGGAGTGCTCCTT		1
GTTGTGAGGTAGGGAAGTGAGGAGGAAAGCCATGCCGAAGCAAATGTTAG		6
CCTCTGCTCTTGGAAGCCCGTGCAGACCCCACAATAAAGAATAAGCATGG		10
CCCGGTGCTTAGAGGTTAACTTGGTGGCCTAGGAGAGGGAGAAGCCAGGA		10
TAAGTTAGCCCTTTGGGTAAGAGTTCCAAGTGTTTCTTCTGGTTTGTCAG		
AATGATGCCCTGGAGATGTCACAGATTCCTGGCAGAGCCATGGTCCCAGG		4
TCCAACGCATCCCTGCTGATCCAGAATGTCACCCGGAAGGATGCAGGAAC		19
AAAGGCTTGCTTGAGGGAGCTTATAACTTGCACCTAGAGGGTTTACCCAC		21
CCATTCAGGAAGGGCTGGGGGAGTGTGTGTGGCAATAAAGCTTGAAGGCA		3
GGAAAAGGAGGCCTAGAAAGGTTAAGTAACTTGGTCGAGACCACTCAGCC		11
GACACCCACCAGCCAGAGTTACAGGTAAGGTCCCAAAAGTGGCCAGGCTG		1
CAGGCCCCGGCAGCACTGCTACTTGGGAGGAGCCACTTCACCTTTGTATT		1
TCTGATTGTAACGTTGCTGTGGGAACGAGAGGGGAAGAGTGTACTGGGGG		15
GGCGAAGTCGTCAGACCAAAAGGATGGCTCAGCAAGTGAAGTACCCAGTG		
AACTCAGCAGAAGCTGGTAAAAACATGGGGAGCCCGGAGGACAGGCTGCT		
CATTGCACTCCAGCCTGGGCAATAAGAGTGAAACTCTGTCTCCAGGAAAA		19
GGCCCCATTGCTTGGGCGGCTGCTGTATTTTCATTTACTCTGGCCCTTGG		
GGCCCTATACCTGATCACCTCTAAAAACATGAACAGTGTTACAAAGGCAG		
GGGGACAGTAAATGTATGGGGTCGCAGGGTGTTGAGTGACAGGAGGCTGC		19
TCACAGTTCTGGAGGCTGAGAAGATCGTGAGGCTGCATCTGGCAAGGGCC		10
GTCCCAGTTTCCCACTCTGAAGATCCCAACATGGTCCTAGCACTGCACAC		1
GGACAGGTTCACCTTCCCACAGAGTAAGGGGTGAGTGACCCAAGGCTCAA		1
TTTATTACTTAAAGGTACCGAAGGAAGGCCAGGTGCAGTGGCTCACGCCC		10
GCCAAAGTATATGTTCAGCAGTGTGCCCAGGATTGAAGGTGTAAATGGGA		

GTAATCTGAACATTTGGGAGGCTGAGGCAGGAAGATCGCTTGAGGTCCAG		12
AGGTACAGCTGGCATTTTGGCAGATGCATAGAGACATCTGAGACCCTCAG		5
ACACTCAGCACCCCTCCCAAAGAGCGCCCCCTGTGTGTTCTGGATCTCTA		
CCCAGGAAGTGTGCTGGGCAGCCTGTTCTTACTCCAGCTCAACCCATTGG		7
TTGAACCCAGGAGGCGGAGGTTGCGGTGACCCTCCAGCTTGGGCAACATG		17
GTGGGGACTCAGGGCTGGACCGACGTCCTAGTGGACCTGATGTGAAATTC		12
GGCCAAGATCGCCCCACTGCACTCCAGCCTGGCAATAGACCGAGCTCCGT		7
CTGACTCAGCCAGGCTCCCTGAACTTTTTTCCTTGTCCCATCCTGGGGTC		20
TCGGGGAAACTGTGTGTGCTGAAGAGTACGTGGGAGCTCTCTGTGCTATC		
CTTGAAGGCCTACTGTCATCCACAAAGCTCCTTTTTTACGGGCATCCCAT		2
CGCTGAAAGAGATTCCAGTGGGACATGGTGCCGTTTTTCTGTTTGCCTTC		9
TGATGTGTCACGCCACTGTACTCCAGCCTGACGGCAGAGCGAGACTCCAT		2
CTCTGAGAAGTGGGGTGTGGTCTCTCAGCTGTTCTGCCCTCATACCCTTA	X	
TTTTTTGAGACAGAGTCTCACTCCAGCCTGGGTGACAGAGCAAGACTCCG		21
CCATCCCTCATGTGTTCTCAGCTGGCACTGGCTCTGCCATCCTGCCTCAT		
TGACCTATGGTAGTGCTACTGCACTCCAGCCAGGGCAACAGAGTGAGACC		19
CAGCTGGTTGGTCAGAGTGAGTCAGAAGGCCCCGTGATACAAGAATCAGC		7
GCATGAGTGAACCCAGGGGAGACATGCAGAGACTTGCCCAGCTGTCTTAC		
GAATAGGGCAAGACTAAAGGACAGAGTAAGGGTGCTGGCCGCCACCTGAC		
cctgaggggagAGCTGGTGGAGGCTGTGAAATGAGATGGGGAAGTAGGGA		
GCACCAACTTCCCTGATGGGCATGGCTCCCTTCACTGTCGGGTTGGATTC		
CGCAGTGGGATAAGTTGCAGGGTCTCTTAACTGCCAAGGTACCAAGTAGG		
GGGCTGCTGAAAGGGGAAGTTCTTGATGCCTTCATCCTAGCTCAGAGGGG		
GCAGTGCTAGGTTAACAACAAGGTGCTTACTTGCTTGGGGTGCCAGCCAA		
GCCAAGGAGGCAAACCTAGGGTGATGTCATTGTTCGAAGCTGTCACCGCC		
ACAGAGCCCTAGTTGGGAGGAGCCCACGTTCAGCAGGGCACATGGCAAAA		
CTCCTGACCCAGGCTGCCGACCCCTAACCCTGTTTTCTCTCCACAGGACA		
ACCCAGAAGGTATCACCAGCGAGCCTCATCCAATAGCCTCAGAACGCCCG		
TCCCACAGTTGGTAGTGCCAGGCAGGAAGCAGGAGTGCAGGTTGCAGGAA		
CACCCGATTGATAGGAGAGTGTCATGCAGTCAGCCGAACTCCGACTTCTG		
GGGAGTGCAGTCCGATGCTGCAGAAGACAGACTGTTCCCTCTCTCTTCCC		
AGCGCCCGCCTGGATTTATAGGGGTGAGCTTCCCTCATGGAGAGCCTTGT		
GATGTGTGGCACCTCAGTGTTGTCATAGCTTCTGCCTCCTCCGTGTGGAG		
AGGCCCTGCTTCTATTATGAGAGCCTGGGCCCATAGTGAAAAAATGTACC		
GGTTCACACATCTTGTCTGTGGTGGAGCTAGGACTTGAACTCAGGCAGCC		
TGTAGTTTTCCCTCCCCATGGAGAATCAAACATTTATACAGCATTCTACC		
CTCTGGACAGGCTGACTGGTAAAGGGCTTCCTATGTTAAAGCTCCTCTGT		
GCCAGCTCGTCCTTTCAAACTATGTGAGGGCCTACCTTGAGTCACCCCCA		
GATGAGCTGCCTCTGCCCACAAGTGCAATAATATCAGAGCCCAAGTGACC		
CCACAGAACCCCTTTCTCTAGCAAAACCTAACATTCTATGCCGATCCAAC		
GGAACCTGAGTGAAACCATCTCGATCCTCCAGCATAGCACAATCAGCAGC		
TTTTTAAGCGATTTAGATGGAAGTGGCCACAGTTGCGAGTGGCTCCCCCC		
GAGCTGCTTAGGGACAGGTGAGTCCTTGGGGGCATGGAACAAAGCTGGTC		
TCCAGGCCAGTAGGTGGTGCTTGCAGATAAAAGCCAGCTGAGCAATGTGC		
TTTTCAAAGAGGCCCCATTAACCGGCCCCCCAGAGGGTTCCCTTTAGGAG		
GTCTTTGGAGCCCAGCCTTCCCTGTGTAGTGAGCTGGAAACTCCTTTAGA		
GCTGCACCGATCCAGTGAGTGTCTGGGTAAATGCCTTCACTGCTCTGGGC		
CTGTCAAACGTGGCACTTGGGCTGCACAGCCTTAGTTGGATACTCTCTGG		

GTGGCTTAGGAGAGTTGTTCTCCACACAGCTGGGCACCAGAAGCAGCCAG
GCACCATGCATGGAGTCAGCCATTTCTCTAGGAACCTTGATTCCTGTCTG
GCACCAATACATGGGGGAAAAATGGGAAAGAAGCGTTCCGGGTAGCCATG
GACCTCCTTTAAATCCATAAATTCAGCCGGGTGAGGTGGCTCATGCCCGG
TCCTGCAGAACCTTAGACTCGTGTATTAAGTACGATTACCCAGCACTTGC
GGCAAAGCCAAGAACCCTCGTGGGCTAAAACCCCGCTTTGGGGCTTAAAG
GCAGACATAGAAAGGGGCAAAGTCTTGGTGGTGCGTGCCTGTAATCCCAG
CCCACACGGTGCCATGGTTTAATTATTCCCCAGCTCTACTTTTGGGAGCC
GAAGCTTCTCTGTGGGTGGATGCTGCCAAGATGATGGGGCTGCAGCTGTC
GGCAGGAAAATTGTATGTCAGGGTGTATTTCTGTGTGGGGTTGCAAGGGC
CACTGGCTGGGTATGGTGGCTCACGCCCCATTTACTTTTTAGCAACAATG
GCCTCTAACCCATCAGGAATGCCATAAGTATAGACCCTGTCTTGGGAGAG
CCTCCCAGAAGAAGTCAGTGGGAAGAGATGGCCAGGGGAGGAAGTGGGTT
TCCCTGGGGTTCCCCTTTTTACTTTTCCAGCCACAGAGATGAGACATTAG
GCACTCTAGACCTAAGTTGTCCAATATGGTAGCCACTTGCCACATGTGAC
GACCCGCGGCAGACAGTCCTCGAATCCTGGAGTTTTCTCTGCTCAACCCT
CGTGGCAGGTGTACATCTTGCTCCCTTAAATGAATGCTCCTTGACCACTC
GATATCACACCCACACAGTAGCGTCTTTGTATTTGGGTGATGGTAAGATG
GTGCTTGGAAAGGGCCAGGGGAAGGCTATTGTCTGCTCGCACAAGTTACT
CTGGCATGGGACTGGCTAGGTTCAAATCCAGAGAGCATGGGTTTATACAC
GAGTCCGGCTCCAGCCAGTAAAGGGTCAACTCAGCTCTGAATGTTGGGAG
CTCAGGGAAGTTAAGCATGTTCGGCCCTCCAGATTTGCGGATTCATCATC
GACTACCCATTCCGTCATCTTATCAACACTCAGATGTGAAAGGCTAAGCA
GGATGATGAGAGGGAACATCTTTGCCCGCTTCCTGATCTTAGTGGGAAAG
ACACAACCTACACAGCCTCTCTGTTTCCAAATACATGCACTGAACCTCCC
GCAGTCACTAGGGCCATAAGTCAAATGCTCAGAGTCTTGAGACAGTCGCC
GCTGACAAGGAAGTAAGCCAAGCAGAGGGAGGATAGAACATTGCAGGTGG
CTGCTTCCCTCTTCCGCATTTAAGGACGCTGTGATTGCATTAGACCCACC
CACAGCCCGTTCTTAACTCTCACAGTACACCCAAAGCCCCTTTACCCTGC
CGAGAAAAGGGTGCCCCAAGCCACGAGGGTAACATGAGCTATGGCAGTCG
ACCCACTGTTTCCCTGTGTTTCTGCCCAGTAGAAGGTTCCTGGATGTGCC
CCAGGTGCCGCCCACTCTTGACGTGATACTTACCGTCAATGCTCCTTACC
CAAAATTCTCCGTAGCAACTCACCCACTTTGCAGTTTATGTGATCCACAC
GGCTGGTTATCAGGAGTGGCCTGTCCCAAAGCAAATGCTGCATGAGGGTG
GCGGGGAAACTCCCAGTAGGCGCTGCTGTCCAGGGAAAATGATGGGCTTT
CATGCCTAGTGCCTGCCCCAGTGCAGATGCTCAGGAAATATCGTCCTTAC
CAAGTTCTTAACCATCCCGGGTTCCAGTGGTTACAGAGTTCTGCCCTGGG
CTGAGTAGGCCGGACTCGTGTGGCTCACGCCTGTAATCCCAGATGCTGTT
GGGACCATTCCTGTTTGGTTCTCCATTGTACCCTCAGAACTTATGCAGTC
CTGGAATTCAGTACGTGAGGACTTGGACTTGGTGGGCACAGAATGCATCT
CCGGTCCTTTCCAAGCGATGTGTGGACTCGACTTTGACCAAACCCCTCCC
AGCCTGTGAGTGAAGATCTGGTTCAGGAGTACACTGGGAAATTGTCTGGC
CATCGGTCCTTACAGCCGCTCGTCAGACTCCAGCAGCCAAGATGGTGAAG

Probe_Chr_Orientation	Probe_Coordinates
+	74340137-74340186
-	38299200-38299203:38301057-38301102
-	229729706-229729755
-	51285425-51285474
-	130443163-130443212
+	3257423-3257472
+	93094373-93094422
+	8293227-8293276
+	91411148-91411197
-	44615731-44615752:44615753-44615780
-	185184354-185184403
+	113471394-113471443
-	70442883-70442884:70443063-70443110
+	36153798-36153847
-	20078363-20078412
-	3720269-3720318
-	4316893-4316942
-	47980580-47980629
-	75454418-75454425:75454426-75454467
+	75346964-75347013
+	137348860-137348909
+	53063842-53063891
+	44597523-44597572
-	101900852-101900901
+	58332875-58332924
-	24652407-24652456
-	90407224-90407273

+	245748661-245748710
-	3756-3805
-	148080892-148080941
+	6486367-6486368:6486995-6487042
+	60710651-60710700
+	152229888-152229896:152229897-152229902:152230215-152230249
-	68336908-68336957
-	28306729-28306778
+	1388309-1388358
+	19558234-19558283
-	34634020-34634069
+	171285570-171285619
-	12664737-12664786
+	22371126-22371175
+	82593742-82593791
+	180037176-180037225
+	107346975-107347024
-	23897916-23897965
-	148051810-148051859
+	22664611-22664660
-	247071517-247071566
+	61085713-61085762
+	34459695-34459744
-	52083405-52083454
+	100520197-100520246
+	12331650-12331699
-	23592413-23592462
+	11337048-11337097
-	45720017-45720066
+	111903182-111903231
-	114859890-114859939
+	134107742-134107791
-	51243958-51244007
-	66796993-66797042
+	157084272-157084321
+	20210812-20210861
-	174921573-174921622
-	46295471-46295520
-	230759010-230759059
+	126200169-126200218
+	76166279-76166328

-	76866031-76866080
-	88514916-88514965
-	97394601-97394650
-	1491641-1491690
-	137560553-137560602
-	146068544-146068593
-	53295365-53295414
+	241905635-241905684
+	8565844-8565893
-	184908064-184908113
+	23252401-23252450
+	31198110-31198159
+	47416014-47416063
-	133574936-133574985
-	133106828-133106877
-	179950251-179950300
-	41873670-41873719
-	112053558-112053563:112058513-112058536:112058537-112058556
-	145585935-145585984
-	48763124-48763173
-	104837929-104837978
-	103936333-103936382
+	205317527-205317576
-	153767258-153767307
-	114881070-114881119
-	185913945-185913994
-	48220757-48220806
-	93486339-93486388
+	37676117-37676166
-	101598419-101598468
-	103534340-103534389
+	219124373-219124422
-	45721550-45721599
+	113784584-113784633
-	27865202-27865251
+	48241909-48241918:48241919-48241958
+	26360209-26360238:26360239-26360258
+	3012033-3012082

+	38159368-38159417
-	20993986-20994035
-	191548621-191548670
-	48842368-48842417
-	3067402-3067451
+	27273891-27273940
-	19682836-19682885
+	31031334-31031383
+	65170462-65170511
+	232955648-232955697
+	67924838-67924858:67925932-67925960
-	8227510-8227553:8233793-8233798
-	49122497-49122517:49122753-49122781
+	148089810-148089859
-	104816333-104816382
-	177962642-177962691
-	50441973-50442022
+	10514353-10514402
+	81530777-81530826
-	223143575-223143624
+	48319749-48319749:48319750-48319751:48319840-48319886
+	91149642-91149691
+	13481379-13481428
-	10174011-10174060
+	174535022-174535053:174535054-174535071
-	13918925-13918974
-	151166817-151166866
+	70023312-70023361
+	43834109-43834158
-	24150565-24150568:24152574-24152603:24152604-24152619
-	118566707-118566756
+	12907768-12907817
-	3934-3983
-	37187233-37187282
-	32161087-32161136
-	37770607-37770656
+	29289975-29290024

-	142556030-142556079
+	191047547-191047596
-	
-	67689479-67689528
-	44510871-44510920
-	56780689-56780735:56780736-56780738
+	65144146-65144195
+	132565798-132565847
-	143072183-143072232
+	2962146-2962195
-	69712823-69712872
-	151773806-151773855
-	148397711-148397760
-	178071321-178071370
+	72067992-72068041
+	9998450-9998477:9998478-9998499
-	48399408-48399457
-	158517590-158517639
-	94892061-94892110
+	63928321-63928370
-	76970258-76970307
-	229535630-229535679
-	34816549-34816598
+	70564812-70564861
+	23855914-23855963
+	74973428-74973477
-	54546773-54546822
+	97673262-97673311
+	1716348-1716357:1716473-1716512
+	75103777-75103826
+	42795187-42795191:42795819-42795832:42795833-42795846:42797097-42797[illegible]
+	32030109-32030158
+	93213090-93213139
+	39061214-39061263
+	99472880-99472929
-	159860148-159860197
+	28692285-28692292:28692293-28692334
+	15970908-15970957
-	157024937-157024986
-	179326416-179326465

+	1818688-1818714:1818837-1818859
-	151629237-151629286
+	71528496-71528523:71528524-71528545
+	14806601-14806650
+	27841663-27841712
-	38474336-38474385
+	75909314-75909363
-	8226343-8226353:8226354-8226358:8227200-8227233
+	116133696-116133745
-	46820864-46820913
+	177409019-177409068
+	44290026-44290075
-	3811452-3811501
-	77415032-77415081
-	3918-3967
-	38041724-38041773
+	15634207-15634256
+	13391737-13391773:13394357-13394369
-	52892908-52892957
-	17183199-17183248
-	49120092-49120134:49120135-49120141
+	40646836-40646885
-	193631-193680
-	130058218-130058267
+	89330079-89330128
-	12124760-12124809
+	52968633-52968682
-	13869775-13869824
-	78692404-78692453
+	135643839-135643888
-	89852902-89852908:89854102-89854144
+	60091988-60092037
-	59290134-59290183
+	10289291-10289340
+	64481496-64481545
+	202785830-202785879
-	32597972-32597996:32597997-32598021
+	55386776-55386825
-	157979818-157979867
+	8382616-8382665
+	176425940-176425989
+	75006821-75006870
+	22556843-22556892
+	48486565-48486614
-	151612983-151613032
+	165941667-165941716

+	1388406-1388455
-	31715625-31715674
+	81587223-81587233:81596237-81596275
-	42915789-42915838
-	42178233-42178282
+	158439222-158439271
-	13116239-13116288
-	30360904-30360953
-	45889570-45889619
-	52320501-52320550
-	197568143-197568192
+	138775797-138775846
+	38114867-38114916
-	34260200-34260249
+	20225225-20225274
+	107720716-107720765
+	69730861-69730910
+	190421210-190421259
-	32906458-32906507
+	151993740-151993789
-	22002600-22002649
+	70533801-70533850
-	27732933-27732982
-	14589154-14589203
-	148866616-148866665
-	121578995-121579044
+	61282171-61282220
-	89219327-89219376
-	26655419-26655468
-	109204764-109204813
-	88129409-88129458
-	239635447-239635496
-	42355401-42355450
-	127780308-127780357
+	111971650-111971699
+	113433823-113433844:113468916-113468943
+	72657673-72657722
-	181862120-181862169
-	31836086-31836135
-	33021836-33021885
-	12856899-12856948
-	55431745-55431766:55431767-55431794
+	15342563-15342566:15342567-15342612

-	43502841-43502890
+	60706623-60706672
+	42861220-42861269
-	8577850-8577899
+	54527196-54527245
+	7309013-7309062
+	154846536-154846585
-	37875474-37875523
-	34940976-34941017:34941351-34941358
+	110552004-110552053
-	36019743-36019792
-	196772392-196772441
+	14326989-14327038
-	110714861-110714910
+	39045111-39045119:39047075-39047115
-	32901537-32901586
-	111519243-111519292
-	85379025-85379074
+	61941594-61941643
-	23784959-23785008
-	60232730-60232779
-	16829066-16829115
-	57945667-57945716
-	75269880-75269929
+	218709902-218709951
-	46828278-46828327
+	180365765-180365814
-	219823282-219823317:219823318-219823331
+	123775641-123775690
+	52026216-52026265
-	53042762-53042811
-	69239594-69239643
+	2269595-2269644
+	120505381-120505430
-	203398622-203398648:203398422-203398444
+	111882076-111882125
+	74955506-74955555
-	5426395-5426444
-	15454347-15454396

+	26207885-26207934
+	83500277-83500326
+	29648-29697
-	105156317-105156366
+	100100006-100100055
-	102241107-102241156
+	93779668-93779717
+	37978594-37978643
-	71639809-71639858
-	148067255-148067304
+	77044806-77044855
+	165651657-165651671:165651672-165651706
-	155749982-155750031
+	15477974-15478023
+	44078320-44078369
+	54174433-54174482
-	181791603-181791652
+	13313238-13313287
+	101876927-101876976
+	30236347-30236396
+	32935355-32935404
+	5923495-5923544
-	35908947-35908996
+	65179379-65179428
+	219054033-219054082
+	18576492-18576541
-	90572750-90572755:90573017-90573060
+	11411493-11411542
+	6749666-6749670:6749671-6749715
-	48463849-48463898
-	39484301-39484350
+	128882396-128882445
-	92520593-92520642
-	198642746-198642768:198642769-198642795
+	39243212-39243261
-	66859320-66859369
+	20171476-20171525
-	46552207-46552256
-	81930715-81930764
+	17562982-17563031
-	35677307-35677356
+	115981532-115981581

-	100073632-100073681
-	16526589-16526638
+	36307365-36307414
-	34185801-34185846:34185847-34185850
-	119017395-119017444
+	17351414-17351463
+	43947237-43947286
-	230744660-230744709
-	99926695-99926744
-	112851351-112851400
+	154893513-154893562
-	33779905-33779937:33782011-33782027
+	49263889-49263938
-	74241769-74241773:74242870-74242911:74244194-74244196

Definition

PREDICTED: Homo sapiens region containing similar to Williams Beuren syndrome chromosome region 19; hyp
PREDICTED: Homo sapiens hypothetical protein LOC642035 (LOC642035), mRNA.
PREDICTED: Homo sapiens hypothetical protein LOC641825 (LOC641825), mRNA.
PREDICTED: Homo sapiens hypothetical protein LOC283547 (LOC283547), mRNA.
PREDICTED: Homo sapiens similar to hypothetical LOC389634 (LOC654053), mRNA.
PREDICTED: Homo sapiens hypothetical LOC440731, transcript variant 2 (LOC440731), mRNA.
PREDICTED: Homo sapiens hypothetical protein LOC647834 (LOC647834), mRNA.
PREDICTED: Homo sapiens hypothetical protein FLJ37307 (FLJ37307), misc RNA.
PREDICTED: Homo sapiens hypothetical protein LOC649753, transcript variant 1 (LOC649754), mRNA.
PREDICTED: Homo sapiens hypothetical protein FLJ43663, transcript variant 2 (FLJ43663), mRNA.
PREDICTED: Homo sapiens hypothetical protein FLJ12078 (FLJ12078), misc RNA.
PREDICTED: Homo sapiens hypothetical protein FLJ39639 (FLJ39639), mRNA.
Homo sapiens small Cajal body-specific RNA 9 (SCARNA9) on chromosome 11.
UI-H-BI3-akp-h-05-0-UI.s1 NCI_CGAP_Sub5 Homo sapiens cDNA clone IMAGE:2735097 3, mRNA sequence
Homo sapiens cDNA: FLJ23098 fis, clone LNG07440
PREDICTED: Homo sapiens hypothetical LOC389089 (LOC389089), mRNA
Homo sapiens clone KU010717 unknown mRNA
Homo sapiens cDNA: FLJ22140 fis, clone HEP20977
UI-CF-FN0-afp-n-21-0-UI.s1 UI-CF-FN0 Homo sapiens cDNA clone UI-CF-FN0-afp-n-21-0-UI 3, mRNA sequence
603071853F1 NIH_MGC_119 Homo sapiens cDNA clone IMAGE:5163756 5, mRNA sequence
PREDICTED: Homo sapiens hypothetical gene supported by AY007155 (LOC439949), mRNA
BX103256 Soares_parathyroid_tumor_NbHPA Homo sapiens cDNA clone IMAGp998J244235, mRNA sequence
Homo sapiens cDNA FLJ41270 fis, clone BRAMY2036387
Homo sapiens thioredoxin (TXN), mRNA.
Homo sapiens ribosomal protein S28 (RPS28), mRNA.
Homo sapiens growth arrest and DNA-damage-inducible, gamma (GADD45G), mRNA.
Homo sapiens ribosomal protein S16 (RPS16), mRNA.
Homo sapiens ATP-binding cassette, sub-family C (CFTR/MRP), member 5 (ABCC5), transcript variant 2, mRNA
Homo sapiens guanine nucleotide binding protein (G protein), gamma 10 (GNG10), mRNA.
Homo sapiens LMBR1 domain containing 1 (LMBRD1), mRNA.
PREDICTED: Homo sapiens similar to 40S ribosomal protein S29 (LOC647361), mRNA.
Homo sapiens chromosome 9 open reading frame 19 (C9orf19), mRNA.
Homo sapiens ribosomal protein S6 kinase, 90kDa, polypeptide 3 (RPS6KA3), mRNA.
Homo sapiens retina and anterior neural fold homeobox like 1 (RAXL1), mRNA.
PREDICTED: Homo sapiens similar to Ssu72 RNA polymerase II CTD phosphatase homolog (LOC390033), mRNA
Homo sapiens pre-B-cell colony enhancing factor 1 (PBEF1), mRNA.
PREDICTED: Homo sapiens hypothetical protein LOC644250 (LOC644250), mRNA.
Homo sapiens transmembrane protein induced by tumor necrosis factor alpha (TMPIT), mRNA.
Homo sapiens plasminogen activator, urokinase (PLAU), mRNA.
Homo sapiens protein phosphatase 2 (formerly 2A), regulatory subunit B'', alpha (PPP2R3A), transcript variant
Homo sapiens zyg-11 homolog B (C. elegans) (ZYG11B), mRNA.
Homo sapiens CD82 molecule (CD82), transcript variant 1, mRNA.
Homo sapiens polymerase (RNA) II (DNA directed) polypeptide J, 13.3kDa (POLR2J), mRNA.
Homo sapiens hypothetical protein LOC284757 (LOC284757), mRNA.
Homo sapiens KIAA0319 (KIAA0319), mRNA.
Homo sapiens ribosomal protein S6 kinase, 90kDa, polypeptide 5 (RPS6KA5), transcript variant 1, mRNA.
Homo sapiens multiple EGF-like-domains 9 (MEGF9), mRNA.

PREDICTED: Homo sapiens hypothetical LOC644852, transcript variant 2 (LOC644852), mRNA.
PREDICTED: Homo sapiens similar to CXYorf1-related protein (LOC653635), mRNA.
Homo sapiens histone cluster 2, H2aa3 (HIST2H2AA3), mRNA.
Homo sapiens thioredoxin-like 5 (TXNL5), mRNA.
PREDICTED: Homo sapiens similar to hypothetical protein MGC40405, transcript variant 1 (LOC653158), mRN[illegible]
PREDICTED: Homo sapiens p21 (CDKN1A)-activated kinase 2 (PAK2), mRNA.
Homo sapiens ribosomal protein S27 (metallopanstimulin 1) (RPS27), mRNA.
Homo sapiens G protein-coupled receptor 177 (GPR177), transcript variant 2, mRNA.
PREDICTED: Homo sapiens similar to eukaryotic translation initiation factor 3, subunit 8, transcript variant 12 [illegible]
Homo sapiens colony stimulating factor 2 receptor, alpha, low-affinity (granulocyte-macrophage) (CSF2RA), tr[illegible]
PREDICTED: Homo sapiens hypothetical LOC646424 (LOC646424), mRNA.
Homo sapiens zinc finger, MYM-type 2 (ZMYM2), mRNA.
Homo sapiens protein phosphatase 2 (formerly 2A), regulatory subunit B'', gamma (PPP2R3C), mRNA.
Homo sapiens G protein-coupled receptor 160 (GPR160), mRNA.
Homo sapiens hypothetical protein FLJ11151 (FLJ11151), mRNA.
Homo sapiens Ral-GDS related protein Rgr (Rgr), mRNA.
Homo sapiens EF-hand calcium binding protein 2 (EFCBP2), mRNA.
Homo sapiens calcium channel, voltage-dependent, R type, alpha 1E subunit (CACNA1E), mRNA.
Homo sapiens dihydrolipoamide dehydrogenase (DLD), mRNA.
Homo sapiens unc-119 homolog (C. elegans) (UNC119), transcript variant 1, mRNA.
PREDICTED: Homo sapiens similar to H3 histone, family 2 isoform 2 (LOC653604), mRNA.
Homo sapiens transmembrane BAX inhibitor motif containing 4 (TMBIM4), mRNA.
Homo sapiens WD repeat and SOCS box-containing 1 (WSB1), transcript variant 1, mRNA.
Homo sapiens SH3-binding domain protein 5-like (SH3BP5L), mRNA.
Homo sapiens ubiquitin specific peptidase 15 (USP15), mRNA.
PREDICTED: Homo sapiens similar to SMT3 suppressor of mif two 3 homolog 2 (LOC390466), mRNA.
Homo sapiens AKT interacting protein (AKTIP), transcript variant 2, mRNA.
Homo sapiens tripartite motif-containing 56 (TRIM56), mRNA.
Homo sapiens cell division cycle 123 homolog (S. cerevisiae) (CDC123), mRNA.
Homo sapiens NK3 transcription factor related, locus 1 (Drosophila) (NKX3-1), mRNA.
Homo sapiens lipid phosphate phosphatase-related protein type 2 (LPPR2), mRNA.
Homo sapiens sulfatase 2 (SULF2), transcript variant 2, mRNA.
Homo sapiens interferon-related developmental regulator 1 (IFRD1), transcript variant 1, mRNA.
PREDICTED: Homo sapiens similar to SMT3 suppressor of mif two 3 homolog 2 (LOC728825), mRNA.
Homo sapiens netrin G2 (NTNG2), mRNA.
PREDICTED: Homo sapiens similar to F15D3.1a (LOC391475), mRNA.
Homo sapiens dehydrogenase/reductase (SDR family) member 12 (DHRS12), transcript variant 1, mRNA.
Homo sapiens phosphodiesterase 7A (PDE7A), transcript variant 2, mRNA.
Homo sapiens myeloid cell nuclear differentiation antigen (MNDA), mRNA.
Homo sapiens AT rich interactive domain 3A (BRIGHT-like) (ARID3A), mRNA.
Homo sapiens chromosome Y open reading frame 15A (CYorf15A), mRNA.
Homo sapiens CBF1 interacting corepressor (CIR), mRNA.
Homo sapiens chloride channel CLIC-like 1 (CLCC1), transcript variant 1, mRNA.
Homo sapiens transducer of ERBB2, 1 (TOB1), mRNA.
Homo sapiens SP110 nuclear body protein (SP110), transcript variant b, mRNA.
Homo sapiens lamin B1 (LMNB1), mRNA.
Homo sapiens coagulation factor II (thrombin) receptor-like 1 (F2RL1), mRNA.
PREDICTED: Homo sapiens similar to Fc fragment of IgG, low affinity IIIa, receptor for (CD16) (LOC652578), m[illegible]

Homo sapiens chromosome 9 open reading frame 95 (C9orf95), mRNA.
Homo sapiens hydroxysteroid (17-beta) dehydrogenase 11 (HSD17B11), mRNA.
Homo sapiens hypothetical gene supported by BC044942 (LOC441268), mRNA.
Homo sapiens signal-regulatory protein beta 1 (SIRPB1), mRNA.
Homo sapiens interferon gamma receptor 1 (IFNGR1), mRNA.
PREDICTED: Homo sapiens neuroblastoma breakpoint family, member 9, transcript variant 26 (NBPF9), mRNA
Homo sapiens CUE domain containing 1 (CUEDC1), mRNA.
Homo sapiens septin 2 (SEPT2), transcript variant 1, mRNA.
Homo sapiens C-type lectin domain family 4, member D (CLEC4D), mRNA.
PREDICTED: Homo sapiens similar to 60S ribosomal protein L21 (LOC731706), mRNA.
Homo sapiens prostaglandin-endoperoxide synthase 2 (prostaglandin G/H synthase and cyclooxygenase) (PTG
Homo sapiens RAB24, member RAS oncogene family (RAB24), transcript variant 2, mRNA.
Homo sapiens membrane metallo-endopeptidase (MME), transcript variant 2a, mRNA.
Homo sapiens ureidopropionase, beta (UPB1), mRNA.
Homo sapiens TAF15 RNA polymerase II, TATA box binding protein (TBP)-associated factor, 68kDa (TAF15), tra
Homo sapiens brain cytoplasmic RNA 1, Bc1 analog (mouse) (BCYRN1) on chromosome 2.
Homo sapiens Rap guanine nucleotide exchange factor (GEF) 1 (RAPGEF1), transcript variant 2, mRNA.
Homo sapiens vanin 2 (VNN2), transcript variant 2, mRNA.
Homo sapiens secretoglobin, family 3A, member 1 (SCGB3A1), mRNA.
PREDICTED: Homo sapiens similar to ribosomal protein S12 (LOC651894), mRNA.
Homo sapiens ubiquitin specific peptidase 49 (USP49), mRNA.
Homo sapiens thioredoxin (TXN), mRNA.
Homo sapiens acid phosphatase 6, lysophosphatidic (ACP6), mRNA.
PREDICTED: Homo sapiens hypothetical protein LOC650919 (LOC650919), mRNA.
Homo sapiens protein kinase, cAMP-dependent, regulatory, type II, alpha (PRKAR2A), mRNA.
Homo sapiens 5'-nucleotidase, cytosolic II (NT5C2), mRNA.
Homo sapiens ubiquitin-conjugating enzyme E2D 3 (UBC4/5 homolog, yeast) (UBE2D3), transcript variant 5, m
Homo sapiens pre-B-cell leukemia homeobox 2 (PBX2), mRNA.
Homo sapiens 6-phosphofructo-2-kinase/fructose-2,6-biphosphatase 2 (PFKFB2), transcript variant 1, mRNA.
Homo sapiens transmembrane protein 154 (TMEM154), mRNA.
Homo sapiens thyroid hormone receptor associated protein 2 (THRAP2), mRNA.
Homo sapiens N-acylsphingosine amidohydrolase (acid ceramidase) 1 (ASAH1), transcript variant 1, mRNA.
Homo sapiens acyl-CoA synthetase long-chain family member 1 (ACSL1), mRNA.
Homo sapiens pregnancy specific beta-1-glycoprotein 11 (PSG11), transcript variant 1, mRNA.
Homo sapiens transmembrane and coiled-coil domain family 3 (TMCC3), mRNA.
Homo sapiens chromosome 15 open reading frame 51 (C15orf51) on chromosome 15.
Homo sapiens thrombospondin 1 (THBS1), mRNA.
Homo sapiens solute carrier organic anion transporter family, member 4C1 (SLCO4C1), mRNA.
Homo sapiens meningioma expressed antigen 5 (hyaluronidase) (MGEA5), mRNA.
Homo sapiens H2.0-like homeobox 1 (Drosophila) (HLX1), mRNA.
Homo sapiens sulfatase 2 (SULF2), transcript variant 1, mRNA.
Homo sapiens RNA binding motif protein 7 (RBM7), mRNA.
Homo sapiens interferon, alpha-inducible protein 6 (IFI6), transcript variant 1, mRNA.
PREDICTED: Homo sapiens similar to ribosomal protein S12 (LOC651894), mRNA.
PREDICTED: Homo sapiens protein tyrosine phosphatase type IVA, member 2, transcript variant 9 (PTP4A2), m
Homo sapiens cathelicidin antimicrobial peptide (CAMP), mRNA.
Homo sapiens histone cluster 1, H2bh (HIST1H2BH), mRNA.
Homo sapiens tumor necrosis factor receptor superfamily, member 12A (TNFRSF12A), mRNA.

Homo sapiens chromosome 19 open reading frame 40 (C19orf40), mRNA.
Homo sapiens protein tyrosine phosphatase-like A domain containing 2 (PTPLAD2), mRNA.
Homo sapiens signal transducer and activator of transcription 1, 91kDa (STAT1), transcript variant beta, mRNA
PREDICTED: Homo sapiens proteasome (prosome, macropain) 26S subunit, non-ATPase, 12 (PSMD12), mRNA.
Homo sapiens plasminogen activator, urokinase receptor (PLAUR), transcript variant 2, mRNA.
PREDICTED: Homo sapiens hypothetical protein LOC646064 (LOC646064), mRNA.
Homo sapiens interleukin 4 receptor (IL4R), transcript variant 2, mRNA.
Homo sapiens zinc finger protein 14 (ZNF14), mRNA.
Homo sapiens branched chain ketoacid dehydrogenase kinase (BCKDK), mRNA.
Homo sapiens CKLF-like MARVEL transmembrane domain containing 1 (CMTM1), transcript variant 23, mRNA.
Homo sapiens alkaline phosphatase, placental (Regan isozyme) (ALPP), mRNA.
Homo sapiens growth arrest and DNA-damage-inducible, alpha (GADD45A), mRNA.
Homo sapiens islet cell autoantigen 1, 69kDa (ICA1), transcript variant 3, mRNA.
Homo sapiens clathrin, heavy chain-like 1 (CLTCL1), mRNA.
Homo sapiens ribosomal protein S29 (RPS29), transcript variant 1, mRNA.
PREDICTED: Homo sapiens similar to Histone H2A.o (H2A/o) (H2A.2) (H2a-615) (LOC653610), mRNA.
Homo sapiens SFRS protein kinase 2 (SRPK2), transcript variant 1, mRNA.
Homo sapiens CDC-like kinase 4 (CLK4), mRNA.
PREDICTED: Homo sapiens similar to M-phase phosphoprotein, mpp8 (LOC642333), mRNA.
Homo sapiens membrane metallo-endopeptidase (MME), transcript variant 1, mRNA.
Homo sapiens phosphorylase, glycogen; liver (Hers disease, glycogen storage disease type VI) (PYGL), mRNA.
Homo sapiens ropporin 1-like (ROPN1L), mRNA.
Homo sapiens transducin-like enhancer of split 4 (E(sp1) homolog, Drosophila) (TLE4), mRNA.
Homo sapiens phenylalanine-tRNA synthetase-like, beta subunit (FARSLB), mRNA.
PREDICTED: Homo sapiens similar to protein immuno-reactive with anti-PTH polyclonal antibodies (LOC64984
Homo sapiens RNA binding motif (RNP1, RRM) protein 3 (RBM3), transcript variant 3, mRNA.
PREDICTED: Homo sapiens H3 histone, family 3A pseudogene, transcript variant 3 (LOC440926), misc RNA.
Homo sapiens interferon-induced protein with tetratricopeptide repeats 1 (IFIT1), transcript variant 2, mRNA.
PREDICTED: Homo sapiens similar to Williams Beuren syndrome chromosome region 19, transcript variant 8 (L
Homo sapiens ubiquitin specific peptidase 9, Y-linked (fat facets-like, Drosophila) (USP9Y), mRNA.
PREDICTED: Homo sapiens similar to Zinc finger protein 418 (LOC400721), mRNA.
Homo sapiens C-type lectin domain family 7, member A (CLEC7A), transcript variant 8, mRNA.
Homo sapiens Sin3A-associated protein, 30kDa (SAP30), mRNA.
Homo sapiens ubiquitously transcribed tetratricopeptide repeat gene, Y-linked (UTY), transcript variant 2, mR
Homo sapiens profilin 2 (PFN2), transcript variant 1, mRNA.
Homo sapiens MAX dimerization protein 1 (MXD1), mRNA.
Homo sapiens hydroxysteroid (17-beta) dehydrogenase 12 (HSD17B12), mRNA.
Homo sapiens splicing factor 3B, 14 kDa subunit (SF3B14), mRNA.
PREDICTED: Homo sapiens similar to H3 histone, family 3B (LOC347376), mRNA.
Homo sapiens hypothetical protein LOC283152 (LOC283152), mRNA.
Homo sapiens calcium/calmodulin-dependent protein kinase ID (CAMK1D), transcript variant 1, mRNA.
PREDICTED: Homo sapiens similar to CXYorf1-related protein (LOC653635), mRNA.
Homo sapiens eukaryotic translation initiation factor 2-alpha kinase 2 (EIF2AK2), mRNA.
Homo sapiens bridging integrator 3 (BIN3), mRNA.
Homo sapiens golgi phosphoprotein 3 (coat-protein) (GOLPH3), mRNA.
Homo sapiens exosome component 3 (EXOSC3), transcript variant 2, mRNA.
Homo sapiens RUN domain containing 2B (RUNDC2B), mRNA.
PREDICTED: Homo sapiens similar to CG7889-PA, transcript variant 2 (LOC647499), mRNA.

PREDICTED: Homo sapiens similar to peptidylprolyl isomerase A (cyclophilin A)-like 4 (LOC644591), mRNA.
Homo sapiens regulator of G-protein signalling 2, 24kDa (RGS2), mRNA.
PREDICTED: Homo sapiens similar to Ran-binding protein 2 (RanBP2) (Nuclear pore complex protein Nup358) (
PREDICTED: Homo sapiens hypothetical protein LOC645895 (LOC645895), mRNA.
Homo sapiens glia maturation factor, gamma (GMFG), mRNA.
PREDICTED: Homo sapiens similar to Ubiquinol-cytochrome c reductase complex 14 kDa protein (Complex III s
Homo sapiens chemokine-like factor (CKLF), transcript variant 3, mRNA.
Homo sapiens nudix (nucleoside diphosphate linked moiety X)-type motif 16 pseudogene (NUDT16P) on chror
PREDICTED: Homo sapiens similar to peptidylprolyl isomerase A (cyclophilin A)-like 4 (LOC653505), mRNA.
Homo sapiens NAD(P)H dehydrogenase, quinone 2 (NQO2), mRNA.
Homo sapiens phenazine biosynthesis-like protein domain containing (PBLD), transcript variant 1, mRNA.
Homo sapiens S100 calcium binding protein A6 (S100A6), mRNA.
Homo sapiens zinc finger protein 786 (ZNF786), mRNA.
PREDICTED: Homo sapiens family with sequence similarity 101, member B (FAM101B), mRNA.
Homo sapiens family with sequence similarity 101, member B (FAM101B), mRNA.
Homo sapiens zinc finger protein 354A (ZNF354A), mRNA.
Homo sapiens S100 calcium binding protein A9 (S100A9), mRNA.
PREDICTED: Homo sapiens hypothetical LOC440160 (LOC440160), mRNA.
Homo sapiens opioid growth factor receptor-like 1 (OGFRL1), mRNA.
Homo sapiens retinol binding protein 7, cellular (RBP7), mRNA.
Homo sapiens chromosome 22 open reading frame 34 (C22orf34), mRNA.
Homo sapiens poliovirus receptor-related 2 (herpesvirus entry mediator B) (PVRL2), transcript variant alpha, n
Homo sapiens hypothetical protein FLJ31951 (FLJ31951), mRNA.
Homo sapiens histidine ammonia-lyase (HAL), mRNA.
Homo sapiens arylsulfatase G (ARSG), mRNA.
Homo sapiens ubiquitin specific peptidase 10 (USP10), mRNA.
PREDICTED: Homo sapiens colony stimulating factor 2 receptor, alpha, low-affinity (granulocyte-macrophage)
Homo sapiens implantation-associated protein (DKFZp564K142), mRNA.
Homo sapiens exocyst complex component 8 (EXOC8), mRNA.
Homo sapiens PPAR binding protein (PPARBP), mRNA.
PREDICTED: Homo sapiens dehydrogenase/reductase (SDR family) member 8 (DHRS8), mRNA.
PREDICTED: Homo sapiens similar to nuclear pore membrane protein 121 (LOC728575), mRNA.
Homo sapiens leukotriene B4 receptor (LTB4R), mRNA.
Homo sapiens hexokinase 2 (HK2), mRNA.
Homo sapiens similar to 60S ribosomal protein L21 (LOC402176), mRNA.
Homo sapiens lemur tyrosine kinase 2 (LMTK2), mRNA.
Homo sapiens transforming, acidic coiled-coil containing protein 3 (TACC3), mRNA.
Homo sapiens lymphocyte antigen 96 (LY96), mRNA.
Homo sapiens beta-2-microglobulin (B2M), mRNA.
Homo sapiens chromosome 12 open reading frame 35 (C12orf35), mRNA.
Homo sapiens HECT domain containing 2 (HECTD2), transcript variant 2, mRNA.
Homo sapiens trinucleotide repeat containing 6B (TNRC6B), transcript variant 2, mRNA.
Homo sapiens zinc finger with KRAB and SCAN domains 1 (ZKSCAN1), mRNA.
Homo sapiens Fc fragment of IgG, low affinity IIIb, receptor (CD16b) (FCGR3B), mRNA.
Homo sapiens cAMP responsive element binding protein 5 (CREB5), transcript variant 4, mRNA.
Homo sapiens interleukin 17 receptor A (IL17RA), mRNA.
Homo sapiens protein tyrosine phosphatase, receptor type, N polypeptide 2 (PTPRN2), transcript variant 3, ml
Homo sapiens ring finger protein 130 (RNF130), mRNA.

Homo sapiens troponin I type 2 (skeletal, fast) (TNNI2), mRNA.
Homo sapiens S100 calcium binding protein A8 (S100A8), mRNA.
Homo sapiens folate receptor 3 (gamma) (FOLR3), mRNA.
Homo sapiens ankyrin repeat domain 30B (ANKRD30B), mRNA.
Homo sapiens cyclin-dependent kinase 5, regulatory subunit 1 (p35) (CDK5R1), mRNA.
Homo sapiens toll-like receptor 1 (TLR1), mRNA.
Homo sapiens KIAA1618 (KIAA1618), mRNA.
Homo sapiens ribosomal protein L26 (RPL26), mRNA.
Homo sapiens orosomucoid 2 (ORM2), mRNA.
Homo sapiens centrosomal protein 152kDa (CEP152), mRNA.
PREDICTED: Homo sapiens similar to NY-REN-7 antigen, transcript variant 4 (LOC653316), mRNA.
Homo sapiens protein phosphatase 1B (formerly 2C), magnesium-dependent, beta isoform (PPM1B), transcrip
Homo sapiens Kruppel-like factor 6 (KLF6), transcript variant 2, mRNA.
Homo sapiens cysteinyl leukotriene receptor 1 (CYSLTR1), mRNA.
PREDICTED: Homo sapiens similar to similar to RPL23AP7 protein (LOC644033), mRNA.
Homo sapiens yrdC domain containing (E. coli) (YRDC), mRNA.
Homo sapiens cytochrome P450, family 4, subfamily F, polypeptide 3 (CYP4F3), mRNA.
Homo sapiens phosphatase and actin regulator 1 (PHACTR1), mRNA.
Homo sapiens protein-L-isoaspartate (D-aspartate) O-methyltransferase domain containing 1 (PCMTD1), mRN
Homo sapiens hypothetical protein FLJ34047 (FLJ34047), mRNA.
Homo sapiens ribosomal protein S29 (RPS29), transcript variant 1, mRNA.
Homo sapiens centrosomal protein 27kDa (CEP27), mRNA.
PREDICTED: Homo sapiens hypothetical protein LOC644964 (LOC644964), mRNA.
Homo sapiens golgi autoantigen, golgin subfamily a, 2 (GOLGA2), mRNA.
PREDICTED: Homo sapiens similar to 40S ribosomal protein S29 (LOC643284), mRNA.
Homo sapiens UPF2 regulator of nonsense transcripts homolog (yeast) (UPF2), transcript variant 2, mRNA.
Homo sapiens RNA, 7SK, nuclear (RN7SK) on chromosome 6.
Homo sapiens ubiquitously transcribed tetratricopeptide repeat gene, Y-linked (UTY), transcript variant 3, mRI
Homo sapiens protein phosphatase 1, regulatory (inhibitor) subunit 12A (PPP1R12A), mRNA.
Homo sapiens RAB3 GTPase activating protein subunit 1 (catalytic) (RAB3GAP1), mRNA.
Homo sapiens chromosome 14 open reading frame 102 (C14orf102), transcript variant 1, mRNA.
Homo sapiens Fc fragment of IgA, receptor for (FCAR), transcript variant 9, mRNA.
Homo sapiens osteoclast-associated receptor (OSCAR), transcript variant 3, mRNA.
Homo sapiens kinesin family member 1B (KIF1B), transcript variant 2, mRNA.
Homo sapiens PHD finger protein 3 (PHF3), mRNA.
PREDICTED: Homo sapiens similar to Williams Beuren syndrome chromosome region 19, transcript variant 6 (l
Homo sapiens Mdm4, transformed 3T3 cell double minute 4, p53 binding protein (mouse) (MDM4), mRNA.
Homo sapiens enhancer of polycomb homolog 1 (Drosophila) (EPC1), mRNA.
Homo sapiens RAE1 RNA export 1 homolog (S. pombe) (RAE1), transcript variant 1, mRNA.
Homo sapiens pleckstrin homology, Sec7 and coiled-coil domains, binding protein (PSCDBP), mRNA.
PREDICTED: Homo sapiens hypothetical protein LOC643882 (LOC643882), mRNA.
Homo sapiens zinc finger protein 346 (ZNF346), mRNA.
Homo sapiens jun dimerization protein 2 (JDP2), mRNA.
Homo sapiens solute carrier family 2 (facilitated glucose transporter), member 11 (SLC2A11), transcript varian
Homo sapiens solute carrier family 5 (sodium/glucose cotransporter), member 9 (SLC5A9), mRNA.
PREDICTED: Homo sapiens similar to H3 histone, family 3B (LOC730740), mRNA.
Homo sapiens S100 calcium binding protein A12 (S100A12), mRNA.
Homo sapiens RCSD domain containing 1 (RCSD1), mRNA.

Homo sapiens colony stimulating factor 2 receptor, alpha, low-affinity (granulocyte-macrophage) (CSF2RA), tra
Homo sapiens succinate dehydrogenase complex, subunit A, flavoprotein pseudogene 1 (SDHALP1) on chromo
Homo sapiens antagonist of mitotic exit network 1 homolog (S. cerevisiae) (AMN1), mRNA.
Homo sapiens transmembrane 6 superfamily member 1 (TM6SF1), mRNA.
Homo sapiens chromosome 7 open reading frame 25 (C7orf25), mRNA.
PREDICTED: Homo sapiens similar to AI661453 protein (FLJ90086), mRNA.
Homo sapiens synaptojanin 2 (SYNJ2), mRNA.
Homo sapiens syntaxin 10 (STX10), mRNA.
Homo sapiens ring finger and FYVE-like domain containing 1 (RFFL), transcript variant 2, mRNA.
Homo sapiens aarF domain containing kinase 4 (ADCK4), mRNA.
Homo sapiens tripartite motif-containing 25 (TRIM25), mRNA.
Homo sapiens ankyrin repeat domain 44 (ANKRD44), mRNA.
Homo sapiens heme binding protein 2 (HEBP2), mRNA.
Homo sapiens eukaryotic translation initiation factor 2 alpha kinase 4 (EIF2AK4), mRNA.
Homo sapiens ribosomal protein L23 (RPL23), mRNA.
Homo sapiens chromosome Y open reading frame 15B (CYorf15B), mRNA.
PREDICTED: Homo sapiens similar to dicer1 (LOC647691), mRNA.
Homo sapiens tumor necrosis factor (ligand) superfamily, member 13b (TNFSF13B), mRNA.
Homo sapiens Fas (TNFRSF6)-associated via death domain (FADD), mRNA.
Homo sapiens regulator of G-protein signalling 18 (RGS18), mRNA.
Homo sapiens transporter 2, ATP-binding cassette, sub-family B (MDR/TAP) (TAP2), transcript variant 2, mRNA
Homo sapiens paraneoplastic antigen like 6A (PNMA6A), mRNA.
PREDICTED: Homo sapiens similar to Dihydrofolate reductase, transcript variant 1 (LOC643509), mRNA.
Homo sapiens serglycin (SRGN), mRNA.
Homo sapiens eomesodermin homolog (Xenopus laevis) (EOMES), mRNA.
Homo sapiens proteasome (prosome, macropain) subunit, alpha type, 1 (PSMA1), transcript variant 1, mRNA.
Homo sapiens endosulfine alpha (ENSA), transcript variant 8, mRNA.
Homo sapiens chromosome 10 open reading frame 119 (C10orf119), mRNA.
Homo sapiens hypoxia-inducible factor 1, alpha subunit (basic helix-loop-helix transcription factor) (HIF1A), tra
Homo sapiens RNA binding motif protein, X-linked-like 1 (RBMXL1), mRNA.
Homo sapiens ecotropic viral integration site 2B (EVI2B), mRNA.
Homo sapiens insulin receptor substrate 2 (IRS2), mRNA.
Homo sapiens alanyl (membrane) aminopeptidase (aminopeptidase N, aminopeptidase M, microsomal amino
PREDICTED: Homo sapiens membrane bound O-acyltransferase domain containing 1, transcript variant 1 (MBO
Homo sapiens histone deacetylase 4 (HDAC4), mRNA.
Homo sapiens zinc finger protein 585A (ZNF585A), transcript variant 1, mRNA.
Homo sapiens oxoeicosanoid (OXE) receptor 1 (OXER1), mRNA.
Homo sapiens mitogen-activated protein kinase kinase kinase 2 (MAP3K2), mRNA.
Homo sapiens RAP1A, member of RAS oncogene family (RAP1A), transcript variant 1, mRNA.
Homo sapiens hypothetical protein LOC552891 (LOC552891), mRNA.
Homo sapiens RNA binding motif protein 25 (RBM25), mRNA.
Homo sapiens actin related protein 2/3 complex, subunit 5, 16kDa (ARPC5), mRNA.
Homo sapiens similar to H3 histone, family 3B (LOC440093), mRNA.
Homo sapiens 5'-nucleotidase, cytosolic III (NT5C3), transcript variant 2, mRNA.
Homo sapiens ribosomal protein L32 (RPL32), transcript variant 3, mRNA.
Homo sapiens ankyrin repeat domain 55 (ANKRD55), transcript variant 1, mRNA.
PREDICTED: Homo sapiens similar to 60S ribosomal protein L14 (CAG-ISL 7), transcript variant 1 (LOC649821),
Homo sapiens bone marrow stromal cell antigen 1 (BST1), mRNA.

Homo sapiens chromobox homolog 1 (HP1 beta homolog Drosophila) (CBX1), mRNA.
PREDICTED: Homo sapiens similar to general transcription factor II I (LOC641978), mRNA.
PREDICTED: Homo sapiens similar to hypothetical protein MGC40405, transcript variant 1 (LOC653158), mRNA
Homo sapiens FLJ38717 protein (FLJ38717), mRNA.
Homo sapiens C-type lectin domain family 4, member E (CLEC4E), mRNA.
Homo sapiens chromosome 20 open reading frame 43 (C20orf43), mRNA.
Homo sapiens protein kinase, Y-linked (PRKY), mRNA.
Homo sapiens toll-like receptor 2 (TLR2), mRNA.
Homo sapiens mitochondrial carrier triple repeat 1 (MCART1), mRNA.
Homo sapiens nuclear factor of kappa light polypeptide gene enhancer in B-cells inhibitor, alpha (NFKBIA), mR
Homo sapiens leucine rich repeat neuronal 3 (LRRN3), mRNA.
Homo sapiens solute carrier family 26, member 8 (SLC26A8), transcript variant 1, mRNA.
Homo sapiens HECT, C2 and WW domain containing E3 ubiquitin protein ligase 2 (HECW2), mRNA.
Homo sapiens thymosin, beta 4, Y-linked (TMSB4Y), mRNA.
PREDICTED: Homo sapiens similar to RAN-binding protein 2-like 1 isoform 2, transcript variant 11 (LOC653596
Homo sapiens metallophosphoesterase 1 (MPPE1), mRNA.
Homo sapiens leucine-rich repeat kinase 2 (LRRK2), mRNA.
Homo sapiens transporter 2, ATP-binding cassette, sub-family B (MDR/TAP) (TAP2), transcript variant 1, mRNA
PREDICTED: Homo sapiens hypothetical protein FLJ40722, transcript variant 3 (FLJ40722), mRNA.
Homo sapiens interleukin 18 (interferon-gamma-inducing factor) (IL18), mRNA.
Homo sapiens phosphatidylinositol binding clathrin assembly protein (PICALM), transcript variant 1, mRNA.
Homo sapiens chromodomain helicase DNA binding protein 7 (CHD7), mRNA.
Homo sapiens cadherin 2, type 1, N-cadherin (neuronal) (CDH2), mRNA.
PREDICTED: Homo sapiens similar to pleckstrin homology domain containing, family M (with RUN domain) me
PREDICTED: Homo sapiens hypothetical protein MGC12760, transcript variant 2 (MGC12760), mRNA.
Homo sapiens protein phosphatase 1, regulatory (inhibitor) subunit 3D (PPP1R3D), mRNA.
Homo sapiens oxysterol binding protein-like 8 (OSBPL8), transcript variant 2, mRNA.
PREDICTED: Homo sapiens solute carrier family 25 (carnitine/acylcarnitine translocase), member 20 (SLC25A2
Homo sapiens interleukin 8 receptor, beta (IL8RB), mRNA.
Homo sapiens ankyrin repeat and SOCS box-containing 8 (ASB8), mRNA.
Homo sapiens butyrophilin-like 3 (BTNL3), transcript variant 2, mRNA.
Homo sapiens paraneoplastic antigen MA3 (PNMA3), mRNA.
Homo sapiens tubulin, alpha 4a (TUBA4A), mRNA.
Homo sapiens deltex 3-like (Drosophila) (DTX3L), mRNA.
PREDICTED: Homo sapiens hypothetical protein LOC647389 (LOC647389), mRNA.
Homo sapiens oxysterol binding protein-like 9 (OSBPL9), transcript variant 6, mRNA.
Homo sapiens G protein-coupled receptor 84 (GPR84), mRNA.
Homo sapiens SUMO1 pseudogene 1 (SUMO1P1) on chromosome 20.
Homo sapiens leiomodin 3 (fetal) (LMOD3), mRNA.
Homo sapiens transglutaminase 3 (E polypeptide, protein-glutamine-gamma-glutamyltransferase) (TGM3), m
Homo sapiens nephroblastoma overexpressed gene (NOV), mRNA.
Homo sapiens amyotrophic lateral sclerosis 2 (juvenile) chromosome region, candidate 14 (ALS2CR14), mRNA.
Homo sapiens adducin 3 (gamma) (ADD3), transcript variant 1, mRNA.
Homo sapiens centrosomal protein 63kDa (CEP63), transcript variant 1, mRNA.
Homo sapiens chemokine (C-X-C motif) ligand 1 (melanoma growth stimulating activity, alpha) (CXCL1), mRNA
Homo sapiens lines homolog 1 (Drosophila) (LINS1), transcript variant 6, mRNA.
Homo sapiens similar to zinc finger protein 469 (LOC441193), mRNA.
Homo sapiens PC4 and SFRS1 interacting protein 1 (PSIP1), transcript variant 2, mRNA.

Homo sapiens chromobox homolog 3 (HP1 gamma homolog, Drosophila) (CBX3), transcript variant 2, mRNA.
Homo sapiens cysteine-rich secretory protein LCCL domain containing 2 (CRISPLD2), mRNA.
PREDICTED: Homo sapiens similar to Proline-rich nuclear receptor coactivator 2, transcript variant 3 (LOC6531
Homo sapiens cytoskeleton-associated protein 4 (CKAP4), mRNA.
Homo sapiens dJ341D10.1 (novel protein) (dJ341D10.1), mRNA.
Homo sapiens F-box and leucine-rich repeat protein 13 (FBXL13), mRNA.
Homo sapiens BTAF1 RNA polymerase II, B-TFIID transcription factor-associated, 170kDa (Mot1 homolog, S. ce
Homo sapiens MAX-like protein X (MLX), transcript variant 3, mRNA.
Homo sapiens neuroplastin (NPTN), transcript variant beta, mRNA.
PREDICTED: Homo sapiens similar to H3 histone, family 2 isoform 2 (LOC653604), mRNA.
Homo sapiens cytochrome c oxidase subunit VIIb (COX7B), nuclear gene encoding mitochondrial protein, mRN
Homo sapiens POU domain, class 2, transcription factor 1 (POU2F1), mRNA.
PREDICTED: Homo sapiens similar to Fc receptor-like 5 (LOC729853), mRNA.
Homo sapiens BMX non-receptor tyrosine kinase (BMX), mRNA.
Homo sapiens matrix metallopeptidase 9 (gelatinase B, 92kDa gelatinase, 92kDa type IV collagenase) (MMP9),
Homo sapiens acyltransferase like 1 (AYTL1), mRNA.
Homo sapiens neutrophil cytosolic factor 2 (65kDa, chronic granulomatous disease, autosomal 2) (NCF2), mRN
Homo sapiens testis-specific transcript, Y-linked 15 (TTTY15) on chromosome Y.
Homo sapiens mitogen-activated protein kinase kinase kinase kinase 4 (MAP4K4), transcript variant 2, mRNA.
Homo sapiens arachidonate 5-lipoxygenase-activating protein (ALOX5AP), mRNA.
Homo sapiens proteasome (prosome, macropain) subunit, beta type, 9 (large multifunctional peptidase 2) (PS
Homo sapiens MCM8 minichromosome maintenance deficient 8 (S. cerevisiae) (MCM8), transcript variant 2, r
Homo sapiens SFRS protein kinase 1 (SRPK1), mRNA.
Homo sapiens CKLF-like MARVEL transmembrane domain containing 2 (CMTM2), mRNA.
Homo sapiens MOCO sulphurase C-terminal domain containing 1 (MOSC1), mRNA.
Homo sapiens IBR domain containing 2 (IBRDC2), mRNA.
Homo sapiens ankyrin repeat domain 22 (ANKRD22), mRNA.
Homo sapiens CUG triplet repeat, RNA binding protein 2 (CUGBP2), transcript variant 3, mRNA.
PREDICTED: Homo sapiens hypothetical protein LOC648226 (LOC648226), mRNA.
Homo sapiens S100 calcium binding protein P (S100P), mRNA.
Homo sapiens pregnancy specific beta-1-glycoprotein 9 (PSG9), mRNA.
Homo sapiens bromodomain and WD repeat domain containing 1 (BRWD1), transcript variant 2, mRNA.
Homo sapiens ankyrin repeat and BTB (POZ) domain containing 1 (ABTB1), transcript variant 2, mRNA.
Homo sapiens solute carrier family 36 (proton/amino acid symporter), member 4 (SLC36A4), mRNA.
Homo sapiens zinc finger protein 281 (ZNF281), mRNA.
Homo sapiens chromosome 1 open reading frame 108 (C1orf108), mRNA.
Homo sapiens acidic (leucine-rich) nuclear phosphoprotein 32 family, member A (ANP32A), mRNA.
PREDICTED: Homo sapiens hypothetical gene supported by AF147354 (LOC388789), mRNA.
Homo sapiens 4-aminobutyrate aminotransferase (ABAT), nuclear gene encoding mitochondrial protein, trans
PREDICTED: Homo sapiens zinc finger protein 486 (ZNF486), mRNA.
Homo sapiens chromodomain helicase DNA binding protein 8 (CHD8), mRNA.
PREDICTED: Homo sapiens KIAA0492 protein (KIAA0492), mRNA.
Homo sapiens hypothetical protein MGC4093 (MGC4093), mRNA.
Homo sapiens annexin A11 (ANXA11), transcript variant c, mRNA.
Homo sapiens peptidyl arginine deiminase, type IV (PADI4), mRNA.
Homo sapiens KIAA0319-like (KIAA0319L), transcript variant 2, mRNA.
Homo sapiens tudor domain containing 1 (TDRD1), mRNA.
Homo sapiens AT rich interactive domain 4A (RBP1-like) (ARID4A), transcript variant 3, mRNA.

Homo sapiens solute carrier family 5 (iodide transporter), member 8 (SLC5A8), mRNA.
Homo sapiens hypothetical protein FLJ20152 (FLJ20152), transcript variant 2, mRNA.
PREDICTED: Homo sapiens hypothetical protein FLJ14107 (FLJ14107), misc RNA.
Homo sapiens KIAA1706 protein (KIAA1706), mRNA.
Homo sapiens phosphatidylinositol-4-phosphate 5-kinase, type II, beta (PIP5K2B), transcript variant 2, mRNA.
Homo sapiens RAB35, member RAS oncogene family (RAB35), mRNA.
Homo sapiens aryl hydrocarbon receptor (AHR), mRNA.
Homo sapiens zinc finger, SWIM-type containing 1 (ZSWIM1), mRNA.
Homo sapiens ankyrin repeat and death domain containing 1A (ANKDD1A), mRNA.
Homo sapiens SP110 nuclear body protein (SP110), transcript variant c, mRNA.
Homo sapiens coronin, actin binding protein, 2A (CORO2A), transcript variant 1, mRNA.
PREDICTED: Homo sapiens similar to RAN-binding protein 2-like 1 isoform 2, transcript variant 10 (LOC653086
Homo sapiens interleukin 9 receptor (IL9R), transcript variant 1, mRNA.
Homo sapiens chromosome 21 open reading frame 55 (C21orf55), mRNA.
Homo sapiens pleiomorphic adenoma gene-like 1 (PLAGL1), transcript variant 3, mRNA.
Homo sapiens zinc finger protein 223 (ZNF223), mRNA.
Homo sapiens general transcription factor II, i, pseudogene 1 (GTF2IP1) on chromosome 7.
CR738550 Soares fetal liver spleen 1NFLS Homo sapiens cDNA clone IMAGp971E092 ; IMAGE:113723 5, mRNA
Homo sapiens cDNA FLJ40660 fis, clone THYMU2019686
Homo sapiens cDNA FLJ30935 fis, clone FEBRA2007176
tt27g09.x1 NCI_CGAP_GC6 Homo sapiens cDNA clone IMAGE:2242048 3, mRNA sequence
zv04h05.r1 Soares_NhHMPu_S1 Homo sapiens cDNA clone IMAGE:752697 5, mRNA sequence
BX108917 Soares fetal liver spleen 1NFLS Homo sapiens cDNA clone IMAGp998F09386, mRNA sequence
CM4-MT0361-060201-735-a04 MT0361 Homo sapiens cDNA, mRNA sequence
AGENCOURT_8585572 NIH_MGC_113 Homo sapiens cDNA clone IMAGE:6302814 5, mRNA sequence
DA385619 BRTHA2 Homo sapiens cDNA clone BRTHA2020660 5, mRNA sequence
DA572426 HEMBB1 Homo sapiens cDNA clone HEMBB1001157 5, mRNA sequence
il42e04.y1 HR85 islet Homo sapiens cDNA clone IMAGE:6032719 5, mRNA sequence
Homo sapiens mRNA; cDNA DKFZp667A182 (from clone DKFZp667A182)
Homo sapiens cDNA clone IMAGE:6277782, partial cds
AGENCOURT_8844418 NIH_MGC_141 Homo sapiens cDNA clone IMAGE:6385379 5, mRNA sequence
BX113731 Soares_NFL_T_GBC_S1 Homo sapiens cDNA clone IMAGp998E153815, mRNA sequence
UI-E-EJ0-aiq-m-17-0-UI.r1 UI-E-EJ0 Homo sapiens cDNA clone UI-E-EJ0-aiq-m-17-0-UI 5, mRNA sequence
CR746297 NCI_CGAP_Kid11 Homo sapiens cDNA clone IMAGp971P2385 ; IMAGE:2029602 5, mRNA sequence
AV645572 GLC Homo sapiens cDNA clone GLCABG03 3, mRNA sequence
df10f04.y1 Morton Fetal Cochlea Homo sapiens cDNA clone IMAGE:2483071 5, mRNA sequence
Homo sapiens cDNA FLJ42781 fis, clone BRAWH3005534
AGENCOURT_7761579 NIH_MGC_70 Homo sapiens cDNA clone IMAGE:6020085 5, mRNA sequence
UI-E-CL1-afc-h-16-0-UI.r1 UI-E-CL1 Homo sapiens cDNA clone UI-E-CL1-afc-h-16-0-UI 5, mRNA sequence
BX111927 Soares_multiple_sclerosis_2NbHMSP Homo sapiens cDNA clone IMAGp998G05619, mRNA sequenc
AGENCOURT_8353231 NIH_MGC_113 Homo sapiens cDNA clone IMAGE:6278225 5, mRNA sequence
zp47f07.s1 Stratagene HeLa cell s3 937216 Homo sapiens cDNA clone IMAGE:612613 3, mRNA sequence
UI-H-DI0-auw-h-24-0-UI.s1 NCI_CGAP_DI0 Homo sapiens cDNA clone IMAGE:5875271 3, mRNA sequence
AGENCOURT_13462296 NIH_MGC_187 Homo sapiens cDNA clone IMAGE:30318122 5, mRNA sequence
AGENCOURT_8803344 Lupski_sciatic_nerve Homo sapiens cDNA clone IMAGE:6198982 5, mRNA sequence
Homo sapiens cDNA clone IMAGE:5270591
UI-1-BB1p-axy-f-04-0-UI.s2 NCI_CGAP_Pl6 Homo sapiens cDNA clone UI-1-BB1p-axy-f-04-0-UI 3, mRNA sequer
UI-E-EJ0-ahi-k-07-0-UI.r2 UI-E-EJ0 Homo sapiens cDNA clone UI-E-EJ0-ahi-k-07-0-UI 5, mRNA sequence

BX099079 Soares fetal liver spleen 1NFLS Homo sapiens cDNA clone IMAGp998O14606, mRNA sequence
xv24e03.x1 Soares_NFL_T_GBC_S1 Homo sapiens cDNA clone IMAGE:2814076 3, mRNA sequence
AGENCOURT_10480778 NIH_MGC_107 Homo sapiens cDNA clone IMAGE:6646806 5, mRNA sequence
wi12b07.x1 NCI_CGAP_Co16 Homo sapiens cDNA clone IMAGE:2390005 3, mRNA sequence
Homo sapiens cDNA clone IMAGE:5272804
BX109404 NCI_CGAP_Br2 Homo sapiens cDNA clone IMAGp998G234083, mRNA sequence
PM0-NN1173-231000-001-h11 NN1173 Homo sapiens cDNA, mRNA sequence
BX100504 Soares_fetal_heart_NbHH19W Homo sapiens cDNA clone IMAGp998D19788, mRNA sequence
DA395916 BRTHA2 Homo sapiens cDNA clone BRTHA2035482 5, mRNA sequence
Homo sapiens partial mRNA; ID YG39-1C
ny62g05.s1 NCI_CGAP_GCB1 Homo sapiens cDNA clone IMAGE:1282904 3, mRNA sequence
Homo sapiens cDNA FLJ26044 fis, clone PRS01852
Homo sapiens mRNA; cDNA DKFZp313O229 (from clone DKFZp313O229)
cs75c04.y1 Human Retinal pigment epithelium/choroid cDNA (Un-normalized, unamplified): cs Homo sapiens
UI-E-CK1-afn-i-01-0-UI.s2 UI-E-CK1 Homo sapiens cDNA clone UI-E-CK1-afn-i-01-0-UI 3, mRNA sequence
BX092006 NCI_CGAP_Kid11 Homo sapiens cDNA clone IMAGp998M175911 ; IMAGE:2380600, mRNA sequenc
df27f11.w1 Morton Fetal Cochlea Homo sapiens cDNA clone IMAGE:2484644 3, mRNA sequence
FNPATC04 FNP Homo sapiens cDNA, mRNA sequence
AV651069 GLC Homo sapiens cDNA clone GLCCLD10 3, mRNA sequence
wd35b02.x1 Soares_NFL_T_GBC_S1 Homo sapiens cDNA clone IMAGE:2330091 3, mRNA sequence
603183417F1 NIH_MGC_121 Homo sapiens cDNA clone IMAGE:5247269 5, mRNA sequence
BX117171 NCI_CGAP_Co3 Homo sapiens cDNA clone IMAGp998J142299, mRNA sequence
nw90g12.s1 NCI_CGAP_Pr12 Homo sapiens cDNA clone IMAGE:1253926, mRNA sequence
yy19a06.s1 Soares melanocyte 2NbHM Homo sapiens cDNA clone IMAGE:271666 3 similar to PIR:S22049 S22(
wq37a10.x1 NCI_CGAP_GC6 Homo sapiens cDNA clone IMAGE:2473434 3 similar to contains element MER18
hv70f04.x1 NCI_CGAP_Lu24 Homo sapiens cDNA clone IMAGE:3178783 3, mRNA sequence
Homo sapiens cDNA FLJ30923 fis, clone FEBRA2006491
Homo sapiens mRNA; cDNA DKFZp586O1318 (from clone DKFZp586O1318)
CM2-HT0242-251099-024-a09 HT0242 Homo sapiens cDNA, mRNA sequence
Homo sapiens mRNA; cDNA DKFZp762F0616 (from clone DKFZp762F0616)
zn90c01.r1 Stratagene lung carcinoma 937218 Homo sapiens cDNA clone IMAGE:565440 5, mRNA sequence
Homo sapiens cDNA FLJ37202 fis, clone BRALZ2006734
Homo sapiens cDNA FLJ42306 fis, clone TRACH2001646
Homo sapiens cDNA: FLJ21679 fis, clone COL09221
SM011178 Brain 3 EST Homo sapiens cDNA clone ID_11178 3', mRNA sequence
Homo sapiens mRNA; cDNA DKFZp686J19209 (from clone DKFZp686J19209)
603089645F1 NIH_MGC_120 Homo sapiens cDNA clone IMAGE:5228627 5, mRNA sequence
zr87e09.r1 NCI_CGAP_GCB1 Homo sapiens cDNA clone IMAGE:682696 5, mRNA sequence
AGENCOURT_14538855 NIH_MGC_191 Homo sapiens cDNA clone IMAGE:30418792 5, mRNA sequence
AV705309 ADB Homo sapiens cDNA clone ADBBWE05 5, mRNA sequence
601590965F1 NIH_MGC_7 Homo sapiens cDNA clone IMAGE:3944752 5, mRNA sequence
BX111162 Soares_testis_NHT Homo sapiens cDNA clone IMAGp998E074158, mRNA sequence
Homo sapiens thioredoxin (TXN), mRNA.

Ontology_Component

pothetical LOC441257, transcript variant 2 (LOC643862), mRNA.

:

intracellular [goid 5622] [evidence IEA]; cytosolic small ribosomal subunit (sensu Eukaryota) [goid 5843] [pmid

ribosome [goid 5840] [evidence IEA]; intracellular [goid 5622] [evidence IEA]; cytosolic small ribosomal subuni
integral to membrane [goid 16021] [evidence IEA]; membrane fraction [goid 5624] [pmid 15297306] [evidence
heterotrimeric G-protein complex [goid 5834] [evidence IEA]; integral to membrane [goid 16021] [evidence IE

membrane [goid 16020] [evidence IEA]; extracellular region [goid 5576] [evidence IEA]

nucleus [goid 5634] [evidence IEA]
\.

integral to membrane [goid 16021] [evidence IEA]
extracellular space [goid 5615] [evidence NR]
protein phosphatase type 2A complex [goid 159] [pmid 1849734] [evidence ISS]

plasma membrane [goid 5886] [pmid 7754374] [evidence TAS]; integral to plasma membrane [goid 5887] [pm
DNA-directed RNA polymerase II, core complex [goid 5665] [pmid 8797801] [evidence TAS]; nucleus [goid 563

membrane [goid 16020] [evidence IEA]; integral to membrane [goid 16021] [evidence IEA]
nucleus [goid 5634] [pmid 9687510] [evidence IEP]
membrane [goid 16020] [evidence IEA]; integral to membrane [goid 16021] [evidence IEA]

chromosome [goid 5694] [evidence IEA]; nucleus [goid 5634] [evidence IEA]; nucleosome [goid 786] [evidence

\.

ribosome [goid 5840] [evidence IEA]; intracellular [goid 5622] [evidence IEA]; cytosolic small ribosomal subuni

LOC653352), mRNA.
integral to plasma membrane [goid 5887] [pmid 2555171] [evidence TAS]; membrane [goid 16020] [evidence

nucleus [goid 5634] [evidence IEA]

membrane [goid 16020] [evidence IEA]; integral to membrane [goid 16021] [evidence IEA]

intracellular [goid 5622] [evidence IEA]
cytoplasm [goid 5737] [evidence IEA]
voltage-gated calcium channel complex [goid 5891] [pmid 8071363] [evidence TAS]; integral to membrane [gc
mitochondrion [goid 5739] [pmid 3278312] [evidence TAS]; cytoplasm [goid 5737] [evidence IEA]
cytosol [goid 5829] [pmid 9538874] [evidence TAS]; soluble fraction [goid 5625] [pmid 9538874] [evidence TA

membrane [goid 16020] [evidence IEA]; integral to membrane [goid 16021] [evidence IEA]
intracellular [goid 5622] [pmid 11256614] [evidence IDA]

intracellular [goid 5622] [evidence IEA]

nucleus [goid 5634] [pmid 11137288] [evidence NAS]

endoplasmic reticulum [goid 5783] [evidence IEA]; extracellular space [goid 5615] [pmid 12368295] [evidence

membrane [goid 16020] [evidence IEA]; extracellular matrix (sensu Metazoa) [goid 5578] [evidence IEA]; anch

cell fraction [goid 267] [evidence NAS]
nucleus [goid 5634] [evidence IEA]
nucleus [goid 5634] [evidence NAS]; intracellular [goid 5622] [evidence IEA]

nucleus [goid 5634] [pmid 9874765] [evidence IDA]; histone deacetylase complex [goid 118] [pmid 9874765] [
nucleus [goid 5634] [pmid 11279057] [evidence ISS]; Golgi apparatus [goid 5794] [pmid 11279057] [evidence I

nucleus [goid 5634] [pmid 7693701] [evidence TAS]
nucleus [goid 5634] [evidence IEA]; lamin filament [goid 5638] [pmid 7557986] [evidence TAS]
plasma membrane [goid 5886] [pmid 8615752] [evidence TAS]; Golgi apparatus [goid 5794] [pmid 8615752] [e
{NA.

integral to plasma membrane [goid 5887] [pmid 9062191] [evidence TAS]; membrane [goid 16020] [evidence
integral to plasma membrane [goid 5887] [pmid 2971451] [evidence TAS]; membrane [goid 16020] [evidence
.

contractile ring [goid 5826] [pmid 9203580] [evidence ISS]; nucleus [goid 5634] [pmid 10942595] [evidence ID
membrane [goid 16020] [evidence IEA]; integral to membrane [goid 16021] [evidence IEA]

nucleus [goid 5634] [evidence ISS]; membrane [goid 16020] [evidence IEA]; cytoplasm [goid 5737] [pmid 1451
membrane [goid 16020] [evidence IEA]; endoplasmic reticulum [goid 5783] [evidence IEA]
plasma membrane [goid 5886] [pmid 1660144] [evidence TAS]; integral to plasma membrane [goid 5887] [pm

nucleus [goid 5634] [evidence IEA]; transcription factor TFIID complex [goid 5669] [evidence NR]; intracellular

intracellular [goid 5622] [evidence IEA]
membrane [goid 16020] [evidence IEA]
extracellular region [goid 5576] [pmid 11481438] [evidence NAS]; extracellular space [goid 5615] [evidence IE

plasma membrane [goid 5886] [pmid 10799517] [evidence TAS]; cAMP-dependent protein kinase complex [gc
cytosol [goid 5829] [evidence NR]
RNA.
nucleus [goid 5634] [evidence IEA]

integral to membrane [goid 16021] [evidence IEA]
nucleus [goid 5634] [evidence IEA]
lysosome [goid 5764] [evidence IEA]
membrane [goid 16020] [evidence IEA]; peroxisome [goid 5777] [evidence IEA]; integral to membrane [goid 1
extracellular space [goid 5615] [evidence NR]
membrane [goid 16020] [evidence IEA]; integral to membrane [goid 16021] [evidence IEA]

extracellular region [goid 5576] [pmid 14718574] [evidence NAS]
membrane [goid 16020] [evidence IEA]; integral to membrane [goid 16021] [evidence IEA]

nucleus [goid 5634] [evidence IEA]
endoplasmic reticulum [goid 5783] [evidence IEA]; extracellular space [goid 5615] [pmid 12368295] [evidence

membrane [goid 16020] [evidence IEA]; mitochondrion [goid 5739] [pmid 15685448] [evidence IDA]; integral

RNA.
extracellular region [goid 5576] [evidence IEA]
chromosome [goid 5694] [evidence IEA]; nucleus [goid 5634] [evidence IEA]; nucleosome [goid 786] [pmid 91
membrane [goid 16020] [evidence IEA]; integral to membrane [goid 16021] [evidence IEA]

nucleus [goid 5634] [evidence IEA]

nucleus [goid 5634] [pmid 10820245] [evidence TAS]; cytoplasm [goid 5737] [pmid 10820245] [evidence TAS]

plasma membrane [goid 5886] [pmid 10749881] [evidence TAS]; integral to membrane [goid 16021] [pmid 16

integral to plasma membrane [goid 5887] [pmid 2307934] [evidence TAS]; membrane [goid 16020] [evidence
nucleus [goid 5634] [evidence IEA]; intracellular [goid 5622] [evidence IEA]
alpha-ketoglutarate dehydrogenase complex (sensu Eukaryota) [goid 5947] [pmid 11839747] [evidence TAS];
integral to membrane [goid 16021] [evidence IEA]; membrane [goid 16020] [evidence IEA]; extracellular space
membrane [goid 16020] [evidence IEA]; integral to membrane [goid 16021] [evidence IEA]
nucleus [goid 5634] [pmid 7798274] [evidence TAS]
Golgi membrane [goid 139] [pmid 12682071] [evidence IDA]; membrane [goid 16020] [evidence IEA]; secretor
clathrin coat of coated pit [goid 30132] [evidence IEA]; clathrin vesicle coat [goid 30125] [evidence IEA]
ribosome [goid 5840] [evidence IEA]; intracellular [goid 5622] [evidence IEA]; cytosolic small ribosomal subuni

nucleus [goid 5634] [pmid 9472028] [evidence IDA]; cytoplasm [goid 5737] [pmid 9472028] [evidence IDA]
nucleus [goid 5634] [evidence IEA]

plasma membrane [goid 5886] [pmid 1660144] [evidence TAS]; integral to plasma membrane [goid 5887] [pm

nucleus [goid 5634] [pmid 1303260] [evidence NAS]
cytoplasm [goid 5737] [pmid 10049785] [evidence TAS]; soluble fraction [goid 5625] [pmid 10049785] [eviden
1), mRNA.

cytoplasm [goid 5737] [pmid 3753936] [evidence NAS]
OC442609), mRNA.

integral to membrane [goid 16021] [pmid 11567029] [evidence TAS]
nucleus [goid 5634] [evidence IEA]; histone deacetylase complex [goid 118] [pmid 9651585] [evidence TAS]
nucleus [goid 5634] [evidence IEA]
actin cytoskeleton [goid 15629] [evidence NAS]
nucleus [goid 5634] [pmid 8425218] [evidence TAS]
membrane [goid 16020] [evidence IEA]; integral to membrane [goid 16021] [evidence IEA]; endoplasmic reticu
nucleus [goid 5634] [evidence IEA]

nucleus [goid 5634] [evidence IEA]; calcium- and calmodulin-dependent protein kinase complex [goid 5954] [p

intracellular [goid 5622] [evidence IEA]
cytoskeleton [goid 5856] [evidence IEA]; cytoplasm [goid 5737] [evidence IEA]
membrane [goid 16020] [evidence IEA]
cytoplasmic exosome (RNase complex) [goid 177] [pmid 11110791] [evidence IDA]; nuclear exosome (RNase c

(Nucleoporin Nup358) (358 kDa nucleoporin) (P270), transcript variant 7 (LOC653489), mRNA.

intracellular [goid 5622] [evidence IEA]
ubunit VI) (QP-C) (LOC644969), mRNA.
integral to membrane [goid 16021] [pmid 11415443] [evidence IDA]; extracellular region [goid 5576] [pmid 11
nosome 3.

ruffle [goid 1726] [pmid 10913138] [evidence IDA]; nucleus [goid 5634] [pmid 10913138] [evidence IDA]; cyto
nucleus [goid 5634] [pmid 11256614] [evidence IDA]; intracellular [goid 5622] [evidence IEA]

nucleus [goid 5634] [evidence IEA]; intracellular [goid 5622] [evidence IEA]
extracellular space [goid 5615] [evidence NR]

membrane [goid 16020] [evidence IEA]

plasma membrane [goid 5886] [pmid 9657005] [evidence TAS]; integral to membrane [goid 16021] [evidence

cytosol [goid 5829] [evidence NR]; cytoplasm [goid 5737] [evidence IEA]
endoplasmic reticulum [goid 5783] [pmid 12461688] [evidence IDA]

(CSF2RA), mRNA.
membrane [goid 16020] [evidence IEA]; integral to membrane [goid 16021] [evidence IEA]; endoplasmic reticu

nucleus [goid 5634] [pmid 10235267] [evidence IDA]; mediator complex [goid 119] [pmid 10198638] [evidenc

integral to plasma membrane [goid 5887] [pmid 9177352] [evidence TAS]; membrane [goid 16020] [evidence
mitochondrial outer membrane [goid 5741] [pmid 7622509] [evidence NAS]; membrane [goid 16020] [eviden
ribosome [goid 5840] [evidence IEA]; intracellular [goid 5622] [evidence IEA]
integral to membrane [goid 16021] [pmid 12393858] [evidence IDA]; membrane [goid 16020] [evidence IEA]

plasma membrane [goid 5886] [pmid 10359581] [evidence TAS]
extracellular region [goid 5576] [evidence NAS]

intracellular [goid 5622] [evidence IEA]

nucleus [goid 5634] [pmid 7557990] [evidence NAS]; intracellular [goid 5622] [evidence IEA]
membrane [goid 16020] [evidence IEA]
nucleus [goid 5634] [pmid 8378084] [evidence IC]; intracellular [goid 5622] [evidence IEA]
integral to plasma membrane [goid 5887] [pmid 9367539] [evidence NAS]; membrane [goid 16020] [evidence
integral to plasma membrane [goid 5887] [pmid 8798755] [evidence TAS]; membrane [goid 16020] [evidence
integral to membrane [goid 16021] [pmid 13679316] [evidence ISS]; membrane [goid 16020] [evidence IEA]

extracellular space [goid 5615] [evidence NR]
membrane fraction [goid 5624] [pmid 8110752] [evidence TAS]; extrinsic to membrane [goid 19898] [pmid 81
cellular component unknown [goid 8372] [evidence ND]
cyclin-dependent protein kinase 5 activator complex [goid 16533] [evidence IEA]; membrane fraction [goid 56
plasma membrane [goid 5886] [pmid 9435236] [evidence TAS]; phagocytic vesicle [goid 45335] [pmid 110957

large ribosomal subunit [goid 15934] [evidence IEA]; intracellular [goid 5622] [evidence IEA]
extracellular space [goid 5615] [pmid 2970990] [evidence TAS]

protein serine/threonine phosphatase complex [goid 8287] [evidence IEA]
nucleus [goid 5634] [pmid 9689109] [evidence TAS]; intracellular [goid 5622] [evidence IEA]
membrane fraction [goid 5624] [pmid 10391245] [evidence TAS]; integral to plasma membrane [goid 5887] [p

endoplasmic reticulum [goid 5783] [evidence IEA]; microsome [goid 5792] [evidence IEA]; membrane [goid 16

A.

ribosome [goid 5840] [evidence IEA]; intracellular [goid 5622] [evidence IEA]; cytosolic small ribosomal subuni
centrosome [goid 5813] [pmid 14654843] [evidence IDA]

Golgi apparatus [goid 5794] [pmid 15229288] [evidence IDA]

cytoplasm [goid 5737] [pmid 14636577] [evidence IDA]; nucleus [goid 5634] [pmid 14636577] [evidence IDA]

nucleus [goid 5634] [evidence IEA]
actin cytoskeleton [goid 15629] [evidence NR]
soluble fraction [goid 5625] [pmid 9852129] [evidence IDA]

plasma membrane [goid 5886] [pmid 2258698] [evidence TAS]; integral to plasma membrane [goid 5887] [pm

microtubule [goid 5874] [evidence IEA]; microtubule associated complex [goid 5875] [evidence ISS]; cytoplasm

.OC653829), mRNA.
nucleus [goid 5634] [pmid 9226370] [evidence NAS]; intracellular [goid 5622] [evidence IEA]
nucleus [goid 5634] [pmid 10976108] [evidence IDA]
cytoskeleton [goid 5856] [pmid 9256445] [evidence TAS]; nucleus [goid 5634] [pmid 9370289] [evidence TAS];
membrane [goid 16020] [evidence IEA]; cytoplasm [goid 5737] [pmid 12606567] [evidence IDA]; cell cortex [g

nucleus [goid 5634] [pmid 10488071] [evidence TAS]; nucleolus [goid 5730] [pmid 10488071] [evidence TAS];
nucleus [goid 5634] [evidence IEA]
membrane [goid 16020] [evidence IEA]; integral to membrane [goid 16021] [evidence IEA]
membrane [goid 16020] [evidence IEA]; integral to membrane [goid 16021] [evidence IEA]

insoluble fraction [goid 5626] [pmid 7626002] [evidence TAS]; cytosol [goid 5829] [pmid 7626002] [evidence T

integral to plasma membrane [goid 5887] [pmid 2555171] [evidence TAS]; membrane [goid 16020] [evidence
›some 3.

membrane [goid 16020] [evidence NAS]; integral to membrane [goid 16021] [evidence IEA]

membrane [goid 16020] [evidence IEA]; integral to membrane [goid 16021] [evidence IEA]; Golgi membrane [
membrane [goid 16020] [evidence IEA]

cytosolic ribosome (sensu Eukaryota) [goid 5830] [evidence ISS]; intracellular [goid 5622] [evidence NAS]
ribosome [goid 5840] [pmid 1874450] [evidence NAS]; intracellular [goid 5622] [evidence IEA]

integral to plasma membrane [goid 5887] [evidence NR]; membrane [goid 16020] [evidence IEA]; extracellula
cytoplasm [goid 5737] [evidence NR]

TAP complex [goid 42825] [pmid 12202157] [evidence NAS]; membrane [goid 16020] [evidence IEA]; endoplas

nucleus [goid 5634] [evidence IEA]
nucleus [goid 5634] [pmid 7681138] [evidence TAS]; cytosol [goid 5829] [evidence IEA]; polysome [goid 5844]

nucleus [goid 5634] [pmid 15261140] [evidence IDA]

integral to plasma membrane [goid 5887] [pmid 1903357] [evidence TAS]; membrane [goid 16020] [evidence

ER-Golgi intermediate compartment [goid 5793] [pmid 15308636] [evidence IDA]; integral to plasma membra
)AT1), mRNA.
nucleus [goid 5634] [evidence NAS]; histone deacetylase complex [goid 118] [pmid 12711221] [evidence TAS];
nucleus [goid 5634] [evidence IEA]; intracellular [goid 5622] [evidence IEA]
integral to membrane [goid 16021] [evidence IEA]; membrane [goid 16020] [evidence IEA]
nucleus [goid 5634] [evidence IEA]
membrane [goid 16020] [evidence IEA]; intracellular [goid 5622] [evidence IEA]

Arp2/3 protein complex [goid 5885] [pmid 9230079] [evidence TAS]; cytoskeleton [goid 5856] [evidence IEA];
chromosome [goid 5694] [evidence IEA]; nucleus [goid 5634] [evidence IEA]; nucleosome [goid 786] [evidence
endoplasmic reticulum [goid 5783] [pmid 8557639] [evidence IDA]; cytoplasm [goid 5737] [pmid 8557639] [ev
ribosome [goid 5840] [evidence IEA]; intracellular [goid 5622] [evidence IEA]

mRNA.
membrane [goid 16020] [evidence IEA]; extrinsic to membrane [goid 19898] [pmid 8202488] [evidence TAS]

chromatin [goid 785] [evidence IEA]; nuclear heterochromatin [goid 5720] [pmid 9169582] [evidence TAS]; nu

\.

membrane [goid 16020] [evidence IEA]; integral to membrane [goid 16021] [evidence IEA]

plasma membrane [goid 5886] [pmid 9435236] [evidence TAS]; integral to plasma membrane [goid 5887] [pm
integral to membrane [goid 16021] [evidence IEA]; mitochondrion [goid 5739] [evidence IEA]
nucleus [goid 5634] [pmid 7679069] [evidence IDA]; cytoplasm [goid 5737] [pmid 3140380] [evidence IDA]
membrane [goid 16020] [evidence IEA]; integral to membrane [goid 16021] [evidence IEA]
integral to membrane [goid 16021] [evidence IEA]
intracellular [goid 5622] [evidence IEA]
cytoskeleton [goid 5856] [evidence IEA]; cytoplasm [goid 5737] [evidence IEA]
), mRNA.

TAP complex [goid 42825] [pmid 12202157] [evidence NAS]; membrane [goid 16020] [evidence IEA]; endoplas

extracellular region [goid 5576] [pmid 11598150] [evidence TAS]; extracellular space [goid 5615] [evidence IE/
coated pit [goid 5905] [pmid 10436022] [evidence IDA]
nucleus [goid 5634] [evidence IEA]; chromatin [goid 785] [evidence IEA]
membrane [goid 16020] [evidence IEA]; integral to membrane [goid 16021] [evidence IEA]
mber 1; adapter protein 162, transcript variant 4 (LOC440456), mRNA.

)), mRNA.
integral to plasma membrane [goid 5887] [pmid 10438939] [evidence TAS]; membrane [goid 16020] [pmid 10

membrane [goid 16020] [evidence IEA]; integral to membrane [goid 16021] [evidence IEA]

microtubule [goid 5874] [evidence IEA]; protein complex [goid 43234] [evidence IEA]

membrane [goid 16020] [evidence IEA]; integral to membrane [goid 16021] [evidence NAS]

actin cytoskeleton [goid 15629] [pmid 11256614] [evidence IDA]; cytoskeleton [goid 5856] [evidence IEA]
cornified envelope [goid 1533] [evidence ISS]; extrinsic to internal side of plasma membrane [goid 31234] [pm
extracellular region [goid 5576] [evidence IEA]

membrane [goid 16020] [pmid 8893809] [evidence TAS]; cytoskeleton [goid 5856] [evidence IEA]
centrosome [goid 5813] [pmid 14654843] [evidence IDA]
extracellular region [goid 5576] [evidence IEA]; extracellular space [goid 5615] [pmid 10881932] [evidence TAS

nucleus [goid 5634] [evidence IEA]

nucleus [goid 5634] [pmid 8663349] [evidence TAS]; chromatin [goid 785] [evidence IEA]
extracellular region [goid 5576] [evidence IEA]; transport vesicle [goid 30133] [pmid 11256614] [evidence IDA]
05), mRNA.
membrane fraction [goid 5624] [pmid 8314870] [evidence TAS]; integral to membrane [goid 16021] [pmid 831

cytoplasm [goid 5737] [evidence ISS]
nucleus [goid 5634] [evidence NAS]
nucleus [goid 5634] [pmid 10918583] [evidence IDA]; cytoplasm [goid 5737] [pmid 10918583] [evidence IDA]
integral to membrane [goid 16021] [evidence IEA]; postsynaptic membrane [goid 45211] [evidence ISS]; presy

membrane [goid 16020] [evidence IEA]; mitochondrion [goid 5739] [evidence IEA]; integral to membrane [goi
nucleus [goid 5634] [pmid 11891224] [evidence IDA]

extracellular matrix (sensu Metazoa) [goid 5578] [evidence IEA]; extracellular space [goid 5615] [pmid 225189
membrane [goid 16020] [evidence IEA]; integral to membrane [goid 16021] [evidence IEA]
cytosol [goid 5829] [evidence NR]; soluble fraction [goid 5625] [evidence NR]

membrane fraction [goid 5624] [evidence NR]; membrane [goid 16020] [evidence IEA]; integral to membrane
cytosol [goid 5829] [evidence IEA]; proteasome core complex (sensu Eukaryota) [goid 5839] [evidence IEA]
nucleus [goid 5634] [evidence IEA]
nucleus [goid 5634] [pmid 11509566] [evidence IDA]; cytoplasm [goid 5737] [pmid 11509566] [evidence IDA]
membrane [goid 16020] [evidence IEA]; integral to membrane [goid 16021] [evidence IEA]; extracellular space

integral to membrane [goid 16021] [evidence ISS]; ubiquitin ligase complex [goid 151] [pmid 12853982] [evide

nucleus [goid 5634] [pmid 15632002] [evidence IDA]; cytoplasm [goid 5737] [pmid 15632002] [evidence TAS]
membrane fraction [goid 5624] [pmid 2537311] [evidence TAS]; extracellular region [goid 5576] [evidence NA
nucleus [goid 5634] [evidence IEA]

DNA-directed RNA polymerase II, core complex [goid 5665] [pmid 10448078] [evidence TAS]; nucleus [goid 56

nucleus [goid 5634] [pmid 11729309] [evidence IDA]; perinuclear region [goid 48471] [pmid 11555662] [evide

mitochondrion [goid 5739] [evidence ISS]

chromatin [goid 785] [evidence IEA]; nucleus [goid 5634] [evidence NAS]

nucleus [goid 5634] [pmid 7508441] [evidence TAS]; nucleoplasm [goid 5654] [pmid 12577318] [evidence NAS
nucleus [goid 5634] [evidence IEA]
membrane [goid 16020] [evidence IEA]

chromatin [goid 785] [evidence IEA]; transcriptional repressor complex [goid 17053] [pmid 12724404] [eviden

membrane [goid 16020] [evidence IEA]; integral to membrane [goid 16021] [evidence IEA]

intracellular [goid 5622] [evidence IEA]
endoplasmic reticulum [goid 5783] [evidence IEA]; membrane [goid 16020] [evidence IEA]
membrane [goid 16020] [evidence IEA]
nucleus [goid 5634] [pmid 10395741] [evidence IDA]

nucleus [goid 5634] [pmid 7693701] [evidence TAS]

), mRNA.
integral to plasma membrane [goid 5887] [pmid 1376929] [evidence TAS]; membrane [goid 16020] [evidence

nucleus [goid 5634] [evidence IEA]; intracellular [goid 5622] [evidence IEA]
nucleus [goid 5634] [evidence NAS]; intracellular [goid 5622] [evidence IEA]

\ sequence

:e

ıce

cDNA clone cs75c04 5, mRNA sequence

:e

)49 retrovirus-related reverse transcriptase - rabbit ;contains L1.t2 L1 repetitive element ;, mRNA sequence
repetitive element ;, mRNA sequence

Ontology_Process

protein biosynthesis [goid 6412] [pmid 15883184] [evidence IC]
cell differentiation [goid 30154] [evidence IEA]; apoptosis [goid 6915] [evidence IEA]; DNA repair [goid 6281] [
protein biosynthesis [goid 6412] [pmid 12082018] [evidence NAS]
transport [goid 6810] [pmid 9827529] [evidence TAS]
G-protein coupled receptor protein signaling pathway [goid 7186] [evidence IEA]; protein folding [goid 6457] [

skeletal development [goid 1501] [pmid 8955270] [evidence TAS]; central nervous system development [goid
regulation of transcription, DNA-dependent [goid 6355] [evidence IEA]

pyridine nucleotide biosynthesis [goid 19363] [evidence IEA]; cell-cell signaling [goid 7267] [pmid 8289818] [ev

blood coagulation [goid 7596] [evidence IEA]; fibrinolysis [goid 42730] [evidence IEA]; chemotaxis [goid 6935]
protein amino acid dephosphorylation [goid 6470] [pmid 1849734] [evidence ISS]

id 1842498] [evidence TAS]; integral to membrane [goid 16021] [evidence IEA]
transcription [goid 6350] [evidence IEA]; transcription from RNA polymerase II promoter [goid 6366] [pmid 87

homophilic cell adhesion [goid 7156] [evidence IEA]
response to external stimulus [goid 9605] [pmid 9873047] [evidence IEP]; histone phosphorylation [goid 1657

nucleosome assembly [goid 6334] [evidence IEA]; chromosome organization and biogenesis (sensu Eukaryota)

protein biosynthesis [goid 6412] [pmid 8908372] [evidence TAS]; cell proliferation [goid 8283] [pmid 8407955]
positive regulation of I-kappaB kinase/NF-kappaB cascade [goid 43123] [evidence ISS]

IEA]

regulation of transcription, DNA-dependent [goid 6355] [evidence IEA]; transcription [goid 6350] [evidence IE

G-protein coupled receptor protein signaling pathway [goid 7186] [evidence IEA]; signal transduction [goid 71

small GTPase mediated signal transduction [goid 7264] [evidence IEA]
antibiotic biosynthesis [goid 17000] [evidence IEA]
calcium ion transport [goid 6816] [evidence IEA]; ion transport [goid 6811] [evidence IEA]; synaptic transmissi
electron transport [goid 6118] [evidence IEA]
phototransduction [goid 7602] [pmid 9538874] [evidence TAS]; visual perception [goid 7601] [pmid 10329014

intracellular signaling cascade [goid 7242] [evidence IEA]

ubiquitin-dependent protein catabolism [goid 6511] [evidence IEA]

ubiquitin cycle [goid 6512] [evidence IEA]

cell cycle [goid 7049] [evidence IEA]; cell division [goid 51301] [evidence IEA]
regulation of transcription, DNA-dependent [goid 6355] [pmid 11137288] [evidence NAS]; development [goid

metabolism [goid 8152] [evidence IEA]; heparan sulfate proteoglycan metabolism [goid 30201] [pmid 123682
cell differentiation [goid 30154] [evidence IEA]; myoblast cell fate determination [goid 7518] [pmid 7756174]

cell differentiation [goid 30154] [evidence IEA]; axonogenesis [goid 7409] [evidence ISS]

metabolism [goid 8152] [evidence IEA]
signal transduction [goid 7165] [evidence NAS]
cellular defense response [goid 6968] [pmid 1644857] [evidence TAS]; regulation of transcription, DNA-depen
regulation of transcription, DNA-dependent [goid 6355] [evidence NAS]; transcription [goid 6350] [evidence IE

transcription [goid 6350] [evidence IEA]; negative regulation of transcription, DNA-dependent [goid 45892] [p
chloride transport [goid 6821] [pmid 11279057] [evidence ISS]
negative regulation of cell proliferation [goid 8285] [pmid 8632892] [evidence TAS]
regulation of transcription, DNA-dependent [goid 6355] [evidence IEA]; transcription [goid 6350] [evidence IE

blood coagulation [goid 7596] [evidence IEA]; G-protein coupled receptor protein signaling pathway [goid 718

pyridine nucleotide biosynthesis [goid 19363] [evidence IEA]
metabolism [goid 8152] [evidence IEA]; steroid biosynthesis [goid 6694] [evidence IEA]

cell surface receptor linked signal transduction [goid 7166] [pmid 9062191] [evidence TAS]
response to virus [goid 9615] [pmid 2954953] [evidence TAS]; signal transduction [goid 7165] [pmid 2971451]

cell cycle [goid 7049] [evidence IEA]; cytokinesis [goid 910] [pmid 9203580] [evidence ISS]
immune response [goid 6955] [evidence IEA]

physiological process [goid 7582] [pmid 9630216] [evidence TAS]; cell motility [goid 6928] [pmid 9630216] [ev
autophagy [goid 6914] [evidence IEA]; small GTPase mediated signal transduction [goid 7264] [evidence IEA];
cell-cell signaling [goid 7267] [pmid 2521388] [evidence TAS]
nitrogen compound metabolism [goid 6807] [evidence IEA]
[goid 5622] [evidence IEA]

small GTPase mediated signal transduction [goid 7264] [evidence IEA]; regulation of small GTPase mediated si
nitrogen compound metabolism [goid 6807] [evidence IEA]; cell motility [goid 6928] [pmid 9790769] [evidenc
regulation of cell proliferation [goid 42127] [pmid 11481438] [evidence NAS]; negative regulation of cell grow

ubiquitin-dependent protein catabolism [goid 6511] [evidence IEA]
cell motility [goid 6928] [pmid 10947064] [evidence TAS]; transport [goid 6810] [evidence IEA]; cell-cell signali

intracellular signaling cascade [goid 7242] [pmid 2540040] [evidence TAS]; protein amino acid phosphorylatio

ubiquitin cycle [goid 6512] [evidence IEA]
regulation of transcription, DNA-dependent [goid 6355] [evidence IEA]; anterior compartment specification [g
metabolism [goid 8152] [evidence IEA]; fructose 2,6-bisphosphate metabolism [goid 6003] [evidence IEA]

regulation of transcription, DNA-dependent [goid 6355] [evidence IEA]; transcription [goid 6350] [evidence IE
ceramide metabolism [goid 6672] [pmid 8955159] [evidence TAS]; fatty acid metabolism [goid 6631] [evidenc
metabolism [goid 8152] [evidence IEA]; digestion [goid 7586] [pmid 1531127] [evidence TAS]; lipid metabolism
pregnancy [goid 7565] [pmid 7794280] [evidence TAS]

cell motility [goid 6928] [evidence NR]; blood coagulation [goid 7596] [evidence NR]; cell adhesion [goid 715
transport [goid 6810] [evidence IEA]
glycoprotein catabolism [goid 6516] [pmid 9811929] [evidence TAS]
regulation of transcription, DNA-dependent [goid 6355] [evidence IEA]; development [goid 7275] [pmid 8094
metabolism [goid 8152] [evidence IEA]; heparan sulfate proteoglycan metabolism [goid 30201] [pmid 123682
meiosis [goid 7126] [evidence IEA]
anti-apoptosis [goid 6916] [pmid 15685448] [evidence IMP]; immune response [goid 6955] [pmid 3017706] [e

nucleosome assembly [goid 6334] [pmid 9119399] [evidence NAS]; chromosome organization and biogenesis
cell differentiation [goid 30154] [evidence IEA]; angiogenesis [goid 1525] [evidence IEA]; cell motility [goid 69

DNA repair [goid 6281] [evidence IEA]

transcription from RNA polymerase II promoter [goid 6366] [pmid 9630226] [evidence TAS]; tyrosine phospho

cell motility [goid 6928] [pmid 1689240] [evidence NAS]; blood coagulation [goid 7596] [pmid 8392005] [evide

immune response [goid 6955] [pmid 2307934] [evidence TAS]; signal transduction [goid 7165] [pmid 2307934
regulation of transcription, DNA-dependent [goid 6355] [evidence IEA]; transcription [goid 6350] [evidence IE/
branched chain family amino acid catabolism [goid 9083] [pmid 11839747] [evidence TAS]; protein amino acic
chemotaxis [goid 6935] [evidence IEA]; sensory perception [goid 7600] [evidence IEA]
metabolism [goid 8152] [evidence IEA]
apoptosis [goid 6915] [pmid 10828065] [evidence TAS]; DNA repair [goid 6281] [pmid 7973727] [evidence TAS
regulation of neurotransmitter secretion [goid 46928] [evidence ISS]; neurotransmitter transport [goid 6836] |
morphogenesis [goid 9653] [pmid 8733128] [evidence TAS]; receptor mediated endocytosis [goid 6898] [pmid
protein biosynthesis [goid 6412] [pmid 15883184] [evidence IC]

protein kinase cascade [goid 7243] [pmid 9472028] [evidence IDA]; RNA splicing [goid 8380] [pmid 9472028] [
protein amino acid phosphorylation [goid 6468] [evidence IEA]

cell-cell signaling [goid 7267] [pmid 2521388] [evidence TAS]
glycogen metabolism [goid 5977] [evidence IEA]; carbohydrate metabolism [goid 5975] [evidence IEA]

regulation of transcription [goid 45449] [evidence IEA]; regulation of transcription, DNA-dependent [goid 6355
protein biosynthesis [goid 6412] [pmid 10049785] [evidence TAS]; phenylalanyl-tRNA aminoacylation [goid 64

RNA processing [goid 6396] [pmid 8634703] [evidence TAS]

immune response [goid 6955] [evidence IEA]

ubiquitin-dependent protein catabolism [goid 6511] [evidence IEA]; spermatogenesis [goid 7283] [pmid 93846

phagocytosis, recognition [goid 6910] [pmid 11567029] [evidence IDA]; cell recognition [goid 8037] [pmid 115
regulation of transcription, DNA-dependent [goid 6355] [evidence IEA]; transcription [goid 6350] [evidence IE/

regulation of actin polymerization and/or depolymerization [goid 8064] [evidence NAS]; cytoskeleton organiz
cell proliferation [goid 8283] [pmid 7829091] [evidence TAS]; regulation of transcription, DNA-dependent [goi
metabolism [goid 8152] [evidence IEA]; steroid biosynthesis [goid 6694] [evidence IEA]
nuclear mRNA splicing, via spliceosome [goid 398] [evidence IEA]

protein amino acid phosphorylation [goid 6468] [evidence IEA]

immune response [goid 6955] [evidence IEA]; apoptosis [goid 6915] [evidence NR]; regulation of transcriptior
barrier septum formation [goid 917] [evidence IEA]; protein localization [goid 8104] [pmid 11274158] [eviden

rRNA processing [goid 6364] [pmid 11110791] [evidence IDA]

regulation of G-protein coupled receptor protein signaling pathway [goid 8277] [pmid 10747990] [evidence TA

protein amino acid phosphorylation [goid 6468] [pmid 7598724] [evidence TAS]

macrophage chemotaxis [goid 48246] [pmid 11415443] [evidence IDA]; lymphocyte chemotaxis [goid 48247] [

electron transport [goid 6118] [evidence IEA]
biosynthesis [goid 9058] [evidence IEA]
cell-cell signaling [goid 7267] [evidence NR]; positive regulation of fibroblast proliferation [goid 48146] [pmid
regulation of transcription, DNA-dependent [goid 6355] [evidence IEA]

transcription [goid 6350] [evidence IEA]; regulation of transcription from RNA polymerase II promoter [goid 63
cell-cell signaling [goid 7267] [pmid 3313057] [evidence TAS]; inflammatory response [goid 6954] [pmid 33130

transport [goid 6810] [evidence IEA]

homophilic cell adhesion [goid 7156] [evidence ISS]

biosynthesis [goid 9058] [evidence IEA]; histidine catabolism [goid 6548] [evidence IEA]
metabolism [goid 8152] [evidence IEA]
ubiquitin-dependent protein catabolism [goid 6511] [evidence IEA]

ılum [goid 5783] [evidence IEA]
protein transport [goid 15031] [evidence IEA]; exocytosis [goid 6887] [evidence IEA]
transcription initiation from RNA polymerase II promoter [goid 6367] [pmid 12218053] [evidence IDA]; fat cell

cell motility [goid 6928] [pmid 9177352] [evidence TAS]; G-protein signaling, coupled to IP3 second messenger
glycolysis [goid 6096] [evidence IEA]
protein biosynthesis [goid 6412] [evidence IEA]

cellular defense response [goid 6968] [pmid 10359581] [evidence TAS]; cell surface receptor linked signal tran
immune response [goid 6955] [pmid 1737380] [evidence NAS]

ubiquitin cycle [goid 6512] [evidence IEA]; oxygen transport [goid 15671] [evidence IEA]

regulation of transcription, DNA-dependent [goid 6355] [pmid 7557990] [evidence NAS]; transcription [goid 63
immune response [goid 6955] [pmid 2139735] [evidence TAS]
positive regulation of transcription, DNA-dependent [goid 45893] [pmid 8378084] [evidence IDA]; transcriptio
cell surface receptor linked signal transduction [goid 7166] [pmid 9367539] [evidence NAS]
protein amino acid dephosphorylation [goid 6470] [pmid 8798755] [evidence TAS]
apoptosis [goid 6915] [evidence IEA]

regulation of muscle contraction [goid 6937] [evidence NR]
inflammatory response [goid 6954] [pmid 3313057] [evidence TAS]
folic acid transport [goid 15884] [pmid 8110752] [evidence TAS]
biological process unknown [goid 4] [evidence ND]
brain development [goid 7420] [pmid 10915792] [evidence NAS]; cell proliferation [goid 8283] [pmid 8090221
positive regulation of interleukin-6 biosynthesis [goid 45410] [pmid 12077222] [evidence ISS]; positive regulat

protein biosynthesis [goid 6412] [evidence IEA]
acute-phase response [goid 6953] [pmid 2970990] [evidence TAS]

protein amino acid dephosphorylation [goid 6470] [pmid 9684878] [evidence TAS]
B cell differentiation [goid 30183] [pmid 9000136] [evidence NAS]; cell growth [goid 16049] [pmid 9000136] [e
respiratory gaseous exchange [goid 7585] [pmid 10391245] [evidence TAS]; defense response [goid 6952] [pm

leukotriene metabolism [goid 6691] [pmid 8486631] [evidence TAS]; electron transport [goid 6118] [evidence

protein modification [goid 6464] [evidence IEA]

protein biosynthesis [goid 6412] [pmid 15883184] [evidence IC]

regulation of translation [goid 6445] [pmid 16488880] [evidence TAS]; mRNA catabolism, nonsense-mediated

regulation of muscle contraction [goid 6937] [evidence NR]
regulation of GTPase activity [goid 43087] [pmid 10859313] [evidence IDA]

immune response [goid 6955] [pmid 10835685] [evidence TAS]

neuromuscular synaptic transmission [goid 7274] [evidence ISS]; anterograde axon cargo transport [goid 8089
regulation of transcription, DNA-dependent [goid 6355] [evidence IEA]; development [goid 7275] [pmid 1185[illegible]

protein stabilization [goid 50821] [pmid 10608892] [evidence IEP]; apoptosis [goid 6915] [pmid 14660608] [ev
chromatin modification [goid 16568] [evidence IEA]; regulation of transcription, DNA-dependent [goid 6355] [
mitotic spindle assembly [goid 51227] [pmid 15851029] [evidence IDA]
regulation of cell adhesion [goid 30155] [pmid 12606567] [evidence IDA]

apoptosis [goid 6915] [pmid 10488071] [evidence TAS]; RNA processing [goid 6396] [pmid 10488071] [eviden[illegible]
regulation of transcription, DNA-dependent [goid 6355] [evidence IEA]
transport [goid 6810] [evidence IEA]; carbohydrate transport [goid 8643] [evidence IEA]
ion transport [goid 6811] [evidence IEA]; sodium ion transport [goid 6814] [evidence IEA]

xenobiotic metabolism [goid 6805] [pmid 11522286] [evidence IDA]; inflammatory response [goid 6954] [pmi[illegible]

IEA]

protein transport [goid 15031] [evidence IEA]
ubiquitin cycle [goid 6512] [evidence IEA]; apoptosis [goid 6915] [evidence IEA]

regulation of translational initiation [goid 6446] [evidence ISS]; protein biosynthesis [goid 6412] [evidence IEA]
protein biosynthesis [goid 6412] [evidence IEA]

immune response [goid 6955] [evidence IEA]; cell proliferation [goid 8283] [pmid 10908663] [evidence TAS]; s
antimicrobial humoral response (sensu Vertebrata) [goid 19735] [evidence NR]; induction of apoptosis via de
negative regulation of signal transduction [goid 9968] [evidence IEA]
immune response [goid 6955] [evidence IEA]; cytosol to ER transport [goid 46967] [pmid 11133832] [evidence

morphogenesis [goid 9653] [pmid 9888994] [evidence TAS]; regulation of transcription, DNA-dependent [goid
ubiquitin-dependent protein catabolism [goid 6511] [evidence IEA]
transport [goid 6810] [pmid 9653196] [evidence TAS]; response to nutrient [goid 7584] [pmid 9653196] [evide

positive regulation of transcription [goid 45941] [pmid 9887100] [evidence IDA]; regulation of transcription, D

IEA]
glucose metabolism [goid 6006] [pmid 9495343] [evidence TAS]; signal transduction [goid 7165] [pmid 767508
angiogenesis [goid 1525] [evidence IEA]; cell differentiation [goid 30154] [evidence IEA]

B cell differentiation [goid 30183] [pmid 12711221] [evidence TAS]; transcription [goid 6350] [evidence IEA]; r
regulation of transcription, DNA-dependent [goid 6355] [evidence IEA]; transcription [goid 6350] [evidence IE
G-protein coupled receptor protein signaling pathway [goid 7186] [pmid 12065583] [evidence NAS]; signal tra
protein amino acid phosphorylation [goid 6468] [pmid 8621389] [evidence TAS]
small GTPase mediated signal transduction [goid 7264] [evidence IEA]; cell cycle [goid 7049] [evidence IEA]

nuclear mRNA splicing, via spliceosome [goid 398] [evidence NAS]
cell motility [goid 6928] [pmid 9230079] [evidence TAS]; regulation of actin filament polymerization [goid 308
nucleosome assembly [goid 6334] [evidence IEA]; chromosome organization and biogenesis (sensu Eukaryota
nucleotide metabolism [goid 9117] [evidence IEA]; pyrimidine nucleoside metabolism [goid 6213] [pmid 1094
protein biosynthesis [goid 6412] [evidence IEA]

development [goid 7275] [pmid 8202488] [evidence TAS]; humoral immune response [goid 6959] [pmid 8202

chromatin assembly or disassembly [goid 6333] [evidence IEA]

immune response [goid 6955] [evidence IEA]; antimicrobial humoral response (sensu Vertebrata) [goid 19735]

protein amino acid phosphorylation [goid 6468] [pmid 9154127] [evidence TAS]
induction of apoptosis [goid 6917] [pmid 10426996] [evidence TAS]; innate immune response [goid 45087] [ev
transport [goid 6810] [evidence IEA]
cytoplasmic sequestering of NF-kappaB [goid 7253] [evidence NAS]; apoptosis [goid 6915] [pmid 10747850] [e

transport [goid 6810] [evidence IEA]
ubiquitin cycle [goid 6512] [evidence IEA]
regulation of actin polymerization and/or depolymerization [goid 8064] [pmid 8838802] [evidence TAS]; seque

small GTPase mediated signal transduction [goid 7264] [evidence IEA]; protein amino acid phosphorylation [go
protein complex assembly [goid 6461] [pmid 12047747] [evidence NAS]; immune response [goid 6955] [evide

immune response [goid 6955] [pmid 8999896] [evidence TAS]; chemokine biosynthesis [goid 42033] [pmid 11
protein complex assembly [goid 6461] [pmid 8643484] [evidence TAS]; receptor mediated endocytosis [goid 6
chromatin assembly or disassembly [goid 6333] [evidence IEA]
homophilic cell adhesion [goid 7156] [evidence IEA]; cell adhesion [goid 7155] [pmid 2384753] [evidence TAS]

glycogen metabolism [goid 5977] [evidence IEA]; carbohydrate metabolism [goid 5975] [evidence IEA]
lipid transport [goid 6869] [evidence IEA]; steroid metabolism [goid 8202] [evidence IEA]

neutrophil activation [goid 42119] [pmid 10878382] [evidence IDA]; G-protein signaling, coupled to IP3 second
intracellular signaling cascade [goid 7242] [evidence IEA]
lipid metabolism [goid 6629] [pmid 10429365] [evidence TAS]

microtubule-based movement [goid 7018] [evidence IEA]; protein polymerization [goid 51258] [evidence IEA]

lipid transport [goid 6869] [evidence IEA]; steroid metabolism [goid 8202] [evidence IEA]
G-protein coupled receptor protein signaling pathway [goid 7186] [evidence IEA]; signal transduction [goid 71

skin development [goid 43588] [evidence ISS]; keratinocyte differentiation [goid 30216] [pmid 12850301] [evi
regulation of cell growth [goid 1558] [evidence IEA]

negative regulation of cell proliferation [goid 8285] [pmid 9551928] [evidence TAS]; chemotaxis [goid 6935] [p

regulation of transcription, DNA-dependent [goid 6355] [evidence IEA]; transcription [goid 6350] [evidence IE

chromatin modification [goid 16568] [evidence IEA]; regulation of transcription, DNA-dependent [goid 6355] [

.4870] [evidence TAS]

ubiquitin cycle [goid 6512] [evidence IEA]
negative regulation of transcription [goid 16481] [pmid 9488487] [evidence NAS]
regulation of transcription [goid 45449] [evidence IEA]; regulation of transcription, DNA-dependent [goid 6355
positive regulation of long-term neuronal synaptic plasticity [goid 48170] [evidence ISS]; homophilic cell adhes

electron transport [goid 6118] [evidence IEA]
regulation of transcription, DNA-dependent [goid 6355] [evidence IEA]; negative regulation of transcription [g

mesoderm development [goid 7498] [pmid 9520419] [evidence TAS]; intracellular signaling cascade [goid 724
peptidoglycan metabolism [goid 270] [evidence IEA]; macrophage differentiation [goid 30225] [pmid 2251898
metabolism [goid 8152] [evidence IEA]; phospholipid biosynthesis [goid 8654] [evidence IEA]
cellular defense response [goid 6968] [pmid 1692159] [evidence TAS]; superoxide metabolism [goid 6801] [ev

protein kinase cascade [goid 7243] [pmid 9890973] [evidence IDA]; response to stress [goid 6950] [pmid 9890
leukotriene biosynthesis [goid 19370] [evidence IEA]; inflammatory response [goid 6954] [pmid 2300173] [evi
immune response [goid 6955] [evidence IEA]; ubiquitin-dependent protein catabolism [goid 6511] [evidence I
DNA replication initiation [goid 6270] [evidence IEA]; transcription [goid 6350] [evidence IEA]; DNA replication
protein kinase cascade [goid 7243] [pmid 11509566] [evidence IDA]; regulation of mRNA processing [goid 506
chemotaxis [goid 6935] [evidence IEA]

ubiquitin cycle [goid 6512] [evidence IEA]; protein ubiquitination during ubiquitin-dependent protein catabolis

neuromuscular junction development [goid 7528] [evidence NR]; RNA processing [goid 6396] [pmid 9887331]

defense response [goid 6952] [evidence NAS]; pregnancy [goid 7565] [pmid 7794280] [evidence TAS]
regulation of transcription, DNA-dependent [goid 6355] [evidence IEA]; transcription [goid 6350] [evidence IE
protein biosynthesis [goid 6412] [evidence IEA]

transcription [goid 6350] [evidence IEA]; regulation of transcription, DNA-dependent [goid 6355] [evidence IE

intracellular signaling cascade [goid 7242] [pmid 8192856] [evidence TAS]; nucleocytoplasmic transport [goid

neurotransmitter catabolism [goid 42135] [pmid 15528998] [evidence NAS]; behavior [goid 7610] [pmid 1191

chromatin modification [goid 16568] [evidence IEA]; chromatin assembly or disassembly [goid 6333] [evidenc

immune response [goid 6955] [pmid 7508441] [evidence TAS]
chromatin modification [goid 16568] [evidence IEA]; transcription [goid 6350] [evidence IEA]; regulation of tra
homophilic cell adhesion [goid 7156] [evidence IEA]

transcription [goid 6350] [evidence IEA]; chromatin assembly or disassembly [goid 6333] [evidence IEA]; negat

ion transport [goid 6811] [evidence IEA]; sodium ion transport [goid 6814] [evidence IEA]

cell surface receptor linked signal transduction [goid 7166] [pmid 9038203] [evidence TAS]
small GTPase mediated signal transduction [goid 7264] [evidence IEA]; protein transport [goid 15031] [eviden
response to xenobiotic stimulus [goid 9410] [pmid 7961644] [evidence IDA]; transcription from RNA polymera

signal transduction [goid 7165] [evidence IEA]
regulation of transcription, DNA-dependent [goid 6355] [evidence IEA]; transcription [goid 6350] [evidence IE
nitrogen compound metabolism [goid 6807] [evidence IEA]; glutamine biosynthesis [goid 6542] [evidence IEA]

cell proliferation [goid 8283] [pmid 1376929] [evidence TAS]; signal transduction [goid 7165] [pmid 1376929]
protein folding [goid 6457] [evidence IEA]
positive regulation of transcription from RNA polymerase II promoter [goid 45944] [pmid 15888726] [evidenc
regulation of transcription, DNA-dependent [goid 6355] [evidence IEA]

Ontology_Function

structural constituent of ribosome [goid 3735] [pmid 15883184] [evidence IDA]
protein binding [goid 5515] [pmid 15383276] [evidence IPI]
structural constituent of ribosome [goid 3735] [pmid 15883184] [evidence IDA]
ATPase activity, coupled to transmembrane movement of substances [goid 42626] [evidence IEA]; organic ani
GTPase activity [goid 3924] [pmid 7665596] [evidence TAS]; heat shock protein binding [goid 31072] [evidence

transferase activity [goid 16740] [evidence IEA]; nucleotide binding [goid 166] [evidence IEA]; ATP binding [goi
sequence-specific DNA binding [goid 43565] [evidence IEA]; transcription factor activity [goid 3700] [evidence

transferase activity, transferring glycosyl groups [goid 16757] [evidence IEA]; cytokine activity [goid 5125] [pm

serine-type endopeptidase activity [goid 4252] [evidence IEA]; kinase activity [goid 16301] [evidence IEA]; plas
protein phosphatase type 2A regulator activity [goid 8601] [pmid 8392071] [evidence TAS]; calcium ion bindin
binding [goid 5488] [evidence IEA]
protein binding [goid 5515] [pmid 15205336] [evidence IPI]
transferase activity [goid 16740] [evidence IEA]; DNA-directed RNA polymerase activity [goid 3899] [pmid 879

calcium ion binding [goid 5509] [evidence IEA]
transferase activity [goid 16740] [evidence IEA]; metallopeptidase activity [goid 8237] [evidence IEA]; ATP binc
receptor activity [goid 4872] [evidence IEA]; calcium ion binding [goid 5509] [evidence NAS]

DNA binding [goid 3677] [evidence IEA]
electron carrier activity [goid 9055] [evidence IEA]; protein disulfide oxidoreductase activity [goid 15035] [evic

RNA binding [goid 3723] [pmid 8908372] [evidence TAS]; metal ion binding [goid 46872] [evidence IEA]; zinc ic
receptor activity [goid 4872] [evidence IEA]

receptor activity [goid 4872] [pmid 1358805] [evidence TAS]; hematopoietin/interferon-class (D200-domain) c

zinc ion binding [goid 8270] [pmid 9576949] [evidence NAS]; metal ion binding [goid 46872] [evidence IEA]; pr
calcium ion binding [goid 5509] [evidence IEA]
receptor activity [goid 4872] [evidence IEA]; rhodopsin-like receptor activity [goid 1584] [evidence IEA]
hydrolase activity [goid 16787] [evidence IEA]
guanyl-nucleotide exchange factor activity [goid 5085] [evidence IEA]
oxidoreductase activity [goid 16491] [evidence IEA]; calcium ion binding [goid 5509] [evidence IEA]
voltage-gated calcium channel activity [goid 5245] [pmid 8071363] [evidence TAS]; ion channel activity [goid 5
disulfide oxidoreductase activity [goid 15036] [evidence IEA]; FAD binding [goid 50660] [evidence IEA]; dihydro
] [evidence TAS]; synaptic transmission [goid 7268] [pmid 9538874] [evidence TAS]

peptidase activity [goid 8233] [evidence IEA]; ubiquitin thiolesterase activity [goid 4221] [evidence IEA]; cystei

ubiquitin-protein ligase activity [goid 4842] [evidence IEA]
zinc ion binding [goid 8270] [evidence IEA]; metal ion binding [goid 46872] [evidence IEA]; protein binding [goi

sequence-specific DNA binding [goid 43565] [evidence IEA]; transcription factor activity [goid 3700] [pmid 111
zinc ion binding [goid 8270] [evidence IEA]; nucleic acid binding [goid 3676] [evidence IEA]
hydrolase activity [goid 16787] [evidence IEA]; sulfuric ester hydrolase activity [goid 8484] [evidence IEA]; aryl
binding [goid 5488] [evidence IEA]

GPI anchor binding [goid 48503] [evidence IEA]

oxidoreductase activity [goid 16491] [evidence IEA]
hydrolase activity [goid 16787] [evidence IEA]; 3',5'-cyclic-nucleotide phosphodiesterase activity [goid 4114] [e
DNA binding [goid 3677] [evidence IEA]
transcription factor activity [goid 3700] [evidence NAS]; DNA binding [goid 3677] [evidence IEA]

protein binding [goid 5515] [pmid 15652350] [evidence IPI]
chloride channel activity [goid 5254] [pmid 11279057] [evidence ISS]
SH3/SH2 adaptor activity [goid 5070] [pmid 8632892] [evidence TAS]; protein binding [goid 5515] [pmid 8632[illegible]
zinc ion binding [goid 8270] [evidence IEA]; metal ion binding [goid 46872] [evidence IEA]; hematopoietin/inte
structural molecule activity [goid 5198] [pmid 7557986] [evidence TAS]
thrombin receptor activity [goid 15057] [evidence IEA]; receptor binding [goid 5102] [pmid 8615752] [evidenc

kinase activity [goid 16301] [evidence IEA]; ATP binding [goid 5524] [evidence IEA]; transferase activity [goid 1
oxidoreductase activity [goid 16491] [evidence IEA]

cytokine binding [goid 19955] [evidence IEA]; receptor activity [goid 4872] [evidence IEA]; interferon-gamma r

GTPase activity [goid 3924] [pmid 9203580] [evidence ISS]; nucleotide binding [goid 166] [evidence IEA]; GTP b
sugar binding [goid 5529] [evidence IEA]

oxidoreductase activity [goid 16491] [evidence IEA]; oxidoreductase activity, acting on single donors with inco
GTP binding [goid 5525] [evidence IEA]; nucleotide binding [goid 166] [evidence IEA]
zinc ion binding [goid 8270] [evidence IEA]; metal ion binding [goid 46872] [evidence IEA]; neprilysin activity [g
zinc ion binding [goid 8270] [evidence IEA]; metal ion binding [goid 46872] [evidence IEA]; beta-ureidopropion
RNA polymerase II transcription factor activity [goid 3702] [evidence NR]; single-stranded RNA binding [goid 3

guanyl-nucleotide exchange factor activity [goid 5085] [evidence IEA]; protein binding [goid 5515] [pmid 9748
hydrolase activity, acting on carbon-nitrogen (but not peptide) bonds, in linear amides [goid 16811] [evidence
cytokine activity [goid 5125] [pmid 11481438] [evidence NAS]

zinc ion binding [goid 8270] [evidence IEA]; metal ion binding [goid 46872] [evidence IEA]; peptidase activity [g
thiol-disulfide exchange intermediate activity [goid 30508] [pmid 10947064] [evidence TAS]; protein disulfide
hydrolase activity [goid 16787] [evidence IEA]; acid phosphatase activity [goid 3993] [evidence IEA]

kinase activity [goid 16301] [evidence IEA]; cAMP-dependent protein kinase regulator activity [goid 8603] [pm
hydrolase activity [goid 16787] [evidence IEA]; 5'-nucleotidase activity [goid 8253] [pmid 7999131] [evidence T
ligase activity [goid 16874] [evidence IEA]; ubiquitin-protein ligase activity [goid 4842] [evidence IEA]; protein
sequence-specific DNA binding [goid 43565] [evidence IEA]; transcription factor activity [goid 3700] [evidence
kinase activity [goid 16301] [evidence IEA]; transferase activity [goid 16740] [evidence IEA]; hydrolase activity

receptor activity [goid 4872] [evidence IEA]
transferase activity, transferring acyl groups, acyl groups converted into alkyl on transfer [goid 46912] [eviden
ligase activity [goid 16874] [evidence IEA]; magnesium ion binding [goid 287] [evidence IEA]; long-chain-fatty-a

signal transducer activity [goid 4871] [pmid 8751720] [evidence TAS]; structural molecule activity [goid 5198]
transporter activity [goid 5215] [evidence IEA]
hyaluronoglucosaminidase activity [goid 4415] [pmid 9811929] [evidence TAS]
sequence-specific DNA binding [goid 43565] [evidence IEA]; transcription factor activity [goid 3700] [evidence
hydrolase activity [goid 16787] [evidence IEA]; sulfuric ester hydrolase activity [goid 8484] [evidence IEA]; aryl
RNA binding [goid 3723] [evidence IEA]; nucleic acid binding [goid 3676] [evidence IEA]; nucleotide binding [go
protein binding [goid 5515] [pmid 15685448] [evidence IPI]

DNA binding [goid 3677] [pmid 9119399] [evidence NAS]
receptor activity [goid 4872] [evidence IEA]; protein binding [goid 5515] [evidence IEA]

DNA binding [goid 3677] [evidence IEA]

transcription factor activity [goid 3700] [pmid 10848577] [evidence TAS]; hematopoietin/interferon-class (D20

GPI anchor binding [goid 48503] [evidence IEA]; enzyme binding [goid 19899] [pmid 14688365] [evidence IPI];

receptor activity [goid 4872] [evidence IEA]; interleukin-4 receptor activity [goid 4913] [pmid 2307934] [evide
zinc ion binding [goid 8270] [evidence IEA]; metal ion binding [goid 46872] [evidence IEA]; DNA binding [goid 3
kinase activity [goid 16301] [pmid 11839747] [evidence TAS]; ATP binding [goid 5524] [evidence IEA]; nucleoti
cytokine activity [goid 5125] [evidence IEA]
zinc ion binding [goid 8270] [evidence IEA]; alkaline phosphatase activity [goid 4035] [pmid 3042787] [evidenc
]; cell cycle [goid 7049] [evidence IEA]; cell cycle arrest [goid 7050] [pmid 10747892] [evidence TAS]
molecular function unknown [goid 5554] [evidence ND]
binding [goid 5488] [evidence IEA]; signal transducer activity [goid 4871] [pmid 8844170] [evidence TAS]
structural constituent of ribosome [goid 3735] [pmid 15883184] [evidence IDA]

transferase activity [goid 16740] [evidence IEA]; nucleotide binding [goid 166] [evidence IEA]; ATP binding [goi
ATP binding [goid 5524] [evidence IEA]; transferase activity [goid 16740] [evidence IEA]; protein serine/threon

zinc ion binding [goid 8270] [evidence IEA]; metal ion binding [goid 46872] [evidence IEA]; neprilysin activity [
pyridoxal phosphate binding [goid 30170] [evidence IEA]; nucleotide binding [goid 166] [evidence IEA]; phosph

] [evidence IEA]; development [goid 7275] [evidence IEA]; frizzled signaling pathway [goid 7222] [evidence IEA
phenylalanine-tRNA ligase activity [goid 4826] [pmid 10049785] [evidence TAS]; ligase activity [goid 16874] [e

RNA binding [goid 3723] [pmid 8634703] [evidence TAS]; nucleic acid binding [goid 3676] [evidence IEA]; nucle

binding [goid 5488] [evidence IEA]

calcium-dependent phospholipid binding [goid 5544] [evidence IEA]; ubiquitin thiolesterase activity [goid 422

pattern recognition receptor activity [goid 8329] [pmid 11567029] [evidence IDA]; MHC protein binding [goid
transcription corepressor activity [goid 3714] [pmid 9651585] [evidence TAS]; protein binding [goid 5515] [pm
binding [goid 5488] [evidence IEA]
actin binding [goid 3779] [evidence NAS]; protein binding [goid 5515] [pmid 16169070] [evidence IPI]; phosph
transcription corepressor activity [goid 3714] [pmid 8425218] [evidence TAS]; transcription factor activity [goi
oxidoreductase activity [goid 16491] [evidence IEA]; estradiol 17-beta-dehydrogenase activity [goid 4303] [evi
RNA binding [goid 3723] [evidence IEA]; nucleotide binding [goid 166] [evidence IEA]

calmodulin binding [goid 5516] [evidence IEA]; ATP binding [goid 5524] [evidence IEA]; transferase activity [go

transferase activity [goid 16740] [evidence IEA]; eukaryotic translation initiation factor 2alpha kinase activity [
cytoskeletal adaptor activity [goid 8093] [pmid 11274158] [evidence NAS]; protein binding [goid 5515] [evider

exonuclease activity [goid 4527] [evidence IEA]; 3'-5'-exoribonuclease activity [goid 175] [pmid 11110791] [ev

calmodulin binding [goid 5516] [pmid 10747990] [evidence TAS]; signal transducer activity [goid 4871] [evider

enzyme activator activity [goid 8047] [pmid 8798479] [evidence TAS]; protein kinase inhibitor activity [goid 48

chemokine activity [goid 8009] [pmid 11415443] [evidence IDA]

zinc ion binding [goid 8270] [evidence IEA]; metal ion binding [goid 46872] [evidence IEA]; electron carrier acti
catalytic activity [goid 3824] [evidence IEA]; isomerase activity [goid 16853] [evidence IEA]
protein homodimerization activity [goid 42803] [pmid 10913138] [evidence IDA]; S100 beta binding [goid 481
zinc ion binding [goid 8270] [evidence IEA]; nucleic acid binding [goid 3676] [evidence IEA]

RNA polymerase II transcription factor activity [goid 3702] [pmid 9919311] [evidence TAS]; DNA binding [goid
signal transducer activity [goid 4871] [pmid 3313057] [evidence TAS]; calcium ion binding [goid 5509] [pmid 2

receptor activity [goid 4872] [evidence IEA]
retinal binding [goid 16918] [evidence IEA]; retinol binding [goid 19841] [evidence IEA]; lipid binding [goid 828

protein homodimerization activity [goid 42803] [evidence ISS]; coreceptor activity [goid 15026] [pmid 965700
protein binding [goid 5515] [evidence IEA]; zinc ion binding [goid 8270] [evidence IEA]; metal ion binding [goid
lyase activity [goid 16829] [evidence IEA]; ammonia ligase activity [goid 16211] [evidence IEA]; histidine ammo
hydrolase activity [goid 16787] [evidence IEA]; calcium ion binding [goid 5509] [evidence IEA]; arylsulfatase ac
ubiquitin thiolesterase activity [goid 4221] [evidence IEA]; cysteine-type endopeptidase activity [goid 4197] [e

protein binding [goid 5515] [pmid 16189514] [evidence IPI]
DNA binding [goid 3677] [evidence IEA]; ATP binding [goid 5524] [evidence IEA]; ligand-dependent nuclear rec

nucleotide binding [goid 166] [pmid 8702478] [evidence TAS]; leukotriene receptor activity [goid 4974] [pmid
kinase activity [goid 16301] [evidence IEA]; transferase activity [goid 16740] [evidence IEA]; nucleotide binding
structural constituent of ribosome [goid 3735] [evidence IEA]
transferase activity [goid 16740] [evidence IEA]; protein phosphatase inhibitor activity [goid 4864] [pmid 1239

coreceptor activity [goid 15026] [pmid 10359581] [evidence TAS]
MHC class I receptor activity [goid 30106] [evidence IEA]

oxygen transporter activity [goid 5344] [evidence IEA]; heme binding [goid 20037] [evidence IEA]; ligase activi
nucleotide binding [goid 166] [evidence IEA]
zinc ion binding [goid 8270] [evidence IEA]; metal ion binding [goid 46872] [evidence IEA]; transcription factor
receptor activity [goid 4872] [evidence IEA]; IgG binding [goid 19864] [evidence IEA]; GPI anchor binding [goid
sequence-specific DNA binding [goid 43565] [evidence IEA]; protein dimerization activity [goid 46983] [eviden
receptor activity [goid 4872] [evidence IEA]; interleukin-17 receptor activity [goid 30368] [pmid 9367539] [evi
transmembrane receptor protein tyrosine phosphatase activity [goid 5001] [pmid 8798755] [evidence TAS]; re
zinc ion binding [goid 8270] [evidence IEA]; metal ion binding [goid 46872] [evidence IEA]; peptidase activity [g

actin binding [goid 3779] [evidence IEA]
calcium ion binding [goid 5509] [pmid 2149559] [evidence TAS]
folic acid binding [goid 5542] [pmid 8110752] [evidence TAS]; receptor activity [goid 4872] [evidence IEA]
molecular function unknown [goid 5554] [evidence ND]
cadherin binding [goid 45296] [evidence ISS]; protein kinase activity [goid 4672] [pmid 7592934] [evidence TA[illegible]
transferase activity [goid 16740] [evidence IEA]; transmembrane receptor activity [goid 4888] [pmid 1108151[illegible]

structural constituent of ribosome [goid 3735] [evidence IEA]
binding [goid 5488] [evidence IEA]

hydrolase activity [goid 16787] [evidence IEA]; magnesium ion binding [goid 287] [evidence IEA]; manganese i[illegible]
transcriptional activator activity [goid 16563] [pmid 9689109] [evidence TAS]; DNA binding [goid 3677] [evide[illegible]
leukotriene receptor activity [goid 4974] [pmid 10391245] [evidence TAS]; receptor activity [goid 4872] [evide[illegible]

iron ion binding [goid 5506] [evidence IEA]; monooxygenase activity [goid 4497] [pmid 8486631] [evidence TA[illegible]
protein phosphatase inhibitor activity [goid 4864] [evidence IEA]; actin binding [goid 3779] [evidence IEA]
protein-L-isoaspartate (D-aspartate) O-methyltransferase activity [goid 4719] [evidence IEA]

structural constituent of ribosome [goid 3735] [pmid 15883184] [evidence IDA]

protein binding [goid 5515] [pmid 15194699] [evidence IPI]

protein binding [goid 5515] [pmid 11544179] [evidence IPI]; RNA binding [goid 3723] [evidence IEA]

binding [goid 5488] [evidence IEA]
signal transducer activity [goid 4871] [pmid 8662509] [evidence NAS]
Rab GTPase binding [goid 17137] [pmid 10859313] [evidence IPI]; Rab GTPase activator activity [goid 5097] [p[illegible]
binding [goid 5488] [evidence IEA]
receptor activity [goid 4872] [evidence IEA]; IgA binding [goid 19862] [evidence IEA]; receptor signaling protei[illegible]
receptor activity [goid 4872] [evidence IEA]
ATPase activity [goid 16887] [evidence ISS]; microtubule binding [goid 8017] [evidence ISS]; ATP binding [goid [illegible]
zinc ion binding [goid 8270] [evidence IEA]; metal ion binding [goid 46872] [evidence IEA]; protein binding [goi[illegible]

zinc ion binding [goid 8270] [pmid 10608892] [evidence TAS]; metal ion binding [goid 46872] [evidence IEA]; p[illegible]
transcriptional activator activity [goid 16563] [pmid 10976108] [evidence TAS]; transcriptional repressor activi[illegible]
RNA binding [goid 3723] [pmid 9256445] [evidence TAS]; microtubule binding [goid 8017] [pmid 15851029] [e[illegible]
protein binding [goid 5515] [pmid 12606567] [evidence IDA]

zinc ion binding [goid 8270] [evidence IEA]; metal ion binding [goid 46872] [evidence IEA]; double-stranded R[illegible]
protein dimerization activity [goid 46983] [evidence IEA]; sequence-specific DNA binding [goid 43565] [eviden[illegible]
glucose transporter activity [goid 5355] [evidence IEA]; transporter activity [goid 5215] [evidence IEA]; sugar p[illegible]
transporter activity [goid 5215] [evidence IEA]

zinc ion binding [goid 8270] [pmid 11522286] [evidence TAS]; calcium ion binding [goid 5509] [pmid 7626002] [illegible]
kinase activity [goid 16301] [evidence IEA]

receptor activity [goid 4872] [pmid 1358805] [evidence TAS]; hematopoietin/interferon-class (D200-domain) c

RNA binding [goid 3723] [evidence IEA]; hydrolase activity [goid 16787] [evidence IEA]; nucleotide binding [goi

zinc ion binding [goid 8270] [evidence IEA]; metal ion binding [goid 46872] [evidence IEA]; protein binding [goi
kinase activity [goid 16301] [evidence IEA]
zinc ion binding [goid 8270] [evidence IEA]; metal ion binding [goid 46872] [evidence IEA]; transcription factor

transferase activity [goid 16740] [evidence IEA]; nucleotide binding [goid 166] [evidence IEA]; eukaryotic trans
structural constituent of ribosome [goid 3735] [evidence NAS]

tumor necrosis factor receptor binding [goid 5164] [evidence IEA]
protein kinase binding [goid 19901] [pmid 11034606] [evidence IPI]; signal transducer activity [goid 4871] [pm
signal transducer activity [goid 4871] [evidence IEA]
tapasin binding [goid 46980] [pmid 12047747] [evidence IPI]; peptide antigen binding [goid 42605] [pmid 111

transcription factor activity [goid 3700] [evidence IEA]
threonine endopeptidase activity [goid 4298] [evidence IEA]; RNA binding [goid 3723] [pmid 7681138] [eviden
ion channel inhibitor activity [goid 8200] [pmid 9653196] [evidence TAS]; receptor binding [goid 5102] [pmid 9

transcription factor activity [goid 3700] [evidence IEA]; protein heterodimerization activity [goid 46982] [pmid

receptor activity [goid 4872] [evidence IEA]; signal transducer activity [goid 4871] [pmid 9495343] [evidence T
metallopeptidase activity [goid 8237] [pmid 2564851] [evidence TAS]; aminopeptidase activity [goid 4177] [pr

transcriptional repressor activity [goid 16564] [pmid 10523670] [evidence TAS]; transcription factor binding [g
zinc ion binding [goid 8270] [evidence IEA]; metal ion binding [goid 46872] [evidence IEA]; nucleic acid binding
5-oxo-6E,8Z,11Z,14Z-icosatetraenoic acid binding [goid 50646] [pmid 12065583] [evidence NAS]; receptor acti
transferase activity [goid 16740] [evidence IEA]; nucleotide binding [goid 166] [evidence IEA]; ATP binding [goi
nucleotide binding [goid 166] [evidence IEA]; GTP binding [goid 5525] [evidence IEA]; protein binding [goid 55

mRNA binding [goid 3729] [evidence NAS]; nucleic acid binding [goid 3676] [evidence IEA]; nucleotide binding
structural constituent of cytoskeleton [goid 5200] [pmid 9230079] [evidence TAS]
DNA binding [goid 3677] [evidence IEA]
hydrolase activity [goid 16787] [evidence IEA]; magnesium ion binding [goid 287] [pmid 11795870] [evidence I
structural constituent of ribosome [goid 3735] [evidence IEA]

hydrolase activity [goid 16787] [evidence IEA]; NAD+ nucleosidase activity [goid 3953] [evidence IEA]; GPI ancl

chromatin binding [goid 3682] [pmid 9169582] [evidence TAS]; protein binding [goid 5515] [pmid 11242053] [

sugar binding [goid 5529] [pmid 10528209] [evidence TAS]

ATP binding [goid 5524] [evidence IEA]; transferase activity [goid 16740] [evidence IEA]; protein serine/threon
transferase activity [goid 16740] [evidence IEA]; Gram-positive bacterial binding [goid 51076] [pmid 11521063
binding [goid 5488] [evidence IEA]
ubiquitin protein ligase binding [goid 31625] [pmid 9859996] [evidence IPI]; NF-kappaB binding [goid 51059] [
transferase activity [goid 16740] [evidence IEA]
transporter activity [goid 5215] [evidence IEA]
ligase activity [goid 16874] [evidence IEA]; ubiquitin-protein ligase activity [goid 4842] [evidence IEA]
actin binding [goid 3779] [evidence IEA]

hydrolase activity [goid 16787] [evidence IEA]
binding [goid 5488] [evidence IEA]; ATP binding [goid 5524] [evidence IEA]; transferase activity [goid 16740] [e
tapasin binding [goid 46980] [pmid 12047747] [evidence IPI]; peptide antigen binding [goid 42605] [pmid 111

interleukin-1 receptor binding [goid 5149] [evidence IEA]; signal transducer activity [goid 4871] [pmid 899989
clathrin binding [goid 30276] [pmid 10436022] [evidence IDA]; phospholipid binding [goid 5543] [evidence IEA
ATP binding [goid 5524] [evidence IEA]; chromatin binding [goid 3682] [evidence IEA]; DNA binding [goid 3677
calcium ion binding [goid 5509] [evidence IEA]; protein binding [goid 5515] [evidence IEA]

protein phosphatase type 1 activity [goid 163] [pmid 9414128] [evidence TAS]

receptor activity [goid 4872] [evidence IEA]; rhodopsin-like receptor activity [goid 1584] [evidence IEA]; interle

zinc ion binding [goid 8270] [evidence IEA]; nucleic acid binding [goid 3676] [evidence IEA]
GTP binding [goid 5525] [evidence IEA]; structural molecule activity [goid 5198] [evidence IEA]; GTPase activity
zinc ion binding [goid 8270] [evidence IEA]; metal ion binding [goid 46872] [evidence IEA]; protein binding [goi

receptor activity [goid 4872] [evidence IEA]; G-protein coupled receptor activity, unknown ligand [goid 16526]

tropomyosin binding [goid 5523] [evidence IEA]
GTPase activity [goid 3924] [pmid 14645372] [evidence IDA]; GTP binding [goid 5525] [pmid 14645372] [evide
growth factor activity [goid 8083] [evidence IEA]; insulin-like growth factor binding [goid 5520] [evidence IEA]

calmodulin binding [goid 5516] [evidence IEA]; structural constituent of cytoskeleton [goid 5200] [pmid 88938
protein binding [goid 5515] [pmid 12812986] [evidence IPI]
enzyme activator activity [goid 8047] [pmid 9079638] [evidence TAS]; growth factor activity [goid 8083] [evide

DNA binding [goid 3677] [evidence IEA]

chromatin binding [goid 3682] [pmid 8663349] [evidence TAS]; protein binding [goid 5515] [pmid 15502821] [

transferase activity [goid 16740] [evidence IEA]; ubiquitin-protein ligase activity [goid 4842] [evidence ISS]; pro
ATP binding [goid 5524] [evidence IEA]; hydrolase activity [goid 16787] [evidence IEA]; nucleotide binding [goi
transcription regulator activity [goid 30528] [evidence IEA]; transcription factor activity [goid 3700] [pmid 109
cell adhesion molecule binding [goid 50839] [evidence ISS]

oxidoreductase activity [goid 16491] [evidence IEA]; cytochrome-c oxidase activity [goid 4129] [pmid 8382530
sequence-specific DNA binding [goid 43565] [evidence IEA]; transcription factor activity [goid 3700] [pmid 234

transferase activity [goid 16740] [evidence IEA]; protein-tyrosine kinase activity [goid 4713] [pmid 7970727] [e
zinc ion binding [goid 8270] [pmid 2251898] [evidence TAS]; gelatinase B activity [goid 4229] [pmid 16192646]
acyltransferase activity [goid 8415] [evidence IEA]; transferase activity [goid 16740] [evidence IEA]; calcium ion
electron carrier activity [goid 9055] [pmid 1692159] [evidence TAS]; binding [goid 5488] [evidence IEA]; protei

transferase activity [goid 16740] [evidence IEA]; small GTPase regulator activity [goid 5083] [evidence IEA]; nu
enzyme activator activity [goid 8047] [pmid 2300173] [evidence TAS]; binding [goid 5488] [pmid 2300173] [ev
threonine endopeptidase activity [goid 4298] [evidence IEA]
DNA-dependent ATPase activity [goid 8094] [evidence IEA]; nucleoside-triphosphatase activity [goid 17111] [e
transferase activity [goid 16740] [evidence IEA]; nucleotide binding [goid 166] [evidence IEA]; ATP binding [goi
cytokine activity [goid 5125] [evidence IEA]

ligase activity [goid 16874] [evidence IEA]; ubiquitin-protein ligase activity [goid 4842] [pmid 12853982] [evide

RNA binding [goid 3723] [pmid 9887331] [evidence TAS]; nucleotide binding [goid 166] [evidence IEA]

magnesium ion binding [goid 287] [pmid 15632002] [evidence TAS]; calcium ion binding [goid 5509] [pmid 163

A]
protein binding [goid 5515] [evidence IEA]; translation elongation factor activity [goid 3746] [evidence IEA]

DNA binding [goid 3677] [evidence IEA]; zinc ion binding [goid 8270] [evidence IEA]; metal ion binding [goid 46

transferase activity [goid 16740] [evidence IEA]; protein binding [goid 5515] [pmid 16169070] [evidence IPI]

pyridoxal phosphate binding [goid 30170] [pmid 15650327] [evidence IDA]; transferase activity [goid 16740] [e

hydrolase activity [goid 16787] [evidence IEA]; nucleotide binding [goid 166] [evidence IEA]; helicase activity [g

calcium-dependent phospholipid binding [goid 5544] [evidence IEA]; calcium ion binding [goid 5509] [evidence
protein-arginine deiminase activity [goid 4668] [pmid 10488123] [evidence TAS]; hydrolase activity [goid 1678
calcium ion binding [goid 5509] [evidence IEA]
zinc ion binding [goid 8270] [evidence IEA]; nucleic acid binding [goid 3676] [evidence IEA]
transcriptional repressor activity [goid 16564] [pmid 12724404] [evidence IDA]; transcription factor activity [g

transporter activity [goid 5215] [evidence IEA]

sequence-specific DNA binding [goid 43565] [evidence IEA]
1-phosphatidylinositol-4-phosphate 5-kinase activity [goid 16308] [evidence IEA]; kinase activity [goid 16301]
GTP binding [goid 5525] [evidence IEA]; GTPase activity [goid 3924] [pmid 7811277] [evidence TAS]; nucleotid
transcription factor activity [goid 3700] [pmid 11782478] [evidence IDA]; ligand-dependent nuclear receptor a
zinc ion binding [goid 8270] [evidence IEA]; metal ion binding [goid 46872] [evidence IEA]
protein binding [goid 5515] [evidence IEA]
zinc ion binding [goid 8270] [evidence IEA]; metal ion binding [goid 46872] [evidence IEA]; hematopoietin/inte
glutamate-ammonia ligase activity [goid 4356] [evidence IEA]; actin binding [goid 3779] [evidence IEA]; protei

receptor activity [goid 4872] [evidence IEA]; interleukin-9 receptor activity [goid 4919] [pmid 1376929] [evide
unfolded protein binding [goid 51082] [evidence IEA]; heat shock protein binding [goid 31072] [evidence IEA]
DNA binding [goid 3677] [pmid 15888725] [evidence IDA]; zinc ion binding [goid 8270] [evidence IEA]; metal ic
DNA binding [goid 3677] [pmid 10449920] [evidence NAS]

Synonyms

Z32; mgU2-19/30

DDIT2; CR6; GADD45gamma; GRP17

MOAT-C; pABC11; ABC33; MRP5; SMRP; EST277145; DKFZp686C1782; MOATC
: IEA]; signal transducer activity [goid 4871] [evidence IEA]
bA810I22.1; RP11-810I22.1; FLJ11240; C6orf209

GLIPR2; GAPR-1
pp90RSK2; MAPKAPK1B; CLS; RSK2; RSK; S6K-alpha3; p90-RSK2; ISPK-1; MRX19; HU-3
QRX; MGC15631; ARMD6; CORD11

DKFZP666B131; PBEF; 1110035O14Rik; NAMPT; MGC117256

UPA; URK; u-PA; ATF
PR72; PPP2R3; PR130
FLJ13456; ZYG11
SAR2; TSPAN27; GR15; ST6; KAI1; 4F9; C33; IA4; R2
RPB11; MGC71910; hRPB14; RPB11m; RPB11A; POLR2J1
FLJ46426
DLX2; DYLX2; DYX2
RLPK; MSPK1; MGC1911; MSK1
EGFL5

H2a-615; H2A; H2AFO; HIST2H2AA; H2A/q; H2A/O; H2A.2
TRP14; MGC14353

MPS-1; MPS1
C1orf139; FLJ23091; WLS; MGC131760; MRP; MGC14878

CSF2R; CSF2RAY; CSF2RX; GM-CSF-R-alpha; CSF2RAX; CD116; MGC3848; CDw116; GMR; MGC4838; CSF2RY; G

RAMP; SCLL; MYM; FIM; ZNF198
G5pr; FLJ20644; G4-1; C14orf10
GPCR1; GPCR150
CSTP1
MGC119678; MGC119680
NECAB2
CACH6; BII; CACNL1A6; Cav2.3
E3; PHE3; DLDH; GCSL; LAD
HRG4

CGI-119; ZPRO; S1R
SWIP1; WSB-1
FLJ33845; KIAA1720
MGC131982; KIAA0529; UNPH4; MGC149838; MGC74854

FTS; FT1
DKFZP667O116; RNF109; FLJ35608
C10orf7; D123
NKX3A; BAPX2; NKX3; NKX3.1
FLJ13055; DKFZp761E1121; PRG-4
MGC126411; FLJ90554; KIAA1247; HSULF-2; DKFZp313E091
PC4; TIS7

bA479K20.1; KIAA0625; Lmnt2; MGC21884; NTNG1; KIAA1857

FLJ13639
HCP1; PDE7
PYHIN3
E2FBP1; BRIGHT; DRIL3; DRIL1
MGC131732; FLJ33216

RP11-475E11.6; KIAA0761; MCLC
MGC104792; TROB; APRO6; TROB1; PIG49; TOB; MGC34446
FLJ22835; IFI75; VODI; IFI41
MGC111419; LMN; LMNB; LMN2
PAR2; GPR11

NRK1; bA235O14.2; RP11-235O14.2; FLJ20559
DHRS8; PAN1B; RETSDR2

SIRP-BETA-1; CD172b; DKFZp686A05192
CD119; IFNGR; FLJ45734

DKFZp547L163; FLJ20739
KIAA0158; hNedd5; DIFF6; NEDD5
CLEC6; MGC40078; CLECSF8; CLEC-6; MPCL; MCL

PHS-2; COX-2; hCox-2; COX2; PGHS-2; PGG/HS

MGC126681; NEP; CD10; MGC126707; DKFZp686O16152; CALLA
BUP1
TAFII68; TAF2N; RBP56; hTAFII68; Npl3
BC200a; BC200
DKFZp781P1719; C3G; GRF2
FOAP-4; GPI-80
MGC87867; PnSP-2; HIN1; HIN-1; UGRP2; LU105

MGC20741
DKFZp686B1993; TRX; MGC61975
PACPL1; ACPL1; LPAP

MGC3606; PKR2; PRKAR2
cN-II; NT5B; PNT5; GMP
E2(17)KB3; MGC43926; UBCH5C; MGC5416; UBC4/5
G17; PBX2MHC; HOX12
DKFZp781D2217; PFK-2/FBPase-2; MGC138308; MGC138310
FLJ32028
PROSIT240; FLJ21627; MED13L; KIAA1025; TRAP240L; DKFZp781D0112
AC; FLJ22079; FLJ21558; PHP32; ASAH; PHP
FACL2; LACS; FACL1; ACS1; LACS2; LACS1
MGC22484; PSG14; PSG13
KIAA1145
DKFZp434I1020; FLJ45937; MGC119618
TSP; THBS; TSP1
OATP-M1; SLC21A20; OATPX; OATP4C1; OATP-H; PRO2176
MEA5; FLJ23355; NCOAT; FLJ11229; KIAA0679
HB24
MGC126411; FLJ90554; KIAA1247; HSULF-2; DKFZp313E091
FLJ11153
IFI-6-16; 6-16; IFI616; G1P3; FAM14C

LL37; FALL39; CAP18; HSD26; FALL-39
H2B/j; H2BFJ
CD266; TWEAKR; FN14

FLJ46828; FAAP24; MGC32020
DKFZp686F01145; DKFZp686G24132
ISGF-3; STAT91; DKFZp686B04100

CD87; UPAR; URKR

IL4RA; CD124
GIOT-4; KOX6
de binding [goid 166] [evidence IEA]
CKLFH; CKLFSF1; CKLFH1; MGC71870
PLAP; ALP
GADD45; DDIT1
ICAp69; ICA69
CLH22; CLTD; CHC22; CLTCL

FLJ36101; SFRSK2
DKFZp686A20267

NEP; MGC126681; CD10; MGC126707; DKFZp686O16152; CALLA
ıorylase activity [goid 4645] [evidence IEA]; glycogen phosphorylase activity [goid 8184] [pmid 9529348] [evide
ASP; FLJ25776
BCE-1; ESG4; ESG; GRG4; E(spl)
PheHB; FRSB

RNPL; IS1-RNPL

RNM561; IFI-56; IFI56; GARG-16; IFNAI1; G10P1

DFFRY; SP3; AZF; AZFA

BGR; DECTIN1; CLECSF12
id 12670868] [evidence IPI]
UTY1; DKFZp686L12190
D3S1319E; PFL
MGC104659; MAD; MAD1
KAR
Ht006; SF3B14a; SAP14; CGI-110; HSPC175; P14

MGC125447; MGC125448
CKLiK; CaMKID; CaM-K1

PRKR; PKR; MGC126524; EIF2AK1
MGC14978
FLJ90675; GPP34; GOPP1
RP11-3J10.8; hRrp40p; MGC15120; p10; Rrp40p; CGI-102; RRP40; MGC723; bA3J10.7

G0S8

MGC126867; GMF-GAMMA

UCK-1; CKLF1; CKLF4; CKLF2; CKLF3; C32; HSPC224
FLJ20525

DHQV; DIA6; NMOR2; QR2
FLJ14767; MAWDBP; FLJ35507; MAWBP
PRA; CABP; 2A9; 5B10; CACY
DKFZp762I137; MGC156120

MGC45871
EZNF; TCF17; KID-1; HKL1; KID1
CFAG; MAC387; 60B8AG; MIF; MRP14; CGLB; CAGB; NIF; LIAG; P14; L1AG

dJ331H24.1; FLJ21079; MGC102783
CRBP4; MGC70641; CRBPIV

PVRR2; HVEB; PRR2; CD112
DKFZp686M11215
HSTD; histidase; HIS
KIAA1001
MGC2621; UBPO; KIAA0190

RP11-217H1.1; bA217H1.1; PRO0756; MGC64926; FLJ14726
Exo84p; SEC84; EXO84
TRIP2; PPARGBP; RB18A; MGC71488; MED1; CRSP200; TRAP220; CRSP1; PBP; DRIP205; DRIP230

LTBR1; P2Y7; CMKRL1; P2RY7; BLTR; BLT1; GPR16; LTB4R1
HKII; HXK2; DKFZp686M1669

KIAA1079; cprk; AATYK2; KPI-2; BREK; KPI2; LMR2
MGC133242; ERIC1; MGC117382
MD-2

FLJ20696; FLJ10652
FLJ16050
KIAA1093
MGC138429; 9130423L19Rik; KOX18; ZNF139; PHZ-37; ZNF36
CD16b; FCG3; CD16; FCGR3
CRE-BPA
hIL-17R; IL-17RA; MGC10262; CDw217; IL17R
PTPRP; ICAAR; IAR; IA-2beta
G1RZFP; MGC138647; GOLIATH; MGC99542; MGC117241; GP

DA2B; AMCD2B; FSSV
CFAG; 60B8AG; MRP8; CGLA; L1Ag; MIF; MA387; CAGA; CP-10; NIF; P8
FR-gamma; gamma-hFR; FR-G
NY-BR-1.1
p35nck5a; CDK5P35; MGC33831; NCK5A; p23; p35; p25; CDK5R
KIAA0012; DKFZp547I0610; TIL; CD281; MGC126312; MGC104956; rsc786; DKFZp564I0682; MGC126311

AGP2; AGP-B; AGP-B'
KIAA0912

PPC2BETAX; MGC21657; PP2CB; PP2C-beta-X; PP2CBETA
GBF; ZF9; ST12; CPBP; BCD1; PAC1; DKFZp686N0199; COPEB
CYSLTR; MGC46139; HMTMF81; CYSLT1R; CYSLT1; HG55

FLJ23476; IRIP; FLJ26165; RP11-109P14.4; SUA5; DRIP3
CPF3; CYP4F; LTB4H
RPEL; MGC126577; KIAA1733; MGC126575; RPEL1; dJ257A7.2
FLJ10883

FLJ10460; C15orf25; HsT17025

MGC20672; GM130

KIAA1408; MGC138835; HUPF2; DKFZP434D222; RENT2; MGC138834
7SK
UTY1; DKFZp686L12190
MGC133042; MYPT1; MBS
RAB3GAP; RAB3GAP130; P130; DKFZp434A012; KIAA0066; WARBM1
FLJ14051; FLJ10008
CD89
MGC33613; PIGR3
KIAA0591; HMSNII; CMT2; KIAA1448; CMT2A; MGC134844; KLP; FLJ23699; CMT2A1
MGC142212; MGC142210; KIAA0244

MDMX; DKFZp781B1423; MRP1; MGC132766
Epl1; DKFZp781P2312
MIG14; MGC117333; dJ800J21.1; FLJ30608; MGC126076; Mnrp41; MRNP41; dJ481F12.3; MGC126077
CASP; CYTIP; HE; CYBR; B3-1

Zfp346; JAZ; DKFZp547M223
JUNDM2
MGC118833; MGC118830; GLUT10; SLC2A11-a; SLC2A11-c; GLUT11
MGC132523; SGLT4; MGC132517

CAAF1; CAGC; ENRAGE; p6; CGRP; MRP6
RP3-503M14.1; MGC21854; CAPZIP; MGC126611; MK2S4; MGC126585

CSF2R; CSF2RAY; CSF2RX; GM-CSF-R-alpha; CSF2RAX; CD116; MGC3848; CDw116; GMR; MGC4838; CSF2RY; G
SDHAL1

MGC2821; FLJ21167

KIAA0348; INPP5H; MGC44422
SYN10; hsyn10
RNF189; RNF34L
COQ8; FLJ12229
Z147; ZNF147; EFP; RNF147
MGC70444; MGC21968
RP3-422G23.1; KIAA1244; C6ORF34B; PP23; SOUL; C6orf34
GCN2; KIAA1338
MGC117346; rpL17; MGC111167; MGC72008

ZTNF4; TALL-1; THANK; TNFSF20; CD257; delta BAFF; TALL1; BLYS; BAFF
GIG3; MORT1; MGC8528
RGS13
RING11; PSF2; APT2; ABC18; ABCB3; D6S217E
MGC15827

PRG1; PPG; PRG; FLJ12930; MGC9289
TBR2
MGC22853; MGC14751; PROS30; HC2; MGC1667; MGC21459; MGC14542; MGC23915; NU; MGC14575
MGC4319; MGC78563; MGC8394
FLJ36756; FLJ13081
HIF-1alpha; MOP1; PASD8; HIF1-ALPHA
KAT3
EVDB; D17S376
AS]; insulin receptor binding [goid 5158] [evidence IEA]
APN; gp150; CD13; LAP1; PEPN

HD4; HDAC-A; KIAA0288; HA6116; HDACA
FLJ23765; FLJ31827
GPR170; GPCR; TG1019
MEKK2; MEKK2B
RAP1; KREV-1; KREV1; SMGP21

S164; MGC105088; RNPC7; MGC117168
ARC16; dJ127C7.3; p16-Arc

MGC27337; PN-I; PSN1; UMPH1; MGC87828; UMPH; MGC87109; cN-III; P5'N-1
PP9932
FLJ11795; MGC126014; MGC126013

CD157

M31; HP1Hs-beta; CBX; HP1-BETA; MOD1

FLJ40915
CLECSF9; MINCLE
HSPC164; SHUJUN-3; CDAO5
ine kinase activity [goid 4674] [pmid 9154127] [evidence TAS]; protein-tyrosine kinase activity [goid 4713] [evi
CD282; TIL4
CG7943; MGC14836
IKBA; NFKBI; MAD-3
NLRR-3; NLRR3; FLJ11129
TAT1; FLJ32714
DKFZp686M17164; NEDL2
TB4Y; MGC26307

RIPK7; ROCO2; PARK8; AURA17
APT2; RING11; PSF2; ABC18; ABCB3; D6S217E

MGC12320; IL-18; IL1F4; IGIF; IL-1g
LAP; CALM; CLTH
FLJ20361; KIAA1416; FLJ20357
CDw325; CDHN; NCAD

PPP1R6; DKFZp781L2441
ORP8; MGC126578; DKFZp686A11164; OSBP10; MGC133203

CD182; CMKAR2; IL8RA; IL8R2; CXCR2; CDw128b
FLJ21255; MGC5540; PP14212
BTNLR
MGC132756; MA3; MGC132758; MA5
TUBA1; H2-ALPHA; FLJ30169
BBAP

FLJ14629; FLJ34384; ORP9; FLJ14801; FLJ32055; MGC15035; FLJ12492
GPCR4; EX33

DKFZp313F0135
MGC126249; TGE; MGC126250
IGFBP9; NOVH; CCN3

ADDL
MGC78416; FLJ13386
GROa; GRO1; MGSA alpha; NAP-3; MGSA-a; MGSA; SCYB1
FLJ10583; WINS1

MGC74712; PAIP; LEDGF; p75; PSIP2; p52; DFS70

HP1-GAMMA; HP1Hs-gamma; HECH
DKFZP434B044; MGC74865; CRISP11; LCRISP2

p63; CLIMP-63; MGC99554; ERGIC-63

Fbl13; FLJ38068; MGC21636
TAF172; KIAA0940; TAF(II)170; TAFII170; MOT1; MGC138406
TCFL4; MXD7; MAD7
SDFR1; GP55; DKFZp686L2477; np65; np55; GP65; SDR1; MGC102805

] [evidence TAS]
OCT1; OTF1

PSCTK2; PSCTK3; ETK
MMP-9; GELB; CLG4B
FLJ20481; LPCAT2; LysoPAFAT; DKFZp686H22112
p67phox; NOXA2; P67-PHOX
DKFZP434I143
KIAA0687; FLJ90111; FLJ20373; HGK; NIK; FLJ10410; FLH21957
FLAP
MGC70470; LMP2; RING12
MGC4816; C20orf154; MGC12866; MGC119523; dJ967N21.5; MGC119522
SFRSK1
MGC39436; CKLFSF2
FLJ22390; RP11-295M18.1
KIAA0161; MGC71786; p53RFP; bA528A10.3
MGC22805
BRUNOL3; ETR-3; NAPOR

MIG9
PSG11
C21orf107; FLJ43918; WDR9; N143
BPOZ; PP2259; EF1ABP; MGC20585
FLJ38932; PAT4
FLJ12859; ZNP-99; ZBP-99; FLJ14378
STRF2; FLJ12666; RP11-781D11.2
C15orf1; PP32; PHAPI; MGC150373; PHAP1; I1PP2A; LANP; MAPM; MGC119787

NPD009; GABAT; GABA-AT

KIAA1564; DKFZp686N17164; HELSNF1

ANX11; CAP50
PADI5; PDI4; PDI5; PAD
PP791; RP4-765A10.3; KIAA1837; PKD1-like
FLJ21082
RBP1; RBP-1; RBBP1

SMCT; MGC125354; AIT
FLJ22179; FLJ22155

Pip4k2B; PIP5KIIB
RAB1C; H-ray; RAY
ctivity [goid 4879] [pmid 10395741] [evidence IDA]; protein binding [goid 5515] [pmid 10395741] [evidence IP
C20orf162
MGC120306; FLJ25870; MGC120307; MGC120305
FLJ22835; IFI75; VODI; IFI41
WDR2; CLIPINB; IR10

CD129
C21orf78
MGC126276; LOT1; ZAC; MGC126275; ZAC1; DKFZp781P1017

WBSCR7

GB_ACC	group
XM_933516.1	lncRNA
XM_936105.1	lncRNA
XM_935575.1	lncRNA
XM_378454.3	lncRNA
XM_943677.1	lncRNA
XM_933693.2	lncRNA
XM_943048.1	lncRNA
XR_000601.1	lncRNA
XM_941963.1	lncRNA
XM_930545.1	lncRNA
XR_001261.1	lncRNA
XM_370932.4	lncRNA
NR_002569.1	lncRNA
AW450591	lncRNA
AK026751	lncRNA
XM_374029	lncRNA
AF452720	lncRNA
AK025793	lncRNA
CB852325	lncRNA
BI520375	lncRNA
XM_498474	lncRNA
BX103256	lncRNA
AK123264	lncRNA
NM_003329.1	mRNA
NM_001031.4	mRNA
NM_006705.2	mRNA
NM_001020.4	mRNA
NM_001023587.1	mRNA
NM_001017998.2	mRNA
NM_018368.2	mRNA
XM_936430.2	mRNA
NM_022343.2	mRNA
NM_004586.2	mRNA
NM_032753.2	mRNA
XM_372345.3	mRNA
NM_005746.2	mRNA
XM_929199.1	mRNA
NM_031925.1	mRNA
NM_002658.2	mRNA
NM_181897.1	mRNA
NM_024646.1	mRNA
NM_002231.3	mRNA
NM_006234.4	mRNA
NM_001004305.1	mRNA
NM_014809.2	mRNA
NM_004755.2	mRNA
NM_001080497.1	mRNA

XM_934218.2	mRNA
XR_017611.1	mRNA
NM_003516.2	mRNA
NM_032731.2	mRNA
XM_926814.1	mRNA
XM_936511.1	mRNA
NM_001030.3	mRNA
NM_001002292.1	mRNA
XM_934316.1	mRNA
NM_172246.1	mRNA
XM_941267.2	mRNA
NM_003453.2	mRNA
NM_017917.2	mRNA
NM_014373.1	mRNA
NM_018340.1	mRNA
NM_153615.1	mRNA
NM_019065.2	mRNA
NM_000721.2	mRNA
NM_000108.3	mRNA
NM_005148.2	mRNA
XM_497711.2	mRNA
NM_016056.2	mRNA
NM_015626.8	mRNA
NM_030645.1	mRNA
NM_006313.1	mRNA
XM_372521.1	mRNA
NM_022476.2	mRNA
NM_030961.1	mRNA
NM_006023.1	mRNA
NM_006167.2	mRNA
NM_022737.1	mRNA
NM_198596.1	mRNA
NM_001550.2	mRNA
XM_001128769.1	mRNA
NM_032536.1	mRNA
XM_937277.1	mRNA
NM_001031719.1	mRNA
NM_002604.1	mRNA
NM_002432.1	mRNA
NM_005224.2	mRNA
NM_001005852.1	mRNA
NM_004882.3	mRNA
NM_001048210.1	mRNA
NM_005749.2	mRNA
NM_004510.2	mRNA
NM_005573.2	mRNA
NM_005242.3	mRNA
XM_942097.1	mRNA

NM_017881.1	mRNA
NM_016245.2	mRNA
NM_001013725.1	mRNA
NM_006065.1	mRNA
NM_000416.1	mRNA
XM_934022.1	mRNA
NM_017949.1	mRNA
NM_001008491.1	mRNA
NM_080387.4	mRNA
XR_016005.1	mRNA
NM_000963.1	mRNA
NM_130781.2	mRNA
NM_007288.2	mRNA
NM_016327.2	mRNA
NM_139215.1	mRNA
NR_001568.1	mRNA
NM_198679.1	mRNA
NM_078488.1	mRNA
NM_052863.2	mRNA
XM_941155.2	mRNA
NM_018561.3	mRNA
NM_003329.2	mRNA
NM_016361.2	mRNA
XM_944417.1	mRNA
NM_004157.2	mRNA
NM_012229.2	mRNA
NM_181889.1	mRNA
NM_002586.4	mRNA
NM_006212.2	mRNA
NM_152680.1	mRNA
NM_015335.2	mRNA
NM_177924.2	mRNA
NM_001995.2	mRNA
NM_002785.2	mRNA
NM_020698.1	mRNA
NR_003260.1	mRNA
NM_003246.2	mRNA
NM_180991.4	mRNA
NM_012215.1	mRNA
NM_021958.2	mRNA
NM_018837.2	mRNA
NM_016090.2	mRNA
NM_002038.3	mRNA
XM_941155.2	mRNA
XM_944915.1	mRNA
NM_004345.3	mRNA
NM_003524.2	mRNA
NM_016639.1	mRNA

NM_152266.1	mRNA
NM_001010915.1	mRNA
NM_139266.1	mRNA
XM_001134072.1	mRNA
NM_001005376.1	mRNA
XM_933375.1	mRNA
NM_001008699.1	mRNA
NM_021030.2	mRNA
NM_005881.1	mRNA
NM_181301.1	mRNA
NM_001632.3	mRNA
NM_001924.2	mRNA
NM_022308.1	mRNA
NM_007098.2	mRNA
NM_001032.3	mRNA
XM_928387.1	mRNA
NM_182692.1	mRNA
NM_020666.2	mRNA
XR_019071.1	mRNA
NM_000902.3	mRNA
NM_002863.3	mRNA
NM_031916.2	mRNA
NM_007005.3	mRNA
NM_005687.2	mRNA
XM_938906.2	mRNA
NM_001017431.1	mRNA
XR_017837.1	mRNA
NM_001548.2	mRNA
XM_935827.1	mRNA
NM_004654.3	mRNA
XR_018616.1	mRNA
NM_197953.1	mRNA
NM_003864.3	mRNA
NM_182659.1	mRNA
NM_053024.3	mRNA
NM_002357.2	mRNA
NM_016142.1	mRNA
NM_016047.3	mRNA
XM_937928.1	mRNA
NM_001033658.1	mRNA
NM_020397.2	mRNA
XR_017611.1	mRNA
NM_002759.1	mRNA
NM_018688.4	mRNA
NM_022130.3	mRNA
NM_001002269.1	mRNA
NM_001012391.1	mRNA
XM_942939.1	mRNA

XM_927706.2	mRNA
NM_002923.1	mRNA
XM_934113.1	mRNA
XM_928866.1	mRNA
NM_004877.1	mRNA
XM_928047.1	mRNA
NM_016326.2	mRNA
NR_002949.1	mRNA
XM_927767.2	mRNA
NM_000904.1	mRNA
NM_022129.3	mRNA
NM_014624.3	mRNA
NM_152411.1	mRNA
XM_001126665.1	mRNA
NM_182705.2	mRNA
NM_005649.2	mRNA
NM_002965.3	mRNA
XM_498571.2	mRNA
NM_024576.3	mRNA
NM_052960.1	mRNA
NM_001039473.1	mRNA
NM_002856.2	mRNA
NM_144726.1	mRNA
NM_002108.2	mRNA
NM_014960.2	mRNA
NM_005153.2	mRNA
XM_942501.1	mRNA
NM_032121.3	mRNA
NM_175876.3	mRNA
NM_004774.2	mRNA
XM_001132265.1	mRNA
XM_001127825.1	mRNA
NM_181657.1	mRNA
NM_000189.4	mRNA
NM_001011538.1	mRNA
NM_014916.2	mRNA
NM_006342.1	mRNA
NM_015364.2	mRNA
NM_004048.2	mRNA
NM_018169.2	mRNA
NM_173497.1	mRNA
NM_001024843.1	mRNA
NM_003439.1	mRNA
NM_000570.2	mRNA
NM_001011666.1	mRNA
NM_014339.4	mRNA
NM_130843.1	mRNA
NM_018434.4	mRNA

NM_003282.2	mRNA
NM_002964.3	mRNA
NM_000804.2	mRNA
NM_001029862.1	mRNA
NM_003885.2	mRNA
NM_003263.3	mRNA
NM_020954.2	mRNA
NM_000987.3	mRNA
NM_000608.2	mRNA
NM_014985.1	mRNA
XM_933119.1	mRNA
NM_001033556.1	mRNA
NM_001300.4	mRNA
NM_006639.2	mRNA
XM_927280.1	mRNA
NM_024640.3	mRNA
NM_000896.1	mRNA
NM_030948.1	mRNA
NM_052937.1	mRNA
NM_173669.1	mRNA
NM_001032.3	mRNA
NM_018097.1	mRNA
XM_928041.1	mRNA
NM_004486.4	mRNA
XM_928180.1	mRNA
NM_015542.2	mRNA
NR_001445.1	mRNA
NM_007125.3	mRNA
NM_002480.1	mRNA
NM_012233.1	mRNA
NM_017970.2	mRNA
NM_133279.1	mRNA
NM_130771.2	mRNA
NM_183416.2	mRNA
NM_015153.1	mRNA
XM_935802.1	mRNA
NM_002393.2	mRNA
NM_025209.2	mRNA
NM_003610.3	mRNA
NM_004288.3	mRNA
XM_931879.1	mRNA
NM_012279.2	mRNA
NM_130469.2	mRNA
NM_001024938.1	mRNA
NM_001011547.1	mRNA
XM_001128558.1	mRNA
NM_005621.1	mRNA
NM_052862.2	mRNA

NM_172246.1	mRNA
NR_003264.1	mRNA
NM_207337.1	mRNA
NM_023003.1	mRNA
NM_024054.1	mRNA
XM_371820.5	mRNA
NM_003898.2	mRNA
NM_003765.1	mRNA
NM_001017368.1	mRNA
NM_024876.2	mRNA
NM_005082.4	mRNA
NM_153697.1	mRNA
NM_014320.2	mRNA
NM_001013703.2	mRNA
NM_000978.3	mRNA
NM_032576.2	mRNA
XM_936748.1	mRNA
NM_006573.3	mRNA
NM_003824.2	mRNA
NM_130782.2	mRNA
NM_018833.2	mRNA
NM_032882.2	mRNA
XM_928156.1	mRNA
NM_002727.2	mRNA
NM_005442.2	mRNA
NM_148976.1	mRNA
NM_207168.1	mRNA
NM_024834.1	mRNA
NM_001530.2	mRNA
NM_019610.3	mRNA
NM_006495.2	mRNA
NM_003749.2	mRNA
NM_001150.1	mRNA
XM_001131044.1	mRNA
NM_006037.2	mRNA
NM_152655.2	mRNA
NM_148962.4	mRNA
NM_006609.3	mRNA
NM_001010935.1	mRNA
NM_004125.2	mRNA
NM_021239.1	mRNA
NM_005717.2	mRNA
NM_001013699.1	mRNA
NM_001002009.1	mRNA
NM_001007074.1	mRNA
NM_024669.2	mRNA
XM_942212.2	mRNA
NM_004334.1	mRNA

NM_006807.3	mRNA
XM_935752.1	mRNA
XM_926814.1	mRNA
NM_001004322.1	mRNA
NM_014358.2	mRNA
NM_016407.1	mRNA
NM_002760.3	mRNA
NM_003264.3	mRNA
NM_033412.1	mRNA
NM_020529.1	mRNA
NM_018334.3	mRNA
NM_052961.2	mRNA
NM_020760.1	mRNA
NM_004202.2	mRNA
XM_934651.1	mRNA
NM_023075.4	mRNA
NM_198578.2	mRNA
NM_000544.3	mRNA
XM_942096.1	mRNA
NM_001562.2	mRNA
NM_007166.2	mRNA
NM_017780.2	mRNA
NM_001792.2	mRNA
XM_935186.1	mRNA
XM_930480.1	mRNA
NM_006242.3	mRNA
NM_001003712.1	mRNA
XM_001133926.1	mRNA
NM_001557.2	mRNA
NM_024095.3	mRNA
NM_006707.2	mRNA
NM_013364.3	mRNA
NM_006000.1	mRNA
NM_138287.2	mRNA
XM_936461.1	mRNA
NM_024586.3	mRNA
NM_020370.1	mRNA
NR_002189.2	mRNA
NM_198271.2	mRNA
NM_003245.2	mRNA
NM_002514.2	mRNA
NM_178231.1	mRNA
NM_016824.3	mRNA
NM_025180.3	mRNA
NM_001511.1	mRNA
NM_001040616.1	mRNA
NM_001013722.1	mRNA
NM_033222.2	mRNA

NM_016587.2	mRNA
NM_031476.1	mRNA
XM_931214.1	mRNA
NM_006825.2	mRNA
NM_001007535.1	mRNA
NM_145032.2	mRNA
NM_003972.2	mRNA
NM_170607.2	mRNA
NM_012428.2	mRNA
XM_497711.2	mRNA
NM_001866.2	mRNA
NM_002697.2	mRNA
XM_001134256.1	mRNA
NM_001721.4	mRNA
NM_004994.2	mRNA
NM_017839.3	mRNA
NM_000433.2	mRNA
NR_001545.1	mRNA
NM_145686.2	mRNA
NM_001629.2	mRNA
NM_148954.2	mRNA
NM_182802.1	mRNA
NM_003137.3	mRNA
NM_144673.2	mRNA
NM_022746.2	mRNA
NM_182757.2	mRNA
NM_144590.1	mRNA
NM_001025077.1	mRNA
XM_938919.1	mRNA
NM_005980.2	mRNA
NM_002784.2	mRNA
NM_033656.2	mRNA
NM_172027.1	mRNA
NM_152313.2	mRNA
NM_012482.3	mRNA
NM_024595.1	mRNA
NM_006305.2	mRNA
XM_939954.2	mRNA
NM_020686.4	mRNA
XM_371152.3	mRNA
NM_020920.2	mRNA
XM_944290.2	mRNA
NM_030578.2	mRNA
NM_145869.1	mRNA
NM_012387.1	mRNA
NM_182686.1	mRNA
NM_198795.1	mRNA
NM_023001.2	mRNA

NM_145913.2	mRNA
NM_019000.3	mRNA
XR_017962.1	mRNA
NM_030636.2	mRNA
NM_138687.1	mRNA
NM_006861.4	mRNA
NM_001621.2	mRNA
NM_080603.3	mRNA
NM_182703.3	mRNA
NM_080424.1	mRNA
NM_003389.2	mRNA
XM_930995.1	mRNA
NM_002186.2	mRNA
NM_017833.2	mRNA
NM_001080951.1	mRNA
NM_013361.2	mRNA
NR_002206.1	mRNA
CR738550	mRNA
AK097979	mRNA
AK055497	mRNA
AI655567	mRNA
AA417811	mRNA
BX108917	mRNA
BI026144	mRNA
BU151653	mRNA
DA385619	mRNA
DA572426	mRNA
BQ777029	mRNA
AL832858	mRNA
BC062546	mRNA
BU587445	mRNA
BX113731	mRNA
BM723547	mRNA
CR746297	mRNA
AV645572	mRNA
AW020492	mRNA
AK124771	mRNA
BQ439091	mRNA
BM703133	mRNA
BX111927	mRNA
BQ709709	mRNA
AA179392	mRNA
BM997836	mRNA
CB963631	mRNA
BQ948854	mRNA
BC045657	mRNA
BQ025535	mRNA
BM716528	mRNA

BX099079	mRNA
AW273831	mRNA
BU857004	mRNA
AI738931	mRNA
BC037864	mRNA
BX109404	mRNA
BF945346	mRNA
BX100504	mRNA
DA395916	mRNA
AJ227862	mRNA
AA744250	mRNA
AK129555	mRNA
AL832727	mRNA
CA396207	mRNA
BM668860	mRNA
BX092006	mRNA
BI492679	mRNA
CD238045	mRNA
AV651069	mRNA
AI678043	mRNA
BI915787	mRNA
BX117171	mRNA
AA938457	mRNA
N35069	mRNA
AI948831	mRNA
BE220308	mRNA
AK055485	mRNA
AL049390	mRNA
BE148741	mRNA
AL359560	mRNA
AA134590	mRNA
AK094521	mRNA
AK124299	mRNA
AK025332	mRNA
DR977977	mRNA
BX538075	mRNA
BI836710	mRNA
AA204695	mRNA
CD642055	mRNA
AV705309	mRNA
BE796139	mRNA
BX111162	mRNA
NM_003329.1	mRNA
