# Supplementary material for: The ceRNA Crosstalk between mRNAs and lncRNAs in Diabetes Myocardial Infarction
Source: Dis Markers. 2022 May 9;2022:4283534. doi: 10.1155/2022/4283534 (PMC9112177; doi:10.1155/2022/4283534)
Supplement: Supplementary 2 — Table S2: the differentially expressed miRNAs found in GSE112690. [file 4283534.f2.pdf]

ID_REF	SAMPLE 1	SAMPLE 2	SAMPLE 3	SAMPLE 4	SAMPLE 5	SAMPLE 6	SAMPLE 8	SAMPLE 1
hsa_miR_4	NA	NA	NA	NA	21.62745	NA	NA	20.406
hsa_miR_1	NA	NA	NA	NA	NA	NA	20.97495	NA
has_miR_4	NA	19.16549	17.41167	20.10947	16.74546	17.81623	20.37218	15.93473
hsa_miR_2	16.81364	13.10882	11.90237	14.34934	11.71852	12.48067	17.05926	11.2793

SAMPLE 1[illegible]	SAMPLE 1[illegible]	SAMPLE 1[illegible]	SAMPLE 1[illegible]	SAMPLE 1[illegible]	SAMPLE 1[illegible]	SAMPLE 1[illegible]	SAMPLE 1[illegible]	SAMPLE 1[illegible]
20.29419	21.96203	20.89518	21.45361	NA	NA	NA	20.3908	NA
20.544	NA	NA	NA	NA	NA	NA	NA	NA
16.16858	18.8438	16.3159	20.0424	NA	21.54157	17.79583	16.75162	18.29313
11.85411	12.71857	11.61339	11.34513	14.32036	18.04019	13.36034	11.22205	13.73786

SAMPLE 2(	SAMPLE 2:	SAMPLE 2:	SAMPLE 2‹	SAMPLE 2!	SAMPLE 2(	SAMPLE 2:	SAMPLE 2!	SAMPLE 3(
21.56171	NA	NA	NA	NA	NA	21.08756	NA	NA
NA	NA	NA	NA	NA	NA	21.36362	NA	NA
15.23375	18.71181	19.88769	NA	21.80207	20.71982	15.99602	19.2381	NA
10.50383	13.69927	13.94763	16.33523	15.15049	13.64122	11.81833	12.96049	15.284

SAMPLE 3[illegible]	SAMPLE 3[illegible]	SAMPLE 3[illegible]	SAMPLE 3[illegible]	SAMPLE 3[illegible]	SAMPLE 4[illegible]	SAMPLE 4[illegible]	SAMPLE 4[illegible]	SAMPLE 4[illegible]
NA	21.26031	NA	NA	NA	NA	NA	NA	NA
NA	NA	NA	NA	NA	NA	NA	NA	NA
18.96003	17.47397	NA	NA	NA	NA	21.68303	NA	21.03388
13.67284	12.67481	13.98653	15.16492	15.79735	18.96092	14.41464	17.53115	14.21527

**SAMPLE 4[illegible]**	**SAMPLE 4[illegible]**	**SAMPLE 4[illegible]**	**SAMPLE 5[illegible]**	**SAMPLE 5[illegible]**	**SAMPLE 5[illegible]**	**SAMPLE 5[illegible]**	**SAMPLE 5[illegible]**	**SAMPLE 5[illegible]**
NA	21.74166	20.58315	20.95206	NA	20.62812	NA	NA	NA
NA	NA	NA	NA	NA	NA	NA	NA	NA
NA	NA	17.36558	17.55988	NA	17.69982	18.98096	17.71117	NA
16.07021	13.58342	11.48253	13.09753	15.33193	13.40838	15.0089	13.13871	15.19101

SAMPLE 5[illegible]	SAMPLE 5[illegible]	SAMPLE 5[illegible]	SAMPLE 6[illegible]	SAMPLE 6[illegible]	SAMPLE 6[illegible]	SAMPLE 6[illegible]	SAMPLE 6[illegible]	SAMPLE 6[illegible]
NA	NA	20.58877	21.88975	NA	NA	NA	NA	NA
NA	NA	NA	NA	NA	NA	NA	NA	NA
NA	17.97505	17.98546	18.0872	18.04114	18.61819	17.8926	NA	17.96698
14.14163	15.41161	13.12914	13.40109	12.90966	15.7957	15.36431	15.05674	16.67234

SAMPLE 6[illegible]	SAMPLE 6[illegible]	SAMPLE 7[illegible]	SAMPLE 7[illegible]	SAMPLE 7[illegible]	SAMPLE 7[illegible]	SAMPLE 7[illegible]	SAMPLE 7[illegible]	SAMPLE 7[illegible]
21.92042	NA	20.6819	20.1233	NA	NA	20.50163	19.57306	NA
NA	NA	NA	NA	NA	NA	20.02025	NA	NA
16.78176	19.25429	17.38595	16.44638	NA	17.00555	18.07298	14.01767	NA
14.23855	13.44627	12.19542	11.89305	16.71513	12.25064	13.87401	10.10492	13.53198

SAMPLE 7[illegible]	SAMPLE 8[illegible]	SAMPLE 8[illegible]	SAMPLE 8[illegible]	SAMPLE 8[illegible]	SAMPLE 8[illegible]	SAMPLE 9[illegible]	SAMPLE 9[illegible]	SAMPLE 9[illegible]
20.47586	NA	NA	NA	NA	NA	NA	NA	NA
20.18259	NA	NA	NA	18.23661	NA	NA	NA	NA
16.3524	17.68951	NA	18.59093	NA	17.56083	NA	20.67471	NA
13.3776	13.46086	16.31019	13.1323	16.03175	14.45111	17.77344	14.52784	15.36013

SAMPLE 9	SAMPLE 9	SAMPLE 1(	SAMPLE 1(	SAMPLE 1(	SAMPLE 1(	SAMPLE 1(	SAMPLE 1(	SAMPLE 1(
NA	NA	NA	NA	NA	NA	NA	NA	NA
NA	NA	NA	20.33971	NA	21.93474	NA	NA	NA
16.6826	14.82194	16.89285	18.12342	16.9251	21.91382	NA	NA	NA
13.32138	11.8448	13.98723	14.74712	14.58109	15.26902	15.11772	17.24201	14.18921

SAMPLE 1(	SAMPLE 1:	SAMPLE 1:	SAMPLE 1:	SAMPLE 1:	SAMPLE 1:	SAMPLE 1:	SAMPLE 1:	SAMPLE 1:
NA	NA	NA	NA	NA	21.96414	NA	19.41391	21.64185
NA	NA	NA	NA	NA	NA	NA	21.35592	NA
NA	20.45711	21.72979	20.51366	16.46058	18.81439	NA	14.27734	17.26818
16.82357	14.52656	14.84342	13.85347	13.91408	13.24796	14.08902	10.68021	13.9771

SAMPLE 1:	SAMPLE 1:	SAMPLE 1:	SAMPLE 1:	SAMPLE 1:	SAMPLE 1:	SAMPLE 1:	SAMPLE 1:	SAMPLE 1:
21.44055	NA	NA	NA	NA	21.19941	NA	21.76696	21.73044
NA	NA	NA	NA	NA	NA	21.20835	NA	20.99379
19.65211	19.49167	21.77454	21.80458	16.88467	17.53101	20.71889	17.09956	15.63982
13.74509	12.96578	13.56867	14.74801	13.84309	14.93915	13.36239	15.16127	13.58738

SAMPLE 1[illegible]	SAMPLE 1[illegible]	SAMPLE 1[illegible]	SAMPLE 1[illegible]	SAMPLE 1[illegible]	SAMPLE 1[illegible]	SAMPLE 1[illegible]	SAMPLE 1[illegible]	SAMPLE 1[illegible]
NA	NA	18.75858	NA	NA	20.05868	NA	21.67958	NA
NA	NA	NA	NA	NA	20.7143	NA	NA	NA
NA	18.03415	13.99636	21.44189	20.81748	14.05425	17.88789	19.49763	17.30812
16.30249	12.82618	11.39555	15.39625	12.03556	11.55661	13.87722	15.89872	14.2878

SAMPLE 1[illegible]	SAMPLE 1[illegible]	SAMPLE 1[illegible]	SAMPLE 1[illegible]	SAMPLE 1[illegible]	SAMPLE 1[illegible]	SAMPLE 1[illegible]	SAMPLE 1[illegible]	SAMPLE 1[illegible]
NA	NA	NA	NA	20.91265	21.29975	21.13332	21.26715	20.79721
NA	20.89118	NA	NA	NA	NA	20.10978	NA	NA
17.18863	21.20353	17.18488	18.50231	17.81123	20.41376	16.53959	16.52726	16.13661
14.24856	18.48671	14.22579	15.08355	12.97093	13.08764	13.92992	14.28499	11.91837

SAMPLE 1!	SAMPLE 1!	SAMPLE 1!	SAMPLE 1!	SAMPLE 1(	SAMPLE 1(	SAMPLE 1(	SAMPLE 1(	SAMPLE 1(
20.96716	NA	NA	NA	20.77473	21.32041	20.12199	NA	NA
21.94265	NA	NA	NA	NA	NA	NA	NA	NA
14.55633	20.81772	16.85436	19.70076	16.36382	15.30881	15.13215	18.14382	NA
12.58571	19.37189	14.50096	14.56045	13.66929	13.35751	10.17002	15.38849	15.61381

SAMPLE 1[illegible]	SAMPLE 1[illegible]	SAMPLE 1[illegible]	SAMPLE 1[illegible]	SAMPLE 1[illegible]	SAMPLE 1[illegible]	SAMPLE 1[illegible]	SAMPLE 1[illegible]	SAMPLE 1[illegible]
NA	NA	21.15064	21.02233	NA	NA	NA	NA	20.62815
NA	NA	20.74042	20.82096	NA	NA	21.34257	NA	19.63841
16.86831	15.70126	17.88591	18.00043	18.44175	19.3339	15.59754	16.23037	17.03492
14.79523	13.24724	15.46203	15.15635	16.62277	16.86335	13.274	13.79494	13.9755

SAMPLE 18	SAMPLE 18	SAMPLE 18	SAMPLE 18	SAMPLE 18	SAMPLE 18	SAMPLE 19	SAMPLE 19	SAMPLE 19
NA	NA	NA	21.21394	NA	20.82938	NA	21.87908	NA
NA	NA	NA	NA	NA	20.6948	NA	NA	NA
17.29367	18.47523	18.0807	15.89682	16.60466	13.67533	17.2972	15.35072	15.78499
15.24057	16.71346	15.0079	13.68185	14.10929	12.84209	14.56748	13.45011	13.47698

SAMPLE 1!	SAMPLE 1!	SAMPLE 1!	SAMPLE 1!	SAMPLE 1!	SAMPLE 2(	SAMPLE 2(	SAMPLE 2(	SAMPLE 2(
21.96268	20.80346	NA	20.2044	20.64566	21.37221	20.30755	20.17305	18.28001
NA	NA	NA	NA	NA	NA	NA	18.36373	NA
15.49249	15.174	21.33861	13.95377	14.59416	16.30797	15.70609	12.55045	10.81389
13.19008	12.7462	15.52364	9.600353	10.45049	11.84986	12.26637	8.756581	7.442652

SAMPLE 2(	SAMPLE 2(	SAMPLE 2(	SAMPLE 2:	SAMPLE 2:	SAMPLE 2:	SAMPLE 2:	SAMPLE 2:	SAMPLE 2:
18.50962	20.71176	NA	NA	NA	19.94498	20.99469	21.38967	NA
NA	NA	NA	21.93916	20.08058	NA	NA	NA	NA
13.67875	16.36766	16.07376	16.26382	13.49633	12.30974	16.33088	15.30716	18.1714
9.411159	11.6916	21.21671	13.2395	9.840706	7.991071	10.2879	10.98439	13.68931

SAMPLE 2[illegible]	SAMPLE 2[illegible]	SAMPLE 2[illegible]	SAMPLE 2[illegible]	SAMPLE 2[illegible]	SAMPLE 2[illegible]	SAMPLE 2[illegible]	SAMPLE 2[illegible]	SAMPLE 2[illegible]
20.51869	NA	NA	NA	19.81456	NA	19.96708	NA	20.26612
NA	NA	NA	NA	NA	NA	NA	NA	20.88049
14.36578	16.76523	17.33531	19.1631	14.60532	17.60303	15.25933	18.02448	14.45143
9.888546	12.0046	17.3858	11.57889	10.06102	12.6806	11.10099	14.0717	10.62198

SAMPLE 2:	SAMPLE 2:	SAMPLE 2:	SAMPLE 2:	SAMPLE 2	SAMPLE 2	SAMPLE 2	SAMPLE 2	SAMPLE 2
NA	21.73313	21.60738	NA	19.13526	21.835	19.80324	21.63244	NA
NA	NA	NA	NA	19.74013	NA	NA	NA	NA
17.08157	17.14688	15.15934	15.68441	13.68039	NA	14.25198	14.51287	16.65426
13.28624	11.9058	10.20979	11.68961	9.003936	NA	10.10305	9.859328	11.91448

SAMPLE 2[illegible]	SAMPLE 2[illegible]	SAMPLE 2[illegible]	SAMPLE 2[illegible]	SAMPLE 2[illegible]	SAMPLE 2[illegible]	SAMPLE 2[illegible]	SAMPLE 2[illegible]	SAMPLE 2[illegible]
20.18324	20.1475	20.08083	20.26743	NA	20.80734	21.09156	21.46212	20.91675
NA	21.82897	21.23804	NA	NA	NA	NA	NA	NA
15.28535	13.54616	12.90246	14.20774	15.01573	15.78545	16.17662	15.84891	13.64065
10.78376	8.753276	8.686641	9.953928	10.70958	11.05958	11.46495	11.72325	9.980806

SAMPLE 25	SAMPLE 2(	SAMPLE 2(	SAMPLE 2(	SAMPLE 2(	SAMPLE 2(	SAMPLE 2(	SAMPLE 2(	SAMPLE 27
NA	20.18937	NA	NA	NA	NA	20.11712	NA	NA
NA	20.59467	NA	NA	NA	NA	NA	NA	NA
NA	13.47983	16.46057	16.68778	17.22944	20.0809	16.91954	17.13248	17.43274
NA	8.376382	13.62229	14.12098	14.88317	15.05257	12.46899	14.25466	15.26372

**SAMPLE 2[illegible]**	**SAMPLE 2[illegible]**	**SAMPLE 2[illegible]**	**SAMPLE 2[illegible]**	**SAMPLE 2[illegible]**	**SAMPLE 2[illegible]**	**SAMPLE 2[illegible]**	**SAMPLE 2[illegible]**	**SAMPLE 2[illegible]**
NA	NA	NA	20.96855	NA	21.66573	NA	NA	NA
NA	NA	NA	NA	NA	NA	21.13467	NA	NA
20.85868	18.00166	19.96289	16.88032	17.59733	18.06307	19.90491	16.74231	17.98772
17.58681	15.48247	16.43837	14.38442	14.78858	13.79603	12.36328	13.57614	14.43741

SAMPLE 2[illegible]	SAMPLE 2[illegible]	SAMPLE 2[illegible]	SAMPLE 2[illegible]	SAMPLE 2[illegible]	SAMPLE 2[illegible]	SAMPLE 2[illegible]	SAMPLE 2[illegible]	SAMPLE 2[illegible]
20.98865	NA	18.55834	17.72918	20.59811	20.77282	NA	NA	NA
NA	NA	21.22695	20.43866	NA	NA	NA	NA	NA
16.53303	17.48876	14.47127	11.83317	16.68231	16.81049	16.7735	18.52258	17.46614
13.08297	14.15683	11.11266	10.01415	13.37679	12.83976	13.18258	14.57972	13.80326

SAMPLE 2[illegible]	SAMPLE 2[illegible]	SAMPLE 2[illegible]	SAMPLE 2[illegible]	SAMPLE 2[illegible]	SAMPLE 2[illegible]	SAMPLE 3[illegible]	SAMPLE 3[illegible]	SAMPLE 3[illegible]
NA	19.86701	NA	NA	20.89383	NA	NA	NA	NA
NA	20.58354	NA	NA	NA	NA	NA	NA	NA
16.91461	14.66966	16.69411	16.57326	16.70854	20.67998	16.00686	17.99048	15.59694
13.83403	11.68999	13.23482	13.22106	13.51324	12.69099	14.34471	15.22162	13.28217

SAMPLE 3(	SAMPLE 3(	SAMPLE 3(	SAMPLE 3(	SAMPLE 3(	SAMPLE 3(	SAMPLE 3:	SAMPLE 3:	SAMPLE 3:
20.38392	21.11975	NA	20.42376	NA	21.92019	21.44096	20.13496	NA
NA	NA	NA	NA	NA	NA	NA	NA	21.06493
16.51272	15.77696	17.64775	16.97855	18.88094	17.69431	16.93114	14.6002	16.27095
13.53812	13.12186	14.29482	14.23099	15.03847	14.5577	12.44458	12.66888	14.58491

SAMPLE 3:	SAMPLE 3:	SAMPLE 3:	SAMPLE 3:	SAMPLE 3:	SAMPLE 3:	SAMPLE 3:	SAMPLE 3:	SAMPLE 3:
19.25389	18.90284	NA	21.36541	NA	NA	NA	NA	NA
NA	NA	NA	NA	NA	NA	NA	NA	NA
15.40181	14.10556	18.09081	14.69338	19.49871	20.19236	19.49505	18.26881	18.66012
12.4502	11.73641	15.99355	12.69331	16.3135	16.10709	15.47999	16.21928	16.11746

SAMPLE 3[illegible]	SAMPLE 3[illegible]	SAMPLE 7	SAMPLE 9	SAMPLE 2[illegible]	SAMPLE 2[illegible]	SAMPLE 3[illegible]	SAMPLE 3[illegible]	SAMPLE 3[illegible]
NA	NA	NA	18.30804	19.89488	19.53259	NA	NA	NA
NA	NA	NA	20.36298	NA	21.28797	NA	NA	NA
17.98148	16.0928	19.54155	15.00832	16.70669	13.65865	17.71903	20.21248	NA
14.2534	14.84031	13.36223	10.7367	11.72438	9.872611	11.70032	17.8352	16.2766

SAMPLE 3[illegible]	SAMPLE 4[illegible]	SAMPLE 4[illegible]	SAMPLE 4[illegible]	SAMPLE 5[illegible]	SAMPLE 6[illegible]	SAMPLE 6[illegible]	SAMPLE 7[illegible]	SAMPLE 7[illegible]
NA	NA	NA	NA	NA	NA	NA	NA	20.63282
NA	NA	NA	NA	NA	NA	NA	NA	20.41122
NA	NA	19.58818	17.49653	NA	18.39966	20.92027	17.89146	16.37969
14.21147	14.20917	14.74111	12.46194	16.91218	13.10475	13.9186	13.25163	14.97232

SAMPLE 8[illegible]	SAMPLE 8[illegible]	SAMPLE 8[illegible]	SAMPLE 8[illegible]	SAMPLE 9[illegible]	SAMPLE 9[illegible]	SAMPLE 9[illegible]	SAMPLE 9[illegible]	SAMPLE 9[illegible]
NA	NA	NA	NA	18.09743	NA	NA	NA	21.98979
NA	NA	NA	NA	NA	NA	21.26209	NA	21.92731
NA	NA	NA	20.69605	13.72273	14.05357	NA	NA	15.58049
13.86984	15.3069	12.33946	13.20648	10.40056	13.59922	15.42867	16.20969	13.51868

SAMPLE 1(	SAMPLE 1(	SAMPLE 1:	SAMPLE 1:	SAMPLE 1:	SAMPLE 1:	SAMPLE 1:	SAMPLE 1·	SAMPLE 1·
NA	NA	NA	NA	NA	NA	NA	NA	NA
NA	21.69824	NA	NA	NA	NA	NA	NA	NA
NA	20.01697	19.73502	NA	NA	NA	NA	17.92586	16.70937
13.45481	13.59966	12.49154	19.55356	12.80218	13.80926	14.71596	12.73793	15.18245

**SAMPLE 1[illegible]**	**SAMPLE 1[illegible]**	**SAMPLE 1[illegible]**	**SAMPLE 1[illegible]**	**SAMPLE 1[illegible]**	**SAMPLE 1[illegible]**	**SAMPLE 1[illegible]**	**SAMPLE 1[illegible]**	**SAMPLE 1[illegible]**
NA	21.77128	17.87117	NA	NA	NA	NA	20.38625	NA
NA	NA	NA	NA	NA	NA	NA	NA	NA
20.82093	17.15527	14.76983	17.43418	18.29078	NA	18.86011	17.02976	18.53986
13.85835	11.72971	11.02056	12.2224	13.3562	14.58648	15.68594	12.40897	16.01648

SAMPLE 1	SAMPLE 1	SAMPLE 1	SAMPLE 184	SAMPLE 185	SAMPLE 196	SAMPLE 199
20.72475	NA	NA	NA	NA	19.50484897	18.63473355
NA	NA	NA	NA	NA	NA	NA
15.12727	17.61753	20.79559	19.08899356	15.86175005	13.99554893	13.55365788
13.78521	14.95498	12.79536	16.59662639	13.5222616	9.868774111	9.69216929

SAMPLE 201	SAMPLE 207	SAMPLE 209	SAMPLE 211	SAMPLE 212	SAMPLE 215
20.1197196	18.83379957	19.81831993	NA	NA	NA
21.55895024	NA	20.51915811	NA	21.828746	21.92313879
14.55384935	13.45946174	13.75948421	15.2057764	14.15911121	13.4396196
10.04873134	9.617049036	10.73902755	12.34887701	12.35774118	9.137443807

SAMPLE 216	SAMPLE 218	SAMPLE 220	SAMPLE 223	SAMPLE 226	SAMPLE 228
19.23129557	16.81281397	NA	20.77724593	19.20567874	20.02302595
NA	20.86696497	NA	NA	NA	NA
14.83772666	12.51263574	17.1278504	15.95045004	14.04884835	15.40036713
11.2184057	8.530058909	11.55508278	11.24172194	10.10945681	11.34860484

SAMPLE 231	SAMPLE 237	SAMPLE 240	SAMPLE 241	SAMPLE 247	SAMPLE 248
17.70422252	NA	21.71058466	20.57086866	NA	NA
21.53680407	NA	NA	NA	NA	NA
13.55516168	17.03318621	15.80468997	14.89899842	13.60518924	16.24995572
9.550553141	12.72442533	10.9513848	10.65445662	9.956791233	11.97289763

SAMPLE 258	SAMPLE 260	SAMPLE 262	SAMPLE 268	SAMPLE 276	SAMPLE 292
NA	20.27094698	20.31562495	20.70309632	20.95113029	NA
NA	NA	NA	NA	NA	NA
16.23623654	13.89257284	13.71560047	18.22682614	17.46972196	14.9036531
11.65621752	10.23515234	9.137712345	13.00266034	13.64161863	12.98890777

SAMPLE 297	SAMPLE 298	SAMPLE 300	SAMPLE 310	SAMPLE 314	SAMPLE 315	SAMPLE 321
NA	NA	19.55973184	NA	NA	NA	NA
NA	NA	20.49622537	NA	NA	NA	NA
16.42415221	17.85603208	16.59171906	17.281753	17.86738932	16.8150523	18.81006564
13.82239157	14.41680279	13.99838996	14.4670326	14.55487681	14.22242888	15.98375027

SAMPLE 322	SAMPLE 325	SAMPLE 326	SAMPLE 329	miRNA_ID	pvalue	foldchange
NA	NA	NA	NA	hsa-miR-4306	0.000991	1.897824093
NA	NA	NA	NA	hsa-miR-155-5p	0.031	0.705053598
15.59306571	19.41081921	16.03111146	18.45669569	hsa-miR-423-5p	0.047	1.519877574
13.72088587	16.32082499	14.55097795	15.53296292	hsa-miR-21-5p	0.05	1.444567157
