# Supplementary material for: The ceRNA Crosstalk between mRNAs and lncRNAs in Diabetes Myocardial Infarction
Source: Dis Markers. 2022 May 9;2022:4283534. doi: 10.1155/2022/4283534 (PMC9112177; doi:10.1155/2022/4283534)
Supplement: Supplementary 3 — Table S3: the gene ontology (GO) term enrichment for differentially expressed genes. [file 4283534.f3.pdf]

ID	Description	GeneRatio	BgRatio	pvalue	p.adjust	qvalue	geneID	Count
GO:00421	neutrophil act	37/272	498/1867(	3.25E-16	1.15E-12	9.94E-13	PLAU/MNI	37
GO:0024	neutrophil me	36/272	499/1867(	2.12E-15	3.75E-12	3.24E-12	PLAU/MNI	36
GO:00433	neutrophil de	35/272	485/1867(	5.33E-15	5.68E-12	4.9E-12	PLAU/MNI	35
GO:00022	neutrophil act	35/272	488/1867(	6.42E-15	5.68E-12	4.9E-12	PLAU/MNI	35
GO:00313	positive regul	20/272	384/1867(	9.78E-07	0.000692	0.000597	RPS6KA5/I	20
GO:00327	positive regul	9/272	86/18670	4.47E-06	0.002108	0.001818	IFNGR1/TH	9
GO:00027	regulation of	22/272	500/1867(	4.48E-06	0.002108	0.001818	CACNA1E/	22
GO:00018	positive regul	21/272	464/1867(	4.83E-06	0.002108	0.001818	SULF2/MN	21
GO:19035	positive regul	9/272	88/18670	5.42E-06	0.002108	0.001818	IFNGR1/TH	9
GO:00326	chemokine pr	9/272	89/18670	5.95E-06	0.002108	0.001818	F2RL1/IL4I	9
GO:20001	regulation of	14/272	239/1867(	1.16E-05	0.003727	0.003215	RPS6KA3/I	14
GO:00432	regulation of	13/272	215/1867(	1.7E-05	0.005025	0.004334	RPS6KA3/I	13
GO:00507	regulation of	20/272	472/1867(	2.12E-05	0.005754	0.004964	CACNA1E/	20
GO:00027	positive regul	15/272	288/1867(	2.28E-05	0.005754	0.004964	F2RL1/IFN	15
GO:00028	regulation of	18/272	400/1867(	2.51E-05	0.005934	0.005119	RPS6KA5/I	18
GO:00431	negative regul	8/272	84/18670	3.07E-05	0.006784	0.005852	RPS6KA3/I	8
GO:00022	pattern recog	12/272	197/1867(	3.34E-05	0.006959	0.006003	RPS6KA3/I	12
GO:00507	positive regul	14/272	268/1867(	4.13E-05	0.00812	0.007005	F2RL1/IFN	14
GO:00022	toll-like recep	10/272	146/1867(	5.68E-05	0.010482	0.009042	RPS6KA3/I	10
GO:20001	negative regul	8/272	92/18670	5.92E-05	0.010482	0.009042	RPS6KA3/I	8
GO:00725	reactive oxyge	14/272	284/1867(	7.71E-05	0.012903	0.011131	F2RL1/PTG	14
GO:00716	granulocyte c	9/272	123/1867(	8.02E-05	0.012903	0.011131	THBS1/CAI	9
GO:00507	positive regul	10/272	153/1867(	8.42E-05	0.012963	0.011182	PTGS2/CLE	10
GO:00321	activation of	15/272	333/1867(	0.000118	0.017413	0.015021	TXN/GADD	15
GO:00985	detection of e	4/272	19/18670	0.000144	0.020354	0.017557	CLEC7A/LY	4
GO:00711	regulation of	8/272	108/1867(	0.000183	0.023586	0.020346	ARID3A/G	8
GO:00510	positive regul	17/272	428/1867(	0.000192	0.023586	0.020346	NKX3-1/F2	17
GO:00711	positive regul	7/272	82/18670	0.000193	0.023586	0.020346	ARID3A/G	7
GO:00070	cell cycle arre	12/272	237/1867(	0.000195	0.023586	0.020346	CDC123/N	12
GO:00507	positive regul	9/272	139/1867(	0.000204	0.023586	0.020346	F2RL1/IFN	9
GO:00346	response to tu	14/272	312/1867(	0.000207	0.023586	0.020346	NKX3-1/F2	14
GO:00328	negative regul	4/272	21/18670	0.000217	0.023996	0.0207	THBS1/RG	4
GO:00975	granulocyte m	9/272	141/1867(	0.000227	0.024369	0.021021	THBS1/CAI	9
GO:00009	nuclear-transc	11/272	207/1867(	0.000237	0.024657	0.02127	RPS28/RPS	11
GO:00512	positive regul	17/272	440/1867(	0.000265	0.026843	0.023155	F2RL1/IFN	17
GO:19035	positive regul	16/272	403/1867(	0.000297	0.028124	0.02426	NKX3-1/F2	16
GO:20012	positive regul	10/272	179/1867(	0.000304	0.028124	0.02426	PAK2/NKX	10
GO:00064	mRNA catabol	15/272	364/1867(	0.000309	0.028124	0.02426	RPS28/RPS	15
GO:00709	protein localiz	9/272	147/1867(	0.00031	0.028124	0.02426	RPS28/RPS	9
GO:00713	cellular respo	13/272	291/1867(	0.000361	0.031986	0.027592	NKX3-1/F2	13
GO:00001	nuclear-transc	8/272	120/1867(	0.000377	0.032534	0.028065	RPS28/RPS	8
GO:19049	positive regul	17/272	456/1867(	0.0004	0.033738	0.029103	F2RL1/IFN	17
GO:00725	establishment	8/272	122/1867(	0.000421	0.034673	0.02991	RPS28/RPS	8
GO:00324	detection of n	3/272	11/18670	0.000463	0.037231	0.032117	LY96/TLR1	3
GO:00026	positive regul	15/272	380/1867(	0.000485	0.038167	0.032924	PAK2/PPP2	15
GO:00507	positive regul	6/272	69/18670	0.000503	0.038271	0.033014	F2RL1/SIRI	6
GO:00190	viral gene exp	10/272	191/1867(	0.000508	0.038271	0.033014	RPS28/RPS	10

GO:00064:	translational i	10/272	193/1867(	0.000551	0.040651	0.035066	RPS28/RPS	10
GO:00326{	regulation of t	9/272	160/1867(	0.000576	0.04086	0.035247	IFNGR1/TH	9
GO:00507(	regulation of p	7/272	98/18670	0.000577	0.04086	0.035247	F2RL1/SIRI	7
GO:20003:	regulation of r	10/272	195/1867(	0.000597	0.041457	0.035762	F2RL1/PTG	10
GO:00326	tumor necrosi	9/272	163/1867(	0.000658	0.042144	0.036355	IFNGR1/TH	9
GO:19035!	regulation of t	9/272	163/1867(	0.000658	0.042144	0.036355	IFNGR1/TH	9
GO:19037!	negative regul	4/272	28/18670	0.000685	0.042144	0.036355	THBS1/RG!	4
GO:00485:	negative regul	7/272	101/1867(	0.000692	0.042144	0.036355	STAT1/SRF	7
GO:00486(	positive regula	7/272	101/1867(	0.000692	0.042144	0.036355	PTGS2/THI	7
GO:00508(	positive regula	15/272	394/1867(	0.000704	0.042144	0.036355	PAK2/PPP2	15
GO:00327!	positive regula	5/272	49/18670	0.000715	0.042144	0.036355	F2RL1/CLE	5
GO:20012	regulation of i	9/272	165/1867(	0.000718	0.042144	0.036355	NKX3-1/PT	9
GO:20003:	positive regula	7/272	102/1867(	0.000733	0.042144	0.036355	F2RL1/PTG	7
GO:00064(	RNA catabolic	15/272	397/1867(	0.00076	0.042144	0.036355	RPS28/RPS	15
GO:00516(	defense respo	11/272	238/1867(	0.000768	0.042144	0.036355	TRIM56/F2	11
GO:00332(	tumor necrosi	9/272	167/1867(	0.000783	0.042144	0.036355	F2RL1/TNF	9
GO:19039(	negative regul	3/272	13/18670	0.000785	0.042144	0.036355	THBS1/RG!	3
GO:00024:	antigen proce	6/272	75/18670	0.000786	0.042144	0.036355	PSMD12/B	6
GO:01500:	neuroinflamm	6/272	75/18670	0.000786	0.042144	0.036355	IFNGR1/PT	6
GO:00717(	tumor necrosi	9/272	168/1867(	0.000817	0.042232	0.03643	IFNGR1/TH	9
GO:00506(	cytokine secre	11/272	240/1867(	0.000822	0.042232	0.03643	F2RL1/IFN(	11
GO:00305!	neutrophil che	7/272	104/1867(	0.000823	0.042232	0.03643	CAMK1D/(	7
GO:00066:	SRP-depender	7/272	105/1867(	0.000871	0.044058	0.038005	RPS28/RPS	7
GO:00095!	detection of b	4/272	30/18670	0.000896	0.044677	0.038539	CLEC7A/LY	4
GO:00022{	T cell activatic	7/272	106/1867(	0.000921	0.044856	0.038693	F2RL1/CLE	7
GO:00096:	response to fu	5/272	52/18670	0.00094	0.044856	0.038693	CLEC7A/S1	5
GO:00300:	insulin secreti	10/272	207/1867(	0.000945	0.044856	0.038693	CACNA1E/	10
GO:20012:	regulation of a	15/272	406/1867(	0.000953	0.044856	0.038693	PAK2/NKX:	15
GO:00902:	regulation of p	10/272	208/1867(	0.000981	0.044856	0.038693	CACNA1E/	10
GO:19033:	regulation of r	13/272	324/1867(	0.000984	0.044856	0.038693	TOB1/RBM	13
GO:00985	detection of o	3/272	14/18670	0.000988	0.044856	0.038693	CLEC7A/TL	3
GO:19046!	glucose transr	7/272	108/1867(	0.001029	0.045731	0.039448	CLTCL1/HK	7
GO:00425!	antigen proce	6/272	79/18670	0.001033	0.045731	0.039448	PSMD12/B	6
GO:00507(	regulation of c	10/272	210/1867(	0.001055	0.046087	0.039755	F2RL1/IFN(	10
GO:00066:	cotranslationa	7/272	109/1867(	0.001086	0.046877	0.040437	RPS28/RPS	7
GO:00028:	positive regula	11/272	249/1867(	0.001109	0.047027	0.040567	RPS6KA5/F	11
GO:00507!	regulation of i	9/272	176/1867(	0.001135	0.047027	0.040567	CACNA1E/	9
GO:00022:	activation of i	8/272	142/1867(	0.001144	0.047027	0.040567	RPS6KA5/F	8
GO:00300:	peptide horm	11/272	250/1867(	0.001145	0.047027	0.040567	CACNA1E/	11
GO:00190{	viral transcrip	9/272	177/1867(	0.001181	0.047027	0.040567	RPS28/RPS	9
GO:00341:	toll-like recep	3/272	15/18670	0.001222	0.047027	0.040567	F2RL1/TLR	3
GO:00445:	long-chain fat	3/272	15/18670	0.001222	0.047027	0.040567	ACSL1/THE	3
GO:00517:	positive regula	3/272	15/18670	0.001222	0.047027	0.040567	STAT1/TLR	3
GO:01403!	lipid import in	3/272	15/18670	0.001222	0.047027	0.040567	ACSL1/THE	3
GO:20004{	positive regula	3/272	15/18670	0.001222	0.047027	0.040567	F2RL1/TLR	3
GO:00326	regulation of c	6/272	82/18670	0.001256	0.047793	0.041227	F2RL1/IL4F	6
GO:00086	hexose transn	7/272	112/1867(	0.001272	0.047921	0.041338	CLTCL1/HK	7
GO:00513	negative regul	16/272	463/1867(	0.001311	0.048591	0.041916	RPS6KA3/F	16

GO:00069:	DNA damage i	5/272	56/18670	0.001318	0.048591	0.041916	ARID3A/G/	5
GO:00022:	stimulatory C-	7/272	113/1867(	0.00134	0.048902	0.042184	RPS6KA5/F	7
GO:00157	monosacchari	7/272	114/1867(	0.00141	0.050533	0.043591	CLTCL1/HK	7
GO:00724	signal transdu	5/272	57/18670	0.001427	0.050533	0.043591	ARID3A/G/	5
GO:19024(	intracellular si	5/272	57/18670	0.001427	0.050533	0.043591	ARID3A/G/	5
GO:00525	regulation of e	15/272	425/1867(	0.0015	0.051316	0.044266	RPS6KA3/F	15
GO:19039(	negative regul	6/272	85/18670	0.001512	0.051316	0.044266	SRPK2/IFIT	6
GO:00327	positive regula	5/272	58/18670	0.001544	0.051316	0.044266	IL4R/CLEC	5
GO:00510	positive regula	8/272	149/1867(	0.001556	0.051316	0.044266	RPS6KA5/E	8
GO:00022	innate immun	7/272	116/1867(	0.001559	0.051316	0.044266	RPS6KA5/F	7
GO:00342	carbohydrate	7/272	116/1867(	0.001559	0.051316	0.044266	CLTCL1/HK	7
GO:00510	positive regula	11/272	261/1867(	0.001615	0.051316	0.044266	RPS6KA5/N	11
GO:20010	negative regul	4/272	35/18670	0.001617	0.051316	0.044266	TXN/RGS2,	4
GO:00027	innate immun	7/272	117/1867(	0.001638	0.051316	0.044266	RPS6KA5/F	7
GO:00468	regulation of l	11/272	262/1867(	0.001664	0.051316	0.044266	CACNA1E/	11
GO:00328	regulation of c	5/272	59/18670	0.001667	0.051316	0.044266	THBS1/RG	5
GO:00450	negative regul	5/272	59/18670	0.001667	0.051316	0.044266	SRPK2/IFIT	5
GO:00724	signal transdu	5/272	59/18670	0.001667	0.051316	0.044266	ARID3A/G/	5
GO:19024(	signal transdu	5/272	59/18670	0.001667	0.051316	0.044266	ARID3A/G/	5
GO:19024(	signal transdu	5/272	59/18670	0.001667	0.051316	0.044266	ARID3A/G/	5
GO:00305	leukocyte che	10/272	224/1867(	0.001708	0.051616	0.044525	F2RL1/THE	10
GO:00450	protein target	7/272	118/1867(	0.001721	0.051616	0.044525	RPS28/RPS	7
GO:19902	neutrophil mi	7/272	118/1867(	0.001721	0.051616	0.044525	CAMK1D/C	7
GO:00325(	maintenance	5/272	60/18670	0.001797	0.05302	0.045736	TXN/GOLP	5
GO:00463	regulation of g	5/272	60/18670	0.001797	0.05302	0.045736	CLTCL1/HK	5
GO:00163	dephosphoryl	16/272	478/1867(	0.001814	0.053068	0.045778	PPP2R3A/F	16
GO:00308	regulation of l	5/272	61/18670	0.001935	0.056142	0.048429	MNDA/TN	5
GO:00468	hormone secr	12/272	308/1867(	0.001952	0.056167	0.048451	CACNA1E/	12
GO:00451	maintenance	6/272	90/18670	0.002027	0.057874	0.049924	TXN/GOLP	6
GO:00097	response to ca	10/272	230/1867(	0.002074	0.058749	0.050678	PTGS2/PFK	10
GO:00435	regulation of t	3/272	18/18670	0.002122	0.059154	0.051028	RPS6KA3/E	3
GO:19020(	fatty acid tran	3/272	18/18670	0.002122	0.059154	0.051028	THBS1/IRS	3
GO:00463	positive regula	4/272	38/18670	0.002203	0.060801	0.052448	CLTCL1/IRS	4
GO:00315	mitotic G1 DN	5/272	63/18670	0.002233	0.060801	0.052448	ARID3A/G/	5
GO:00448	mitotic G1/S t	5/272	63/18670	0.002233	0.060801	0.052448	ARID3A/G/	5
GO:00447	G1 DNA dama	5/272	64/18670	0.002394	0.064684	0.055798	ARID3A/G/	5
GO:00099	hormone tran	12/272	317/1867(	0.002474	0.066352	0.057237	CACNA1E/	12
GO:00421	macrophage a	6/272	95/18670	0.002665	0.070443	0.060766	IFNGR1/TH	6
GO:00508	defense respo	4/272	40/18670	0.002666	0.070443	0.060766	S100A9/IL	4
GO:00525	regulation of p	15/272	452/1867(	0.00271	0.071063	0.0613	RPS6KA3/F	15
GO:00024	antigen proce	6/272	96/18670	0.002808	0.072829	0.062824	PSMD12/B	6
GO:00096	response to vi	12/272	323/1867(	0.002882	0.072829	0.062824	TRIM56/F2	12
GO:00425	positive regula	3/272	20/18670	0.002902	0.072829	0.062824	THBS1/CLE	3
GO:00517(	nitric-oxide sy	3/272	20/18670	0.002902	0.072829	0.062824	STAT1/TLR	3
GO:00517(	regulation of r	3/272	20/18670	0.002902	0.072829	0.062824	STAT1/TLR	3
GO:00097	response to h	9/272	202/1867(	0.00291	0.072829	0.062824	PTGS2/PFK	9
GO:01500	regulation of r	4/272	41/18670	0.002921	0.072829	0.062824	PTGS2/LRF	4
GO:00480	regulation of r	6/272	98/18670	0.003113	0.076228	0.065756	RBM7/SRP	6

GO:00022!	T cell differen!	5/272	68/18670	0.003122	0.076228	0.065756	CLEC4D/IL4	5
GO:00463	glucose impor	5/272	68/18670	0.003122	0.076228	0.065756	CLTCL1/HK	5
GO:00307	ovulation	3/272	21/18670	0.003349	0.079181	0.068303	PTGS2/IL4I	3
GO:00519(	regulation of r	3/272	21/18670	0.003349	0.079181	0.068303	PPP2R3C/I	3
GO:19002	positive regul	3/272	21/18670	0.003349	0.079181	0.068303	MME/EIF2	3
GO:19044(	positive regul	3/272	21/18670	0.003349	0.079181	0.068303	IFNGR1/OI	3
GO:00427	signal transdu	7/272	133/1867(	0.003379	0.079181	0.068303	ARID3A/G	7
GO:00510!	regulation of I	13/272	373/1867(	0.003387	0.079181	0.068303	TXN/AKTIP	13
GO:00342	response to m	9/272	207/1867(	0.003422	0.079181	0.068303	PTGS2/PFK	9
GO:00705!	response to in	9/272	207/1867(	0.003422	0.079181	0.068303	RPS6KA5/N	9
GO:00507	regulation of i	13/272	374/1867(	0.003465	0.079642	0.068701	PTGS2/CLE	13
GO:00434	regulation of I	7/272	135/1867(	0.00367	0.08381	0.072297	RBM7/SRP	7
GO:00096	response to m	9/272	210/1867(	0.003762	0.084803	0.073153	KIAA0319/	9
GO:00975	myeloid leuko	9/272	210/1867(	0.003762	0.084803	0.073153	THBS1/CAI	9
GO:00108	positive regul	4/272	44/18670	0.003785	0.084803	0.073153	CLTCL1/IRS	4
GO:00506	regulation of	5/272	73/18670	0.004243	0.091655	0.079064	STAT1/IFIT	5
GO:00724(	signal transdu	5/272	73/18670	0.004243	0.091655	0.079064	ARID3A/G	5
GO:00724	signal transdu	5/272	73/18670	0.004243	0.091655	0.079064	ARID3A/G	5
GO:00508!	cognition	11/272	296/1867(	0.004246	0.091655	0.079064	PTGS2/MN	11
GO:00450	positive regul	9/272	214/1867(	0.004255	0.091655	0.079064	RPS6KA5/I	9
GO:19037(	regulation of I	15/272	475/1867(	0.004295	0.091655	0.079064	PPP2R3C/T	15
GO:00341	positive regul	3/272	23/18670	0.004365	0.091655	0.079064	F2RL1/TLR	3
GO:00515	negative regul	3/272	23/18670	0.004365	0.091655	0.079064	RGS2/RAP	3
GO:00518	mitochondrial	3/272	23/18670	0.004365	0.091655	0.079064	PPP2R3C/I	3
GO:00711!	negative regul	3/272	23/18670	0.004365	0.091655	0.079064	MDM4/RP	3
GO:00028	positive regul	6/272	105/1867(	0.004376	0.091655	0.079064	EXOSC3/B	6
GO:00326	regulation of i	5/272	74/18670	0.004497	0.093097	0.080308	F2RL1/CLE	5
GO:00723!	signal transdu	5/272	74/18670	0.004497	0.093097	0.080308	ARID3A/G	5
GO:00510!	regulation of I	14/272	432/1867(	0.004572	0.094103	0.081175	RPS6KA5/N	14
GO:00433!	regulation of	9/272	217/1867(	0.004657	0.095301	0.082209	AKTIP/NKX	9
GO:00303	DNA damage	6/272	107/1867(	0.004797	0.097333	0.083962	ARID3A/G	6
GO:00713	cellular respo	8/272	179/1867(	0.004812	0.097333	0.083962	RPS6KA5/N	8
GO:00022	lymphocyte a	8/272	181/1867(	0.00514	0.103383	0.08918	F2RL1/CLE	8
GO:00450	regulation of i	11/272	305/1867(	0.005298	0.105953	0.091398	RPS6KA5/I	11
GO:00439(	regulation of i	9/272	222/1867(	0.005393	0.107123	0.092407	POLR2J/F2	9
GO:00000	DNA damage	7/272	145/1867(	0.005417	0.107123	0.092407	ARID3A/G	7
GO:00380(	NIK/NF-kappa	8/272	183/1867(	0.005485	0.107873	0.093054	PSMD12/E	8
GO:20004	regulation of i	3/272	25/18670	0.005549	0.107986	0.093151	F2RL1/TLR	3
GO:19900!	cellular respo	4/272	49/18670	0.005582	0.107986	0.093151	RAPGEF1/I	4
GO:20012	positive regul	4/272	49/18670	0.005582	0.107986	0.093151	PAK2/THB	4
GO:00108	regulation of	5/272	78/18670	0.005624	0.108198	0.093334	CLTCL1/HK	5
GO:00301!	regulation of I	5/272	79/18670	0.005934	0.112033	0.096642	PLAU/F2RI	5
GO:00712(	cellular respo	5/272	79/18670	0.005934	0.112033	0.096642	PTGS2/GA	5
GO:00723	signal transdu	10/272	267/1867(	0.005955	0.112033	0.096642	ARID3A/T	10
GO:00002(	protein polyu	11/272	310/1867(	0.005966	0.112033	0.096642	WSB1/TRI	11
GO:00986!	regulation of	6/272	112/1867(	0.005981	0.112033	0.096642	PFN2/CDK	6
GO:00086	carbohydrate	7/272	148/1867(	0.006046	0.11205	0.096657	CLTCL1/HK	7
GO:00610	regulation of	7/272	148/1867(	0.006046	0.11205	0.096657	PLAU/F2RI	7

GO:00022:	positive regul:	3/272	26/18670	0.006206	0.112581	0.097115	STAT1/EIF:	3
GO:19001:	regulation of r	3/272	26/18670	0.006206	0.112581	0.097115	NKX3-1/B2	3
GO:19039!	regulation of a	3/272	26/18670	0.006206	0.112581	0.097115	THBS1/RG:	3
GO:00219{	cerebral corte	6/272	113/1867(	0.006242	0.112581	0.097115	TACC3/CDI	6
GO:19000	regulation of l	5/272	80/18670	0.006256	0.112581	0.097115	PLAU/F2RL	5
GO:00072	I-kappaB kina:	10/272	269/1867(	0.006265	0.112581	0.097115	F2RL1/STA	10
GO:00016(	response to h	12/272	359/1867(	0.006622	0.118385	0.102122	PLAU/NKX	12
GO:00109:	regulation of r	15/272	499/1867(	0.006684	0.118902	0.102567	KIAA0319/	15
GO:20012:	negative regul	9/272	230/1867(	0.006753	0.11952	0.1031	PTGS2/THI	9
GO:19900{	response to n	4/272	52/18670	0.006895	0.120312	0.103784	RAPGEF1/I	4
GO:00430:	positive regul:	3/272	27/18670	0.006907	0.120312	0.103784	THBS1/IL4I	3
GO:00109	negative regul	12/272	361/1867(	0.006908	0.120312	0.103784	ARID3A/PS	12
GO:00326:	interleukin-8	5/272	82/18670	0.006937	0.120312	0.103784	F2RL1/CLE	5
GO:00342!	positive regul:	7/272	152/1867(	0.006967	0.120312	0.103784	CDC123/IF	7
GO:00436(	regulation of	6/272	117/1867(	0.007366	0.125503	0.108261	PPP2R3A/I	6
GO:00001{	activation of M	4/272	53/18670	0.007374	0.125503	0.108261	RAPGEF1/I	4
GO:00301!	negative regul	4/272	53/18670	0.007374	0.125503	0.108261	PLAU/THB:	4
GO:00427:	fibrinolysis	3/272	28/18670	0.007654	0.129246	0.111491	PLAU/THB:	3
GO:00508:	regulation of	5/272	84/18670	0.007667	0.129246	0.111491	PLAU/F2RL	5
GO:19000	negative regul	4/272	54/18670	0.007875	0.132117	0.113967	PLAU/THB:	4
GO:00315:	DNA integrity	7/272	157/1867(	0.008261	0.13469	0.116187	ARID3A/G/	7
GO:00432:	negative regul	7/272	157/1867(	0.008261	0.13469	0.116187	PTGS2/THI	7
GO:00300!	myeloid cell d	13/272	416/1867(	0.008269	0.13469	0.116187	F2RL1/THE	13
GO:00362!	response to d	12/272	370/1867(	0.00832	0.13469	0.116187	PLAU/NKX	12
GO:00421:	T cell activatic	14/272	464/1867(	0.008383	0.13469	0.116187	PAK2/F2RL	14
GO:00097	response to gl	8/272	197/1867(	0.00842	0.13469	0.116187	PFKFB2/TH	8
GO:00105:	positive regul:	3/272	29/18670	0.008447	0.13469	0.116187	SULF2/PTG	3
GO:00356(	TRIF-depende	3/272	29/18670	0.008447	0.13469	0.116187	UBE2D3/L'	3
GO:00482	sperm capacit	3/272	29/18670	0.008447	0.13469	0.116187	DLD/ROPN	3
GO:00702:	negative regul	3/272	29/18670	0.008447	0.13469	0.116187	FADD/HIF1	3
GO:00726(	interleukin-8 s	3/272	29/18670	0.008447	0.13469	0.116187	F2RL1/TLR	3
GO:00165:	protein deubi	10/272	283/1867(	0.008807	0.136783	0.117992	USP15/USI	10
GO:00109!	regulation of t	2/272	10/18670	0.00881	0.136783	0.117992	EIF2AK2/EI	2
GO:00341:	regulation of t	2/272	10/18670	0.00881	0.136783	0.117992	F2RL1/TLR	2
GO:00519!	negative regul	2/272	10/18670	0.00881	0.136783	0.117992	RGS2/ABA	2
GO:00711(	response to in	2/272	10/18670	0.00881	0.136783	0.117992	STAT1/IL9I	2
GO:19001:	positive regul:	2/272	10/18670	0.00881	0.136783	0.117992	B2M/MMI	2
GO:00434:	regulation of	5/272	87/18670	0.008861	0.136977	0.11816	PFKFB2/R/	5
GO:00197:	antimicrobial	6/272	122/1867(	0.008968	0.138024	0.119063	PPP2R3C/(	6
GO:00380!	Fc receptor si	9/272	241/1867(	0.009035	0.138453	0.119433	PAK2/CLE(	9
GO:00028:	regulation of	7/272	160/1867(	0.009118	0.139125	0.120012	IL4R/EXOS	7
GO:00017{	B cell homeos	3/272	30/18670	0.009286	0.139877	0.120661	PPP2R3C/T	3
GO:00217:	olfactory bulb	3/272	30/18670	0.009286	0.139877	0.120661	EOMES/LR	3
GO:00506!	regulation of	3/272	30/18670	0.009286	0.139877	0.120661	PAK2/EIF2	3
GO:00435:	positive regul:	4/272	57/18670	0.009509	0.14167	0.122208	SRPK2/NQ	4
GO:00508:	negative regul	4/272	57/18670	0.009509	0.14167	0.122208	PLAU/THB:	4
GO:00469	carboxylic acid	11/272	331/1867(	0.009525	0.14167	0.122208	ACSL1/THE	11
GO:00066:	protein target	8/272	202/1867(	0.009711	0.143839	0.124079	RPS28/RPS	8

GO:00158	organic acid tr	11/272	333/1867	0.009934	0.145778	0.125752	ACSL1/THE	11
GO:00308	negative regul	4/272	58/18670	0.010099	0.145778	0.125752	PFN2/TMS	4
GO:00316	lipopolysaccha	4/272	58/18670	0.010099	0.145778	0.125752	LY96/TLR2	4
GO:00971	intrinsic apopt	10/272	289/1867	0.010111	0.145778	0.125752	NKX3-1/PT	10
GO:00512	positive regul	11/272	334/1867	0.010144	0.145778	0.125752	PAK2/PPP2	11
GO:00066	leukotriene m	3/272	31/18670	0.010172	0.145778	0.125752	CYP4F3/TL	3
GO:00219	olfactory lobe	3/272	31/18670	0.010172	0.145778	0.125752	EOMES/LR	3
GO:00519	regulation of a	3/272	31/18670	0.010172	0.145778	0.125752	RGS2/RAB	3
GO:00001	activation of M	2/272	11/18670	0.010665	0.150409	0.129746	GADD45G/	2
GO:00316	positive regul	2/272	11/18670	0.010665	0.150409	0.129746	PTGS2/AB	2
GO:00456	regulation of T	2/272	11/18670	0.010665	0.150409	0.129746	IL4R/IL18	2
GO:00487	regulation of s	2/272	11/18670	0.010665	0.150409	0.129746	HDAC4/LM	2
GO:00487	skeletal muscl	3/272	32/18670	0.011105	0.155381	0.134035	BIN3/HDA	3
GO:19044	regulation of t	3/272	32/18670	0.011105	0.155381	0.134035	IFNGR1/OI	3
GO:00109	negative regul	9/272	250/1867	0.011302	0.157512	0.135873	RPS6KA3/	9
GO:00507	regulation of v	8/272	208/1867	0.011449	0.15879	0.136976	POLR2J/ST	8
GO:00320	positive regul	5/272	93/18670	0.011622	0.15879	0.136976	AKTIP/B2M	5
GO:00019	cell killing	7/272	168/1867	0.011719	0.15879	0.136976	F2RL1/CAN	7
GO:19059	negative regul	4/272	61/18670	0.012009	0.15879	0.136976	THBS1/IRS	4
GO:20012	positive regul	4/272	61/18670	0.012009	0.15879	0.136976	NKX3-1/S1	4
GO:00486	regulation of s	7/272	169/1867	0.012078	0.15879	0.136976	PTGS2/THI	7
GO:00511	negative regul	7/272	169/1867	0.012078	0.15879	0.136976	NKX3-1/IFI	7
GO:00027	MyD88-indep	3/272	33/18670	0.012086	0.15879	0.136976	UBE2D3/L	3
GO:00100	response to ir	3/272	33/18670	0.012086	0.15879	0.136976	B2M/HIF1	3
GO:19012	positive regul	5/272	94/18670	0.012133	0.15879	0.136976	SRPK2/NQ	5
GO:00181	peptidyl-serin	10/272	299/1867	0.012607	0.15879	0.136976	TXN/RPS6	10
GO:00706	protein modif	10/272	299/1867	0.012607	0.15879	0.136976	USP15/USI	10
GO:00027	positive regul	5/272	95/18670	0.012659	0.15879	0.136976	F2RL1/IL4	5
GO:00311	SCF-depender	5/272	95/18670	0.012659	0.15879	0.136976	PSMD12/A	5
GO:00421	B cell prolifer	5/272	95/18670	0.012659	0.15879	0.136976	MNDA/TN	5
GO:00440	regulation of a	5/272	95/18670	0.012659	0.15879	0.136976	THBS1/RG	5
GO:00450	regulation of v	5/272	95/18670	0.012659	0.15879	0.136976	SRPK2/IFIT	5
GO:00160	detection of b	2/272	12/18670	0.012675	0.15879	0.136976	TLR1/TLR2	2
GO:00381	nerve growth	2/272	12/18670	0.012675	0.15879	0.136976	RAPGEF1/I	2
GO:00515	positive regul	2/272	12/18670	0.012675	0.15879	0.136976	MMP9/M	2
GO:00518	killing by host	2/272	12/18670	0.012675	0.15879	0.136976	F2RL1/CAN	2
GO:00602	positive regul	2/272	12/18670	0.012675	0.15879	0.136976	TOB1/TNR	2
GO:00603	regulation of I	2/272	12/18670	0.012675	0.15879	0.136976	RAPGEF1/I	2
GO:00704	leukocyte agg	2/272	12/18670	0.012675	0.15879	0.136976	S100A9/S1	2
GO:00901	Golgi ribbon f	2/272	12/18670	0.012675	0.15879	0.136976	GOLPH3/G	2
GO:00022	lymphocyte h	4/272	62/18670	0.012694	0.15879	0.136976	PPP2R3C/T	4
GO:00108	regulation of r	4/272	62/18670	0.012694	0.15879	0.136976	SCGB3A1/I	4
GO:00316	killing of cells	4/272	62/18670	0.012694	0.15879	0.136976	F2RL1/CAN	4
GO:00702	necrotic cell d	4/272	62/18670	0.012694	0.15879	0.136976	PYGL/LY96	4
GO:00486	smooth muscl	7/272	171/1867	0.012821	0.159807	0.137853	PTGS2/THI	7
GO:00076	learning or me	9/272	256/1867	0.013034	0.160099	0.138105	PTGS2/MN	9
GO:00105	regulation of v	3/272	34/18670	0.013115	0.160099	0.138105	SULF2/PTG	3
GO:00321	activation of p	3/272	34/18670	0.013115	0.160099	0.138105	TXN/IL18/	3

GO:00425:	tumor necrosi	3/272	34/18670	0.013115	0.160099	0.138105	THBS1/CLE	3
GO:00425:	regulation of t	3/272	34/18670	0.013115	0.160099	0.138105	THBS1/CLE	3
GO:00622(	positive regula	3/272	34/18670	0.013115	0.160099	0.138105	F2RL1/TLR	3
GO:00704{	response to o:	12/272	394/1867(	0.013204	0.160621	0.138556	PLAU/NKX:	12
GO:00159(	fatty acid tran	5/272	97/18670	0.013757	0.166211	0.143377	ACSL1/THE	5
GO:00447:	mitotic DNA d	5/272	97/18670	0.013757	0.166211	0.143377	ARID3A/G/	5
GO:00603:	cell chemotax	10/272	304/1867(	0.014017	0.167334	0.144346	F2RL1/THE	10
GO:00465:	positive regula	4/272	64/18670	0.014137	0.167334	0.144346	F2RL1/RAF	4
GO:00157:	organic anion	14/272	495/1867(	0.014139	0.167334	0.144346	ABCC5/AC:	14
GO:00149(	myotube cell (	3/272	35/18670	0.014193	0.167334	0.144346	BIN3/HDA(	3
GO:00341<	toll-like recep	3/272	35/18670	0.014193	0.167334	0.144346	F2RL1/LY9	3
GO:00450:	positive regula	3/272	35/18670	0.014193	0.167334	0.144346	SRPK2/IFIT	3
GO:19907:	tumor necrosi	3/272	35/18670	0.014193	0.167334	0.144346	IFNGR1/OI	3
GO:19038:	organic acid tr	6/272	135/1867(	0.014275	0.167334	0.144346	THBS1/RG:	6
GO:19050:	carboxylic aci(	6/272	135/1867(	0.014275	0.167334	0.144346	THBS1/RG:	6
GO:20012<	negative regul	5/272	98/18670	0.014329	0.167411	0.144413	PTGS2/PL/	5
GO:00027(	positive regula	6/272	136/1867(	0.014758	0.168952	0.145742	F2RL1/IL4F	6
GO:00300:	lymphocyte di	11/272	353/1867(	0.014818	0.168952	0.145742	PPP2R3C/(	11
GO:00067(	estrogen bios	2/272	13/18670	0.014837	0.168952	0.145742	HSD17B11	2
GO:00316:	regulation of I	2/272	13/18670	0.014837	0.168952	0.145742	PTGS2/AB/	2
GO:00343<	response to ty	5/272	99/18670	0.014917	0.168952	0.145742	TRIM56/IF	5
GO:00104(	negative regul	9/272	262/1867(	0.014956	0.168952	0.145742	RPS6KA3/F	9
GO:00026:	positive regula	8/272	219/1867(	0.015216	0.168952	0.145742	F2RL1/RAF	8
GO:00516:	maintenance (	8/272	219/1867(	0.015216	0.168952	0.145742	TXN/GOLP	8
GO:00506{	regulation of r	6/272	137/1867(	0.015252	0.168952	0.145742	RBM7/SRP	6
GO:00027:	MyD88-deper	3/272	36/18670	0.01532	0.168952	0.145742	LY96/TLR1	3
GO:00105:	vascular endo	3/272	36/18670	0.01532	0.168952	0.145742	SULF2/PTG	3
GO:00107<	macrophage c	3/272	36/18670	0.01532	0.168952	0.145742	STAT1/NFK	3
GO:00400:	regulation of f	3/272	36/18670	0.01532	0.168952	0.145742	SULF2/THE	3
GO:00425:	superoxide an	3/272	36/18670	0.01532	0.168952	0.145742	F2RL1/CLE	3
GO:00716:	regulation of t	3/272	36/18670	0.01532	0.168952	0.145742	PTGS2/THI	3
GO:00900:	foam cell diffe	3/272	36/18670	0.01532	0.168952	0.145742	STAT1/NFK	3
GO:00903:	regulation of s	3/272	36/18670	0.01532	0.168952	0.145742	F2RL1/CLE	3
GO:00972<	amyloid-beta	3/272	36/18670	0.01532	0.168952	0.145742	IFNGR1/M	3
GO:00015(	regulation of r	11/272	355/1867(	0.015391	0.169206	0.145961	PTGS2/UPI	11
GO:00028:	positive regula	5/272	100/1867(	0.015521	0.169584	0.146287	EXOSC3/B:	5
GO:00704:	interleukin-1-r	5/272	100/1867(	0.015521	0.169584	0.146287	RPS6KA5/F	5
GO:00900:	negative regul	7/272	178/1867(	0.015678	0.170767	0.147307	RAPGEF1/I	7
GO:00080(	regulation of a	7/272	179/1867(	0.01612	0.173449	0.149621	F2RL1/PFN	7
GO:00108:	regulation of r	7/272	179/1867(	0.01612	0.173449	0.149621	UBE2D3/P	7
GO:00510:	positive regula	7/272	179/1867(	0.01612	0.173449	0.149621	TXN/AKTIP	7
GO:19030:	regulation of r	7/272	179/1867(	0.01612	0.173449	0.149621	PLAU/F2RL	7
GO:00353(	regulation of p	6/272	139/1867(	0.016273	0.173607	0.149758	PPP2R3A/E	6
GO:00515{	regulation of r	6/272	139/1867(	0.016273	0.173607	0.149758	PFN2/RGS:	6
GO:00458(	negative regul	11/272	358/1867(	0.016282	0.173607	0.149758	RPS6KA3/F	11
GO:00457:	respiratory bu	3/272	37/18670	0.016496	0.174839	0.15082	CLEC7A/C/	3
GO:00506:	regulation of (	3/272	37/18670	0.016496	0.174839	0.15082	STAT1/EIF:	3
GO:00308:	regulation of a	7/272	180/1867(	0.016571	0.175108	0.151052	F2RL1/PFN	7

GO:00252	leukocyte mig	2/272	14/18670	0.017146	0.176954	0.152644	S100A9/S1	2
GO:00343	NAD biosynth	2/272	14/18670	0.017146	0.176954	0.152644	PTGS2/SLC	2
GO:00435	regulation of t	2/272	14/18670	0.017146	0.176954	0.152644	EIF2AK2/EI	2
GO:00450	T-helper 2 cell	2/272	14/18670	0.017146	0.176954	0.152644	IL4R/IL18	2
GO:00515	regulation of I	2/272	14/18670	0.017146	0.176954	0.152644	MMP9/MA	2
GO:00517	positive regula	2/272	14/18670	0.017146	0.176954	0.152644	F2RL1/CLE	2
GO:00602	regulation of r	2/272	14/18670	0.017146	0.176954	0.152644	TOB1/TNR	2
GO:19001	negative regul	2/272	14/18670	0.017146	0.176954	0.152644	NKX3-1/B2	2
GO:00313	positive regula	4/272	68/18670	0.017326	0.178296	0.153802	F2RL1/CLE	4
GO:00320	negative regul	5/272	103/1867(	0.017431	0.178337	0.153837	NKX3-1/IFI	5
GO:00347	negative regul	5/272	103/1867(	0.017431	0.178337	0.153837	THBS1/RG	5
GO:00512	maintenance	10/272	315/1867(	0.01753	0.178837	0.154269	TXN/GOLP	10
GO:00108	positive regula	3/272	38/18670	0.017721	0.179751	0.155057	SCGB3A1/I	3
GO:00716	transforming	3/272	38/18670	0.017721	0.179751	0.155057	PTGS2/THI	3
GO:00198	antigen proce	8/272	226/1867(	0.018038	0.182445	0.157381	THBS1/PSN	8
GO:00159	long-chain fat	4/272	69/18670	0.018187	0.183429	0.15823	ACSL1/THE	4
GO:00026	regulation of i	13/272	462/1867(	0.018379	0.184834	0.159442	F2RL1/RAF	13
GO:00508	regulation of I	7/272	184/1867(	0.018464	0.185168	0.15973	PPP2R3C/N	7
GO:19037	positive regula	7/272	185/1867(	0.018961	0.189148	0.163163	PPP2R3C/S	7
GO:00713	cellular respo	6/272	144/1867(	0.019032	0.189148	0.163163	CLEC7A/PT	6
GO:00341	regulation of t	4/272	70/18670	0.019075	0.189148	0.163163	F2RL1/LY9	4
GO:00519	regulation of s	4/272	70/18670	0.019075	0.189148	0.163163	PTGS2/RAI	4
GO:00447	mitotic DNA ir	5/272	106/1867(	0.019489	0.18976	0.163692	ARID3A/GA	5
GO:00024	antigen proce	2/272	15/18670	0.019595	0.18976	0.163692	B2M/TAP2	2
GO:00170	protein nitros	2/272	15/18670	0.019595	0.18976	0.163692	S100A9/S1	2
GO:00181	peptidyl-cyste	2/272	15/18670	0.019595	0.18976	0.163692	S100A9/S1	2
GO:00198	antigen proce	2/272	15/18670	0.019595	0.18976	0.163692	B2M/TAP2	2
GO:00324	Rap protein si	2/272	15/18670	0.019595	0.18976	0.163692	RAPGEF1/I	2
GO:00454	positive regula	2/272	15/18670	0.019595	0.18976	0.163692	CLEC7A/TL	2
GO:00518	killing of cells	2/272	15/18670	0.019595	0.18976	0.163692	F2RL1/CAN	2
GO:00028	regulation of a	6/272	145/1867(	0.019619	0.18976	0.163692	IL4R/EXOS	6
GO:00064	protein depho	10/272	321/1867(	0.019701	0.190032	0.163926	PPP2R3A/I	10
GO:00069	phagocytosis	11/272	369/1867(	0.019881	0.19125	0.164976	F2RL1/SIRI	11
GO:00510	positive regula	4/272	71/18670	0.019989	0.191766	0.165422	F2RL1/RAF	4
GO:00182	peptidyl-serin	10/272	322/1867(	0.020081	0.192129	0.165734	TXN/RPS6I	10
GO:00325	regulation of	11/272	370/1867(	0.020236	0.193083	0.166558	IFRD1/F2R	11
GO:00313	negative regul	7/272	188/1867(	0.020505	0.19513	0.168323	KIAA0319/	7
GO:00320	positive regula	4/272	72/18670	0.02093	0.197052	0.169981	PFKFB2/HI	4
GO:00322	negative regul	4/272	72/18670	0.02093	0.197052	0.169981	PFN2/TMS	4
GO:00518	regulation of r	4/272	72/18670	0.02093	0.197052	0.169981	PPP2R3C/I	4
GO:19020	regulation of I	4/272	72/18670	0.02093	0.197052	0.169981	PSMD12/E	4
GO:00163	dendrite deve	8/272	233/1867(	0.021219	0.199241	0.171869	KIAA0319/	8
GO:00607	regulation of r	7/272	190/1867(	0.021582	0.1993	0.171921	F2RL1/IFN	7
GO:00308	positive regula	3/272	41/18670	0.021693	0.1993	0.171921	TNFSF13B/	3
GO:00610	establishment	3/272	41/18670	0.021693	0.1993	0.171921	F2RL1/RAF	3
GO:00024	immune respo	13/272	473/1867(	0.0218	0.1993	0.171921	RPS6KA5/I	13
GO:00027	immune respo	13/272	473/1867(	0.0218	0.1993	0.171921	RPS6KA5/I	13
GO:00068	superoxide m	4/272	73/18670	0.021897	0.1993	0.171921	F2RL1/CLE	4

GO:00618[illegible]	antimicrobial [illegible]	4/272	73/18670	0.021897	0.1993	0.171921	CAMP/S10	4
GO:00196[illegible]	ribose phosph	12/272	424/1867[illegible]	0.022087	0.1993	0.171921	DLD/PDE7[illegible]	12
GO:19039[illegible]	regulation of [illegible]	6/272	149/1867[illegible]	0.022094	0.1993	0.171921	SRPK2/IFIT	6
GO:00024[illegible]	antigen proce[illegible]	2/272	16/18670	0.022182	0.1993	0.171921	B2M/TAP2	2
GO:00028[illegible]	positive regul[illegible]	2/272	16/18670	0.022182	0.1993	0.171921	IL4R/IL18	2
GO:00193[illegible]	lipoxygenase [illegible]	2/272	16/18670	0.022182	0.1993	0.171921	PTGS2/AL[illegible]	2
GO:00305[illegible]	nuclear body [illegible]	2/272	16/18670	0.022182	0.1993	0.171921	SRPK2/SU[illegible]	2
GO:00459[illegible]	negative regul	2/272	16/18670	0.022182	0.1993	0.171921	PTGS2/RG[illegible]	2
GO:00512[illegible]	sequestering [illegible]	2/272	16/18670	0.022182	0.1993	0.171921	S100A9/S1	2
GO:00514[illegible]	negative regul	2/272	16/18670	0.022182	0.1993	0.171921	RPL23/DT[illegible]	2
GO:19020[illegible]	positive regul[illegible]	2/272	16/18670	0.022182	0.1993	0.171921	IFNGR1/PI[illegible]	2
GO:19018[illegible]	regulation of [illegible]	7/272	192/1867[illegible]	0.022698	0.203314	0.175383	NTNG2/RA	7
GO:00060[illegible]	pyruvate met[illegible]	6/272	150/1867[illegible]	0.022744	0.203314	0.175383	DLD/PFKF[illegible]	6
GO:00511[illegible]	cofactor trans	3/272	42/18670	0.023116	0.206126	0.177809	LMBRD1/F	3
GO:00995[illegible]	synaptic vesic[illegible]	7/272	193/1867[illegible]	0.023271	0.206981	0.178546	PFN2/CDK[illegible]	7
GO:00712[illegible]	cellular respo[illegible]	10/272	331/1867[illegible]	0.023748	0.21017	0.181298	RGR/PTGS[illegible]	10
GO:01040[illegible]	cellular respo[illegible]	10/272	331/1867[illegible]	0.023748	0.21017	0.181298	RGR/PTGS[illegible]	10
GO:00149[illegible]	myotube diffe	5/272	112/1867[illegible]	0.024072	0.211392	0.182351	SCGB3A1/[illegible]	5
GO:00016[illegible]	cellular glucos	6/272	152/1867[illegible]	0.024082	0.211392	0.182351	HK2/PTPR[illegible]	6
GO:00901[illegible]	establishment	10/272	332/1867[illegible]	0.024183	0.211392	0.182351	RPS28/RP[illegible]	10
GO:00513[illegible]	negative regul	9/272	285/1867[illegible]	0.024289	0.211392	0.182351	PAK2/SH3[illegible]	9
GO:00309[illegible]	forebrain dev[illegible]	11/272	381/1867[illegible]	0.024446	0.211392	0.182351	TACC3/B2[illegible]	11
GO:00608[illegible]	regulation of [illegible]	9/272	286/1867[illegible]	0.024771	0.211392	0.182351	SULF2/RA[illegible]	9
GO:00302[illegible]	T cell differen[illegible]	8/272	240/1867[illegible]	0.024778	0.211392	0.182351	CLEC4D/IL[illegible]	8
GO:00060[illegible]	glycolytic pro[illegible]	5/272	113/1867[illegible]	0.024897	0.211392	0.182351	PFKFB2/H[illegible]	5
GO:00326[illegible]	interferon-ga[illegible]	5/272	113/1867[illegible]	0.024897	0.211392	0.182351	F2RL1/CLE	5
GO:00060[illegible]	lactate metab	2/272	17/18670	0.024901	0.211392	0.182351	PFKFB2/HI	2
GO:00316[illegible]	heat generati[illegible]	2/272	17/18670	0.024901	0.211392	0.182351	PTGS2/AB[illegible]	2
GO:00517[illegible]	regulation of [illegible]	2/272	17/18670	0.024901	0.211392	0.182351	F2RL1/CLE	2
GO:00602[illegible]	regulation of [illegible]	2/272	17/18670	0.024901	0.211392	0.182351	CLEC7A/C[illegible]	2
GO:00718[illegible]	mitotic cell cy[illegible]	2/272	17/18670	0.024901	0.211392	0.182351	NKX3-1/G[illegible]	2
GO:00901[illegible]	negative regul	2/272	17/18670	0.024901	0.211392	0.182351	STAT1/MM	2
GO:19026[illegible]	regulation of [illegible]	2/272	17/18670	0.024901	0.211392	0.182351	RAP1A/NP	2
GO:20012[illegible]	regulation of [illegible]	2/272	17/18670	0.024901	0.211392	0.182351	PLAUR/MN	2
GO:00610[illegible]	negative regul	4/272	76/18670	0.024961	0.211395	0.182355	PLAU/THB[illegible]	4
GO:00719[illegible]	positive regul[illegible]	10/272	334/1867[illegible]	0.025072	0.211822	0.182722	GADD45G/	10
GO:00067[illegible]	ATP generatio	5/272	114/1867[illegible]	0.02574	0.216086	0.1864	PFKFB2/H[illegible]	5
GO:00061[illegible]	regulation of [illegible]	4/272	77/18670	0.026037	0.216086	0.1864	PFKFB2/R[illegible]	4
GO:00165[illegible]	histone deace	4/272	77/18670	0.026037	0.216086	0.1864	SAP30/JDP	4
GO:00614[illegible]	regulation of [illegible]	4/272	77/18670	0.026037	0.216086	0.1864	PSMD12/P	4
GO:00512[illegible]	regulation of [illegible]	13/272	485/1867[illegible]	0.026053	0.216086	0.1864	PAK2/PPP[illegible]	13
GO:00302[illegible]	macrophage c	3/272	44/18670	0.026111	0.216086	0.1864	FADD/TLR[illegible]	3
GO:00702[illegible]	necroptotic p[illegible]	3/272	44/18670	0.026111	0.216086	0.1864	PYGL/LY96	3
GO:00716[illegible]	regulation of [illegible]	3/272	44/18670	0.026111	0.216086	0.1864	THBS1/CA[illegible]	3
GO:00109[illegible]	negative regul	6/272	155/1867[illegible]	0.026187	0.216086	0.1864	KIAA0319/	6
GO:20012[illegible]	regulation of [illegible]	6/272	155/1867[illegible]	0.026187	0.216086	0.1864	PAK2/THB[illegible]	6
GO:00160[illegible]	carbohydrate	7/272	198/1867[illegible]	0.026284	0.216381	0.186655	PFKFB2/PY	7
GO:00610[illegible]	regulation of [illegible]	7/272	199/1867[illegible]	0.026917	0.221079	0.190708	TOB1/PSM	7

GO:19033:	positive regula	4/272	78/18670	0.027141	0.221739	0.191277	TOB1/RBM	4
GO:20003(	regulation of s	4/272	78/18670	0.027141	0.221739	0.191277	PFN2/RAB3	4
GO:00108:	positive regula	5/272	116/18670	0.027482	0.221739	0.191277	UBE2D3/P	5
GO:00434(	regulation of g	6/272	157/1867(	0.027656	0.221739	0.191277	PFKFB2/RA	6
GO:19002:	regulation of l	3/272	45/18670	0.027683	0.221739	0.191277	MME/EIF2	3
GO:00310(	positive regula	2/272	18/18670	0.027749	0.221739	0.191277	JDP2/LRRK	2
GO:00420!	interferon-gar	2/272	18/18670	0.027749	0.221739	0.191277	CLEC7A/IL1	2
GO:00519!	positive regula	2/272	18/18670	0.027749	0.221739	0.191277	RAB3GAP1	2
GO:00702:	negative regul	2/272	18/18670	0.027749	0.221739	0.191277	FADD/HIF1	2
GO:00901!	regulation of c	2/272	18/18670	0.027749	0.221739	0.191277	F2RL1/IL4F	2
GO:01500:	positive regula	2/272	18/18670	0.027749	0.221739	0.191277	LRRK2/IL18	2
GO:19001!	positive regula	2/272	18/18670	0.027749	0.221739	0.191277	TOB1/TNR	2
GO:00488:	homeostasis c	8/272	246/1867(	0.028145	0.224397	0.19357	PPP2R3C/S	8
GO:00511!	regulation of s	5/272	117/1867(	0.02838	0.224994	0.194085	SCGB3A1/I	5
GO:19012:	regulation of I	5/272	117/1867(	0.02838	0.224994	0.194085	EIF2AK2/P	5
GO:00164{	cytosolic trans	6/272	158/1867(	0.02841	0.224994	0.194085	AKTIP/CLT(	6
GO:00000{	G2/M transitic	8/272	247/1867(	0.028735	0.22706	0.195867	PSMD12/C	8
GO:00516·	mitochondrioı	3/272	46/18670	0.029304	0.230452	0.198793	KIF1B/HIF1	3
GO:19019!	negative regul	8/272	248/1867(	0.029334	0.230452	0.198793	ARID3A/PS	8
GO:00022:	response to m	10/272	343/1867(	0.02936	0.230452	0.198793	RPS6KA3/F	10
GO:00215:	telencephalon	8/272	249/1867(	0.029942	0.232801	0.20082	TACC3/CDI	8
GO:19033(	regulation of r	6/272	160/1867(	0.02996	0.232801	0.20082	F2RL1/IL4F	6
GO:19019!	regulation of r	12/272	444/1867(	0.030081	0.232801	0.20082	ARID3A/PS	12
GO:00332:	regulation of c	4/272	81/18670	0.030617	0.232801	0.20082	PSMD12/P	4
GO:00000:	ribosomal sma	2/272	19/18670	0.03072	0.232801	0.20082	RPS28/RPS	2
GO:00019!	regulation of s	2/272	19/18670	0.03072	0.232801	0.20082	F2RL1/MM	2
GO:00025·	chronic inflam	2/272	19/18670	0.03072	0.232801	0.20082	THBS1/S10	2
GO:00027:	positive regula	2/272	19/18670	0.03072	0.232801	0.20082	B2M/IL18	2
GO:00108{	negative regul	2/272	19/18670	0.03072	0.232801	0.20082	NFKBIA/OS	2
GO:00329:	positive regula	2/272	19/18670	0.03072	0.232801	0.20082	F2RL1/CLE	2
GO:00341:	toll-like recept	2/272	19/18670	0.03072	0.232801	0.20082	F2RL1/FAD	2
GO:00353:	long-chain fat	2/272	19/18670	0.03072	0.232801	0.20082	ACSL1/HSD	2
GO:00515·	keratinocyte r	2/272	19/18670	0.03072	0.232801	0.20082	MMP9/MA	2
GO:19017·	positive regula	2/272	19/18670	0.03072	0.232801	0.20082	SCGB3A1/I	2
GO:20006!	regulation of e	2/272	19/18670	0.03072	0.232801	0.20082	STAT1/MM	2
GO:00511(	nuclear transp	10/272	346/1867(	0.030899	0.232801	0.20082	TXN/PTGS:	10
GO:00450:	G0 to G1 tran:	3/272	47/18670	0.030974	0.232801	0.20082	MDM4/EP	3
GO:00456·	regulation of e	3/272	47/18670	0.030974	0.232801	0.20082	STAT1/B2M	3
GO:00504:	amyloid-beta	3/272	47/18670	0.030974	0.232801	0.20082	IFNGR1/M	3
GO:20001(	negative regul	3/272	47/18670	0.030974	0.232801	0.20082	FADD/HIF1	3
GO:00456:	regulation of r	8/272	251/1867(	0.031183	0.23339	0.201328	THBS1/STA	8
GO:00105(	positive regula	5/272	120/1867(	0.031185	0.23339	0.201328	HK2/RAB3(	5
GO:00308:	regulation of a	6/272	162/1867(	0.031564	0.235731	0.203347	PFN2/GMF	6
GO:00302:	negative regul	4/272	82/18670	0.031831	0.236233	0.20378	TOB1/SRG	4
GO:00346·	cellular respor	4/272	82/18670	0.031831	0.236233	0.20378	PTGS2/MN	4
GO:00975{	lamellipodium	4/272	82/18670	0.031831	0.236233	0.20378	BIN3/GOLF	4
GO:00714!	cellular respor	7/272	207/1867(	0.032358	0.238053	0.20535	NKX3-1/PT	7
GO:00157:	monocarboxyl	6/272	163/1867(	0.032387	0.238053	0.20535	ACSL1/THE	6

GO:00091!	purine ribonu	11/272	399/1867(	0.032662	0.238053	0.20535	DLD/PDE7/	11
GO:00017	microglial cell	3/272	48/18670	0.032694	0.238053	0.20535	IFNGR1/TL	3
GO:0002(	leukocyte acti	3/272	48/18670	0.032694	0.238053	0.20535	IFNGR1/TL	3
GO:00027	positive regul	3/272	48/18670	0.032694	0.238053	0.20535	B2M/FADD	3
GO:00459	positive regul	3/272	48/18670	0.032694	0.238053	0.20535	PTGS2/RG!	3
GO:00603	face developn	3/272	48/18670	0.032694	0.238053	0.20535	RAB3GAP1	3
GO:00602	hematopoieti	4/272	83/18670	0.033074	0.238053	0.20535	PSMD12/E	4
GO:00506	regulation of l	7/272	208/1867(	0.033086	0.238053	0.20535	MNDA/TN	7
GO:00190	viral genome	5/272	122/1867(	0.033148	0.238053	0.20535	SRPK2/IFIT	5
GO:00460	ADP metaboli	5/272	122/1867(	0.033148	0.238053	0.20535	PFKFB2/HK	5
GO:00023	cytokine secre	2/272	20/18670	0.033811	0.238053	0.20535	F2RL1/TLR	2
GO:00100	response to m	2/272	20/18670	0.033811	0.238053	0.20535	PTGS2/LRF	2
GO:00327	positive regul	2/272	20/18670	0.033811	0.238053	0.20535	RPS6KA5/(	2
GO:00354!	cellular respo	2/272	20/18670	0.033811	0.238053	0.20535	MNDA/ST/	2
GO:00481	behavioral res	2/272	20/18670	0.033811	0.238053	0.20535	FADD/ABA	2
GO:00716	positive regul	2/272	20/18670	0.033811	0.238053	0.20535	PTGS2/THI	2
GO:00901!	chemokine se	2/272	20/18670	0.033811	0.238053	0.20535	F2RL1/IL4F	2
GO:19000	positive regul	2/272	20/18670	0.033811	0.238053	0.20535	CLEC7A/IL	2
GO:19001!	regulation of	2/272	20/18670	0.033811	0.238053	0.20535	TOB1/TNR	2
GO:19029!	positive regul	2/272	20/18670	0.033811	0.238053	0.20535	IFNGR1/PI	2
GO:19035!	positive regul	2/272	20/18670	0.033811	0.238053	0.20535	HK2/HIF1A	2
GO:00081!	actin polymer	7/272	209/1867(	0.033825	0.238053	0.20535	F2RL1/PFN	7
GO:00329	regulation of	7/272	209/1867(	0.033825	0.238053	0.20535	MNDA/TN	7
GO:00353(	regulation of	7/272	209/1867(	0.033825	0.238053	0.20535	PPP2R3A/I	7
GO:00032!	regulation of	3/272	49/18670	0.034462	0.240621	0.207565	PPP2R3C/I	3
GO:00431	negative regul	3/272	49/18670	0.034462	0.240621	0.207565	STAT1/USI	3
GO:00450	actin nucleati	3/272	49/18670	0.034462	0.240621	0.207565	GMFG/ARI	3
GO:00973(	programmed	3/272	49/18670	0.034462	0.240621	0.207565	PYGL/LY96	3
GO:00301	negative regul	7/272	210/1867(	0.034575	0.240936	0.207837	RAPGEF1/I	7
GO:00165	histone modif	12/272	454/1867(	0.034781	0.241898	0.208667	RPS6KA5/U	12
GO:00070!	centrosome c	5/272	124/1867(	0.035186	0.244235	0.210683	GADD45A/	5
GO:19015	regulation of	4/272	85/18670	0.035642	0.246916	0.212996	PSMD12/E	4
GO:00434(	positive regul	8/272	258/1867(	0.035804	0.247154	0.2132	GADD45G/	8
GO:19015(	fatty acid deri	6/272	167/1867(	0.035816	0.247154	0.2132	PTGS2/AC!	6
GO:00090(	cellular amino	5/272	125/1867(	0.036234	0.247715	0.213684	DLD/BCKD	5
GO:20001	negative regul	5/272	125/1867(	0.036234	0.247715	0.213684	ARID3A/G/	5
GO:00094(	response to c	3/272	50/18670	0.036279	0.247715	0.213684	RBM3/EIF2	3
GO:00101	body morpho	3/272	50/18670	0.036279	0.247715	0.213684	RAB3GAP1	3
GO:00326(	interferon-bet	3/272	50/18670	0.036279	0.247715	0.213684	TRIM56/PI	3
GO:00508(	positive regul	6/272	168/1867(	0.036709	0.247715	0.213684	PTGS2/MN	6
GO:00990(	vesicle-media	7/272	213/1867(	0.036891	0.247715	0.213684	PFN2/CDK!	7
GO:00017	leukocyte hon	4/272	86/18670	0.036969	0.247715	0.213684	PPP2R3C/T	4
GO:00069	activation of c	4/272	86/18670	0.036969	0.247715	0.213684	NKX3-1/S1	4
GO:00090	branched-cha	2/272	21/18670	0.037017	0.247715	0.213684	DLD/BCKD	2
GO:00090	branched-cha	2/272	21/18670	0.037017	0.247715	0.213684	DLD/BCKD	2
GO:00305(	negative regul	2/272	21/18670	0.037017	0.247715	0.213684	SRGN/HIF1	2
GO:00361!	phosphatidyls	2/272	21/18670	0.037017	0.247715	0.213684	MBOAT1/(	2
GO:00480	positive regul	2/272	21/18670	0.037017	0.247715	0.213684	RBM3/RBN	2

GO:01400!	neuron projec	2/272	21/18670	0.037017	0.247715	0.213684	PHACTR1/I	2
GO:19017:	regulation of r	2/272	21/18670	0.037017	0.247715	0.213684	SCGB3A1/I	2
GO:00324:	regulation of t	5/272	126/1867(	0.037301	0.249142	0.214916	TRIM56/ST	5
GO:00215‹	pallium develo	6/272	169/1867(	0.037615	0.25077	0.21632	TACC3/CDI	6
GO:00308:	regulation of a	3/272	51/18670	0.038144	0.253525	0.218697	F2RL1/LM(	3
GO:00140(	positive regula	4/272	87/18670	0.038324	0.253525	0.218697	NKX3-1/F2	4
GO:00436:	regulation of I	5/272	127/1867(	0.038387	0.253525	0.218697	RPS6KA3/I	5
GO:00457:	positive regula	5/272	127/1867(	0.038387	0.253525	0.218697	CDC123/TI	5
GO:00468{	positive regula	5/272	127/1867(	0.038387	0.253525	0.218697	NKX3-1/PF	5
GO:00421:	B cell activatic	9/272	310/1867(	0.038482	0.253681	0.218831	PPP2R3C/I	9
GO:00000:	cell cycle chec	7/272	216/1867(	0.039307	0.254522	0.219557	ARID3A/G/	7
GO:00436{	post-translatic	10/272	361/1867(	0.039452	0.254522	0.219557	WSB1/PTP	10
GO:00326(	type I interfer	5/272	128/1867(	0.039492	0.254522	0.219557	TRIM56/ST	5
GO:00347(	negative regul	5/272	128/1867(	0.039492	0.254522	0.219557	THBS1/RG!	5
GO:20001:	regulation of r	4/272	88/18670	0.039706	0.254522	0.219557	RAPGEF1/I	4
GO:00069(	cellular defen:	3/272	52/18670	0.040057	0.254522	0.219557	MNDA/LY!	3
GO:00327:	positive regula	3/272	52/18670	0.040057	0.254522	0.219557	MNDA/CLI	3
GO:00514:	regulation of u	3/272	52/18670	0.040057	0.254522	0.219557	GOLGA2/R	3
GO:00100:	response to m	10/272	362/1867(	0.040075	0.254522	0.219557	PTGS2/THI	10
GO:00313:	positive regula	10/272	362/1867(	0.040075	0.254522	0.219557	TOB1/PFKI	10
GO:00362!	cellular respor	7/272	217/1867(	0.040135	0.254522	0.219557	NKX3-1/PT	7
GO:0003(!	glomerular filt	2/272	22/18670	0.040335	0.254522	0.219557	SULF2/F2R	2
GO:00059{	glycogen catal	2/272	22/18670	0.040335	0.254522	0.219557	PYGL/PPP1	2
GO:00198{	antigen proce:	2/272	22/18670	0.040335	0.254522	0.219557	B2M/TAP2	2
GO:00329:	regulation of s	2/272	22/18670	0.040335	0.254522	0.219557	F2RL1/CLE	2
GO:00346‹	establishment	2/272	22/18670	0.040335	0.254522	0.219557	KIF1B/HIF1	2
GO:00400:	negative regul	2/272	22/18670	0.040335	0.254522	0.219557	SULF2/THE	2
GO:00420!	interleukin-2 I	2/272	22/18670	0.040335	0.254522	0.219557	CLEC7A/IL:	2
GO:00456:	positive regula	2/272	22/18670	0.040335	0.254522	0.219557	IL4R/IL18	2
GO:00474!	mitochondrioı	2/272	22/18670	0.040335	0.254522	0.219557	KIF1B/HIF1	2
GO:00603!	regulation of S	2/272	22/18670	0.040335	0.254522	0.219557	TOB1/PBLI	2
GO:00903:	positive regula	2/272	22/18670	0.040335	0.254522	0.219557	JDP2/LRRK	2
GO:19017!	positive regula	2/272	22/18670	0.040335	0.254522	0.219557	RPL26/RPL	2
GO:19046(	regulation of u	2/272	22/18670	0.040335	0.254522	0.219557	GOLGA2/R	2
GO:00714{	cellular respor	5/272	129/1867(	0.040616	0.255839	0.220693	RGR/PTGS:	5
GO:00301:	regulation of \	10/272	363/1867(	0.040705	0.25594	0.22078	SULF2/RAF	10
GO:00092!	ribonucleotide	11/272	414/1867(	0.040873	0.256544	0.221301	DLD/PDE7/	11
GO:00322:	regulation of p	7/272	218/1867(	0.040974	0.256722	0.221454	PFN2/GMF	7
GO:00016!	temperature I	6/272	173/1867(	0.041385	0.258837	0.223278	GADD45G/	6
GO:00448:	cell cycle G2/I	8/272	266/1867(	0.041627	0.259893	0.22419	PSMD12/C	8
GO:00506:	positive regula	5/272	130/1867(	0.04176	0.260264	0.224509	TNFSF13B/	5
GO:00316:	zymogen activ	3/272	53/18670	0.042018	0.260496	0.22471	PLAU/THB!	3
GO:00702:	regulation of I	3/272	53/18670	0.042018	0.260496	0.22471	FADD/HIF1	3
GO:00713:	cellular respor	3/272	53/18670	0.042018	0.260496	0.22471	RAPGEF1/I	3
GO:00423:	negative regul	12/272	468/1867(	0.042211	0.261089	0.225222	PAK2/SH3I	12
GO:19019{	negative regul	8/272	267/1867(	0.042396	0.261089	0.225222	ARID3A/PS	8
GO:19030:	negative regul	4/272	90/18670	0.042556	0.261089	0.225222	PLAU/THB!	4
GO:00329‹	positive regula	5/272	131/1867(	0.042923	0.261089	0.225222	TNFSF13B/	5

GO:19028(	negative regul	5/272	131/1867(	0.042923	0.261089	0.225222	ARID3A/G/	5
GO:00024:	antigen proce:	6/272	175/1867(	0.043356	0.261089	0.225222	PSMD12/B	6
GO:00109:	regulation of p	6/272	175/1867(	0.043356	0.261089	0.225222	PPP2R3A/E	6
GO:00092!	glucan catabo	2/272	23/18670	0.043761	0.261089	0.225222	PYGL/PPP1	2
GO:00104!	proteasomal u	2/272	23/18670	0.043761	0.261089	0.225222	PSMA1/PS	2
GO:00165:	histone deubi	2/272	23/18670	0.043761	0.261089	0.225222	USP15/USI	2
GO:00355(	regulation of c	2/272	23/18670	0.043761	0.261089	0.225222	DTX3L/BT/	2
GO:00458:	positive regul	2/272	23/18670	0.043761	0.261089	0.225222	PFKFB2/HI	2
GO:00897:	amino acid im	2/272	23/18670	0.043761	0.261089	0.225222	RGS2/SLC3	2
GO:00972(	renal filtratior	2/272	23/18670	0.043761	0.261089	0.225222	SULF2/F2R	2
GO:19010:	negative regul	2/272	23/18670	0.043761	0.261089	0.225222	TXN/LRRK:	2
GO:19032(	negative regul	2/272	23/18670	0.043761	0.261089	0.225222	TXN/LRRK:	2
GO:19034:	regulation of c	2/272	23/18670	0.043761	0.261089	0.225222	CLEC7A/LR	2
GO:00456:	positive regul	4/272	91/18670	0.044023	0.261089	0.225222	STAT1/FAD	4
GO:00518!	membrane de	4/272	91/18670	0.044023	0.261089	0.225222	PPP2R3C/(	4
GO:00603:	interferon-gar	4/272	91/18670	0.044023	0.261089	0.225222	IFNGR1/ST	4
GO:00027:	positive regul	3/272	54/18670	0.044026	0.261089	0.225222	F2RL1/B2N	3
GO:00300	actin filament	3/272	54/18670	0.044026	0.261089	0.225222	F2RL1/LM(	3
GO:00326!	regulation of i	3/272	54/18670	0.044026	0.261089	0.225222	THBS1/CLE	3
GO:00353!	thioester bios	3/272	54/18670	0.044026	0.261089	0.225222	DLD/ACSL1	3
GO:00716:	acyl-CoA biosy	3/272	54/18670	0.044026	0.261089	0.225222	DLD/ACSL1	3
GO:00061(	nucleoside dip	5/272	132/1867(	0.044105	0.261089	0.225222	PFKFB2/HK	5
GO:00432!	positive regul	5/272	132/1867(	0.044105	0.261089	0.225222	NKX3-1/CL	5
GO:00706(	regulation of l	7/272	222/1867(	0.044445	0.262661	0.226577	MNDA/TN	7
GO:00068:	neurotransmit	8/272	270/1867(	0.044762	0.264094	0.227813	ICA1/PFN2	8
GO:00310:	microtubule o	5/272	133/1867(	0.045307	0.265555	0.229074	GADD45A/	5
GO:00550:	transition met	5/272	133/1867(	0.045307	0.265555	0.229074	S100A9/B2	5
GO:00713:	cellular respor	5/272	133/1867(	0.045307	0.265555	0.229074	PTPRN2/H	5
GO:00019!	regulation of c	6/272	177/1867(	0.045384	0.265555	0.229074	F2RL1/IFN(	6
GO:00484(	cell maturatio	6/272	177/1867(	0.045384	0.265555	0.229074	DLD/ROPN	6
GO:00165(	covalent chror	12/272	474/1867(	0.045711	0.267	0.23032	RPS6KA5/U	12
GO:00326:	interleukin-10	3/272	55/18670	0.046082	0.267	0.23032	F2RL1/CLE	3
GO:00430:	regulation of r	3/272	55/18670	0.046082	0.267	0.23032	THBS1/IL4I	3
GO:00974:	synaptic vesicl	3/272	55/18670	0.046082	0.267	0.23032	LRRK2/PIC.	3
GO:00109!	positive regul	6/272	178/1867(	0.046421	0.267	0.23032	NKX3-1/CL	6
GO:00091:	purine nucleo:	5/272	134/1867(	0.046528	0.267	0.23032	PFKFB2/HK	5
GO:00091:	purine ribonu	5/272	134/1867(	0.046528	0.267	0.23032	PFKFB2/HK	5
GO:00322:	positive regul	5/272	134/1867(	0.046528	0.267	0.23032	PFN2/GMF	5
GO:00466!	response to oı	5/272	134/1867(	0.046528	0.267	0.23032	PTGS2/RAI	5
GO:00469:	nucleotide ph	5/272	134/1867(	0.046528	0.267	0.23032	PFKFB2/HK	5
GO:00342	regulation of c	12/272	476/1867(	0.046921	0.267	0.23032	RPS6KA3/(	12
GO:00064:	protein deace	4/272	93/18670	0.047042	0.267	0.23032	SAP30/JDP	4
GO:00352	synaptic trans	4/272	93/18670	0.047042	0.267	0.23032	PTGS2/RAI	4
GO:00622(	regulation of p	4/272	93/18670	0.047042	0.267	0.23032	F2RL1/LY9	4
GO:00456(	negative regul	7/272	225/1867(	0.047168	0.267	0.23032	KIAA0319/	7
GO:00108(	negative regul	2/272	24/18670	0.047291	0.267	0.23032	F2RL1/RFF	2
GO:00360:	protein localiz	2/272	24/18670	0.047291	0.267	0.23032	DTX3L/RAI	2
GO:00421(	positive regul	2/272	24/18670	0.047291	0.267	0.23032	FADD/IL18	2

GO:00428:	defense respo	2/272	24/18670	0.047291	0.267	0.23032	IL4R/CLEC7	2
GO:00433(	positive regul	2/272	24/18670	0.047291	0.267	0.23032	F2RL1/IL4F	2
GO:00468:	carbohydrate	2/272	24/18670	0.047291	0.267	0.23032	PFKFB2/HK	2
GO:19001:	negative regul	2/272	24/18670	0.047291	0.267	0.23032	PAK2/RFFL	2
GO:00301:	positive regul	6/272	179/1867(	0.047472	0.267594	0.230833	SULF2/PSN	6
GO:00098!	positive regul	11/272	425/1867(	0.047739	0.268411	0.231537	TOB1/PFKI	11
GO:00713:	cellular respo	5/272	135/1867(	0.047768	0.268411	0.231537	PTPRN2/H	5
GO:00019:	positive regul	3/272	56/18670	0.048184	0.26904	0.23208	F2RL1/B2N	3
GO:00326:	interleukin-12	3/272	56/18670	0.048184	0.26904	0.23208	THBS1/CLE	3
GO:00709:	protein K48-li	3/272	56/18670	0.048184	0.26904	0.23208	UBE2D3/R	3
GO:19032(	negative regul	3/272	56/18670	0.048184	0.26904	0.23208	TXN/HIF1A	3
GO:19017!	regulation of s	6/272	180/1867(	0.048537	0.270585	0.233412	TAF15/CDI	6
GO:00091ℓ	ribonucleoside	5/272	136/1867(	0.049028	0.272463	0.235033	PFKFB2/HK	5
GO:00713:	cellular respo	5/272	136/1867(	0.049028	0.272463	0.235033	PTPRN2/H	5
GO:19019ℓ	regulation of (	12/272	480/1867(	0.049407	0.274141	0.236481	ARID3A/PS	12
GO:00511ℓ	regulation of r	6/272	181/1867(	0.049617	0.274784	0.237034	SCGB3A1/I	6
GO:00603:	type I interfer	4/272	95/18670	0.050173	0.274784	0.237034	IFI6/STAT1	4
GO:00713!	cellular respo	4/272	95/18670	0.050173	0.274784	0.237034	IFI6/STAT1	4
GO:00607(	positive regul	3/272	57/18670	0.050333	0.274784	0.237034	FADD/HIF1	3
GO:19004(	negative regul	3/272	57/18670	0.050333	0.274784	0.237034	TXN/HIF1A	3
GO:19034:	positive regul	3/272	57/18670	0.050333	0.274784	0.237034	PTGS2/CLE	3
GO:00198ℓ	antigen proce	6/272	182/1867(	0.050712	0.274784	0.237034	PSMD12/B	6
GO:00300ℓ	actin filament	6/272	182/1867(	0.050712	0.274784	0.237034	PFN2/GMI	6
GO:00015(	response to p	2/272	25/18670	0.05092	0.274784	0.237034	IL4R/CLEC7	2
GO:00020!	positive regul	2/272	25/18670	0.05092	0.274784	0.237034	STAT1/IRS:	2
GO:00316:	plasminogen a	2/272	25/18670	0.05092	0.274784	0.237034	PLAU/THB!	2
GO:00326:	interleukin-13	2/272	25/18670	0.05092	0.274784	0.237034	IL17RA/IL1	2
GO:00353:	long-chain fat	2/272	25/18670	0.05092	0.274784	0.237034	ACSL1/HSD	2
GO:00442ℓ	cellular polysa	2/272	25/18670	0.05092	0.274784	0.237034	PYGL/PPP1	2
GO:00516!	establishment	2/272	25/18670	0.05092	0.274784	0.237034	KIF1B/HIF1	2
GO:00519!	negative regul	2/272	25/18670	0.05092	0.274784	0.237034	RGS2/ABA	2
GO:00603:	regulation of r	2/272	25/18670	0.05092	0.274784	0.237034	IFNGR1/ST	2
GO:00603:	regulation of i	2/272	25/18670	0.05092	0.274784	0.237034	IFNGR1/ST	2
GO:00105(	regulation of a	9/272	328/1867(	0.051657	0.277734	0.239579	USP10/EX(	9
GO:00068:	dicarboxylic a	4/272	96/18670	0.051781	0.277734	0.239579	FOLR3/RAI	4
GO:00327!	positive regul	4/272	96/18670	0.051781	0.277734	0.239579	F2RL1/CLE	4
GO:00902:	positive regul	4/272	96/18670	0.051781	0.277734	0.239579	PFKFB2/HI	4
GO:00061(	purine nucleo	11/272	432/1867(	0.052496	0.280462	0.241933	DLD/PDE7/	11
GO:00619(	glial cell activ	3/272	58/18670	0.052527	0.280462	0.241933	IFNGR1/TL	3
GO:19020ℓ	regulation of e	3/272	58/18670	0.052527	0.280462	0.241933	THBS1/RFI	3
GO:00027(	regulation of p	5/272	139/1867(	0.052925	0.281735	0.243031	F2RL1/IL4F	5
GO:00706(	positive regul	5/272	139/1867(	0.052925	0.281735	0.243031	TNFSF13B/	5
GO:00515!	response to c	4/272	97/18670	0.053416	0.283499	0.244552	RAPGEF1/S	4
GO:01201(	positive regul	4/272	97/18670	0.053416	0.283499	0.244552	GADD45G/	4
GO:00716!	anatomical str	7/272	232/1867(	0.053932	0.285532	0.246307	DLD/ROPN	7
GO:00463:	regulation of J	6/272	185/1867(	0.054085	0.285532	0.246307	GADD45G/	6
GO:00019:	positive regul	2/272	26/18670	0.054647	0.285532	0.246307	B2M/FADI	2
GO:00030ℓ	regulation of s	2/272	26/18670	0.054647	0.285532	0.246307	F2RL1/MN	2

GO:00356	exploration be	2/272	26/18670	0.054647	0.285532	0.246307	LRRK2/AB	2
GO:00430	amino acid im	2/272	26/18670	0.054647	0.285532	0.246307	RGS2/SLC3	2
GO:19024	regulation of	2/272	26/18670	0.054647	0.285532	0.246307	RAP1A/NP	2
GO:00018	release of cyto	3/272	59/18670	0.054767	0.285532	0.246307	IFI6/PLAUI	3
GO:00018	endothelial ce	3/272	59/18670	0.054767	0.285532	0.246307	F2RL1/RAF	3
GO:00327	positive regula	3/272	59/18670	0.054767	0.285532	0.246307	MNDA/CLE	3
GO:00433	positive regula	3/272	59/18670	0.054767	0.285532	0.246307	TXN/PLAU	3
GO:19028	negative regul	3/272	59/18670	0.054767	0.285532	0.246307	TXN/HIF1A	3
GO:00313	regulation of	4/272	98/18670	0.05508	0.286738	0.247346	F2RL1/CLE	4
GO:00508	modulation of	11/272	436/18670	0.055353	0.287739	0.24821	NTNG2/PT	11
GO:00714	cellular respo	7/272	234/18670	0.05597	0.290256	0.250381	NKX3-1/PT	7
GO:00512	protein polym	8/272	283/18670	0.056007	0.290256	0.250381	PFN2/GMF	8
GO:00991	regulation of t	11/272	437/18670	0.056083	0.290256	0.250381	NTNG2/PT	11
GO:00308	positive regula	4/272	99/18670	0.056771	0.291858	0.251764	PFN2/GMF	4
GO:19015	fatty acid deri	4/272	99/18670	0.056771	0.291858	0.251764	PTGS2/AC	4
GO:00217	developmenta	8/272	284/18670	0.05694	0.291858	0.251764	DLD/ROPN	8
GO:00380	Fc-gamma rec	5/272	142/18670	0.056997	0.291858	0.251764	CLEC4D/R	5
GO:00508	positive regula	5/272	142/18670	0.056997	0.291858	0.251764	PPP2R3C/	5
GO:19029	negative regul	5/272	142/18670	0.056997	0.291858	0.251764	PFN2/GMF	5
GO:00467	protein autop	7/272	235/18670	0.057007	0.291858	0.251764	PAK2/CLK4	7
GO:00325	negative regul	3/272	60/18670	0.057053	0.291858	0.251764	LMTK2/PH	3
GO:00600	canonical Wnt	9/272	335/18670	0.057495	0.293695	0.253348	SULF2/RAF	9
GO:00712	cellular respo	7/272	236/18670	0.058056	0.294108	0.253704	CLEC7A/LY	7
GO:00488	dendrite morp	5/272	143/18670	0.058393	0.294108	0.253704	CDK5R1/PI	5
GO:00002	polysaccharid	2/272	27/18670	0.058466	0.294108	0.253704	PYGL/PPP1	2
GO:00028	regulation of T	2/272	27/18670	0.058466	0.294108	0.253704	IL4R/IL18	2
GO:00459	negative regul	2/272	27/18670	0.058466	0.294108	0.253704	PTGS2/RG	2
GO:00482	negative regul	2/272	27/18670	0.058466	0.294108	0.253704	UNC119/P	2
GO:00601	positive regula	2/272	27/18670	0.058466	0.294108	0.253704	SCGB3A1/	2
GO:00603	innervation	2/272	27/18670	0.058466	0.294108	0.253704	SULF2/CHD	2
GO:20001	regulation of f	2/272	27/18670	0.058466	0.294108	0.253704	THBS1/IRS	2
GO:20010	negative regul	2/272	27/18670	0.058466	0.294108	0.253704	TXN/LRRK	2
GO:00021	cytoplasmic tr	4/272	100/18670	0.058489	0.294108	0.253704	RPS28/RPS	4
GO:00480	antigen proce	6/272	189/18670	0.05879	0.29483	0.254327	PSMD12/B	6
GO:00328	regulation of	7/272	237/18670	0.059116	0.29483	0.254327	GADD45G/	7
GO:00431	regulation of I	7/272	237/18670	0.059116	0.29483	0.254327	F2RL1/STA	7
GO:00071	leukocyte cell	9/272	337/18670	0.059238	0.29483	0.254327	PAK2/SIRP	9
GO:00434	regulation of I	9/272	337/18670	0.059238	0.29483	0.254327	GADD45G/	9
GO:00022	alpha-beta T c	3/272	61/18670	0.059382	0.29483	0.254327	IL4R/EOMI	3
GO:00022	alpha-beta T c	3/272	61/18670	0.059382	0.29483	0.254327	IL4R/EOMI	3
GO:00451	cellular extrav	3/272	61/18670	0.059382	0.29483	0.254327	GOLPH3/F	3
GO:19039	positive regula	3/272	61/18670	0.059382	0.29483	0.254327	SRPK2/IFIT	3
GO:00457	positive regula	10/272	389/18670	0.05952	0.295101	0.254561	NKX3-1/AF	10
GO:19021	positive regula	5/272	144/18670	0.059809	0.296119	0.255439	PPP2R3C/I	5
GO:00459	negative regul	9/272	338/18670	0.060122	0.296654	0.2559	NKX3-1/AF	9
GO:00326	interleukin-1	4/272	101/18670	0.060236	0.296654	0.2559	MNDA/F2F	4
GO:00069	autophagy	12/272	496/18670	0.060253	0.296654	0.2559	RAB24/S1	12
GO:00619	process utilizi	12/272	496/18670	0.060253	0.296654	0.2559	RAB24/S1	12

GO:00714:	cellular respo	6/272	191/1867(	0.061232	0.300011	0.258796	RGR/PTGS:	6
GO:00761	learning	5/272	145/1867(	0.061245	0.300011	0.258796	PTGS2/EIF:	5
GO:00330(	muscle cell pr	7/272	239/1867(	0.061273	0.300011	0.258796	PTGS2/THI	7
GO:00703(	regulation of s	7/272	239/1867(	0.061273	0.300011	0.258796	GADD45G/	7
GO:00065:	regulation of c	3/272	62/18670	0.061756	0.301958	0.260476	PSMD12/P	3
GO:00023(	cytokine prod	4/272	102/1867(	0.062009	0.30201	0.260521	F2RL1/B2N	4
GO:00988(	cellular oxidar	4/272	102/1867(	0.062009	0.30201	0.260521	TXN/PTGS:	4
GO:00442ξ	small molecul	11/272	445/1867(	0.062156	0.30201	0.260521	DLD/UPB1,	11
GO:00026ξ	regulation of c	2/272	28/18670	0.062374	0.30201	0.260521	FADD/BST:	2
GO:00354ξ	response to in	2/272	28/18670	0.062374	0.30201	0.260521	MNDA/STA	2
GO:00454(	regulation of i	2/272	28/18670	0.062374	0.30201	0.260521	CLEC7A/TL	2
GO:19020(	regulation of a	2/272	28/18670	0.062374	0.30201	0.260521	IFNGR1/PI(	2
GO:00509(	leukocyte mig	12/272	499/1867(	0.06245	0.30201	0.260521	F2RL1/THE	12
GO:00091(	coenzyme bio	5/272	146/1867(	0.062699	0.302805	0.261206	DLD/PTGS:	5
GO:00425ξ	glucose home	7/272	241/1867(	0.063478	0.306148	0.26409	PYGL/HK2/	7
GO:00986ξ	anion transme	8/272	291/1867(	0.063749	0.306915	0.264752	CLCC1/THE	8
GO:00356(	protein deacy	4/272	103/1867(	0.063811	0.306915	0.264752	SAP30/JDP	4
GO:00902(	positive regul	5/272	147/1867(	0.064174	0.308242	0.265897	SULF2/PSN	5
GO:00335(	carbohydrate	7/272	242/1867(	0.064598	0.309364	0.266864	PYGL/HK2/	7
GO:00069:	nucleocytopla	9/272	343/1867(	0.064669	0.309364	0.266864	TXN/PTGS:	9
GO:00329ξ	regulation of a	9/272	343/1867(	0.064669	0.309364	0.266864	F2RL1/PFN	9
GO:00148:	muscle cell mi	4/272	104/1867(	0.065639	0.311913	0.269063	PLAU/BIN3	4
GO:00469:	regulation of r	4/272	104/1867(	0.065639	0.311913	0.269063	PFN2/RAB:	4
GO:00987:	macromolecu	4/272	104/1867(	0.065639	0.311913	0.269063	SAP30/JDP	4
GO:20012:	negative regul	4/272	104/1867(	0.065639	0.311913	0.269063	THBS1/IFI6	4
GO:00514ξ	negative regul	5/272	148/1867(	0.065667	0.311913	0.269063	PFN2/GMF	5
GO:00092(	response to te	7/272	243/1867(	0.065731	0.311913	0.269063	PTGS2/THI	7
GO:00059:	regulation of g	2/272	29/18670	0.066369	0.312428	0.269507	IRS2/PPP1	2
GO:00094:	NAD biosynth	2/272	29/18670	0.066369	0.312428	0.269507	PTGS2/SLC	2
GO:00109(	regulation of g	2/272	29/18670	0.066369	0.312428	0.269507	IRS2/PPP1	2
GO:00310(	regulation of l	2/272	29/18670	0.066369	0.312428	0.269507	JDP2/LRRK	2
GO:00422:	interleukin-6 l	2/272	29/18670	0.066369	0.312428	0.269507	CLEC7A/TL	2
GO:00601ʻ	regulation of s	2/272	29/18670	0.066369	0.312428	0.269507	SCGB3A1/I	2
GO:00300:	lamellipodium	3/272	64/18670	0.066635	0.312848	0.269869	BIN3/GOLI	3
GO:00357:	insulin secreti	3/272	64/18670	0.066635	0.312848	0.269869	PTPRN2/H	3
GO:00140:	response to p	5/272	149/1867(	0.06718	0.314158	0.271	PTGS2/RAI	5
GO:00507:	regulation of c	5/272	149/1867(	0.06718	0.314158	0.271	KIAA0319/	5
GO:20010ξ	positive regul	5/272	149/1867(	0.06718	0.314158	0.271	NKX3-1/CL	5
GO:00027(	positive regul	4/272	105/1867(	0.067495	0.315212	0.271909	EXOSC3/B:	4
GO:00103ξ	regulation of (	6/272	196/1867(	0.067595	0.315267	0.271957	PSMD12/C	6
GO:00109ξ	positive regul	6/272	197/1867(	0.068913	0.319718	0.275796	NKX3-1/CL	6
GO:00069ʻ	regulation of s	3/272	65/18670	0.069138	0.319718	0.275796	PTGS2/RG:	3
GO:00327:	positive regul	3/272	65/18670	0.069138	0.319718	0.275796	CLEC7A/FA	3
GO:00353(	negative regul	3/272	65/18670	0.069138	0.319718	0.275796	LMTK2/PH	3
GO:00421:	neurotransmit	4/272	106/1867(	0.069377	0.319718	0.275796	PTGS2/UPI	4
GO:20010:	regulation of r	4/272	106/1867(	0.069377	0.319718	0.275796	TXN/RGS2,	4
GO:00065:	cellular amino	9/272	348/1867(	0.06943	0.319718	0.275796	DLD/UPB1,	9
GO:00310ξ	regeneration	6/272	198/1867(	0.070245	0.319718	0.275796	SULF2/IFRI	6

GO:00028:	regulation of t	2/272	30/18670	0.070446	0.319718	0.275796	IL4R/IL18	2
GO:00107	regulation of r	2/272	30/18670	0.070446	0.319718	0.275796	NFKBIA/IL1	2
GO:00381:	interleukin-7-r	2/272	30/18670	0.070446	0.319718	0.275796	IRS2/BRWI	2
GO:00433:	positive regul	2/272	30/18670	0.070446	0.319718	0.275796	IL4R/IL18	2
GO:00459	positive regul	2/272	30/18670	0.070446	0.319718	0.275796	CDC123/EI	2
GO:00701	negative regul	2/272	30/18670	0.070446	0.319718	0.275796	SRGN/HIF1	2
GO:00900:	regulation of r	2/272	30/18670	0.070446	0.319718	0.275796	CAMK1D/E	2
GO:00902	positive regul	2/272	30/18670	0.070446	0.319718	0.275796	PLAUR/MN	2
GO:00974:	liver regenera	2/272	30/18670	0.070446	0.319718	0.275796	SULF2/RAF	2
GO:00990	postsynapse a	2/272	30/18670	0.070446	0.319718	0.275796	NTNG2/CD	2
GO:01101	negative regul	2/272	30/18670	0.070446	0.319718	0.275796	SRGN/HIF1	2
GO:19018	negative regul	2/272	30/18670	0.070446	0.319718	0.275796	THBS1/TLR	2
GO:19032	regulation of I	2/272	30/18670	0.070446	0.319718	0.275796	TXN/LRRK2	2
GO:00706	leukocyte prol	8/272	298/1867	0.071045	0.322023	0.277784	MNDA/F2R	8
GO:00019	leukocyte me	4/272	107/1867	0.071286	0.322291	0.278016	F2RL1/B2M	4
GO:00485:	positive regul	4/272	107/1867	0.071286	0.322291	0.278016	POLR2J/SR	4
GO:00061	regulation of	6/272	199/1867	0.071592	0.32326	0.278851	PFKFB2/RA	6
GO:00065:	protein mono	3/272	66/18670	0.071684	0.323262	0.278853	UBE2D3/TI	3
GO:00421:	neurotransmit	5/272	153/1867	0.073425	0.330692	0.285263	PTGS2/UPI	5
GO:00421	positive regul	3/272	67/18670	0.074271	0.331778	0.286199	THBS1/CLE	3
GO:00422	ribosomal sm	3/272	67/18670	0.074271	0.331778	0.286199	RPS28/RPS	3
GO:00487	muscle fiber d	3/272	67/18670	0.074271	0.331778	0.286199	BIN3/HDA	3
GO:00027:	regulation of T	2/272	31/18670	0.074603	0.331778	0.286199	B2M/IL18	2
GO:00066	phosphatidyls	2/272	31/18670	0.074603	0.331778	0.286199	MBOAT1/C	2
GO:00107	regulation of f	2/272	31/18670	0.074603	0.331778	0.286199	THBS1/RFF	2
GO:00190	modulation by	2/272	31/18670	0.074603	0.331778	0.286199	EIF2AK2/EI	2
GO:00456	positive regul	2/272	31/18670	0.074603	0.331778	0.286199	STAT1/HIF	2
GO:00457	positive regul	2/272	31/18670	0.074603	0.331778	0.286199	PLAUR/MN	2
GO:00506	positive regul	2/272	31/18670	0.074603	0.331778	0.286199	RBM3/RBN	2
GO:00091:	nucleoside dip	5/272	154/1867	0.075034	0.333276	0.287491	PFKFB2/HK	5
GO:00511	positive regul	4/272	109/1867	0.075185	0.333529	0.28771	SCGB3A1/I	4
GO:00508:	positive regul	6/272	202/1867	0.075722	0.335488	0.2894	PAK2/SIRP	6
GO:00338	nucleoside bis	3/272	68/18670	0.076899	0.339431	0.292801	DLD/ACSL1	3
GO:00340:	ribonucleoside	3/272	68/18670	0.076899	0.339431	0.292801	DLD/ACSL1	3
GO:00340:	purine nucleo	3/272	68/18670	0.076899	0.339431	0.292801	DLD/ACSL1	3
GO:00069:	smooth muscl	4/272	110/1867	0.077174	0.339798	0.293117	SULF2/PTG	4
GO:00335	unsaturated fa	4/272	110/1867	0.077174	0.339798	0.293117	PTGS2/AC	4
GO:00725:	purine-contair	11/272	464/1867	0.078262	0.342011	0.295026	DLD/PDE7	11
GO:19059	regulation of I	5/272	156/1867	0.07831	0.342011	0.295026	NKX3-1/TH	5
GO:00025:	myeloid leuko	6/272	204/1867	0.078549	0.342011	0.295026	F2RL1/OSC	6
GO:19029	positive regul	6/272	204/1867	0.078549	0.342011	0.295026	F2RL1/PFN	6
GO:00082:	estrogen meta	2/272	32/18670	0.078836	0.342011	0.295026	HSD17B11	2
GO:00193	nicotinamide	2/272	32/18670	0.078836	0.342011	0.295026	PTGS2/SLC	2
GO:00193	pyridine nucle	2/272	32/18670	0.078836	0.342011	0.295026	PTGS2/SLC	2
GO:00459	positive regul	2/272	32/18670	0.078836	0.342011	0.295026	PTGS2/AB	2
GO:00469	fatty-acyl-CoA	2/272	32/18670	0.078836	0.342011	0.295026	ACSL1/HSD	2
GO:00519	positive regul	2/272	32/18670	0.078836	0.342011	0.295026	PTGS2/RAI	2
GO:00603:	face morphog	2/272	32/18670	0.078836	0.342011	0.295026	RAB3GAP1	2

GO:19007	positive regul	2/272	32/18670	0.078836	0.342011	0.295026	GADD45G/	2
GO:00518	protein autou	3/272	69/18670	0.079568	0.344341	0.297036	UBE2D3/LI	3
GO:00702	lymphocyte a	3/272	69/18670	0.079568	0.344341	0.297036	FADD/HIF1	3
GO:00027	regulation of l	6/272	205/1867	0.079984	0.345299	0.297863	F2RL1/IL4R	6
GO:00158	drug transport	6/272	205/1867	0.079984	0.345299	0.297863	LMBRD1/R	6
GO:19907	cellular detoxi	4/272	112/1867	0.081231	0.350254	0.302137	TXN/PTGS2	4
GO:00027	regulation of T	3/272	70/18670	0.082277	0.350388	0.302252	B2M/FADD	3
GO:00615	myeloid cell d	3/272	70/18670	0.082277	0.350388	0.302252	EVI2B/TLR	3
GO:00720	regulation of s	3/272	70/18670	0.082277	0.350388	0.302252	EIF2AK2/H	3
GO:00336	negative regul	7/272	257/1867	0.08284	0.350388	0.302252	PAK2/SH3I	7
GO:00488	stem cell diffe	7/272	257/1867	0.08284	0.350388	0.302252	PSMD12/E	7
GO:00019	regulation of T	2/272	33/18670	0.083143	0.350388	0.302252	B2M/FADD	2
GO:00028	positive regul	2/272	33/18670	0.083143	0.350388	0.302252	F2RL1/IL4R	2
GO:00104	regulation of r	2/272	33/18670	0.083143	0.350388	0.302252	STAT1/IRS	2
GO:00105	regulation of l	2/272	33/18670	0.083143	0.350388	0.302252	BIN3/HDA	2
GO:00109	negative regul	2/272	33/18670	0.083143	0.350388	0.302252	THBS1/LRF	2
GO:00193	pyridine nucle	2/272	33/18670	0.083143	0.350388	0.302252	PTGS2/SLC	2
GO:00199	cGMP-mediat	2/272	33/18670	0.083143	0.350388	0.302252	PRKAR2A/	2
GO:00464	nicotinamide	2/272	33/18670	0.083143	0.350388	0.302252	PTGS2/SLC	2
GO:00485	spleen develo	2/272	33/18670	0.083143	0.350388	0.302252	PPP2R3C/F	2
GO:00702	regulation of T	2/272	33/18670	0.083143	0.350388	0.302252	FADD/HIF1	2
GO:00990	postsynaptic s	2/272	33/18670	0.083143	0.350388	0.302252	NTNG2/CD	2
GO:19011	positive regul	2/272	33/18670	0.083143	0.350388	0.302252	PLAUR/MN	2
GO:19026	regulation of r	2/272	33/18670	0.083143	0.350388	0.302252	HIF1A/LRR	2
GO:19033	negative regul	2/272	33/18670	0.083143	0.350388	0.302252	THBS1/LRF	2
GO:00069	muscle contra	9/272	363/1867	0.084998	0.35689	0.307862	SULF2/PTG	9
GO:00181	peptidyl-tyros	9/272	363/1867	0.084998	0.35689	0.307862	PAK2/UNC	9
GO:00075	skeletal muscl	5/272	160/1867	0.085088	0.35689	0.307862	SAP30/BIN	5
GO:00026	regulation of l	4/272	114/1867	0.085392	0.35689	0.307862	F2RL1/THE	4
GO:00066	icosanoid met	4/272	114/1867	0.085392	0.35689	0.307862	PTGS2/CYF	4
GO:00085	fibroblast gro	4/272	114/1867	0.085392	0.35689	0.307862	POLR2J/SU	4
GO:00302	erythrocyte di	4/272	114/1867	0.085392	0.35689	0.307862	STAT1/B2N	4
GO:00171	negative regul	6/272	209/1867	0.085874	0.358482	0.309235	TOB1/RBM	6
GO:00326	interleukin-6	5/272	161/1867	0.086829	0.361877	0.312163	F2RL1/CLE	5
GO:00431	proteasome-n	10/272	419/1867	0.087376	0.361877	0.312163	UBE2D3/P	10
GO:00326	interleukin-1	4/272	115/1867	0.08751	0.361877	0.312163	MNDA/F2F	4
GO:19005	regulation of	4/272	115/1867	0.08751	0.361877	0.312163	PFKFB2/RA	4
GO:00328	positive regul	2/272	34/18670	0.08752	0.361877	0.312163	RAB3GAP1	2
GO:00331	positive regul	2/272	34/18670	0.08752	0.361877	0.312163	RBM3/RBN	2
GO:00342	amyloid-beta	2/272	34/18670	0.08752	0.361877	0.312163	IFNGR1/PI	2
GO:00364	cell death in r	2/272	34/18670	0.08752	0.361877	0.312163	TXN/LRRK	2
GO:00025	production of	3/272	72/18670	0.087811	0.361877	0.312163	IL4R/CLEC	3
GO:00508	negative regul	3/272	72/18670	0.087811	0.361877	0.312163	PTGS2/RAI	3
GO:00511	positive regul	3/272	72/18670	0.087811	0.361877	0.312163	SCGB3A1/I	3
GO:01100	regulation of	7/272	261/1867	0.088159	0.362887	0.313034	F2RL1/PFN	7
GO:00182	peptidyl-tyros	9/272	366/1867	0.088344	0.363227	0.313328	PAK2/UNC	9
GO:00345	protein localiz	7/272	262/1867	0.089518	0.367332	0.316869	TXN/PTGS	7
GO:00094	response to li	8/272	314/1867	0.089565	0.367332	0.316869	RGR/UNC1	8

GO:20007:	regulation of s	4/272	116/1867(	0.089654	0.367332	0.316869	PSMD12/E	4
GO:00712:	cellular respo	6/272	212/1867(	0.090444	0.370072	0.319232	LY96/TLR1	6
GO:00311(	animal organ	3/272	73/18670	0.090636	0.370072	0.319232	SULF2/UPF	3
GO:19035:	positive regul:	3/272	73/18670	0.090636	0.370072	0.319232	F2RL1/PTG	3
GO:0031O9	stress-activate	8/272	315/1867(	0.090808	0.370344	0.319467	GADD45G/	8
GO:00061	regulation of r	4/272	117/1867(	0.091823	0.370754	0.31982	PFKFB2/RA	4
GO:00327:	positive regul:	2/272	35/18670	0.091964	0.370754	0.31982	CLEC7A/TL	2
GO:00420	type 2 immun	2/272	35/18670	0.091964	0.370754	0.31982	IL4R/IL18	2
GO:00456	regulation of T	2/272	35/18670	0.091964	0.370754	0.31982	IL4R/IL18	2
GO:00519!	positive regul:	2/272	35/18670	0.091964	0.370754	0.31982	RAB3GAP1	2
GO:00708	regulation of g	2/272	35/18670	0.091964	0.370754	0.31982	IRS2/PPP1	2
GO:00725	pyridine-conta	2/272	35/18670	0.091964	0.370754	0.31982	PTGS2/SLC	2
GO:19029	regulation of a	2/272	35/18670	0.091964	0.370754	0.31982	IFNGR1/PI	2
GO:20007	regulation of a	2/272	35/18670	0.091964	0.370754	0.31982	RAB3GAP1	2
GO:19027	regulation of c	6/272	213/1867(	0.091996	0.370754	0.31982	PSMD12/C	6
GO:00519(	negative regul	8/272	316/1867(	0.09206	0.370754	0.31982	KIAA0319/	8
GO:00064:	regulation of t	10/272	424/1867(	0.092677	0.372816	0.3216	RPS6KA3/C	10
GO:00059	glycogen meta	3/272	74/18670	0.093499	0.375097	0.323568	PYGL/IRS2,	3
GO:00063(	rRNA processi	6/272	214/1867(	0.093562	0.375097	0.323568	RPS28/RPS	6
GO:00072!	JNK cascade	6/272	214/1867(	0.093562	0.375097	0.323568	GADD45G/	6
GO:00070	mitotic cell cy	5/272	165/1867(	0.09398	0.37592	0.324278	ARID3A/GA	5
GO:00506	negative regul	5/272	165/1867(	0.09398	0.37592	0.324278	NKX3-1/TH	5
GO:00022	hematopoietic	5/272	166/1867(	0.095814	0.380725	0.328422	ACP6/PSM	5
GO:00019!	regulation of c	4/272	119/1867(	0.096236	0.380725	0.328422	PLAU/THB!	4
GO:00071	epidermal gro	4/272	119/1867(	0.096236	0.380725	0.328422	RPS6KA5/I	4
GO:00060	cellular glucar	3/272	75/18670	0.096398	0.380725	0.328422	PYGL/IRS2,	3
GO:00440	glucan metab	3/272	75/18670	0.096398	0.380725	0.328422	PYGL/IRS2,	3
GO:19032(	regulation of c	3/272	75/18670	0.096398	0.380725	0.328422	TXN/HIF1A	3
GO:00060(	glucose catab	2/272	36/18670	0.096472	0.380725	0.328422	PFKFB2/HK	2
GO:00068	cellular zinc io	2/272	36/18670	0.096472	0.380725	0.328422	S100A9/S1	2
GO:00327:	positive regul:	2/272	36/18670	0.096472	0.380725	0.328422	CLEC7A/TL	2
GO:00343:	Arp2/3 compl	2/272	36/18670	0.096472	0.380725	0.328422	GMFG/ARF	2
GO:19026	regulation of r	2/272	36/18670	0.096472	0.380725	0.328422	CAMK1D/E	2
GO:20005:	positive regul:	2/272	36/18670	0.096472	0.380725	0.328422	IL4R/IL18	2
GO:00019:	negative regul	10/272	429/1867(	0.098167	0.386062	0.333026	PAK2/SH3E	10
GO:00620:	regulation of s	10/272	429/1867(	0.098167	0.386062	0.333026	PTGS2/PFK	10
GO:00164	protein proce	6/272	217/1867(	0.098348	0.386062	0.333026	PLAU/MM	6
GO:00171!	regulation of e	6/272	217/1867(	0.098348	0.386062	0.333026	F2RL1/IL4F	6
GO:00160	synaptic vesic	4/272	120/1867(	0.098479	0.386062	0.333026	PFN2/RAB:	4
GO:00987:	import across	4/272	120/1867(	0.098479	0.386062	0.333026	THBS1/RG!	4
GO:00454!	cell redox hon	3/272	76/18670	0.099334	0.38867	0.335275	TXN/DLD/I	3
GO:00434	protein kinase	7/272	269/1867(	0.099363	0.38867	0.335275	TXN/NKX3	7
GO:00508(	regulation of s	6/272	218/1867(	0.099972	0.389639	0.336112	NTNG2/CD	6
GO:19030:	positive regul:	6/272	218/1867(	0.099972	0.389639	0.336112	PAK2/SIRP	6
GO:00436:	regulation of t	4/272	121/1867(	0.100746	0.389639	0.336112	PSMD12/P	4
GO:19035	regulation of A	4/272	121/1867(	0.100746	0.389639	0.336112	PFKFB2/RA	4
GO:00002	nuclear-transc	2/272	37/18670	0.101042	0.389639	0.336112	TOB1/TNR	2
GO:00709	centriole repli	2/272	37/18670	0.101042	0.389639	0.336112	CEP152/CE	2

GO:00328	regulation of p	2/272	37/18670	0.101042	0.389639	0.336112	IRS2/PPP1	2
GO:00332	response to vi	2/272	37/18670	0.101042	0.389639	0.336112	PTGS2/TRI	2
GO:00430	T cell homeos	2/272	37/18670	0.101042	0.389639	0.336112	PPP2R3C/F	2
GO:00459	positive regula	2/272	37/18670	0.101042	0.389639	0.336112	PTGS2/IRS	2
GO:00460	regulation of a	2/272	37/18670	0.101042	0.389639	0.336112	FADD/IL18	2
GO:00602	regulation of r	2/272	37/18670	0.101042	0.389639	0.336112	TACC3/RAI	2
GO:00603	head morphog	2/272	37/18670	0.101042	0.389639	0.336112	RAB3GAP1	2
GO:00605	skeletal muscl	5/272	169/18670	0.101423	0.390683	0.337012	SAP30/BIN	5
GO:00002	nuclear-transc	3/272	77/18670	0.102305	0.392799	0.338837	TOB1/EXO	3
GO:00517	interaction wi	3/272	77/18670	0.102305	0.392799	0.338837	F2RL1/CAN	3
GO:19012	positive regula	3/272	77/18670	0.102305	0.392799	0.338837	EIF2AK2/TI	3
GO:00341	erythrocyte ho	4/272	122/18670	0.103038	0.395182	0.340893	STAT1/B2N	4
GO:00072	neurotransmit	5/272	170/18670	0.103329	0.395442	0.341117	PFN2/PTPF	5
GO:00996	signal release	5/272	170/18670	0.103329	0.395442	0.341117	PFN2/PTPF	5
GO:00003	RNA splicing, v	9/272	379/18670	0.103737	0.395453	0.341127	POLR2J/US	9
GO:00003	mRNA splicing	9/272	379/18670	0.103737	0.395453	0.341127	POLR2J/US	9
GO:00466	lymphocyte pr	7/272	272/18670	0.103758	0.395453	0.341127	MNDA/TN	7
GO:00160	vesicle organiz	8/272	325/18670	0.103779	0.395453	0.341127	AKTIP/GOL	8
GO:00068	lipid transport	9/272	380/18670	0.104981	0.397107	0.342554	NKX3-1/AC	9
GO:00066	protein target	10/272	435/18670	0.105003	0.397107	0.342554	RPS28/RPS	10
GO:00069	regulation of r	5/272	171/18670	0.105252	0.397107	0.342554	PTGS2/RGS	5
GO:00106	positive regula	5/272	171/18670	0.105252	0.397107	0.342554	PTGS2/THI	5
GO:00162	regulation of r	5/272	171/18670	0.105252	0.397107	0.342554	EXOC8/CD	5
GO:00019	regulation of l	3/272	78/18670	0.105312	0.397107	0.342554	F2RL1/B2N	3
GO:00420	cytokine biosy	4/272	123/18670	0.105353	0.397107	0.342554	THBS1/CLE	4
GO:00109	regulation of r	2/272	38/18670	0.105671	0.397107	0.342554	HEBP2/FAI	2
GO:00158	neutral amino	2/272	38/18670	0.105671	0.397107	0.342554	RGS2/SLC3	2
GO:00450	early endoson	2/272	38/18670	0.105671	0.397107	0.342554	AKTIP/LMT	2
GO:00482	macrophage c	2/272	38/18670	0.105671	0.397107	0.342554	THBS1/CKL	2
GO:00516	actin filament	2/272	38/18670	0.105671	0.397107	0.342554	LMOD3/AI	2
GO:00550	zinc ion home	2/272	38/18670	0.105671	0.397107	0.342554	S100A9/S1	2
GO:00466	response to ai	8/272	327/18670	0.106491	0.399764	0.344846	TXN/STAT1	8
GO:00329	mononuclear	7/272	274/18670	0.106746	0.400296	0.345304	MNDA/TN	7
GO:00328	positive regula	5/272	172/18670	0.107193	0.401223	0.346104	GADD45G/	5
GO:00003	RNA splicing, v	9/272	382/18670	0.107494	0.401223	0.346104	POLR2J/US	9
GO:00086	apoptotic mit	4/272	124/18670	0.107692	0.401223	0.346104	IFI6/PLAUF	4
GO:00140	regulation of p	4/272	124/18670	0.107692	0.401223	0.346104	NKX3-1/F2	4
GO:00421	cytokine meta	4/272	124/18670	0.107692	0.401223	0.346104	THBS1/CLE	4
GO:00511	regulation of l	4/272	124/18670	0.107692	0.401223	0.346104	TXN/PLAU	4
GO:00190	viral life cycle	8/272	328/18670	0.107862	0.401223	0.346104	SRPK2/IFIT	8
GO:00160	organic acid ca	7/272	275/18670	0.108257	0.401223	0.346104	DLD/BCKD	7
GO:00463	carboxylic acid	7/272	275/18670	0.108257	0.401223	0.346104	DLD/BCKD	7
GO:00064	regulation of t	3/272	79/18670	0.108353	0.401223	0.346104	CDC123/EI	3
GO:00435	positive regula	3/272	79/18670	0.108353	0.401223	0.346104	PTGS2/THI	3
GO:00459	positive regula	3/272	79/18670	0.108353	0.401223	0.346104	PFKFB2/HI	3
GO:00313	positive regula	9/272	383/18670	0.108764	0.402323	0.347053	F2RL1/RAF	9
GO:00703	positive regula	5/272	173/18670	0.109152	0.402743	0.347415	GADD45G/	5
GO:00971	extrinsic apop	6/272	224/18670	0.11001	0.402743	0.347415	PAK2/THBS	6

GO:00068(	xenobiotic me	4/272	125/1867(	0.110054	0.402743	0.347415	ACSL1/NQ(	4
GO:00323(	regulation of l	4/272	125/1867(	0.110054	0.402743	0.347415	NKX3-1/TH	4
GO:00107(	fibroblast mig	2/272	39/18670	0.110356	0.402743	0.347415	THBS1/RFF	2
GO:00325(	cytokinetic pr	2/272	39/18670	0.110356	0.402743	0.347415	BIN3/ANX	2
GO:00335:	response to te	2/272	39/18670	0.110356	0.402743	0.347415	NKX3-1/TH	2
GO:00381:	neurotrophin	2/272	39/18670	0.110356	0.402743	0.347415	RAPGEF1/I	2
GO:00511{	vitamin transp	2/272	39/18670	0.110356	0.402743	0.347415	LMBRD1/F	2
GO:00717(	membrane as	2/272	39/18670	0.110356	0.402743	0.347415	PICALM/CI	2
GO:00725:	pyridine-conta	2/272	39/18670	0.110356	0.402743	0.347415	PTGS2/SLC	2
GO:00725!	maintenance (	2/272	39/18670	0.110356	0.402743	0.347415	TXN/HK2	2
GO:19020(	negative regul	2/272	39/18670	0.110356	0.402743	0.347415	RFFL/FADD	2
GO:00324!	response to lip	8/272	330/1867(	0.110633	0.402921	0.347569	RPS6KA3/F	8
GO:00427(	defense respo	8/272	330/1867(	0.110633	0.402921	0.347569	F2RL1/CLE	8
GO:00426!	muscle cell dif	9/272	385/1867(	0.111327	0.405035	0.349392	IFRD1/SCG	9
GO:00160:	rRNA metabol	6/272	225/1867(	0.111732	0.40609	0.350302	RPS28/RPS	6
GO:00018:	kidney develo	7/272	278/1867(	0.112858	0.409763	0.353471	NKX3-1/SL	7
GO:00161!	endosomal tra	6/272	226/1867(	0.113467	0.410711	0.354288	CLTCL1/LM	6
GO:00342(	negative regul	6/272	226/1867(	0.113467	0.410711	0.354288	TOB1/RBM	6
GO:00514!	positive regul	6/272	226/1867(	0.113467	0.410711	0.354288	F2RL1/PFN	6
GO:00311(	anaphase-pro	3/272	81/18670	0.114536	0.410811	0.354375	PSMD12/P	3
GO:00550:	iron ion home	3/272	81/18670	0.114536	0.410811	0.354375	B2M/HIF1	3
GO:19035:	regulation of p	3/272	81/18670	0.114536	0.410811	0.354375	UBE2D3/C	3
GO:00019!	regulation of s	2/272	40/18670	0.115095	0.410811	0.354375	F2RL1/MM	2
GO:00023(	T cell cytokine	2/272	40/18670	0.115095	0.410811	0.354375	B2M/IL18	2
GO:00076:	mating	2/272	40/18670	0.115095	0.410811	0.354375	HDAC4/AB	2
GO:00440(	modulation by	2/272	40/18670	0.115095	0.410811	0.354375	EIF2AK2/EI	2
GO:00714:	cellular respo	2/272	40/18670	0.115095	0.410811	0.354375	PTGS2/RCS	2
GO:00725{	clathrin-deper	2/272	40/18670	0.115095	0.410811	0.354375	UNC119/P	2
GO:00985:	centriole asse	2/272	40/18670	0.115095	0.410811	0.354375	CEP152/CE	2
GO:00987(	response to in	2/272	40/18670	0.115095	0.410811	0.354375	IRS2/BRWI	2
GO:00987(	cellular respo	2/272	40/18670	0.115095	0.410811	0.354375	IRS2/BRWI	2
GO:00518!	positive regul	5/272	176/1867(	0.115132	0.410811	0.354375	TXN/THBS:	5
GO:00508(	regulation of s	6/272	227/1867(	0.115217	0.410811	0.354375	NTNG2/CD	6
GO:00329:	regulation of a	9/272	388/1867(	0.115236	0.410811	0.354375	F2RL1/PFN	9
GO:00069(	striated muscl	5/272	177/1867(	0.117159	0.415997	0.358849	RGS2/TNN	5
GO:00074:	synapse assen	5/272	177/1867(	0.117159	0.415997	0.358849	NTNG2/TL	5
GO:00434{	regulation of r	5/272	177/1867(	0.117159	0.415997	0.358849	PSMD12/E	5
GO:00025:	platelet degra	4/272	128/1867(	0.117278	0.415997	0.358849	THBS1/OR	4
GO:00026{	positive regul	4/272	128/1867(	0.117278	0.415997	0.358849	F2RL1/THE	4
GO:00109:	positive regul	7/272	281/1867(	0.117562	0.416159	0.358988	RAPGEF1/	7
GO:00169:	protein sumoy	3/272	82/18670	0.117677	0.416159	0.358988	RAE1/HDA	3
GO:00462(	nitric oxide m	3/272	82/18670	0.117677	0.416159	0.358988	PTGS2/CLE	3
GO:00442(	cellular carbol	7/272	282/1867(	0.119152	0.417298	0.35997	PFKFB2/PY	7
GO:00441(	cellular amine	4/272	129/1867(	0.119731	0.417298	0.35997	PSMD12/P	4
GO:00075:	myoblast fusi	2/272	41/18670	0.119885	0.417298	0.35997	SCGB3A1/I	2
GO:00199{	translesion sy	2/272	41/18670	0.119885	0.417298	0.35997	USP10/TRI	2
GO:00308:	negative regul	2/272	41/18670	0.119885	0.417298	0.35997	LMOD3/AI	2
GO:00341:	negative regul	2/272	41/18670	0.119885	0.417298	0.35997	F2RL1/LY9	2

GO:00353:	fatty-acyl-CoA	2/272	41/18670	0.119885	0.417298	0.35997	ACSL1/HSD	2
GO:00434(	skeletal muscl	2/272	41/18670	0.119885	0.417298	0.35997	IFRD1/BIN:	2
GO:00507!	activated T ce	2/272	41/18670	0.119885	0.417298	0.35997	FADD/IL18	2
GO:00508:	brown fat cell	2/272	41/18670	0.119885	0.417298	0.35997	PTGS2/RG!	2
GO:00703:	negative regul	2/272	41/18670	0.119885	0.417298	0.35997	EPC1/CBX3	2
GO:00902:	regulation of s	2/272	41/18670	0.119885	0.417298	0.35997	TACC3/RAI	2
GO:00903:	negative regul	2/272	41/18670	0.119885	0.417298	0.35997	TXN/LRRK2	2
GO:00989:	vesicle-mediat	2/272	41/18670	0.119885	0.417298	0.35997	AKTIP/LMT	2
GO:19001:	regulation of e	2/272	41/18670	0.119885	0.417298	0.35997	PAK2/RFFL	2
GO:19028!	regulation of p	2/272	41/18670	0.119885	0.417298	0.35997	HIF1A/HDA	2
GO:00093:	response to ra	10/272	448/1867(	0.120733	0.419418	0.3618	TXN/RGR/U	10
GO:19018:	regulation of p	3/272	83/18670	0.120849	0.419418	0.3618	F2RL1/LM(	3
GO:20001(	regulation of l	3/272	83/18670	0.120849	0.419418	0.3618	FADD/HIF1	3
GO:00105(	regulation of c	5/272	180/1867(	0.123343	0.427235	0.368542	PTGS2/PSN	5
GO:00713	cellular respor	5/272	180/1867(	0.123343	0.427235	0.368542	IFNGR1/ST	5
GO:00714!	cellular respor	8/272	339/1867(	0.123578	0.427415	0.368698	PTGS2/GA	8
GO:00002	spliceosomal c	3/272	84/18670	0.124053	0.427415	0.368698	SRPK2/PSI	3
GO:00027:	regulation of c	3/272	84/18670	0.124053	0.427415	0.368698	F2RL1/B2N	3
GO:00508&	endocrine pro	3/272	84/18670	0.124053	0.427415	0.368698	NKX3-1/F2	3
GO:00026:	positive regula	2/272	42/18670	0.124723	0.427415	0.368698	IL4R/EXOS	2
GO:00305:	negative regul	2/272	42/18670	0.124723	0.427415	0.368698	IFRD1/CDK	2
GO:00440&	regulation of v	2/272	42/18670	0.124723	0.427415	0.368698	RAB3GAP1	2
GO:00484&	synaptic vesicl	2/272	42/18670	0.124723	0.427415	0.368698	LRRK2/PIC	2
GO:00511!	negative regul	2/272	42/18670	0.124723	0.427415	0.368698	RGS2/HDA	2
GO:19010:	regulation of r	2/272	42/18670	0.124723	0.427415	0.368698	TXN/LRRK2	2
GO:19031	regulation of a	2/272	42/18670	0.124723	0.427415	0.368698	HK2/HIF1A	2
GO:00024	production of	7/272	286/1867(	0.125625	0.428832	0.36992	F2RL1/IL4F	7
GO:00514(	stress-activate	7/272	286/1867(	0.125625	0.428832	0.36992	GADD45G/	7
GO:00313:	negative regul	4/272	132/1867(	0.127223	0.428832	0.36992	PFN2/TMS	4
GO:20010!	reactive nitrog	3/272	85/18670	0.127288	0.428832	0.36992	PTGS2/CLE	3
GO:00019!	negative regul	2/272	43/18670	0.129608	0.428832	0.36992	THBS1/MA	2
GO:00030(	skeletal muscl	2/272	43/18670	0.129608	0.428832	0.36992	TNNI2/RCS	2
GO:00328&	regulation of p	2/272	43/18670	0.129608	0.428832	0.36992	IRS2/PPP1	2
GO:00341!	cellular respor	2/272	43/18670	0.129608	0.428832	0.36992	EIF2AK2/EI	2
GO:00420&	T-helper 1 typ	2/272	43/18670	0.129608	0.428832	0.36992	IL4R/IL18	2
GO:00454:	positive regula	2/272	43/18670	0.129608	0.428832	0.36992	PTGS2/CLE	2
GO:20010:	regulation of c	2/272	43/18670	0.129608	0.428832	0.36992	TXN/LRRK2	2
GO:00434&	regulation of I	5/272	183/1867(	0.129675	0.428832	0.36992	PSMD12/E	5
GO:00064(	negative regul	6/272	235/1867(	0.129693	0.428832	0.36992	PAK2/SH3I	6
GO:00086:	extrinsic apop	3/272	86/18670	0.130552	0.428832	0.36992	THBS1/RFF	3
GO:00420!	regulation of e	3/272	86/18670	0.130552	0.428832	0.36992	IFI6/PLAUF	3
GO:00424	hormone bios	3/272	86/18670	0.130552	0.428832	0.36992	HSD17B11	3
GO:00480:	ephrin recept	3/272	86/18670	0.130552	0.428832	0.36992	CDK5R1/A	3
GO:00602!	long-term syn	3/272	86/18670	0.130552	0.428832	0.36992	MME/EIF2	3
GO:00705	response to fa	3/272	86/18670	0.130552	0.428832	0.36992	PTGS2/AC!	3
GO:00107:	negative regul	8/272	344/1867(	0.1311	0.428832	0.36992	KIAA0319/	8
GO:00070:	actin filament	9/272	400/1867(	0.131616	0.428832	0.36992	F2RL1/PFN	9
GO:20000	regulation of (	5/272	184/1867(	0.131819	0.428832	0.36992	ARID3A/G/	5

GO:00026!	positive regul	3/272	87/18670	0.133846	0.428832	0.36992	F2RL1/THE	3
GO:00061	energy reserv	3/272	87/18670	0.133846	0.428832	0.36992	PYGL/IRS2,	3
GO:00106	regulation of	7/272	291/1867C	0.133962	0.428832	0.36992	PTGS2/THI	7
GO:00061!	purine nucleo	2/272	44/18670	0.134536	0.428832	0.36992	PDE7A/NT	2
GO:00104(	mesenchymal	2/272	44/18670	0.134536	0.428832	0.36992	STAT1/IRS	2
GO:00140(	astrocyte dev	2/272	44/18670	0.134536	0.428832	0.36992	IFNGR1/S1	2
GO:00358!	epithelial cell	2/272	44/18670	0.134536	0.428832	0.36992	STAT1/MN	2
GO:00429	amyloid precu	2/272	44/18670	0.134536	0.428832	0.36992	IFNGR1/PI	2
GO:00440(	regulation of	2/272	44/18670	0.134536	0.428832	0.36992	NKX3-1/F2	2
GO:00440!	membrane bi	2/272	44/18670	0.134536	0.428832	0.36992	PICALM/CI	2
GO:00610(	regulation of	2/272	44/18670	0.134536	0.428832	0.36992	CDK5R1/LI	2
GO:00903	regulation of	2/272	44/18670	0.134536	0.428832	0.36992	JDP2/LRRK	2
GO:19032	regulation of	2/272	44/18670	0.134536	0.428832	0.36992	UBE2D3/LI	2
GO:19044(	positive regul	2/272	44/18670	0.134536	0.428832	0.36992	PTGS2/CLE	2
GO:00094	response to x	7/272	292/1867C	0.135661	0.428832	0.36992	ACSL1/RGS	7
GO:00016(	fever generati	1/272	10/18670	0.13653	0.428832	0.36992	PTGS2	1
GO:00017	natural killer	1/272	10/18670	0.13653	0.428832	0.36992	IL18	1
GO:00065	histidine meta	1/272	10/18670	0.13653	0.428832	0.36992	HAL	1
GO:00107	regulation of	1/272	10/18670	0.13653	0.428832	0.36992	THBS1	1
GO:00140	regulation of	1/272	10/18670	0.13653	0.428832	0.36992	LRRK2	1
GO:00140	positive regul	1/272	10/18670	0.13653	0.428832	0.36992	RAB3GAP1	1
GO:00148	response to m	1/272	10/18670	0.13653	0.428832	0.36992	HDAC4	1
GO:00148!	response to d	1/272	10/18670	0.13653	0.428832	0.36992	HDAC4	1
GO:00158(	aromatic amir	1/272	10/18670	0.13653	0.428832	0.36992	SLC36A4	1
GO:00215	pons develop	1/272	10/18670	0.13653	0.428832	0.36992	CDK5R1	1
GO:00320(	positive regul	1/272	10/18670	0.13653	0.428832	0.36992	IRS2	1
GO:00323	alanine transp	1/272	10/18670	0.13653	0.428832	0.36992	SLC36A4	1
GO:00326	interleukin-18	1/272	10/18670	0.13653	0.428832	0.36992	TLR2	1
GO:00328	glomerular ba	1/272	10/18670	0.13653	0.428832	0.36992	SULF2	1
GO:00331	regulation of	1/272	10/18670	0.13653	0.428832	0.36992	PFKFB2	1
GO:00334	gas homeosta	1/272	10/18670	0.13653	0.428832	0.36992	HIF1A	1
GO:00344	nuclear-trans	1/272	10/18670	0.13653	0.428832	0.36992	EXOSC3	1
GO:00347	histone H4-K2	1/272	10/18670	0.13653	0.428832	0.36992	ARID4A	1
GO:00355	monoubiquiti	1/272	10/18670	0.13653	0.428832	0.36992	USP15	1
GO:00420	olfactory beha	1/272	10/18670	0.13653	0.428832	0.36992	CHD7	1
GO:00427!	long-chain fat	1/272	10/18670	0.13653	0.428832	0.36992	CYP4F3	1
GO:00429	sequestering	1/272	10/18670	0.13653	0.428832	0.36992	TMSB4Y	1
GO:00434	regulation of	1/272	10/18670	0.13653	0.428832	0.36992	PPP1R3D	1
GO:00445!	relaxation of s	1/272	10/18670	0.13653	0.428832	0.36992	RGS2	1
GO:00456	regulation of	1/272	10/18670	0.13653	0.428832	0.36992	IL4R	1
GO:00459	positive regul	1/272	10/18670	0.13653	0.428832	0.36992	CHD8	1
GO:00481(	autophagic ce	1/272	10/18670	0.13653	0.428832	0.36992	EIF2AK4	1
GO:00483	mesendoderm	1/272	10/18670	0.13653	0.428832	0.36992	EOMES	1
GO:00487!	branching mo	1/272	10/18670	0.13653	0.428832	0.36992	LRRK2	1
GO:00510	actin filament	1/272	10/18670	0.13653	0.428832	0.36992	GMFG	1
GO:00511	regulation of	1/272	10/18670	0.13653	0.428832	0.36992	IL18	1
GO:00519	negative regul	1/272	10/18670	0.13653	0.428832	0.36992	THBS1	1
GO:00526	alditol phosph	1/272	10/18670	0.13653	0.428832	0.36992	ACP6	1

GO:00607(	regulation of e	1/272	10/18670	0.13653	0.428832	0.36992	NKX3-1	1
GO:00700!	regulation of c	1/272	10/18670	0.13653	0.428832	0.36992	HIF1A	1
GO:00703!	response to lip	1/272	10/18670	0.13653	0.428832	0.36992	TLR2	1
GO:00704:	regulation of u	1/272	10/18670	0.13653	0.428832	0.36992	ABAT	1
GO:00709	neutrophil me	1/272	10/18670	0.13653	0.428832	0.36992	F2RL1	1
GO:00712:	cellular respor	1/272	10/18670	0.13653	0.428832	0.36992	TLR2	1
GO:00712{	cellular respor	1/272	10/18670	0.13653	0.428832	0.36992	B2M	1
GO:00722(	cell proliferati	1/272	10/18670	0.13653	0.428832	0.36992	STAT1	1
GO:00903:	positive regula	1/272	10/18670	0.13653	0.428832	0.36992	PTGS2	1
GO:01060	regulation of c	1/272	10/18670	0.13653	0.428832	0.36992	PTGS2	1
GO:19002:	negative regul	1/272	10/18670	0.13653	0.428832	0.36992	IFNGR1	1
GO:19004:	positive regula	1/272	10/18670	0.13653	0.428832	0.36992	F2RL1	1
GO:19017:	regulation of h	1/272	10/18670	0.13653	0.428832	0.36992	LRRK2	1
GO:19020:	regulation of h	1/272	10/18670	0.13653	0.428832	0.36992	EIF2AK2	1
GO:19046{	negative regul	1/272	10/18670	0.13653	0.428832	0.36992	PICALM	1
GO:19902!	cellular respor	1/272	10/18670	0.13653	0.428832	0.36992	EIF2AK4	1
GO:20000:	regulation of s	1/272	10/18670	0.13653	0.428832	0.36992	EVI2B	1
GO:20004{	negative regul	1/272	10/18670	0.13653	0.428832	0.36992	PRKAR2A	1
GO:20006	regulation of r	1/272	10/18670	0.13653	0.428832	0.36992	DTX3L	1
GO:20006(	regulation of i	1/272	10/18670	0.13653	0.428832	0.36992	IL17RA	1
GO:20008:	positive regula	1/272	10/18670	0.13653	0.428832	0.36992	NKX3-1	1
GO:20010!	negative regul	1/272	10/18670	0.13653	0.428832	0.36992	HIF1A	1
GO:20012:	positive regula	1/272	10/18670	0.13653	0.428832	0.36992	RAP1A	1
GO:00090(	aerobic respir	3/272	88/18670	0.137169	0.428832	0.36992	DLD/HIF1A	3
GO:00421	retrograde tra	3/272	88/18670	0.137169	0.428832	0.36992	CLTCL1/ST.	3
GO:00610!	regulation of p	3/272	88/18670	0.137169	0.428832	0.36992	PAK2/UNC	3
GO:19004(	regulation of c	3/272	88/18670	0.137169	0.428832	0.36992	TXN/HIF1A	3
GO:00514(	neuron apopt	6/272	239/1867(	0.137245	0.428832	0.36992	SRPK2/NQ	6
GO:00709!	neuron death	8/272	348/1867(	0.137285	0.428832	0.36992	SRPK2/NQ	8
GO:00511	striated muscl	7/272	293/1867(	0.137372	0.428832	0.36992	SCGB3A1/I	7
GO:00720(	renal system c	7/272	293/1867(	0.137372	0.428832	0.36992	NKX3-1/SU	7
GO:00072!	female gamet	4/272	136/1867(	0.137508	0.428879	0.369961	PTGS2/IL4I	4
GO:00481(	regulation of s	5/272	187/1867(	0.138344	0.431107	0.371882	PTGS2/MN	5
GO:00435	positive regula	9/272	405/1867(	0.138786	0.431497	0.372219	F2RL1/RAF	9
GO:00324!	endocytic recy	2/272	45/18670	0.139506	0.431497	0.372219	LMTK2/RA	2
GO:00325:	response to pr	2/272	45/18670	0.139506	0.431497	0.372219	THBS1/TLF	2
GO:00359{	endodermal c	2/272	45/18670	0.139506	0.431497	0.372219	EOMES/MI	2
GO:00442:	cellular carbol	2/272	45/18670	0.139506	0.431497	0.372219	PYGL/PPP1	2
GO:00703:	regulation of (	2/272	45/18670	0.139506	0.431497	0.372219	EPC1/CBX3	2
GO:19027	regulation of l	2/272	45/18670	0.139506	0.431497	0.372219	BIN3/HDA(	2
GO:00106:	regulation of c	4/272	137/1867(	0.14013	0.431497	0.372219	PFKFB2/IR!	4
GO:00987!	detoxification	4/272	137/1867(	0.14013	0.431497	0.372219	TXN/PTGS:	4
GO:00064!	protein lipidat	3/272	89/18670	0.140519	0.431497	0.372219	PPM1B/RA	3
GO:00712	cellular respor	5/272	188/1867(	0.14055	0.431497	0.372219	PTGS2/B2M	5
GO:00507(	negative regul	7/272	295/1867(	0.140824	0.431497	0.372219	KIAA0319/	7
GO:00018:	negative regul	7/272	296/1867(	0.142566	0.431497	0.372219	RPS6KA5/F	7
GO:00093(	amine metabo	4/272	138/1867(	0.142773	0.431497	0.372219	PSMD12/P	4
GO:00106:	epithelial cell	8/272	352/1867(	0.143615	0.431497	0.372219	PTGS2/THI	8

GO:00310!	positive regul:	3/272	90/18670	0.143897	0.431497	0.372219	RPS6KA5/J	3
GO:00326!	regulation of i	3/272	90/18670	0.143897	0.431497	0.372219	MNDA/CLE	3
GO:00024:	inflammatory	2/272	46/18670	0.144515	0.431497	0.372219	CYSLTR1/P	2
GO:00059:	glycogen bios	2/272	46/18670	0.144515	0.431497	0.372219	IRS2/PPP1	2
GO:00092!	glucan biosyn	2/272	46/18670	0.144515	0.431497	0.372219	IRS2/PPP1	2
GO:00108{	regulation of l	2/272	46/18670	0.144515	0.431497	0.372219	NFKBIA/OS	2
GO:00304!	maturation of	2/272	46/18670	0.144515	0.431497	0.372219	RPS28/RPS	2
GO:00323(	negative regul	2/272	46/18670	0.144515	0.431497	0.372219	THBS1/IRS	2
GO:00324{	negative regul	2/272	46/18670	0.144515	0.431497	0.372219	PPM1B/TR	2
GO:00433(	regulation of l	2/272	46/18670	0.144515	0.431497	0.372219	F2RL1/IL4F	2
GO:00433:	response to e:	2/272	46/18670	0.144515	0.431497	0.372219	IFIT1/NFKE	2
GO:00459{	positive regul:	2/272	46/18670	0.144515	0.431497	0.372219	PFKFB2/HI	2
GO:19000:	regulation of (	2/272	46/18670	0.144515	0.431497	0.372219	CLEC7A/IL:	2
GO:19005	positive regul:	2/272	46/18670	0.144515	0.431497	0.372219	PFKFB2/HI	2
GO:19909:	response to ai	2/272	46/18670	0.144515	0.431497	0.372219	EIF2AK2/EI	2
GO:00343:	cell junction a	9/272	409/1867(	0.144664	0.431497	0.372219	NTNG2/RA	9
GO:19029(	regulation of s	8/272	353/1867(	0.14522	0.431497	0.372219	F2RL1/PFN	8
GO:00900(	positive regul:	7/272	298/1867(	0.146081	0.431497	0.372219	ARID3A/G/	7
GO:00313:	positive regul:	6/272	244/1867(	0.146969	0.431497	0.372219	CLEC7A/PF	6
GO:00518!	regulation of p	6/272	244/1867(	0.146969	0.431497	0.372219	TXN/RAPG	6
GO:00986!	import into ce	5/272	191/1867(	0.147261	0.431497	0.372219	ACSL1/THE	5
GO:00149(	smooth muscl	3/272	91/18670	0.147302	0.431497	0.372219	PLAU/HDA	3
GO:00488:	regulation of (	3/272	91/18670	0.147302	0.431497	0.372219	CDK5R1/H	3
GO:19040(	negative regul	3/272	91/18670	0.147302	0.431497	0.372219	RGS2/HEC'	3
GO:00074(	axonogenesis	10/272	468/1867(	0.147329	0.431497	0.372219	RPS6KA5/I	10
GO:00463:	positive regul:	4/272	140/1867(	0.148117	0.431497	0.372219	GADD45G/	4
GO:00901:	epithelium mi	8/272	355/1867(	0.148456	0.431497	0.372219	PTGS2/THI	8
GO:00083{	RNA splicing	10/272	469/1867(	0.148733	0.431497	0.372219	POLR2J/US	10
GO:00026(	regulation of (	1/272	11/18670	0.149116	0.431497	0.372219	THBS1	1
GO:00030!	regulation of g	1/272	11/18670	0.149116	0.431497	0.372219	F2RL1	1
GO:00068!	carnitine shut	1/272	11/18670	0.149116	0.431497	0.372219	SLC25A20	1
GO:00101:	embryonic bo	1/272	11/18670	0.149116	0.431497	0.372219	ZNF281	1
GO:00148:	response to m	1/272	11/18670	0.149116	0.431497	0.372219	HDAC4	1
GO:00190	viral latency	1/272	11/18670	0.149116	0.431497	0.372219	PSIP1	1
GO:00193:	cyclooxygenas	1/272	11/18670	0.149116	0.431497	0.372219	PTGS2	1
GO:00195:	oxalate transp	1/272	11/18670	0.149116	0.431497	0.372219	SLC26A8	1
GO:00319:	positive regul:	1/272	11/18670	0.149116	0.431497	0.372219	PTGS2	1
GO:00329(	regulation of t	1/272	11/18670	0.149116	0.431497	0.372219	THBS1	1
GO:00332:	positive regul:	1/272	11/18670	0.149116	0.431497	0.372219	HDAC4	1
GO:00354(	histone-serine	1/272	11/18670	0.149116	0.431497	0.372219	RPS6KA5	1
GO:00364(	TRAIL-activate	1/272	11/18670	0.149116	0.431497	0.372219	FADD	1
GO:00436:	regulation of t	1/272	11/18670	0.149116	0.431497	0.372219	HIF1A	1
GO:00465:	sphingosine bi	1/272	11/18670	0.149116	0.431497	0.372219	ASAH1	1
GO:00468{	positive regul:	1/272	11/18670	0.149116	0.431497	0.372219	HIF1A	1
GO:00601!	regulation of (	1/272	11/18670	0.149116	0.431497	0.372219	LRRK2	1
GO:00607(	epithelial cell	1/272	11/18670	0.149116	0.431497	0.372219	NKX3-1	1
GO:00701(	interleukin-27	1/272	11/18670	0.149116	0.431497	0.372219	STAT1	1
GO:00704:	uterine smoot	1/272	11/18670	0.149116	0.431497	0.372219	ABAT	1

GO:00706:	response to in	1/272	11/18670	0.149116	0.431497	0.372219	IL18	1
GO:00707!	interleukin-35	1/272	11/18670	0.149116	0.431497	0.372219	STAT1	1
GO:00709:	histone H4 de	1/272	11/18670	0.149116	0.431497	0.372219	HDAC4	1
GO:00725:	fibroblast acti	1/272	11/18670	0.149116	0.431497	0.372219	IL17RA	1
GO:00725{	caveolin-medi	1/272	11/18670	0.149116	0.431497	0.372219	UNC119	1
GO:00726(	interleukin-10	1/272	11/18670	0.149116	0.431497	0.372219	F2RL1	1
GO:00726:	interleukin-13	1/272	11/18670	0.149116	0.431497	0.372219	IL17RA	1
GO:00726{	T cell extravas	1/272	11/18670	0.149116	0.431497	0.372219	FADD	1
GO:00903!	negative regul	1/272	11/18670	0.149116	0.431497	0.372219	LRRK2	1
GO:19015(	fatty acid deri	1/272	11/18670	0.149116	0.431497	0.372219	CYP4F3	1
GO:19029⁄	protein localiz	1/272	11/18670	0.149116	0.431497	0.372219	DTX3L	1
GO:19032!	regulation of I	1/272	11/18670	0.149116	0.431497	0.372219	PFKFB2	1
GO:19039{	positive regul:	1/272	11/18670	0.149116	0.431497	0.372219	LRRK2	1
GO:19043:	response to fc	1/272	11/18670	0.149116	0.431497	0.372219	AHR	1
GO:19043:	cellular respoı	1/272	11/18670	0.149116	0.431497	0.372219	AHR	1
GO:19052⁄	regulation of ä	1/272	11/18670	0.149116	0.431497	0.372219	PICALM	1
GO:19056(	positive regul:	1/272	11/18670	0.149116	0.431497	0.372219	DTX3L	1
GO:20007{	positive regul:	1/272	11/18670	0.149116	0.431497	0.372219	RAB3GAP1	1
GO:00075(	female pregna	5/272	192/1867(	0.149528	0.431497	0.372219	PTGS2/PS(	5
GO:00069!	acute-phase r	2/272	47/18670	0.149561	0.431497	0.372219	PTGS2/ORI	2
GO:00181!	peptidyl-cyste	2/272	47/18670	0.149561	0.431497	0.372219	S100A9/S1	2
GO:00357:	interleukin-12	2/272	47/18670	0.149561	0.431497	0.372219	PAK2/LMN	2
GO:00433:	regulation of (	2/272	47/18670	0.149561	0.431497	0.372219	IL4R/IL18	2
GO:00466:	positive regul:	2/272	47/18670	0.149561	0.431497	0.372219	IL4R/IL18	2
GO:00616:	pri-miRNA tra	2/272	47/18670	0.149561	0.431497	0.372219	HIF1A/HD/	2
GO:00702:	T cell apoptoti	2/272	47/18670	0.149561	0.431497	0.372219	FADD/HIF1	2
GO:19007⁄	regulation of ı	2/272	47/18670	0.149561	0.431497	0.372219	GADD45G/	2
GO:19035{	positive regul:	2/272	47/18670	0.149561	0.431497	0.372219	PFKFB2/HI	2
GO:00068(	amino acid tra	4/272	141/1867(	0.150817	0.43406	0.37443	RGS2/RAB:	4
GO:00094:	response to U	4/272	141/1867(	0.150817	0.43406	0.37443	PTGS2/MN	4
GO:00620:	positive regul:	4/272	141/1867(	0.150817	0.43406	0.37443	PTGS2/PFk	4
GO:00512:	negative regul	5/272	193/1867(	0.15181	0.434153	0.37451	TXN/F2RL1	5
GO:19016!	response to ke	5/272	193/1867(	0.15181	0.434153	0.37451	NKX3-1/TH	5
GO:00972:	cellular respoı	6/272	247/1867(	0.15295	0.434153	0.37451	TXN/PTGS:	6
GO:00381:	ERBB signaling	4/272	142/1867(	0.153537	0.434153	0.37451	RPS6KA5/I	4
GO:00108:	lipid localizati	9/272	415/1867(	0.153716	0.434153	0.37451	NKX3-1/A(	9
GO:00109:	negative regul	3/272	93/18670	0.154189	0.434153	0.37451	PSMD12/P	3
GO:00364:	cell death in r	3/272	93/18670	0.154189	0.434153	0.37451	TXN/HIF1A	3
GO:00519!	regulation of ä	3/272	93/18670	0.154189	0.434153	0.37451	RGS2/RAB:	3
GO:19011{	regulation of I	3/272	93/18670	0.154189	0.434153	0.37451	IFI6/PLAUI	3
GO:00075!	lactation	2/272	48/18670	0.154643	0.434153	0.37451	HK2/HIF1A	2
GO:00080:	neuron recogr	2/272	48/18670	0.154643	0.434153	0.37451	CDK5R1/N	2
GO:00085⁄	visual learning	2/272	48/18670	0.154643	0.434153	0.37451	HIF1A/NPT	2
GO:00326⁄	regulation of i	2/272	48/18670	0.154643	0.434153	0.37451	PPM1B/TL	2
GO:00458(	negative regul	2/272	48/18670	0.154643	0.434153	0.37451	UNC119/P	2
GO:00610:	positive regul:	2/272	48/18670	0.154643	0.434153	0.37451	TOB1/TNR	2
GO:00901!	regulation of ı	2/272	48/18670	0.154643	0.434153	0.37451	PLAUR/MN	2
GO:00507(	positive regul:	10/272	474/1867(	0.155854	0.434153	0.37451	MME/RAP(	10

GO:00066(	protein impor	4/272	143/1867(	0.156275	0.434153	0.37451	PTGS2/RPL	4
GO:00443	cellular respor	4/272	143/1867(	0.156275	0.434153	0.37451	POLR2J/SU	4
GO:01061(	cold-induced t	4/272	143/1867(	0.156275	0.434153	0.37451	GADD45G/	4
GO:01201(	regulation of c	4/272	143/1867(	0.156275	0.434153	0.37451	GADD45G/	4
GO:00460	ATP metabolic	7/272	304/1867(	0.15687	0.434153	0.37451	DLD/PFKFE	7
GO:00421	lipoprotein bi	3/272	94/18670	0.157671	0.434153	0.37451	PPM1B/RA	3
GO:00456	positive regula	3/272	94/18670	0.157671	0.434153	0.37451	PPP2R3C/I	3
GO:00512	protein homo	3/272	94/18670	0.157671	0.434153	0.37451	UPB1/B2M	3
GO:00901	tissue migratio	8/272	361/1867(	0.158374	0.434153	0.37451	PTGS2/THI	8
GO:00026	regulation of l	5/272	196/1867(	0.158742	0.434153	0.37451	F2RL1/THE	5
GO:00019	T cell mediate	2/272	49/18670	0.159757	0.434153	0.37451	B2M/FADD	2
GO:00025	cytokine prod	2/272	49/18670	0.159757	0.434153	0.37451	CLEC7A/IL	2
GO:00030	regulation of s	2/272	49/18670	0.159757	0.434153	0.37451	F2RL1/MM	2
GO:00308	negative regul	2/272	49/18670	0.159757	0.434153	0.37451	STAT1/MM	2
GO:00342	negative regul	2/272	49/18670	0.159757	0.434153	0.37451	PTPRN2/LF	2
GO:00350	response to ni	2/272	49/18670	0.159757	0.434153	0.37451	B2M/ABAT	2
GO:00713	cellular respor	2/272	49/18670	0.159757	0.434153	0.37451	PAK2/LMN	2
GO:19047(	positive regula	2/272	49/18670	0.159757	0.434153	0.37451	NQO2/MM	2
GO:00104	proteasomal p	10/272	477/1867(	0.160207	0.434153	0.37451	UBE2D3/P	10
GO:19049	negative regul	5/272	197/1867(	0.161081	0.434153	0.37451	TXN/F2RL1	5
GO:00421(	positive regula	3/272	95/18670	0.161176	0.434153	0.37451	TNFSF13B/	3
GO:00977	ciliary basal b	3/272	95/18670	0.161176	0.434153	0.37451	CEP152/TU	3
GO:00020(	regulation of a	1/272	12/18670	0.16152	0.434153	0.37451	MME	1
GO:00020(	angiotensin m	1/272	12/18670	0.16152	0.434153	0.37451	MME	1
GO:00024	dendritic cell a	1/272	12/18670	0.16152	0.434153	0.37451	THBS1	1
GO:00028(	negative regul	1/272	12/18670	0.16152	0.434153	0.37451	PSMA1	1
GO:00029(	negative regul	1/272	12/18670	0.16152	0.434153	0.37451	IRS2	1
GO:00033	mesenchymal	1/272	12/18670	0.16152	0.434153	0.37451	STAT1	1
GO:00065	arginine catab	1/272	12/18670	0.16152	0.434153	0.37451	PADI4	1
GO:00065	lysine cataboli	1/272	12/18670	0.16152	0.434153	0.37451	DLD	1
GO:00066(	glycerol ether	1/272	12/18670	0.16152	0.434153	0.37451	TXN	1
GO:00108	regulation of s	1/272	12/18670	0.16152	0.434153	0.37451	OSBPL8	1
GO:00148	gastro-intestir	1/272	12/18670	0.16152	0.434153	0.37451	SULF2	1
GO:00156	quaternary an	1/272	12/18670	0.16152	0.434153	0.37451	SLC25A20	1
GO:00158	glycine transp	1/272	12/18670	0.16152	0.434153	0.37451	RGS2	1
GO:00161	synaptic vesicl	1/272	12/18670	0.16152	0.434153	0.37451	PICALM	1
GO:00218	layer formatio	1/272	12/18670	0.16152	0.434153	0.37451	CDK5R1	1
GO:00311	snoRNA 3'-enc	1/272	12/18670	0.16152	0.434153	0.37451	EXOSC3	1
GO:00313	regulation of p	1/272	12/18670	0.16152	0.434153	0.37451	PTGS2	1
GO:00322	secretory grar	1/272	12/18670	0.16152	0.434153	0.37451	KIF1B	1
GO:00329(	transforming g	1/272	12/18670	0.16152	0.434153	0.37451	THBS1	1
GO:00335	histone H2B u	1/272	12/18670	0.16152	0.434153	0.37451	DTX3L	1
GO:00350	sperm chroma	1/272	12/18670	0.16152	0.434153	0.37451	SRPK1	1
GO:00357	regulation of l	1/272	12/18670	0.16152	0.434153	0.37451	LRRK2	1
GO:00360(	response to m	1/272	12/18670	0.16152	0.434153	0.37451	TLR2	1
GO:00360(	cellular respor	1/272	12/18670	0.16152	0.434153	0.37451	TLR2	1
GO:00380(	opioid recepto	1/272	12/18670	0.16152	0.434153	0.37451	OGFRL1	1
GO:00454	positive regula	1/272	12/18670	0.16152	0.434153	0.37451	CLEC7A	1

GO:00455:	positive regula	1/272	12/18670	0.16152	0.434153	0.37451	PPP2R3C	1
GO:00465:	sphingoid bios	1/272	12/18670	0.16152	0.434153	0.37451	ASAH1	1
GO:00469:	negative regul	1/272	12/18670	0.16152	0.434153	0.37451	RAP1A	1
GO:00510!	regulation of I	1/272	12/18670	0.16152	0.434153	0.37451	IFIT1	1
GO:00511:	NK T cell activ	1/272	12/18670	0.16152	0.434153	0.37451	IL18	1
GO:00528(	imidazole-con	1/272	12/18670	0.16152	0.434153	0.37451	HAL	1
GO:00600:	radial glial cell	1/272	12/18670	0.16152	0.434153	0.37451	CDH2	1
GO:00603!	negative regul	1/272	12/18670	0.16152	0.434153	0.37451	PBLD	1
GO:00604	branching invo	1/272	12/18670	0.16152	0.434153	0.37451	NKX3-1	1
GO:00702	regulation of t	1/272	12/18670	0.16152	0.434153	0.37451	HIF1A	1
GO:00704!	thrombin-acti	1/272	12/18670	0.16152	0.434153	0.37451	F2RL1	1
GO:00711(	response to pa	1/272	12/18670	0.16152	0.434153	0.37451	HDAC4	1
GO:00716:	positive regula	1/272	12/18670	0.16152	0.434153	0.37451	CLEC7A	1
GO:00903:	negative regul	1/272	12/18670	0.16152	0.434153	0.37451	ABAT	1
GO:00971(	postsynaptic r	1/272	12/18670	0.16152	0.434153	0.37451	CDH2	1
GO:00988	modification c	1/272	12/18670	0.16152	0.434153	0.37451	PFN2	1
GO:00995!	trans-synaptic	1/272	12/18670	0.16152	0.434153	0.37451	NPTN	1
GO:01500(	regulation of c	1/272	12/18670	0.16152	0.434153	0.37451	LRRK2	1
GO:19046(	positive regula	1/272	12/18670	0.16152	0.434153	0.37451	GOLGA2	1
GO:19056(	regulation of p	1/272	12/18670	0.16152	0.434153	0.37451	DTX3L	1
GO:20006!	negative regul	1/272	12/18670	0.16152	0.434153	0.37451	HECW2	1
GO:20010!	regulation of r	1/272	12/18670	0.16152	0.434153	0.37451	HIF1A	1
GO:00430	regulation of (	10/272	479/1867(	0.163143	0.437759	0.377621	F2RL1/RAF	10
GO:00305:	regulation of a	3/272	96/18670	0.164705	0.437759	0.377621	IFRD1/TNF	3
GO:00480:	vascular endo	3/272	96/18670	0.164705	0.437759	0.377621	PAK2/HIF1	3
GO:19908:	response to le	3/272	96/18670	0.164705	0.437759	0.377621	TLE4/HK2/	3
GO:19908:	cellular respor	3/272	96/18670	0.164705	0.437759	0.377621	TLE4/HK2/	3
GO:00017(	endoderm for	2/272	50/18670	0.164902	0.437759	0.377621	EOMES/MI	2
GO:00019(	positive regula	2/272	50/18670	0.164902	0.437759	0.377621	FADD/HIF1	2
GO:00706:	response to in	2/272	50/18670	0.164902	0.437759	0.377621	PAK2/LMN	2
GO:19037!	positive regula	2/272	50/18670	0.164902	0.437759	0.377621	RAB3GAP1	2
GO:00343	response to in	5/272	199/1867(	0.165802	0.437759	0.377621	IFNGR1/ST	5
GO:00022(	myeloid cell h	4/272	147/1867(	0.167406	0.437759	0.377621	STAT1/B2N	4
GO:00712:	cellular respor	4/272	147/1867(	0.167406	0.437759	0.377621	TXN/RPL23	4
GO:00609!	dendritic spin	3/272	97/18670	0.168257	0.437759	0.377621	PAK2/CDK!	3
GO:19028	regulation of r	3/272	97/18670	0.168257	0.437759	0.377621	TXN/HIF1A	3
GO:20010:	positive regula	3/272	97/18670	0.168257	0.437759	0.377621	NKX3-1/RF	3
GO:00067:	coenzyme me	6/272	255/1867(	0.169416	0.437759	0.377621	DLD/PTGS:	6
GO:00224(	positive regula	6/272	255/1867(	0.169416	0.437759	0.377621	PAK2/SIRP	6
GO:00007:	DNA synthesis	2/272	51/18670	0.170075	0.437759	0.377621	USP10/TRI	2
GO:00067:	oxidoreductio	2/272	51/18670	0.170075	0.437759	0.377621	PTGS2/SLC	2
GO:00075(	embryo impla	2/272	51/18670	0.170075	0.437759	0.377621	PTGS2/MN	2
GO:00196:	NAD metaboli	2/272	51/18670	0.170075	0.437759	0.377621	HK2/BST1	2
GO:00456(	negative regul	2/272	51/18670	0.170075	0.437759	0.377621	TOB1/HDA	2
GO:00486	regulation of s	2/272	51/18670	0.170075	0.437759	0.377621	HDAC4/LN	2
GO:00725:	purine-contai	2/272	51/18670	0.170075	0.437759	0.377621	PDE7A/NT:	2
GO:00140(	phosphatidyli	4/272	148/1867(	0.170232	0.437759	0.377621	NKX3-1/F2	4
GO:00160	cell growth	10/272	484/1867(	0.170596	0.437759	0.377621	RPS6KA3/I	10

GO:00458(	positive regul	3/272	98/18670	0.171831	0.437759	0.377621	F2RL1/B2M	3
GO:00508	regulation of	3/272	98/18670	0.171831	0.437759	0.377621	TMBIM4/E	3
GO:00991	regulation of	3/272	98/18670	0.171831	0.437759	0.377621	CDK5R1/LF	3
GO:00508	T cell recepto	5/272	202/1867(	0.172984	0.437759	0.377621	PAK2/PSM	5
GO:00516	vesicle localiz	5/272	202/1867(	0.172984	0.437759	0.377621	GOLGA2/K	5
GO:00517(	interaction wi	5/272	202/1867(	0.172984	0.437759	0.377621	IFIT1/EIF2	5
GO:19028(	regulation of	5/272	202/1867(	0.172984	0.437759	0.377621	ARID3A/G	5
GO:00027(	regulation of	4/272	149/1867(	0.173075	0.437759	0.377621	EXOSC3/B	4
GO:19012	regulation of	7/272	313/1867(	0.173714	0.437759	0.377621	SRPK2/NQ	7
GO:00002	meiotic spindl	1/272	13/18670	0.173743	0.437759	0.377621	GOLGA2	1
GO:00065	lysine metabo	1/272	13/18670	0.173743	0.437759	0.377621	DLD	1
GO:00066	sphingosine m	1/272	13/18670	0.173743	0.437759	0.377621	ASAH1	1
GO:00082	intracellular m	1/272	13/18670	0.173743	0.437759	0.377621	EXOSC3	1
GO:00107	negative regul	1/272	13/18670	0.173743	0.437759	0.377621	NFKBIA	1
GO:00107(	positive regul	1/272	13/18670	0.173743	0.437759	0.377621	THBS1	1
GO:00108	positive regul	1/272	13/18670	0.173743	0.437759	0.377621	HIF1A	1
GO:00109	positive regul	1/272	13/18670	0.173743	0.437759	0.377621	HEBP2	1
GO:00158	gamma-aminc	1/272	13/18670	0.173743	0.437759	0.377621	ABAT	1
GO:00198	axonal transp	1/272	13/18670	0.173743	0.437759	0.377621	HIF1A	1
GO:00219	telencephalor	1/272	13/18670	0.173743	0.437759	0.377621	EOMES	1
GO:00312	positive regul	1/272	13/18670	0.173743	0.437759	0.377621	F2RL1	1
GO:00327	positive regul	1/272	13/18670	0.173743	0.437759	0.377621	IL18	1
GO:00327	positive regul	1/272	13/18670	0.173743	0.437759	0.377621	IL17RA	1
GO:00331	regulation of	1/272	13/18670	0.173743	0.437759	0.377621	RPS6KA5	1
GO:00349	mitochondrial	1/272	13/18670	0.173743	0.437759	0.377621	LRRK2	1
GO:00355(	positive regul	1/272	13/18670	0.173743	0.437759	0.377621	DTX3L	1
GO:00356	locomotory e	1/272	13/18670	0.173743	0.437759	0.377621	LRRK2	1
GO:00361(	alpha-linoleni	1/272	13/18670	0.173743	0.437759	0.377621	ACSL1	1
GO:00380(	collagen-activ	1/272	13/18670	0.173743	0.437759	0.377621	OSCAR	1
GO:00421	xenobiotic cat	1/272	13/18670	0.173743	0.437759	0.377621	ACSL1	1
GO:00447	negative regul	1/272	13/18670	0.173743	0.437759	0.377621	EIF2AK4	1
GO:00450(	DNA deamina	1/272	13/18670	0.173743	0.437759	0.377621	EXOSC3	1
GO:00450	positive regul	1/272	13/18670	0.173743	0.437759	0.377621	CLEC7A	1
GO:00450	positive regul	1/272	13/18670	0.173743	0.437759	0.377621	CLEC7A	1
GO:00458	negative regul	1/272	13/18670	0.173743	0.437759	0.377621	HDAC4	1
GO:00460	IMP metaboli	1/272	13/18670	0.173743	0.437759	0.377621	NT5C2	1
GO:00474	regulation of	1/272	13/18670	0.173743	0.437759	0.377621	PTGS2	1
GO:00507	regulation of	1/272	13/18670	0.173743	0.437759	0.377621	RAB3GAP1	1
GO:00514	myoblast migr	1/272	13/18670	0.173743	0.437759	0.377621	BIN3	1
GO:00601(	positive regul	1/272	13/18670	0.173743	0.437759	0.377621	F2RL1	1
GO:00601	regulation of	1/272	13/18670	0.173743	0.437759	0.377621	CHD7	1
GO:00603	positive regul	1/272	13/18670	0.173743	0.437759	0.377621	FADD	1
GO:00605	intestinal epit	1/272	13/18670	0.173743	0.437759	0.377621	HIF1A	1
GO:00610	negative regul	1/272	13/18670	0.173743	0.437759	0.377621	RGS2	1
GO:00614	response to pl	1/272	13/18670	0.173743	0.437759	0.377621	AHR	1
GO:00704	nucleotide-bir	1/272	13/18670	0.173743	0.437759	0.377621	NFKBIA	1
GO:00710	nuclear RNA s	1/272	13/18670	0.173743	0.437759	0.377621	EXOSC3	1
GO:00710	nuclear mRNA	1/272	13/18670	0.173743	0.437759	0.377621	EXOSC3	1

GO:00712{	cellular respo	1/272	13/18670	0.173743	0.437759	0.377621	LRRK2	1
GO:00721{	regulation of r	1/272	13/18670	0.173743	0.437759	0.377621	STAT1	1
GO:00903{	replicative ser	1/272	13/18670	0.173743	0.437759	0.377621	MME	1
GO:00905{	inflammatory	1/272	13/18670	0.173743	0.437759	0.377621	HIF1A	1
GO:00971!	mesenchymal	1/272	13/18670	0.173743	0.437759	0.377621	HIF1A	1
GO:19002:	regulation of I	1/272	13/18670	0.173743	0.437759	0.377621	EIF2AK2	1
GO:19023(	negative regul	1/272	13/18670	0.173743	0.437759	0.377621	HECW2	1
GO:19028{	negative regul	1/272	13/18670	0.173743	0.437759	0.377621	HDAC4	1
GO:19029(	negative regul	1/272	13/18670	0.173743	0.437759	0.377621	LRRK2	1
GO:19049:	positive regul	1/272	13/18670	0.173743	0.437759	0.377621	HK2	1
GO:19051!	positive regul	1/272	13/18670	0.173743	0.437759	0.377621	F2RL1	1
GO:20000(	negative regul	1/272	13/18670	0.173743	0.437759	0.377621	PICALM	1
GO:20010:	positive regul	1/272	13/18670	0.173743	0.437759	0.377621	DTX3L	1
GO:20010	positive regul	1/272	13/18670	0.173743	0.437759	0.377621	LRRK2	1
GO:00063(	postreplicatio	2/272	52/18670	0.175276	0.440367	0.379871	USP10/TRI	2
GO:00083:	endosome to	2/272	52/18670	0.175276	0.440367	0.379871	AKTIP/DTX	2
GO:00326!	regulation of i	2/272	52/18670	0.175276	0.440367	0.379871	CLEC7A/TL	2
GO:00481(	regulation of r	2/272	52/18670	0.175276	0.440367	0.379871	RAB3GAP1	2
GO:00432!	regulation of p	9/272	429/1867(	0.175883	0.440768	0.380217	CLEC7A/PF	9
GO:00355(	non-canonical	4/272	150/1867(	0.175934	0.440768	0.380217	PSMD12/T	4
GO:00613!	neural precurs	4/272	150/1867(	0.175934	0.440768	0.380217	RAPGEF1/I	4
GO:00717:	response to fil	4/272	150/1867(	0.175934	0.440768	0.380217	POLR2J/SU	4
GO:00158:	amine transpo	3/272	100/1867(	0.179043	0.445592	0.384378	RGS2/RAB:	3
GO:00616	cytoskeleton-(	3/272	100/1867(	0.179043	0.445592	0.384378	UNC119/B	3
GO:00508!	antigen recep	7/272	316/1867(	0.179498	0.445592	0.384378	PAK2/MNI	7
GO:00301(	regulation of e	5/272	205/1867(	0.180285	0.445592	0.384378	UNC119/F:	5
GO:00712:	cellular respo	5/272	205/1867(	0.180285	0.445592	0.384378	LY96/TLR2	5
GO:00076:	visual behavio	2/272	53/18670	0.180502	0.445592	0.384378	HIF1A/NPT	2
GO:00220:	telencephalon	2/272	53/18670	0.180502	0.445592	0.384378	CDK5R1/LF	2
GO:00380(	p38MAPK cas	2/272	53/18670	0.180502	0.445592	0.384378	GADD45G/	2
GO:00433:	response to d	2/272	53/18670	0.180502	0.445592	0.384378	IFIT1/NFKE	2
GO:00507(	interleukin-1	2/272	53/18670	0.180502	0.445592	0.384378	F2RL1/ORI	2
GO:19024:	L-alpha-aminc	2/272	53/18670	0.180502	0.445592	0.384378	RGS2/SLC3	2
GO:19033(	positive regul	2/272	53/18670	0.180502	0.445592	0.384378	F2RL1/IL4F	2
GO:19055:	macrophage r	2/272	53/18670	0.180502	0.445592	0.384378	THBS1/CKL	2
GO:00018{	placenta deve	4/272	152/1867(	0.1817	0.445592	0.384378	PTGS2/MN	4
GO:00072{	spermatid dev	4/272	152/1867(	0.1817	0.445592	0.384378	DLD/ROPN	4
GO:00326:	regulation of i	4/272	152/1867(	0.1817	0.445592	0.384378	F2RL1/CLE	4
GO:00356{	cellular respo	9/272	433/1867(	0.182474	0.445592	0.384378	TXN/NKX3-	9
GO:00326	regulation of i	3/272	101/1867(	0.18268	0.445592	0.384378	CLEC7A/FA	3
GO:00466:	alpha-beta T c	3/272	101/1867(	0.18268	0.445592	0.384378	IL4R/EOMI	3
GO:00482!	regulation of r	3/272	101/1867(	0.18268	0.445592	0.384378	UNC119/B	3
GO:19030(	organelle disa	3/272	101/1867(	0.18268	0.445592	0.384378	HK2/GOLG	3
GO:19034:	regulation of r	3/272	101/1867(	0.18268	0.445592	0.384378	PTGS2/CLE	3
GO:00020(	epithelial cell	5/272	207/1867(	0.185215	0.445592	0.384378	F2RL1/RAF	5
GO:00027(	positive regul	2/272	54/18670	0.185751	0.445592	0.384378	FADD/EVI2	2
GO:00066:	unsaturated f	2/272	54/18670	0.185751	0.445592	0.384378	PTGS2/ALC	2
GO:00070{	metaphase/ar	2/272	54/18670	0.185751	0.445592	0.384378	TACC3/HE(	2

GO:00610(	cell differentia	2/272	54/18670	0.185751	0.445592	0.384378	STAT1/MM	2
GO:00092:	cyclic nucleoti	1/272	14/18670	0.185789	0.445592	0.384378	PDE7A	1
GO:00107!	regulation of (	1/272	14/18670	0.185789	0.445592	0.384378	THBS1	1
GO:00107!	regulation of		1/272	14/18670	0.185789	0.445592	0.384378	THBS1	1
GO:00109:	negative regul	1/272	14/18670	0.185789	0.445592	0.384378	HEBP2	1
GO:00148!	response to in	1/272	14/18670	0.185789	0.445592	0.384378	HDAC4	1
GO:00190(	viral translatic	1/272	14/18670	0.185789	0.445592	0.384378	EIF2AK4	1
GO:00200:	hemoglobin m	1/272	14/18670	0.185789	0.445592	0.384378	HIF1A	1
GO:00302:	hyaluronan bi	1/272	14/18670	0.185789	0.445592	0.384378	ABCC5	1
GO:00302‹	skeletal muscl	1/272	14/18670	0.185789	0.445592	0.384378	LMOD3	1
GO:00308:	positive regul	1/272	14/18670	0.185789	0.445592	0.384378	F2RL1	1
GO:00312:	regulation of		1/272	14/18670	0.185789	0.445592	0.384378	F2RL1	1
GO:00326(	negative regul	1/272	14/18670	0.185789	0.445592	0.384378	PPM1B	1
GO:00431‹	snoRNA proce	1/272	14/18670	0.185789	0.445592	0.384378	EXOSC3	1
GO:00433:	CD8-positive,	1/272	14/18670	0.185789	0.445592	0.384378	EOMES	1
GO:00440!	positive regul	1/272	14/18670	0.185789	0.445592	0.384378	RAB3GAP1	1
GO:00445‹	NLRP3 inflamr	1/272	14/18670	0.185789	0.445592	0.384378	EIF2AK2	1
GO:00452:	cell-cell juncti	1/272	14/18670	0.185789	0.445592	0.384378	F2RL1	1
GO:00456!	positive regul	1/272	14/18670	0.185789	0.445592	0.384378	FADD	1
GO:00468!	inositol phosp	1/272	14/18670	0.185789	0.445592	0.384378	SYNJ2	1
GO:00482(	behavioral res	1/272	14/18670	0.185789	0.445592	0.384378	THBS1	1
GO:00483(	organelle inhe	1/272	14/18670	0.185789	0.445592	0.384378	GOLGA2	1
GO:00483:	Golgi inheritar	1/272	14/18670	0.185789	0.445592	0.384378	GOLGA2	1
GO:00519:	regulation of f	1/272	14/18670	0.185789	0.445592	0.384378	THBS1	1
GO:00600(	Sertoli cell dev	1/272	14/18670	0.185789	0.445592	0.384378	ARID4A	1
GO:00600‹	regulation of		1/272	14/18670	0.185789	0.445592	0.384378	GOLGA2	1
GO:00604!	positive regul	1/272	14/18670	0.185789	0.445592	0.384378	RGS2	1
GO:00725(	chemokine (C-	1/272	14/18670	0.185789	0.445592	0.384378	F2RL1	1
GO:00972(	activation of c	1/272	14/18670	0.185789	0.445592	0.384378	FADD	1
GO:19016:	regulation of		1/272	14/18670	0.185789	0.445592	0.384378	CDH2	1
GO:19020‹	positive regul	1/272	14/18670	0.185789	0.445592	0.384378	THBS1	1
GO:19049:	regulation of a	1/272	14/18670	0.185789	0.445592	0.384378	HK2	1
GO:19050‹	negative regul	1/272	14/18670	0.185789	0.445592	0.384378	PICALM	1
GO:20003‹	regulation of I	1/272	14/18670	0.185789	0.445592	0.384378	SULF2	1
GO:20010‹	regulation of i	1/272	14/18670	0.185789	0.445592	0.384378	BST1	1
GO:20012:	regulation of v	1/272	14/18670	0.185789	0.445592	0.384378	RAP1A	1
GO:20012:	regulation of u	1/272	14/18670	0.185789	0.445592	0.384378	PTGS2	1
GO:00069(	vesicle buddin	3/272	102/1867(	0.186336	0.446602	0.385249	GOLPH3/G	3
GO:19908‹	adaptive therr	4/272	154/1867(	0.187529	0.449156	0.387452	GADD45G/	4
GO:00075(	aging	7/272	321/1867(	0.189315	0.453102	0.390856	DLD/PTGS:	7
GO:00326!	regulation of i	3/272	103/1867(	0.190012	0.453102	0.390856	MNDA/CLE	3
GO:00442(	cellular polysa	3/272	103/1867(	0.190012	0.453102	0.390856	PYGL/IRS2,	3
GO:19052(	positive regul	3/272	103/1867(	0.190012	0.453102	0.390856	RPS6KA5/J	3
GO:00422:	response to cc	2/272	55/18670	0.191022	0.453102	0.390856	FADD/ABA	2
GO:00464!	icosanoid bios	2/272	55/18670	0.191022	0.453102	0.390856	PTGS2/ALC	2
GO:00610!	positive regul	2/272	55/18670	0.191022	0.453102	0.390856	PAK2/UNC	2
GO:00971:	receptor local	2/272	55/18670	0.191022	0.453102	0.390856	RAP1A/NP	2
GO:00435:	regulation of r	5/272	210/1867(	0.192702	0.453102	0.390856	SRPK2/NQ	5

GO:00072	neuropeptide	3/272	104/1867(	0.193707	0.453102	0.390856	LTB4R/CYS	3
GO:00109	negative regul	3/272	104/1867(	0.193707	0.453102	0.390856	LMTK2/PH	3
GO:00468	regulation of r	3/272	104/1867(	0.193707	0.453102	0.390856	TXN/PTGS	3
GO:00718	leukocyte apo	3/272	104/1867(	0.193707	0.453102	0.390856	FADD/HIF1	3
GO:00007	syncytium fori	2/272	56/18670	0.196312	0.453102	0.390856	SCGB3A1/	2
GO:00218	forebrain cell	2/272	56/18670	0.196312	0.453102	0.390856	CDK5R1/LI	2
GO:00312	T cell costimul	2/272	56/18670	0.196312	0.453102	0.390856	PAK2/TNF	2
GO:00447	metaphase/ar	2/272	56/18670	0.196312	0.453102	0.390856	TACC3/HE	2
GO:00484	synaptic vesic	2/272	56/18670	0.196312	0.453102	0.390856	LRRK2/PIC	2
GO:00508	multicellular c	2/272	56/18670	0.196312	0.453102	0.390856	TNNI2/RCS	2
GO:00508	musculoskelet	2/272	56/18670	0.196312	0.453102	0.390856	TNNI2/RCS	2
GO:00901	regulation of l	2/272	56/18670	0.196312	0.453102	0.390856	STAT1/MN	2
GO:01402	presynaptic er	2/272	56/18670	0.196312	0.453102	0.390856	LRRK2/PIC	2
GO:01402	cell-cell fusion	2/272	56/18670	0.196312	0.453102	0.390856	SCGB3A1/	2
GO:00198	stem cell popu	4/272	157/1867(	0.196383	0.453102	0.390856	POLR2J/EC	4
GO:00305	BMP signaling	4/272	157/1867(	0.196383	0.453102	0.390856	USP15/TOI	4
GO:00066	acyl-CoA meta	3/272	105/1867(	0.197419	0.453102	0.390856	DLD/ACSL1	3
GO:00320	regulation of T	3/272	105/1867(	0.197419	0.453102	0.390856	GOLPH3/R	3
GO:00331	positive regul	3/272	105/1867(	0.197419	0.453102	0.390856	TXN/PTGS	3
GO:00353	thioester met	3/272	105/1867(	0.197419	0.453102	0.390856	DLD/ACSL1	3
GO:19027	negative regul	3/272	105/1867(	0.197419	0.453102	0.390856	PSMD12/P	3
GO:00017	endodermal c	1/272	15/18670	0.19766	0.453102	0.390856	EOMES	1
GO:00018	trophectoderr	1/272	15/18670	0.19766	0.453102	0.390856	EOMES	1
GO:00024	germinal cent	1/272	15/18670	0.19766	0.453102	0.390856	TNFSF13B	1
GO:00060	fructose meta	1/272	15/18670	0.19766	0.453102	0.390856	PFKFB2	1
GO:00075	adult heart de	1/272	15/18670	0.19766	0.453102	0.390856	CHD7	1
GO:00107	positive regul	1/272	15/18670	0.19766	0.453102	0.390856	THBS1	1
GO:00148	response to st	1/272	15/18670	0.19766	0.453102	0.390856	HDAC4	1
GO:00182	protein-chrom	1/272	15/18670	0.19766	0.453102	0.390856	RGR	1
GO:00307	sequestering	1/272	15/18670	0.19766	0.453102	0.390856	OSBPL8	1
GO:00308	regulation of	1/272	15/18670	0.19766	0.453102	0.390856	EVI2B	1
GO:00323	positive regul	1/272	15/18670	0.19766	0.453102	0.390856	HIF1A	1
GO:00326	regulation of	1/272	15/18670	0.19766	0.453102	0.390856	IL18	1
GO:00327	positive regul	1/272	15/18670	0.19766	0.453102	0.390856	IL17RA	1
GO:00330	positive regul	1/272	15/18670	0.19766	0.453102	0.390856	IL4R	1
GO:00336	negative regul	1/272	15/18670	0.19766	0.453102	0.390856	ABAT	1
GO:00336	negative regul	1/272	15/18670	0.19766	0.453102	0.390856	EIF2AK2	1
GO:00341	negative regul	1/272	15/18670	0.19766	0.453102	0.390856	ABAT	1
GO:00433	positive regul	1/272	15/18670	0.19766	0.453102	0.390856	IL4R	1
GO:00435	positive regul	1/272	15/18670	0.19766	0.453102	0.390856	RPL26	1
GO:00435	nose developr	1/272	15/18670	0.19766	0.453102	0.390856	CHD7	1
GO:00436	engulfment of	1/272	15/18670	0.19766	0.453102	0.390856	THBS1	1
GO:00458	negative regul	1/272	15/18670	0.19766	0.453102	0.390856	HEBP2	1
GO:00463	positive regul	1/272	15/18670	0.19766	0.453102	0.390856	IRS2	1
GO:00468	phosphorylate	1/272	15/18670	0.19766	0.453102	0.390856	SYNJ2	1
GO:00481	regulation of	1/272	15/18670	0.19766	0.453102	0.390856	RAB3GAP1	1
GO:00600	regulation of	1/272	15/18670	0.19766	0.453102	0.390856	F2RL1	1
GO:00601	prepulse inhib	1/272	15/18670	0.19766	0.453102	0.390856	CHD8	1

GO:00705:	negative regul	1/272	15/18670	0.19766	0.453102	0.390856	MAP4K4	1
GO:00710:	RNA surveillar	1/272	15/18670	0.19766	0.453102	0.390856	EXOSC3	1
GO:00714:	cellular hyper	1/272	15/18670	0.19766	0.453102	0.390856	RCSD1	1
GO:00720:	metanephric r	1/272	15/18670	0.19766	0.453102	0.390856	STAT1	1
GO:00721(	nephron tubu	1/272	15/18670	0.19766	0.453102	0.390856	STAT1	1
GO:00722{	metanephric r	1/272	15/18670	0.19766	0.453102	0.390856	STAT1	1
GO:00901{	positive regul	1/272	15/18670	0.19766	0.453102	0.390856	IL4R	1
GO:19021{	negative regul	1/272	15/18670	0.19766	0.453102	0.390856	TRIM25	1
GO:19058:	regulation of	1/272	15/18670	0.19766	0.453102	0.390856	CDH2	1
GO:19035:	negative regul	5/272	212/1867(	0.197752	0.453102	0.390856	F2RL1/SRG	5
GO:00485:	spermatid diff	4/272	158/1867(	0.199363	0.456497	0.393785	DLD/ROPN	4
GO:00024!	T cell mediate	3/272	106/1867(	0.201148	0.458404	0.39543	B2M/FADD	3
GO:00070	lysosomal trar	3/272	106/1867(	0.201148	0.458404	0.39543	AKTIP/LRR	3
GO:00070!	mitotic spindl	3/272	106/1867(	0.201148	0.458404	0.39543	TACC3/GO	3
GO:00091:	nucleoside me	3/272	106/1867(	0.201148	0.458404	0.39543	UPB1/NT5	3
GO:00519(	regulation of s	3/272	106/1867(	0.201148	0.458404	0.39543	NTNG2/TL	3
GO:19016(	alpha-amino a	3/272	106/1867(	0.201148	0.458404	0.39543	DLD/HAL/	3
GO:00028{	regulation of r	2/272	57/18670	0.20162	0.458404	0.39543	F2RL1/IL4	2
GO:00109(	regulation of r	2/272	57/18670	0.20162	0.458404	0.39543	TACC3/HE	2
GO:00193:	hexose catabo	2/272	57/18670	0.20162	0.458404	0.39543	PFKFB2/HK	2
GO:00312{	lymphocyte cc	2/272	57/18670	0.20162	0.458404	0.39543	PAK2/TNF	2
GO:00611:	regulation of i	2/272	57/18670	0.20162	0.458404	0.39543	HIF1A/MA	2
GO:00987:	maintenance	4/272	159/1867(	0.202356	0.458904	0.395861	POLR2J/EC	4
GO:00108:	regulation of	5/272	215/1867(	0.205412	0.458904	0.395861	PLAU/THB	5
GO:00703:	positive regul	5/272	215/1867(	0.205412	0.458904	0.395861	F2RL1/RAF	5
GO:00712	cellular respo	5/272	215/1867(	0.205412	0.458904	0.395861	PTGS2/B2I	5
GO:00069	syncytium for	2/272	58/18670	0.206945	0.458904	0.395861	SCGB3A1/	2
GO:00106:	positive regul	2/272	58/18670	0.206945	0.458904	0.395861	PFKFB2/IR	2
GO:00359(	aorta develop	2/272	58/18670	0.206945	0.458904	0.395861	NKX3-1/CH	2
GO:00420{	T-helper cell d	2/272	58/18670	0.206945	0.458904	0.395861	IL4R/IL18	2
GO:00609{	dendritic spin	2/272	58/18670	0.206945	0.458904	0.395861	CDK5R1/LF	2
GO:00989:	axonal transp	2/272	58/18670	0.206945	0.458904	0.395861	KIF1B/HIF1	2
GO:00016!	urogenital sys	7/272	330/1867(	0.207521	0.458904	0.395861	NKX3-1/SU	7
GO:00025:	acute inflamm	3/272	108/1867(	0.208656	0.458904	0.395861	PTGS2/S1C	3
GO:00600:	Wnt signaling	3/272	108/1867(	0.208656	0.458904	0.395861	PSMD12/P	3
GO:00995(	chemical syna	3/272	108/1867(	0.208656	0.458904	0.395861	RAB3GAP1	3
GO:00019:	renal system	1/272	16/18670	0.209359	0.458904	0.395861	F2RL1	1
GO:00020:	epithelial cell	1/272	16/18670	0.209359	0.458904	0.395861	HIF1A	1
GO:00027:	regulation of	1/272	16/18670	0.209359	0.458904	0.395861	F2RL1	1
GO:00032:	ventricular tra	1/272	16/18670	0.209359	0.458904	0.395861	CHD7	1
GO:00060{	acetyl-CoA bic	1/272	16/18670	0.209359	0.458904	0.395861	DLD	1
GO:00061(	2-oxoglutarat	1/272	16/18670	0.209359	0.458904	0.395861	DLD	1
GO:00071!	neuron cell-ce	1/272	16/18670	0.209359	0.458904	0.395861	CDK5R1	1
GO:00108:	positive regul	1/272	16/18670	0.209359	0.458904	0.395861	NFKBIA	1
GO:00140	regulation of	1/272	16/18670	0.209359	0.458904	0.395861	RAB3GAP1	1
GO:00148(	skeletal myofi	1/272	16/18670	0.209359	0.458904	0.395861	LMOD3	1
GO:00160:	snoRNA metal	1/272	16/18670	0.209359	0.458904	0.395861	EXOSC3	1
GO:00189(	ether metabo	1/272	16/18670	0.209359	0.458904	0.395861	TXN	1

GO:00302!	growth hormc	1/272	16/18670	0.209359	0.458904	0.395861	CHD7	1
GO:00308{	negative regul	1/272	16/18670	0.209359	0.458904	0.395861	MNDA	1
GO:00312(	pseudopodiur	1/272	16/18670	0.209359	0.458904	0.395861	F2RL1	1
GO:00322:	regulation of :	1/272	16/18670	0.209359	0.458904	0.395861	PTGS2	1
GO:00326(	granulocyte m	1/272	16/18670	0.209359	0.458904	0.395861	IL18	1
GO:00326!	negative regul	1/272	16/18670	0.209359	0.458904	0.395861	THBS1	1
GO:00343:	regulation of /	1/272	16/18670	0.209359	0.458904	0.395861	GMFG	1
GO:00359:	peptidyl-threc	1/272	16/18670	0.209359	0.458904	0.395861	PPM1B	1
GO:00361:	histone H3-K9	1/272	16/18670	0.209359	0.458904	0.395861	ARID4A	1
GO:00420:	chemokine bi	1/272	16/18670	0.209359	0.458904	0.395861	IL18	1
GO:00424	hormone cata	1/272	16/18670	0.209359	0.458904	0.395861	HSD17B11	1
GO:00432!	sodium-indep	1/272	16/18670	0.209359	0.458904	0.395861	SLCO4C1	1
GO:00448:	modulation by	1/272	16/18670	0.209359	0.458904	0.395861	EIF2AK4	1
GO:00456:	negative regul	1/272	16/18670	0.209359	0.458904	0.395861	IL4R	1
GO:00459{	positive regul:	1/272	16/18670	0.209359	0.458904	0.395861	RGS2	1
GO:00465:	sphingoid met	1/272	16/18670	0.209359	0.458904	0.395861	ASAH1	1
GO:00507!	chemokine m	1/272	16/18670	0.209359	0.458904	0.395861	IL18	1
GO:00551:	relaxation of c	1/272	16/18670	0.209359	0.458904	0.395861	RGS2	1
GO:00606:	regulation of I	1/272	16/18670	0.209359	0.458904	0.395861	LRRK2	1
GO:00705!	dendrite self-a	1/272	16/18670	0.209359	0.458904	0.395861	NPTN	1
GO:00715	inositol phosp	1/272	16/18670	0.209359	0.458904	0.395861	SYNJ2	1
GO:00717{	endoplasmic r	1/272	16/18670	0.209359	0.458904	0.395861	RAB3GAP1	1
GO:00801{	histone H3-K4	1/272	16/18670	0.209359	0.458904	0.395861	ARID4A	1
GO:00861(	G protein-cou	1/272	16/18670	0.209359	0.458904	0.395861	RGS2	1
GO:00903:	regulation of I	1/272	16/18670	0.209359	0.458904	0.395861	PTGS2	1
GO:00970!	synaptic vesicl	1/272	16/18670	0.209359	0.458904	0.395861	CDH2	1
GO:19002	regulation of :	1/272	16/18670	0.209359	0.458904	0.395861	LRRK2	1
GO:19023!	sulfate transm	1/272	16/18670	0.209359	0.458904	0.395861	SLC26A8	1
GO:19029!	negative regul	1/272	16/18670	0.209359	0.458904	0.395861	PICALM	1
GO:19051!	regulation of r	1/272	16/18670	0.209359	0.458904	0.395861	F2RL1	1
GO:19900(	amyloid fibril	1/272	16/18670	0.209359	0.458904	0.395861	B2M	1
GO:20003{	regulation of c	1/272	16/18670	0.209359	0.458904	0.395861	UNC119	1
GO:00147(	striated muscl	8/272	390/1867(	0.210406	0.460914	0.397595	IFRD1/SAP	8
GO:00076(	phototransdu	2/272	59/18670	0.212284	0.463634	0.399941	RGR/UNC1	2
GO:00977!	positive regul:	2/272	59/18670	0.212284	0.463634	0.399941	F2RL1/RGS	2
GO:01201:	steroid hormc	2/272	59/18670	0.212284	0.463634	0.399941	HSD17B11	2
GO:00016:	long-chain fat	3/272	109/1867(	0.212433	0.463634	0.399941	PTGS2/ACS	3
GO:00217(	limbic system	3/272	109/1867(	0.212433	0.463634	0.399941	CDK5R1/R/	3
GO:00512(	protein depol	3/272	109/1867(	0.212433	0.463634	0.399941	F2RL1/LM(	3
GO:00511:	import into nu	4/272	163/1867(	0.214461	0.467483	0.403262	PTGS2/RPI	4
GO:00991:	postsynapse c	4/272	163/1867(	0.214461	0.467483	0.403262	NTNG2/CD	4
GO:00075{	response to n	5/272	219/1867(	0.215776	0.468509	0.404146	PTGS2/ACS	5
GO:00325(	DNA duplex u	3/272	110/1867(	0.216226	0.468509	0.404146	CHD7/MCI	3
GO:00469:	cellular transit	3/272	110/1867(	0.216226	0.468509	0.404146	S100A9/S1	3
GO:00613{	regulation of e	3/272	110/1867(	0.216226	0.468509	0.404146	IFRD1/TNF	3
GO:00901:	regulation of e	3/272	110/1867(	0.216226	0.468509	0.404146	PSMD12/P	3
GO:00022!	CD4-positive,	2/272	60/18670	0.217637	0.468509	0.404146	IL4R/IL18	2
GO:00181	peptide cross-	2/272	60/18670	0.217637	0.468509	0.404146	THBS1/TGI	2

GO:00455	mast cell activ	2/272	60/18670	0.217637	0.468509	0.404146	IL4R/S100	2
GO:00461	purine ribonu	2/272	60/18670	0.217637	0.468509	0.404146	NT5C2/LRI	2
GO:00513	mitotic sister	2/272	60/18670	0.217637	0.468509	0.404146	TACC3/HE	2
GO:00518	regulation of	2/272	60/18670	0.217637	0.468509	0.404146	THBS1/MA	2
GO:00901	regulation of	2/272	60/18670	0.217637	0.468509	0.404146	THBS1/MA	2
GO:01501	regulation of	2/272	60/18670	0.217637	0.468509	0.404146	THBS1/MA	2
GO:00063	chromatin ren	5/272	220/1867	0.218393	0.468509	0.404146	HDAC4/CH	5
GO:00353	negative regul	3/272	111/1867	0.220032	0.468509	0.404146	LMTK2/PH	3
GO:00026	positive regul	1/272	17/18670	0.220887	0.468509	0.404146	FADD	1
GO:00069	DNA damage	1/272	17/18670	0.220887	0.468509	0.404146	RPL26	1
GO:00073	fusion of sper	1/272	17/18670	0.220887	0.468509	0.404146	FOLR3	1
GO:00076	chemosensory	1/272	17/18670	0.220887	0.468509	0.404146	CHD7	1
GO:00082	sulfate transp	1/272	17/18670	0.220887	0.468509	0.404146	SLC26A8	1
GO:00083	determinatior	1/272	17/18670	0.220887	0.468509	0.404146	LRRK2	1
GO:00102	response to U	1/272	17/18670	0.220887	0.468509	0.404146	MME	1
GO:00107	positive regul	1/272	17/18670	0.220887	0.468509	0.404146	IL18	1
GO:00170	response to in	1/272	17/18670	0.220887	0.468509	0.404146	KIF1B	1
GO:00300	maintenance	1/272	17/18670	0.220887	0.468509	0.404146	ARPC5	1
GO:00305	female genital	1/272	17/18670	0.220887	0.468509	0.404146	CHD7	1
GO:00312	pseudopodiur	1/272	17/18670	0.220887	0.468509	0.404146	F2RL1	1
GO:00327	positive regul	1/272	17/18670	0.220887	0.468509	0.404146	IL18	1
GO:00329	negative regul	1/272	17/18670	0.220887	0.468509	0.404146	RAP1A	1
GO:00429	lipoprotein tra	1/272	17/18670	0.220887	0.468509	0.404146	UNC119	1
GO:00429	cytoplasmic se	1/272	17/18670	0.220887	0.468509	0.404146	NFKBIA	1
GO:00436	linoleic acid m	1/272	17/18670	0.220887	0.468509	0.404146	ACSL1	1
GO:00439	histone H2A a	1/272	17/18670	0.220887	0.468509	0.404146	EPC1	1
GO:00448	lipoprotein loc	1/272	17/18670	0.220887	0.468509	0.404146	UNC119	1
GO:00450	regulation of i	1/272	17/18670	0.220887	0.468509	0.404146	CLEC7A	1
GO:00457	positive regul	1/272	17/18670	0.220887	0.468509	0.404146	IRS2	1
GO:00483	mitochondrioi	1/272	17/18670	0.220887	0.468509	0.404146	LRRK2	1
GO:00485	lymph node d	1/272	17/18670	0.220887	0.468509	0.404146	FADD	1
GO:00600	inhibitory pos	1/272	17/18670	0.220887	0.468509	0.404146	ABAT	1
GO:00606	mammary gla	1/272	17/18670	0.220887	0.468509	0.404146	HIF1A	1
GO:00612	retina vascula	1/272	17/18670	0.220887	0.468509	0.404146	HIF1A	1
GO:00702	thymocyte ap	1/272	17/18670	0.220887	0.468509	0.404146	HIF1A	1
GO:00713	cellular respor	1/272	17/18670	0.220887	0.468509	0.404146	IFIT1	1
GO:00990	modification c	1/272	17/18670	0.220887	0.468509	0.404146	PFN2	1
GO:19053	negative regul	1/272	17/18670	0.220887	0.468509	0.404146	STAT1	1
GO:00000	G1/S transitio	6/272	279/1867	0.222819	0.469897	0.405344	ARID3A/G	6
GO:00070	lysosome orga	2/272	61/18670	0.223002	0.469897	0.405344	AKTIP/LRR	2
GO:00074	neuroblast pr	2/272	61/18670	0.223002	0.469897	0.405344	HIF1A/LRR	2
GO:00108	regulation of t	2/272	61/18670	0.223002	0.469897	0.405344	F2RL1/RFF	2
GO:00323	positive regul	2/272	61/18670	0.223002	0.469897	0.405344	NKX3-1/NI	2
GO:00447	multi-organisr	2/272	61/18670	0.223002	0.469897	0.405344	IFIT1/RAE1	2
GO:00507	interleukin-1 s	2/272	61/18670	0.223002	0.469897	0.405344	F2RL1/ORI	2
GO:00801	lytic vacuole c	2/272	61/18670	0.223002	0.469897	0.405344	AKTIP/LRR	2
GO:00903	positive regul	2/272	61/18670	0.223002	0.469897	0.405344	THBS1/CLE	2
GO:19025	multi-organisr	2/272	61/18670	0.223002	0.469897	0.405344	IFIT1/RAE1	2

GO:20005:	regulation of (	2/272	61/18670	0.223002	0.469897	0.405344	IL4R/IL18	2
GO:00447(	multi-multicel	5/272	222/1867(	0.223655	0.47029	0.405683	PTGS2/PS(	5
GO:00902{	negative regul	4/272	166/1867(	0.223669	0.47029	0.405683	SULF2/TOE	4
GO:00109(	regulation of {	3/272	112/1867(	0.223853	0.47029	0.405683	PFKFB2/IR:	3
GO:00159:	sterol transpo	3/272	112/1867(	0.223853	0.47029	0.405683	NFKBIA/OS	3
GO:19038;	negative regul	3/272	112/1867(	0.223853	0.47029	0.405683	TXN/LRRK;	3
GO:00358;	modulation of	3/272	113/1867(	0.227686	0.47414	0.409004	EIF2AK2/S:	3
GO:00432	regulation of	3/272	113/1867(	0.227686	0.47414	0.409004	F2RL1/LM(	3
GO:00486	negative regul	3/272	113/1867(	0.227686	0.47414	0.409004	IFRD1/RGS	3
GO:00323{	negative regul	2/272	62/18670	0.228377	0.47414	0.409004	TXN/LRRK;	2
GO:00326;	interleukin-2	2/272	62/18670	0.228377	0.47414	0.409004	CLEC7A/IL:	2
GO:00466:	positive regul	2/272	62/18670	0.228377	0.47414	0.409004	IL4R/IL18	2
GO:19058:	regulation of (	2/272	62/18670	0.228377	0.47414	0.409004	TACC3/HE(	2
GO:19901:	neuron projec	4/272	168/1867(	0.229864	0.47414	0.409004	IFRD1/TNF	4
GO:00059:	polysaccharid	3/272	114/1867(	0.231532	0.47414	0.409004	PYGL/IRS2,	3
GO:00106:	negative regul	3/272	114/1867(	0.231532	0.47414	0.409004	THBS1/PFN	3
GO:00302{	bone minerali	3/272	114/1867(	0.231532	0.47414	0.409004	PTGS2/SR(	3
GO:00420:	regulation of (	3/272	114/1867(	0.231532	0.47414	0.409004	THBS1/CLE	3
GO:00454	endothelial ce	3/272	114/1867(	0.231532	0.47414	0.409004	F2RL1/RAF	3
GO:00029(	regulation of I	1/272	18/18670	0.232248	0.47414	0.409004	IRS2	1
GO:00063	regulation of {	1/272	18/18670	0.232248	0.47414	0.409004	ARID4A	1
GO:00072;	positive regul	1/272	18/18670	0.232248	0.47414	0.409004	STAT1	1
GO:00072!	I-kappaB phos	1/272	18/18670	0.232248	0.47414	0.409004	TLR2	1
GO:00080!	retrograde ax	1/272	18/18670	0.232248	0.47414	0.409004	KIF1B	1
GO:00107:	negative regul	1/272	18/18670	0.232248	0.47414	0.409004	RAP1A	1
GO:00107(	negative regul	1/272	18/18670	0.232248	0.47414	0.409004	HECW2	1
GO:00108:	negative regul	1/272	18/18670	0.232248	0.47414	0.409004	HDAC4	1
GO:00190!	modulation by	1/272	18/18670	0.232248	0.47414	0.409004	EIF2AK4	1
GO:00217!	striatum deve	1/272	18/18670	0.232248	0.47414	0.409004	LRRK2	1
GO:00319!	regulation of f	1/272	18/18670	0.232248	0.47414	0.409004	IRS2	1
GO:00341!	activation of p	1/272	18/18670	0.232248	0.47414	0.409004	PRKAR2A	1
GO:00345{	piRNA metabo	1/272	18/18670	0.232248	0.47414	0.409004	TDRD1	1
GO:00359!	response to m	1/272	18/18670	0.232248	0.47414	0.409004	NFKBIA	1
GO:00427:	DNA damage i	1/272	18/18670	0.232248	0.47414	0.409004	RPL26	1
GO:00450!	transcytosis	1/272	18/18670	0.232248	0.47414	0.409004	PICALM	1
GO:00482(	clathrin coat a	1/272	18/18670	0.232248	0.47414	0.409004	PICALM	1
GO:00518!	negative regul	1/272	18/18670	0.232248	0.47414	0.409004	THBS1	1
GO:00601!	negative regul	1/272	18/18670	0.232248	0.47414	0.409004	RGS2	1
GO:00605	negative regul	1/272	18/18670	0.232248	0.47414	0.409004	FADD	1
GO:00607	mammary gla	1/272	18/18670	0.232248	0.47414	0.409004	HIF1A	1
GO:00613:	mammary gla	1/272	18/18670	0.232248	0.47414	0.409004	HIF1A	1
GO:00620!	negative regul	1/272	18/18670	0.232248	0.47414	0.409004	FADD	1
GO:00708:	positive regul	1/272	18/18670	0.232248	0.47414	0.409004	IRS2	1
GO:00713:	cellular respo	1/272	18/18670	0.232248	0.47414	0.409004	PTGS2	1
GO:00716(	monocyte che	1/272	18/18670	0.232248	0.47414	0.409004	CLEC7A	1
GO:00716:	regulation of r	1/272	18/18670	0.232248	0.47414	0.409004	CLEC7A	1
GO:00720:	renal vesicle n	1/272	18/18670	0.232248	0.47414	0.409004	STAT1	1
GO:00725:	hepatocyte pr	1/272	18/18670	0.232248	0.47414	0.409004	SULF2	1

GO:00725	epithelial cell	1/272	18/18670	0.232248	0.47414	0.409004	SULF2	1
GO:00903	regulation of p	1/272	18/18670	0.232248	0.47414	0.409004	ABAT	1
GO:01501	negative regul	1/272	18/18670	0.232248	0.47414	0.409004	THBS1	1
GO:19002	regulation of a	1/272	18/18670	0.232248	0.47414	0.409004	IFNGR1	1
GO:19005	negative regul	1/272	18/18670	0.232248	0.47414	0.409004	HDAC4	1
GO:19021	regulation of i	1/272	18/18670	0.232248	0.47414	0.409004	RPL26	1
GO:20004	regulation of c	1/272	18/18670	0.232248	0.47414	0.409004	PRKAR2A	1
GO:00303	positive regula	4/272	169/1867C	0.232978	0.474219	0.409072	RPS6KA3/T	4
GO:00380	Fc-epsilon rec	4/272	169/1867C	0.232978	0.474219	0.409072	PAK2/PSM	4
GO:01400	organelle loca	4/272	169/1867C	0.232978	0.474219	0.409072	CEP152/ST	4
GO:00016	ameboidal-typ	9/272	462/1867C	0.233346	0.474219	0.409072	PTGS2/THI	9
GO:00026	regulation of i	2/272	63/18670	0.23376	0.474219	0.409072	IL4R/EXOS	2
GO:00068	Golgi to plasm	2/272	63/18670	0.23376	0.474219	0.409072	GOLPH3/E	2
GO:00422	ribosome asse	2/272	63/18670	0.23376	0.474219	0.409072	RPS28/RPS	2
GO:00422	purine nucleo	2/272	63/18670	0.23376	0.474219	0.409072	NT5C2/LRI	2
GO:00429	amyloid precu	2/272	63/18670	0.23376	0.474219	0.409072	IFNGR1/PI	2
GO:00466	regulation of a	2/272	63/18670	0.23376	0.474219	0.409072	IL4R/IL18	2
GO:00466	response to ca	2/272	63/18670	0.23376	0.474219	0.409072	B2M/MMI	2
GO:00329	collagen meta	3/272	115/1867C	0.235389	0.47725	0.411687	HIF1A/RAI	3
GO:00457	positive regula	8/272	403/1867C	0.235671	0.477328	0.411754	PAK2/SIRP	8
GO:00070	spindle organi	4/272	170/1867C	0.236102	0.477328	0.411754	TACC3/GO	4
GO:00434	negative regul	4/272	170/1867C	0.236102	0.477328	0.411754	EIF2AK4/E	4
GO:00717	response to B	4/272	170/1867C	0.236102	0.477328	0.411754	USP15/TOI	4
GO:00717	cellular respo	4/272	170/1867C	0.236102	0.477328	0.411754	USP15/TOI	4
GO:00516	protein matur	6/272	285/1867C	0.236974	0.478818	0.413039	PLAU/MM	6
GO:00601	maternal proc	2/272	64/18670	0.239152	0.481989	0.415774	PTGS2/RG	2
GO:20003	negative regul	2/272	64/18670	0.239152	0.481989	0.415774	HK2/HIF1A	2
GO:00009	cytokinesis	4/272	171/1867C	0.239236	0.481989	0.415774	UNC119/B	4
GO:00066	phosphatidyli	3/272	116/1867C	0.239258	0.481989	0.415774	SYNJ2/MPI	3
GO:00217	glial cell devel	3/272	116/1867C	0.239258	0.481989	0.415774	IFNGR1/S1	3
GO:00159	energy derivat	6/272	286/1867C	0.23936	0.481989	0.415774	DLD/PYGL/	6
GO:00226	ribonucleopro	5/272	229/1867C	0.242365	0.482257	0.416006	RPS28/RPS	5
GO:00512	protein tetran	4/272	172/1867C	0.24238	0.482257	0.416006	UPB1/B2M	4
GO:00076	memory	3/272	117/1867C	0.243138	0.482257	0.416006	PTGS2/NQ	3
GO:00020	osteoblast dev	1/272	19/18670	0.243445	0.482257	0.416006	HDAC4	1
GO:00072	G protein-cou	1/272	19/18670	0.243445	0.482257	0.416006	CDK5R1	1
GO:00105	negative regul	1/272	19/18670	0.243445	0.482257	0.416006	ABAT	1
GO:00160	rRNA catabolic	1/272	19/18670	0.243445	0.482257	0.416006	EXOSC3	1
GO:00309	positive regula	1/272	19/18670	0.243445	0.482257	0.416006	HIF1A	1
GO:00320	ARF protein si	1/272	19/18670	0.243445	0.482257	0.416006	MAP4K4	1
GO:00320	regulation of A	1/272	19/18670	0.243445	0.482257	0.416006	MAP4K4	1
GO:00320	response to m	1/272	19/18670	0.243445	0.482257	0.416006	THBS1	1
GO:00322	myelin asseml	1/272	19/18670	0.243445	0.482257	0.416006	TLR2	1
GO:00327	negative regul	1/272	19/18670	0.243445	0.482257	0.416006	IFIT1	1
GO:00343	diol biosynthe	1/272	19/18670	0.243445	0.482257	0.416006	ASAH1	1
GO:00427	mRNA transcr	1/272	19/18670	0.243445	0.482257	0.416006	HIF1A	1
GO:00450	T-helper 1 cell	1/272	19/18670	0.243445	0.482257	0.416006	IL4R	1
GO:00450	regulation of i	1/272	19/18670	0.243445	0.482257	0.416006	CLEC7A	1

GO:00454:	regulation of i	1/272	19/18670	0.243445	0.482257	0.416006	CLEC7A	1
GO:00459	negative regul	1/272	19/18670	0.243445	0.482257	0.416006	EIF2AK4	1
GO:00459	negative regul	1/272	19/18670	0.243445	0.482257	0.416006	HDAC4	1
GO:00465	negative regul	1/272	19/18670	0.243445	0.482257	0.416006	TRIM25	1
GO:00466	positive regul	1/272	19/18670	0.243445	0.482257	0.416006	IL18	1
GO:00602	mesenchymal	1/272	19/18670	0.243445	0.482257	0.416006	STAT1	1
GO:00720	kidney mesen	1/272	19/18670	0.243445	0.482257	0.416006	STAT1	1
GO:00720	renal vesicle d	1/272	19/18670	0.243445	0.482257	0.416006	STAT1	1
GO:00725	liver morphog	1/272	19/18670	0.243445	0.482257	0.416006	SULF2	1
GO:19021	negative regul	1/272	19/18670	0.243445	0.482257	0.416006	HIF1A	1
GO:19046	regulation of r	1/272	19/18670	0.243445	0.482257	0.416006	PICALM	1
GO:20008	regulation of s	1/272	19/18670	0.243445	0.482257	0.416006	NKX3-1	1
GO:00226	ribonucleopro	9/272	468/1867C	0.244478	0.482554	0.416262	RPS28/RPS	9
GO:00003	regulation of a	2/272	65/18670	0.244549	0.482554	0.416262	RBM7/RBM	2
GO:00070	endoplasmic r	2/272	65/18670	0.244549	0.482554	0.416262	RAB3GAP1	2
GO:00091	nucleotide cat	2/272	65/18670	0.244549	0.482554	0.416262	PDE7A/NT	2
GO:00343	adherens junc	2/272	65/18670	0.244549	0.482554	0.416262	CDH2/MAI	2
GO:00511	negative regul	2/272	65/18670	0.244549	0.482554	0.416262	RGS2/HDA	2
GO:00603	pathway-restr	2/272	65/18670	0.244549	0.482554	0.416262	USP15/PBI	2
GO:19040	positive regul	5/272	230/1867C	0.245072	0.483317	0.41692	PTGS2/THI	5
GO:00550	striated muscl	4/272	173/1867C	0.245533	0.483671	0.417225	BIN3/RGS2	4
GO:00508	synapse orgar	8/272	408/1867C	0.245661	0.483671	0.417225	NTNG2/PF	8
GO:00605	muscle tissue	8/272	408/1867C	0.245661	0.483671	0.417225	IFRD1/SAP	8
GO:00511	cofactor biosy	5/272	231/1867C	0.247787	0.487314	0.420368	DLD/PTGS	5
GO:19033	regulation of p	5/272	231/1867C	0.247787	0.487314	0.420368	GOLGA2/R	5
GO:00158	acidic amino a	2/272	66/18670	0.249951	0.487738	0.420734	RAB3GAP1	2
GO:00454	regulation of r	2/272	66/18670	0.249951	0.487738	0.420734	PTGS2/CLE	2
GO:00706	regulation of p	2/272	66/18670	0.249951	0.487738	0.420734	THBS1/LRI	2
GO:19018	negative regul	2/272	66/18670	0.249951	0.487738	0.420734	LMOD3/AI	2
GO:00621	cellular respor	7/272	350/1867C	0.250146	0.487738	0.420734	TXN/PTGS	7
GO:00323	DNA geometri	3/272	119/1867C	0.250928	0.487738	0.420734	CHD7/MCI	3
GO:01200	regulation of p	4/272	175/1867C	0.251867	0.487738	0.420734	F2RL1/PFN	4
GO:00030	muscle systen	9/272	472/1867C	0.252001	0.487738	0.420734	SULF2/PTG	9
GO:00902	regulation of c	6/272	292/1867C	0.253826	0.487738	0.420734	SULF2/TOI	6
GO:00025	regulation of a	1/272	20/18670	0.254478	0.487738	0.420734	THBS1	1
GO:00028	positive regul	1/272	20/18670	0.254478	0.487738	0.420734	IL18	1
GO:00032	cardiac right v	1/272	20/18670	0.254478	0.487738	0.420734	CHD7	1
GO:00065	arginine meta	1/272	20/18670	0.254478	0.487738	0.420734	PADI4	1
GO:00076	copulation	1/272	20/18670	0.254478	0.487738	0.420734	ABAT	1
GO:00083	asymmetric ce	1/272	20/18670	0.254478	0.487738	0.420734	GOLGA2	1
GO:00100	cardioblast dif	1/272	20/18670	0.254478	0.487738	0.420734	EOMES	1
GO:00105	positive regul	1/272	20/18670	0.254478	0.487738	0.420734	GOLGA2	1
GO:00105	positive regul	1/272	20/18670	0.254478	0.487738	0.420734	HDAC4	1
GO:00107	regulation of p	1/272	20/18670	0.254478	0.487738	0.420734	LRRK2	1
GO:00193	leukotriene bi	1/272	20/18670	0.254478	0.487738	0.420734	ALOX5AP	1
GO:00324	response to m	1/272	20/18670	0.254478	0.487738	0.420734	NFKBIA	1
GO:00328	positive regul	1/272	20/18670	0.254478	0.487738	0.420734	IL18	1
GO:00330	positive regul	1/272	20/18670	0.254478	0.487738	0.420734	IL4R	1

GO:00341	regulation of t	1/272	20/18670	0.254478	0.487738	0.420734	F2RL1	1
GO:00420	regulation of c	1/272	20/18670	0.254478	0.487738	0.420734	ABAT	1
GO:00420	regulation of c	1/272	20/18670	0.254478	0.487738	0.420734	ABAT	1
GO:00422	interleukin-8 b	1/272	20/18670	0.254478	0.487738	0.420734	CLEC7A	1
GO:00430	DNA methylat	1/272	20/18670	0.254478	0.487738	0.420734	TDRD1	1
GO:00433	negative regul	1/272	20/18670	0.254478	0.487738	0.420734	IL4R	1
GO:00465	ceramide cata	1/272	20/18670	0.254478	0.487738	0.420734	ASAH1	1
GO:00468	negative regul	1/272	20/18670	0.254478	0.487738	0.420734	TXN	1
GO:00486	regulation of c	1/272	20/18670	0.254478	0.487738	0.420734	IFRD1	1
GO:00510	barbed-end ac	1/272	20/18670	0.254478	0.487738	0.420734	ADD3	1
GO:00515	regulation of r	1/272	20/18670	0.254478	0.487738	0.420734	RGS2	1
GO:00600	Sertoli cell diff	1/272	20/18670	0.254478	0.487738	0.420734	ARID4A	1
GO:00709	histone H3 de	1/272	20/18670	0.254478	0.487738	0.420734	HDAC4	1
GO:00722	metanephric r	1/272	20/18670	0.254478	0.487738	0.420734	STAT1	1
GO:00977	connective tis	1/272	20/18670	0.254478	0.487738	0.420734	HIF1A	1
GO:00986	postsynaptic s	1/272	20/18670	0.254478	0.487738	0.420734	NTNG2	1
GO:00987	response to m	1/272	20/18670	0.254478	0.487738	0.420734	HK2	1
GO:19004	regulation of c	1/272	20/18670	0.254478	0.487738	0.420734	F2RL1	1
GO:19018	positive regul	1/272	20/18670	0.254478	0.487738	0.420734	F2RL1	1
GO:19022	negative regul	1/272	20/18670	0.254478	0.487738	0.420734	LRRK2	1
GO:19032	negative regul	1/272	20/18670	0.254478	0.487738	0.420734	HIF1A	1
GO:19039	regulation of r	1/272	20/18670	0.254478	0.487738	0.420734	LRRK2	1
GO:19055	positive regul	1/272	20/18670	0.254478	0.487738	0.420734	THBS1	1
GO:20007	positive regul	1/272	20/18670	0.254478	0.487738	0.420734	B2M	1
GO:00030	renal system p	3/272	120/1867	0.254837	0.487837	0.420819	SULF2/F2R	3
GO:00720	stem cell proli	3/272	120/1867	0.254837	0.487837	0.420819	EIF2AK2/H	3
GO:00094	response to h	4/272	176/1867	0.255047	0.487837	0.420819	PTGS2/THI	4
GO:00158	L-amino acid t	2/272	67/18670	0.255356	0.487837	0.420819	RGS2/SLC3	2
GO:00463	monosacchari	2/272	67/18670	0.255356	0.487837	0.420819	PFKFB2/HK	2
GO:00512	centrosome d	2/272	67/18670	0.255356	0.487837	0.420819	CEP152/CE	2
GO:00485	development	5/272	234/1867	0.255979	0.488762	0.421617	IFRD1/TNF	5
GO:00604	regulation of c	4/272	177/1867	0.258235	0.492804	0.425104	F2RL1/PFN	4
GO:00605	development	5/272	235/1867	0.258724	0.493264	0.425501	IFRD1/TNF	5
GO:00486	axon extensio	3/272	121/1867	0.258755	0.493264	0.425501	IFRD1/TNF	3
GO:00315	cell-substrate	7/272	354/1867	0.258982	0.493431	0.425645	PLAU/MEG	7
GO:00108	negative regul	2/272	68/18670	0.260764	0.495228	0.427195	THBS1/MA	2
GO:00162	positive regul	2/272	68/18670	0.260764	0.495228	0.427195	RAB3GAP1	2
GO:00199	lipid storage	2/272	68/18670	0.260764	0.495228	0.427195	NFKBIA/OS	2
GO:00330	regulation of r	2/272	68/18670	0.260764	0.495228	0.427195	TACC3/HE	2
GO:00359	skeletal muscl	2/272	68/18670	0.260764	0.495228	0.427195	SAP30/EOI	2
GO:19033	regulation of p	2/272	68/18670	0.260764	0.495228	0.427195	THBS1/LRF	2
GO:00463	carboxylic aci	7/272	355/1867	0.261205	0.495517	0.427444	ABCC5/PT	7
GO:00224	membrane do	4/272	178/1867	0.261431	0.495517	0.427444	CEP152/ST	4
GO:00508	protein stabili	4/272	178/1867	0.261431	0.495517	0.427444	PFN2/MDN	4
GO:00718	ribonucleopro	5/272	236/1867	0.261476	0.495517	0.427444	RPS28/RPS	5
GO:00015	regulation of c	8/272	416/1867	0.261929	0.495957	0.427824	RPS6KA3/I	8
GO:19034	reactive oxyg	3/272	122/1867	0.26268	0.495957	0.427824	PTGS2/CLE	3
GO:00069	humoral imm	7/272	356/1867	0.263434	0.495957	0.427824	PPP2R3C/C	7

GO:00160!	organic acid b	7/272	356/1867(	0.263434	0.495957	0.427824	ABCC5/PT(	7
GO:0072З:	monocarboxyl	5/272	237/1867(	0.264236	0.495957	0.427824	PTGS2/HSI	5
GO:00019!	positive regul	1/272	21/18670	0.265351	0.495957	0.427824	RAB3GAP1	1
GO:00032{	atrial septum	1/272	21/18670	0.265351	0.495957	0.427824	MDM4	1
GO:00061.	mitochondrial	1/272	21/18670	0.265351	0.495957	0.427824	COX7B	1
GO:00064!	regulation of t	1/272	21/18670	0.265351	0.495957	0.427824	YRDC	1
GO:00196‹	aerobic electr	1/272	21/18670	0.265351	0.495957	0.427824	COX7B	1
GO:00302	maintenance	1/272	21/18670	0.265351	0.495957	0.427824	PBLD	1
GO:00325:	positive regul	1/272	21/18670	0.265351	0.495957	0.427824	PPP1R12A	1
GO:00326	regulation of i	1/272	21/18670	0.265351	0.495957	0.427824	IL17RA	1
GO:00326{	negative regul	1/272	21/18670	0.265351	0.495957	0.427824	F2RL1	1
GO:00329{	protein-DNA c	1/272	21/18670	0.265351	0.495957	0.427824	RPL23	1
GO:00343{	lipid droplet o	1/272	21/18670	0.265351	0.495957	0.427824	RAB3GAP1	1
GO:00351(	embryonic he	1/272	21/18670	0.265351	0.495957	0.427824	HIF1A	1
GO:00354!	response to in	1/272	21/18670	0.265351	0.495957	0.427824	EIF2AK2	1
GO:00357‹	CD4-positive,	1/272	21/18670	0.265351	0.495957	0.427824	IL18	1
GO:00421:	neurotransmit	1/272	21/18670	0.265351	0.495957	0.427824	ABAT	1
GO:00450.	plasma memb	1/272	21/18670	0.265351	0.495957	0.427824	FOLR3	1
GO:00467:	muscle cell ce	1/272	21/18670	0.265351	0.495957	0.427824	HIF1A	1
GO:00487!	calcium ion-re	1/272	21/18670	0.265351	0.495957	0.427824	RAB3GAP1	1
GO:00714!	cellular respo	1/272	21/18670	0.265351	0.495957	0.427824	PTGS2	1
GO:00721:	cell proliferati	1/272	21/18670	0.265351	0.495957	0.427824	STAT1	1
GO:20002(	regulation of f	1/272	21/18670	0.265351	0.495957	0.427824	CHD8	1
GO:00422!	ribosome biog	6/272	297/1867(	0.266062	0.496445	0.428245	RPS28/RPS	6
GO:00080{	axo-dendritic	2/272	69/18670	0.266173	0.496445	0.428245	KIF1B/HIF1	2
GO:00364(	synaptic vesic	2/272	69/18670	0.266173	0.496445	0.428245	LRRK2/PIC.	2
GO:00507	negative regul	2/272	69/18670	0.266173	0.496445	0.428245	IFRD1/CDK	2
GO:00510‹	negative regul	5/272	238/1867(	0.267002	0.497728	0.429352	F2RL1/SRG	5
GO:00714(	cellular respo	4/272	180/1867(	0.267846	0.498629	0.430128	ACSL1/NQ(	4
GO:19053:	regulation of r	4/272	180/1867(	0.267846	0.498629	0.430128	STAT1/PSN	4
GO:00458(	positive regul	7/272	358/1867(	0.267907	0.498629	0.430128	NKX3-1/CL	7
GO:00448‹	cell cycle G1/S	6/272	298/1867(	0.268527	0.49952	0.430897	ARID3A/G/	6
GO:00017:	establishment	3/272	124/1867(	0.270554	0.501166	0.432317	PSMD12/P	3
GO:00071(	establishment	3/272	124/1867(	0.270554	0.501166	0.432317	PSMD12/P	3
GO:00480:	phosphatidyli	4/272	181/1867(	0.271064	0.501166	0.432317	NKX3-1/F2	4
GO:00330	T cell differen	2/272	70/18670	0.271582	0.501166	0.432317	B2M/FADI	2
GO:00603!	SMAD protein	2/272	70/18670	0.271582	0.501166	0.432317	TOB1/PBLI	2
GO:00082:	regulation of l	4/272	182/1867(	0.27429	0.501166	0.432317	F2RL1/PTG	4
GO:00319.	TOR signaling	3/272	125/1867(	0.274501	0.501166	0.432317	GOLPH3/R	3
GO:00454	response to e	3/272	125/1867(	0.274501	0.501166	0.432317	RGS2/S10(	3
GO:00107(	regulation of (	6/272	301/1867(	0.275957	0.501166	0.432317	IFRD1/TNF	6
GO:00004(	maturation of	1/272	22/18670	0.276066	0.501166	0.432317	EXOSC3	1
GO:00004(	cleavage invol	1/272	22/18670	0.276066	0.501166	0.432317	EXOSC3	1
GO:00020!	positive regul	1/272	22/18670	0.276066	0.501166	0.432317	HIF1A	1
GO:00060{	acetyl-CoA bic	1/272	22/18670	0.276066	0.501166	0.432317	DLD	1
GO:00072{	spermatid nuc	1/272	22/18670	0.276066	0.501166	0.432317	SRPK1	1
GO:00076:	mating behavi	1/272	22/18670	0.276066	0.501166	0.432317	HDAC4	1
GO:00092:	cobalamin me	1/272	22/18670	0.276066	0.501166	0.432317	LMBRD1	1

GO:00102	response to lit	1/272	22/18670	0.276066	0.501166	0.432317	PTGS2	1
GO:00108	regulation of r	1/272	22/18670	0.276066	0.501166	0.432317	HIF1A	1
GO:00218	cerebral corte	1/272	22/18670	0.276066	0.501166	0.432317	CDK5R1	1
GO:00218	hypothalamus	1/272	22/18670	0.276066	0.501166	0.432317	RAB3GAP1	1
GO:00220	central nervou	1/272	22/18670	0.276066	0.501166	0.432317	TLR2	1
GO:00220	telencephalon	1/272	22/18670	0.276066	0.501166	0.432317	CDK5R1	1
GO:00322	axon ensheath	1/272	22/18670	0.276066	0.501166	0.432317	TLR2	1
GO:00326	interleukin-5 p	1/272	22/18670	0.276066	0.501166	0.432317	IL17RA	1
GO:00326	regulation of i	1/272	22/18670	0.276066	0.501166	0.432317	IL17RA	1
GO:00327	positive regula	1/272	22/18670	0.276066	0.501166	0.432317	STAT1	1
GO:00332	regulation of p	1/272	22/18670	0.276066	0.501166	0.432317	HDAC4	1
GO:00343	diol metabolic	1/272	22/18670	0.276066	0.501166	0.432317	ASAH1	1
GO:00359	steroid hormo	1/272	22/18670	0.276066	0.501166	0.432317	NKX3-1	1
GO:00456	regulation of r	1/272	22/18670	0.276066	0.501166	0.432317	FADD	1
GO:00457	positive regula	1/272	22/18670	0.276066	0.501166	0.432317	PTGS2	1
GO:00460	cAMP metabo	1/272	22/18670	0.276066	0.501166	0.432317	PDE7A	1
GO:00466	positive regula	1/272	22/18670	0.276066	0.501166	0.432317	OSBPL8	1
GO:00483	immunoglobu	1/272	22/18670	0.276066	0.501166	0.432317	TNFSF13B	1
GO:00507	negative regul	1/272	22/18670	0.276066	0.501166	0.432317	TLR2	1
GO:00510	positive regula	1/272	22/18670	0.276066	0.501166	0.432317	HIF1A	1
GO:00514	maintenance o	1/272	22/18670	0.276066	0.501166	0.432317	TXN	1
GO:00605	negative regul	1/272	22/18670	0.276066	0.501166	0.432317	FADD	1
GO:00605	intestinal epit	1/272	22/18670	0.276066	0.501166	0.432317	HIF1A	1
GO:00713	cellular respon	1/272	22/18670	0.276066	0.501166	0.432317	IFIT1	1
GO:00726	interferon-gar	1/272	22/18670	0.276066	0.501166	0.432317	F2RL1	1
GO:00973	plasma memb	1/272	22/18670	0.276066	0.501166	0.432317	BIN3	1
GO:00996	protein localiz	1/272	22/18670	0.276066	0.501166	0.432317	RAP1A	1
GO:00996	neurotransmit	1/272	22/18670	0.276066	0.501166	0.432317	RAP1A	1
GO:19015	positive regula	1/272	22/18670	0.276066	0.501166	0.432317	HIF1A	1
GO:19030	negative regul	1/272	22/18670	0.276066	0.501166	0.432317	PICALM	1
GO:20010	regulation of c	1/272	22/18670	0.276066	0.501166	0.432317	DTX3L	1
GO:00336	cellular polysa	2/272	71/18670	0.276989	0.501556	0.432653	IRS2/PPP1	2
GO:00427	snRNA transcr	2/272	71/18670	0.276989	0.501556	0.432653	POLR2J/PC	2
GO:00507	regulation of k	2/272	71/18670	0.276989	0.501556	0.432653	EIF2AK4/H	2
GO:00714	cellular respon	2/272	71/18670	0.276989	0.501556	0.432653	GADD45A/	2
GO:19012	nucleoside ph	2/272	71/18670	0.276989	0.501556	0.432653	PDE7A/NT	2
GO:00431	positive regula	4/272	183/1867(	0.277521	0.502005	0.433041	F2RL1/S10	4
GO:00507	regulation of a	4/272	183/1867(	0.277521	0.502005	0.433041	IFRD1/TNF	4
GO:00181	peptidyl-threo	3/272	126/1867(	0.278453	0.503435	0.434274	LMTK2/CD	3
GO:00488	multicellular o	9/272	486/1867(	0.27891	0.504003	0.434764	GADD45G/	9
GO:00480	inositol lipid-n	4/272	184/1867(	0.280759	0.506826	0.4372	NKX3-1/F2	4
GO:00516	establishment	4/272	184/1867(	0.280759	0.506826	0.4372	GOLGA2/K	4
GO:00002	mitotic cytoki	2/272	72/18670	0.282394	0.508482	0.438628	UNC119/R	2
GO:00093	snRNA transcr	2/272	72/18670	0.282394	0.508482	0.438628	POLR2J/PC	2
GO:00380	signal transdu	2/272	72/18670	0.282394	0.508482	0.438628	IFI6/FADD	2
GO:00971	extrinsic apop	2/272	72/18670	0.282394	0.508482	0.438628	IFI6/FADD	2
GO:19037	regulation of e	2/272	72/18670	0.282394	0.508482	0.438628	UBE2D3/LI	2
GO:19030	regulation of l	6/272	304/1867(	0.283436	0.510098	0.440022	PAK2/SIRP	6

GO:00105!	positive regula	3/272	128/1867(	0.286375	0.510646	0.440495	PTGS2/THI	3
GO:00063!	regulation of t	1/272	23/18670	0.286625	0.510646	0.440495	CHD8	1
GO:00074:	axonal fascicu	1/272	23/18670	0.286625	0.510646	0.440495	CDK5R1	1
GO:00090(	aspartate fam	1/272	23/18670	0.286625	0.510646	0.440495	DLD	1
GO:00157	C4-dicarboxyl	1/272	23/18670	0.286625	0.510646	0.440495	ABAT	1
GO:00323:	positive regula	1/272	23/18670	0.286625	0.510646	0.440495	NFKBIA	1
GO:00323:	positive regula	1/272	23/18670	0.286625	0.510646	0.440495	NFKBIA	1
GO:00360:	CD8-positive,	1/272	23/18670	0.286625	0.510646	0.440495	EOMES	1
GO:00461:	pyrimidine nu	1/272	23/18670	0.286625	0.510646	0.440495	UPB1	1
GO:00487:	positive regula	1/272	23/18670	0.286625	0.510646	0.440495	TLR2	1
GO:00714:	hematopoieti	1/272	23/18670	0.286625	0.510646	0.440495	EIF2AK2	1
GO:00721:	metanephric t	1/272	23/18670	0.286625	0.510646	0.440495	STAT1	1
GO:00722:	regulation of r	1/272	23/18670	0.286625	0.510646	0.440495	STAT1	1
GO:00722	metanephric r	1/272	23/18670	0.286625	0.510646	0.440495	STAT1	1
GO:00723:	modified amir	1/272	23/18670	0.286625	0.510646	0.440495	FOLR3	1
GO:00901	regulation of r	1/272	23/18670	0.286625	0.510646	0.440495	LRRK2	1
GO:00903	positive regula	1/272	23/18670	0.286625	0.510646	0.440495	B2M	1
GO:00971!	neuronal sten	1/272	23/18670	0.286625	0.510646	0.440495	CDH2	1
GO:01060:	neuron projec	1/272	23/18670	0.286625	0.510646	0.440495	CDK5R1	1
GO:20007:	negative regul	1/272	23/18670	0.286625	0.510646	0.440495	RGS2	1
GO:00229(	electron trans	4/272	186/1867(	0.287252	0.510991	0.440793	DLD/NQO2	4
GO:00303(	negative regul	4/272	186/1867(	0.287252	0.510991	0.440793	IFRD1/SCG	4
GO:00550(	muscle cell de	4/272	186/1867(	0.287252	0.510991	0.440793	BIN3/RGS2	4
GO:00032(	cardiac ventri	2/272	73/18670	0.287796	0.511444	0.441184	HIF1A/CHD	2
GO:00436:	response to e:	2/272	73/18670	0.287796	0.511444	0.441184	IL4R/TRIM	2
GO:00421{	cellular keton	5/272	246/1867(	0.28935	0.513948	0.443343	PTGS2/PSN	5
GO:19054:	regulation of	4/272	187/1867(	0.290507	0.515745	0.444893	CDK5R1/R/	4
GO:00090(	glutamine fam	2/272	74/18670	0.293193	0.516449	0.445501	HAL/PADI4	2
GO:00091:	ribonucleosid	2/272	74/18670	0.293193	0.516449	0.445501	NT5C2/LRF	2
GO:00095{	detection of li	2/272	74/18670	0.293193	0.516449	0.445501	RGR/UNC1	2
GO:00324:	lysosome loca	2/272	74/18670	0.293193	0.516449	0.445501	IL4R/KIF1B	2
GO:00433(	CD4-positive,	2/272	74/18670	0.293193	0.516449	0.445501	IL4R/IL18	2
GO:00507:	negative regul	2/272	74/18670	0.293193	0.516449	0.445501	F2RL1/SRG	2
GO:00609!	regulation of (	2/272	74/18670	0.293193	0.516449	0.445501	CDK5R1/LF	2
GO:19001!	positive regula	2/272	74/18670	0.293193	0.516449	0.445501	PTGS2/DT)	2
GO:19016:	organic hydro:	2/272	74/18670	0.293193	0.516449	0.445501	CYP4F3/SY	2
GO:19030:	positive regula	2/272	74/18670	0.293193	0.516449	0.445501	THBS1/CLE	2
GO:00098!	negative regul	6/272	308/1867(	0.293477	0.516449	0.445501	TOB1/GOL	6
GO:00032:	cardiac ventri	3/272	130/1867(	0.294314	0.516449	0.445501	MDM4/HII	3
GO:00069!	nucleus organ	3/272	130/1867(	0.294314	0.516449	0.445501	SRPK2/SUI	3
GO:00421!	lipoprotein m	3/272	130/1867(	0.294314	0.516449	0.445501	PPM1B/RA	3
GO:19016!	glycosyl comp	3/272	130/1867(	0.294314	0.516449	0.445501	UPB1/NT5	3
GO:00323{	regulation of i	7/272	370/1867(	0.295159	0.516449	0.445501	TXN/PTGS:	7
GO:00017{	B cell apoptot	1/272	24/18670	0.297031	0.516449	0.445501	IRS2	1
GO:00022:	innate immun	1/272	24/18670	0.297031	0.516449	0.445501	CAMP	1
GO:00028(	regulation of i	1/272	24/18670	0.297031	0.516449	0.445501	PSMA1	1
GO:00031{	atrioventricul	1/272	24/18670	0.297031	0.516449	0.445501	MDM4	1
GO:00102{	response to le	1/272	24/18670	0.297031	0.516449	0.445501	PTGS2	1

GO:00148!	response to m	1/272	24/18670	0.297031	0.516449	0.445501	HIF1A	1
GO:00215‹	subpallium de	1/272	24/18670	0.297031	0.516449	0.445501	LRRK2	1
GO:0021‹	forebrain regi	1/272	24/18670	0.297031	0.516449	0.445501	EOMES	1
GO:00218!	cerebral corte	1/272	24/18670	0.297031	0.516449	0.445501	EOMES	1
GO:00443‹	fibroblast apo	1/272	24/18670	0.297031	0.516449	0.445501	CHD8	1
GO:00461‹	polyol catabol	1/272	24/18670	0.297031	0.516449	0.445501	SYNJ2	1
GO:0046‹	negative regul	1/272	24/18670	0.297031	0.516449	0.445501	IL4R	1
GO:00466!	decidualizatio	1/272	24/18670	0.297031	0.516449	0.445501	PTGS2	1
GO:00506‹	negative regul	1/272	24/18670	0.297031	0.516449	0.445501	PPM1B	1
GO:00509!	positive regul	1/272	24/18670	0.297031	0.516449	0.445501	IRS2	1
GO:00512‹	cytoplasmic se	1/272	24/18670	0.297031	0.516449	0.445501	NFKBIA	1
GO:00620(	secondary pal	1/272	24/18670	0.297031	0.516449	0.445501	CHD7	1
GO:00900‹	positive regul	1/272	24/18670	0.297031	0.516449	0.445501	CAMK1D	1
GO:00995(	modification c	1/272	24/18670	0.297031	0.516449	0.445501	PFN2	1
GO:19000‹	positive regul	1/272	24/18670	0.297031	0.516449	0.445501	OSBPL8	1
GO:19030‹	positive regul	1/272	24/18670	0.297031	0.516449	0.445501	GOLGA2	1
GO:19034‹	regulation of s	1/272	24/18670	0.297031	0.516449	0.445501	LRRK2	1
GO:19036‹	regulation of c	1/272	24/18670	0.297031	0.516449	0.445501	LRRK2	1
GO:19043‹	negative regul	1/272	24/18670	0.297031	0.516449	0.445501	PICALM	1
GO:20001‹	negative regul	1/272	24/18670	0.297031	0.516449	0.445501	RAPGEF1	1
GO:20010‹	regulation of e	1/272	24/18670	0.297031	0.516449	0.445501	THBS1	1
GO:00456(	positive regul	7/272	371/1867(	0.297459	0.516939	0.445923	RAPGEF1/	7
GO:00459‹	negative regul	5/272	249/1867(	0.297819	0.51731	0.446243	IFRD1/SCG	5
GO:00301‹	B cell differen	3/272	131/1867(	0.298289	0.517371	0.446296	PPP2R3C/	3
GO:19028!	microtubule c	3/272	131/1867(	0.298289	0.517371	0.446296	TACC3/GO	3
GO:00019(	negative regul	2/272	75/18670	0.298585	0.517371	0.446296	F2RL1/RFF	2
GO:00324‹	Rab protein si	2/272	75/18670	0.298585	0.517371	0.446296	RAB24/RA	2
GO:00432‹	negative regul	2/272	75/18670	0.298585	0.517371	0.446296	LMOD3/AI	2
GO:00423!	regulation of r	8/272	434/1867(	0.299627	0.518669	0.447416	PPP2R3C/(	8
GO:00487‹	gland develop	8/272	434/1867(	0.299627	0.518669	0.447416	NKX3-1/SL	8
GO:00331!	regulation of i	5/272	250/1867(	0.300651	0.520188	0.448726	TXN/PTGS	5
GO:00031!	endothelium c	3/272	132/1867(	0.302268	0.520474	0.448973	F2RL1/RAF	3
GO:00464‹	organophosph	3/272	132/1867(	0.302268	0.520474	0.448973	PDE7A/NT	3
GO:00723‹	monocarboxyl	3/272	132/1867(	0.302268	0.520474	0.448973	CYP4F3/IR	3
GO:00450‹	glycerolipid bi	5/272	251/1867(	0.303487	0.520474	0.448973	ACP6/ACSI	5
GO:00003‹	alternative mI	2/272	76/18670	0.30397	0.520474	0.448973	RBM7/RBN	2
GO:00004‹	autophagy of	2/272	76/18670	0.30397	0.520474	0.448973	HK2/HIF1A	2
GO:00019‹	negative regul	2/272	76/18670	0.30397	0.520474	0.448973	THBS1/STA	2
GO:00068‹	iron ion trans	2/272	76/18670	0.30397	0.520474	0.448973	LMTK2/B2	2
GO:00068!	drug transmer	2/272	76/18670	0.30397	0.520474	0.448973	RGS2/SLC3	2
GO:00074!	endoderm dev	2/272	76/18670	0.30397	0.520474	0.448973	EOMES/MI	2
GO:00305(	regulation of l	2/272	76/18670	0.30397	0.520474	0.448973	SRGN/HIF1	2
GO:00467‹	microtubule p	2/272	76/18670	0.30397	0.520474	0.448973	CDK5R1/G	2
GO:00469(	regulation of r	2/272	76/18670	0.30397	0.520474	0.448973	HK2/HEBP	2
GO:00617‹	mitochondrioi	2/272	76/18670	0.30397	0.520474	0.448973	HK2/HIF1A	2
GO:00170‹	protein impor	4/272	192/1867(	0.306852	0.520474	0.448973	PTGS2/RPI	4
GO:00431‹	receptor meta	4/272	192/1867(	0.306852	0.520474	0.448973	LMTK2/HII	4
GO:00463!	ribose phosph	4/272	192/1867(	0.306852	0.520474	0.448973	DLD/ACSL1	4

GO:00030	renal system p	1/272	25/18670	0.307286	0.520474	0.448973	F2RL1	1
GO:00092	mRNA transcr	1/272	25/18670	0.307286	0.520474	0.448973	HIF1A	1
GO:00310	hair follicle m	1/272	25/18670	0.307286	0.520474	0.448973	TGM3	1
GO:00316	regulation of l	1/272	25/18670	0.307286	0.520474	0.448973	LY96	1
GO:00335	histone H2A u	1/272	25/18670	0.307286	0.520474	0.448973	DTX3L	1
GO:00341	regulation of h	1/272	25/18670	0.307286	0.520474	0.448973	ABAT	1
GO:00352	ionotropic glu	1/272	25/18670	0.307286	0.520474	0.448973	CDK5R1	1
GO:00354	vesicle cargo l	1/272	25/18670	0.307286	0.520474	0.448973	PICALM	1
GO:00439	negative regul	1/272	25/18670	0.307286	0.520474	0.448973	RGS2	1
GO:00440	modulation by	1/272	25/18670	0.307286	0.520474	0.448973	EIF2AK4	1
GO:00456	negative regul	1/272	25/18670	0.307286	0.520474	0.448973	IL18	1
GO:00458	positive regula	1/272	25/18670	0.307286	0.520474	0.448973	EXOSC3	1
GO:00460	GTP metaboli	1/272	25/18670	0.307286	0.520474	0.448973	LRRK2	1
GO:00468	phosphatidyli	1/272	25/18670	0.307286	0.520474	0.448973	SYNJ2	1
GO:00509	leukocyte teth	1/272	25/18670	0.307286	0.520474	0.448973	GOLPH3	1
GO:00509	positive regula	1/272	25/18670	0.307286	0.520474	0.448973	F2RL1	1
GO:00511	glucose 6-pho	1/272	25/18670	0.307286	0.520474	0.448973	HK2	1
GO:00518	positive regula	1/272	25/18670	0.307286	0.520474	0.448973	MAP4K4	1
GO:00605	morphogenes	1/272	25/18670	0.307286	0.520474	0.448973	HIF1A	1
GO:00610	regulation of	1/272	25/18670	0.307286	0.520474	0.448973	RGS2	1
GO:00722	cell differentia	1/272	25/18670	0.307286	0.520474	0.448973	STAT1	1
GO:00722	metanephric r	1/272	25/18670	0.307286	0.520474	0.448973	STAT1	1
GO:19033	negative regul	1/272	25/18670	0.307286	0.520474	0.448973	RAP1A	1
GO:19035	negative regul	1/272	25/18670	0.307286	0.520474	0.448973	THBS1	1
GO:19048	excitatory syn	1/272	25/18670	0.307286	0.520474	0.448973	NPTN	1
GO:19058	negative regul	1/272	25/18670	0.307286	0.520474	0.448973	TLR2	1
GO:20003	positive regula	1/272	25/18670	0.307286	0.520474	0.448973	THBS1	1
GO:00508	regulation of T	6/272	314/1867(	0.308669	0.522568	0.450779	PAK2/SIRP	6
GO:20000	regulation of a	5/272	253/1867(	0.309173	0.522716	0.450907	SULF2/STA	5
GO:00068	nitric oxide bi	2/272	77/18670	0.309348	0.522716	0.450907	PTGS2/CLE	2
GO:00313	negative regul	2/272	77/18670	0.309348	0.522716	0.450907	RPL23/DTX	2
GO:00324	positive regula	2/272	77/18670	0.309348	0.522716	0.450907	STAT1/TLR	2
GO:00182	peptidyl-threo	3/272	134/1867(	0.310232	0.523864	0.451897	LMTK2/CD	3
GO:00199	second-messe	8/272	439/1867(	0.310323	0.523864	0.451897	TMBIM4/P	8
GO:00506	regulation of e	7/272	378/1867(	0.313662	0.527001	0.454603	NKX3-1/SU	7
GO:00018	liver developn	3/272	135/1867(	0.314216	0.527001	0.454603	SULF2/UPF	3
GO:00509	positive regula	3/272	135/1867(	0.314216	0.527001	0.454603	F2RL1/THE	3
GO:19033	positive regula	3/272	135/1867(	0.314216	0.527001	0.454603	GOLGA2/H	3
GO:00002	polysaccharid	2/272	78/18670	0.314717	0.527001	0.454603	IRS2/PPP1	2
GO:00083	associative lea	2/272	78/18670	0.314717	0.527001	0.454603	HIF1A/NPT	2
GO:00324	negative regul	2/272	78/18670	0.314717	0.527001	0.454603	HECW2/M	2
GO:00422	tissue regener	2/272	78/18670	0.314717	0.527001	0.454603	IFRD1/BIN	2
GO:00723	response to a	2/272	78/18670	0.314717	0.527001	0.454603	FADD/ABA	2
GO:00031	atrioventricula	1/272	26/18670	0.317391	0.527001	0.454603	MDM4	1
GO:00033	epithelial ciliu	1/272	26/18670	0.317391	0.527001	0.454603	ROPN1L	1
GO:00070	microtubule n	1/272	26/18670	0.317391	0.527001	0.454603	GOLGA2	1
GO:00107	regulation of r	1/272	26/18670	0.317391	0.527001	0.454603	THBS1	1
GO:00109	response to a	1/272	26/18670	0.317391	0.527001	0.454603	KIAA0319	1

GO:00301	positive regul	1/272	26/18670	0.317391	0.527001	0.454603	THBS1	1
GO:00316	negative regul	1/272	26/18670	0.317391	0.527001	0.454603	LRRK2	1
GO:00328	receptor bios	1/272	26/18670	0.317391	0.527001	0.454603	HIF1A	1
GO:00361	phosphatidyle	1/272	26/18670	0.317391	0.527001	0.454603	MBOAT1	1
GO:00435	regulation of	1/272	26/18670	0.317391	0.527001	0.454603	NKX3-1	1
GO:00481	regulation of	1/272	26/18670	0.317391	0.527001	0.454603	NPTN	1
GO:00509	regulation of	1/272	26/18670	0.317391	0.527001	0.454603	F2RL1	1
GO:00511	regulation of	1/272	26/18670	0.317391	0.527001	0.454603	GMFG	1
GO:00600	pharyngeal sy	1/272	26/18670	0.317391	0.527001	0.454603	NKX3-1	1
GO:00605	regulation of	1/272	26/18670	0.317391	0.527001	0.454603	FADD	1
GO:00607	cell differenti	1/272	26/18670	0.317391	0.527001	0.454603	EOMES	1
GO:00607	prostate glan	1/272	26/18670	0.317391	0.527001	0.454603	NKX3-1	1
GO:00620	regulation of	1/272	26/18670	0.317391	0.527001	0.454603	FADD	1
GO:00713	cellular respo	1/272	26/18670	0.317391	0.527001	0.454603	PRKAR2A	1
GO:00716	positive regul	1/272	26/18670	0.317391	0.527001	0.454603	CAMK1D	1
GO:00995	synaptic mem	1/272	26/18670	0.317391	0.527001	0.454603	NTNG2	1
GO:01501	positive regul	1/272	26/18670	0.317391	0.527001	0.454603	MAP4K4	1
GO:19000	positive regul	1/272	26/18670	0.317391	0.527001	0.454603	THBS1	1
GO:19032	regulation of	1/272	26/18670	0.317391	0.527001	0.454603	HIF1A	1
GO:19035	negative regul	1/272	26/18670	0.317391	0.527001	0.454603	HDAC4	1
GO:19050	regulation of	1/272	26/18670	0.317391	0.527001	0.454603	PICALM	1
GO:00507	regulation of	5/272	256/1867	0.317729	0.527314	0.454873	PAK2/UNC	5
GO:00066	steroid biosyn	4/272	196/1867	0.319997	0.530216	0.457377	HSD17B11	4
GO:00070	endosome org	2/272	79/18670	0.320077	0.530216	0.457377	AKTIP/EXO	2
GO:00216	nerve develop	2/272	79/18670	0.320077	0.530216	0.457377	SULF2/CHD	2
GO:00456	regulation of	2/272	79/18670	0.320077	0.530216	0.457377	THBS1/TN	2
GO:00066	glycerophosp	6/272	319/1867	0.321433	0.532213	0.459099	ACP6/SYN	6
GO:00346	cellular respo	3/272	137/1867	0.322189	0.532967	0.459749	PTGS2/TH	3
GO:00726	establishment	3/272	137/1867	0.322189	0.532967	0.459749	UBE2D3/H	3
GO:00102	endomembra	8/272	445/1867	0.323263	0.533772	0.460444	AKTIP/BIN	8
GO:00068	mitochondrial	5/272	258/1867	0.32345	0.533772	0.460444	UBE2D3/H	5
GO:00066	fatty acid met	7/272	383/1867	0.325335	0.533772	0.460444	DLD/PTGS	7
GO:00330	regulation of	2/272	80/18670	0.325427	0.533772	0.460444	TACC3/HE	2
GO:00434	regulation of	2/272	80/18670	0.325427	0.533772	0.460444	IFIT1/PFN	2
GO:00481	Golgi vesicle b	2/272	80/18670	0.325427	0.533772	0.460444	GOLPH3/G	2
GO:00970	dendritic spin	2/272	80/18670	0.325427	0.533772	0.460444	CDK5R1/L	2
GO:00466	alpha-beta T c	3/272	138/1867	0.326176	0.533772	0.460444	IL4R/EOM	3
GO:00610	hepaticobiliar	3/272	138/1867	0.326176	0.533772	0.460444	SULF2/UP	3
GO:00902	regulation of	5/272	259/1867	0.326315	0.533772	0.460444	PTGS2/RG	5
GO:00004	maturation of	1/272	27/18670	0.32735	0.533772	0.460444	EXOSC3	1
GO:00019	startle respon	1/272	27/18670	0.32735	0.533772	0.460444	CHD8	1
GO:00067	steroid catabo	1/272	27/18670	0.32735	0.533772	0.460444	HSD17B11	1
GO:00067	NADH regene	1/272	27/18670	0.32735	0.533772	0.460444	HK2	1
GO:00069	substrate-dep	1/272	27/18670	0.32735	0.533772	0.460444	TNFRSF12	1
GO:00181	peptidyl-argin	1/272	27/18670	0.32735	0.533772	0.460444	PADI4	1
GO:00340	protein localiz	1/272	27/18670	0.32735	0.533772	0.460444	GOLPH3	1
GO:00364	neuron death	1/272	27/18670	0.32735	0.533772	0.460444	HIF1A	1
GO:00425	serine phosph	1/272	27/18670	0.32735	0.533772	0.460444	CDK5R1	1

GO:00468ɛ	regulation of I	1/272	27/18670	0.32735	0.533772	0.460444	HIF1A	1
GO:00481ɛ	astrocyte activ	1/272	27/18670	0.32735	0.533772	0.460444	IFNGR1	1
GO:00486ɛ	positive regul	1/272	27/18670	0.32735	0.533772	0.460444	LMOD3	1
GO:00486(	collateral spro	1/272	27/18670	0.32735	0.533772	0.460444	IFRD1	1
GO:00508ɛ	positive regul	1/272	27/18670	0.32735	0.533772	0.460444	THBS1	1
GO:00602ɛ	regulation of f	1/272	27/18670	0.32735	0.533772	0.460444	EIF2AK4	1
GO:00616ɛ	canonical glyc	1/272	27/18670	0.32735	0.533772	0.460444	HK2	1
GO:00617ɛ	glucose catab	1/272	27/18670	0.32735	0.533772	0.460444	HK2	1
GO:00715ɛ	genetic imprir	1/272	27/18670	0.32735	0.533772	0.460444	ARID4A	1
GO:00722(	metanephric e	1/272	27/18670	0.32735	0.533772	0.460444	STAT1	1
GO:19021ɛ	regulation of c	1/272	27/18670	0.32735	0.533772	0.460444	HIF1A	1
GO:19052(	negative regul	1/272	27/18670	0.32735	0.533772	0.460444	RGS2	1
GO:00086ɛ	phospholipid I	5/272	260/1867(	0.329182	0.536512	0.462808	ACP6/SYNJ	5
GO:00071ɛ	transforming g	4/272	199/1867(	0.329885	0.536869	0.463115	USP15/THI	4
GO:00024ɛ	immune respo	3/272	139/1867(	0.330163	0.536869	0.463115	RAPGEF1/I	3
GO:00331ɛ	regulation of p	3/272	139/1867(	0.330163	0.536869	0.463115	TXN/PTGS2	3
GO:00380ɛ	Fc-gamma rec	3/272	139/1867(	0.330163	0.536869	0.463115	RAPGEF1/I	3
GO:00086ɛ	cellular amino	2/272	81/18670	0.330766	0.536869	0.463115	UPB1/ABA	2
GO:00226ɛ	extracellular n	2/272	81/18670	0.330766	0.536869	0.463115	EXOC8/MN	2
GO:00480ɛ	focal adhesion	2/272	81/18670	0.330766	0.536869	0.463115	THBS1/MA	2
GO:00487(	astrocyte diffe	2/272	81/18670	0.330766	0.536869	0.463115	IFNGR1/S1	2
GO:00607(	negative regul	2/272	81/18670	0.330766	0.536869	0.463115	F2RL1/RFF	2
GO:00338(	nucleoside bis	3/272	140/1867(	0.334149	0.539097	0.465037	DLD/ACSL1	3
GO:00338ɛ	ribonucleoside	3/272	140/1867(	0.334149	0.539097	0.465037	DLD/ACSL1	3
GO:00340ɛ	purine nucleo	3/272	140/1867(	0.334149	0.539097	0.465037	DLD/ACSL1	3
GO:00600ɛ	cardiac muscle	3/272	140/1867(	0.334149	0.539097	0.465037	RGS2/TNN	3
GO:00600ɛ	regulation of p	3/272	140/1867(	0.334149	0.539097	0.465037	RAB3GAP1	3
GO:00072ɛ	germ cell deve	5/272	262/1867(	0.334925	0.539097	0.465037	DLD/ROPN	5
GO:00158ɛ	organic hydro	5/272	262/1867(	0.334925	0.539097	0.465037	NFKBIA/OS	5
GO:00033ɛ	amino acid tra	2/272	82/18670	0.336093	0.539097	0.465037	RGS2/SLC3	2
GO:00551ɛ	regulation of c	2/272	82/18670	0.336093	0.539097	0.465037	RGS2/HDA	2
GO:00900ɛ	regulation of c	2/272	82/18670	0.336093	0.539097	0.465037	PTGS2/THI	2
GO:19059ɛ	positive regul	2/272	82/18670	0.336093	0.539097	0.465037	NKX3-1/NF	2
GO:00069ɛ	response to o	8/272	451/1867(	0.336301	0.539097	0.465037	TXN/PTGS2	8
GO:00015ɛ	prostaglandin	1/272	28/18670	0.337164	0.539097	0.465037	PTGS2	1
GO:00017ɛ	myeloid dendı	1/272	28/18670	0.337164	0.539097	0.465037	CLEC4D	1
GO:00069ɛ	hyperosmotic	1/272	28/18670	0.337164	0.539097	0.465037	RCSD1	1
GO:00080ɛ	motor neuron	1/272	28/18670	0.337164	0.539097	0.465037	NTNG2	1
GO:00082(	androgen met	1/272	28/18670	0.337164	0.539097	0.465037	HSD17B11	1
GO:00090(	glutamine fam	1/272	28/18670	0.337164	0.539097	0.465037	PADI4	1
GO:00090ɛ	aromatic amir	1/272	28/18670	0.337164	0.539097	0.465037	HAL	1
GO:00326ɛ	regulation of i	1/272	28/18670	0.337164	0.539097	0.465037	STAT1	1
GO:00336ɛ	regulation of c	1/272	28/18670	0.337164	0.539097	0.465037	EIF2AK2	1
GO:00351ɛ	embryonic hir	1/272	28/18670	0.337164	0.539097	0.465037	CHD7	1
GO:00455ɛ	regulation of I	1/272	28/18670	0.337164	0.539097	0.465037	PPP2R3C	1
GO:00464ɛ	prostanoid bic	1/272	28/18670	0.337164	0.539097	0.465037	PTGS2	1
GO:00485ɛ	positive regul	1/272	28/18670	0.337164	0.539097	0.465037	HDAC4	1
GO:00605ɛ	prostate glanc	1/272	28/18670	0.337164	0.539097	0.465037	NKX3-1	1

GO:00616:	glycolytic proc	1/272	28/18670	0.337164	0.539097	0.465037	HK2	1
GO:00616:	glycolytic proc	1/272	28/18670	0.337164	0.539097	0.465037	HK2	1
GO:00700:	histone lysine	1/272	28/18670	0.337164	0.539097	0.465037	UTY	1
GO:00711(	protein localiz	1/272	28/18670	0.337164	0.539097	0.465037	MCM8	1
GO:00955(	acetylcholine	1/272	28/18670	0.337164	0.539097	0.465037	CDK5R1	1
GO:19027	positive regul	1/272	28/18670	0.337164	0.539097	0.465037	HDAC4	1
GO:19907:	response to a	1/272	28/18670	0.337164	0.539097	0.465037	PTGS2	1
GO:00017:	morphogenes	3/272	141/1867(	0.338135	0.539674	0.465535	PSMD12/P	3
GO:00018:	epithelial to m	3/272	141/1867(	0.338135	0.539674	0.465535	PBLD/EOM	3
GO:00507(	negative regul	3/272	141/1867(	0.338135	0.539674	0.465535	F2RL1/SRG	3
GO:00705	protein localiz	3/272	141/1867(	0.338135	0.539674	0.465535	UBE2D3/H	3
GO:00069:	response to o	2/272	83/18670	0.341408	0.543513	0.468847	PTGS2/RCS	2
GO:00354:	protein localiz	2/272	83/18670	0.341408	0.543513	0.468847	RAP1A/NP	2
GO:00455	positive regul	2/272	83/18670	0.341408	0.543513	0.468847	IL4R/IL18	2
GO:00070:	Golgi organiza	3/272	142/1867(	0.342119	0.543513	0.468847	GOLPH3/G	3
GO:00302:	regulation of	4/272	203/1867(	0.343094	0.543513	0.468847	TOB1/SRG	4
GO:00070:	vacuolar trans	3/272	143/1867(	0.346101	0.543513	0.468847	AKTIP/LRR	3
GO:00308:	mammary gla	3/272	143/1867(	0.346101	0.543513	0.468847	HK2/HIF1A	3
GO:00310!	regulation of	3/272	143/1867(	0.346101	0.543513	0.468847	RPS6KA5/J	3
GO:00352!	regulation of	3/272	143/1867(	0.346101	0.543513	0.468847	F2RL1/PTG	3
GO:00977	regulation of	3/272	143/1867(	0.346101	0.543513	0.468847	F2RL1/PTG	3
GO:00060(	glucose metab	4/272	204/1867(	0.346399	0.543513	0.468847	PFKFB2/HK	4
GO:00313!	regulation of	4/272	204/1867(	0.346399	0.543513	0.468847	GOLGA2/R	4
GO:00347(	positive regul	4/272	204/1867(	0.346399	0.543513	0.468847	CLTCL1/IRS	4
GO:00457(	positive regul	4/272	204/1867(	0.346399	0.543513	0.468847	PTGS2/THI	4
GO:00105(	negative regul	2/272	84/18670	0.346709	0.543513	0.468847	GOLGA2/L	2
GO:00149:	regulation of	2/272	84/18670	0.346709	0.543513	0.468847	PLAU/HDA	2
GO:00454	myoblast diffe	2/272	84/18670	0.346709	0.543513	0.468847	IFRD1/IL18	2
GO:00003!	mRNA 5'-splic	1/272	29/18670	0.346835	0.543513	0.468847	PSIP1	1
GO:00068!	extracellular t	1/272	29/18670	0.346835	0.543513	0.468847	ROPN1L	1
GO:00072(	nitric oxide m	1/272	29/18670	0.346835	0.543513	0.468847	THBS1	1
GO:00076:	adult walking	1/272	29/18670	0.346835	0.543513	0.468847	CHD7	1
GO:00103!	histone mono	1/272	29/18670	0.346835	0.543513	0.468847	DTX3L	1
GO:00106(	epithelial stru	1/272	29/18670	0.346835	0.543513	0.468847	PBLD	1
GO:00108	regulation of	1/272	29/18670	0.346835	0.543513	0.468847	HDAC4	1
GO:00165:	histone deme	1/272	29/18670	0.346835	0.543513	0.468847	UTY	1
GO:00217!	cerebral corte	1/272	29/18670	0.346835	0.543513	0.468847	CDK5R1	1
GO:00305:	positive regul	1/272	29/18670	0.346835	0.543513	0.468847	THBS1	1
GO:00311:	positive regul	1/272	29/18670	0.346835	0.543513	0.468847	CDK5R1	1
GO:00313(	N-terminal pr	1/272	29/18670	0.346835	0.543513	0.468847	PPM1B	1
GO:00343:	cell junction m	1/272	29/18670	0.346835	0.543513	0.468847	F2RL1	1
GO:00350:	positive regul	1/272	29/18670	0.346835	0.543513	0.468847	F2RL1	1
GO:00487:	epidermis mo	1/272	29/18670	0.346835	0.543513	0.468847	TGM3	1
GO:00610!	negative regul	1/272	29/18670	0.346835	0.543513	0.468847	SH3BP5L	1
GO:00701(	interleukin-6-	1/272	29/18670	0.346835	0.543513	0.468847	STAT1	1
GO:00901(	establishment	1/272	29/18670	0.346835	0.543513	0.468847	GOLPH3	1
GO:01200:	negative regul	1/272	29/18670	0.346835	0.543513	0.468847	PFN2	1
GO:19000:	regulation of	1/272	29/18670	0.346835	0.543513	0.468847	PFN2	1

GO:19026:	positive regul	1/272	29/18670	0.346835	0.543513	0.468847	CAMK1D	1
GO:19038	positive regul	1/272	29/18670	0.346835	0.543513	0.468847	THBS1	1
GO:20001	negative regul	1/272	29/18670	0.346835	0.543513	0.468847	KIAA0319	1
GO:20005	negative regul	1/272	29/18670	0.346835	0.543513	0.468847	IL4R	1
GO:00305	adult behavio	3/272	144/1867(	0.350081	0.548115	0.472816	FADD/CHD	3
GO:00351	regulation of t	3/272	144/1867(	0.350081	0.548115	0.472816	F2RL1/PTG	3
GO:00723	organelle tran	2/272	85/18670	0.351996	0.550383	0.474773	KIF1B/HIF1	2
GO:19047	regulation of v	2/272	85/18670	0.351996	0.550383	0.474773	NQO2/MN	2
GO:19908	vascular smoc	2/272	85/18670	0.351996	0.550383	0.474773	NQO2/MN	2
GO:00024	Fc receptor m	3/272	145/1867(	0.354059	0.551129	0.475417	RAPGEF1/I	3
GO:00072	neuromuscula	1/272	30/18670	0.356366	0.551129	0.475417	KIF1B	1
GO:00190	reproductive	1/272	30/18670	0.356366	0.551129	0.475417	HDAC4	1
GO:00326	interferon-alp	1/272	30/18670	0.356366	0.551129	0.475417	STAT1	1
GO:00327	positive regul	1/272	30/18670	0.356366	0.551129	0.475417	TLR2	1
GO:00327	positive regul	1/272	30/18670	0.356366	0.551129	0.475417	CLEC7A	1
GO:00344	snRNA 3'-end	1/272	30/18670	0.356366	0.551129	0.475417	EXOSC3	1
GO:00350	positive regul	1/272	30/18670	0.356366	0.551129	0.475417	RPS6KA5	1
GO:00427	long-chain fat	1/272	30/18670	0.356366	0.551129	0.475417	PTGS2	1
GO:00431	peptide catab	1/272	30/18670	0.356366	0.551129	0.475417	ANPEP	1
GO:00447	modulation by	1/272	30/18670	0.356366	0.551129	0.475417	EIF2AK4	1
GO:00463	regulation of f	1/272	30/18670	0.356366	0.551129	0.475417	IRS2	1
GO:00465	regulation of v	1/272	30/18670	0.356366	0.551129	0.475417	TRIM25	1
GO:00466	regulation of a	1/272	30/18670	0.356366	0.551129	0.475417	IL18	1
GO:00482	response to p	1/272	30/18670	0.356366	0.551129	0.475417	THBS1	1
GO:00508	negative regul	1/272	30/18670	0.356366	0.551129	0.475417	MNDA	1
GO:00618	microtubule o	1/272	30/18670	0.356366	0.551129	0.475417	ARPC5	1
GO:00709	protein K11-li	1/272	30/18670	0.356366	0.551129	0.475417	UBE2D3	1
GO:00714	cellular respo	1/272	30/18670	0.356366	0.551129	0.475417	RPL26	1
GO:00903	positive regul	1/272	30/18670	0.356366	0.551129	0.475417	CDK5R1	1
GO:19048	beta-catenin-T	1/272	30/18670	0.356366	0.551129	0.475417	TLE4	1
GO:19051	response to a	1/272	30/18670	0.356366	0.551129	0.475417	CDK5R1	1
GO:19051	cellular respo	1/272	30/18670	0.356366	0.551129	0.475417	CDK5R1	1
GO:19054	negative regul	1/272	30/18670	0.356366	0.551129	0.475417	PICALM	1
GO:19056	regulation of p	1/272	30/18670	0.356366	0.551129	0.475417	NTNG2	1
GO:00346	cellular carbo	2/272	86/18670	0.357269	0.552044	0.476205	IRS2/PPP1	2
GO:00905	regulation of r	2/272	86/18670	0.357269	0.552044	0.476205	HK2/HEBP	2
GO:00459	positive regul	5/272	270/1867(	0.357971	0.552743	0.476809	RPS6KA3/T	5
GO:00425	response to h	3/272	146/1867(	0.358034	0.552743	0.476809	TXN/STAT1	3
GO:00018	embryonic pla	2/272	87/18670	0.362526	0.555075	0.47882	EOMES/HI	2
GO:00303	flagellated sp	2/272	87/18670	0.362526	0.555075	0.47882	ROPN1L/SI	2
GO:00977	sperm motility	2/272	87/18670	0.362526	0.555075	0.47882	ROPN1L/SI	2
GO:19021	regulation of l	5/272	272/1867(	0.363745	0.555075	0.47882	PPP2R3C/I	5
GO:00075	blood coagula	6/272	336/1867(	0.365308	0.555075	0.47882	PLAU/F2RI	6
GO:00321	negative regul	7/272	400/1867(	0.365477	0.555075	0.47882	PLAU/THB	7
GO:00019	synaptic trans	1/272	31/18670	0.365758	0.555075	0.47882	PTGS2	1
GO:00026	positive regul	1/272	31/18670	0.365758	0.555075	0.47882	PTGS2	1
GO:00032	muscle hypert	1/272	31/18670	0.365758	0.555075	0.47882	HDAC4	1
GO:00064	protein deme	1/272	31/18670	0.365758	0.555075	0.47882	UTY	1

GO:00065(	GPI anchor bio	1/272	31/18670	0.365758	0.555075	0.47882	MPPE1	1
GO:00082:	protein dealky	1/272	31/18670	0.365758	0.555075	0.47882	UTY	1
GO:00091!	purine ribonu	1/272	31/18670	0.365758	0.555075	0.47882	PDE7A	1
GO:00099!	proximal/dista	1/272	31/18670	0.365758	0.555075	0.47882	PBX2	1
GO:00105	regulation of p	1/272	31/18670	0.365758	0.555075	0.47882	ABAT	1
GO:00107:	negative regul	1/272	31/18670	0.365758	0.555075	0.47882	PBLD	1
GO:00148	cardiac muscle	1/272	31/18670	0.365758	0.555075	0.47882	HDAC4	1
GO:00148!	cardiac muscle	1/272	31/18670	0.365758	0.555075	0.47882	HDAC4	1
GO:00301	sphingolipid c	1/272	31/18670	0.365758	0.555075	0.47882	ASAH1	1
GO:00304:	sleep	1/272	31/18670	0.365758	0.555075	0.47882	IL18	1
GO:00327:	positive regula	1/272	31/18670	0.365758	0.555075	0.47882	HIF1A	1
GO:00331!	response to A	1/272	31/18670	0.365758	0.555075	0.47882	PTGS2	1
GO:00336	osteoblast pro	1/272	31/18670	0.365758	0.555075	0.47882	EIF2AK2	1
GO:00346(	ncRNA catabo	1/272	31/18670	0.365758	0.555075	0.47882	EXOSC3	1
GO:00357(	endothelial ce	1/272	31/18670	0.365758	0.555075	0.47882	THBS1	1
GO:00400:	positive regula	1/272	31/18670	0.365758	0.555075	0.47882	CHD7	1
GO:00433(	regulation of r	1/272	31/18670	0.365758	0.555075	0.47882	IL4R	1
GO:00434!	regulation of c	1/272	31/18670	0.365758	0.555075	0.47882	HIF1A	1
GO:00457:	positive regula	1/272	31/18670	0.365758	0.555075	0.47882	CDK5R1	1
GO:00514	positive regula	1/272	31/18670	0.365758	0.555075	0.47882	F2RL1	1
GO:00900:	relaxation of r	1/272	31/18670	0.365758	0.555075	0.47882	RGS2	1
GO:00906!	walking behav	1/272	31/18670	0.365758	0.555075	0.47882	CHD7	1
GO:00971(	postsynaptic c	1/272	31/18670	0.365758	0.555075	0.47882	CDH2	1
GO:00991:	regulation of p	1/272	31/18670	0.365758	0.555075	0.47882	NTNG2	1
GO:19021	regulation of v	1/272	31/18670	0.365758	0.555075	0.47882	TRIM25	1
GO:19022!	regulation of i	1/272	31/18670	0.365758	0.555075	0.47882	RPL26	1
GO:19028!	positive regula	1/272	31/18670	0.365758	0.555075	0.47882	HIF1A	1
GO:20004(	positive regula	1/272	31/18670	0.365758	0.555075	0.47882	FADD	1
GO:20007	positive regula	1/272	31/18670	0.365758	0.555075	0.47882	DTX3L	1
GO:00027!	negative regul	3/272	148/1867(	0.365973	0.555075	0.47882	F2RL1/SRG	3
GO:00515!	response to ca	3/272	148/1867(	0.365973	0.555075	0.47882	THBS1/ALC	3
GO:00067(	water-soluble	2/272	88/18670	0.367767	0.55589	0.479523	LMBRD1/V	2
GO:00459:	positive regula	2/272	88/18670	0.367767	0.55589	0.479523	F2RL1/IL4F	2
GO:00508:	defense respo	2/272	88/18670	0.367767	0.55589	0.479523	F2RL1/CAN	2
GO:00700!	chemokine-m	2/272	88/18670	0.367767	0.55589	0.479523	HIF1A/CXC	2
GO:00971!	execution pha	2/272	88/18670	0.367767	0.55589	0.479523	PAK2/RFFL	2
GO:01060:	neuron projec	2/272	88/18670	0.367767	0.55589	0.479523	CDK5R1/LF	2
GO:19024:	chloride trans	2/272	88/18670	0.367767	0.55589	0.479523	CLCC1/SLC	2
GO:19033:	negative regul	2/272	88/18670	0.367767	0.55589	0.479523	RPL23/DTX	2
GO:00092(	cellular respor	3/272	149/1867(	0.369937	0.558931	0.482147	EIF2AK2/EI	3
GO:00224(	regulation of c	7/272	402/1867(	0.370231	0.559137	0.482324	PAK2/SIRP	7
GO:00199:	cyclic-nucleoti	4/272	212/1867(	0.372843	0.560857	0.483808	PRKAR2A/	4
GO:00071!	adenylate cycl	2/272	89/18670	0.372991	0.560857	0.483808	RGS2/OXE	2
GO:00324:	negative regul	2/272	89/18670	0.372991	0.560857	0.483808	HECW2/M	2
GO:00610!	somite develo	2/272	89/18670	0.372991	0.560857	0.483808	PPP2R3A/I	2
GO:00019:	response to ar	1/272	32/18670	0.375013	0.560857	0.483808	RGS2	1
GO:00033:	metanephros	1/272	32/18670	0.375013	0.560857	0.483808	STAT1	1
GO:00065(	GPI anchor me	1/272	32/18670	0.375013	0.560857	0.483808	MPPE1	1

GO:00076:	long-term me	1/272	32/18670	0.375013	0.560857	0.483808	EIF2AK4	1
GO:00092(	ribonucleotid	1/272	32/18670	0.375013	0.560857	0.483808	PDE7A	1
GO:00108(	regulation of	1/272	32/18670	0.375013	0.560857	0.483808	CHD7	1
GO:00328:	regulation of	1/272	32/18670	0.375013	0.560857	0.483808	IL18	1
GO:00330(	regulation of	1/272	32/18670	0.375013	0.560857	0.483808	IL4R	1
GO:00423:	vasodilation	1/272	32/18670	0.375013	0.560857	0.483808	RGS2	1
GO:00459(	positive regul	1/272	32/18670	0.375013	0.560857	0.483808	PTGS2	1
GO:00550:	negative regul	1/272	32/18670	0.375013	0.560857	0.483808	RGS2	1
GO:00611:	negative regul	1/272	32/18670	0.375013	0.560857	0.483808	RGS2	1
GO:00613(	heart trabecul	1/272	32/18670	0.375013	0.560857	0.483808	CHD7	1
GO:00702(	protein homo	1/272	32/18670	0.375013	0.560857	0.483808	ALOX5AP	1
GO:00705:	regulation of	1/272	32/18670	0.375013	0.560857	0.483808	MAP4K4	1
GO:00713!	cellular respo	1/272	32/18670	0.375013	0.560857	0.483808	IL4R	1
GO:19022:	regulation of	1/272	32/18670	0.375013	0.560857	0.483808	LRRK2	1
GO:19039!	positive regul	1/272	32/18670	0.375013	0.560857	0.483808	UBE2D3	1
GO:20007:	positive regul	1/272	32/18670	0.375013	0.560857	0.483808	F2RL1	1
GO:00074:	axon guidance	5/272	276/1867(	0.375298	0.561045	0.48397	RPS6KA5/N	5
GO:00974(	neuron projec	5/272	277/1867(	0.378186	0.564094	0.4866	RPS6KA5/N	5
GO:00016!	metanephros	2/272	90/18670	0.378198	0.564094	0.4866	NKX3-1/ST	2
GO:00432!	regulation of	2/272	90/18670	0.378198	0.564094	0.4866	IRS2/PPP1	2
GO:00513(	chromosome	2/272	90/18670	0.378198	0.564094	0.4866	TACC3/HE(	2
GO:00716:	mononuclear	2/272	90/18670	0.378198	0.564094	0.4866	THBS1/S1(	2
GO:00075!	hemostasis	6/272	341/1867(	0.378294	0.564094	0.4866	PLAU/F2RL	6
GO:00508:	coagulation	6/272	342/1867(	0.380892	0.566362	0.488557	PLAU/F2RL	6
GO:00091(	nucleotide bic	5/272	278/1867(	0.381074	0.566362	0.488557	DLD/PTGS:	5
GO:00001(	activation of M	3/272	152/1867(	0.3818	0.566362	0.488557	THBS1/MA	3
GO:00162(	regulation of s	3/272	152/1867(	0.3818	0.566362	0.488557	RGS2/HDA	3
GO:00080:	cell recognitio	4/272	215/1867(	0.382746	0.566362	0.488557	CLEC7A/FC	4
GO:00604(	mesenchyme	5/272	279/1867(	0.383962	0.566362	0.488557	STAT1/PBL	5
GO:00063:	7-methylguan	1/272	33/18670	0.384134	0.566362	0.488557	POLR2J	1
GO:00091(	nucleoside ca	1/272	33/18670	0.384134	0.566362	0.488557	UPB1	1
GO:00107:	protein kinase	1/272	33/18670	0.384134	0.566362	0.488557	LRRK2	1
GO:00140:	response to is	1/272	33/18670	0.384134	0.566362	0.488557	FADD	1
GO:00162(	negative regul	1/272	33/18670	0.384134	0.566362	0.488557	LRRK2	1
GO:00308!	granulocyte di	1/272	33/18670	0.384134	0.566362	0.488557	EVI2B	1
GO:00311:	positive regul	1/272	33/18670	0.384134	0.566362	0.488557	CDK5R1	1
GO:00357:	positive regul	1/272	33/18670	0.384134	0.566362	0.488557	HIF1A	1
GO:00360(	positive regul	1/272	33/18670	0.384134	0.566362	0.488557	HIF1A	1
GO:00432:	response to m	1/272	33/18670	0.384134	0.566362	0.488557	FADD	1
GO:00439:	exonucleolytic	1/272	33/18670	0.384134	0.566362	0.488557	EXOSC3	1
GO:00451!	regulation of i	1/272	33/18670	0.384134	0.566362	0.488557	EXOSC3	1
GO:00466:	alpha-beta T c	1/272	33/18670	0.384134	0.566362	0.488557	IL18	1
GO:00488:	erythrocyte d	1/272	33/18670	0.384134	0.566362	0.488557	ARID4A	1
GO:00488!	brain morpho	1/272	33/18670	0.384134	0.566362	0.488557	CDH2	1
GO:00507:	positive regul	1/272	33/18670	0.384134	0.566362	0.488557	ORM2	1
GO:00514(	positive regul	1/272	33/18670	0.384134	0.566362	0.488557	GOLGA2	1
GO:19012:	negative regul	1/272	33/18670	0.384134	0.566362	0.488557	PPM1B	1
GO:19017!	negative regul	1/272	33/18670	0.384134	0.566362	0.488557	RFFL	1

GO:20007!	positive regul:	1/272	33/18670	0.384134	0.566362	0.488557	RPS6KA5	1
GO:20010:	positive regul:	1/272	33/18670	0.384134	0.566362	0.488557	LRRK2	1
GO:00510:	actin filament	3/272	153/1867(	0.385743	0.568498	0.490399	PFN2/PHA	3
GO:00357:	CD4-positive,	2/272	92/18670	0.388556	0.572405	0.49377	IL4R/IL18	2
GO:00075:	muscle organ	7/272	410/1867(	0.38928	0.572842	0.494146	SAP30/BIN	7
GO:00464:	glycerophospl	4/272	217/1867(	0.389338	0.572842	0.494146	ACP6/SYNJ	4
GO:00509:	regulation of (	4/272	217/1867(	0.389338	0.572842	0.494146	F2RL1/THE	4
GO:00610:	membrane fu:	3/272	154/1867(	0.389682	0.573077	0.494349	FOLR3/STX	3
GO:19012!	nucleoside ph	5/272	282/1867(	0.392622	0.573077	0.494349	DLD/PTGS:	5
GO:00100(	glial cell differ	4/272	218/1867(	0.392631	0.573077	0.494349	IFNGR1/S1	4
GO:00197:	calcium-medi:	4/272	218/1867(	0.392631	0.573077	0.494349	TMBIM4/F	4
GO:00060!	tricarboxylic a	1/272	34/18670	0.393123	0.573077	0.494349	DLD	1
GO:00064(	tRNA export fi	1/272	34/18670	0.393123	0.573077	0.494349	RAE1	1
GO:00065:	glutamate me	1/272	34/18670	0.393123	0.573077	0.494349	HAL	1
GO:00074:	salivary gland	1/272	34/18670	0.393123	0.573077	0.494349	NKX3-1	1
GO:00094!	7-methylguan	1/272	34/18670	0.393123	0.573077	0.494349	POLR2J	1
GO:00109:	positive regul:	1/272	34/18670	0.393123	0.573077	0.494349	PPP1R12A	1
GO:00164!	peptide horm(	1/272	34/18670	0.393123	0.573077	0.494349	MME	1
GO:00326(	regulation of i	1/272	34/18670	0.393123	0.573077	0.494349	IL18	1
GO:00362(	RNA capping	1/272	34/18670	0.393123	0.573077	0.494349	POLR2J	1
GO:00435:	regulation of I	1/272	34/18670	0.393123	0.573077	0.494349	RPL26	1
GO:00459:	negative regul	1/272	34/18670	0.393123	0.573077	0.494349	RAP1A	1
GO:00607!	cell fate comn	1/272	34/18670	0.393123	0.573077	0.494349	EOMES	1
GO:00706:	response to in	1/272	34/18670	0.393123	0.573077	0.494349	IL4R	1
GO:00714:	tRNA-containi	1/272	34/18670	0.393123	0.573077	0.494349	RAE1	1
GO:00718:	adrenergic rec	1/272	34/18670	0.393123	0.573077	0.494349	RGS2	1
GO:01404	signaling rece	1/272	34/18670	0.393123	0.573077	0.494349	MME	1
GO:19018(	regulation of r	3/272	155/1867(	0.393613	0.573077	0.494349	RGS2/HDA	3
GO:00000	autophagosor	2/272	93/18670	0.393707	0.573077	0.494349	RAB3GAP1	2
GO:00030:	regulation of s	2/272	93/18670	0.393707	0.573077	0.494349	F2RL1/MM	2
GO:00075!	body fluid sec	2/272	93/18670	0.393707	0.573077	0.494349	HK2/HIF1A	2
GO:00332:	response to vi	2/272	93/18670	0.393707	0.573077	0.494349	PTGS2/TRI	2
GO:00466:	regulation of a	2/272	93/18670	0.393707	0.573077	0.494349	IL4R/IL18	2
GO:00421:	regulation of T	3/272	156/1867(	0.397539	0.576351	0.497173	TNFSF13B/	3
GO:00435:	regulation of I	3/272	156/1867(	0.397539	0.576351	0.497173	PTGS2/THI	3
GO:00486:	regulation of r	3/272	156/1867(	0.397539	0.576351	0.497173	RGS2/HDA	3
GO:00316	regulation of p	5/272	284/1867(	0.398391	0.576351	0.497173	PFN2/MDN	5
GO:00464!	glycerolipid m	7/272	414/1867(	0.398815	0.576351	0.497173	ACP6/ACSI	7
GO:00069(	vesicle fusion	2/272	94/18670	0.398838	0.576351	0.497173	STX10/TAF	2
GO:00609!	kidney morph	2/272	94/18670	0.398838	0.576351	0.497173	STAT1/LRR	2
GO:00701(	regulation of I	2/272	94/18670	0.398838	0.576351	0.497173	SRGN/HIF1	2
GO:01101	regulation of I	2/272	94/18670	0.398838	0.576351	0.497173	SRGN/HIF1	2
GO:19024:	protein localiz	2/272	94/18670	0.398838	0.576351	0.497173	RAP1A/NP	2
GO:00071:	transmembrai	6/272	349/1867(	0.399085	0.576351	0.497173	USP15/TOI	6
GO:00487(	mesenchymal	4/272	220/1867(	0.399209	0.576351	0.497173	STAT1/PBL	4
GO:00349:	response to ei	5/272	285/1867(	0.401273	0.576351	0.497173	THBS1/EIF:	5
GO:00017(	neuron migrat	3/272	157/1867(	0.401457	0.576351	0.497173	KIAA0319/	3
GO:00615:	actin filament	3/272	157/1867(	0.401457	0.576351	0.497173	PFN2/PHA	3

GO:00002(	mitochondrial	1/272	35/18670	0.40198	0.576351	0.497173	LRRK2	1
GO:00002!	nuclear-transc	1/272	35/18670	0.40198	0.576351	0.497173	EXOSC3	1
GO:00004(	maturation of	1/272	35/18670	0.40198	0.576351	0.497173	RPS16	1
GO:00018{	receptor recy	1/272	35/18670	0.40198	0.576351	0.497173	LMTK2	1
GO:00018!	maternal plac	1/272	35/18670	0.40198	0.576351	0.497173	PTGS2	1
GO:00032(	endocardial cu	1/272	35/18670	0.40198	0.576351	0.497173	MDM4	1
GO:00032:	cardiac atrium	1/272	35/18670	0.40198	0.576351	0.497173	MDM4	1
GO:00102!	multicellular c	1/272	35/18670	0.40198	0.576351	0.497173	LRRK2	1
GO:00156!	organic cation	1/272	35/18670	0.40198	0.576351	0.497173	SLC25A20	1
GO:00190	viral release fr	1/272	35/18670	0.40198	0.576351	0.497173	TRIM25	1
GO:00328(	receptor catal	1/272	35/18670	0.40198	0.576351	0.497173	DTX3L	1
GO:00333(	secretory grar	1/272	35/18670	0.40198	0.576351	0.497173	SRGN	1
GO:00358!	exit from host	1/272	35/18670	0.40198	0.576351	0.497173	TRIM25	1
GO:00358!	exit from host	1/272	35/18670	0.40198	0.576351	0.497173	TRIM25	1
GO:00359(	aorta morpho	1/272	35/18670	0.40198	0.576351	0.497173	CHD7	1
GO:00464(	membrane lip	1/272	35/18670	0.40198	0.576351	0.497173	ASAH1	1
GO:00513!	negative regul	1/272	35/18670	0.40198	0.576351	0.497173	LRRK2	1
GO:00603:	regulation of t	1/272	35/18670	0.40198	0.576351	0.497173	FADD	1
GO:00704:	nucleotide-bir	1/272	35/18670	0.40198	0.576351	0.497173	NFKBIA	1
GO:00988(	regulation of r	1/272	35/18670	0.40198	0.576351	0.497173	F2RL1	1
GO:01101:	negative regul	1/272	35/18670	0.40198	0.576351	0.497173	STAT1	1
GO:19040:	positive regula	1/272	35/18670	0.40198	0.576351	0.497173	CDK5R1	1
GO:00192:	regulation of f	2/272	96/18670	0.40904	0.582963	0.502877	PTGS2/IRS	2
GO:00302:	megakaryocyt	2/272	96/18670	0.40904	0.582963	0.502877	THBS1/TNI	2
GO:00973:	response to ar	2/272	96/18670	0.40904	0.582963	0.502877	KIF1B/RAP	2
GO:00988	vesicle-mediat	2/272	96/18670	0.40904	0.582963	0.502877	GOLPH3/E.	2
GO:19050:	autophagosor	2/272	96/18670	0.40904	0.582963	0.502877	RAB3GAP1	2
GO:00226(	regulation of c	8/272	484/1867(	0.40905	0.582963	0.502877	ZMYM2/IF	8
GO:00454	fat cell differe	4/272	223/1867(	0.409053	0.582963	0.502877	PTGS2/RG!	4
GO:00072!	receptor signa	3/272	159/1867(	0.409273	0.582963	0.502877	STAT1/CDI	3
GO:00023{	mucosal immu	1/272	36/18670	0.410709	0.582963	0.502877	CAMP	1
GO:00030!	renal water hc	1/272	36/18670	0.410709	0.582963	0.502877	PRKAR2A	1
GO:00062:	pyrimidine nu	1/272	36/18670	0.410709	0.582963	0.502877	UPB1	1
GO:00066!	bile acid biosy	1/272	36/18670	0.410709	0.582963	0.502877	OSBPL9	1
GO:00091(	purine ribonu	1/272	36/18670	0.410709	0.582963	0.502877	NT5C2	1
GO:00148(	release of seq	1/272	36/18670	0.410709	0.582963	0.502877	CHD7	1
GO:00161{	snRNA proces	1/272	36/18670	0.410709	0.582963	0.502877	EXOSC3	1
GO:00309	regulation of v	1/272	36/18670	0.410709	0.582963	0.502877	HIF1A	1
GO:00335	transferrin tra	1/272	36/18670	0.410709	0.582963	0.502877	LMTK2	1
GO:00337(	response to gl	1/272	36/18670	0.410709	0.582963	0.502877	PRKAR2A	1
GO:00351:	hindlimb mor	1/272	36/18670	0.410709	0.582963	0.502877	CHD7	1
GO:00358	nucleotide-bir	1/272	36/18670	0.410709	0.582963	0.502877	NFKBIA	1
GO:00439(	positive regula	1/272	36/18670	0.410709	0.582963	0.502877	HDAC4	1
GO:00510:	tRNA transpor	1/272	36/18670	0.410709	0.582963	0.502877	RAE1	1
GO:00903:	regulation of p	1/272	36/18670	0.410709	0.582963	0.502877	CDK5R1	1
GO:19010!	negative regul	1/272	36/18670	0.410709	0.582963	0.502877	IFI6	1
GO:20012	negative regul	1/272	36/18670	0.410709	0.582963	0.502877	IFI6	1
GO:00069	regulation of s	2/272	97/18670	0.414109	0.586144	0.505621	RGS2/HDA	2

GO:00070	cell-substrate	2/272	97/18670	0.414109	0.586144	0.505621	THBS1/MA	2
GO:00107	negative regul	2/272	97/18670	0.414109	0.586144	0.505621	IFRD1/CDK	2
GO:00427	mitochondrial	2/272	97/18670	0.414109	0.586144	0.505621	DLD/COX7	2
GO:01501	cell-substrate	2/272	97/18670	0.414109	0.586144	0.505621	THBS1/MA	2
GO:19908	response to ch	2/272	97/18670	0.414109	0.586144	0.505621	HIF1A/CXC	2
GO:19908	cellular respor	2/272	97/18670	0.414109	0.586144	0.505621	HIF1A/CXC	2
GO:00071	cell-matrix adl	4/272	225/1867	0.415598	0.586704	0.506104	PLAU/THB	4
GO:00420	gliogenesis	5/272	290/1867	0.415661	0.586704	0.506104	IFNGR1/S1	5
GO:19013	regulation of v	7/272	422/1867	0.417876	0.586704	0.506104	PTGS2/THI	7
GO:00020	cell migration	2/272	98/18670	0.419156	0.586704	0.506104	PTGS2/THI	2
GO:00066	protein target	2/272	98/18670	0.419156	0.586704	0.506104	UBE2D3/LI	2
GO:00300	contractile act	2/272	98/18670	0.419156	0.586704	0.506104	PFN2/PHA	2
GO:00427	ATP synthesis	2/272	98/18670	0.419156	0.586704	0.506104	DLD/COX7	2
GO:00431	stress fiber as	2/272	98/18670	0.419156	0.586704	0.506104	PFN2/PHA	2
GO:00027	positive regula	1/272	37/18670	0.419311	0.586704	0.506104	EXOSC3	1
GO:00028	positive regula	1/272	37/18670	0.419311	0.586704	0.506104	EXOSC3	1
GO:00074	salivary gland	1/272	37/18670	0.419311	0.586704	0.506104	NKX3-1	1
GO:00091	purine nucleo	1/272	37/18670	0.419311	0.586704	0.506104	NT5C2	1
GO:00106	negative regul	1/272	37/18670	0.419311	0.586704	0.506104	RGS2	1
GO:00302	heparan sulfat	1/272	37/18670	0.419311	0.586704	0.506104	SULF2	1
GO:00302	hyaluronan m	1/272	37/18670	0.419311	0.586704	0.506104	ABCC5	1
GO:00344	response to fl	1/272	37/18670	0.419311	0.586704	0.506104	PTGS2	1
GO:00380	peptidyl-tyros	1/272	37/18670	0.419311	0.586704	0.506104	BMX	1
GO:00423	positive regula	1/272	37/18670	0.419311	0.586704	0.506104	PTGS2	1
GO:00432	positive regula	1/272	37/18670	0.419311	0.586704	0.506104	F2RL1	1
GO:00468	phospholipid	1/272	37/18670	0.419311	0.586704	0.506104	SYNJ2	1
GO:00515	histone H3-K9	1/272	37/18670	0.419311	0.586704	0.506104	ARID4A	1
GO:00523	modulation by	1/272	37/18670	0.419311	0.586704	0.506104	TRIM25	1
GO:00712	cellular respor	1/272	37/18670	0.419311	0.586704	0.506104	MMP9	1
GO:00715	dopaminergic	1/272	37/18670	0.419311	0.586704	0.506104	HIF1A	1
GO:00900	positive regula	1/272	37/18670	0.419311	0.586704	0.506104	PTGS2	1
GO:00989	postsynaptic s	1/272	37/18670	0.419311	0.586704	0.506104	CDK5R1	1
GO:19035	release of seq	1/272	37/18670	0.419311	0.586704	0.506104	CHD7	1
GO:20002	regulation of a	1/272	37/18670	0.419311	0.586704	0.506104	GMFG	1
GO:20004	positive regula	1/272	37/18670	0.419311	0.586704	0.506104	FADD	1
GO:00436	dicarboxylic ac	2/272	99/18670	0.424182	0.592708	0.511283	DLD/HAL	2
GO:00518	modulation of	2/272	99/18670	0.424182	0.592708	0.511283	EIF2AK2/EI	2
GO:00703	cellular respor	2/272	99/18670	0.424182	0.592708	0.511283	TXN/LRRK2	2
GO:00070	vacuole organ	3/272	163/1867	0.424809	0.592708	0.511283	AKTIP/RAB	3
GO:00312	biomineral tiss	3/272	163/1867	0.424809	0.592708	0.511283	PTGS2/SRC	3
GO:01101	biomineralizat	3/272	163/1867	0.424809	0.592708	0.511283	PTGS2/SRC	3
GO:00018	blastocyst forı	1/272	38/18670	0.427788	0.592708	0.511283	EOMES	1
GO:00032	physiological r	1/272	38/18670	0.427788	0.592708	0.511283	RGS2	1
GO:00033	physiological c	1/272	38/18670	0.427788	0.592708	0.511283	RGS2	1
GO:00060	acetyl-CoA me	1/272	38/18670	0.427788	0.592708	0.511283	DLD	1
GO:00072	Wnt signaling	1/272	38/18670	0.427788	0.592708	0.511283	TNRC6B	1
GO:00109	positive regula	1/272	38/18670	0.427788	0.592708	0.511283	IRS2	1
GO:00165	histone phosp	1/272	38/18670	0.427788	0.592708	0.511283	RPS6KA5	1

GO:00323!	regulation of I	1/272	38/18670	0.427788	0.592708	0.511283	HIF1A	1
GO:00326:	interleukin-17	1/272	38/18670	0.427788	0.592708	0.511283	IL18	1
GO:00463:	negative regul	1/272	38/18670	0.427788	0.592708	0.511283	F2RL1	1
GO:00466:	negative regul	1/272	38/18670	0.427788	0.592708	0.511283	IL4R	1
GO:00466:	negative regul	1/272	38/18670	0.427788	0.592708	0.511283	MAP4K4	1
GO:00468:	regulation of p	1/272	38/18670	0.427788	0.592708	0.511283	TXN	1
GO:00480(	insulin-like gro	1/272	38/18670	0.427788	0.592708	0.511283	NKX3-1	1
GO:00507:	positive regul	1/272	38/18670	0.427788	0.592708	0.511283	ORM2	1
GO:00515!	positive regul	1/272	38/18670	0.427788	0.592708	0.511283	RAB3GAP1	1
GO:00610	cell growth inv	1/272	38/18670	0.427788	0.592708	0.511283	RGS2	1
GO:00970(	ncRNA export	1/272	38/18670	0.427788	0.592708	0.511283	RAE1	1
GO:19040:	positive regul	1/272	38/18670	0.427788	0.592708	0.511283	THBS1	1
GO:00323!	positive regul	4/272	229/1867(	0.428639	0.593018	0.511551	PTGS2/UBI	4
GO:00066:	fatty acid bios	3/272	164/1867(	0.428672	0.593018	0.511551	PTGS2/HSI	3
GO:00072(	phospholipase	2/272	100/1867(	0.429185	0.593018	0.511551	F2RL1/LTB	2
GO:00600:	excitatory pos	2/272	100/1867(	0.429185	0.593018	0.511551	RAB3GAP1	2
GO:00608	artery develop	2/272	100/1867(	0.429185	0.593018	0.511551	NKX3-1/CH	2
GO:00901:	organelle mer	2/272	100/1867(	0.429185	0.593018	0.511551	STX10/TAF	2
GO:01200:	positive regul	2/272	100/1867(	0.429185	0.593018	0.511551	F2RL1/HD/	2
GO:00162:	macroautopha	5/272	295/1867(	0.429995	0.593717	0.512154	EXOC8/CD	5
GO:00024(	adaptive imm	6/272	361/1867(	0.430194	0.593717	0.512154	IL4R/EXOS	6
GO:00224:	cellular proce	6/272	361/1867(	0.430194	0.593717	0.512154	DLD/ROPN	6
GO:00315:	actin cytoskel	2/272	101/1867(	0.434164	0.596807	0.514819	GMFG/PH/	2
GO:00508:	defense respo	2/272	101/1867(	0.434164	0.596807	0.514819	CAMP/TLR	2
GO:19018!	positive regul	2/272	101/1867(	0.434164	0.596807	0.514819	LRRN3/M/	2
GO:00022!	organ or tissu	1/272	39/18670	0.436141	0.596807	0.514819	CAMP	1
GO:00072!	activation of J	1/272	39/18670	0.436141	0.596807	0.514819	MAP3K2	1
GO:00108:	regulation of	1/272	39/18670	0.436141	0.596807	0.514819	NFKBIA	1
GO:00147	negative regul	1/272	39/18670	0.436141	0.596807	0.514819	RGS2	1
GO:00300	muscle filame	1/272	39/18670	0.436141	0.596807	0.514819	TNNI2	1
GO:00332:	actin-myosin f	1/272	39/18670	0.436141	0.596807	0.514819	TNNI2	1
GO:00358!	vascular smoo	1/272	39/18670	0.436141	0.596807	0.514819	EPC1	1
GO:00424:	dopamine me	1/272	39/18670	0.436141	0.596807	0.514819	ABAT	1
GO:00430(	Golgi to plasm	1/272	39/18670	0.436141	0.596807	0.514819	GOLPH3	1
GO:00459:	positive regul	1/272	39/18670	0.436141	0.596807	0.514819	EXOSC3	1
GO:00487:	regulation of	1/272	39/18670	0.436141	0.596807	0.514819	TLR2	1
GO:00550!	acylglycerol h	1/272	39/18670	0.436141	0.596807	0.514819	IL18	1
GO:00703:	triglyceride hc	1/272	39/18670	0.436141	0.596807	0.514819	IL18	1
GO:00725:	pyrimidine-co	1/272	39/18670	0.436141	0.596807	0.514819	UPB1	1
GO:19021:	negative regul	1/272	39/18670	0.436141	0.596807	0.514819	LRRK2	1
GO:19022:	regulation of i	1/272	39/18670	0.436141	0.596807	0.514819	RPL26	1
GO:19045!	positive regul	1/272	39/18670	0.436141	0.596807	0.514819	PTGS2	1
GO:19055:	regulation of r	1/272	39/18670	0.436141	0.596807	0.514819	THBS1	1
GO:20006	positive regul	1/272	39/18670	0.436141	0.596807	0.514819	HIF1A	1
GO:00003(	response to re	4/272	232/1867(	0.438372	0.599627	0.517252	TXN/STAT1	4
GO:00000:	regulation of	2/272	102/1867(	0.439121	0.599917	0.517502	GADD45A/	2
GO:00215	cerebellum de	2/272	102/1867(	0.439121	0.599917	0.517502	CDK5R1/AI	2
GO:00353:	peptidyl-tyros	2/272	102/1867(	0.439121	0.599917	0.517502	PTP4A2/PT	2

GO:00486(	reproductive s	7/272	431/1867(	0.439261	0.599917	0.517502	NKX3-1/PT	7
GO:00424	hormone met	4/272	233/1867(	0.441605	0.601791	0.519118	HSD17B11	4
GO:00973(	response to al	4/272	233/1867(	0.441605	0.601791	0.519118	RGS2/S10(	4
GO:00316(	response to n	8/272	499/1867(	0.442229	0.601791	0.519118	PTGS2/ACS	8
GO:00346	cellular respo	3/272	168/1867(	0.444028	0.601791	0.519118	TXN/LRRK	3
GO:00519	regulation of	2/272	103/1867(	0.444054	0.601791	0.519118	TACC3/HE	2
GO:00703	regulation of	5/272	300/1867(	0.44426	0.601791	0.519118	F2RL1/RAF	5
GO:0017(	cell fate deter	1/272	40/18670	0.444373	0.601791	0.519118	IFRD1	1
GO:00019	postsynaptic r	1/272	40/18670	0.444373	0.601791	0.519118	CDH2	1
GO:00028	negative regul	1/272	40/18670	0.444373	0.601791	0.519118	IL4R	1
GO:00067	NADH metabo	1/272	40/18670	0.444373	0.601791	0.519118	HK2	1
GO:00090	aromatic amir	1/272	40/18670	0.444373	0.601791	0.519118	HAL	1
GO:00091	cyclic nucleoti	1/272	40/18670	0.444373	0.601791	0.519118	PDE7A	1
GO:00140	dopamine sec	1/272	40/18670	0.444373	0.601791	0.519118	ABAT	1
GO:00140	regulation of	1/272	40/18670	0.444373	0.601791	0.519118	ABAT	1
GO:00217	cerebral corte	1/272	40/18670	0.444373	0.601791	0.519118	CDK5R1	1
GO:00320(	positive regul	1/272	40/18670	0.444373	0.601791	0.519118	GOLPH3	1
GO:00420	protein refold	1/272	40/18670	0.444373	0.601791	0.519118	B2M	1
GO:00458	positive regul	1/272	40/18670	0.444373	0.601791	0.519118	RGS2	1
GO:00466	negative regul	1/272	40/18670	0.444373	0.601791	0.519118	RGS2	1
GO:00722	metanephric r	1/272	40/18670	0.444373	0.601791	0.519118	STAT1	1
GO:19010(	guanosine-cor	1/272	40/18670	0.444373	0.601791	0.519118	LRRK2	1
GO:20000(	regulation of	1/272	40/18670	0.444373	0.601791	0.519118	PICALM	1
GO:00506	epithelial cell	7/272	434/1867(	0.446366	0.604028	0.521048	NKX3-1/SU	7
GO:00614	reproductive s	7/272	434/1867(	0.446366	0.604028	0.521048	NKX3-1/PT	7
GO:00456	regulation of	3/272	169/1867(	0.447842	0.605562	0.522371	PPP2R3C/I	3
GO:00976	receptor signa	3/272	169/1867(	0.447842	0.605562	0.522371	STAT1/CD	3
GO:00301	extracellular n	6/272	368/1867(	0.448234	0.60586	0.522629	SULF2/THE	6
GO:00026	negative regul	7/272	435/1867(	0.448731	0.606151	0.52288	MNDA/TH	7
GO:00068	post-Golgi ves	2/272	104/1867(	0.448963	0.606151	0.52288	GOLPH3/E	2
GO:00192	sensory perce	2/272	104/1867(	0.448963	0.606151	0.52288	PTGS2/MN	2
GO:00345	cellular respo	5/272	302/1867(	0.449942	0.607241	0.52382	TXN/HIF1A	5
GO:00430(	extracellular s	6/272	369/1867(	0.450802	0.60817	0.524622	SULF2/THE	6
GO:00194	triglyceride bi	1/272	41/18670	0.452485	0.60928	0.525579	ACSL1	1
GO:00504	positive regul	1/272	41/18670	0.452485	0.60928	0.525579	POLR2J	1
GO:00971	ruffle assembl	1/272	41/18670	0.452485	0.60928	0.525579	PFN2	1
GO:00988	modulation of	1/272	41/18670	0.452485	0.60928	0.525579	LRRK2	1
GO:01403	lipid export fr	1/272	41/18670	0.452485	0.60928	0.525579	NKX3-1	1
GO:00344(	nucleobase-co	2/272	105/1867(	0.453847	0.610882	0.52696	PDE7A/NT	2
GO:00316(	cellular respo	4/272	237/1867(	0.454484	0.611506	0.527499	PTGS2/EIF	4
GO:00032(	cardiac cham	3/272	171/1867(	0.455439	0.612558	0.528406	MDM4/HI	3
GO:00465	regulation of	4/272	238/1867(	0.457689	0.615351	0.530816	F2RL1/RAF	4
GO:00033(	cardiac muscl	2/272	106/1867(	0.458707	0.615828	0.531227	RGS2/HDA	2
GO:19040	regulation of	2/272	106/1867(	0.458707	0.615828	0.531227	GADD45A/	2
GO:00091	purine ribonu	3/272	172/1867(	0.459221	0.615828	0.531227	DLD/ACSL1	3
GO:00066	phosphatidic	1/272	42/18670	0.46048	0.615828	0.531227	ACP6	1
GO:00105	regulation of	1/272	42/18670	0.46048	0.615828	0.531227	GOLGA2	1
GO:00106	negative regul	1/272	42/18670	0.46048	0.615828	0.531227	HDAC4	1

GO:00157(	bicarbonate tr	1/272	42/18670	0.46048	0.615828	0.531227	SLC26A8	1
GO:00160:	snRNA metab	1/272	42/18670	0.46048	0.615828	0.531227	EXOSC3	1
GO:00171	stem cell divis	1/272	42/18670	0.46048	0.615828	0.531227	EVI2B	1
GO:00330(	regulation of r	1/272	42/18670	0.46048	0.615828	0.531227	IL4R	1
GO:00905(	RNA phospho	1/272	42/18670	0.46048	0.615828	0.531227	EXOSC3	1
GO:00970:	dendritic cell	1/272	42/18670	0.46048	0.615828	0.531227	F2RL1	1
GO:19016!	glycosyl comp	1/272	42/18670	0.46048	0.615828	0.531227	UPB1	1
GO:20012:	regulation of r	1/272	42/18670	0.46048	0.615828	0.531227	PHACTR1	1
GO:00435(	regulation of r	2/272	107/1867(	0.463541	0.619221	0.534154	RGS2/HDA	2
GO:00513	regulation of	2/272	107/1867(	0.463541	0.619221	0.534154	HIF1A/LRR	2
GO:00987	ncRNA transcr	2/272	107/1867(	0.463541	0.619221	0.534154	POLR2J/PC	2
GO:00464	phosphatidyli	3/272	174/1867(	0.466751	0.620968	0.535661	SYNJ2/MPI	3
GO:20012!	positive regul	3/272	174/1867(	0.466751	0.620968	0.535661	RPS6KA5/J	3
GO:00068:	chloride trans	2/272	108/1867(	0.46835	0.620968	0.535661	CLCC1/SLC	2
GO:00090(	fatty acid cata	2/272	108/1867(	0.46835	0.620968	0.535661	CYP4F3/IR!	2
GO:00512:	spindle assem	2/272	108/1867(	0.46835	0.620968	0.535661	GOLGA2/C	2
GO:00066!	prostanoid me	1/272	43/18670	0.468357	0.620968	0.535661	PTGS2	1
GO:00066!	prostaglandin	1/272	43/18670	0.468357	0.620968	0.535661	PTGS2	1
GO:00086:	intrinsic apopt	1/272	43/18670	0.468357	0.620968	0.535661	HIF1A	1
GO:00171!	regulation of	1/272	43/18670	0.468357	0.620968	0.535661	RAB3GAP1	1
GO:00456	positive regul	1/272	43/18670	0.468357	0.620968	0.535661	TLR2	1
GO:00457	negative regul	1/272	43/18670	0.468357	0.620968	0.535661	NFKBIA	1
GO:00457:	positive regul	1/272	43/18670	0.468357	0.620968	0.535661	TNFRSF12	1
GO:00464(	neutral lipid b	1/272	43/18670	0.468357	0.620968	0.535661	ACSL1	1
GO:00464(	acylglycerol bi	1/272	43/18670	0.468357	0.620968	0.535661	ACSL1	1
GO:00464:	phosphatidic	1/272	43/18670	0.468357	0.620968	0.535661	ACP6	1
GO:00550:	negative regul	1/272	43/18670	0.468357	0.620968	0.535661	RGS2	1
GO:00702!	sarcoplasmic r	1/272	43/18670	0.468357	0.620968	0.535661	CHD7	1
GO:00723	sulfur compou	1/272	43/18670	0.468357	0.620968	0.535661	SLC26A8	1
GO:00990!	presynapse as	1/272	43/18670	0.468357	0.620968	0.535661	NTNG2	1
GO:19019	positive regul	1/272	43/18670	0.468357	0.620968	0.535661	RPS6KA5	1
GO:00030:	vascular proce	3/272	175/1867(	0.470499	0.623575	0.53791	F2RL1/PTG	3
GO:00148!	striated muscl	2/272	109/1867(	0.473134	0.626831	0.540719	RGS2/HDA	2
GO:00019:	regulation of	3/272	176/1867(	0.474235	0.627034	0.540894	THBS1/STA	3
GO:00903:	positive regul	3/272	176/1867(	0.474235	0.627034	0.540894	PTGS2/UBI	3
GO:00031!	endocardial cu	1/272	44/18670	0.476121	0.627034	0.540894	MDM4	1
GO:00072:	dopamine rec	1/272	44/18670	0.476121	0.627034	0.540894	LRRK2	1
GO:00140(	oligodendrocy	1/272	44/18670	0.476121	0.627034	0.540894	TLR2	1
GO:00140	glutamate sec	1/272	44/18670	0.476121	0.627034	0.540894	RAB3GAP1	1
GO:00180:	peptidyl-lysin	1/272	44/18670	0.476121	0.627034	0.540894	ARID4A	1
GO:00329(	regulation of	1/272	44/18670	0.476121	0.627034	0.540894	RAP1A	1
GO:00341(	positive regul	1/272	44/18670	0.476121	0.627034	0.540894	IL18	1
GO:00353(	positive regul	1/272	44/18670	0.476121	0.627034	0.540894	PPP1R12A	1
GO:00443:	cell-cell adhes	1/272	44/18670	0.476121	0.627034	0.540894	CDH2	1
GO:00617!	leukocyte adh	1/272	44/18670	0.476121	0.627034	0.540894	GOLPH3	1
GO:00900!	negative regul	1/272	44/18670	0.476121	0.627034	0.540894	THBS1	1
GO:19035:	protein localiz	1/272	44/18670	0.476121	0.627034	0.540894	RAP1A	1
GO:19035:	negative regul	1/272	44/18670	0.476121	0.627034	0.540894	LRRK2	1

GO:20004(	regulation of T	1/272	44/18670	0.476121	0.627034	0.540894	FADD	1
GO:00193:	hexose metab	4/272	244/1867(	0.47678	0.627669	0.541442	PFKFB2/HK	4
GO:00066(	glycolipid met	2/272	110/1867(	0.477891	0.628898	0.542502	ASAH1/MF	2
GO:00072(	Ras protein si	7/272	448/1867(	0.479289	0.630502	0.543886	F2RL1/RAE	7
GO:00511{	cofactor meta	7/272	449/1867(	0.481622	0.632637	0.545727	LMBRD1/D	7
GO:00064(	mRNA export	2/272	111/1867(	0.482623	0.632637	0.545727	UPF2/RAE:	2
GO:00148!	muscle hypert	2/272	111/1867(	0.482623	0.632637	0.545727	RGS2/HDA	2
GO:00220:	metencephalo	2/272	111/1867(	0.482623	0.632637	0.545727	CDK5R1/A	2
GO:00714:	mRNA-contair	2/272	111/1867(	0.482623	0.632637	0.545727	UPF2/RAE:	2
GO:00726:	lymphocyte m	2/272	111/1867(	0.482623	0.632637	0.545727	CKLF/FADD	2
GO:00986(	inorganic anio	2/272	111/1867(	0.482623	0.632637	0.545727	CLCC1/SLC	2
GO:19035(	liposaccharide	2/272	111/1867(	0.482623	0.632637	0.545727	ASAH1/MF	2
GO:00019:	blood vessel r	1/272	45/18670	0.483771	0.632637	0.545727	CHD7	1
GO:00028:	negative regul	1/272	45/18670	0.483771	0.632637	0.545727	IL4R	1
GO:00165:	histone ubiqui	1/272	45/18670	0.483771	0.632637	0.545727	DTX3L	1
GO:00323(	intracellular li	1/272	45/18670	0.483771	0.632637	0.545727	SLC25A20	1
GO:00427:	intrinsic apop	1/272	45/18670	0.483771	0.632637	0.545727	RPL26	1
GO:00455{	negative regul	1/272	45/18670	0.483771	0.632637	0.545727	IL4R	1
GO:00485:	thymus devel	1/272	45/18670	0.483771	0.632637	0.545727	FADD	1
GO:00902:	negative regul	1/272	45/18670	0.483771	0.632637	0.545727	MAP4K4	1
GO:00066:	protein expor	3/272	179/1867(	0.485371	0.634027	0.546926	TXN/UPF2/	3
GO:00083(	regulation of	3/272	179/1867(	0.485371	0.634027	0.546926	IFRD1/TNF	3
GO:00487:	tissue remode	3/272	179/1867(	0.485371	0.634027	0.546926	HIF1A/IL1{	3
GO:00457(	regulation of	6/272	383/1867(	0.486444	0.634943	0.547716	PTGS2/THI	6
GO:00020(	columnar/cub	2/272	112/1867(	0.487328	0.634943	0.547716	HIF1A/CDH	2
GO:00032:	cardiac septur	2/272	112/1867(	0.487328	0.634943	0.547716	MDM4/CH	2
GO:00311(	microtubule p	2/272	112/1867(	0.487328	0.634943	0.547716	CDK5R1/G	2
GO:00423(	molting cycle	2/272	112/1867(	0.487328	0.634943	0.547716	PTGS2/TGI	2
GO:00426:	hair cycle	2/272	112/1867(	0.487328	0.634943	0.547716	PTGS2/TGI	2
GO:00432:	response to al	2/272	112/1867(	0.487328	0.634943	0.547716	FADD/ABA	2
GO:00344:	ncRNA proces	6/272	384/1867(	0.488964	0.636384	0.548959	RPS28/RPS	6
GO:00435:	blood vessel e	3/272	180/1867(	0.489058	0.636384	0.548959	PTGS2/THI	3
GO:00435	protein acylat	4/272	248/1867(	0.489366	0.636384	0.548959	RPS6KA5/D	4
GO:00015(	neurotransmit	1/272	46/18670	0.49131	0.636384	0.548959	RGS2	1
GO:00026:	regulation of	1/272	46/18670	0.49131	0.636384	0.548959	PTGS2	1
GO:00071!	heterophilic c	1/272	46/18670	0.49131	0.636384	0.548959	CDH2	1
GO:00140:	response to a	1/272	46/18670	0.49131	0.636384	0.548959	RGS2	1
GO:00197:	antibacterial	1/272	46/18670	0.49131	0.636384	0.548959	CAMP	1
GO:00336:	regulation of	1/272	46/18670	0.49131	0.636384	0.548959	PLAU	1
GO:00432:	apoptotic cell	1/272	46/18670	0.49131	0.636384	0.548959	THBS1	1
GO:00457:	negative regul	1/272	46/18670	0.49131	0.636384	0.548959	ABAT	1
GO:00507(	regulation of i	1/272	46/18670	0.49131	0.636384	0.548959	ORM2	1
GO:00713!	cellular respo	1/272	46/18670	0.49131	0.636384	0.548959	STAT1	1
GO:00716:	regulation of r	1/272	46/18670	0.49131	0.636384	0.548959	THBS1	1
GO:00991:	presynapse or	1/272	46/18670	0.49131	0.636384	0.548959	NTNG2	1
GO:19035{	regulation of l	1/272	46/18670	0.49131	0.636384	0.548959	THBS1	1
GO:00609(	regulation of g	2/272	113/1867(	0.492006	0.636838	0.549351	POLR2J/RA	2
GO:00703:	ERK1 and ERK	5/272	317/1867(	0.49202	0.636838	0.549351	F2RL1/RAF	5

GO:00513:	meiotic cell cy	4/272	249/1867(	0.492493	0.636984	0.549477	RBM7/GOI	4
GO:00715(	cellular respor	4/272	249/1867(	0.492493	0.636984	0.549477	USP15/THI	4
GO:00015:	cilium or flage	2/272	114/1867(	0.496657	0.641081	0.553011	ROPN1L/SI	2
GO:00602:	cilium-depenc	2/272	114/1867(	0.496657	0.641081	0.553011	ROPN1L/SI	2
GO:19907:	protein localiz	5/272	319/1867(	0.497546	0.641081	0.553011	GOLPH3/K	5
GO:00022(	somatic recon	1/272	47/18670	0.498739	0.641081	0.553011	EXOSC3	1
GO:00022(	somatic divers	1/272	47/18670	0.498739	0.641081	0.553011	EXOSC3	1
GO:00082(	bile acid meta	1/272	47/18670	0.498739	0.641081	0.553011	OSBPL9	1
GO:00149:	positive regul	1/272	47/18670	0.498739	0.641081	0.553011	HDAC4	1
GO:00305:	collagen catab	1/272	47/18670	0.498739	0.641081	0.553011	MMP9	1
GO:00425!	neuron matur	1/272	47/18670	0.498739	0.641081	0.553011	LRRK2	1
GO:00433(	mast cell degr	1/272	47/18670	0.498739	0.641081	0.553011	IL4R	1
GO:00451!	isotype switch	1/272	47/18670	0.498739	0.641081	0.553011	EXOSC3	1
GO:00474!	vesicle transp	1/272	47/18670	0.498739	0.641081	0.553011	KIF1B	1
GO:00488(	genitalia deve	1/272	47/18670	0.498739	0.641081	0.553011	CHD7	1
GO:00609:	endocrine hor	1/272	47/18670	0.498739	0.641081	0.553011	NKX3-1	1
GO:00613:	trabecula mor	1/272	47/18670	0.498739	0.641081	0.553011	CHD7	1
GO:00622:	protein localiz	1/272	47/18670	0.498739	0.641081	0.553011	RAP1A	1
GO:20012:	regulation of (	1/272	47/18670	0.498739	0.641081	0.553011	IFI6	1
GO:00109:	cellular comp	2/272	115/1867(	0.501281	0.643881	0.555426	TLR2/LMO	2
GO:19043:	regulation of	2/272	115/1867(	0.501281	0.643881	0.555426	RAP1A/PIC	2
GO:00420!	T cell prolifera	3/272	184/1867(	0.503675	0.645104	0.556482	TNFSF13B/	3
GO:00434(	negative regul	3/272	184/1867(	0.503675	0.645104	0.556482	F2RL1/RAF	3
GO:00313:	negative regul	4/272	253/1867(	0.504922	0.645104	0.556482	TOB1/GOL	4
GO:00063(	DNA modifica	2/272	116/1867(	0.505878	0.645104	0.556482	EXOSC3/TI	2
GO:00075(	cell aging	2/272	116/1867(	0.505878	0.645104	0.556482	MME/B2M	2
GO:19001:	regulation of	2/272	116/1867(	0.505878	0.645104	0.556482	PTGS2/DT)	2
GO:00020(	chondrocyte c	1/272	48/18670	0.506061	0.645104	0.556482	SULF2	1
GO:00022:	mast cell activ	1/272	48/18670	0.506061	0.645104	0.556482	IL4R	1
GO:00075:	neuromuscula	1/272	48/18670	0.506061	0.645104	0.556482	LRRK2	1
GO:00080:	anterograde a	1/272	48/18670	0.506061	0.645104	0.556482	KIF1B	1
GO:00163:	calcium-deper	1/272	48/18670	0.506061	0.645104	0.556482	CDH2	1
GO:00308!	prostate glanc	1/272	48/18670	0.506061	0.645104	0.556482	NKX3-1	1
GO:00308(	cortical cytosk	1/272	48/18670	0.506061	0.645104	0.556482	PPP2R3C	1
GO:00352:	exocrine syste	1/272	48/18670	0.506061	0.645104	0.556482	NKX3-1	1
GO:00436:	ncRNA 3'-end	1/272	48/18670	0.506061	0.645104	0.556482	EXOSC3	1
GO:0043!	regulation of	1/272	48/18670	0.506061	0.645104	0.556482	RGS2	1
GO:00459:	negative regul	1/272	48/18670	0.506061	0.645104	0.556482	HDAC4	1
GO:00550:	ventricular ca	1/272	48/18670	0.506061	0.645104	0.556482	CHD7	1
GO:00616	histone H3-K9	1/272	48/18670	0.506061	0.645104	0.556482	ARID4A	1
GO:00726(	interleukin-6	1/272	48/18670	0.506061	0.645104	0.556482	F2RL1	1
GO:20000!	negative regul	1/272	48/18670	0.506061	0.645104	0.556482	RPL23	1
GO:00073(	gastrulation	3/272	185/1867(	0.507296	0.645749	0.557038	DLD/EOMI	3
GO:00092(	ribonucleotid	3/272	185/1867(	0.507296	0.645749	0.557038	DLD/ACSL1	3
GO:00452:	cell-cell juncti	3/272	185/1867(	0.507296	0.645749	0.557038	F2RL1/CDH	3
GO:19022:	regulation of	3/272	185/1867(	0.507296	0.645749	0.557038	RPS6KA5/J	3
GO:00027(	regulation of	2/272	117/1867(	0.510448	0.648595	0.559492	FADD/EVI2	2
GO:00229(	respiratory ele	2/272	117/1867(	0.510448	0.648595	0.559492	DLD/COX7	2

GO:00313	positive regul	2/272	117/18670	0.510448	0.648595	0.559492	GOLGA2/L	2
GO:00601	regulation of p	2/272	117/18670	0.510448	0.648595	0.559492	POLR2J/RA	2
GO:00609	regulation of g	2/272	117/18670	0.510448	0.648595	0.559492	POLR2J/RA	2
GO:00705	regulation of r	3/272	186/18670	0.510904	0.648931	0.559782	TACC3/CDI	3
GO:00715	response to tr	4/272	255/18670	0.511086	0.648931	0.559782	USP15/THI	4
GO:19038	positive regul	5/272	324/18670	0.511262	0.648931	0.559782	PTGS2/UBI	5
GO:00106	negative regul	6/272	393/18670	0.51146	0.64895	0.559799	PFN2/GMF	6
GO:00024	mast cell med	1/272	49/18670	0.513275	0.649855	0.56058	IL4R	1
GO:00083	glial cell migra	1/272	49/18670	0.513275	0.649855	0.56058	CDK5R1	1
GO:00107	regulation of c	1/272	49/18670	0.513275	0.649855	0.56058	RAP1A	1
GO:00218	forebrain neur	1/272	49/18670	0.513275	0.649855	0.56058	B2M	1
GO:00485	digestive tract	1/272	49/18670	0.513275	0.649855	0.56058	HIF1A	1
GO:19030	regulation of g	1/272	49/18670	0.513275	0.649855	0.56058	GOLGA2	1
GO:00991	microtubule-b	3/272	187/18670	0.514497	0.651169	0.561713	ROPN1L/K	3
GO:00069	phagocytosis,	2/272	118/18670	0.514989	0.651559	0.56205	F2RL1/THE	2
GO:00065	cellular modifi	3/272	188/18670	0.518076	0.655231	0.565217	VNN2/MB	3
GO:00029	response to is	1/272	50/18670	0.520385	0.656041	0.565916	HK2	1
GO:00091	ribonucleoside	1/272	50/18670	0.520385	0.656041	0.565916	NT5C2	1
GO:00311	regulation of r	1/272	50/18670	0.520385	0.656041	0.565916	CDK5R1	1
GO:00328	negative regul	1/272	50/18670	0.520385	0.656041	0.565916	F2RL1	1
GO:00346	nucleobase-cc	1/272	50/18670	0.520385	0.656041	0.565916	UPB1	1
GO:00703	negative regul	1/272	50/18670	0.520385	0.656041	0.565916	F2RL1	1
GO:00705	protein K63-li	1/272	50/18670	0.520385	0.656041	0.565916	TRIM56	1
GO:00707	response to in	1/272	50/18670	0.520385	0.656041	0.565916	STAT1	1
GO:20007	regulation of c	1/272	50/18670	0.520385	0.656041	0.565916	RGS2	1
GO:00182	peptidyl-lysine	6/272	397/18670	0.521342	0.657013	0.566755	RPS6KA5/E	6
GO:00026	negative regul	2/272	120/18670	0.523989	0.659645	0.569025	IL4R/PPM1	2
GO:00170	regulation of t	2/272	120/18670	0.523989	0.659645	0.569025	THBS1/PBI	2
GO:00226	gland morpho	2/272	120/18670	0.523989	0.659645	0.569025	NKX3-1/SU	2
GO:00726	protein localiz	4/272	260/18670	0.526343	0.659938	0.569277	GOLPH3/R	4
GO:00019	positive regul	1/272	51/18670	0.527391	0.659938	0.569277	MAP4K4	1
GO:00027	regulation of I	1/272	51/18670	0.527391	0.659938	0.569277	EXOSC3	1
GO:00028	regulation of i	1/272	51/18670	0.527391	0.659938	0.569277	EXOSC3	1
GO:00090	aspartate fam	1/272	51/18670	0.527391	0.659938	0.569277	DLD	1
GO:00193	arachidonic ac	1/272	51/18670	0.527391	0.659938	0.569277	PTGS2	1
GO:00215	cranial nerve c	1/272	51/18670	0.527391	0.659938	0.569277	CHD7	1
GO:00300	regulation of r	1/272	51/18670	0.527391	0.659938	0.569277	HECW2	1
GO:00320	negative regul	1/272	51/18670	0.527391	0.659938	0.569277	HIF1A	1
GO:00455	negative regul	1/272	51/18670	0.527391	0.659938	0.569277	JDP2	1
GO:00457	negative regul	1/272	51/18670	0.527391	0.659938	0.569277	RGS2	1
GO:00481	positive regul	1/272	51/18670	0.527391	0.659938	0.569277	S100A6	1
GO:00482	positive regul	1/272	51/18670	0.527391	0.659938	0.569277	B2M	1
GO:00509	regulation of r	1/272	51/18670	0.527391	0.659938	0.569277	HIF1A	1
GO:00514	positive regul	1/272	51/18670	0.527391	0.659938	0.569277	PFN2	1
GO:00973	response to b	1/272	51/18670	0.527391	0.659938	0.569277	RGS2	1
GO:20012	negative regul	1/272	51/18670	0.527391	0.659938	0.569277	MMP9	1
GO:00017	formation of p	2/272	121/18670	0.528447	0.660445	0.569715	EOMES/MI	2
GO:00108	positive regul	2/272	121/18670	0.528447	0.660445	0.569715	HSD17B12	2

GO:00467:	viral entry into	2/272	121/1867(	0.528447	0.660445	0.569715	TRIM25/AI	2
GO:00019:	endothelial ce	3/272	191/1867(	0.528729	0.660445	0.569715	THBS1/STA	3
GO:00425!	response to st	3/272	191/1867(	0.528729	0.660445	0.569715	EIF2AK2/EI	3
GO:00516!	establishment	6/272	401/1867(	0.531145	0.663186	0.572079	IL4R/GOLG	6
GO:00061(	purine nucleo	3/272	192/1867(	0.532251	0.663186	0.572079	DLD/ACSL1	3
GO:00442:	sulfur compou	3/272	192/1867(	0.532251	0.663186	0.572079	DLD/ACSL1	3
GO:00507:	positive regul	3/272	192/1867(	0.532251	0.663186	0.572079	PAK2/UNC	3
GO:19038	regulation of (	2/272	122/1867(	0.532876	0.663186	0.572079	THBS1/PBI	2
GO:00031:	heart valve m	1/272	52/18670	0.534295	0.663186	0.572079	MDM4	1
GO:00158:	dopamine tra	1/272	52/18670	0.534295	0.663186	0.572079	ABAT	1
GO:00164	somatic recon	1/272	52/18670	0.534295	0.663186	0.572079	EXOSC3	1
GO:00305:	negative regul	1/272	52/18670	0.534295	0.663186	0.572079	TOB1	1
GO:00315:	ruffle organiz	1/272	52/18670	0.534295	0.663186	0.572079	PFN2	1
GO:00351:	social behavio	1/272	52/18670	0.534295	0.663186	0.572079	CHD8	1
GO:00465	negative regul	1/272	52/18670	0.534295	0.663186	0.572079	RAPGEF1	1
GO:00721:	mesenchyme	1/272	52/18670	0.534295	0.663186	0.572079	MDM4	1
GO:00995:	vesicle cytosk	1/272	52/18670	0.534295	0.663186	0.572079	KIF1B	1
GO:19023:	negative regul	1/272	52/18670	0.534295	0.663186	0.572079	TOB1	1
GO:20001:	positive regul	1/272	52/18670	0.534295	0.663186	0.572079	HIF1A	1
GO:20006	regulation of s	1/272	52/18670	0.534295	0.663186	0.572079	HECW2	1
GO:20007:	regulation of (	1/272	52/18670	0.534295	0.663186	0.572079	B2M	1
GO:00023:	immunoglobu	3/272	193/1867(	0.535758	0.664535	0.573243	IL4R/EXOS	3
GO:00072:	Notch signalin	3/272	193/1867(	0.535758	0.664535	0.573243	STAT1/NFK	3
GO:00453:	cellular respir	3/272	194/1867(	0.53925	0.666704	0.575114	DLD/HIF1A	3
GO:00511(	nuclear expor	3/272	194/1867(	0.53925	0.666704	0.575114	TXN/UPF2/	3
GO:00020	blood vessel e	1/272	53/18670	0.541099	0.666704	0.575114	THBS1	1
GO:00063:	mRNA splice s	1/272	53/18670	0.541099	0.666704	0.575114	PSIP1	1
GO:00100	response to zi	1/272	53/18670	0.541099	0.666704	0.575114	S100A8	1
GO:00108:	negative regul	1/272	53/18670	0.541099	0.666704	0.575114	LRRK2	1
GO:00329(	collagen biosy	1/272	53/18670	0.541099	0.666704	0.575114	RAP1A	1
GO:00350:	sperm-egg rec	1/272	53/18670	0.541099	0.666704	0.575114	FOLR3	1
GO:00350(	regulation of I	1/272	53/18670	0.541099	0.666704	0.575114	RPS6KA5	1
GO:00362!	interstrand cr	1/272	53/18670	0.541099	0.666704	0.575114	MCM8	1
GO:00456(	regulation of r	1/272	53/18670	0.541099	0.666704	0.575114	IL18	1
GO:00507(	regulation of i	1/272	53/18670	0.541099	0.666704	0.575114	ORM2	1
GO:00507:	negative regul	1/272	53/18670	0.541099	0.666704	0.575114	SH3BP5L	1
GO:00605(	neuroepitheli	1/272	53/18670	0.541099	0.666704	0.575114	CDH2	1
GO:00985	cellular respo	1/272	53/18670	0.541099	0.666704	0.575114	IFI6	1
GO:19020!	regulation of r	1/272	53/18670	0.541099	0.666704	0.575114	HECW2	1
GO:00346(	ncRNA metab	7/272	475/1867(	0.541123	0.666704	0.575114	RPS28/RPS	7
GO:00000	transition me	2/272	124/1867(	0.54165	0.666704	0.575114	LMTK2/B2	2
GO:00140:	regulation of g	2/272	124/1867(	0.54165	0.666704	0.575114	TLR2/CDH:	2
GO:00197!	polyol metabo	2/272	124/1867(	0.54165	0.666704	0.575114	ASAH1/SYI	2
GO:00467:	acid secretion	2/272	124/1867(	0.54165	0.666704	0.575114	RAB3GAP1	2
GO:00482	organelle fusi	2/272	124/1867(	0.54165	0.666704	0.575114	STX10/TAF	2
GO:00303:	embryonic lim	2/272	125/1867(	0.545994	0.671477	0.579231	PBX2/CHD	2
GO:00351:	embryonic ap	2/272	125/1867(	0.545994	0.671477	0.579231	PBX2/CHD	2
GO:00065	catecholamin	1/272	54/18670	0.547804	0.671477	0.579231	ABAT	1

GO:00097:	catechol-conta	1/272	54/18670	0.547804	0.671477	0.579231	ABAT	1
GO:00326(	regulation of i	1/272	54/18670	0.547804	0.671477	0.579231	CLEC7A	1
GO:00433!	negative regul	1/272	54/18670	0.547804	0.671477	0.579231	NFKBIA	1
GO:00461(	alcohol catabo	1/272	54/18670	0.547804	0.671477	0.579231	SYNJ2	1
GO:00509!	regulation of l	1/272	54/18670	0.547804	0.671477	0.579231	IRS2	1
GO:00513!	positive regula	1/272	54/18670	0.547804	0.671477	0.579231	HIF1A	1
GO:00517(	intraspecies ir	1/272	54/18670	0.547804	0.671477	0.579231	CHD8	1
GO:00702(	protein trimer	1/272	54/18670	0.547804	0.671477	0.579231	ALOX5AP	1
GO:19046‹	response to ai	1/272	54/18670	0.547804	0.671477	0.579231	MMP9	1
GO:00316(	cellular respoi	4/272	268/1867(	0.550266	0.673848	0.581276	PTGS2/EIF:	4
GO:00456(	regulation of (	2/272	126/1867(	0.550309	0.673848	0.581276	TOB1/HDA	2
GO:00901(	negative regul	2/272	126/1867(	0.550309	0.673848	0.581276	TOB1/PBLI	2
GO:00076:	locomotory be	3/272	198/1867(	0.553068	0.676056	0.583181	LRRK2/CHI	3
GO:00605‹	respiratory sy:	3/272	198/1867(	0.553068	0.676056	0.583181	MME/CHD	3
GO:00032:	ventricular cai	1/272	55/18670	0.554411	0.676056	0.583181	CHD7	1
GO:00061:	mitochondrial	1/272	55/18670	0.554411	0.676056	0.583181	DLD	1
GO:00063{	transcription l	1/272	55/18670	0.554411	0.676056	0.583181	CHD8	1
GO:00327{	positive regula	1/272	55/18670	0.554411	0.676056	0.583181	PFN2	1
GO:00423(	regulation of f	1/272	55/18670	0.554411	0.676056	0.583181	PTGS2	1
GO:00423(	regulation of p	1/272	55/18670	0.554411	0.676056	0.583181	PTGS2	1
GO:00431:	receptor clust	1/272	55/18670	0.554411	0.676056	0.583181	CDH2	1
GO:00439!	cellular compo	1/272	55/18670	0.554411	0.676056	0.583181	F2RL1	1
GO:00456:	negative regul	1/272	55/18670	0.554411	0.676056	0.583181	IL4R	1
GO:00714:	ribonucleopro	2/272	127/1867(	0.554595	0.676056	0.583181	UPF2/RAE:	2
GO:00990:	plasma memb	2/272	127/1867(	0.554595	0.676056	0.583181	F2RL1/THE	2
GO:00310:	actomyosin st	3/272	199/1867(	0.556484	0.677892	0.584765	PFN2/PHA	3
GO:00508(	negative regul	3/272	199/1867(	0.556484	0.677892	0.584765	MNDA/IL4	3
GO:00711(	ribonucleopro	2/272	128/1867(	0.558853	0.680344	0.58688	UPF2/RAE:	2
GO:00347(	regulation of i	7/272	483/1867(	0.558881	0.680344	0.58688	CACNA1E/'	7
GO:00330‹	regulation of (	5/272	342/1867(	0.559297	0.680615	0.587114	RPS6KA5/T	5
GO:00023{	immunoglobu	1/272	56/18670	0.560922	0.681188	0.587608	EXOSC3	1
GO:00103:	response to ga	1/272	56/18670	0.560922	0.681188	0.587608	RPL26	1
GO:00452!	mRNA cis splic	1/272	56/18670	0.560922	0.681188	0.587608	PSIP1	1
GO:00515(	histone H3-K4	1/272	56/18670	0.560922	0.681188	0.587608	ARID4A	1
GO:00606{	regulation of r	1/272	56/18670	0.560922	0.681188	0.587608	LRRK2	1
GO:00903(	mitotic spindle	1/272	56/18670	0.560922	0.681188	0.587608	GOLGA2	1
GO:00328(	response to in	4/272	272/1867(	0.56199	0.682251	0.588525	STAT1/IRS:	4
GO:00032(	cardiac chamb	2/272	129/1867(	0.563081	0.682873	0.589062	HIF1A/CHD	2
GO:00347!	cellular hormo	2/272	129/1867(	0.563081	0.682873	0.589062	HSD17B11,	2
GO:00508!	B cell receptoi	2/272	129/1867(	0.563081	0.682873	0.589062	MNDA/BM	2
GO:00435(	muscle adapta	2/272	130/1867(	0.567281	0.68639	0.592095	RGS2/HDA	2
GO:00017!	neural crest ce	1/272	57/18670	0.567338	0.68639	0.592095	HIF1A	1
GO:00020(	columnar/cub	1/272	57/18670	0.567338	0.68639	0.592095	HIF1A	1
GO:00148{	striated muscl	1/272	57/18670	0.567338	0.68639	0.592095	HDAC4	1
GO:00333‹	cholesterol ef	1/272	57/18670	0.567338	0.68639	0.592095	NFKBIA	1
GO:00480(	platelet-derive	1/272	57/18670	0.567338	0.68639	0.592095	RAPGEF1	1
GO:00757:	intracellular tr	1/272	57/18670	0.567338	0.68639	0.592095	RAE1	1
GO:00435‹	endothelial ce	4/272	274/1867(	0.567789	0.686701	0.592364	PTGS2/THI	4

GO:00486:	regulation of (	5/272	347/1867(	0.572223	0.691827	0.596785	IFRD1/TNF	5
GO:19016(	alpha-amino a	3/272	204/1867(	0.573329	0.691911	0.596858	DLD/HAL/F	3
GO:00192:	regulation of v	1/272	58/18670	0.573661	0.691911	0.596858	PTGS2	1
GO:00353(	positive regul:	1/272	58/18670	0.573661	0.691911	0.596858	PPP1R12A	1
GO:00510!	negative regul	1/272	58/18670	0.573661	0.691911	0.596858	RAPGEF1	1
GO:00619!	establishment	1/272	58/18670	0.573661	0.691911	0.596858	GOLPH3	1
GO:19037	positive regul:	1/272	58/18670	0.573661	0.691911	0.596858	UBE2D3	1
GO:19045	regulation of p	1/272	58/18670	0.573661	0.691911	0.596858	PTGS2	1
GO:00421	negative regul	2/272	132/1867(	0.575592	0.693768	0.59846	MDM4/RP	2
GO:00455	regulation of f	2/272	132/1867(	0.575592	0.693768	0.59846	PTGS2/JDF	2
GO:00725:	purine-contair	3/272	205/1867(	0.57665	0.694806	0.599356	DLD/ACSL1	3
GO:00095	detection of e	2/272	133/1867(	0.579704	0.696341	0.60068	RGR/UNC1	2
GO:00218	forebrain gen	1/272	59/18670	0.579891	0.696341	0.60068	B2M	1
GO:00305:	androgen rece	1/272	59/18670	0.579891	0.696341	0.60068	NKX3-1	1
GO:00311(	neuron projec	1/272	59/18670	0.579891	0.696341	0.60068	MAP4K4	1
GO:00458	negative regul	1/272	59/18670	0.579891	0.696341	0.60068	RGS2	1
GO:00705:	platelet aggre	1/272	59/18670	0.579891	0.696341	0.60068	ABAT	1
GO:19023(	negative regul	1/272	59/18670	0.579891	0.696341	0.60068	TOB1	1
GO:20002	negative regul	1/272	59/18670	0.579891	0.696341	0.60068	NKX3-1	1
GO:20003!	regulation of e	1/272	59/18670	0.579891	0.696341	0.60068	THBS1	1
GO:20007!	regulation of p	1/272	59/18670	0.579891	0.696341	0.60068	RPS6KA5	1
GO:00067(	vitamin metat	2/272	134/1867(	0.583787	0.70007	0.603896	LMBRD1/V	2
GO:00070(	mitochondrial	2/272	134/1867(	0.583787	0.70007	0.603896	HK2/HEBP:	2
GO:00444(	entry into hos	2/272	134/1867(	0.583787	0.70007	0.603896	TRIM25/AI	2
GO:00485(	digestive tract	2/272	134/1867(	0.583787	0.70007	0.603896	HIF1A/CHD	2
GO:00024	lymphocyte m	5/272	352/1867(	0.584951	0.701098	0.604783	IL4R/EXOS	5
GO:00330	tetrapyrrole m	1/272	60/18670	0.586031	0.701098	0.604783	LMBRD1	1
GO:00422(	natural killer c	1/272	60/18670	0.586031	0.701098	0.604783	IL18	1
GO:00467!	transport of vi	1/272	60/18670	0.586031	0.701098	0.604783	RAE1	1
GO:00486:	negative regul	1/272	60/18670	0.586031	0.701098	0.604783	RGS2	1
GO:00518!	modulation by	1/272	60/18670	0.586031	0.701098	0.604783	EIF2AK4	1
GO:00903	regulation of (	1/272	60/18670	0.586031	0.701098	0.604783	B2M	1
GO:00315(	protein-contai	4/272	281/1867(	0.587749	0.702314	0.605832	UPF2/RAE:	4
GO:00064(	RNA export fr	2/272	135/1867(	0.587841	0.702314	0.605832	UPF2/RAE:	2
GO:00074!	mesoderm de	2/272	135/1867(	0.587841	0.702314	0.605832	EOMES/BN	2
GO:00103:	membrane inv	2/272	135/1867(	0.587841	0.702314	0.605832	F2RL1/THE	2
GO:00095	detection of a	2/272	136/1867(	0.591866	0.706189	0.609175	RGR/UNC1	2
GO:00019	heart looping	1/272	61/18670	0.592082	0.706189	0.609175	HIF1A	1
GO:00031	heart valve de	1/272	61/18670	0.592082	0.706189	0.609175	MDM4	1
GO:00322:	positive regul:	1/272	61/18670	0.592082	0.706189	0.609175	PFN2	1
GO:00465	ceramide bios	1/272	61/18670	0.592082	0.706189	0.609175	ASAH1	1
GO:00316	regulation of r	2/272	137/1867(	0.595861	0.708763	0.611395	LRRK2/AB/	2
GO:00464:	regulation of r	2/272	137/1867(	0.595861	0.708763	0.611395	CDK5R1/IL	2
GO:19036	regulation of s	2/272	137/1867(	0.595861	0.708763	0.611395	PTGS2/THI	2
GO:00025(	somatic divers	1/272	62/18670	0.598044	0.708763	0.611395	EXOSC3	1
GO:00027!	cytoplasmic p:	1/272	62/18670	0.598044	0.708763	0.611395	NFKBIA	1
GO:00075	respiratory ga	1/272	62/18670	0.598044	0.708763	0.611395	CYSLTR1	1
GO:00095	detection of v	1/272	62/18670	0.598044	0.708763	0.611395	RGR	1

GO:00164	somatic cell D	1/272	62/18670	0.598044	0.708763	0.611395	EXOSC3	1
GO:00323	regulation of c	1/272	62/18670	0.598044	0.708763	0.611395	NFKBIA	1
GO:00328	glomerulus de	1/272	62/18670	0.598044	0.708763	0.611395	SULF2	1
GO:00329	circadian regu	1/272	62/18670	0.598044	0.708763	0.611395	AHR	1
GO:00468	positive regula	1/272	62/18670	0.598044	0.708763	0.611395	PTGS2	1
GO:00488	neural nucleus	1/272	62/18670	0.598044	0.708763	0.611395	CDK5R1	1
GO:00504	regulation of c	1/272	62/18670	0.598044	0.708763	0.611395	ABAT	1
GO:00518	negative regul	1/272	62/18670	0.598044	0.708763	0.611395	RAPGEF1	1
GO:00603	regulation of p	1/272	62/18670	0.598044	0.708763	0.611395	PBLD	1
GO:00700	intrinsic apopt	1/272	62/18670	0.598044	0.708763	0.611395	LRRK2	1
GO:19018	negative regul	1/272	62/18670	0.598044	0.708763	0.611395	RGS2	1
GO:19023	regulation of s	1/272	62/18670	0.598044	0.708763	0.611395	HECW2	1
GO:00068	endoplasmic r	3/272	212/1867C	0.599442	0.710013	0.612473	GOLGA2/N	3
GO:00066	phospholipid r	6/272	430/1867C	0.5995	0.710013	0.612473	ACP6/SYNJ	6
GO:00085	male gonad de	2/272	138/1867C	0.599827	0.710163	0.612602	NKX3-1/AF	2
GO:00455	regulation of T	2/272	139/1867C	0.603763	0.712863	0.614931	IL4R/IL18	2
GO:00465	development	2/272	139/1867C	0.603763	0.712863	0.614931	NKX3-1/AF	2
GO:00609	regulation of g	2/272	139/1867C	0.603763	0.712863	0.614931	POLR2J/RA	2
GO:00068	cellular iron io	1/272	63/18670	0.603919	0.712863	0.614931	HIF1A	1
GO:00316	positive regula	1/272	63/18670	0.603919	0.712863	0.614931	ABAT	1
GO:00323	regulation of s	1/272	63/18670	0.603919	0.712863	0.614931	NFKBIA	1
GO:00343	protein localiz	1/272	63/18670	0.603919	0.712863	0.614931	PICALM	1
GO:00468	negative regul	1/272	63/18670	0.603919	0.712863	0.614931	MAP4K4	1
GO:20004	regulation of l	1/272	63/18670	0.603919	0.712863	0.614931	FADD	1
GO:00059	monosacchari	4/272	287/1867C	0.604426	0.713223	0.615242	PFKFB2/HK	4
GO:20010	regulation of r	3/272	214/1867C	0.605805	0.714612	0.61644	NKX3-1/RF	3
GO:00022	natural killer c	1/272	64/18670	0.609709	0.716807	0.618334	IL18	1
GO:00164	somatic divers	1/272	64/18670	0.609709	0.716807	0.618334	EXOSC3	1
GO:00357	positive regula	1/272	64/18670	0.609709	0.716807	0.618334	HEBP2	1
GO:00456	positive regula	1/272	64/18670	0.609709	0.716807	0.618334	PTGS2	1
GO:00467	regulation of v	1/272	64/18670	0.609709	0.716807	0.618334	POLR2J	1
GO:00482	lymphocyte ch	1/272	64/18670	0.609709	0.716807	0.618334	CKLF	1
GO:00482	vesicle dockin	1/272	64/18670	0.609709	0.716807	0.618334	STX10	1
GO:00504	catecholamine	1/272	64/18670	0.609709	0.716807	0.618334	ABAT	1
GO:00509	negative regul	1/272	64/18670	0.609709	0.716807	0.618334	THBS1	1
GO:19036	positive regula	1/272	64/18670	0.609709	0.716807	0.618334	PTGS2	1
GO:00071	negative regul	4/272	289/1867C	0.609894	0.716807	0.618334	THBS1/IL4	4
GO:00082	insulin recept	2/272	141/1867C	0.611549	0.718514	0.619807	IRS2/OSBP	2
GO:00328	cellular respor	3/272	216/1867C	0.612101	0.718925	0.620161	STAT1/IRS	3
GO:00434	response to p	6/272	436/1867C	0.612969	0.719475	0.620635	PTGS2/PRK	6
GO:00711	DNA conforma	5/272	364/1867C	0.614648	0.719475	0.620635	CHD7/MCN	5
GO:00436	cellular protei	3/272	217/1867C	0.615224	0.719475	0.620635	F2RL1/LMC	3
GO:00442	cellular lipid c	3/272	217/1867C	0.615224	0.719475	0.620635	ASAH1/CYP	3
GO:00082	regulation of C	2/272	142/1867C	0.615398	0.719475	0.620635	RGS2/RGS	2
GO:00464	membrane lip	2/272	142/1867C	0.615398	0.719475	0.620635	ASAH1/MF	2
GO:00720	nephron deve	2/272	142/1867C	0.615398	0.719475	0.620635	SULF2/STA	2
GO:00025	monocyte che	1/272	65/18670	0.615415	0.719475	0.620635	S100A12	1
GO:00092	glycolipid bios	1/272	65/18670	0.615415	0.719475	0.620635	MPPE1	1

GO:00148:	response to a	1/272	65/18670	0.615415	0.719475	0.620635	HIF1A	1
GO:00327(	regulation of r	1/272	65/18670	0.615415	0.719475	0.620635	HIF1A	1
GO:00457:	positive regul	1/272	65/18670	0.615415	0.719475	0.620635	DTX3L	1
GO:00725:	endothelial ce	1/272	65/18670	0.615415	0.719475	0.620635	THBS1	1
GO:00726:	T cell migratio	1/272	65/18670	0.615415	0.719475	0.620635	FADD	1
GO:00068:	sodium ion tra	3/272	218/1867(	0.61833	0.722405	0.623163	SLC5A9/HE	3
GO:00328(	regulation of r	3/272	218/1867(	0.61833	0.722405	0.623163	TACC3/CDI	3
GO:00066(	glycosphingoli	1/272	66/18670	0.621038	0.723182	0.623833	ASAH1	1
GO:00171!	calcium-ion re	1/272	66/18670	0.621038	0.723182	0.623833	RAB3GAP1	1
GO:00400:	regulation of r	1/272	66/18670	0.621038	0.723182	0.623833	CHD7	1
GO:00466:	regulation of i	1/272	66/18670	0.621038	0.723182	0.623833	OSBPL8	1
GO:00482(	vesicle targeti	1/272	66/18670	0.621038	0.723182	0.623833	GOLGA2	1
GO:00482(	COPII vesicle c	1/272	66/18670	0.621038	0.723182	0.623833	GOLGA2	1
GO:00519:	negative regul	1/272	66/18670	0.621038	0.723182	0.623833	PTGS2	1
GO:00990:	regulation of p	1/272	66/18670	0.621038	0.723182	0.623833	RAP1A	1
GO:19052(	regulation of c	1/272	66/18670	0.621038	0.723182	0.623833	RGS2	1
GO:19057:	positive regul	1/272	66/18670	0.621038	0.723182	0.623833	HEBP2	1
GO:00481!	Golgi vesicle t	5/272	368/1867(	0.624269	0.726706	0.626873	GOLPH3/E	5
GO:00031·	embryonic he	1/272	67/18670	0.626578	0.728181	0.628145	HIF1A	1
GO:00439(	histone H4 ac	1/272	67/18670	0.626578	0.728181	0.628145	EPC1	1
GO:00613:	determinatior	1/272	67/18670	0.626578	0.728181	0.628145	HIF1A	1
GO:00061:	oxidative pho	2/272	145/1867(	0.62677	0.728181	0.628145	DLD/COX7	2
GO:00076(	sensory perce	2/272	145/1867(	0.62677	0.728181	0.628145	ZNF354A/(	2
GO:00719(	negative regul	2/272	145/1867(	0.62677	0.728181	0.628145	PRKAR2A/I	2
GO:00458:	positive regul	2/272	146/1867(	0.630503	0.73142	0.63094	PTGS2/IRS	2
GO:00512!	negative regul	2/272	146/1867(	0.630503	0.73142	0.63094	MNDA/IL4	2
GO:00551:	digestive syst	2/272	146/1867(	0.630503	0.73142	0.63094	HIF1A/CHD	2
GO:19048!	regulation of r	2/272	146/1867(	0.630503	0.73142	0.63094	CDK5R1/IL	2
GO:19035:	regulation of l	4/272	297/1867(	0.631298	0.73142	0.63094	F2RL1/PTG	4
GO:00066:	protein target	1/272	68/18670	0.632038	0.73142	0.63094	UBE2D3	1
GO:00336:	cell adhesion i	1/272	68/18670	0.632038	0.73142	0.63094	PLAU	1
GO:00508!	multicellular c	1/272	68/18670	0.632038	0.73142	0.63094	PRKAR2A	1
GO:00509:	positive chem	1/272	68/18670	0.632038	0.73142	0.63094	F2RL1	1
GO:00519(	positive regul	1/272	68/18670	0.632038	0.73142	0.63094	LRRN3	1
GO:00726(	protein localiz	1/272	68/18670	0.632038	0.73142	0.63094	UBE2D3	1
GO:00726(	establishment	1/272	68/18670	0.632038	0.73142	0.63094	UBE2D3	1
GO:00102:	response to ic	2/272	147/1867(	0.634206	0.733689	0.632897	GADD45A/	2
GO:00017(	in utero embr	5/272	373/1867(	0.636092	0.735631	0.634572	EOMES/HI	5
GO:00550(	cardiac muscl	1/272	69/18670	0.637418	0.736924	0.635687	CHD7	1
GO:00351(	appendage m	2/272	148/1867(	0.63788	0.736977	0.635733	PBX2/CHD	2
GO:00351(	limb morphog	2/272	148/1867(	0.63788	0.736977	0.635733	PBX2/CHD	2
GO:00600·	retina develop	2/272	149/1867(	0.641526	0.740635	0.638888	HIF1A/CHD	2
GO:20000!	regulation of u	2/272	149/1867(	0.641526	0.740635	0.638888	RPL23/LRR	2
GO:00017!	somitogenesis	1/272	70/18670	0.64272	0.740635	0.638888	NKX3-1	1
GO:00033·	cilium movem	1/272	70/18670	0.64272	0.740635	0.638888	ROPN1L	1
GO:00069(	vesicle coatin	1/272	70/18670	0.64272	0.740635	0.638888	GOLGA2	1
GO:00105:	regulation of p	1/272	70/18670	0.64272	0.740635	0.638888	RGS2	1
GO:00435:	peroxisomal t	1/272	70/18670	0.64272	0.740635	0.638888	UBE2D3	1

GO:00709	demethylation	1/272	70/18670	0.64272	0.740635	0.638888	UTY	1
GO:00507	negative regul	2/272	150/18670	0.645142	0.743184	0.641087	IL4R/PSMA	2
GO:00422	ribosomal larg	1/272	71/18670	0.647945	0.745442	0.643035	RPL26	1
GO:00425	positive regula	1/272	71/18670	0.647945	0.745442	0.643035	IL18	1
GO:00436	inositol phosp	1/272	71/18670	0.647945	0.745442	0.643035	SYNJ2	1
GO:00439	regulation of r	1/272	71/18670	0.647945	0.745442	0.643035	HDAC4	1
GO:00000	mitotic sister c	2/272	151/18670	0.64873	0.745861	0.643396	TACC3/HE	2
GO:00550	lipid homeosta	2/272	151/18670	0.64873	0.745861	0.643396	IRS2/IL18	2
GO:00018	tissue homeos	3/272	229/18670	0.65137	0.747962	0.645209	PTGS2/PBL	3
GO:00105	regulation of e	3/272	229/18670	0.65137	0.747962	0.645209	PTGS2/TH	3
GO:00309	hindbrain dev	2/272	152/18670	0.652289	0.747962	0.645209	CDK5R1/A	2
GO:00510	mRNA transpo	2/272	152/18670	0.652289	0.747962	0.645209	UPF2/RAE	2
GO:00063	DNA alkylation	1/272	72/18670	0.653094	0.747962	0.645209	TDRD1	1
GO:00063	DNA methylat	1/272	72/18670	0.653094	0.747962	0.645209	TDRD1	1
GO:00066	fatty acid beta	1/272	72/18670	0.653094	0.747962	0.645209	IRS2	1
GO:00091	nucleoside mo	1/272	72/18670	0.653094	0.747962	0.645209	NT5C2	1
GO:00350	somatic stem	1/272	72/18670	0.653094	0.747962	0.645209	POLR2J	1
GO:00611	mammary glan	1/272	72/18670	0.653094	0.747962	0.645209	HIF1A	1
GO:00901	COPII-coated v	1/272	72/18670	0.653094	0.747962	0.645209	GOLGA2	1
GO:19030	negative regul	1/272	72/18670	0.653094	0.747962	0.645209	RPL23	1
GO:00064	RNA localizatio	3/272	230/18670	0.654271	0.749068	0.646163	EXOSC3/U	3
GO:00023	B cell activatio	1/272	73/18670	0.658167	0.751342	0.648125	EXOSC3	1
GO:00062	transcription-c	1/272	73/18670	0.658167	0.751342	0.648125	POLR2J	1
GO:00099	cell-cell recog	1/272	73/18670	0.658167	0.751342	0.648125	FOLR3	1
GO:00302	myofibril asse	1/272	73/18670	0.658167	0.751342	0.648125	LMOD3	1
GO:00329	negative regul	1/272	73/18670	0.658167	0.751342	0.648125	MNDA	1
GO:00335	multicellular o	1/272	73/18670	0.658167	0.751342	0.648125	THBS1	1
GO:00456	regulation of g	1/272	73/18670	0.658167	0.751342	0.648125	TLR2	1
GO:00506	negative regul	1/272	73/18670	0.658167	0.751342	0.648125	MNDA	1
GO:19019	regulation of p	1/272	73/18670	0.658167	0.751342	0.648125	RPS6KA5	1
GO:19037	negative regul	2/272	155/18670	0.662795	0.754374	0.65074	IL4R/NFKB	2
GO:00018	retina homeos	1/272	74/18670	0.663167	0.754374	0.65074	B2M	1
GO:00140	positive regula	1/272	74/18670	0.663167	0.754374	0.65074	TLR2	1
GO:00301	water homeos	1/272	74/18670	0.663167	0.754374	0.65074	PRKAR2A	1
GO:00316	cellular respo	1/272	74/18670	0.663167	0.754374	0.65074	PTGS2	1
GO:00435	positive regula	1/272	74/18670	0.663167	0.754374	0.65074	MAP3K2	1
GO:00481	vesicle targeti	1/272	74/18670	0.663167	0.754374	0.65074	GOLGA2	1
GO:00511	smooth muscl	1/272	74/18670	0.663167	0.754374	0.65074	EPC1	1
GO:00703	negative regul	1/272	74/18670	0.663167	0.754374	0.65074	RAPGEF1	1
GO:19000	regulation of c	1/272	74/18670	0.663167	0.754374	0.65074	OSBPL8	1
GO:19036	negative regul	1/272	74/18670	0.663167	0.754374	0.65074	THBS1	1
GO:19016	cellular respo	5/272	385/18670	0.663525	0.754539	0.650882	PRKAR2A/S	5
GO:00165	histone acetyl	2/272	156/18670	0.66624	0.757139	0.653125	RPS6KA5/E	2
GO:00308	regulation of e	2/272	156/18670	0.66624	0.757139	0.653125	STAT1/MM	2
GO:00017	mesoderm for	1/272	75/18670	0.668094	0.757544	0.653475	EOMES	1
GO:00022	somatic divers	1/272	75/18670	0.668094	0.757544	0.653475	EXOSC3	1
GO:00032	ventricular sep	1/272	75/18670	0.668094	0.757544	0.653475	MDM4	1
GO:00106	regulation of c	1/272	75/18670	0.668094	0.757544	0.653475	RGS2	1

GO:00215:	diencephalon	1/272	75/18670	0.668094	0.757544	0.653475	RAB3GAP1	1
GO:00461:	polyol biosynt	1/272	75/18670	0.668094	0.757544	0.653475	ASAH1	1
GO:19021:	positive regula	1/272	75/18670	0.668094	0.757544	0.653475	RAB3GAP1	1
GO:00423:	vasoconstricti	1/272	76/18670	0.672949	0.761937	0.657264	PTGS2	1
GO:00488	artery morpho	1/272	76/18670	0.672949	0.761937	0.657264	CHD7	1
GO:00604:	cardiac septur	1/272	76/18670	0.672949	0.761937	0.657264	CHD7	1
GO:00720	nephron epith	1/272	76/18670	0.672949	0.761937	0.657264	STAT1	1
GO:00521:	movement in	2/272	158/1867(	0.673045	0.761937	0.657264	TRIM25/AI	2
GO:00068!	receptor-med	4/272	314/1867(	0.674227	0.763031	0.658208	UNC119/C	4
GO:00083	adult locomot	1/272	77/18670	0.677733	0.765531	0.660365	CHD7	1
GO:00092(	purine ribonu	1/272	77/18670	0.677733	0.765531	0.660365	LRRK2	1
GO:00350!	embryonic he	1/272	77/18670	0.677733	0.765531	0.660365	HIF1A	1
GO:00483:	mesoderm mo	1/272	77/18670	0.677733	0.765531	0.660365	EOMES	1
GO:00519:	catecholamine	1/272	77/18670	0.677733	0.765531	0.660365	ABAT	1
GO:20007:	regulation of c	1/272	77/18670	0.677733	0.765531	0.660365	DTX3L	1
GO:00466(	male sex diffe	2/272	160/1867(	0.679737	0.767305	0.661895	NKX3-1/AF	2
GO:20002	regulation of r	2/272	160/1867(	0.679737	0.767305	0.661895	NKX3-1/HI	2
GO:00147	regulation of r	1/272	78/18670	0.682448	0.76816	0.662633	RGS2	1
GO:00304:	ubiquitin-depe	1/272	78/18670	0.682448	0.76816	0.662633	TRIM25	1
GO:00434(	negative regul	1/272	78/18670	0.682448	0.76816	0.662633	RGS2	1
GO:00550:	regulation of c	1/272	78/18670	0.682448	0.76816	0.662633	RGS2	1
GO:00706(	negative regul	1/272	78/18670	0.682448	0.76816	0.662633	MNDA	1
GO:00720:	nephron morp	1/272	78/18670	0.682448	0.76816	0.662633	STAT1	1
GO:00723:	intrinsic apopt	1/272	78/18670	0.682448	0.76816	0.662633	RPL26	1
GO:00903!	cellular senesc	1/272	78/18670	0.682448	0.76816	0.662633	B2M	1
GO:20002	positive regula	1/272	78/18670	0.682448	0.76816	0.662633	HDAC4	1
GO:00109:	transport alon	2/272	161/1867(	0.683041	0.76834	0.662787	KIF1B/HIF1	2
GO:00183!	internal pepti	2/272	161/1867(	0.683041	0.76834	0.662787	RPS6KA5/E	2
GO:00900!	regulation of t	3/272	241/1867(	0.685039	0.770342	0.664515	TOB1/THB	3
GO:00072(	positive regula	4/272	319/1867(	0.686177	0.770938	0.665029	F2RL1/CYS	4
GO:00015:	vasculogenesi	1/272	79/18670	0.687093	0.770938	0.665029	RAP1A	1
GO:00031!	outflow tract	1/272	79/18670	0.687093	0.770938	0.665029	HIF1A	1
GO:00311:	regulation of r	1/272	79/18670	0.687093	0.770938	0.665029	CDK5R1	1
GO:00435:	negative regul	1/272	79/18670	0.687093	0.770938	0.665029	THBS1	1
GO:19000:	regulation of c	1/272	79/18670	0.687093	0.770938	0.665029	RAE1	1
GO:19033:	negative regul	1/272	79/18670	0.687093	0.770938	0.665029	TOB1	1
GO:00713:	cellular respor	4/272	321/1867(	0.69087	0.774931	0.668473	PRKAR2A/!	4
GO:00705(	calcium ion im	1/272	80/18670	0.691671	0.775369	0.668851	CACNA1E	1
GO:00015(	ossification	5/272	398/1867(	0.691699	0.775369	0.668851	TOB1/PTG:	5
GO:00070	regulation of r	2/272	164/1867(	0.692787	0.776342	0.66969	TACC3/HE(	2
GO:00329	protein-contai	4/272	323/1867(	0.695514	0.778177	0.671273	F2RL1/RPL	4
GO:00509!	sensory perce	2/272	165/1867(	0.69598	0.778177	0.671273	ZNF354A/(	2
GO:00070:	peroxisome o	1/272	81/18670	0.696183	0.778177	0.671273	UBE2D3	1
GO:00140:	neural crest c	1/272	81/18670	0.696183	0.778177	0.671273	HIF1A	1
GO:00217(	hippocampus	1/272	81/18670	0.696183	0.778177	0.671273	CDK5R1	1
GO:00305:	negative regul	1/272	81/18670	0.696183	0.778177	0.671273	PBLD	1
GO:00324:	positive regula	1/272	81/18670	0.696183	0.778177	0.671273	LRRK2	1
GO:00341(	homotypic cel	1/272	81/18670	0.696183	0.778177	0.671273	ABAT	1

GO:00064:	internal prote	2/272	166/18670	0.699146	0.781243	0.673918	RPS6KA5/E	2
GO:00512:	regulation of r	1/272	82/18670	0.700628	0.782405	0.67492	CHD7	1
GO:00604:	muscle tissue	1/272	82/18670	0.700628	0.782405	0.67492	CHD7	1
GO:00350!	cardiocyte diff	2/272	167/18670	0.702285	0.784008	0.676303	RGS2/EOM	2
GO:00091!	ribonucleoside	1/272	83/18670	0.705009	0.785253	0.677377	LRRK2	1
GO:00345(	protein localiz	1/272	83/18670	0.705009	0.785253	0.677377	MCM8	1
GO:00425(	regulation of t	1/272	83/18670	0.705009	0.785253	0.677377	IL18	1
GO:00464:	phosphatidylc	1/272	83/18670	0.705009	0.785253	0.677377	MBOAT1	1
GO:00481:	regulation of f	1/272	83/18670	0.705009	0.785253	0.677377	S100A6	1
GO:00712:	cellular respor	1/272	83/18670	0.705009	0.785253	0.677377	ALOX5AP	1
GO:19038:	negative regul	1/272	83/18670	0.705009	0.785253	0.677377	PBLD	1
GO:00071!	homophilic ce	2/272	168/18670	0.705396	0.785253	0.677377	CDH2/NPT	2
GO:00513(	regulation of c	2/272	168/18670	0.705396	0.785253	0.677377	NKX3-1/EV	2
GO:00156!	inorganic anio	2/272	169/18670	0.708481	0.786905	0.678802	CLCC1/SLC	2
GO:00183!	peptidyl-lysine	2/272	169/18670	0.708481	0.786905	0.678802	RPS6KA5/E	2
GO:00063(	transcription e	1/272	84/18670	0.709326	0.786905	0.678802	POLR2J	1
GO:00069:	phagocytosis,	1/272	84/18670	0.709326	0.786905	0.678802	CLEC7A	1
GO:00468!	positive regula	1/272	84/18670	0.709326	0.786905	0.678802	PTGS2	1
GO:00481:	fibroblast prol	1/272	84/18670	0.709326	0.786905	0.678802	S100A6	1
GO:00514!	regulation of i	1/272	84/18670	0.709326	0.786905	0.678802	LRRK2	1
GO:00514!	regulation of s	1/272	84/18670	0.709326	0.786905	0.678802	PFN2	1
GO:00604:	regulation of h	1/272	84/18670	0.709326	0.786905	0.678802	RGS2	1
GO:00977!	negative regul	1/272	84/18670	0.709326	0.786905	0.678802	PTGS2	1
GO:19058!	regulation of r	1/272	84/18670	0.709326	0.786905	0.678802	LRRK2	1
GO:00020:	regulation of s	1/272	85/18670	0.71358	0.790151	0.681602	HECW2	1
GO:00091:	purine nucleo:	1/272	85/18670	0.71358	0.790151	0.681602	LRRK2	1
GO:00140:	mesenchymal	1/272	85/18670	0.71358	0.790151	0.681602	HIF1A	1
GO:00488(	stem cell deve	1/272	85/18670	0.71358	0.790151	0.681602	HIF1A	1
GO:00507:	positive regula	1/272	85/18670	0.71358	0.790151	0.681602	TNFRSF12A	1
GO:00082(	steroid metab	4/272	331/18670	0.713591	0.790151	0.681602	HSD17B11	4
GO:00303:	lung developn	2/272	172/18670	0.717573	0.793033	0.684088	MME/CRIS	2
GO:00019:	hair follicle de	1/272	86/18670	0.717772	0.793033	0.684088	TGM3	1
GO:00063(	double-strand	1/272	86/18670	0.717772	0.793033	0.684088	DTX3L	1
GO:00068!	retrograde ve:	1/272	86/18670	0.717772	0.793033	0.684088	GOLPH3	1
GO:00072(	tyrosine phos	1/272	86/18670	0.717772	0.793033	0.684088	IL18	1
GO:00301(	natural killer c	1/272	86/18670	0.717772	0.793033	0.684088	IL18	1
GO:19033(	negative regul	1/272	86/18670	0.717772	0.793033	0.684088	RPL23	1
GO:00160:	lipid catabolic	4/272	333/18670	0.717986	0.793033	0.684088	HSD17B11	4
GO:00303:	negative regul	4/272	334/18670	0.720165	0.795191	0.68595	THBS1/PFN	4
GO:00165:	negative regul	2/272	173/18670	0.720551	0.795369	0.686104	THBS1/STA	2
GO:00458:	positive regula	1/272	87/18670	0.721902	0.796117	0.686748	LMOD3	1
GO:00486:	positive regula	1/272	87/18670	0.721902	0.796117	0.686748	LMOD3	1
GO:00517!	positive regula	1/272	87/18670	0.721902	0.796117	0.686748	NKX3-1	1
GO:00224(	molting cycle	1/272	88/18670	0.725973	0.798119	0.688476	TGM3	1
GO:00224(	hair cycle proc	1/272	88/18670	0.725973	0.798119	0.688476	TGM3	1
GO:00320!	negative regul	1/272	88/18670	0.725973	0.798119	0.688476	NFKBIA	1
GO:00341(	regulation of t	1/272	88/18670	0.725973	0.798119	0.688476	IL18	1
GO:00430:	ATP-depender	1/272	88/18670	0.725973	0.798119	0.688476	CHD8	1

GO:00435(	regulation of J	1/272	88/18670	0.725973	0.798119	0.688476	MAP3K2	1
GO:00486	muscle organ	1/272	88/18670	0.725973	0.798119	0.688476	CHD7	1
GO:00987	skin epidermis	1/272	88/18670	0.725973	0.798119	0.688476	TGM3	1
GO:19018(	positive regula	1/272	88/18670	0.725973	0.798119	0.688476	LMOD3	1
GO:19040	regulation of e	1/272	88/18670	0.725973	0.798119	0.688476	THBS1	1
GO:00026	negative regul	2/272	175/1867(	0.726427	0.798123	0.688479	MNDA/IL4	2
GO:20001	negative regul	2/272	175/1867(	0.726427	0.798123	0.688479	THBS1/STA	2
GO:00725(	cellular divale	6/272	493/1867(	0.727448	0.798997	0.689233	F2RL1/S10	6
GO:00510	regulation of s	4/272	338/1867(	0.728757	0.800044	0.690136	F2RL1/RAF	4
GO:00069	response to u	2/272	176/1867(	0.729326	0.800044	0.690136	THBS1/EIF	2
GO:00303	respiratory tul	2/272	176/1867(	0.729326	0.800044	0.690136	MME/CRIS	2
GO:00464	positive regula	1/272	89/18670	0.729984	0.800044	0.690136	IL18	1
GO:00550	cardiac muscle	1/272	89/18670	0.729984	0.800044	0.690136	RGS2	1
GO:00600	roof of mouth	1/272	89/18670	0.729984	0.800044	0.690136	CHD7	1
GO:00973(	cellular respo	1/272	89/18670	0.729984	0.800044	0.690136	AHR	1
GO:00030(	heart morpho	3/272	259/1867(	0.730912	0.800814	0.6908	MDM4/HII	3
GO:00107	regulation of e	1/272	90/18670	0.733937	0.803133	0.692801	PBLD	1
GO:00140	neural crest ce	1/272	90/18670	0.733937	0.803133	0.692801	HIF1A	1
GO:00158	monoamine tı	1/272	90/18670	0.733937	0.803133	0.692801	ABAT	1
GO:00306	regulation of (	1/272	90/18670	0.733937	0.803133	0.692801	LRRK2	1
GO:19040(	regulation of (	4/272	342/1867(	0.737151	0.805442	0.694792	RGS2/HEC	4
GO:00159	phospholipid t	1/272	91/18670	0.737832	0.805442	0.694792	OSBPL8	1
GO:00305	regulation of I	1/272	91/18670	0.737832	0.805442	0.694792	TOB1	1
GO:00456	negative regul	1/272	91/18670	0.737832	0.805442	0.694792	NFKBIA	1
GO:00064	protein methy	2/272	179/1867(	0.737866	0.805442	0.694792	PCMTD1/A	2
GO:00082	protein alkylat	2/272	179/1867(	0.737866	0.805442	0.694792	PCMTD1/A	2
GO:00487	appendage de	2/272	179/1867(	0.737866	0.805442	0.694792	PBX2/CHD	2
GO:00601	limb developn	2/272	179/1867(	0.737866	0.805442	0.694792	PBX2/CHD	2
GO:00224(	negative regul	2/272	180/1867(	0.740662	0.807995	0.696995	IL4R/ABAT	2
GO:00307(	cytoskeleton-(	2/272	180/1867(	0.740662	0.807995	0.696995	KIF1B/HIF1	2
GO:00066	ceramide met	1/272	92/18670	0.74167	0.808596	0.697513	ASAH1	1
GO:19048	positive regula	1/272	92/18670	0.74167	0.808596	0.697513	IL18	1
GO:01400	mitotic nuclea	3/272	264/1867(	0.742686	0.809454	0.698254	TACC3/GO	3
GO:00069(	vesicle targeti	1/272	93/18670	0.745452	0.810473	0.699133	GOLGA2	1
GO:00352	miRNA mediat	1/272	93/18670	0.745452	0.810473	0.699133	TNRC6B	1
GO:00400	negative regul	1/272	93/18670	0.745452	0.810473	0.699133	TNRC6B	1
GO:00459	regulation of t	1/272	93/18670	0.745452	0.810473	0.699133	TNRC6B	1
GO:00516(	localization wi	1/272	93/18670	0.745452	0.810473	0.699133	CDH2	1
GO:00720	nephron tubul	1/272	93/18670	0.745452	0.810473	0.699133	STAT1	1
GO:19016	cellular respo	1/272	93/18670	0.745452	0.810473	0.699133	AHR	1
GO:19033	cellular respo	1/272	93/18670	0.745452	0.810473	0.699133	LRRK2	1
GO:00020	sprouting angi	2/272	183/1867(	0.748897	0.812778	0.701121	PTGS2/THI	2
GO:00219	central nervou	2/272	183/1867(	0.748897	0.812778	0.701121	B2M/EOM	2
GO:00007	non-recombin	1/272	94/18670	0.749179	0.812778	0.701121	DTX3L	1
GO:00060	proteoglycan	1/272	94/18670	0.749179	0.812778	0.701121	SULF2	1
GO:00447	DNA methylat	1/272	94/18670	0.749179	0.812778	0.701121	TDRD1	1
GO:19033	response to d	1/272	94/18670	0.749179	0.812778	0.701121	LRRK2	1
GO:19905	mitochondrial	1/272	94/18670	0.749179	0.812778	0.701121	SLC25A20	1

GO:20001	negative regul	4/272	349/1867(	0.75137	0.814267	0.702405	THBS1/PFN	4
GO:00199	cAMP-mediat	2/272	184/1867(	0.751592	0.814267	0.702405	RGS2/AHR	2
GO:00486	positive regul	2/272	184/1867(	0.751592	0.814267	0.702405	TNFRSF12/	2
GO:00028	negative regul	1/272	95/18670	0.752852	0.814267	0.702405	PPM1B	1
GO:00508	regulation of s	1/272	95/18670	0.752852	0.814267	0.702405	ASAH1	1
GO:00550	cardiac cell de	1/272	95/18670	0.752852	0.814267	0.702405	RGS2	1
GO:00613	renal tubule d	1/272	95/18670	0.752852	0.814267	0.702405	STAT1	1
GO:01100	regulation of a	1/272	95/18670	0.752852	0.814267	0.702405	PFN2	1
GO:19030	regulation of p	1/272	95/18670	0.752852	0.814267	0.702405	PICALM	1
GO:20000	positive regul	1/272	95/18670	0.752852	0.814267	0.702405	LRRK2	1
GO:00512	protein homo	4/272	351/1867(	0.755323	0.81669	0.704495	UPB1/B2M	4
GO:00075	sex differentia	3/272	270/1867(	0.756272	0.817432	0.705135	NKX3-1/CH	3
GO:00426	cholesterol hc	1/272	96/18670	0.756471	0.817432	0.705135	IL18	1
GO:19013	negative regul	2/272	187/1867(	0.759527	0.819285	0.706734	THBS1/STA	2
GO:00105	negative regul	1/272	97/18670	0.760037	0.819285	0.706734	THBS1	1
GO:00140	primary neura	1/272	97/18670	0.760037	0.819285	0.706734	HIF1A	1
GO:00303	osteoclast diff	1/272	97/18670	0.760037	0.819285	0.706734	OSCAR	1
GO:00322	regulation of a	1/272	97/18670	0.760037	0.819285	0.706734	PFN2	1
GO:00487	oligodendrocy	1/272	97/18670	0.760037	0.819285	0.706734	TLR2	1
GO:00550	sterol homeos	1/272	97/18670	0.760037	0.819285	0.706734	IL18	1
GO:00906	activation of C	1/272	97/18670	0.760037	0.819285	0.706734	RAPGEF1	1
GO:00063	transcription i	2/272	188/1867(	0.762124	0.821034	0.708242	POLR2J/TA	2
GO:00517	regulation of r	2/272	188/1867(	0.762124	0.821034	0.708242	TACC3/HE(	2
GO:00987	cell-cell adhes	3/272	273/1867(	0.762846	0.821322	0.708491	NTNG2/CD	3
GO:00018	blastocyst dev	1/272	98/18670	0.763551	0.821322	0.708491	EOMES	1
GO:00068	regulation of p	1/272	98/18670	0.763551	0.821322	0.708491	LRRK2	1
GO:00424	inner ear mor	1/272	98/18670	0.763551	0.821322	0.708491	CHD7	1
GO:00601	regulation of l	1/272	98/18670	0.763551	0.821322	0.708491	RGS2	1
GO:00008	sister chromat	2/272	189/1867(	0.764696	0.822303	0.709337	TACC3/HE(	2
GO:00514	regulation of c	4/272	357/1867(	0.766892	0.823296	0.710194	F2RL1/CYS	4
GO:00017	cell fate speci	1/272	99/18670	0.767014	0.823296	0.710194	EOMES	1
GO:00193	fatty acid oxid	1/272	99/18670	0.767014	0.823296	0.710194	IRS2	1
GO:00303	cholesterol tra	1/272	99/18670	0.767014	0.823296	0.710194	NFKBIA	1
GO:00620	negative regul	1/272	99/18670	0.767014	0.823296	0.710194	HDAC4	1
GO:00725	pyrimidine-co	1/272	99/18670	0.767014	0.823296	0.710194	UPB1	1
GO:00510	positive regul	2/272	191/1867(	0.769767	0.825208	0.711843	EXOSC3/D	2
GO:00072	glutamate rec	1/272	100/1867(	0.770427	0.825208	0.711843	CDK5R1	1
GO:00226	digestive syste	1/272	100/1867(	0.770427	0.825208	0.711843	PBLD	1
GO:00344	substrate adh	1/272	100/1867(	0.770427	0.825208	0.711843	MEGF9	1
GO:00352	segmentation	1/272	100/1867(	0.770427	0.825208	0.711843	NKX3-1	1
GO:00365	ERAD pathway	1/272	100/1867(	0.770427	0.825208	0.711843	TRIM25	1
GO:00550	regulation of c	1/272	100/1867(	0.770427	0.825208	0.711843	RGS2	1
GO:19011	carbohydrate	2/272	192/1867(	0.772268	0.826929	0.713328	PDE7A/UP	2
GO:00305	intracellular re	3/272	278/1867(	0.773483	0.827557	0.71387	NKX3-1/NF	3
GO:00000	regulation of l	1/272	101/1867(	0.773789	0.827557	0.71387	EXOSC3	1
GO:00076	feeding behav	1/272	101/1867(	0.773789	0.827557	0.71387	EIF2AK4	1
GO:00344	lipid oxidation	1/272	101/1867(	0.773789	0.827557	0.71387	IRS2	1
GO:00506	nucleic acid tr	2/272	193/1867(	0.774744	0.828078	0.714319	UPF2/RAE	2

GO:00506!	RNA transport	2/272	193/1867(	0.774744	0.828078	0.714319	UPF2/RAE:	2
GO:00025(	antigen proce:	1/272	102/1867(	0.777103	0.829448	0.7155	THBS1	1
GO:00105:	regulation of (	1/272	102/1867(	0.777103	0.829448	0.7155	CHD7	1
GO:00301⁄	sphingolipid b	1/272	102/1867(	0.777103	0.829448	0.7155	ASAH1	1
GO:00458:	negative regul	1/272	102/1867(	0.777103	0.829448	0.7155	EPC1	1
GO:19021:	regulation of (	2/272	194/1867(	0.777197	0.829448	0.7155	RAB3GAP1	2
GO:00072:	integrin-medi:	1/272	103/1867(	0.780368	0.832079	0.71777	BST1	1
GO:00189!	phenol-contai	1/272	103/1867(	0.780368	0.832079	0.71777	ABAT	1
GO:19021(	negative regul	1/272	103/1867(	0.780368	0.832079	0.71777	IL4R	1
GO:00600⁄	heart contract	3/272	282/1867(	0.781711	0.832852	0.718437	RGS2/TNN	3
GO:0017(	morphogenes	2/272	196/1867(	0.782034	0.832852	0.718437	NKX3-1/LR	2
GO:00512:	establishment	2/272	196/1867(	0.782034	0.832852	0.718437	UPF2/RAE:	2
GO:00718!	DNA biosynth	2/272	196/1867(	0.782034	0.832852	0.718437	USP10/TRI	2
GO:00086:	intrinsic apopt	1/272	104/1867(	0.783586	0.833752	0.719213	RPL26	1
GO:00901(	positive regul:	1/272	104/1867(	0.783586	0.833752	0.719213	THBS1	1
GO:19018(	positive regul:	1/272	104/1867(	0.783586	0.833752	0.719213	LRRK2	1
GO:00018⁄	neural tube fo	1/272	105/1867(	0.786756	0.835642	0.720843	HIF1A	1
GO:00066⁄	triglyceride m	1/272	105/1867(	0.786756	0.835642	0.720843	ACSL1	1
GO:00070(	plasma memb	1/272	105/1867(	0.786756	0.835642	0.720843	BIN3	1
GO:00085!	regulation of I	1/272	105/1867(	0.786756	0.835642	0.720843	NFKBIA	1
GO:00550:	cardiac muscl	1/272	105/1867(	0.786756	0.835642	0.720843	RGS2	1
GO:00468!	regulation of I	2/272	198/1867(	0.786778	0.835642	0.720843	PTGS2/AS/	2
GO:00070:	microtubule-b	3/272	285/1867(	0.787719	0.83639	0.721489	ROPN1L/K	3
GO:00359(	response to tc	2/272	199/1867(	0.789116	0.837622	0.722552	THBS1/EIF:	2
GO:00509(	neuromuscula	1/272	107/1867(	0.792959	0.84145	0.725853	CHD8	1
GO:00067!	sulfur compou	4/272	372/1867(	0.793952	0.84225	0.726544	DLD/ACSL1	4
GO:00300(	cellular mono	1/272	108/1867(	0.795993	0.844163	0.728194	LRRK2	1
GO:00062!	nucleotide-ex	1/272	109/1867(	0.798982	0.846066	0.729835	POLR2J	1
GO:00091⁄	nucleoside tri	1/272	109/1867(	0.798982	0.846066	0.729835	LRRK2	1
GO:00718(	cellular respo	1/272	109/1867(	0.798982	0.846066	0.729835	LRRK2	1
GO:00718:	cellular respo	1/272	109/1867(	0.798982	0.846066	0.729835	LRRK2	1
GO:00720(	nephron epith	1/272	109/1867(	0.798982	0.846066	0.729835	STAT1	1
GO:00064:	protein acetyl	2/272	204/1867(	0.800471	0.847388	0.730976	RPS6KA5/E	2
GO:00030:	heart process	3/272	292/1867(	0.801208	0.847916	0.731431	RGS2/TNN	3
GO:00060:	glycosaminogl	1/272	110/1867(	0.801928	0.848424	0.731869	ABCC5	1
GO:00316:	receptor inter	1/272	111/1867(	0.804831	0.850986	0.73408	PICALM	1
GO:19040:	epithelial cell	1/272	111/1867(	0.804831	0.850986	0.73408	THBS1	1
GO:00485:	rhythmic proc	3/272	295/1867(	0.806767	0.852229	0.735152	CDK5R1/CI	3
GO:00066⁄	membrane lip	2/272	207/1867(	0.807022	0.852229	0.735152	ASAH1/MF	2
GO:00019:	positive regul:	1/272	112/1867(	0.807691	0.852229	0.735152	HIF1A	1
GO:00063!	DNA-template	1/272	112/1867(	0.807691	0.852229	0.735152	POLR2J	1
GO:00508(	negative regul	1/272	112/1867(	0.807691	0.852229	0.735152	IL4R	1
GO:00604:	heart growth	1/272	112/1867(	0.807691	0.852229	0.735152	RGS2	1
GO:19035:	mucopolysacc	1/272	112/1867(	0.807691	0.852229	0.735152	ABCC5	1
GO:00400:	negative regul	4/272	381/1867(	0.808946	0.852764	0.735614	THBS1/PFI	4
GO:0042:	regulation of (	4/272	381/1867(	0.808946	0.852764	0.735614	MDM4/RP	4
GO:00160!	carbohydrate	2/272	208/1867(	0.809162	0.852764	0.735614	IRS2/PPP1	2
GO:19012:	negative regul	2/272	208/1867(	0.809162	0.852764	0.735614	HIF1A/LRR	2

GO:00466:	regulation of c	1/272	113/1867(	0.81051	0.853422	0.736181	RGS2	1
GO:00718(	response to m	1/272	113/1867(	0.81051	0.853422	0.736181	LRRK2	1
GO:00718(	response to ca	1/272	113/1867(	0.81051	0.853422	0.736181	LRRK2	1
GO:00512:	cartilage deve	2/272	209/1867(	0.811282	0.853981	0.736664	SULF2/HIF	2
GO:00485	response to st	4/272	383/1867(	0.812155	0.854646	0.737237	NKX3-1/PT	4
GO:00349(	histone lysine	1/272	114/1867(	0.813287	0.855427	0.737911	ARID4A	1
GO:00071(	establishment	2/272	210/1867(	0.813381	0.855427	0.737911	GOLPH3/A	2
GO:00512:	negative regul	4/272	384/1867(	0.813743	0.855554	0.73802	THBS1/PFI	4
GO:00060:	aminoglycan b	1/272	115/1867(	0.816024	0.857443	0.73965	ABCC5	1
GO:00466(	female sex dif	1/272	115/1867(	0.816024	0.857443	0.73965	CHD7	1
GO:00457:	positive regula	2/272	214/1867(	0.82157	0.862758	0.744234	HECW2/LR	2
GO:19030!	regulation of p	2/272	214/1867(	0.82157	0.862758	0.744234	RPL23/LRR	2
GO:00424:	ear morphoge	1/272	118/1867(	0.823997	0.864794	0.745991	CHD7	1
GO:00485!	camera-type e	1/272	118/1867(	0.823997	0.864794	0.745991	HIF1A	1
GO:00073(	determination	1/272	119/1867(	0.826578	0.867245	0.748105	HIF1A	1
GO:00084(	gonad develop	2/272	217/1867(	0.827499	0.867954	0.748717	NKX3-1/AF	2
GO:00109!	regulation of r	4/272	394/1867(	0.829028	0.868956	0.749581	PTGS2/B2I	4
GO:19030!	positive regula	1/272	120/1867(	0.829121	0.868956	0.749581	LRRK2	1
GO:00076(	visual percept	2/272	218/1867(	0.829435	0.868956	0.749581	RGR/UNC1	2
GO:00160(	immunoglobu	2/272	218/1867(	0.829435	0.868956	0.749581	IL4R/EXOS	2
GO:00434:	macromolecu	3/272	309/1867(	0.831006	0.870221	0.750672	PCMTD1/T	3
GO:00157	organophosph	1/272	121/1867(	0.831626	0.870221	0.750672	OSBPL8	1
GO:00309(	endoplasmic r	1/272	121/1867(	0.831626	0.870221	0.750672	EIF2AK2	1
GO:00702!	actin-mediate	1/272	121/1867(	0.831626	0.870221	0.750672	TNNI2	1
GO:00062{	regulation of I	1/272	122/1867(	0.834095	0.871774	0.752012	DTX3L	1
GO:00169!	antibiotic met	1/272	122/1867(	0.834095	0.871774	0.752012	HAL	1
GO:00324:	regulation of p	1/272	122/1867(	0.834095	0.871774	0.752012	LRRK2	1
GO:19054:	positive regula	1/272	122/1867(	0.834095	0.871774	0.752012	CDK5R1	1
GO:00071{	adenylate cycl	2/272	221/1867(	0.835129	0.872339	0.752499	RGS2/OXEI	2
GO:00197:	B cell mediate	2/272	221/1867(	0.835129	0.872339	0.752499	IL4R/EXOS	2
GO:00020(	chondrocyte c	1/272	123/1867(	0.836528	0.873543	0.753538	SULF2	1
GO:00509!	sensory perce	2/272	222/1867(	0.836988	0.873766	0.75373	RGR/UNC1	2
GO:00451:	development	2/272	223/1867(	0.838829	0.874756	0.754584	NKX3-1/AF	2
GO:00018:	embryonic epi	1/272	124/1867(	0.838926	0.874756	0.754584	HIF1A	1
GO:00192:	regulation of s	1/272	124/1867(	0.838926	0.874756	0.754584	ASAH1	1
GO:00512(	release of seq	1/272	124/1867(	0.838926	0.874756	0.754584	CHD7	1
GO:00016	osteoblast dif	2/272	225/1867(	0.842454	0.878176	0.757535	TOB1/HDA	2
GO:00487(	embryonic ske	1/272	126/1867(	0.843616	0.87887	0.758133	SULF2	1
GO:00512{	negative regul	1/272	126/1867(	0.843616	0.87887	0.758133	CHD7	1
GO:00430:	camera-type e	3/272	319/1867(	0.846687	0.88181	0.76067	RAB3GAP1	3
GO:00098!	determination	1/272	128/1867(	0.84817	0.882835	0.761553	HIF1A	1
GO:00512{	regulation of s	1/272	128/1867(	0.84817	0.882835	0.761553	CHD7	1
GO:00070!	chromosome	3/272	321/1867(	0.849668	0.883594	0.762209	TACC3/HE(	3
GO:00066:	acylglycerol m	1/272	129/1867(	0.850397	0.883594	0.762209	ACSL1	1
GO:00070	cell-cell juncti	1/272	129/1867(	0.850397	0.883594	0.762209	CDH2	1
GO:00097!	specification c	1/272	129/1867(	0.850397	0.883594	0.762209	HIF1A	1
GO:00180:	peptidyl-lysine	1/272	129/1867(	0.850397	0.883594	0.762209	ARID4A	1
GO:19030:	negative regul	1/272	129/1867(	0.850397	0.883594	0.762209	IL4R	1

GO:00192:	regulation of l	4/272	410/1867(	0.851316	0.88429	0.762808	PTGS2/ASA	4
GO:00066:	neutral lipid m	1/272	130/1867(	0.852592	0.885095	0.763503	ACSL1	1
GO:00305:	intracellular st	1/272	130/1867(	0.852592	0.885095	0.763503	NKX3-1	1
GO:00512(	sequestering c	1/272	131/1867(	0.854755	0.88708	0.765216	CHD7	1
GO:00097!	hormone-mec	2/272	233/1867(	0.856228	0.88799	0.766001	NKX3-1/AC	2
GO:00487:	cardiac muscle	2/272	233/1867(	0.856228	0.88799	0.766001	RGS2/CHD	2
GO:00425!	myelination	1/272	132/1867(	0.856886	0.88799	0.766001	TLR2	1
GO:00550(	cardiac muscle	1/272	132/1867(	0.856886	0.88799	0.766001	RGS2	1
GO:00721:	epithelial tube	1/272	132/1867(	0.856886	0.88799	0.766001	HIF1A	1
GO:00064:	translational e	1/272	133/1867(	0.858985	0.889906	0.767653	ABTB1	1
GO:00007:	double-strand	1/272	134/1867(	0.861055	0.890746	0.768378	MCM8	1
GO:00029:	regulation of l	1/272	134/1867(	0.861055	0.890746	0.768378	PPP2R3C	1
GO:00072:	ensheathmen	1/272	134/1867(	0.861055	0.890746	0.768378	TLR2	1
GO:00083(	axon ensheatl	1/272	134/1867(	0.861055	0.890746	0.768378	TLR2	1
GO:00323!	response to e:	1/272	134/1867(	0.861055	0.890746	0.768378	PTGS2	1
GO:00091(	glycoprotein r	4/272	419/1867(	0.862742	0.892073	0.769523	SULF2/GOI	4
GO:00007:	recombinatior	1/272	135/1867(	0.863093	0.892073	0.769523	MCM8	1
GO:00300:	establishment	1/272	135/1867(	0.863093	0.892073	0.769523	GOLPH3	1
GO:00302!	lipid modificat	2/272	238/1867(	0.86427	0.893029	0.770347	SYNJ2/IRS2	2
GO:00313	negative regul	2/272	239/1867(	0.865829	0.894378	0.771511	PPM1B/PS	2
GO:00159:	nucleobase-cc	2/272	241/1867(	0.868897	0.897285	0.774019	UPF2/RAE:	2
GO:00075{	digestion	1/272	139/1867(	0.870955	0.898887	0.7754	PBLD	1
GO:00165:	histone methy	1/272	139/1867(	0.870955	0.898887	0.7754	ARID4A	1
GO:00346:	cellular respor	1/272	140/1867(	0.872849	0.899269	0.775729	EIF2AK2	1
GO:00350:	regulation of I	1/272	140/1867(	0.872849	0.899269	0.775729	F2RL1	1
GO:00357:	sodium ion tra	1/272	140/1867(	0.872849	0.899269	0.775729	HECW2	1
GO:00720:	kidney epithel	1/272	140/1867(	0.872849	0.899269	0.775729	STAT1	1
GO:00975!	calcium ion tra	1/272	140/1867(	0.872849	0.899269	0.775729	CHD7	1
GO:19033(	positive regula	1/272	140/1867(	0.872849	0.899269	0.775729	LRRK2	1
