# Supplementary material for: The ceRNA Crosstalk between mRNAs and lncRNAs in Diabetes Myocardial Infarction
Source: Dis Markers. 2022 May 9;2022:4283534. doi: 10.1155/2022/4283534 (PMC9112177; doi:10.1155/2022/4283534)
Supplement: Supplementary 4 — Table S4: the Kyoto Encyclopedia of Genes and Genomes (KEGG) pathway enrichment for differentially expressed genes. [file 4283534.f4.pdf]

ID	Descriptio	GeneRatio	BgRatio	pvalue	p.adjust	qvalue	geneID	Count
hsa04064	NF-kappa I	9/146	104/8034	0.000107	0.02536	0.023316	GADD45G/	9
hsa04657	IL-17 signa	8/146	94/8034	0.000292	0.034642	0.03185	PTGS2/S10	8
hsa05140	Leishmania	7/146	77/8034	0.000471	0.037211	0.034211	IFNGR1/PT	7
hsa05169	Epstein-Ba	11/146	201/8034	0.00105	0.06219	0.057176	GADD45G/	11
hsa05152	Tuberculos	10/146	180/8034	0.001583	0.075036	0.068987	IFNGR1/CA	10
hsa04931	Insulin resi	7/146	108/8034	0.003426	0.122787	0.112889	RPS6KA3/F	7
hsa05134	Legionello	5/146	57/8034	0.003627	0.122787	0.112889	CLK4/TLR2	5
hsa04668	TNF signali	7/146	112/8034	0.004192	0.124182	0.114171	RPS6KA5/F	7
hsa05321	Inflammat	5/146	65/8034	0.006373	0.149957	0.137868	IFNGR1/ST	5
hsa04145	Phagosom	8/146	152/8034	0.006377	0.149957	0.137868	THBS1/CLE	8
hsa05167	Kaposi sarc	9/146	189/8034	0.007407	0.149957	0.137868	GNG10/IFN	9
hsa05168	Herpes sim	17/146	491/8034	0.007593	0.149957	0.137868	IFNGR1/ST	17
hsa04380	Osteoclast	7/146	128/8034	0.00861	0.156967	0.144313	SIRPB1/IFN	7
hsa04115	p53 signali	5/146	73/8034	0.010329	0.16319	0.150035	GADD45G/	5
hsa04620	Toll-like re	6/146	104/8034	0.011478	0.16319	0.150035	STAT1/LY9	6
hsa04625	C-type lect	6/146	104/8034	0.011478	0.16319	0.150035	CLEC4D/PT	6
hsa05164	Influenza A	8/146	170/8034	0.012142	0.16319	0.150035	IFNGR1/ST	8
hsa05132	Salmonella	9/146	206/8034	0.01262	0.16319	0.150035	TXN/PFN2/	9
hsa04659	Th17 cell d	6/146	107/8034	0.013083	0.16319	0.150035	IFNGR1/ST	6
hsa05235	PD-L1 expr	5/146	89/8034	0.022699	0.268981	0.247297	IFNGR1/ST	5
hsa03010	Ribosome	7/146	158/8034	0.024856	0.280515	0.257902	RPS28/RPS	7
hsa05215	Prostate ca	5/146	97/8034	0.031448	0.335075	0.308064	PLAU/NKX	5
hsa04640	Hematopo	5/146	99/8034	0.033924	0.335075	0.308064	CSF2RA/M	5
hsa05211	Renal cell c	4/146	69/8034	0.03644	0.335075	0.308064	PAK2/RAP	4
hsa04210	Apoptosis	6/146	136/8034	0.037273	0.335075	0.308064	GADD45G/	6
hsa05219	Bladder ca	3/146	41/8034	0.037914	0.335075	0.308064	RPS6KA5/T	3
hsa04910	Insulin sigr	6/146	137/8034	0.038419	0.335075	0.308064	RAPGEF1/I	6
hsa05162	Measles	6/146	138/8034	0.039587	0.335075	0.308064	STAT1/EIF2	6
hsa04010	MAPK sign	10/146	294/8034	0.041361	0.338019	0.31077	GADD45G/	10
hsa04621	NOD-like re	7/146	181/8034	0.04664	0.368457	0.338755	TXN/CAMP	7
hsa03050	Proteasom	3/146	46/8034	0.050599	0.374749	0.344539	PSMD12/P	3
hsa04930	Type II dial	3/146	46/8034	0.050599	0.374749	0.344539	CACNA1E/	3
hsa05145	Toxoplasm	5/146	112/8034	0.052938	0.380191	0.349542	IFNGR1/ST	5
hsa00770	Pantothen	2/146	21/8034	0.05498	0.383246	0.352351	UPB1/VNN	2
hsa05144	Malaria	3/146	50/8034	0.062042	0.407304	0.37447	THBS1/TLR	3
hsa05160	Hepatitis C	6/146	155/8034	0.062945	0.407304	0.37447	STAT1/IFIT	6
hsa04614	Renin-angi	2/146	23/8034	0.064709	0.407304	0.37447	MME/ANP	2
hsa04722	Neurotrop	5/146	119/8034	0.065306	0.407304	0.37447	RPS6KA3/F	5
hsa04152	AMPK sign	5/146	120/8034	0.067195	0.408339	0.375421	PPP2R3A/F	5
hsa05131	Shigellosis	8/146	242/8034	0.073476	0.430725	0.396003	RPS6KA5/U	8
hsa05161	Hepatitis B	6/146	162/8034	0.074514	0.430725	0.396003	STAT1/CRE	6
hsa05205	Proteoglyc	7/146	205/8034	0.079567	0.447649	0.411562	PLAU/THBS	7
hsa04658	Th1 and Th	4/146	92/8034	0.086111	0.447649	0.411562	IFNGR1/ST	4
hsa05222	Small cell l	4/146	92/8034	0.086111	0.447649	0.411562	GADD45G/	4
hsa04060	Cytokine-c	9/146	294/8034	0.086958	0.447649	0.411562	CSF2RA/IFI	9
hsa00524	Neomycin,	1/146	5/8034	0.087642	0.447649	0.411562	HK2	1
hsa05323	Rheumato	4/146	93/8034	0.088774	0.447649	0.411562	TNFSF13B/	4

hsa05170	Human im	7/146	212/8034	0.09121	0.45035	0.414046	GNG10/PA	7
hsa05200	Pathways i	14/146	531/8034	0.101878	0.486241	0.447043	GADD45G/	14
hsa00410	beta-Alani	2/146	30/8034	0.102582	0.486241	0.447043	UPB1/ABA	2
hsa01523	Antifolate	2/146	31/8034	0.108401	0.503744	0.463136	ABCC5/FO	2
hsa05142	Chagas dis	4/146	102/8034	0.114478	0.51532	0.473779	IFNGR1/FA	4
hsa05163	Human cyt	7/146	225/8034	0.11524	0.51532	0.473779	GNG10/PT	7
hsa00051	Fructose a	2/146	33/8034	0.120289	0.527936	0.485377	PFKFB2/HK	2
hsa00640	Propanoat	2/146	34/8034	0.12635	0.538807	0.495372	DLD/ABAT	2
hsa04920	Adipocytol	3/146	69/8034	0.130118	0.538807	0.495372	ACSL1/IRS	3
hsa00760	Nicotinate	2/146	35/8034	0.132482	0.538807	0.495372	NT5C2/BST	2
hsa05010	Alzheimer	10/146	369/8034	0.133951	0.538807	0.495372	PTGS2/MN	10
hsa04622	RIG-I-like r	3/146	70/8034	0.134238	0.538807	0.495372	TRIM25/FA	3
hsa05130	Pathogeni	6/146	192/8034	0.136681	0.538807	0.495372	PAK2/FADI	6
hsa00500	Starch and	2/146	36/8034	0.13868	0.538807	0.495372	PYGL/HK2	2
hsa05143	African try	2/146	37/8034	0.144941	0.545255	0.5013	F2RL1/IL1	2
hsa05216	Thyroid ca	2/146	37/8034	0.144941	0.545255	0.5013	GADD45G/	2
hsa05214	Glioma	3/146	75/8034	0.155484	0.574021	0.527748	GADD45G/	3
hsa05212	Pancreatic	3/146	76/8034	0.159854	0.574021	0.527748	GADD45G/	3
hsa05220	Chronic m	3/146	76/8034	0.159854	0.574021	0.527748	GADD45G/	3
hsa04217	Necroptos	5/146	159/8034	0.163024	0.576669	0.530182	IFNGR1/ST	5
hsa04630	JAK-STAT s	5/146	162/8034	0.171986	0.59942	0.551099	CSF2RA/IF	5
hsa04940	Type I diab	2/146	43/8034	0.183585	0.630575	0.579743	ICA1/PTPR	2
hsa02010	ABC transp	2/146	45/8034	0.196786	0.666263	0.612553	ABCC5/TA	2
hsa05206	MicroRNA	8/146	310/8034	0.202145	0.674765	0.62037	PLAU/RPS	8
hsa04926	Relaxin sig	4/146	129/8034	0.207327	0.682451	0.627436	GNG10/CR	4
hsa00280	Valine, leu	2/146	48/8034	0.216794	0.700673	0.644189	DLD/ABAT	2
hsa04728	Dopamine	4/146	132/8034	0.218775	0.700673	0.644189	GNG10/PP	4
hsa05030	Cocaine ac	2/146	49/8034	0.223505	0.706276	0.649341	CREB5/CD	2
hsa03015	mRNA surv	3/146	91/8034	0.229218	0.714799	0.657177	PPP2R3A/	3
hsa05165	Human pa	8/146	330/8034	0.251319	0.763534	0.701984	PPP2R3A/	8
hsa05150	Staphyloc	3/146	96/8034	0.253511	0.763534	0.701984	CAMP/FCG	3
hsa00730	Thiamine r	1/146	16/8034	0.254511	0.763534	0.701984	ALPP	1
hsa04062	Chemokin	5/146	189/8034	0.259973	0.77017	0.708084	GNG10/ST	5
hsa05202	Transcripti	5/146	192/8034	0.270387	0.78008	0.717195	GADD45G/	5
hsa04923	Regulation	2/146	56/8034	0.270791	0.78008	0.717195	PTGS2/IRS	2
hsa00240	Pyrimidine	2/146	57/8034	0.277559	0.78008	0.717195	UPB1/NT5	2
hsa01212	Fatty acid	2/146	57/8034	0.277559	0.78008	0.717195	ACSL1/HSD	2
hsa00061	Fatty acid	1/146	18/8034	0.281415	0.78008	0.717195	ACSL1	1
hsa05213	Endometri	2/146	58/8034	0.284325	0.78008	0.717195	GADD45G/	2
hsa04261	Adrenergic	4/146	149/8034	0.286358	0.78008	0.717195	PPP2R3A/	4
hsa05012	Parkinson	6/146	249/8034	0.299229	0.799963	0.735476	TXN/PSMD	6
hsa04510	Focal adhe	5/146	201/8034	0.302137	0.799963	0.735476	PAK2/RAP	5
hsa04921	Oxytocin s	4/146	154/8034	0.306847	0.799963	0.735476	PTGS2/CA	4
hsa04066	HIF-1 signa	3/146	109/8034	0.318006	0.799963	0.735476	IFNGR1/HK	3
hsa00590	Arachidoni	2/146	63/8034	0.318027	0.799963	0.735476	PTGS2/CY	2
hsa04623	Cytosolic D	2/146	63/8034	0.318027	0.799963	0.735476	NFKBIA/IL	2
hsa05217	Basal cell c	2/146	63/8034	0.318027	0.799963	0.735476	GADD45G/	2
hsa05016	Huntingto	7/146	306/8034	0.321461	0.799963	0.735476	POLR2J/PS	7

hsa00340	Histidine m	1/146	22/8034	0.332363	0.799963	0.735476	HAL	1
hsa04670	Leukocyte	3/146	113/8034	0.337997	0.799963	0.735476	RAP1A/MM	3
hsa00010	Glycolysis ,	2/146	67/8034	0.344732	0.799963	0.735476	DLD/HK2	2
hsa04720	Long-term	2/146	67/8034	0.344732	0.799963	0.735476	RPS6KA3/F	2
hsa04810	Regulation	5/146	214/8034	0.348921	0.799963	0.735476	PAK2/PFN2	5
hsa04137	Mitophagy	2/146	68/8034	0.351359	0.799963	0.735476	USP15/HIF	2
hsa05223	Non-small	2/146	68/8034	0.351359	0.799963	0.735476	GADD45G/	2
hsa04977	Vitamin dig	1/146	24/8034	0.356475	0.799963	0.735476	LMBRD1	1
hsa04022	cGMP-PKG	4/146	167/8034	0.360673	0.799963	0.735476	RGS2/CREI	4
hsa04141	Protein pro	4/146	167/8034	0.360673	0.799963	0.735476	UBE2D3/EI	4
hsa05120	Epithelial c	2/146	70/8034	0.364543	0.799963	0.735476	NFKBIA/CX	2
hsa05230	Central car	2/146	70/8034	0.364543	0.799963	0.735476	HK2/HIF1A	2
hsa04071	Sphingolip	3/146	119/8034	0.367916	0.799963	0.735476	PPP2R3A/I	3
hsa04935	Growth ho	3/146	119/8034	0.367916	0.799963	0.735476	STAT1/CRE	3
hsa05218	Melanoma	2/146	72/8034	0.377626	0.80352	0.738746	GADD45G/	2
hsa04919	Thyroid ho	3/146	121/8034	0.377845	0.80352	0.738746	PFKFB2/ST	3
hsa00790	Folate bios	1/146	26/8034	0.379722	0.80352	0.738746	ALPP	1
hsa05100	Bacterial ir	2/146	73/8034	0.384127	0.805646	0.740701	CLTCL1/AR	2
hsa00062	Fatty acid (	1/146	27/8034	0.391031	0.805864	0.740901	HSD17B12	1
hsa01040	Biosynthes	1/146	27/8034	0.391031	0.805864	0.740901	HSD17B12	1
hsa00650	Butanoate	1/146	28/8034	0.402135	0.815972	0.750194	ABAT	1
hsa04714	Thermoger	5/146	231/8034	0.410621	0.815972	0.750194	RPS6KA3/A	5
hsa04142	Lysosome	3/146	128/8034	0.412319	0.815972	0.750194	ASAH1/CLT	3
hsa04612	Antigen pr	2/146	78/8034	0.416174	0.815972	0.750194	B2M/TAP2	2
hsa05135	Yersinia inf	3/146	130/8034	0.422068	0.815972	0.750194	RPS6KA3/M	3
hsa03018	RNA degra	2/146	79/8034	0.422485	0.815972	0.750194	TOB1/EXO	2
hsa00020	Citrate cyc	1/146	30/8034	0.423743	0.815972	0.750194	DLD	1
hsa00630	Glyoxylate	1/146	30/8034	0.423743	0.815972	0.750194	DLD	1
hsa04068	FoxO signa	3/146	131/8034	0.426922	0.815972	0.750194	GADD45G/	3
hsa00052	Galactose	1/146	31/8034	0.434255	0.816813	0.750967	HK2	1
hsa03020	RNA polym	1/146	31/8034	0.434255	0.816813	0.750967	POLR2J	1
hsa04215	Apoptosis	1/146	32/8034	0.444576	0.829641	0.762762	FADD	1
hsa04140	Autophagy	3/146	137/8034	0.455739	0.836049	0.768653	EIF2AK4/H	3
hsa04610	Compleme	2/146	85/8034	0.459596	0.836049	0.768653	PLAU/PLAU	2
hsa04151	PI3K-Akt si	7/146	354/8034	0.465037	0.836049	0.768653	GNG10/PP	7
hsa05418	Fluid shear	3/146	139/8034	0.465213	0.836049	0.768653	TXN/MMP	3
hsa05210	Colorectal	2/146	86/8034	0.465648	0.836049	0.768653	GADD45G/	2
hsa04540	Gap junctio	2/146	88/8034	0.477632	0.840468	0.772716	MAP3K2/T	2
hsa04211	Longevity r	2/146	89/8034	0.483563	0.840468	0.772716	CREB5/IRS	2
hsa04727	GABAergic	2/146	89/8034	0.483563	0.840468	0.772716	GNG10/AB	2
hsa00250	Alanine, as	1/146	36/8034	0.484023	0.840468	0.772716	ABAT	1
hsa05032	Morphine	2/146	91/8034	0.4953	0.840468	0.772716	GNG10/PD	2
hsa05340	Primary im	1/146	38/8034	0.50269	0.840468	0.772716	TAP2	1
hsa04666	Fc gamma	2/146	93/8034	0.506869	0.840468	0.772716	FCGR3B/AI	2
hsa04970	Salivary se	2/146	93/8034	0.506869	0.840468	0.772716	CAMP/BST	2
hsa05203	Viral carcir	4/146	204/8034	0.510575	0.840468	0.772716	EIF2AK2/C	4
hsa04932	Non-alcoh	3/146	149/8034	0.511451	0.840468	0.772716	IRS2/MLX/	3
hsa00620	Pyruvate n	1/146	39/8034	0.51177	0.840468	0.772716	DLD	1

hsa04974	Protein dig	2/146	95/8034	0.518266	0.840468	0.772716	MME/SLC3	2
hsa00260	Glycine, se	1/146	40/8034	0.520686	0.840468	0.772716	DLD	1
hsa04216	Ferroptosi	1/146	41/8034	0.52944	0.840468	0.772716	ACSL1	1
hsa04713	Circadian e	2/146	97/8034	0.529488	0.840468	0.772716	GNG10/RP	2
hsa04015	Rap1 signa	4/146	210/8034	0.533545	0.840468	0.772716	RAPGEF1/	4
hsa04925	Aldosteror	2/146	98/8034	0.535033	0.840468	0.772716	CAMK1D/C	2
hsa00380	Tryptopha	1/146	42/8034	0.538035	0.840468	0.772716	DLD	1
hsa04934	Cushing sy	3/146	155/8034	0.538178	0.840468	0.772716	CREB5/RAI	3
hsa04061	Viral prote	2/146	100/8034	0.545989	0.840468	0.772716	IL18/CXCL1	2
hsa00071	Fatty acid	1/146	44/8034	0.554761	0.840468	0.772716	ACSL1	1
hsa04962	Vasopressi	1/146	44/8034	0.554761	0.840468	0.772716	CREB5	1
hsa04024	cAMP sign	4/146	216/8034	0.555979	0.840468	0.772716	CREB5/PPF	4
hsa04972	Pancreatic	2/146	102/8034	0.556766	0.840468	0.772716	RAP1A/BS	2
hsa05146	Amoebiasi	2/146	102/8034	0.556766	0.840468	0.772716	TLR2/CXCL	2
hsa03022	Basal trans	1/146	45/8034	0.562897	0.844345	0.77628	TAF15	1
hsa04660	T cell recep	2/146	104/8034	0.567361	0.845689	0.777516	PAK2/NFKI	2
hsa04922	Glucagon s	2/146	106/8034	0.577775	0.846658	0.778406	PYGL/CREE	2
hsa00600	Sphingolip	1/146	47/8034	0.578728	0.846658	0.778406	ASAH1	1
hsa04973	Carbohydr	1/146	47/8034	0.578728	0.846658	0.778406	HK2	1
hsa00520	Amino sug	1/146	48/8034	0.586429	0.85266	0.783925	HK2	1
hsa04672	Intestinal i	1/146	49/8034	0.59399	0.858387	0.78919	TNFSF13B	1
hsa04913	Ovarian ste	1/146	51/8034	0.608702	0.873513	0.803096	PTGS2	1
hsa04725	Cholinergi	2/146	113/8034	0.612781	0.873513	0.803096	GNG10/CR	2
hsa04726	Serotoner	2/146	115/8034	0.62237	0.873513	0.803096	GNG10/PT	2
hsa04330	Notch sign	1/146	53/8034	0.622885	0.873513	0.803096	DTX3L	1
hsa04961	Endocrine	1/146	53/8034	0.622885	0.873513	0.803096	CLTCL1	1
hsa01200	Carbon me	2/146	117/8034	0.631777	0.880772	0.80977	DLD/HK2	2
hsa03013	RNA transp	3/146	180/8034	0.640012	0.884786	0.813461	TACC3/UPI	3
hsa00480	Glutathion	1/146	57/8034	0.649737	0.884786	0.813461	ANPEP	1
hsa04370	VEGF signa	1/146	59/8034	0.662442	0.884786	0.813461	PTGS2	1
hsa04110	Cell cycle	2/146	124/8034	0.663273	0.884786	0.813461	GADD45G/	2
hsa04611	Platelet ac	2/146	124/8034	0.663273	0.884786	0.813461	PPP1R12A/	2
hsa04144	Endocytosi	4/146	248/8034	0.665146	0.884786	0.813461	CLTCL1/FC	4
hsa05034	Alcoholism	3/146	187/8034	0.66557	0.884786	0.813461	GNG10/CR	3
hsa00140	Steroid ho	1/146	61/8034	0.674689	0.884786	0.813461	HSD17B12	1
hsa00310	Lysine deg	1/146	61/8034	0.674689	0.884786	0.813461	DLD	1
hsa00561	Glycerolipi	1/146	61/8034	0.674689	0.884786	0.813461	MBOAT1	1
hsa04213	Longevity	1/146	62/8034	0.680646	0.884786	0.813461	IRS2	1
hsa00230	Purine met	2/146	130/8034	0.688529	0.884786	0.813461	PDE7A/NT	2
hsa04650	Natural kill	2/146	131/8034	0.692585	0.884786	0.813461	IFNGR1/FC	2
hsa04020	Calcium sig	3/146	196/8034	0.696476	0.884786	0.813461	CACNA1E/	3
hsa04927	Cortisol sy	1/146	65/8034	0.697874	0.884786	0.813461	CREB5	1
hsa05017	Spinocereb	2/146	133/8034	0.700567	0.884786	0.813461	PSMD12/P	2
hsa04664	Fc epsilon	1/146	68/8034	0.71418	0.884786	0.813461	ALOX5AP	1
hsa04371	Apelin sign	2/146	137/8034	0.71602	0.884786	0.813461	GNG10/HD	2
hsa05031	Amphetam	1/146	69/8034	0.719418	0.884786	0.813461	CREB5	1
hsa04915	Estrogen si	2/146	138/8034	0.719778	0.884786	0.813461	CREB5/MN	2
hsa04917	Prolactin s	1/146	70/8034	0.724561	0.884786	0.813461	STAT1	1

hsa00562	Inositol ph	1/146	73/8034	0.739436	0.884786	0.813461	SYNJ2	1
hsa01524	Platinum d	1/146	73/8034	0.739436	0.884786	0.813461	FADD	1
hsa04918	Thyroid ho	1/146	75/8034	0.748906	0.884786	0.813461	CREB5	1
hsa05224	Breast can	2/146	147/8034	0.751758	0.884786	0.813461	GADD45G/	2
hsa05133	Pertussis	1/146	76/8034	0.753512	0.884786	0.813461	LY96	1
hsa04514	Cell adhesi	2/146	148/8034	0.755112	0.884786	0.813461	NTNG2/CD	2
hsa04723	Retrograde	2/146	148/8034	0.755112	0.884786	0.813461	GNG10/PT	2
hsa03320	PPAR signa	1/146	77/8034	0.758034	0.884786	0.813461	ACSL1	1
hsa05412	Arrhythmo	1/146	77/8034	0.758034	0.884786	0.813461	CDH2	1
hsa03040	Spliceosom	2/146	149/8034	0.758427	0.884786	0.813461	RBMXL1/R	2
hsa05226	Gastric car	2/146	149/8034	0.758427	0.884786	0.813461	GADD45G/	2
hsa04721	Synaptic ve	1/146	78/8034	0.762474	0.884786	0.813461	CLTCL1	1
hsa05166	Human T-c	3/146	219/8034	0.765632	0.884786	0.813461	B2M/CREB	3
hsa00983	Drug meta	1/146	79/8034	0.766833	0.884786	0.813461	UPB1	1
hsa04662	B cell rece	1/146	82/8034	0.779438	0.884786	0.813461	NFKBIA	1
hsa05204	Chemical c	1/146	82/8034	0.779438	0.884786	0.813461	PTGS2	1
hsa04218	Cellular se	2/146	156/8034	0.78057	0.884786	0.813461	GADD45G/	2
hsa04146	Peroxisom	1/146	83/8034	0.783488	0.884786	0.813461	ACSL1	1
hsa04012	ErbB signal	1/146	85/8034	0.791367	0.884786	0.813461	PAK2	1
hsa04911	Insulin sec	1/146	86/8034	0.795199	0.884786	0.813461	CREB5	1
hsa04530	Tight junct	2/146	161/8034	0.795287	0.884786	0.813461	RAP1A/TU	2
hsa04014	Ras signali	3/146	232/8034	0.798761	0.884786	0.813461	GNG10/PA	3
hsa04260	Cardiac mu	1/146	87/8034	0.798961	0.884786	0.813461	COX7B	1
hsa04512	ECM-recep	1/146	88/8034	0.802654	0.884786	0.813461	THBS1	1
hsa05225	Hepatocell	2/146	168/8034	0.814434	0.886962	0.815461	GADD45G/	2
hsa04912	GnRH signa	1/146	93/8034	0.820134	0.886962	0.815461	MAP3K2	1
hsa04350	TGF-beta s	1/146	94/8034	0.823441	0.886962	0.815461	THBS1	1
hsa04070	Phosphatic	1/146	97/8034	0.833004	0.886962	0.815461	SYNJ2	1
hsa00564	Glyceroph	1/146	98/8034	0.836076	0.886962	0.815461	MBOAT1	1
hsa01522	Endocrine	1/146	98/8034	0.836076	0.886962	0.815461	MMP9	1
hsa05231	Choline me	1/146	98/8034	0.836076	0.886962	0.815461	HIF1A	1
hsa04914	Progesterc	1/146	99/8034	0.839092	0.886962	0.815461	RPS6KA3	1
hsa04750	Inflammat	1/146	100/8034	0.842052	0.886962	0.815461	F2RL1	1
hsa04933	AGE-RAGE	1/146	100/8034	0.842052	0.886962	0.815461	STAT1	1
hsa04360	Axon guida	2/146	181/8034	0.845814	0.886982	0.81548	PAK2/NTN	2
hsa04928	Parathyroi	1/146	106/8034	0.858714	0.896542	0.82427	CREB5	1
hsa04724	Glutamate	1/146	114/8034	0.878244	0.912911	0.839319	GNG10	1
hsa04114	Oocyte me	1/146	128/8034	0.906186	0.937843	0.862241	RPS6KA3	1
hsa00190	Oxidative p	1/146	133/8034	0.914536	0.938997	0.863302	COX7B	1
hsa04270	Vascular sr	1/146	135/8034	0.917666	0.938997	0.863302	PPP1R12A	1
hsa05322	Systemic lu	1/146	136/8034	0.919187	0.938997	0.863302	FCGR3B	1
hsa04120	Ubiquitin r	1/146	140/8034	0.925	0.94088	0.865033	UBE2D3	1
hsa04150	mTOR sign	1/146	155/8034	0.943332	0.954518	0.877571	RPS6KA3	1
hsa04310	Wnt signal	1/146	160/8034	0.948393	0.954518	0.877571	CHD8	1
hsa04080	Neuroactiv	3/146	340/8034	0.95049	0.954518	0.877571	F2RL1/LTB	3
hsa04740	Olfactory t	1/146	448/8034	0.999787	0.999787	0.919192	RGS2	1
