# Supplementary material for: The ceRNA Crosstalk between mRNAs and lncRNAs in Diabetes Myocardial Infarction
Source: Dis Markers. 2022 May 9;2022:4283534. doi: 10.1155/2022/4283534 (PMC9112177; doi:10.1155/2022/4283534)
Supplement: Supplementary 5 — Table S5: detailed mRNAs and lncRNAs in each WGCNA module. [file 4283534.f5.pdf]

Probe_Set_ID	Symbol	Protein_Pr	Array_Adc	Probe_Typ	Probe_Sta	SEQUENCE	Chromoso	Probe_Chr
ILMN_1653412	RAXL1	NP_11614	3830382	S	2182	TTGAGACC	19	-
ILMN_1654320	LOC64425	XP_93429	940386	S	259	AAGTGGA	13	-
ILMN_1656233	LOC64386	XP_93860	3610465	I	3261	GGAGCCC	7	+
ILMN_1656393	PPP2R3A	NP_87162	540041	A	4082	CACTCCAG	3	+
ILMN_1658921	LOC64485	XP_93931	2360053	I	879	CCCAGCG	1	+
ILMN_1659878	PAK2	XP_94160	6110608	I	1688	AGAGGTGCGGTCGATCGGCGAAG		
ILMN_1660942	LOC65335	XP_93940	4540653	I	2039	AAGTAGA	16	-
ILMN_1672661	SP110	NP_00450	70338	A	1274	GGTGGAT	2	-
ILMN_1675258	LOC44126	NP_00101	3840102	S	2048	CAGGCCC	7	-
ILMN_1676665	CUEDC1	NP_06041	2680725	S	2114	GCAGCAG	17	-
ILMN_1677262	LOC731706		4180564	S	466	AGCCTGAGCTGCTGGAAACTATTC		
ILMN_1677843	RAB24	NP_57013	4730156	A	1124	TGGAACGGAGACAGCATTGGGCT		
ILMN_1678757	BCYRN1		2450154	S	57	GGAGGAT	2	+
ILMN_1680279	USP49	NP_06103	7320088	S	2818	TCCTAGCT	6	-
ILMN_1685628	C15orf51		5910131	I	3782	AGCTCACACCACTGCAGTCCAGCC		
ILMN_1688490	PTP4A2	XP_95000	5360603	I	2364	ACTCTGGCCTGGGTGATAGAGCG		
ILMN_1688666	HIST1H2B	NP_00351	5720433	S	352	GCCGTCAC	6	+
ILMN_1690114	PTPLAD2	NP_00101	1470091	S	3665	GAGACAG	9	-
ILMN_1691428	PSMD12	XP_00113	1440131	I	2849	TGCAGTGGATCCCAGCACTTTGGG		
ILMN_1691881	IL4R	NP_00100	1770678	I	962	GGGGAAT	16	+
ILMN_1692145	ZNF14	NP_06635	2340674	S	2358	AGGCCAA	19	-
ILMN_1693789	ALPP	NP_00162	4230088	S	2672	TCTATGCG	2	+
ILMN_1694399	ICA1	NP_07168	6560411	I	543	GGATCAC	7	-
ILMN_1695468	SRPK2	NP_87263	2350072	I	107	CTGGCCAT	7	-
ILMN_1696027	LOC642333		1010296	A	611	GGCCGGGAAGCTAAAAGACAAGT		
ILMN_1697304	FARSLB	NP_00567	460128	S	2992	CAGTCCCA	2	-
ILMN_1697377	LOC64984	XP_94399	4830300	S	644	GGACAGTGTCTTTTGACCAACCAC		
ILMN_1699383	LOC44260	XP_94092	5360300	A	2603	TTTTGGAGGTCATGGCGGGAGGA		
ILMN_1700182	LOC400721		5890162	S	5744	AAGGTCATGCCACTGCACTCCAGC		
ILMN_1709164	RUNDC2B	NP_00101	4780064	I	1140	GAGCTGG	16	+
ILMN_1710362	LOC64459	XP_93279	1510196	S	3719	TGGTGAT	1	-
ILMN_1711073	LOC65348	XP_93920	3890053	I	5975	TCTGTCAC	2	-
ILMN_1713319	PBLD	NP_07141	1340474	I	2137	GGAGTTC	10	-
ILMN_1713706	ZNF786	NP_68962	4200541	S	3091	TGGGAGG	7	-
ILMN_1721204	CSF2RA	XP_94759	5340037	I	1791	CCGTTGCACTCCAGCCTGGGTAAC		
ILMN_1721349	DKFZp564I	NP_11549	5700139	S	2093	GATATGA	X	-
ILMN_1722223	LOC72857	XP_00112	5270575	A	224	AGAATCAC	5	+
ILMN_1726636	HECTD2	NP_77576	2230408	I	1864	ATTGAGTA	10	+
ILMN_1730678	ANKRD30E	NP_00102	1690184	I	2942	CATGCACC	18	+
ILMN_1731203	KIAA1618	NP_06600	4570561	I	4774	CAAGGCA	17	+
ILMN_1732311	CEP152	NP_05580	3930370	S	4067	AGGCAGA	15	-
ILMN_1733983	LOC65331	XP_93821	7320170	I	1162	CAGTGGC	5	+
ILMN_1734991	PPM1B	NP_00102	3450026	I	1448	GTAATCCT	2	+
ILMN_1736008	YRDC	NP_07891	1340491	S	1263	AAGTCTCA	1	-
ILMN_1737878	FLJ34047	NP_77594	4850735	S	2829	TACTCTTG	5	-
ILMN_1738482	CEP27	NP_06056	3850524	S	1256	AGTCATGA	15	+
ILMN_1739283	UPF2	NP_05635	2230307	I	5	GCTGGAG	10	-

ILMN_1740165	C14orf102	NP_06044(	3140358	I	161	GAAGCAG(	14	-
ILMN_1743476	LOC65382!	XP_94089!	7100044	A	3711	GTTTTGGAGGTCATGGTGGGAGG/		
ILMN_1745931	FLJ43663	XP_93563{	3930180	A	1437	AGGACAA(	7	-
ILMN_1746919	LOC64388:	XP_93697:	3180482	S	1616	CTACTCGG	12	+
ILMN_1748090	SLC2A11	NP_00102(	2750091	A	1397	TGTCCCAC	22	+
ILMN_1750093	SDHALP1		6420674	I	10150	GATTGTGCCACCGTACTCCAGCCT(		
ILMN_1755114	EIF2AK4	NP_00101:	3370471	S	5290	ATGGATCA	15	+
ILMN_1756998	LOC64769:	XP_94184:	2970600	S	3	GCCACAAATAGAAAAGTTTTCTGC/		
ILMN_1759872	LOC64350!	XP_93324!	70286	A	2564	GTGGAAT(	18	-
ILMN_1766176	RAP1A	NP_00101(	3060692	I	188	GGTGATTA	1	+
ILMN_1771320	FLJ38717	NP_00100	990100	S	2559	TTGCAGG(	6	+
ILMN_1772492	MCART1	NP_21948(	1980521	S	1556	CCTAGCTG	9	-
ILMN_1776290	LOC65359(	XP_93974	430209	I	6963	GTGATGT(	2	-
ILMN_1778202	FLJ40722	XP_94718!	2600632	I	2233	CTGGCGTTCCACACCAGCCTGGCC/		
ILMN_1778457	IL18	NP_00155:	5890196	S	1038	TGTGGTG/	11	-
ILMN_1780496	MGC1276(	XP_93557:	4490338	I	1659	GCCGTCG(	1	-
ILMN_1783060	SLC25A20	XP_00113:	770255	I	561	GTAAGGTTGTCTACTCAGGAGGCT		
ILMN_1784554	LOC64738!	XP_94155	6040181	S	837	AAATCAAAACTACTATGAGGCCGG		
ILMN_1785703	LMOD3	NP_93801:	4250609	S	3155	CCCTAGAA	3	-
ILMN_1787314	ALS2CR14	NP_83994:	4900215	S	830	TTTTCTTT(	2	-
ILMN_1795461	LOC72985:	XP_00113	5720204	S	1261	AGGGCTT/	1	-
ILMN_1798581	MCM8	NP_87795	6270082	A	3364	CAGTGAG(	20	+
ILMN_1805668	ZNF486	XP_37115:	2070471	A	3074	CATTGCAC	19	+
ILMN_1806999	MGC4093	NP_08505:	270519	S	895	GGGGACA(	19	-
ILMN_1809750	TDRD1	NP_94209(	270301	S	3989	TTTATTAC	10	+
ILMN_1811221	SLC5A8	NP_66601:	4010021	S	3702	GTAATCTG	12	-
ILMN_1812105	PIP5K2B	NP_61963:	6370093	I	1324	TTGAACCC	17	-
ILMN_1812640	AHR	NP_00161:	840189	S	4990	GGCCAAG/	7	+
ILMN_1814039	LOC65308(	XP_93608{	6860064	I	6448	TGATGTGT	2	-
ILMN_1814204	C21orf55	NP_06030:	4390136	S	1982	TTTTTTGA(	21	-
ILMN_1815578	ZNF223	NP_03749:	4760767	S	2321	TGACCTAT	19	+
ILMN_1751351	FLJ90086	XP_37182(	5050181	A	4017	AAAAGAG	6	-
ILMN_1759250	TAP2	NP_06131:	1780528	A	1448	GGTTTTCT	6	-
ILMN_1760509	EOMES	NP_00543:	6760075	S	2643	GTCCAGG/	3	-
ILMN_1773650	LRRN3	NP_06080	7380181	S	2676	GCTGCCTC	7	+
ILMN_1783795	BTNL3	NP_00669:	3180082	A	1714	CTCCATCC	5	+
ILMN_1793461	LOC65360	XP_49771:	730468	S	12	GGACCAT(	1	-
ILMN_1796335	AYTL1	NP_06030!	5670100	S	1697	GACAACC(	16	+
ILMN_1343293	TXN		4260048	S	72	GCAGATCGAGAGCAAGACTGCTTT		
ILMN_1651228	RPS28	NP_00102:	7510672	S	329	CGCCACAC	19	+
ILMN_1651498	GADD45G	NP_00669(	70376	S	923	GCCCTGG/	9	+
ILMN_1651850	RPS16	NP_00101	130768	S	496	GCGCTCG(	19	-
ILMN_1651964	ABCC5	NP_00101:	7610097	I	1804	CCGTCGG/	3	-
ILMN_1652003	GNG10	NP_00101	4590608	S	292	GCCTTCAA	9	+
ILMN_1652128	LMBRD1	NP_06083:	4590301	S	1576	CTGGTTCT	6	-
ILMN_1652230	LOC64736:	XP_94152:	3870088	S	27	CCACGCGCGAAAATTCGGCCAGG(		
ILMN_1652631	C9orf19	NP_07173:	4280253	S	1778	GCAGCTCC	9	+
ILMN_1652736	RPS6KA3	NP_00457	5890647	I	7247	GTACTTAT	X	-

ILMN_1653567	LOC39003:	XP_37234!	2140537	S	108	TGGAACTC		11 -
ILMN_1653871	PBEF1	NP_00573	3800243	I	1544	CGTCTTCAAGGACCCAGTTGCTGA		
ILMN_1654516	TMPIT	NP_11413	5560577	S	1055	CCACAAGT		7 -
ILMN_1656057	PLAU	NP_00264!	1740349	S	2049	GTGGTCTT		10 +
ILMN_1656676	ZYG11B	NP_07892:	4610019	S	6333	GGAAGTTC		1 +
ILMN_1656961	CD82	NP_00222:	6770594	A	1323	GGCGCAG		11 +
ILMN_1657317	POLR2J	NP_00622:	4810594	S	627	TGTGGACT		7 -
ILMN_1657398	LOC28475	NP_00100	870349	S	4094	CTGGTAGC		20 +
ILMN_1657497	KIAA0319	NP_05562	5910102	S	6659	CAGCCTGT		6 -
ILMN_1657515	RPS6KA5	NP_00474	2030482	I	3521	GGGGCATC		14 -
ILMN_1657996	LOC64203!	XP_94119	360128	S	3	GTCTTCACATCTACCTTTCTATGTC		
ILMN_1658798	MEGF9	NP_00107:	7560615	A	857	GGCTCTGTCAGCCATGTGACTGTA		
ILMN_1658957	LOC653635		3990128	S	2595	GGGGGCT		1 -
ILMN_1659047	HIST2H2A	NP_00350	1030039	S	1	CGACTTTC		1 -
ILMN_1659437	TXNL5	NP_11612	830047	S	378	CACCTCAA		17 +
ILMN_1659490	LOC65315	XP_93190	5820682	I	2213	GCTACTCA		5 +
ILMN_1660357	LOC64182!	XP_94066	3180246	S	121	GGGAGGAAGGAACGAGGCAAGG		
ILMN_1660498	RPS27	NP_00102	5290523	S	36	CACGAGA		1 +
ILMN_1660549	GPR177	NP_00100:	540288	I	1848	TGTTCAGC		1 -
ILMN_1661196	CSF2RA	NP_75844!	4570168	A	1147	ATTCACCC	X	+
ILMN_1661461	LOC28354	XP_37845	870575	A	4193	GCCTCATG		14 -
ILMN_1661466	LOC64642	XP_94636	520209	S	189	CAGTGACCAGGTTACCTACAGTAG		
ILMN_1661636	ZMYM2	NP_00344	6040079	A	4512	CAAGTTTA		13 +
ILMN_1662617	PPP2R3C	NP_06038	2760110	S	1264	GCTACCAT		14 -
ILMN_1662846	GPR160	NP_05518	6180427	S	1437	TGCCCCCT		3 +
ILMN_1662865	FLJ11151	NP_06081	7550519	S	2496	CAGGTGT		16 -
ILMN_1663422	Rgr	NP_70584:	3440253	S	2648	GCTCTGCA		22 +
ILMN_1663437	EFCBP2	NP_06193	6770669	S	1468	CCACGCAT		16 +
ILMN_1664047	CACNA1E	NP_00071:	2760452	S	9561	CACTGCAC		1 +
ILMN_1664577	DLD	NP_00009!	1690671	S	1675	CCTGGGAC		7 +
ILMN_1664698	UNC119	NP_00513!	1570164	A	1267	CATGAGGC		17 -
ILMN_1664706	LOC65360	XP_49771:	6650593	S	133	ATGGCCCC		1 -
ILMN_1664750	TMBIM4	NP_05714	5270356	S	610	GGAGTTGGTCTTAGCCGCTGCAGC		
ILMN_1664802	WSB1	NP_05644	6650575	A	2671	CTGTTTCT		17 +
ILMN_1665384	SH3BP5L	NP_08514	7320112	S	2889	AGATCTGC		1 -
ILMN_1665557	USP15	NP_00630	70767	S	4159	GCTGGGG		12 +
ILMN_1665781	LOC39046	XP_37252:	6130672	S	61	AACCACCC		14 +
ILMN_1665982	AKTIP	NP_07192	2360414	A	1431	ATGTTAGC		16 -
ILMN_1666376	TRIM56	NP_11222:	1780647	S	3115	GGGCCAA		7 +
ILMN_1666480	CDC123	NP_00601	6110379	S	1191	ACCGGCTA		10 +
ILMN_1667162	NKX3-1	NP_00615	4670735	S	2969	CCATCTTG		8 -
ILMN_1667319	LPPR2	NP_07357	3140274	S	2354	GCTTGCCC		19 +
ILMN_1667460	SULF2	NP_94099	5270762	A	3335	CAGGACAC		20 -
ILMN_1667561	IFRD1	NP_00154	3780243	A	1957	GACAAGAC		7 +
ILMN_1667970	LOC72882!	XP_00112	3060348	S	35	TGCGCGC	X	-
ILMN_1667985	NTNG2	NP_11592	6270382	S	2101	GGACCCCC		9 +
ILMN_1668379	LOC39147!	XP_94237	2630253	A	601	CCTGCTCGGTGCACTCTCTGCAGG		
ILMN_1669177	DHRS12	NP_00102	4890114	A	562	ACCCGGCC		13 -

ILMN_1669608	PDE7A	NP_00259	6960221	I	2530	GCTGAATC	8	-
ILMN_1669790	MNDA	NP_00242	6380228	S	1318	AACTGAGA	1	+
ILMN_1670130	ARID3A	NP_00521	4260446	S	2546	CCACACACTCACCACTCCCAGCTTC		
ILMN_1670821	CYorf15A	NP_00100	2190192	S	1688	TTGATGCA Y		+
ILMN_1671516	CIR	NP_00487	2640088	A	1294	AGGGAGA	2	-
ILMN_1671933	CLCC1	NP_00104	20674	S	4235	CTTCTCTGGACATGCGCGTTTGAG		
ILMN_1672004	TOB1	NP_00574	2470477	S	892	CCAATGGA	17	-
ILMN_1672988	LMNB1	NP_00556	4900048	S	2443	GGGAGGG	5	+
ILMN_1673113	F2RL1	NP_00523	5690201	S	2245	CAGACTGC	5	+
ILMN_1674590	LOC65257	XP_94719	3180192	S	537	GAGATGATGTGTGGTCCCACTGAA		
ILMN_1674650	C9orf95	NP_06035	7320079	S	1013	CTCCCTCTC	9	-
ILMN_1675117	HSD17B11	NP_05732	2190475	I	465	ATGCTGGT	4	-
ILMN_1675424	SIRPB1	NP_00605	1570402	S	3692	CTCCTCTC	20	-
ILMN_1675939	IFNGR1	NP_00040	1500446	S	1771	CGGAACAT	6	-
ILMN_1676016	NBPF9	XP_93911	6270324	I	582	TATTTCTG	1	-
ILMN_1676480	LOC65405	XP_94877	7400592	S	941	TGGGATTCGGCCTCTGGAAAGTGC		
ILMN_1676737	Septin 2	NP_00100	780431	I	352	TTTTTTGTC	2	+
ILMN_1676767	CLEC4D	NP_52512	3990328	S	1551	GCCAAAGC	12	+
ILMN_1677511	PTGS2	NP_00095	1470682	S	3943	GCCTATGT	1	-
ILMN_1678170	MME	NP_00921	240608	A	5051	GAGTCTGCCTCCATGCTGCAGTGT		
ILMN_1678690	UPB1	NP_05741	7160367	S	1915	GAAATTCT	22	+
ILMN_1678707	TAF15	NP_63196	5960128	A	1913	TTGCCAGA	17	+
ILMN_1678799	RAPGEF1	NP_94137	6100576	I	65	AAGAGATA	9	-
ILMN_1678939	VNN2	NP_51104	5570673	A	1770	CTCCAGCT	6	-
ILMN_1679666	SCGB3A1	NP_44309	2060543	S	221	ATCCCCGT	5	-
ILMN_1679920	LOC65189	XP_94624	1570750	S	7	TTGGGCTTCACCCATAACCCACTGC		
ILMN_1680314	TXN	NP_00332	830762	S	43	CGTCAGAC	9	-
ILMN_1681130	ACP6	NP_05744	3180376	S	1595	TGCCGAGA	1	-
ILMN_1681683	LOC65091	XP_94951	4250543	S	1385	GTAGTCTGCAGATGTGGATCCTAA		
ILMN_1681888	PRKAR2A	NP_00414	4900593	S	2304	GGGTCTGC	3	-
ILMN_1682165	NT5C2	NP_03636	520647	S	3272	CTTCCATT	10	-
ILMN_1682443	UBE2D3	NP_87161	4640110	A	2052	TGGTGTGC	4	-
ILMN_1682699	PBX2	NP_00257	5550500	S	3106	GGGAGGCATGGCCGGGGGCAGT		
ILMN_1682757	PFKFB2	NP_00620	3190326	I	6815	GGGCTGAC	1	+
ILMN_1683250	LOC44073	XP_93878	270768	A	111	TGGCTAGA	1	-
ILMN_1683494	TMEM154	NP_68989	7650379	S	2498	TCCAACCC	4	-
ILMN_1683959	THRAP2	NP_05615	1990332	S	9174	GGGGAAA	12	-
ILMN_1684054	ASAH1	NP_80859	840161	I	261	GCTCTTCTTTGCCTCTGCTGGAGTC		
ILMN_1684585	ACSL1	NP_00198	4880717	S	3553	GGGGTCTC	4	-
ILMN_1685312	PSG11	NP_00277	3400292	I	413	TTCCAATG	19	-
ILMN_1685493	TMCC3	NP_06574	2650152	S	4505	GGGCAAA	12	-
ILMN_1686116	THBS1	NP_00323	5910376	S	4947	CAGTCTAA	15	+
ILMN_1686464	SLCO4C1	NP_85132	770717	S	4454	CTCCCACC	5	-
ILMN_1686750	MGEA5	NP_03634	1010402	S	4955	CACAGGTC	10	-
ILMN_1686862	HLX1	NP_06877	1400706	S	1612	CACGGAGC	1	+
ILMN_1686981	SULF2	NP_06132	1850056	I	2862	GTTTCAGC	20	-
ILMN_1687057	RBM7	NP_05717	5390326	S	1656	AGTGCCCA	11	+
ILMN_1687384	IFI6	NP_00202	4390176	A	743	TGCGCCGA	1	-

ILMN_1687805	LOC65189	XP_94624	5130273	S	131	TGGAATTTGCAAAGCTGCCAAAGC		
ILMN_1688580	CAMP	NP_00433	6400736	S	644	AGAGTCCT	3	+
ILMN_1689004	TNFRSF12	NP_05772	2190008	S	634	CAGAAAG	16	+
ILMN_1689955	C19orf40	NP_68947	7150189	S	676	GGAGCAG	19	+
ILMN_1691364	STAT1	NP_64467	3190133	I	2595	CGCCATCA	2	-
ILMN_1691508	PLAUR	NP_00100	6130594	I	1107	TCAGCCAT	19	-
ILMN_1691574	LOC64606	XP_93846	2450014	S	1382	TGTATAGT	16	-
ILMN_1693394	BCKDK	NP_00587	5390056	S	1742	AGCACCAG	16	+
ILMN_1693494	CMTM1	NP_85181	4830017	A	704	CACCCCCG	16	+
ILMN_1693740	LOC64783	XP_94814	2600669	S	2498	TAAGATCATGTCACTGCACTCCAG		
ILMN_1694075	GADD45A	NP_00191	3140239	S	659	CTGCACTG	1	+
ILMN_1694584	CLTCL1	NP_00902	5090026	I	4593	GTTCAGGTGCATTGCGGCCTATCT		
ILMN_1694742	RPS29	NP_00102	1990753	A	63	GCGAAAA	14	-
ILMN_1695435	LOC65361	XP_93348	5670544	S	713	ATGCTGG	1	+
ILMN_1695853	CLK4	NP_06571	6510110	S	2086	GGGTGAT	5	-
ILMN_1696162	MME	NP_00089	2360400	I	28	AGATGTGCAAGTGGCGAAGCTTG		
ILMN_1696187	PYGL	NP_00285	4610113	S	2494	ATCCAAAG	14	-
ILMN_1696466	ROPN1L	NP_11412	1050240	S	740	GGGCCCG	5	+
ILMN_1696704	TLE4	NP_00893	6290170	S	4048	GGCATGCT	9	+
ILMN_1698213	RBM3	NP_00101	7380612	A	346	GAGGTGG	X	+
ILMN_1699015	LOC440926		4590593	A	143	CGTACAAAGCAGACTGCCCGCAAA		
ILMN_1699331	IFIT1	NP_00153	6220673	I	108	GATGCAG	10	+
ILMN_1699687	USP9Y	NP_00464	6290398	S	9257	GTGTAGCC	Y	+
ILMN_1700610	CLEC7A	NP_92294	1090170	A	43	CCTAGCCC	12	-
ILMN_1700896	SAP30	NP_00385	2510133	S	873	CAGATCTC	4	+
ILMN_1701242	UTY	NP_87260	5310196	I	4730	TGCCTTCT	Y	-
ILMN_1701747	PFN2	NP_44425	4210246	I	620	GGTGATG	3	-
ILMN_1701957	MXD1	NP_00234	2260239	S	5318	TTTTGGGG	2	+
ILMN_1702168	HSD17B12	NP_05722	3290411	S	1747	GTACCACT	11	+
ILMN_1703720	SF3B14	NP_05713	1410315	S	199	TTCATCAG	2	-
ILMN_1704385	LOC34737	XP_94302	5900072	A	1	ATACCATGGCTCGTACAAGGTGGA		
ILMN_1704476	LOC28315	NP_00102	4810324	A	314	GCATGCCA	11	-
ILMN_1705477	CAMK1D	NP_06513	3930653	I	1347	GCATCTGC	10	+
ILMN_1705605	LOC653635		2480278	S	2417	ACACCAGC	1	-
ILMN_1706502	EIF2AK2	NP_00275	1190349	S	2728	CGTTCTCT	2	-
ILMN_1708485	BIN3	NP_06115	7550392	S	1578	TTGTGCTGTAACTTAGGCGAGCAG		
ILMN_1708841	GOLPH3	NP_07141	4780114	S	2120	GGGAAGA	5	-
ILMN_1708936	EXOSC3	NP_00100	1980743	A	721	CAGGTCAC	9	-
ILMN_1709707	LOC64749	XP_94803	6560128	I	589	AGGCCCTTTGCCTTCACAGAGAAG		
ILMN_1710974	RGS2	NP_00291	5690500	S	865	CCTCACTG	1	+
ILMN_1711156	LOC64589	XP_93395	7570673	S	83	TGTGGCGC	8	-
ILMN_1711617	GMFG	NP_00486	4250452	S	469	TCCTCAGG	19	-
ILMN_1711729	LOC64496	XP_93314	2600187	S	1	CAAAATGC	X	-
ILMN_1712389	CKLF	NP_05741	2000551	A	174	ACATCGCC	16	+
ILMN_1712596	NUDT16P		6420402	S	1354	AGAGCCTC	3	+
ILMN_1712688	LOC65350	XP_93286	160494	S	3717	TGGTGATC	1	-
ILMN_1712918	NQO2	NP_00089	870400	S	621	CGGGAGG	6	+
ILMN_1713636	S100A6	NP_05543	1500553	S	527	GGAGTATC	1	-

ILMN_1714065	FAM101B	XP_00112	2350093	S	5	CTCCCACCAGCCTCTTAACTACTTG		
ILMN_1714418	FAM101B	NP_87436	940246	S	292	CCTGGCTCCCGGCTGTGCGCTGAG		
ILMN_1714848	ZNF354A	NP_00564	2850670	S	2312	TGTCACGT	5	-
ILMN_1714991	S100A9	NP_00295	830400	S	506	GCCTGTTATGTCAAACTGTCTTGG		
ILMN_1715693	LOC44016	XP_49857	1190731	S	115	TCCTGCAGCTGTGTTTGAACAGGT		
ILMN_1715809	OGFRL1	NP_07885	2750315	S	1038	AAAGGAG	6	+
ILMN_1716465	RBP7	NP_44319	4810131	S	421	GTGCAAA	1	+
ILMN_1717371	C22orf34	NP_00103	5910682	S	1303	TGCTTTCT	22	-
ILMN_1718303	PVRL2	NP_00284	2570544	S	1789	CTGGACGGCTCCCTCATCTCACGG		
ILMN_1719951	FLJ31951	NP_65332	2490437	S	2719	GGGTGGG	5	-
ILMN_1719988	HAL	NP_00209	6660327	S	2301	GGGCAGT	12	-
ILMN_1720604	ARSG	NP_05577	5420379	S	1739	GTTAGCCT	17	+
ILMN_1721116	USP10	NP_00514	1980021	S	2458	GATCAACCAGTACCAGGTGGTGA		
ILMN_1721648	EXOC8	NP_78707	5130491	S	4522	GGAACTT	1	-
ILMN_1721729	PPARBP	NP_00476	6760373	S	5590	GGGTTCA	17	-
ILMN_1722076	DHRS8	XP_00113	1570553	A	1604	CCTACCCATTGCCACTCTGTTTCCT		
ILMN_1723079	LTB4R	NP_85804	3120735	S	2942	TGAGAGC	14	+
ILMN_1723320	FLJ37307		5490440	A	3986	GTCTTCCT	13	-
ILMN_1723486	HK2	NP_00018	4760768	S	6532	GAGCACA	2	+
ILMN_1723909	LOC40217	NP_00101	4050056	S	1350	ACTGCATC	4	-
ILMN_1724315	LMTK2	NP_05573	4050040	S	5327	GGATCTTC	7	+
ILMN_1724407	TACC3	NP_00633	2630544	S	2550	GGAGCAG	4	+
ILMN_1724533	LY96	NP_05617	6980474	S	505	TGGGAGC	8	+
ILMN_1725427	B2M	NP_00403	6290114	S	402	GTGGGAT	15	+
ILMN_1726289	C12orf35	NP_06063	3310446	S	5367	ACGGAAG	12	+
ILMN_1726786	TNRC6B	NP_00102	2600386	A	15415	CTAGGCA	22	+
ILMN_1727603	ZKSCAN1	NP_00343	1580187	S	5035	ATCCTCAG	7	+
ILMN_1728639	FCGR3B	NP_00056	3990360	S	1450	GAGCCCTC	1	-
ILMN_1728677	CREB5	NP_00101	4220026	I	40	GAGCTCTC	7	+
ILMN_1728724	IL17RA	NP_05515	3870110	S	2932	CAGGGGA	22	+
ILMN_1728844	PTPRN2	NP_57085	6580717	A	4200	CCGATTCG	7	-
ILMN_1729294	RNF130	NP_06090	5670603	S	1503	CCTCAGGA	5	-
ILMN_1729421	LOC64975	XP_94705	70041	A	190	GGGGTAGAAGTTATGCTTGTGTTT		
ILMN_1729659	TNNI2	NP_00327	6350142	S	274	GTGAGGG	11	+
ILMN_1729801	S100A8	NP_00295	6280576	S	253	TAACTTCC	1	-
ILMN_1730454	FOLR3	NP_00079	2230601	S	759	GCCCCGTC	11	+
ILMN_1730928	CDK5R1	NP_00387	770484	S	3133	CCCTCAGT	17	+
ILMN_1731048	TLR1	NP_00325	5080398	S	2736	GTGAGTG	4	-
ILMN_1731546	RPL26	NP_00097	2000025	S	57	CTTCCCTT	17	-
ILMN_1731785	ORM2	NP_00059	6370202	S	473	GCTGTTCC	9	+
ILMN_1735014	KLF6	NP_00129	5260397	I	1330	CCCTTCCG	10	-
ILMN_1735506	CYSLTR1	NP_00663	4810204	S	1218	TGCAGAA	X	-
ILMN_1735955	LOC64403	XP_93237	4290669	S	283	CAAACCTG	8	-
ILMN_1736190	CYP4F3	NP_00088	650164	S	4625	CTATCTAT	19	+
ILMN_1736982	PHACTR1	NP_11221	3800095	S	1803	AGATAAG	6	+
ILMN_1737426	PCMTD1	NP_44316	240037	S	3666	TCTAGCAT	8	-
ILMN_1738243	RPS29	NP_00102	5960026	I	194	TGGACTAA	14	-
ILMN_1738643	LOC64496	XP_93313	70221	S	430	CTATGCCA	19	-

ILMN_1738821	GOLGA2	NP_00447	5390445	S	3921	CCATCTTCT	9	-
ILMN_1739263	LOC64328	XP_93327	4230615	S	68	GGCCAGG	9	+
ILMN_1739423	RN7SK		5080673	S	257	CGTAGGGT	6	+
ILMN_1739587	UTY	NP_00905	5420224	I	6354	GCTATACT	Y	-
ILMN_1739622	PPP1R12A	NP_00247	4180280	S	4479	ACATCTTT	12	-
ILMN_1739876	RAB3GAP1	NP_03636	6330561	S	3989	CATGGAG	2	+
ILMN_1741073	FCAR	NP_57981	6860239	I	1361	CTGCACAC	19	+
ILMN_1741917	OSCAR	NP_57012	2190609	A	983	AACGGTCC	19	-
ILMN_1743034	KIF1B	NP_90432	3170719	I	5646	GCCCAGGT	1	+
ILMN_1743281	PHF3	NP_05596	2510687	S	6079	GGAGAAA	6	+
ILMN_1746020	MDM4	NP_00238	4490671	S	2032	CTTCTGCC	1	+
ILMN_1746276	EPC1	NP_07948	2680010	S	2756	ACACAGTA	10	-
ILMN_1746577	RAE1	NP_00360	4850138	A	1662	CTAAGGG	20	+
ILMN_1746864	PSCDBP	NP_00427	5390195	S	1719	GGGGTTA	2	-
ILMN_1747047	ZNF346	NP_03641	3420373	S	2662	GTGTCCAG	5	+
ILMN_1747205	JDP2	NP_56973	3180379	S	1608	GCCAGGG	14	+
ILMN_1748366	SLC5A9	NP_00101	70270	S	2889	CTGTATTT	1	+
ILMN_1748767	LOC73074	XP_00112	4920408	S	228	TGTGGCGCTCCGTGAAATTAGACG		
ILMN_1748915	S100A12	NP_00561	10279	S	241	TCCAAGGC	1	-
ILMN_1749006	RCSD1	NP_44309	3140280	S	2692	TGCAAGGC	1	+
ILMN_1749673	CSF2RA	NP_75844	4150246	A	1244	CCGCCTCC	X	+
ILMN_1750880	AMN1	NP_99722	3990719	S	1629	CCACGCTT	12	-
ILMN_1750961	TM6SF1	NP_07537	10541	S	964	GGAGGTCT	15	+
ILMN_1751051	C7orf25	NP_07695	4570397	S	1351	CAGCCCAG	7	-
ILMN_1751749	SYNJ2	NP_00388	5900592	S	6404	CAAAGAA	6	+
ILMN_1753712	STX10	NP_00375	3190681	S	685	TGGTGTGC	19	-
ILMN_1753819	RFFL	NP_00101	5870551	A	3411	GGGCAGA	17	-
ILMN_1754395	ADCK4	NP_07915	1690221	S	1907	GGAACTCC	19	-
ILMN_1754859	TRIM25	NP_00507	2850576	S	5462	CAGCCTGA	17	-
ILMN_1755049	ANKRD44	NP_71018	4730377	S	2988	CCTACAAG	2	-
ILMN_1755077	HEBP2	NP_05513	5090184	S	804	CGATGAG	6	+
ILMN_1755115	RPL23	NP_00096	4120707	S	321	TCCAGCAG	17	-
ILMN_1756506	CYorf15B	NP_11596	4730458	S	4976	AGCAGTG	Y	+
ILMN_1758418	TNFSF13B	NP_00656	460608	S	634	GGCAACTC	13	+
ILMN_1758658	FADD	NP_00381	1940035	S	1558	CTCTGAGA	11	+
ILMN_1759208	RGS18	NP_57013	1240438	S	1787	GGGGAAG	1	+
ILMN_1759613	PNMA6A	NP_11627	6760270	S	1892	GATGGCG	X	+
ILMN_1760347	SRGN	NP_00271	650541	S	482	CCTTAGGT	10	+
ILMN_1760542	PSMA1	NP_68387	20451	I	233	CCTTGGCC	11	-
ILMN_1760779	ENSA	NP_99705	1110685	A	236	GCCTGAG	1	-
ILMN_1761411	C10orf119	NP_07911	4830192	S	4030	CAGCGTTA	10	-
ILMN_1763260	HIF1A	NP_00152	2850288	I	2496	TTACAGCA	14	+
ILMN_1763408	RBMXL1	NP_06255	5080093	S	3210	CTCCCCGT	1	-
ILMN_1763452	EVI2B	NP_00648	2030593	S	1401	CCTCCCAA	17	-
ILMN_1763809	IRS2	NP_00374	6980095	S	6369	GTAACTCC	13	-
ILMN_1763837	ANPEP	NP_00114	1770605	S	3149	CTCCAGCC	15	-
ILMN_1764082	MBOAT1	XP_00113	5810731	A	2451	GAGCTGAAGAGGGGCTGCTGTTT		
ILMN_1764396	HDAC4	NP_00602	5960341	S	8280	GGTGATGT	2	-

ILMN_1764415	ZNF585A	NP_68986	460504	I	5	CGGAAGT(	19	-
ILMN_1765061	OXER1	NP_68376	510491	S	1501	GTGGGTGGCAGGGAGAGAAACCC		
ILMN_1765122	MAP3K2	NP_00660	2320296	A	3192	GCAGCCTC	2	-
ILMN_1767809	LOC55289	NP_00411	5820176	S	337	AACCGCCT	9	+
ILMN_1768117	RBM25	NP_06706	840075	S	4100	ACACACCT	14	+
ILMN_1768394	ARPC5	NP_00570	3930243	S	1767	CGAGTAG(	1	-
ILMN_1769705	LOC44009	NP_00101	4730100	S	307	TTCAACAC	12	-
ILMN_1769734	NT5C3	NP_00100	3780689	A	962	ACTCCCAA	7	-
ILMN_1769741	RPL32	NP_00100	2340373	I	1	GACCTCCT	3	-
ILMN_1769764	ANKRD55	NP_07894	4670056	S	1969	CTGCCAAC	5	-
ILMN_1769937	LOC64982	XP_94730	5550364	A	192	GGCCTTCTCACCTAACGCCACCAA(		
ILMN_1770161	BST1	NP_00432	4920243	S	1081	GTAACTGG	4	+
ILMN_1770244	CBX1	NP_00679	2450446	S	1745	AGAGGAA	17	-
ILMN_1770831	LOC64197	XP_94084	6040114	S	7	CTAATAACAGTGACCTCCCCGCCA(		
ILMN_1771048	LOC65315	XP_93190	3460735	A	194	TATAAGAT	5	+
ILMN_1771664	CLEC4E	NP_05517	940754	S	1426	GGCTGTA/	12	-
ILMN_1771815	C20orf43	NP_05749	4050537	S	1458	TCCCTCTG	20	+
ILMN_1772163	PRKY	NP_00275	2900048	S	6641	ATCTGGCC	Y	+
ILMN_1772387	TLR2	NP_00325	1260008	S	3246	GCCACAA/	4	+
ILMN_1773154	NFKBIA	NP_06539	4570154	S	992	GAGGACG.	14	-
ILMN_1773757	SLC26A8	NP_44319	6650717	A	2874	GCAGCGTT	6	-
ILMN_1775268	HECW2	NP_06581	3140541	S	6706	CTGGGAC(	2	-
ILMN_1775444	FLJ12078		7150039	A	2413	GGGGTGAAGTGGAAAATCCAGGG		
ILMN_1776195	TMSB4Y	NP_00419	4150095	S	1360	AGTGGCG	Y	+
ILMN_1776515	MPPE1	NP_07556	780168	A	2114	TCGTCACTCACATGCACGACAGTC(		
ILMN_1776649	LRRK2	NP_94098	1450523	A	7502	CACAGCTA	12	+
ILMN_1777565	TAP2	NP_00053	2970240	I	5257	ACTTGCCT	6	-
ILMN_1778709	PICALM	NP_00909	1580364	I	1560	CTGTAGAT	11	-
ILMN_1778930	CHD7	NP_06025	4640750	S	10019	TGTCCCGA	8	+
ILMN_1779228	CDH2	NP_00178	4780376	S	4032	GCCTCTGT	18	-
ILMN_1779572	LOC44045	XP_94027	7510424	I	895	GTCCATCA	17	-
ILMN_1781198	PPP1R3D	NP_00623	6400064	S	3031	GGCCTTCA	20	-
ILMN_1782459	OSBPL8	NP_00100	3830138	A	6974	CCTGCAGT	12	-
ILMN_1783085	IL8RB	NP_00154	2710437	S	2541	GGAGGGG	2	+
ILMN_1783675	ASB8	NP_07700	4280114	S	2124	GGCAGTT(	12	-
ILMN_1783805	PNMA3	NP_03749	2490113	S	3474	CGGGACCTGTGTGGGACCTGTGT(		
ILMN_1784300	TUBA4A	NP_00599	380731	S	1391	GAGGGAG	2	-
ILMN_1784380	DTX3L	NP_61214	2850100	S	4588	GGGATTA(	3	+
ILMN_1785167	OSBPL9	NP_07886	6020685	A	2397	GCTTCCCT	1	+
ILMN_1785345	GPR84	NP_06510	2370576	S	1181	GCATCAAC	12	-
ILMN_1785615	SUMO1P1		5340050	S	447	GACTTTCCAGTTGGCCCTGATTTTC		
ILMN_1786847	TGM3	NP_00323	3440754	S	2485	TGATCCCC	20	+
ILMN_1787186	NOV	NP_00250	6420367	S	1974	TGAGGCC(	8	+
ILMN_1787378	ADD3	NP_05843	1690273	I	2132	GATGACG(	10	+
ILMN_1787808	CEP63	NP_07945	1090300	S	2450	GGAAGAGGAGGAACTGAGGTCTC		
ILMN_1787897	CXCL1	NP_00150	6130576	S	792	GGCTGGC(	4	+
ILMN_1788143	LINS1	NP_00103	730491	A	1016	GAAACCATCTTGCATGCTAGAAGT		
ILMN_1788439	LOC44119	NP_00101	2060113	S	1600	GAAAACC(	7	-

ILMN_1788701	PSIP1	NP_15009	1240541	I	3023	TGAATATT		9	-
ILMN_1790412	FLJ39639	XP_37093	5420452	A	2341	GCTTTACG		16	+
ILMN_1790625	CBX3	NP_05767	4880020	I	37	TCCCCCTT		7	+
ILMN_1790689	CRISPLD2	NP_11366	6060224	S	4224	ACGGCGT		16	+
ILMN_1790715	LOC65310	XP_93630	3800139	I	1	TGCCGACT		1	+
ILMN_1790891	CKAP4	NP_00681	6770348	S	2458	CTGCCTCC		12	-
ILMN_1790985	dJ341D10.	NP_00100	4180402	S	1152	TCAGGGT	X		+
ILMN_1791253	FBXL13	NP_65946	5050653	S	2503	CCCTCCAC		7	-
ILMN_1791536	BTAF1	NP_00396	1820546	S	6646	CTGGGCA		10	+
ILMN_1792207	MLX	NP_73375	5820021	A	2409	GAGATGT		17	+
ILMN_1792997	NPTN	NP_03656	5360338	A	1992	GGGCTTA		15	-
ILMN_1794223	COX7B	NP_00185	70722	S	141	TCAGCAA	X		+
ILMN_1794333	POU2F1	NP_00268	3850554	S	2380	TCCAAGG		1	+
ILMN_1796138	BMX	NP_00171	5560451	A	1987	ACAGGCT	X		+
ILMN_1796316	MMP9	NP_00498	4150224	S	2049	GCTTCTAC		20	+
ILMN_1796642	NCF2	NP_00042	7050360	S	2056	GGGGAGA		1	-
ILMN_1797207	TTTY15		2570082	S	4933	GGCTGCA	Y		+
ILMN_1797745	MAP4K4	NP_66371	2450170	A	6802	GCAGGGA		2	+
ILMN_1797875	ALOX5AP	NP_00162	4180411	S	664	TCTATTGG		13	+
ILMN_1798233	PSMB9	NP_68375	6130669	A	767	TGGGAAA		6	+
ILMN_1798804	SRPK1	NP_00312	3460674	S	4153	GGCAGCT		6	-
ILMN_1799030	CMTM2	NP_65327	5080300	S	763	CCACTTCA		16	+
ILMN_1799106	MOSC1	NP_07358	1980082	S	1680	GGACCCC		1	+
ILMN_1799644	IBRDC2	NP_87743	5910037	S	4431	GTTGTGA		6	+
ILMN_1799848	ANKRD22	NP_65319	4200543	S	609	CCTCTGCT		10	-
ILMN_1800638	CUGBP2	NP_00102	6110672	I	2065	CCCGGTG		10	+
ILMN_1800695	LOC64822	XP_94401	3780189	S	2404	TAAGTTAGCCCTTTGGGTAAGAGT			
ILMN_1801216	S100P	NP_00597	2640609	S	378	AATGATG		4	+
ILMN_1801776	PSG9	NP_00277	1050465	S	404	TCCAACG		19	-
ILMN_1801866	BRWD1	NP_38750	430400	I	12653	AAAGGCT		21	-
ILMN_1802096	ABTB1	NP_74202	5670424	A	1913	CCATTCAG		3	+
ILMN_1802348	SLC36A4	NP_68952	2350195	S	2320	GGAAAAG		11	-
ILMN_1802758	ZNF281	NP_03661	6940113	S	2768	GACACCC		1	-
ILMN_1802799	C1orf108	NP_07887	1690673	A	1613	CAGGCCC		1	+
ILMN_1803500	ANP32A	NP_00629	3360546	S	987	TCTGATTG		15	-
ILMN_1803953	LOC38878	XP_94504	1440259	A	219	GGCGAAGTCGTCAGACCAAAAGG			
ILMN_1805064	SCARNA9		840639	S	46	GCCAAAT		11	+
ILMN_1805104	ABAT	NP_06573	1030685	A	4303	AACTCAGCAGAAGCTGGTAAAA			
ILMN_1806122	CHD8	NP_06597	5310433	S	7016	GGCCCCATTGCTTGGGCGGCTGCT			
ILMN_1806224	KIAA0492	XP_94938	1090609	A	5727	GGCCCTATACCTGATCACCTCTAA			
ILMN_1807003	ANXA11	NP_66587	7380450	I	338	TCACAGTT		10	-
ILMN_1807529	PADI4	NP_03651	5310653	S	2163	GTCCCAGT		1	+
ILMN_1809585	KIAA0319L	NP_87262	150392	I	5065	GGACAGG		1	-
ILMN_1810229	ARID4A	NP_07537	3830228	A	4318	GCCAAAGTATATGTTCAGCAGTGT			
ILMN_1811330	FLJ20152	NP_06187	1230022	S	2592	AGGTACA		5	-
ILMN_1811608	FLJ14107		2900008	S	1503	ACACTCAGCACCCCTCCCAAAGAG			
ILMN_1811616	KIAA1706	NP_08513	6250685	S	4375	CCCAGGA		7	+
ILMN_1812571	RAB35	NP_00685	5340243	S	2738	GTGGGGA		12	-

ILMN_1812856	ZSWIM1	NP_54217(	1260082	S	2094	CTGACTCA	20	+
ILMN_1813139	ANKDD1A	NP_87436:	3290296	S	2807	TCGGGGAAACTGTGTGTGCTGAA(		
ILMN_1813455	SP110	NP_53634!	10546	I	1934	CTTGAAGG	2	-
ILMN_1813746	CORO2A	NP_00338(	4590554	A	1976	CGCTGAAA	9	-
ILMN_1814153	IL9R	NP_00217	290424	A	1990	CTCTGAGA X		+
ILMN_1815121	PLAGL1	NP_00107	2230475	A	2085	CCATCCCTCATGTGTTCTCAGCTGG		
ILMN_1815668	GTF2IP1		6200438	S	2314	CAGCTGGT	7	-
ILMN_2038776	TXN		7610747	S	23	CATCGGTCCTTACAGCCGCTCGTCA		

Probe_Coc	Definition	Ontology_	Ontology_	Ontology_	Synonyms	GB_ACC	group	moduleColors
3720269-3	Homo sapi	nucleus [g	regulation	sequence-s	QRX; MGC	NM_03275	mRNA	blue
47980580-	PREDICTED: Homo sapiens hypothetical protein LOC64					XM_92919	mRNA	blue
74340137-	PREDICTED: Homo sapiens region containing similar to					XM_93351	lncRNA	blue
13734886(	Homo sapi	protein ph	protein am	protein ph	PR72; PPP2	NM_18189	mRNA	blue
24574866	PREDICTED: Homo sapiens hypothetical LOC644852, tr					XM_93421	mRNA	blue
AAACCAGC	PREDICTED: Homo sapiens p21 (CDKN1A)-activated kin					XM_93651	mRNA	blue
28306729-	PREDICTED: Homo sapiens similar to eukaryotic transla					XM_93431	mRNA	blue
23075901(	Homo sapi	nucleus [g	regulation	zinc ion bir	FLJ22835;	NM_00451	mRNA	blue
97394601-	Homo sapiens hypothetical gene supported by BC0449					NM_00101	mRNA	blue
53295365-	Homo sapiens CUE domain containing 1 (CU				DKFZp547I	NM_01794	mRNA	blue
CCTATGAA	PREDICTED: Homo sapiens similar to 60S ribosomal prc					XR_01600	mRNA	blue
GACTGTGG	Homo sapi	membrane	autophagy	GTP binding [goid 552		NM_13078	mRNA	blue
47416014-	Homo sapiens brain cytoplasmic RNA 1, Bc1				BC200a; B(	NR_00156	mRNA	blue
41873670-	Homo sapiens ubiquit	ubiquitin-c		zinc ion bir	MGC20741	NM_0185(	mRNA	blue
TGGGTGAC	Homo sapiens chromosome 15 open readin				DKFZp434I	NR_00326	mRNA	blue
AGACCCTG	PREDICTED: Homo sapiens protein tyrosine phosphatas					XM_94491	mRNA	blue
26360209-	Homo sapi	chromosor	nucleosom	DNA bindir	H2B/j; H2E	NM_00352	mRNA	blue
20993986-	Homo sapiens protein tyrosine phosphatase				DKFZp686I	NM_00101	mRNA	blue
AGGCCAA(	PREDICTED: Homo sapiens proteasome (prosome, mac					XM_00113	mRNA	blue
27273891-	Homo sapi	integral to	immune re	receptor a	IL4RA; CD1	NM_0010(	mRNA	blue
19682836-	Homo sapi	nucleus [g	regulation	zinc ion bir	GIOT-4; KC	NM_02103	mRNA	blue
23295564	Homo sapi	membrane	metabolisr	zinc ion bir	PLAP; ALP	NM_00163	mRNA	blue
8227510-8	Homo sapi	Golgi mem	regulation	molecular	ICAp69; IC	NM_0223(	mRNA	blue
10481633	Homo sapi	nucleus [g	protein kin	transferase	FLJ36101;	NM_18269	mRNA	blue
CCAAAACA	PREDICTED: Homo sapiens similar to M-phase phospho					XR_01907	mRNA	blue
22314357	Homo sapi	cytoplasm	protein bic	phenylalar	PheHB; FR	NM_00568	mRNA	blue
CAGGCTTG	PREDICTED: Homo sapiens similar to protein immuno-r					XM_9389C	mRNA	blue
TCACCTGA(	PREDICTED: Homo sapiens similar to Williams Beuren s					XM_93582	mRNA	blue
CTGGCTGA	PREDICTED: Homo sapiens similar to Zinc finger proteir					XR_01861(	mRNA	blue
29289975-	Homo sapiens RUN domain containing 2B (RUNDC2B),					NM_00101	mRNA	blue
14255603(	PREDICTED: Homo sapiens similar to peptidylprolyl isor					XM_9277C	mRNA	blue
	PREDICTED: Homo sapiens similar to Ran-binding prote					XM_93411	mRNA	blue
69712823-	Homo sapiens phenaz	biosynthes	catalytic a		FLJ14767;	NM_02212	mRNA	blue
14839771	Homo sapi	nucleus [g	regulation	zinc ion bir	DKFZp762I	NM_15241	mRNA	blue
AAACTAAG	PREDICTED: Homo sapiens colony stimulating factor 2 r					XM_9425C	mRNA	blue
76970258-	Homo sapi	membrane [goid 16020] [eviden			RP11-217H	NM_03212	mRNA	blue
70564812-	PREDICTED: Homo sapiens similar to nuclear pore mem					XM_00112	mRNA	blue
93213090-	Homo sapi	intracellula	ubiquitin c	oxygen tra	FLJ16050	NM_17349	mRNA	blue
14806601-	Homo sapi	cellular coi	biological p	molecular	NY-BR-1.1	NM_00102	mRNA	blue
75909314-	Homo sapiens KIAA1618 (KIAA1618), mRNA.					NM_02095	mRNA	blue
46820864-	Homo sapiens centrosomal protein 152kDa				KIAA0912	NM_01498	mRNA	blue
17740901	PREDICTED: Homo sapiens similar to NY-REN-7 antigen					XM_93311	mRNA	blue
44290026-	Homo sapi	protein ser	protein am	hydrolase	PPC2BETA	NM_00103	mRNA	blue
38041724-	Homo sapiens yrdC domain containing (E. cc				FLJ23476;	NM_02464	mRNA	blue
17183199-	Homo sapiens hypothetical protein FLJ34047 (FLJ34047					NM_17366	mRNA	blue
40646836-	Homo sapi	centrosome [goid 5813] [pmid 14			FLJ10460;	NM_01809	mRNA	blue
12124760-	Homo sapi	cytoplasm	regulation	protein bir	KIAA1408;	NM_01554	mRNA	blue

89852902-	Homo sapiens chromosome 14 o binding [gc FLJ14051;	NM_01797	mRNA	blue
ATCACCTG/	PREDICTED: Homo sapiens similar to Williams Beuren s	XM_93580	mRNA	blue
130443163	PREDICTED: Homo sapiens hypothetical protein FLJ436	XM_93054	lncRNA	blue
8382616-8	PREDICTED: Homo sapiens hypothetical protein LOC64:	XM_93187	mRNA	blue
22556843-	Homo sapi membrane transport [glucose tra MGC11883	NM_00102	mRNA	blue
GGGCGACA	Homo sapiens succinate dehydrogenase cor SDHAL1	NR_00326	mRNA	blue
38114867-	Homo sapi cytosolic ri regulation transferase GCN2; KIA/	NM_00101	mRNA	blue
AAATGTAC	PREDICTED: Homo sapiens similar to dicer1 (LOC64769	XM_93674	mRNA	blue
22002600-	PREDICTED: Homo sapiens similar to Dihydrofolate red	XM_92815	mRNA	blue
11197165(	Homo sapi membrane small GTPa nucleotide RAP1; KRE'	NM_00101	mRNA	blue
42861220-	Homo sapiens FLJ38717 protein (FLJ38717), FLJ40915	NM_0010(	mRNA	blue
37875474-	Homo sapi integral to transport [binding [gc CG7943; M	NM_03341	mRNA	blue
110714861	PREDICTED: Homo sapiens similar to RAN-binding prote	XM_93465	mRNA	blue
AACATGAC	PREDICTED: Homo sapiens hypothetical protein FLJ407	XM_94209	mRNA	blue
111519243	Homo sapi extracellul: immune re interleukin MGC1232(	NM_0015(	mRNA	blue
16829066-	PREDICTED: Homo sapiens hypothetical protein MGC1:	XM_93048	mRNA	blue
'AAGGTGG(	PREDICTED: Homo sapiens solute carrier family 25 (car	XM_00113	mRNA	blue
GCACAGT(	PREDICTED: Homo sapiens hypothetical protein LOC64'	XM_93646	mRNA	blue
69239594-	Homo sapi actin cytoskeleton [gc tropomyos DKFZp313I	NM_19827	mRNA	blue
203398622	Homo sapiens amyotrophic lateral sclerosis 2 (juvenile)	NM_17823	mRNA	blue
155749982	PREDICTED: Homo sapiens similar to Fc receptor-like 5	XM_00113	mRNA	blue
5923495-5	Homo sapi nucleus [gc DNA replic DNA-depe| MGC4816;	NM_1828(	mRNA	blue
20171476-	PREDICTED: Homo sapiens zinc finger protein 486 (ZNF	XM_37115	mRNA	blue
46552207-	Homo sapiens hypothetical protein MGC4093 (MGC409	NM_03057	mRNA	blue
115981532	Homo sapiens tudor domain con zinc ion bir FLJ21082	NM_19879	mRNA	blue
100073632	Homo sapi membrane ion transpc transporte SMCT; MG	NM_14591	mRNA	blue
34185801-	Homo sapi endoplasm cell surface 1-phospha Pip4k2B; P	NM_13868	mRNA	blue
17351414-	Homo sapi nucleus [gc response t transcription factor ac	NM_00162	mRNA	blue
112851351	PREDICTED: Homo sapiens similar to RAN-binding prote	XM_93099	mRNA	blue
33779905-	Homo sapiens chromc protein fol unfolded p C21orf78	NM_01783	mRNA	blue
49263889-	Homo sapi nucleus [gc regulation DNA binding [goid 367	NM_01336	mRNA	blue
42178233-	PREDICTED: Homo sapiens similar to AI661453 protein	XM_37182	mRNA	grey
32906458-	Homo sapi TAP compl immune re tapasin bir RING11; PS	NM_01883	mRNA	grey
27732933-	Homo sapi nucleus [gc morphoge transcripti TBR2	NM_00544	mRNA	grey
110552004	Homo sapi membrane [goid 1602 transferase NLRR-3; NI	NM_01833	mRNA	grey
180365765	Homo sapi membrane lipid metabolism [goic BTNLR	NM_0067(	mRNA	grey
148067255	PREDICTED: Homo sapiens similar to H3 histone, family	XM_49771	mRNA	grey
54174433-	Homo sapi membrane metabolisr acyltransfe FLJ20481;	NM_01783	mRNA	grey
TCAGGAA(	Homo sapiens thioredoxin (TXN), mRNA.	NM_00332	mRNA	turquoise
8293227-8	Homo sapi intracellula protein bic structural constituent	NM_00103	mRNA	turquoise
91411148-	Homo sapiens growth cell differe protein bir DDIT2; CR6	NM_0067(	mRNA	turquoise
44615731-	Homo sapi ribosome [protein bic structural constituent	NM_00102	mRNA	turquoise
185184354	Homo sapi integral to transport [ATPase act MOAT-C; p	NM_00102	mRNA	turquoise
113471394	Homo sapi heterotrim G-protein (GTPase activity [goid :	NM_00101	mRNA	turquoise
70442883-	Homo sapiens LMBR1 domain containing 1 (bA810I22.:	NM_01836	mRNA	turquoise
GTTCTCGCT	PREDICTED: Homo sapiens similar to 40S ribosomal prc	XM_93643	mRNA	turquoise
36153798-	Homo sapi membrane [goid 16020] [evidenc GLIPR2; G/	NM_02234	mRNA	turquoise
20078363-	Homo sapiens ribosor skeletal de transferase pp90RSK2;	NM_00458	mRNA	turquoise

4316893-4	PREDICTED: Homo sapiens similar to Ssu72 RNA polym	XM_37234	mRNA	turquoise
TCCCAACA	Homo sapiens pre-B-c pyridine nu transferase DKFZP666I	NM_0057	mRNA	turquoise
75454418-	Homo sapi integral to membrane [goid 16021] [evidenc	NM_0319	mRNA	turquoise
75346964-	Homo sapi extracellul blood coag serine-type UPA; URK;	NM_0026	mRNA	turquoise
53063842-	Homo sapiens zyg-11 homolog B binding [gc FLJ13456;	NM_0246	mRNA	turquoise
44597523-	Homo sapi plasma membrane [gc protein bir SAR2; TSP	NM_0022	mRNA	turquoise
10190085	Homo sapi DNA-direct transcripti transferase RPB11; MC	NM_0062	mRNA	turquoise
58332875-	Homo sapiens hypothetical protein LOC284 FLJ46426	NM_0010	mRNA	turquoise
24652407-	Homo sapi membrane homophilic calcium ior DLX2; DYL	NM_0148	mRNA	turquoise
90407224-	Homo sapi nucleus [gc response t transferase RLPK; MSP	NM_0047	mRNA	turquoise
ACATGTGC	PREDICTED: Homo sapiens hypothetical protein LOC64	XM_9361	lncRNA	turquoise
GTCCACAT	Homo sapi membrane [goid 1602 receptor a EGFL5	NM_0010	mRNA	turquoise
3756-3805	PREDICTED: Homo sapiens similar to CXYorf1-related p	XR_0176	mRNA	turquoise
14808089	Homo sapi chromosor nucleosom DNA bindir H2a-615; H	NM_0035	mRNA	turquoise
6486367-6	Homo sapiens thioredoxin-like 5 electron ca TRP14; MG	NM_0327	mRNA	turquoise
60710651-	PREDICTED: Homo sapiens similar to hypothetical prote	XM_92681	mRNA	turquoise
AGCTAAAG	PREDICTED: Homo sapiens hypothetical protein LOC64	XM_93557	lncRNA	turquoise
15222988	Homo sapi ribosome [protein bic RNA bindir MPS-1; MF	NM_0010	mRNA	turquoise
68336908-	Homo sapiens G prote positive re receptor a C1orf139;	NM_0010	mRNA	turquoise
1388309-1	Homo sapi integral to plasma me receptor a CSF2R; CSF	NM_1722	mRNA	turquoise
38299200-	PREDICTED: Homo sapiens hypothetical protein LOC28	XM_37845	lncRNA	turquoise
TGACTGCC	PREDICTED: Homo sapiens hypothetical LOC646424 (LC	XM_9412	mRNA	turquoise
19558234-	Homo sapi nucleus [gc regulation zinc ion bir RAMP; SCL	NM_0034	mRNA	turquoise
34634020-	Homo sapiens protein phosphata calcium ior G5pr; FLJ2	NM_0179	mRNA	turquoise
17128557	Homo sapi membrane G-protein receptor a GPCR1; GP	NM_0143	mRNA	turquoise
12664737-	Homo sapiens hypothetical prote hydrolase CSTP1	NM_0183	mRNA	turquoise
22371126-	Homo sapi intracellula small GTPa guanyl-nuc MGC1196	NM_1536	mRNA	turquoise
82593742-	Homo sapi cytoplasm antibiotic t oxidoredu NECAB2	NM_0190	mRNA	turquoise
18003717	Homo sapi voltage-ga calcium ior voltage-ga CACH6; BII	NM_0007	mRNA	turquoise
10734697	Homo sapi mitochond electron tr disulfide o E3; PHE3; I	NM_0001	mRNA	turquoise
23897916-	Homo sapi cytosol [gc phototransduction [gc HRG4	NM_0051	mRNA	turquoise
14805181	PREDICTED: Homo sapiens similar to H3 histone, family	XM_49771	mRNA	turquoise
GAGCCCTTC	Homo sapi membrane [goid 16020] [evidenc CGI-119; Z	NM_0160	mRNA	turquoise
22664611-	Homo sapi intracellula intracellular signaling SWIP1; WS	NM_0156	mRNA	turquoise
24707151	Homo sapiens SH3-binding domain protein FLJ33845;	NM_0306	mRNA	turquoise
61085713-	Homo sapiens ubiquit ubiquitin-c peptidase MGC1319	NM_0063	mRNA	turquoise
34459695-	PREDICTED: Homo sapiens similar to SMT3 suppressor	XM_37252	mRNA	turquoise
52083405-	Homo sapiens AKT int ubiquitin c ubiquitin-p FTS; FT1	NM_0224	mRNA	turquoise
10052019	Homo sapi intracellular [goid 562 zinc ion bir DKFZP667	NM_0309	mRNA	turquoise
12331650-	Homo sapiens cell div cell cycle [goid 7049] C10orf7; D	NM_0060	mRNA	turquoise
23592413-	Homo sapi nucleus [gc regulation sequence- NKX3A; BA	NM_0061	mRNA	turquoise
11337048-	Homo sapiens lipid phosphate pl zinc ion bir FLJ13055;	NM_0227	mRNA	turquoise
45720017-	Homo sapi endoplasm metabolisr hydrolase MGC1264	NM_1985	mRNA	turquoise
11190318	Homo sapiens interfe cell differe binding [gc PC4; TIS7	NM_0015	mRNA	turquoise
11485989	PREDICTED: Homo sapiens similar to SMT3 suppressor	XM_00112	mRNA	turquoise
13410774	Homo sapi membrane cell differe GPI anchor bA479K20	NM_0325	mRNA	turquoise
ACTTTCCC	PREDICTED: Homo sapiens similar to F15D3.1a (LOC39	XM_93727	mRNA	turquoise
51243958-	Homo sapiens dehydr metabolisr oxidoredu FLJ13639	NM_0010	mRNA	turquoise

66796993-	Homo sapi	cell fractio	signal tran	hydrolase	HCP1; PDE	NM_0026	mRNA	turquoise
15708427	Homo sapi	nucleus [g	cellular de	DNA bindir	PYHIN3	NM_0024	mRNA	turquoise
TCGTGTCC	Homo sapi	nucleus [g	regulation	transcripti	E2FBP1; BI	NM_0052	mRNA	turquoise
20210812-	Homo sapiens chromosome Y open reading				MGC13173	NM_0010	mRNA	turquoise
17492157	Homo sapi	nucleus [g	transcripti	protein binding [goid		NM_0048	mRNA	turquoise
GGTGGAG	Homo sapi	nucleus [g	chloride tr	chloride ch	RP11-475E	NM_0010	mRNA	turquoise
46295471-	Homo sapiens transd		negative re	SH3/SH2 a	MGC10479	NM_0057	mRNA	turquoise
12620016	Homo sapi	nucleus [goid 5634] [e		structural	MGC1114	NM_0055	mRNA	turquoise
76166279-	Homo sapi	plasma me	blood coag	thrombin r	PAR2; GPR	NM_0052	mRNA	turquoise
ATGGTATCA	PREDICTED: Homo sapiens similar to Fc fragment of IgG					XM_9420	mRNA	turquoise
76866031-	Homo sapiens chrom		pyridine nu	kinase acti	NRK1; bA2	NM_0178	mRNA	turquoise
88514916-	Homo sapiens hydrox		metabolisr	oxidoredu	DHRS8; PA	NM_0162	mRNA	turquoise
1491641-1	Homo sapi	integral to	cell surface receptor l		SIRP-BETA-	NM_0060	mRNA	turquoise
13756055	Homo sapi	integral to	response t	cytokine bi	CD119; IFN	NM_0004	mRNA	turquoise
14606854	PREDICTED: Homo sapiens neuroblastoma breakpoint f					XM_93402	mRNA	turquoise
GTGGTAGT	PREDICTED: Homo sapiens similar to hypothetical LOC3					XM_94367	lncRNA	turquoise
24190563	Homo sapi	contractile	cell cycle [g	GTPase act	KIAA0158;	NM_0010	mRNA	turquoise
8565844-8	Homo sapi	membrane	immune re	sugar bind	CLEC6; MG	NM_0803	mRNA	turquoise
18490806	Homo sapi	nucleus [g	physiologi	oxidoredu	PHS-2; CO	NM_0009	mRNA	turquoise
TCGAGTGG	Homo sapi	plasma me	cell-cell sig	zinc ion bir	MGC12668	NM_0072	mRNA	turquoise
23252401-	Homo sapiens ureido		nitrogen c	zinc ion bir	BUP1	NM_0163	mRNA	turquoise
31198110-	Homo sapi	nucleus [goid 5634] [e		RNA polym	TAFII68; TA	NM_1392	mRNA	turquoise
13357493	Homo sapi	intracellula	small GTPa	guanyl-nuc	DKFZp781I	NM_1986	mRNA	turquoise
13310682	Homo sapi	membrane	nitrogen c	hydrolase	FOAP-4; GI	NM_0784	mRNA	turquoise
17995025	Homo sapi	extracellul	regulation	cytokine a	MGC87867	NM_0528	mRNA	turquoise
CCATGGCC	PREDICTED: Homo sapiens similar to ribosomal protein					XM_94115	mRNA	turquoise
11205355	Homo sapiens thiore		cell motilit	thiol-disulf	DKFZp686I	NM_0033	mRNA	turquoise
14558593	Homo sapiens acid phosphatase			hydrolase	PACPL1; A	NM_0163	mRNA	turquoise
CTCCTGCG	PREDICTED: Homo sapiens hypothetical protein LOC65					XM_94441	mRNA	turquoise
48763124-	Homo sapi	plasma me	intracellula	kinase acti	MGC3606;	NM_0041	mRNA	turquoise
10483792	Homo sapi	cytosol [goid 5829] [e		hydrolase	cN-II; NT5E	NM_0122	mRNA	turquoise
10393633	Homo sapiens ubiquit		ubiquitin c	ligase activ	E2(17)KB3	NM_1818	mRNA	turquoise
TTCTCTCCT	Homo sapi	nucleus [g	regulation	sequence-s	G17; PBX2	NM_0025	mRNA	turquoise
20531752	Homo sapiens 6-phos		metabolisr	kinase acti	DKFZp781I	NM_0062	mRNA	turquoise
22972970	PREDICTED: Homo sapiens hypothetical LOC440731, tr					XM_93369	lncRNA	turquoise
15376725	Homo sapi	integral to membrane [goid 1602			FLJ32028	NM_1526	mRNA	turquoise
11488107	Homo sapi	nucleus [g	regulation	receptor a	PROSIT240	NM_0153	mRNA	turquoise
CGGGGAG	Homo sapi	lysosome [	ceramide r	transferas	AC; FLJ220	NM_1779	mRNA	turquoise
18591394	Homo sapi	membrane	metabolisr	ligase activ	FACL2; LA	NM_0019	mRNA	turquoise
48220757-	Homo sapi	extracellul	pregnancy [goid 7565		MGC22484	NM_0027	mRNA	turquoise
93486339-	Homo sapi	membrane [goid 16020] [eviden			KIAA1145	NM_0206	mRNA	turquoise
37676117-	Homo sapi	extracellul	cell motilit	signal tran	TSP; THBS;	NM_0032	mRNA	turquoise
10159841	Homo sapi	membrane	transport [	transporte	OATP-M1;	NM_1809	mRNA	turquoise
10353434	Homo sapiens mening		glycoprote	hyaluronol	MEA5; FLJ	NM_0122	mRNA	turquoise
21912437	Homo sapi	nucleus [g	regulation	sequence-s	HB24	NM_0219	mRNA	turquoise
45721550-	Homo sapi	endoplasm	metabolisr	hydrolase	MGC12641	NM_0188	mRNA	turquoise
11378458	Homo sapiens RNA bi		meiosis [g	RNA bindir	FLJ11153	NM_0160	mRNA	turquoise
27865202-	Homo sapi	membrane	anti-apopt	protein bir	IFI-6-16; 6-	NM_0020	mRNA	turquoise

:CTTAGACA	PREDICTED: Homo sapiens similar to ribosomal protein					XM_94115	mRNA	turquoise
48241909-	Homo sapi	extracellular region [goid 5576] [			LL37; FALL	NM_00434	mRNA	turquoise
3012033-3	Homo sapi	membrane	cell differe	receptor a	CD266; TW	NM_01663	mRNA	turquoise
38159368-	Homo sapi	nucleus [gc	DNA repai	DNA bindi	FLJ46828;	NM_15226	mRNA	turquoise
19154862	Homo sapi	nucleus [gc	transcripti	transcripti	ISGF-3; STA	NM_13926	mRNA	turquoise
48842368-	Homo sapi	plasma me	cell motilit	GPI anchor	CD87; UPA	NM_00100	mRNA	turquoise
3067402-3	PREDICTED: Homo sapiens hypothetical protein LOC64					XM_93337	mRNA	turquoise
31031334-	Homo sapi	alpha-keto	branched c	kinase activity [goid 1		NM_00588	mRNA	turquoise
65170462-	Homo sapi	integral to	chemotaxi	cytokine a	CKLFH; CKI	NM_18130	mRNA	turquoise
ACTGAGCA	PREDICTED: Homo sapiens hypothetical protein LOC64					XM_94304	lncRNA	turquoise
67924838-	Homo sapi	nucleus [gc	apoptosis [goid 6915]		GADD45; D	NM_00192	mRNA	turquoise
GTACAAGG	Homo sapi	clathrin co	morphoge	binding [gc	CLH22; CLT	NM_00709	mRNA	turquoise
49122497-	Homo sapi	ribosome [	protein bic	structural constituent		NM_00103	mRNA	turquoise
14808981(	PREDICTED: Homo sapiens similar to Histone H2A.o (H2					XM_92838	mRNA	turquoise
17796264	Homo sapi	nucleus [gc	protein am	ATP bindin	DKFZp686	NM_02066	mRNA	turquoise
ACCGAGAG	Homo sapi	plasma me	cell-cell sig	zinc ion bir	NEP; MGC	NM_00090	mRNA	turquoise
50441973-	Homo sapiens phosph		glycogen m	pyridoxal phosphate b		NM_00286	mRNA	turquoise
10514353-	Homo sapiens ropporin 1-like (ROPN1L), mR				ASP; FLJ25	NM_03191	mRNA	turquoise
81530777-	Homo sapi	nucleus [gc	regulation of transcrip		BCE-1; ESG	NM_00700	mRNA	turquoise
48319749-	Homo sapiens RNA bi		RNA proce	RNA bindir	RNPL; IS1-I	NM_00101	mRNA	turquoise
ATCGACCG(	PREDICTED: Homo sapiens H3 histone, family 3A pseud					XR_01783	mRNA	turquoise
91149642-	Homo sapi	cytoplasm	immune re	binding [gc	RNM561; I	NM_00154	mRNA	turquoise
13481379-	Homo sapiens ubiquit		ubiquitin-c	calcium-de	DFFRY; SP3	NM_00465	mRNA	turquoise
10174011-	Homo sapi	integral to	phagocyto	pattern re	BGR; DECT	NM_19795	mRNA	turquoise
17453502	Homo sapi	nucleus [gc	regulation	transcription corepres		NM_00386	mRNA	turquoise
13918925-	Homo sapi	nucleus [goid 5634] [e		binding [gc	UTY1; DKF	NM_18265	mRNA	turquoise
15116681	Homo sapi	actin cytos	regulation	actin bindi	D3S1319E;	NM_05302	mRNA	turquoise
70023312-	Homo sapi	nucleus [gc	cell prolife	transcripti	MGC10465	NM_00235	mRNA	turquoise
43834109-	Homo sapi	membrane	metabolisr	oxidoreduc	KAR	NM_01614	mRNA	turquoise
24150565-	Homo sapi	nucleus [gc	nuclear mF	RNA bindir	Ht006; SF3	NM_01604	mRNA	turquoise
ACTGCCCG(	PREDICTED: Homo sapiens similar to H3 histone, family					XM_93792	mRNA	turquoise
11856670	Homo sapiens hypothetical protein LOC283				MGC12544	NM_00103	mRNA	turquoise
12907768-	Homo sapi	nucleus [gc	protein am	calmodulir	CKLiK; CaM	NM_02039	mRNA	turquoise
3934-3983	PREDICTED: Homo sapiens similar to CXYorf1-related p					XR_01761	mRNA	turquoise
37187233-	Homo sapi	intracellula	immune re	transferase	PRKR; PKR	NM_00275	mRNA	turquoise
GAGGAAAC(	Homo sapi	cytoskelet	barrier sep	cytoskeleta	MGC14978	NM_01868	mRNA	turquoise
32161087-	Homo sapi	membrane [goid 16020] [eviden			FLJ90675;	NM_02213	mRNA	turquoise
37770607-	Homo sapi	cytoplasmi	rRNA proc	exonuclea	RP11-3J10	NM_00100	mRNA	turquoise
AGCAGAC	PREDICTED: Homo sapiens similar to CG7889-PA, trans					XM_94293	mRNA	turquoise
19104754	Homo sapiens regulat		regulation	calmodulir	G0S8	NM_00292	mRNA	turquoise
67689479-	PREDICTED: Homo sapiens hypothetical protein LOC64					XM_92886	mRNA	turquoise
44510871-	Homo sapi	intracellula	protein am	enzyme ac	MGC12686	NM_00487	mRNA	turquoise
56780689-	PREDICTED: Homo sapiens similar to Ubiquinol-cytochr					XM_92804	mRNA	turquoise
65144146-	Homo sapi	integral to	macrophag	chemokine	UCK-1; CKL	NM_01632	mRNA	turquoise
13256579	Homo sapiens nudix (nucleoside diphosphat				FLJ20525	NR_00294	mRNA	turquoise
14307218	PREDICTED: Homo sapiens similar to peptidylprolyl isor					XM_92776	mRNA	turquoise
2962146-2	Homo sapiens NAD(P)		electron tr	zinc ion bir	DHQV; DIA	NM_00090	mRNA	turquoise
15177380	Homo sapi	ruffle [goic	cell-cell sig	protein ho	PRA; CABP	NM_01462	mRNA	turquoise

CCTGAGGA	PREDICTED: Homo sapiens family with sequence simila				XM_00112	mRNA	turquoise
GCTTTGTC	Homo sapiens family with sequence similari			MGC4587	NM_1827	mRNA	turquoise
17807132	Homo sapi	nucleus [g	transcripti	RNA polym	EZNF; TCF	NM_0056	mRNA	turquoise
CTGTGGGG	Homo sapi	extracellul	cell-cell sig	signal tran	CFAG; MA	NM_0029	mRNA	turquoise
CATTTACCA	PREDICTED: Homo sapiens hypothetical LOC440160 (LC				XM_49857	mRNA	turquoise
72067992-	Homo sapi	membrane	[goid 1602	receptor a	dJ331H24.	NM_0245	mRNA	turquoise
9998450-9	Homo sapiens retinol		transport [	retinal bin	CRBP4; M	NM_0529	mRNA	turquoise
48399408-	Homo sapiens chromosome 22 open reading frame 34					NM_0010	mRNA	turquoise
CGGGCAGT	Homo sapi	plasma me	homophili	protein ho	PVRR2; HV	NM_0028	mRNA	turquoise
15851759	Homo sapiens hypothetical prote			protein bir	DKFZp686	NM_1447	mRNA	turquoise
94892061-	Homo sapi	cytosol [g	biosynthes	lyase activ	HSTD; histi	NM_0021	mRNA	turquoise
63928321-	Homo sapi	endoplasm	metabolisr	hydrolase	KIAA1001	NM_0149	mRNA	turquoise
ACCAACTC	Homo sapiens ubiquit		ubiquitin-c	ubiquitin t	MGC2621;	NM_0051	mRNA	turquoise
22953563	Homo sapiens exocys		protein tra	protein bir	Exo84p; SE	NM_1758	mRNA	turquoise
34816549-	Homo sapi	nucleus [g	transcripti	DNA bindi	TRIP2; PPA	NM_0047	mRNA	turquoise
GAGAGATA	PREDICTED: Homo sapiens dehydrogenase/reductase (				XM_00113	mRNA	turquoise
23855914-	Homo sapi	integral to	cell motilit	nucleotide	LTBR1; P2Y	NM_1816	mRNA	turquoise
51285425-	PREDICTED: Homo sapiens hypothetical protein FLJ373				XR_00060	lncRNA	turquoise
74973428-	Homo sapi	mitochond	glycolysis [	kinase acti	HKII; HXK2	NM_0001	mRNA	turquoise
54546773-	Homo sapi	ribosome [	protein bic	structural constituent		NM_0010	mRNA	turquoise
97673262-	Homo sapi	integral to membrane		transferas	KIAA1079;	NM_0149	mRNA	turquoise
1716348-1	Homo sapiens transforming, acidic coiled-cc				MGC1332	NM_0063	mRNA	turquoise
75103777-	Homo sapi	plasma me	cellular de	corecepto	MD-2	NM_0153	mRNA	turquoise
42795187-	Homo sapi	extracellul	immune re	MHC class I receptor		NM_0040	mRNA	turquoise
32030109-	Homo sapiens chromosome 12 open readin				FLJ20696;	NM_0181	mRNA	turquoise
39061214-	Homo sapiens trinucleotide repe			nucleotide	KIAA1093	NM_0010	mRNA	turquoise
99472880-	Homo sapi	nucleus [g	regulation	zinc ion bi	MGC1384	NM_0034	mRNA	turquoise
15986014	Homo sapi	membrane	immune re	receptor a	CD16b; FC	NM_0005	mRNA	turquoise
28692285-	Homo sapi	nucleus [g	positive re	sequence-	CRE-BPA	NM_0010	mRNA	turquoise
15970908-	Homo sapi	integral to	cell surface	receptor a	hIL-17R; IL	NM_0143	mRNA	turquoise
15702493	Homo sapi	integral to	protein am	transmem	PTPRP; ICA	NM_1308	mRNA	turquoise
17932641	Homo sapi	integral to	apoptosis		zinc ion bi	G1RZFP; M	NM_0184	mRNA	turquoise
CTCCAGGC	PREDICTED: Homo sapiens hypothetical protein LOC64				XM_9419	lncRNA	turquoise
1818688-1	Homo sapiens tropon		regulation	actin bindi	DA2B; AM	NM_0032	mRNA	turquoise
15162923	Homo sapi	extracellul	inflammat	calcium io	CFAG; 60B	NM_0029	mRNA	turquoise
71528496-	Homo sapi	membrane	folic acid t	folic acid b	FR-gamma	NM_0008	mRNA	turquoise
27841663-	Homo sapi	cyclin-dep	brain deve	cadherin b	p35nck5a;	NM_0038	mRNA	turquoise
38474336-	Homo sapi	plasma me	positive re	transferas	KIAA0012;	NM_0032	mRNA	turquoise
8226343-8	Homo sapi	large ribos	protein bic	structural constituent		NM_0009	mRNA	turquoise
11613369	Homo sapi	extracellul	acute-phas	binding [g	AGP2; AGF	NM_0006	mRNA	turquoise
3811452-3	Homo sapi	nucleus [g	B cell diffe	transcripti	GBF; ZF9; S	NM_0013	mRNA	turquoise
77415032-	Homo sapi	membrane	respirator	leukotrien	CYSLTR; M	NM_0066	mRNA	turquoise
3918-3967	PREDICTED: Homo sapiens similar to similar to RPL23A				XM_9272	mRNA	turquoise
15634207-	Homo sapi	endoplasm	leukotrien	iron ion bi	CPF3; CYP	NM_0008	mRNA	turquoise
13391737-	Homo sapiens phosphatase and			protein ph	RPEL; MG	NM_0309	mRNA	turquoise
52892908-	Homo sapiens protei		protein m	protein-L-i	FLJ10883	NM_0529	mRNA	turquoise
49120092-	Homo sapi	ribosome [	protein bic	structural constituent		NM_0010	mRNA	turquoise
193631-19	PREDICTED: Homo sapiens hypothetical protein LOC64				XM_92804	mRNA	turquoise

13005821{	Homo sapi	Golgi apparatus [goid	protein bir	MGC2067:	NM_0044{	mRNA	turquoise
89330079-	PREDICTED: Homo sapiens similar to 40S ribosomal prc				XM_92818	mRNA	turquoise
52968633-	Homo sapiens RNA, 7SK, nuclear (RN7SK) or			7SK	NR_00144	mRNA	turquoise
13869775-	Homo sapi	nucleus [goid 5634] [e	binding [gc	UTY1; DKF:	NM_0071:	mRNA	turquoise
78692404-	Homo sapi	actin cytos	regulation	signal tran:	MGC1330‹	NM_0024{	mRNA	turquoise
13564383{	Homo sapi	soluble fra	regulation	Rab GTPas	RAB3GAP;	NM_0122:	mRNA	turquoise
60091988-	Homo sapi	plasma me	immune re	receptor a	CD89	NM_1332:	mRNA	turquoise
59290134-	Homo sapiens osteoclast-associa		receptor a	MGC3361:	NM_1307:	mRNA	turquoise
10289291-	Homo sapi	microtubu	neuromus	ATPase act	KIAA0591;	NM_18341	mRNA	turquoise
64481496-	Homo sapiens PHD fir		regulation	zinc ion bir	MGC1422:	NM_0151!	mRNA	turquoise
20278583(	Homo sapi	nucleus [g	protein sta	zinc ion bir	MDMX; Dk	NM_0023!	mRNA	turquoise
32597972-	Homo sapi	nucleus [g	chromatin	transcripti	Epl1; DKFZ	NM_0252(	mRNA	turquoise
55386776-	Homo sapi	cytoskelet	mitotic spi	RNA bindir	MIG14; M(	NM_00361	mRNA	turquoise
15797981{	Homo sapi	membrane	regulation	protein bir	CASP; CYTI	NM_0042{	mRNA	turquoise
17642594(	Homo sapi	nucleus [g	apoptosis		zinc ion bir	Zfp346; JA:	NM_0122:	mRNA	turquoise
75006821-	Homo sapi	nucleus [g	regulation	protein dir	JUNDM2	NM_1304(	mRNA	turquoise
48486565-	Homo sapi	membrane	ion transp	transporte	MGC1325:	NM_0010:	mRNA	turquoise
;TTATCAGA	PREDICTED: Homo sapiens similar to H3 histone, family				XM_00112	mRNA	turquoise
15161298:	Homo sapi	insoluble f	xenobiotic	zinc ion bir	CAAF1; CA	NM_0056:	mRNA	turquoise
16594166:	Homo sapiens RCSD domain con		kinase acti	RP3-503M	NM_0528(	mRNA	turquoise
1388406-1	Homo sapi	integral to plasma me	receptor a	CSF2R; CSF	NM_1722‹	mRNA	turquoise
31715625-	Homo sapiens antagonist of mitotic exit network 1 hon				NM_2073:	mRNA	turquoise
81587223-	Homo sapi	membrane [goid 16020] [evidence NAS]; int			NM_0230(	mRNA	turquoise
42915789-	Homo sapiens chromosome 7 open reading			MGC2821;	NM_0240!	mRNA	turquoise
15843922:	Homo sapiens synaptojanin 2 (SY		RNA bindir	KIAA0348;	NM_0038!	mRNA	turquoise
13116239-	Homo sapi	membrane	protein transport [goi		SYN10; hsy	NM_0037(	mRNA	turquoise
30360904-	Homo sapi	membrane	ubiquitin c	zinc ion bir	RNF189; R	NM_0010:	mRNA	turquoise
45889570-	Homo sapiens aarF domain cont		kinase acti	COQ8; FLJ:	NM_0248:	mRNA	turquoise
52320501-	Homo sapiens tripartite motif-co		zinc ion bir	Z147; ZNF:	NM_0050{	mRNA	turquoise
19756814:	Homo sapiens ankyrin repeat domain 44 (AI			MGC7044‹	NM_1536!	mRNA	turquoise
13877579:	Homo sapiens heme binding protein 2 (HEBI			RP3-422G:	NM_0143:	mRNA	turquoise
34260200-	Homo sapi	ribosome [	protein bic	structural	MGC1173‹	NM_0009:	mRNA	turquoise
20225225-	Homo sapiens chromosome Y open reading frame 15B				NM_0325:	mRNA	turquoise
10772071(	Homo sapi	integral to	immune re	tumor nec	ZTNF4; TAI	NM_0065:	mRNA	turquoise
69730861-	Homo sapi	cytoplasm	antimicrob	protein kin	GIG3; MOI	NM_0038:	mRNA	turquoise
19042121(	Homo sapiens regulat		negative re	signal tran:	RGS13	NM_1307{	mRNA	turquoise
15199374(	Homo sapiens paraneoplastic antigen like 6/			MGC1582:	NM_0328{	mRNA	turquoise
70533801-	Homo sapiens serglycin (SRGN), mRNA.			PRG1; PPG	NM_0027:	mRNA	turquoise
14589154-	Homo sapi	nucleus [g	ubiquitin-c	threonine	MGC2285:	NM_1489:	mRNA	turquoise
14886661(	Homo sapiens endos		transport [	ion channe	MGC4319;	NM_2071(	mRNA	turquoise
12157899!	Homo sapiens chromosome 10 open readin			FLJ36756;	NM_0248:	mRNA	turquoise
61282171-	Homo sapi	nucleus [g	positive re	transcripti	HIF-1alpha	NM_0015:	mRNA	turquoise
89219327-	Homo sapiens RNA binding motif protein, X-			KAT3	NM_01961	mRNA	turquoise
26655419-	Homo sapi	integral to plasma membrane [g			EVDB; D17	NM_0064!	mRNA	turquoise
10920476‹	Homo sapiens insulin		glucose me	receptor activity [goic		NM_0037‹	mRNA	turquoise
88129409-	Homo sapi	ER-Golgi in	angiogene:	metallope	APN; gp15	NM_0011!	mRNA	turquoise
TCCGAATG	PREDICTED: Homo sapiens membrane bound O-acyltra				XM_0011:	mRNA	turquoise
23963544:	Homo sapi	nucleus [g	B cell diffe	transcripti	HD4; HDA(	NM_0060:	mRNA	turquoise

42355401-	Homo sapi	nucleus [g(	regulation	zinc ion bi	FLJ23765;	NM_15265	mRNA	turquoise
ACCTAGG(	Homo sapi	integral to	G-protein (	5-oxo-6E,8	GPR170; G	NM_14896	mRNA	turquoise
12778030(	Homo sapi	nucleus [g(	protein am	transferase	MEKK2; M	NM_0066(	mRNA	turquoise
11343382:	Homo sapiens hypothetical protein LOC552891 (LOC55					NM_00412	mRNA	turquoise
72657673-	Homo sapiens RNA bi		nuclear mF	mRNA binc	S164; MGC	NM_0212:	mRNA	turquoise
18186212(	Homo sapi	Arp2/3 prc	cell motilit	structural (	ARC16; dJ1	NM_00571	mRNA	turquoise
31836086-	Homo sapi	chromosor	nucleosom	DNA binding [goid 367		NM_00101	mRNA	turquoise
33021836-	Homo sapi	endoplasm	nucleotide	hydrolase :	MGC27337	NM_0010(	mRNA	turquoise
12856899-	Homo sapi	ribosome [	protein bic	structural (	PP9932	NM_0010(	mRNA	turquoise
55431745-	Homo sapiens ankyrin repeat domain 55 (AN				FLJ11795;	NM_02466	mRNA	turquoise
CATGGTGT	PREDICTED: Homo sapiens similar to 60S ribosomal prc					XM_94221	mRNA	turquoise
15342563-	Homo sapi	membrane	developme	hydrolase :	CD157	NM_0043:	mRNA	turquoise
43502841-	Homo sapi	chromatin	chromatin	chromatin	M31; HP1F	NM_0068(	mRNA	turquoise
GGTCCTGT(	PREDICTED: Homo sapiens similar to general transcript					XM_93575	mRNA	turquoise
60706623-	PREDICTED: Homo sapiens similar to hypothetical prote					XM_92681	mRNA	turquoise
8577850-8	Homo sapi	membrane	immune re	sugar bind	CLECSF9; N	NM_01435	mRNA	turquoise
54527196-	Homo sapiens chromosome 20 open readin				HSPC164; S	NM_0164(	mRNA	turquoise
7309013-7	Homo sapiens proteir		protein am	ATP binding [goid 552		NM_00276	mRNA	turquoise
15484653(	Homo sapi	plasma me	induction (	transferase	CD282; TIL	NM_00326	mRNA	turquoise
34940976-	Homo sapi	nucleus [g(	cytoplasmi	ubiquitin p	IKBA; NFKE	NM_02052	mRNA	turquoise
36019743-	Homo sapi	integral to	transport [	transporte	TAT1; FLJ3	NM_05296	mRNA	turquoise
19677239:	Homo sapi	intracellula	ubiquitin c	ligase activ	DKFZp686I	NM_02076	mRNA	turquoise
GCAAGTTTC	PREDICTED: Homo sapiens hypothetical protein FLJ120					XR_00126:	lncRNA	turquoise
14326989-	Homo sapi	cytoskelet(	regulation	actin bindi	TB4Y; MGC	NM_0042(	mRNA	turquoise
CTTGTTCCC	Homo sapiens metallophosphoes			hydrolase activity [goi		NM_02307	mRNA	turquoise
39045111-	Homo sapiens leucine		small GTPa	binding [gc	RIPK7; ROC	NM_19857	mRNA	turquoise
32901537-	Homo sapi	TAP compl	protein coi	tapasin bir	APT2; RINC	NM_00054	mRNA	turquoise
85379025-	Homo sapi	coated pit	protein coi	clathrin bir	LAP; CALM	NM_00716	mRNA	turquoise
61941594-	Homo sapi	nucleus [g(	chromatin	ATP bindin	FLJ20361;	NM_01778	mRNA	turquoise
23784959-	Homo sapi	membrane	homophilic	calcium ioi	CDw325; C	NM_00179	mRNA	turquoise
60232730-	PREDICTED: Homo sapiens similar to pleckstrin homolo					XM_93518	mRNA	turquoise
57945667-	Homo sapiens proteir		glycogen n	protein ph	PPP1R6; D	NM_00624	mRNA	turquoise
75269880-	Homo sapiens oxyster		lipid transport [goid 6		ORP8; MG	NM_0010(	mRNA	turquoise
21870990:	Homo sapi	integral to	neutrophil	receptor a	CD182; CM	NM_00155	mRNA	turquoise
46828278-	Homo sapiens ankyrir		intracellular signaling		FLJ21255;	NM_02409	mRNA	turquoise
CCTGTGGT(	Homo sapiens paraneoplastic an			zinc ion bir	MGC13275	NM_01336	mRNA	turquoise
21982328:	Homo sapi	microtubu	microtubu	GTP bindin	TUBA1; H2	NM_0060(	mRNA	turquoise
12377564:	Homo sapiens deltex 3-like (Dros			zinc ion bir	BBAP	NM_13828	mRNA	turquoise
52026216-	Homo sapiens oxyster		lipid transport [goid 6		FLJ14629;	NM_02458	mRNA	turquoise
53042762-	Homo sapi	membrane	G-protein (	receptor a	GPCR4; EX	NM_02037	mRNA	turquoise
CAACCATGT	Homo sapiens SUMO1 pseudogene 1 (SUMO1P1) on ch					NR_00218	mRNA	turquoise
2269595-2	Homo sapi	cornified e	skin develc	GTPase act	MGC12624	NM_00324	mRNA	turquoise
12050538:	Homo sapi	extracellul	regulation	growth fac	IGFBP9; NC	NM_00251	mRNA	turquoise
11188207(	Homo sapi	membrane [goid 1602		calmodulir	ADDL	NM_01682	mRNA	turquoise
ATCACATT(	Homo sapi	centrosome [goid 581		protein bir	MGC78416	NM_02518	mRNA	turquoise
74955506-	Homo sapi	extracellul	negative re	enzyme ac	GROa; GRC	NM_00151	mRNA	turquoise
TATTACCT(	Homo sapiens lines homolog 1 (Drosophila)				FLJ10583;	NM_00104	mRNA	turquoise
5426395-5	Homo sapiens similar to zinc finger protein 469 (LOC44					NM_00101	mRNA	turquoise

15454347-	Homo sapi	nucleus [g	regulation	DNA bindi	MGC74712	NM_03322	mRNA	turquoise
3257423-3	PREDICTED: Homo sapiens hypothetical protein FLJ396					XM_37093	lncRNA	turquoise
26207885-	Homo sapi	nucleus [g	chromatin	chromatin	HP1-GAMI	NM_01658	mRNA	turquoise
83500277-	Homo sapi	extracellular region [goid 5576] [			DKFZP434I	NM_03147	mRNA	turquoise
29648-296	PREDICTED: Homo sapiens similar to Proline-rich nucle					XM_93121	mRNA	turquoise
10515631	Homo sapi	membrane fraction [goid 5624] [			p63; CLIMI	NM_00682	mRNA	turquoise
10010000	Homo sapiens dJ341D10.1 (novel protein) (dJ341D10.1					NM_0010	mRNA	turquoise
10224110	Homo sapi	cytoplasm	ubiquitin c	transferas	Fbl13; FLJ3	NM_1450	mRNA	turquoise
93779668-	Homo sapi	nucleus [g	negative re	ATP bindin	TAF172; KI	NM_00397	mRNA	turquoise
37978594-	Homo sapi	nucleus [g	regulation	transcripti	TCFL4; MX	NM_1706	mRNA	turquoise
71639809-	Homo sapi	integral to	positive re	cell adhesi	SDFR1; GP	NM_01242	mRNA	turquoise
77044806-	Homo sapi	membrane	electron tr	oxidoreductase activit		NM_00186	mRNA	turquoise
16565165	Homo sapi	nucleus [g	regulation	sequence-	OCT1; OTF	NM_00269	mRNA	turquoise
15477974-	Homo sapiens BMX n		mesoderm	transferas	PSCTK2; PS	NM_00172	mRNA	turquoise
44078320-	Homo sapi	extracellul	peptidogly	zinc ion bi	MMP-9; GI	NM_00499	mRNA	turquoise
18179160	Homo sapi	cytosol [g	cellular de	electron ca	p67phox; I	NM_0004	mRNA	turquoise
13313238-	Homo sapiens testis-specific transcript, Y-lin				DKFZP434I	NR_00154	mRNA	turquoise
10187692	Homo sapiens mitoge		protein kin	transferas	KIAA0687;	NM_14568	mRNA	turquoise
30236347-	Homo sapi	membrane	leukotrien	enzyme ac	FLAP	NM_00162	mRNA	turquoise
32935355-	Homo sapi	cytosol [g	immune re	threonine	MGC7047	NM_14895	mRNA	turquoise
35908947-	Homo sapi	nucleus [g	protein kin	transferas	SFRSK1	NM_0031	mRNA	turquoise
65179379-	Homo sapi	membrane	chemotaxi	cytokine a	MGC39436	NM_14467	mRNA	turquoise
21905403	Homo sapiens MOCO sulphurase C-terminal				FLJ22390;	NM_02274	mRNA	turquoise
18576492-	Homo sapi	integral to	ubiquitin c	ligase activ	KIAA0161;	NM_18275	mRNA	turquoise
90572750-	Homo sapiens ankyrin repeat domain 22 (A				MGC22805	NM_14459	mRNA	turquoise
11411493-	Homo sapiens CUG tri		neuromus	RNA bindi	BRUNOL3;	NM_00102	mRNA	turquoise
TCCAAGTG	PREDICTED: Homo sapiens hypothetical protein LOC64					XM_93891	mRNA	turquoise
6749666-6	Homo sapi	nucleus [goid 5634] [		magnesiun	MIG9	NM_00598	mRNA	turquoise
48463849-	Homo sapi	membrane	defense response [go		PSG11	NM_00278	mRNA	turquoise
39484301-	Homo sapi	nucleus [g	regulation of transcri		C21orf107	NM_03365	mRNA	turquoise
12888239	Homo sapiens ankyrir		protein bi	protein bir	BPOZ; PP2	NM_17202	mRNA	turquoise
92520593-	Homo sapiens solute carrier family 36 (prot				FLJ38932;	NM_15231	mRNA	turquoise
19864274	Homo sapi	DNA-direct	transcripti	DNA bindi	FLJ12859;	NM_01248	mRNA	turquoise
39243212-	Homo sapiens chromosome 1 open reading				STRF2; FLJ	NM_02459	mRNA	turquoise
66859320-	Homo sapi	nucleus [g	intracellul	transferas	C15orf1; P	NM_0063	mRNA	turquoise
ATGGCTCA	PREDICTED: Homo sapiens hypothetical gene support					XM_93995	mRNA	turquoise
93094373-	Homo sapiens small Cajal body-specific RNA				Z32; mgU2	NR_00256	lncRNA	turquoise
CATGGGGA	Homo sapi	mitochond	neurotrans	pyridoxal	NPD009; G	NM_02068	mRNA	turquoise
GTATTTTC	Homo sapi	chromatin	chromatin	hydrolase	KIAA1564;	NM_02092	mRNA	turquoise
AACATGA	PREDICTED: Homo sapiens KIAA0492 protein (KIAA049					XM_94429	mRNA	turquoise
81930715-	Homo sapi	nucleus [g	immune re	calcium-de	ANX11; CA	NM_14586	mRNA	turquoise
17562982-	Homo sapi	nucleus [g	chromatin	protein-ar	PADI5; PDI	NM_01238	mRNA	turquoise
35677307-	Homo sapi	membrane	homophili	calcium io	PP791; RP	NM_18268	mRNA	turquoise
GCCCAGG	Homo sapi	chromatin	transcripti	transcripti	RBP1; RBP	NM_0230	mRNA	turquoise
16526589-	Homo sapiens hypothetical protein FLJ2015				FLJ22179;	NM_0190	mRNA	turquoise
CGCCCCCT	PREDICTED: Homo sapiens hypothetical protein FLJ141					XR_01796	mRNA	turquoise
36307365-	Homo sapi	intracellular [goid 562		sequence-specific DN		NM_03063	mRNA	turquoise
11901739	Homo sapi	membrane	small GTP	GTP bindin	RAB1C; H-	NM_00686	mRNA	turquoise

43947237-	Homo sapiens zinc finger, SWIM-	zinc ion bir	C20orf162	NM_0806(	mRNA		turquoise
GAGTACGT(	Homo sapiens ankyrir	signal tran:	protein bir	MGC1203(	NM_1827(	mRNA	turquoise
23074466(	Homo sapi	nucleus [g(	regulation	zinc ion bir	FLJ22835;	NM_0804: mRNA	turquoise
99926695-	Homo sapiens coronir	nitrogen c(	glutamate-	WDR2; CLI	NM_0033E	mRNA	turquoise
15489351:	Homo sapi	integral to	cell prolife	receptor a	CD129	NM_0021E mRNA	turquoise
GCACTGGCT	Homo sapi	nucleus [g(	positive re	DNA bindir	MGC1262:	NM_0010E mRNA	turquoise
74241769-	Homo sapiens general transcription factor II			WBSCR7	NR_00220	mRNA	turquoise
AGACTCCA(	Homo sapiens thioredoxin (TXN), mRNA.				NM_0033:	mRNA	turquoise
