# Supplementary material for: The ceRNA Crosstalk between mRNAs and lncRNAs in Diabetes Myocardial Infarction
Source: Dis Markers. 2022 May 9;2022:4283534. doi: 10.1155/2022/4283534 (PMC9112177; doi:10.1155/2022/4283534)
Supplement: Supplementary 6 — Table S6: hub genes and lncRNAs in module MEturquoise in the lncRNA–mRNA regulatory network. [file 4283534.f6.pdf]

Gene Name	Group	Degree
DNAJC3-DT	lncRNA	14
LINC00921	lncRNA	13
AC108134.3	lncRNA	9
AL445524.1	lncRNA	7
AC114980.1	lncRNA	5
CYORF15A	mRNA	4
PRKY	mRNA	4
CYORF15B	mRNA	4
OSBPL8	mRNA	31
UBE2D3	mRNA	208
UTY	mRNA	4
PPP1R12A	mRNA	156
NPTN	mRNA	58
HSD17B11	mRNA	27
TMSB4Y	mRNA	4
CIR	mRNA	108
CEP63	mRNA	34
MNDA	mRNA	182
HDAC4	mRNA	24
TLR1	mRNA	46
PHF3	mRNA	70
GNG10	mRNA	25
PSCDBP	mRNA	57
RBM25	mRNA	49
DHRS8	mRNA	58
MAP3K2	mRNA	27
ARPC5	mRNA	24
HEBP2	mRNA	23
PCMTD1	mRNA	128
SULF2	mRNA	23
LRRK2	mRNA	71
RGS18	mRNA	28
DLD	mRNA	40
LOC644033	mRNA	19
PSIP1	mRNA	20
WSB1	mRNA	17
RBM7	mRNA	19
COX7B	mRNA	18
LMBRD1	mRNA	19
AKTIP	mRNA	52
PTGS2	mRNA	45
RGS2	mRNA	70
EXOSC3	mRNA	22
GOLPH3	mRNA	28
DJ341D10.1	mRNA	28
NT5C2	mRNA	137
EXOC8	mRNA	47

BTAF1	mRNA	17
AMN1	mRNA	172
PPP2R3C	mRNA	30
LOC347376	mRNA	13
B2M	mRNA	21
ARID4A	mRNA	43
ACSL1	mRNA	19
CLK4	mRNA	157
PBEF1	mRNA	27
CBX1	mRNA	26
TUBA4A	mRNA	10
TXNL5	mRNA	19
MGEA5	mRNA	25
RCSD1	mRNA	160
LOC646064	mRNA	16
C12ORF35	mRNA	28
LOC647361	mRNA	3
RPS29	mRNA	2
MAP4K4	mRNA	38
USP15	mRNA	145
NT5C3	mRNA	20
CDC123	mRNA	12
ZYG11B	mRNA	14
SLC36A4	mRNA	17
PYGL	mRNA	82
IFNGR1	mRNA	28
S100A12	mRNA	17
LOC650919	mRNA	16
LOC644969	mRNA	12
ADD3	mRNA	19
TRIM56	mRNA	20
LOC388789	mRNA	12
ZMYM2	mRNA	68
LMNB1	mRNA	18
LY96	mRNA	14
C10ORF119	mRNA	13
FLJ11151	mRNA	10
SRGN	mRNA	28
PSG11	mRNA	11
SF3B14	mRNA	13
LOC552891	mRNA	9
NCF2	mRNA	74
RFFL	mRNA	56
ABCC5	mRNA	14
SAP30	mRNA	15
LOC730740	mRNA	9
TMBIM4	mRNA	165
F2RL1	mRNA	23

NOV	mRNA	21
IFRD1	mRNA	30
PPP1R3D	mRNA	66
CXCL1	mRNA	13
IL8RB	mRNA	22
C7ORF25	mRNA	15
MLX	mRNA	7
UNC119	mRNA	13
RPL23	mRNA	7
HAL	mRNA	17
THRAP2	mRNA	8
ENSA	mRNA	9
GPR177	mRNA	6
CHD7	mRNA	59
ZNF354A	mRNA	18
SUMO1P1	mRNA	23
RPS6KA5	mRNA	18
HIF1A	mRNA	21
IBRDC2	mRNA	11
KIAA0319	mRNA	11
LMTK2	mRNA	12
LOC652578	mRNA	22
LOC644964	mRNA	7
LOC653604	mRNA	8
RAE1	mRNA	13
GMFG	mRNA	10
C20ORF43	mRNA	90
FLJ20152	mRNA	16
OSBPL9	mRNA	16
TMEM154	mRNA	14
RNF130	mRNA	7
ASB8	mRNA	15
TNFSF13B	mRNA	11
PSG9	mRNA	5
GADD45A	mRNA	10
GTF2IP1	mRNA	14
ZNF585A	mRNA	9
TMCC3	mRNA	18
FLJ31951	mRNA	13
OGFRL1	mRNA	11
ANP32A	mRNA	12
RAB35	mRNA	13
FCGR3B	mRNA	17
VNN2	mRNA	16
ABAT	mRNA	8
LOC645895	mRNA	5
CYP4F3	mRNA	17
NUDT16P	mRNA	14

GADD45G	mRNA	7
KIAA0492	mRNA	5
RBMXL1	mRNA	8
FADD	mRNA	14
TM6SF1	mRNA	18
TLE4	mRNA	18
SRPK1	mRNA	14
HECW2	mRNA	17
MBOAT1	mRNA	11
RPS6KA3	mRNA	9
EVI2B	mRNA	14
LOC643284	mRNA	3
TACC3	mRNA	7
NFKBIA	mRNA	9
MPPE1	mRNA	7
CLCC1	mRNA	10
TRIM25	mRNA	8
PICALM	mRNA	12
HLX1	mRNA	10
PSMB9	mRNA	8
ACP6	mRNA	6
DHRS12	mRNA	10
BIN3	mRNA	7
TLR2	mRNA	12
PBX2	mRNA	3
GPR160	mRNA	14
LOC440093	mRNA	6
SLCO4C1	mRNA	10
LOC402176	mRNA	8
HSD17B12	mRNA	3
LOC440926	mRNA	7
TAP2	mRNA	9
FBXL13	mRNA	11
TXN	mRNA	9
CUGBP2	mRNA	12
SH3BP5L	mRNA	2
SIRPB1	mRNA	10
MXD1	mRNA	13
ARSG	mRNA	5
ALOX5AP	mRNA	3
C9ORF19	mRNA	5
ARID3A	mRNA	10
EPC1	mRNA	7
C9ORF95	mRNA	5
RBP7	mRNA	5
ZNF281	mRNA	3
SLC5A9	mRNA	3
S100A9	mRNA	7

LOC653505	mRNA	1
IRS2	mRNA	10
ANKRD44	mRNA	1
C19ORF40	mRNA	1
MME	mRNA	5
CKLF	mRNA	4
LOC646424	mRNA	6
HK2	mRNA	7
JDP2	mRNA	4
PPARBP	mRNA	2
CACNA1E	mRNA	5
LOC283152	mRNA	2
THBS1	mRNA	2
KIAA1706	mRNA	4
BCKDK	mRNA	1
S100A8	mRNA	1
TNFRSF12A	mRNA	1
PTPRN2	mRNA	1
CMTM2	mRNA	4
KLF6	mRNA	4
ABTB1	mRNA	2
CDH2	mRNA	2
RPS28	mRNA	1
GOLGA2	mRNA	4
CRISPLD2	mRNA	1
RPL32	mRNA	1
EIF2AK2	mRNA	2
CAMK1D	mRNA	1
RAB3GAP1	mRNA	1
ANKRD55	mRNA	2
RPS27	mRNA	1
PFN2	mRNA	1
FLJ14107	mRNA	1
ADCK4	mRNA	1
LPPR2	mRNA	1
TNNI2	mRNA	1
CLEC4E	mRNA	1
